# Transcriptomic profiling of different developmental stages reveals parasitic strategies of *Wohlfahrtia magnifica*, a myiasis-causing flesh fly

**DOI:** 10.1186/s12864-023-09949-3

**Published:** 2024-01-25

**Authors:** Zhipeng Jia, Surong Hasi, Deng Zhan, Claus Vogl, Pamela A. Burger

**Affiliations:** 1 Research Institute of Wildlife Ecology, Department of Interdisciplinary Life Sciences, University of Veterinary Medicine Vienna, Savoyenstrasse 1, Vienna, 1160 Austria; 2 Key Laboratory of Clinical Diagnosis and Treatment Technology in Animal Disease, Ministry of Agriculture and Rural Affairs, College of Veterinary Medicine, Inner Mongolia Agricultural University, Hohhot, 010018 China; 3 Institute of Animal Breeding and Genetics, Department of Biomedical Sciences, University of Veterinary Medicine Vienna, Veterinaerplatz 1, Vienna, 1210 Austria

**Keywords:** Myiasis, *Wohlfahrtia magnifica*, Parasitic strategy, Excretory/Secretory (ES) proteins, RNA-seq, WGCNA

## Abstract

**Background:**

*Wohlfahrtia magnifica* is an obligatory parasite that causes myiasis in several warm-blooded vertebrates. Adult females deposit the first-stage larvae directly onto wounds or natural body orifices (e.g., genitalia) of the host, from where they quickly colonize the host tissue and feed on it for development. The infestation of *W. magnifica* can lead to health issues, welfare concerns, and substantial economic losses. To date, little is known about the molecular mechanisms of the *W. magnifica*-causing myiasis.

**Results:**

In this study, we collected parasitic-stage larvae of *W. magnifica* from wounds of naturally infested Bactrian camels, as well as pupae and adult flies reared in vitro from the wound-collected larvae, for investigating the gene expression profiles of the different developmental stages of *W. magnifica*, with a particular focus on examining gene families closely related to the parasitism of the wound-collected larvae. As key proteins related to the parasite-host interaction, 2049 excretory/secretory (ES) proteins were identified in *W. magnifica* through the integration of multiple bioinformatics approaches. Functional analysis indicates that these ES proteins are primarily involved in cuticle development, peptidase activity, immune response, and metabolic processes. The global investigation of gene expression at different developmental stages using pairwise comparisons and weighted correlation network analysis (WGCNA) showed that the upregulated genes during second-stage larvae were related to cuticle development, peptidase activity, and RNA transcription and translation; during third-stage larvae to peptidase inhibitor activity and nutrient reservoir activity; during pupae to cell and tissue morphogenesis and cell and tissue development; and during adult flies to signal perception, many of them involved in light perception, and adult behavior, e.g., feeding, mating, and locomotion. Specifically, the expression level analysis of the likely parasitism-related genes in parasitic wound-collected larvae revealed a significant upregulation of 88 peptidase genes (including 47 serine peptidase genes), 110 cuticle protein genes, and 21 heat shock protein (hsp) genes. Interestingly, the expression of 2 antimicrobial peptide (AMP) genes, including 1 defensin and 1 diptericin, was also upregulated in the parasitic larvae.

**Conclusions:**

We identified ES proteins in *W. magnifica* and investigated their functional distribution. In addition, gene expression profiles at different developmental stages of *W. magnifica* were examined. Specifically, we focused on gene families closely related to parasitism of wound-collected larvae. These findings shed light on the molecular mechanisms underlying the life cycle of the myiasis-causing fly, especially during the parasitic larval stages, and provide guidance for the development of control measures against *W. magnifica*.

**Supplementary Information:**

The online version contains supplementary material available at 10.1186/s12864-023-09949-3.

## Introduction

Myiasis is the infestation of live vertebrates (humans or animals) with dipterous larvae [1]. During the parasitic larval stage, the larvae grow and develop by feeding on the host’s tissue. The feeding activity damages the host tissue, resulting in significant morbidity, reduced animal welfare, and diminished milk and meat production and fertility [2]. Myiasis is a widespread problem, and flies causing myiasis are some of the world’s most devastating insect pests, especially in poor countries with limited economic resources and health provisions. One study revealed that myiasis is the fourth most common travel-related skin disease in humans in non-endemic regions [3]. Myiasis can be categorized with respect to (i) the location of the parasitic site, e.g., a cutaneous, ophthalmic, ENT (Ear-nose-throat), intestinal, or urogenital site, or (ii) the relationship between the parasite and its host, which can be either obligatory, facultative, or accidental [1, 4].

*Wohlfahrtia magnifica* (Schiner, 1862) (Diptera: Sarcophagidae) is an obligatory traumatic (wound) myiasis-causing fly that can infest a range of mammals across Eastern and Southern Europe, Northern Africa, and Western and Northeast Asia [5–15]. For example, Remesar et al. examined 73,683 sheep from 122 flocks in the Albacete Province, Spain. They found a high overall prevalence of traumatic myiasis, with 95.9% at the flock level and 7.1% at the individual level [16]. Liu et al. surveyed 2038 female Bactrian camels in Inner Mongolia, China, between May and October 2021, and revealed an overall prevalence of 26.6% [17]. As a larviparous species, the female *W. magnifica* retains fertilized eggs inside her body. Once the first-stage larvae hatch, they are expelled onto or into wounds and natural body orifices (e.g., genitalia) of their host, where they start to develop by feeding on the host’s tissue. As with other obligatory traumatic myiasis-causing flies, the parasitic activities of *W. magnifica* from the first- to the third-stage larvae can lead to health issues, animal welfare concerns, and significant economic losses [6, 18, 19]. In the subsequent non-parasitic post-larval stages, fully grown third-stage larvae leave the damaged tissue, fall to the ground, and burrow into the soil to pupate. Following a period of 10–15 days, adult flies emerge from pupae. About four days after mating, adult females actively search for a new host to lay the next generation of larvae, thus starting a new life cycle. During winter, in response to the cool weather, pupae enter a state of diapause, which is a period of dormancy.

During the parasitic stages, the myiasis-causing larvae employ sophisticated survival strategies. They penetrate the host’s skin to invade the tissue, migrate within tissues, and feed on it. Some studies on myiasis-causing flies have demonstrated that proteolytic enzymes are a major component for the establishment, migration, feeding, growth, and development of myiasis-causing larvae. For instance, in *Lucilia cuprina*, in-vitro feeding assays showed that trypsin (a type of proteases) inhibitors led to significant larval growth retardation of first-stage larvae [20]. The activity study of *L. cuprina* collagenases and proteases suggested that they were not only involved in larval nutrition but also digested structural components of the skin, contributing to the formation of lesions and the production of exudates [21]. Similarly, Sandeman et al. also suggested that the proteases of *L. cuprina* degrade the host’s tissue to facilitate migration and nutrition [22]. In *Oestrus ovis*, six major serine proteases secreted in the digestive tube of larvae were involved in larval trophic activity [23]. Furthermore, myiasis can give rise to necrosis, decay, and the excretion of host tissues, creating an environment conducive to the proliferation of bacteria [24]. Concurrently, the host’s immune system is mobilized to mount an immune response to the invasion of the larvae. Thus, when parasitic larvae inhabit a wound, they actively defend against bacterial infection in the wounds, e.g., secreting antimicrobial peptides (AMPs), and modulate the host’s immune response, e.g., natural killer (NK) cells and the complement system in the non-specific immune response, or antibodies and lymphocytes in the specific immune response, enabling larvae to establish a favorable environment for their survival and development. For example, as an active immune evasion strategy, *L. cuprina* secretes a protein called blowfly larval immunosuppressive protein (BLIP), which inhibits lymphocyte proliferation in sheep by at least 70%, compared with that in the presence of mitogen alone [25]. In *Hypoderma* spp., the proteases, *hypodermin A* (*HA*) and *hypodermin B* (*HB*), are involved in coping with specific and non-specific host immune systems [26]. HA and HB induce cleavage of the α and β chains of complement component 3 (C3) in naive bovine sera [27]. HA is also directly involved in the inhibition of lymphocyte proliferation in animals affected by hypodermosis [28, 29] and the cleavage of bovine IgG molecules into Fab and Fc fragments, with a reduction in the biological activity of these components [30].

From invading to leaving the host, larval gene expression patterns are attuned to the parasitic lifestyle. Investigating the gene expression profiles, such as peptidase genes and immune-related genes, at different developmental stages of *W. magnifica*, will facilitate the understanding of the complex molecular parasitism mechanisms of *W. magnifica* larvae. Transcriptome sequencing (such as RNA-seq) is an accurate, efficient, and economical method for studying gene expression at the genome-wide level. In combination with the genome of *W. magnifica*, which we recently sequenced and annotated [31], RNA-seq technology provides an excellent opportunity for analyzing the expression profiles of virtually all genes in *W. magnifica*. The ontogenetic transcriptional profiles can then be compared to those of numerous related insects exhibiting similar or different ecologies [32–34].

In this study, we identified the excretory/secretory (ES) protein collection in *W. magnifica*, which is involved in parasitism, and analyzed their functional distribution. Then, we sequenced the transcriptome of different developmental stages of *W. magnifica* using RNA-seq technology. Based on these RNA-seq data, we analyzed the global gene expression patterns at each developmental stage of *W. magnifica* using pairwise comparisons and weighted correlation network analysis (WGCNA) methods. Furthermore, with reference to the functional distribution of ES proteins, relevant literature related to myiasis-causing flies, and the nature of *W. magnifica* parasitism, we choose four specific parasitism-related gene families, including peptidase, cuticle protein, heat shock protein (hsp), and immune response genes, and looked into their expression patterns to gain a better understanding of how the larvae regulate expression of these specific genes to parasitize their hosts. Our findings provide valuable insights for the development of control strategies against *W. magnifica* infestations. This includes guiding the selection of potential targets for vaccines or insecticides aimed at disrupting the establishment of larvae on or in hosts.

## Methods

### Prediction of excretory/secretory (ES) proteins of *W. magnifica*

We identified the ES proteins from all protein sequences in *W. magnifica* using a workflow that integrates several tools. SignalP v. 5.0 [35] was employed to identify the classically secretory proteins, with the parameter for eukaryote organisms. The non-classical secretory proteins were predicted using SecretomeP v. 1.0 [36] with default options for mammalian organisms and further filtered by NN-scores larger than 0.9. The identified classical and non-classical secretory proteins were then subjected to a TMHMM v. 2.0 [37] with default parameters to detect transmembrane (TM) helices. The TM domain was further confirmed using the Phobius web server [38]. Among classical secretory proteins, those without a signal peptide and/or with hydrophobic helices of TM topologies that were distinguished from signal peptides were removed. Among non-classical secretory proteins, those with a signal peptide and/or TM helices were removed. Subsequently, mitochondrial proteins, proteins with the endoplasmic reticulum retention signal, and GPI-anchor proteins were identified using the TargetP v. 2.0 [39], the ScanProsite (Prosite pattern: PS00014) [40], and the PredGPI [41] web servers, respectively, and subsequently removed. The remaining proteins were considered to be predicted ES proteins in *W. magnifica*.

### Functional annotation of ES proteins of *W. magnifica*

The eggNOG-mapper [42] was employed to obtain functional annotation of ES proteins. In terms of Gene Ontology (GO), the ES proteins were categorized into three high-level categories (Biological Processes, Cellular Component, and Molecular Function). The GO enrichment analysis was performed with the entire proteome as the reference group using padj < 0.05 as the threshold for significant enrichment. Additionally, the KAAS v. 2.0 program [43] was applied to map the ES proteins to Kyoto Encyclopedia of Genes and Genomes (KEGG) pathways, with the bi-directional best-hit (BBH) method for assigning orthologs.

### Sample collection of *W. magnifica*

The subject of the study, *W. magnifica*, represents a non-endangered or protected invertebrate insect pest that affects agriculture. Second- and third-stage larvae of *W. magnifica* were non-invasively collected from the wounds of naturally infested domestic Bactrian camels in the field in Siziwang Banner, Ulanqab City, Inner Mongolia, China, with the consent and assistance of the owner. Therefore, no animal experimental or ethical permits were necessary. The experimental protocols adhered to the guidelines established by Inner Mongolia Agricultural University. The collected second-stage larvae were immediately dropped into liquid nitrogen to protect the RNA from degradation. The collected third-stage samples were divided into two groups. One group was carefully placed in a foam container filled with local soil for pupal and adult fly rearing, while the other group was submerged in liquid nitrogen. Three days after pupation a part of the individuals were stored at -80 °C. After a span of 14 days, the remaining pupae emerged into adult flies and were immediately frozen at -80 °C. In total, 16 samples, with four replicates for each of second-stage larvae, third-stage larvae, pupae, and adult flies, were collected for RNA extraction, library preparation, and RNA sequencing.

### RNA isolation and assessment

The total RNA from all samples was extracted using the RNA Easy Fast Tissue/Cell kit (Tiangen Biotech, Beijing, China) following the manufacturer’s instructions. To ensure compliance with the standards required for library preparation, the concentration, purity, and integrity of the extracted total RNA were measured using NanoDrop 2000 (Thermo Fisher Scientific, Wilmington, DE, USA), Agilent 5400 (Agilent Technologies, Palo Alto, CA, USA), and 1% agarose gels.

### Illumina RNA-Seq library preparation, sequencing, and data filtering

After a quality check, Illumina RNA-seq libraries of 16 samples were prepared with the NEBNext® Ultra RNA Library Prep Kit for Illumina according to the manufacturer’s protocol. The prepared libraries were then sequenced on an Illumina NovaSeq platform, generating 150 bp paired-end reads. Using BBduk in the BBTools toolset [44], the adaptor sequences, low-quality reads, and contamination from Bactrian camel and rRNA were removed and only reads of at least 75 bp in length were retained.

### Analysis of gene expression of the different development stages

The resulting clean reads from 16 samples were used for the identification of differentially expressed genes (DEG) from pairwise comparisons between different developmental stages and WGCNA analysis. Clean data from three of these adult samples were also utilized in our separate article for the identification of DEGs between the sexes [45]. For each sample, the clean reads were aligned to the genome of *W. magnifica* [31] using the STAR program v. 2.2.1 [46], and read counts were quantified with the featureCounts program v.2.0.3 [47]. To improve the accuracy of gene expression, 5215 genes that had less than 30 reads across all samples were discarded. The raw counts of the remaining 11,506 genes served as input to the DESeq2 program v. 1.38.3 [48] in R for the analysis of DEG. A padj < 0.01 and an absolute log2FoldChange > 2 were set as cutoff criteria for the DEG assignment. The DEGs were subjected to the GO term enrichment analyses with a padj < 0.05 considered statistically significant.

### Co-expression network analysis

A gene co-expression network was constructed using the WGCNA package v.1.72.1 [49] in R to assign the genes to the different modules. In order to reduce noise and improve module identification, genes with raw counts < 30 across all 16 samples were filtered. The remaining 11,506 genes were subject to the WGCNA program. Using the pickSoftThreshold function from the WGCNA package, a soft power of 22 was determined, resulting in a network with high mean connectivity and a coefficient of the scale free topology curve of R^2^ = 0.90. The correlation network was created using the blockwiseModules function with the following parameters: power: 22, TOMType: signed, minModuleSize: 30, networkType: signed, mergeCutHeight: 0.35, and corType: pearson. The correlation of the trait (different development stages) to each module was calculated using the WGCNA function cor, and the respective significant values were generated using the WGCNA function corPvalueStudent. Gene significance (GS) and module membership (MM) were assessed for each module, where GS represented the association between gene expression profiles and each trait, and MM was defined as the correlation between gene expression profiles and each module eigengene (ME), with ME being the first principal component of the expression matrix of the corresponding module. The biological functions of genes in each of the modules were explored through GO enrichment analyses, applying a threshold of padj < 0.05. Furthermore, the hub genes with the top five intramodular connectivity degrees were selected from each module and analyzed their function.

### Analysis of parasitism-associated gene families

ES proteins are closely associated with the successful parasitism of parasitic species. The study showed that the functions of the ES protein of *W. magnifica* are primarily distributed in cuticle development, peptidase activity, and immune response. Therefore, with reference to the functional distribution of ES protein in *W. magnifica*, along with literature on myiasis-causing flies, and the parasitic characteristics of *W. magnifica*, we selected four gene families, including peptidase, cuticle protein, heat shock protein (hsp), and immune response genes, and investigated their expression levels during the parasitic larval stage to understand how larvae regulate the expression of these specific genes for successful parasitization. To identify the members of the four families in the *W. magnifica* genome, the protein sequences of those families from *Drosophila melanogaster* were obtained from FlyBase (http://flybase.org, accessed on July 29, 2023) and used as query sequences to match the protein annotation file of the *W. magnifica* genome [31] using BLAST v. 2.14.0 [50] with an E-value threshold of < 1e–20.

## Results

### Prediction of ES proteins of *W. magnifica*

**Fig. 1:**
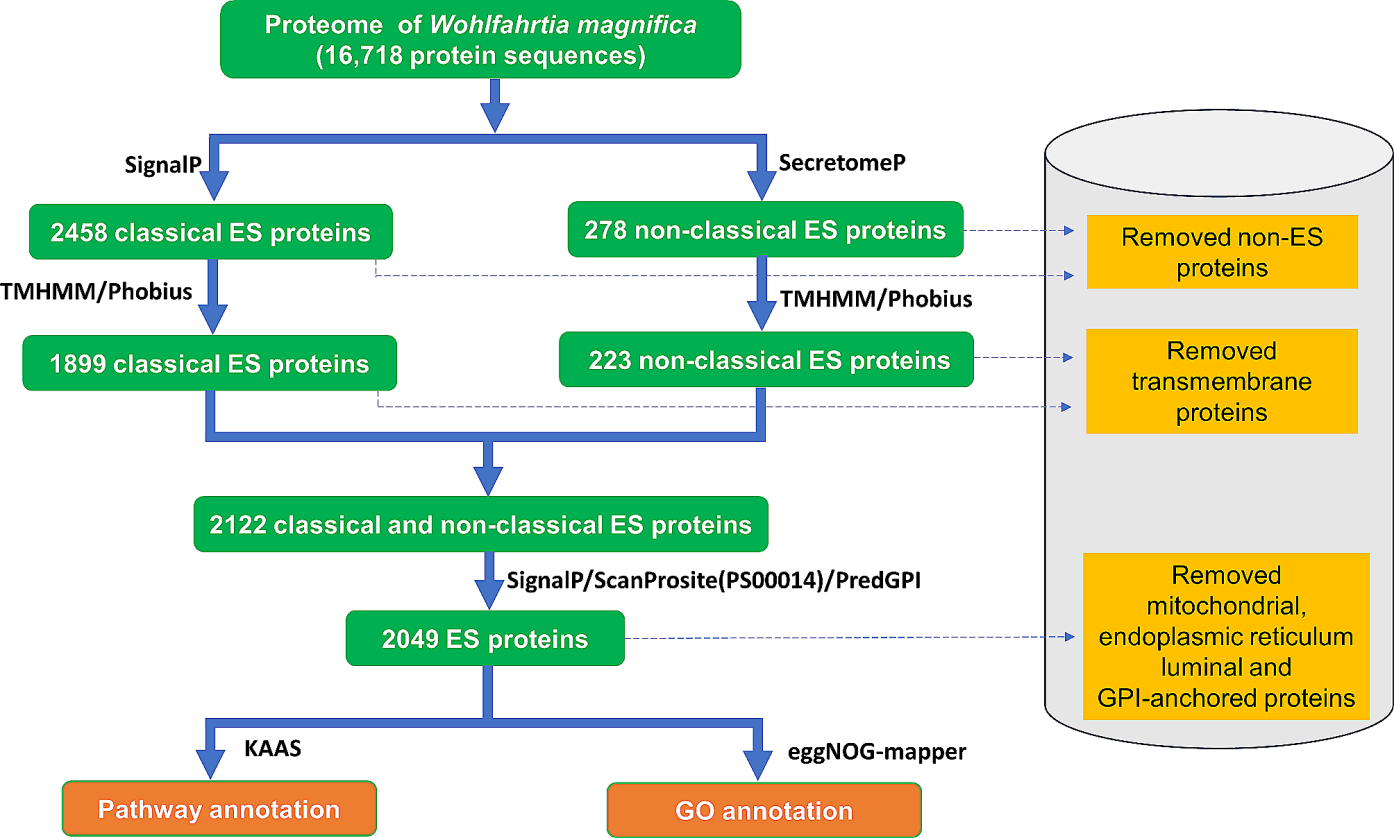
Bioinformatic workflow used for ES protein analysis in *W. magnifica*

In this research, an array of bioinformatics tools was applied to predict the ES proteins of *W. magnifica*. Out of 16,718 putative proteins annotated in *W. magnifica*, a total of 2736 protein sequences were identified as potential ES protein candidates. Among them, 2458 were classified as classical ES proteins based on SignalP analysis, while 278 were characterized as non-classical ES proteins as indicated by SecretomeP prediction. Using the TMHMM and Phobius programs, 614 proteins were excluded from this number: 559 classical secreted proteins lacked a signal peptide and/or contained at least one TM helix, while 55 proteins in the non-classical dataset had signal peptides and/or at least one TM helix (Fig. 1).

For the remaining 2122 putative ES proteins, the TargetP, the ScanProsite, and the PredGPI web servers were used to identify mitochondrial proteins, proteins with endoplasmic reticulum retention signal, and GPI-anchor proteins, respectively. Thus, one protein with the mitochondrial targeting signal, 21 proteins with the endoplasmic reticulum retention signal, and 51 proteins predicted to be GPI-anchored proteins were identified and removed. After this thorough screening, a total of 2049 proteins were recognized as ES proteins of *W. magnifica* (Fig. 1 and Supplementary File S1).

### Functional annotation of ES proteins of *W. magnifica*

**Fig. 2:**
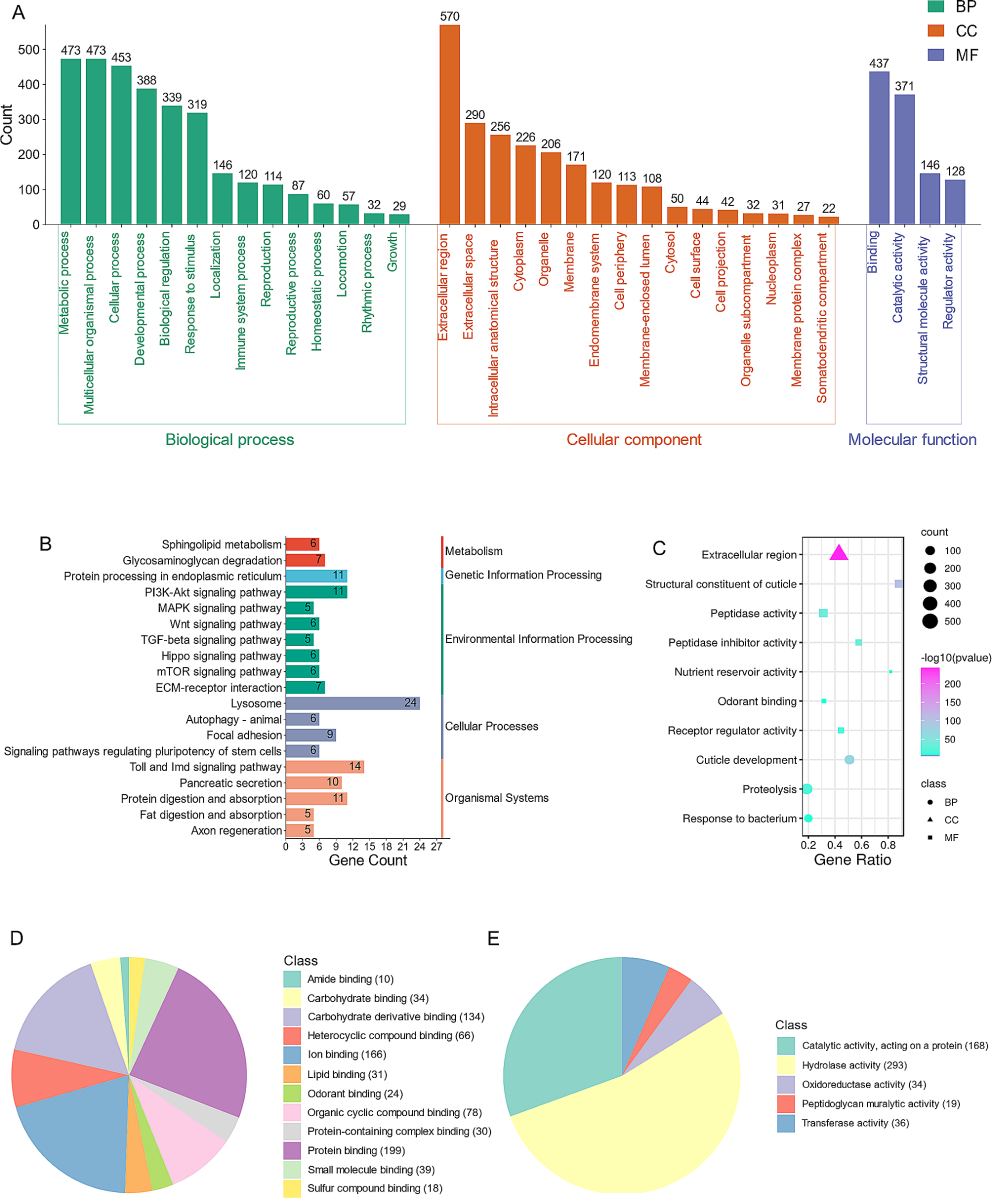
Functional analysis of ES proteins of *W. magnifica*. (**A**) The distribution of GO terms at the second level. The horizontal axis represents the names of the GO terms, while the vertical axis represents the number of genes contained in each term. Green, orange, and blue colors represent the Biological Process, Cellular Component, and Molecular Function categories, respectively. (**B**) The distribution of KEGG pathways. The horizontal axis represents the number of genes contained in each KEGG pathway, while the vertical axis represents the names of the KEGG pathways. (**C**) The representative enriched GO terms. Triangles, squares, and circles indicate that the terms belong to the Cellular Component (CC), Molecular Function (MF), and Biological Process (BP) categories, respectively. (**D**) The terms in the third-level subcategory “binding” of Molecular Function. (**E**) The terms in the third-level subcategory “catalytic activity” of Molecular Function

Among the 2049 ES proteins in *W. magnifica*, 1070 were assigned to a total of 6445 GO terms: 5179 from the Biological Process category, 504 from the Cellular Component category, and 762 from the Molecular Function category (Supplementary Table S1). Regarding the Biological Process category at the second level, the most prevalent GO terms included metabolic process, multicellular organismal process, cellular process, developmental process, biological regulation, response to stimulus, localization, immune system process, and reproduction (Fig. 2A). In the Cellular Component category, after removing redundancy, the second-level GO terms with the highest representation are extracellular region (Fig. 2A). At the second level of Molecular Function, binding, catalytic activity, structural molecule activity, and regulator activity terms are predominant, covering nearly 100% of all annotations (Fig. 2A). Within the third level of the two key second-level Molecular Functional terms, the top three terms under “binding” were protein binding, ion binding, and carbohydrate derivative binding (Fig. 2D), while under “catalytic activity”, hydrolase activity and catalytic activity, acting on a protein terms are prevalent (Fig. 2E).

With respect to the KEGG pathway analysis, 382 out of 2049 ES proteins were found to be distributed across 246 pathways. The KEGG pathways with the highest representation are illustrated in (Fig. 2B), and the comprehensive dataset is available in (Supplementary Table S2). Among them, some of the pathways are likely associated with the parasitic life cycle of *W. magnifica* in hosts, such as immune response-related pathways (Toll and Imd signaling pathways) and digestion-related pathways (protein digestion and absorption and fat digestion and absorption).

### GO enrichment analysis of *W. magnifica* ES proteins

The GO term enrichment analysis provided valuable insights into the function distribution of ES proteins of *W. magnifica* (Supplementary Table S3). In the Biological Process category, the significant enrichment terms were related to proteolysis, developmental process (e.g., cuticle development), and immune response (e.g., response to bacterium) (Fig. 2C). In the Cellular Component category, the terms related to extracellular region showed significant enrichment (Fig. 2C). In the Molecular Function category, terms associated with catalytic activity (e.g., peptidase activity, peptidase inhibitor activity, and nutrient reservoir activity), binding (e.g., odorant binding), and structural constituent (e.g., structural constituent of cuticle), as well as receptor regulator activity displayed significant enrichment (Fig. 2C).

### RNA-seq analysis

#### Summary statistics for the RNA-seq data

With the aim of analyzing DEGs across different developmental stages of *W. magnifica*, especially the parasitic larval stage, we sequenced second-stage larvae, third-stage larvae, pupae, and adult flies, with four replicates for each stage. Following the elimination of adaptor sequences, low-quality reads, and contamination stemming from Bactrian camel and rRNA, as well as the retention of only reads with a minimum length of 75 bp, the biological replicates of each developmental stage yielded a collection of clean reads spanning a range of 38 to 52 million 150-bp paired-end reads (Supplementary Table S4). Principal component analysis (PCA) revealed the presence of four distinct groupings corresponding to the different developmental stages (Supplementary Figure S1). In addition, we mapped the clean reads to the reference genome of *W. magnifica*, revealing that more than 95% of them could be uniquely or multiply mapped (Supplementary Table S4). These metrics indicated these clean reads with high quality. We noted that 1951 out of the 2049 ES protein genes had corresponding mapped mRNA-seq reads.

#### Analysis of differentially expressed genes (DEGs)

We discarded genes that had less than 30 reads across all developmental stage samples. The remaining 11,506 genes (including 1525 ES proteins) were analyzed for differential expression using the DEseq2 program in R. Our pairwise comparisons included: second-stage larvae versus third-stage larvae, second-stage larvae versus pupae, second-stage larvae versus adult flies, third-stage larvae versus pupae, third-stage larvae versus adult flies, and pupae versus adult flies.

#### Second-stage larvae versus third-stage larvae

**Fig. 3:**
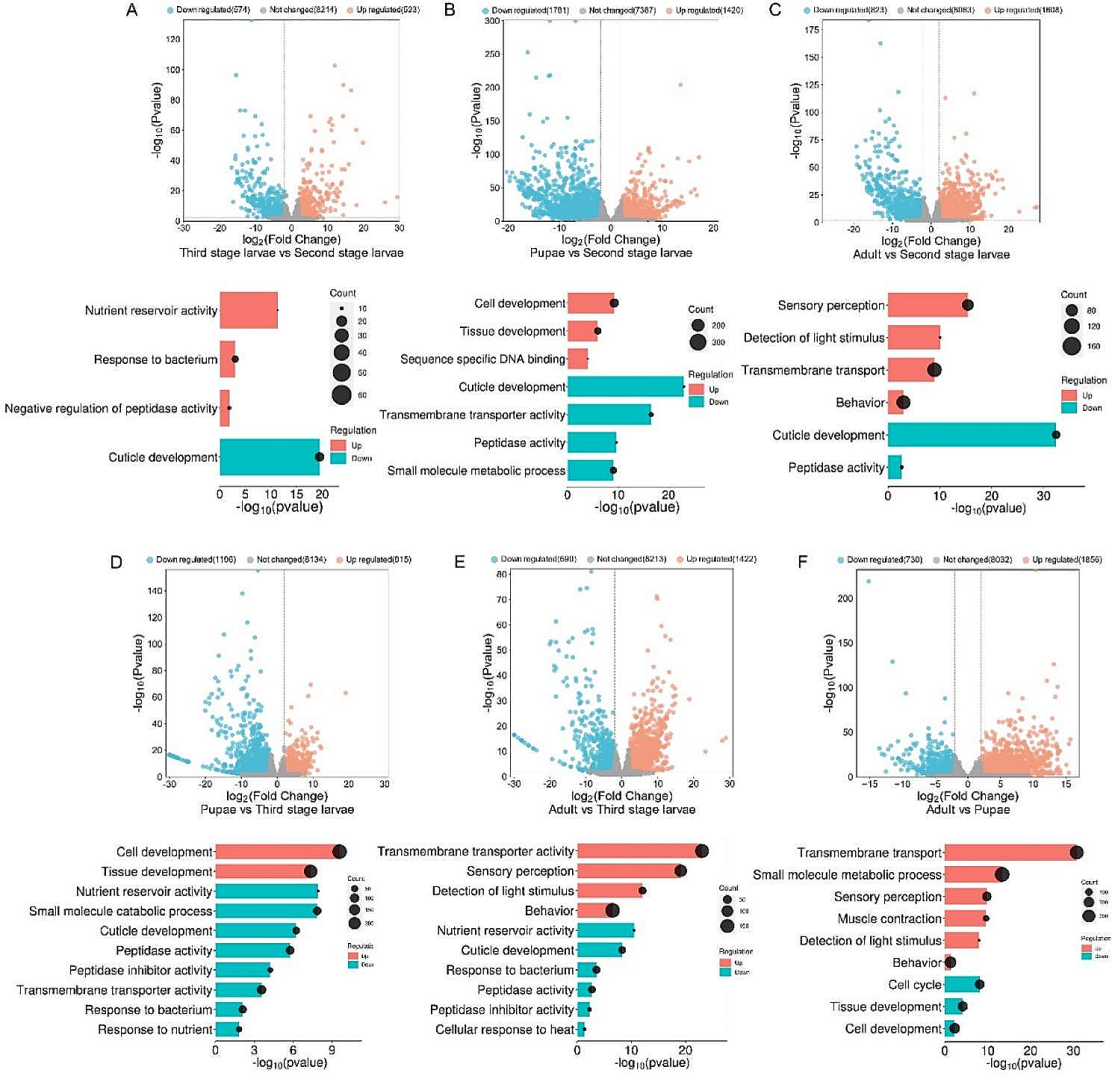
Volcano plots (top) and the representative enriched GO terms (bottom) of upregulated and downregulated DEGs in pairwise comparisons across different developmental stages of *W. magnifica*. Red and blue colors represent upregulated and downregulated DEGs, respectively. (**A**) Second-stage larvae versus third-stage larvae. (**B**) Second-stage larvae versus pupae. (**C**) Second-stage larvae versus adult flies. (**D**) Third-stage larvae versus pupae. (**E**) Third-stage larvae versus adult flies. (**F**) Pupae versus adult flies

When comparing second-stage larvae versus third-stage larvae, 574 genes were downregulated, while 523 genes were upregulated, which is with the smallest total number of DEGs in all pairwise comparisons (Fig. 3A). We performed an enrichment analysis of these DEGs to gain insight into the biological significance of these differences. GO terms dominantly enriched in downregulated DEGs are linked to structural constituent of cuticle in the Molecular Function category, and extracellular region in the Cellular Component category, as well as cuticle development in the Biological Process category. On the other hand, enrichment analysis of the upregulated DEGs revealed some of interesting GO terms associated with extracellular region and larval serum protein complex in the Cellular Components category, nutrient reservoir activity and peptidase inhibitor activity in the Molecular Function category, as well as response to bacterium, negative regulation of peptidase activity in the Biological Process category (Fig. 3A and Supplementary Table S5).

#### Second-stage larvae versus pupae and adult flies

We compared second-stage larvae to pupae and adult flies for DEG analysis. Between second-stage larvae and pupae, a total of 3201 genes were found to be significantly differentially expressed: 1781 genes were downregulated, and 1420 genes were upregulated (Fig. 3B). For the DEGs upregulated during second-stage larvae, a few top enriched GO terms are related to cuticle development. Other overrepresented terms were involved in small molecule metabolic process and transmembrane transporter activity. Also, we found interesting enriched terms connected to the parasitic life cycle in second-stage larvae, such as peptidase activity (e.g., endopeptidase activity, metallopeptidase activity, and exopeptidase activity). The DEGs upregulated during the pupal stage were enriched for cell and tissue development processes (Fig. 3B and Supplementary Table S5).

There are 2431 DEGs in the comparison between second-stage larvae and adult flies, consisting of 823 downregulated and 1608 upregulated genes (Fig. 3C). Consistent with previous comparisons, the majority of DEGs upregulated in second-stage larvae were enriched in cuticle development and peptidase activity. For DEGs upregulated in adult flies, a large portion of enriched GO terms belong to transmembrane transport and sensory perception, especially light perception. We also noted an enrichment of upregulated genes coding for behavior (e.g., reproductive behavior, feeding behavior, and locomotory behavior) (Fig. 3C and Supplementary Table S5).

#### Third-stage larvae versus pupae and adult flies

We also compared third-stage larvae to pupae and adult flies. Between third-stage larvae and pupae, a total of 2111 DEGs were identified, with 1196 genes downregulated and 915 genes upregulated (Fig. 3D). The GO enrichment analyses of the DEGs upregulated in third-stage larvae showed that some of GO terms were associated with cuticle development, peptidase activity, transmembrane transporter activity, response to nutrient, and small molecule catabolic process. Interestingly, we found that in third-stage larvae, the upregulated genes were also annotated for peptidase inhibitor activity, response to bacterium, and nutrient reservoir activity. The genes upregulated in the pupal stage were almost exclusively involved in the development of cells and tissues (Fig. 3D and Supplementary Table S5).

When comparing third-stage larvae and adult flies, a total of 2112 genes (690 downregulated and 1422 upregulated) exhibited significant changes in their expression levels (Fig. 3E). As in the comparison of third-stage larvae to pupae, a majority of the genes upregulated in third-stage larvae were enriched in cuticle development, peptidase activity, peptidase inhibitor activity, response to bacterium, cellular response to heat, and nutrient reservoir activity. On the other hand, DEGs related to transmembrane transporter activity, sensory perception (e.g., detection of light stimulus), and behavior were enriched in adult flies (Fig. 3E and Supplementary Table S5).

#### Pupae versus adult flies

Compared pupae and adult flies, we found that this transition is generally characterized by large-scale gene repression: the majority of DEGs (1856) were upregulated in the adult stage, while only 730 were upregulated in the pupal stage (Fig. 3F). GO term enrichment analysis was performed for DEGs between pupae and adult flies. A very large proportion of the upregulated DEGs in pupae were involved in the cell cycle process (e.g., Cellular Component: meiotic spindle and condensed chromosome; Molecular Function: microtubule binding, DNA-dependent ATPase activity, and ATP-dependent DNA helicase activity; Biological Process: mitotic nuclear division, mitotic spindle organization, and microtubule cytoskeleton organization involved in mitosis), and cells and tissues development (e.g., Molecular Function: sequence-specific DNA binding; Biological Process: epithelium development, sensory organ development, eye development, wing disc development, nervous system development, and muscle organ development). Among the upregulated DEGs in adult flies, the top enriched GO terms were related to the transmembrane activity (e.g., Cellular Component: ion channel complex; Molecular Function: phosphate ion transmembrane transporter activity, amino acid transmembrane transporter activity, G-protein coupled receptor activity, channel activity, and symporter activity; Biological Process: ion transmembrane transport, organic acid transmembrane transport, amino acid transmembrane transport, and inorganic ion transmembrane transport) and sensory perception (e.g., Biological Process: detection of light stimulus, sensory perception of chemical stimulus, sensory perception of smell, and thermotaxis). We also observed some GO terms related to behavior (e.g., mating behavior and feeding behavior), muscle contraction, and small molecule metabolic process (Fig. 3F and Supplementary Table S5).

### Overlapping analysis at different development stages

**Fig. 4:**
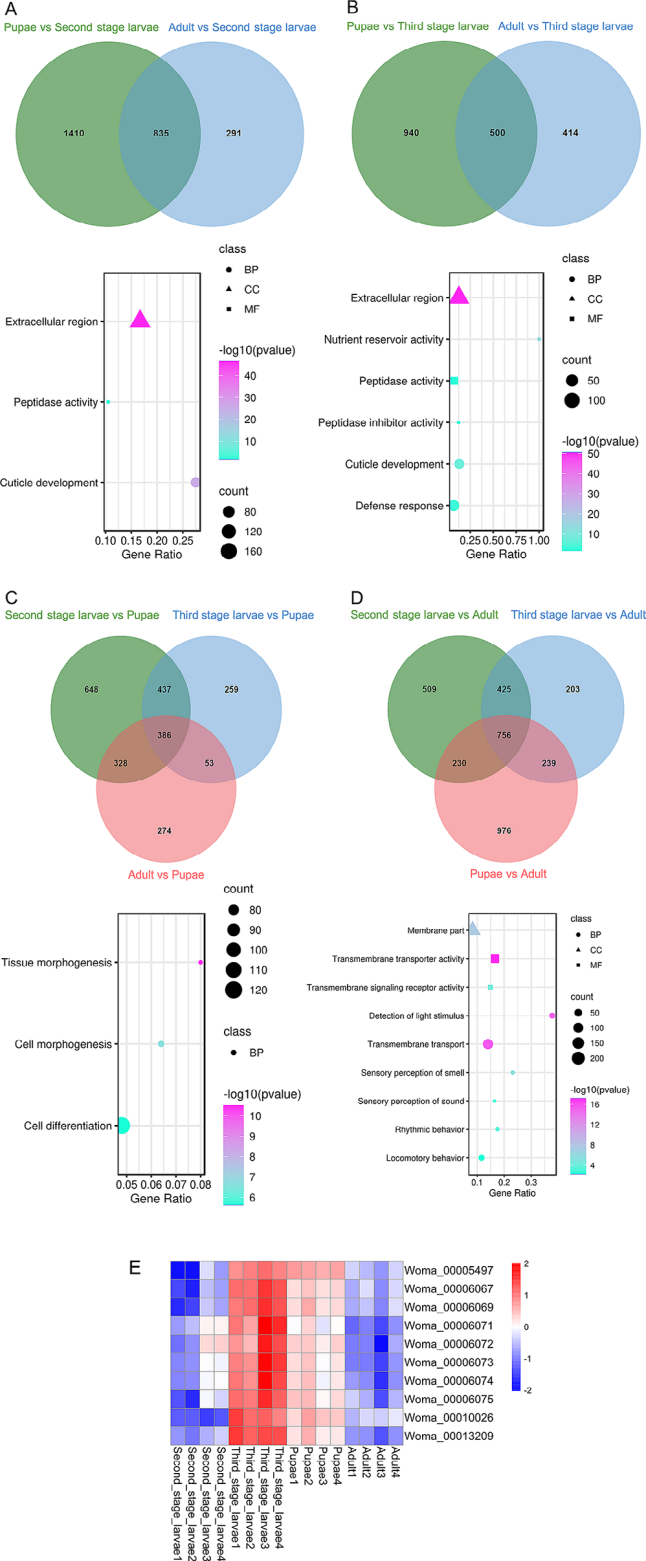
The overlapping analysis across developmental stages of *W. magnifica*. (**A-D**) Venn diagram of overlapping DEGs (top) at each developmental stage and their representative enriched GO terms (bottom). Triangles, squares, and circles represent the terms belonging to the Cellular Component (CC), Molecular Function (MF), and Biological Process (BP) categories, respectively. (**A**) Second-stage larvae. (**B**) Third-stage larvae. (**C**) Pupae. (**D**) Adult flies. (**E**) Expression level heatmap of the genes in the GO term “nutrient reservoir activity” at each developmental stage. The horizontal axis represents the sample name; the vertical axis represents the gene names. Blue and red colors indicate low and high expression, respectively.

We analyzed the overlapping upregulated DEGs at each developmental stage. In comparisons of pupae versus second-stage larvae and adult flies versus second-stage larvae, 835 overlapping upregulated DEGs were identified. The GO enrichment analysis indicated that these genes were predominantly located in the extracellular region and functioned in peptidase activity and cuticle development (Fig. 4A and Supplementary Table S6). There were 500 overlapping upregulated DEGs between pupae versus third-stage larvae and adult flies versus third-stage larvae. The GO enrichment analysis showed that in addition to being enriched for similar functions as second-stage larvae in peptidase activity and cuticle development, DEGs were also enriched for functions in nutrient reservoir activity, defense response, and peptidase inhibitor activity (Fig. 4B and Supplementary Table S6).

There are 386 overlapping upregulated DEGs among second-stage larvae versus pupae, third-stage larvae versus pupae, and adult flies versus pupae. As expected, the functions of these overlapping genes in pupae primarily involve tissue morphogenesis, cell morphogenesis, and cell differentiation (Fig. 4C and Supplementary Table S6). In comparisons of second-stage larvae versus adult flies, third-stage larvae versus adult flies, and pupae versus adult flies, 756 upregulated DEGs were identified. These genes are functionally distributed in transmembrane transport activity, sensory perception, and behavior (Fig. 4D and Supplementary Table S6).

Interestingly, we found that the term “nutrient reservoir activity” was significantly enriched only in third-stage larvae (Fig. 4E).

### Identification of modules specifically associated with different developmental stages

**Fig. 5:**
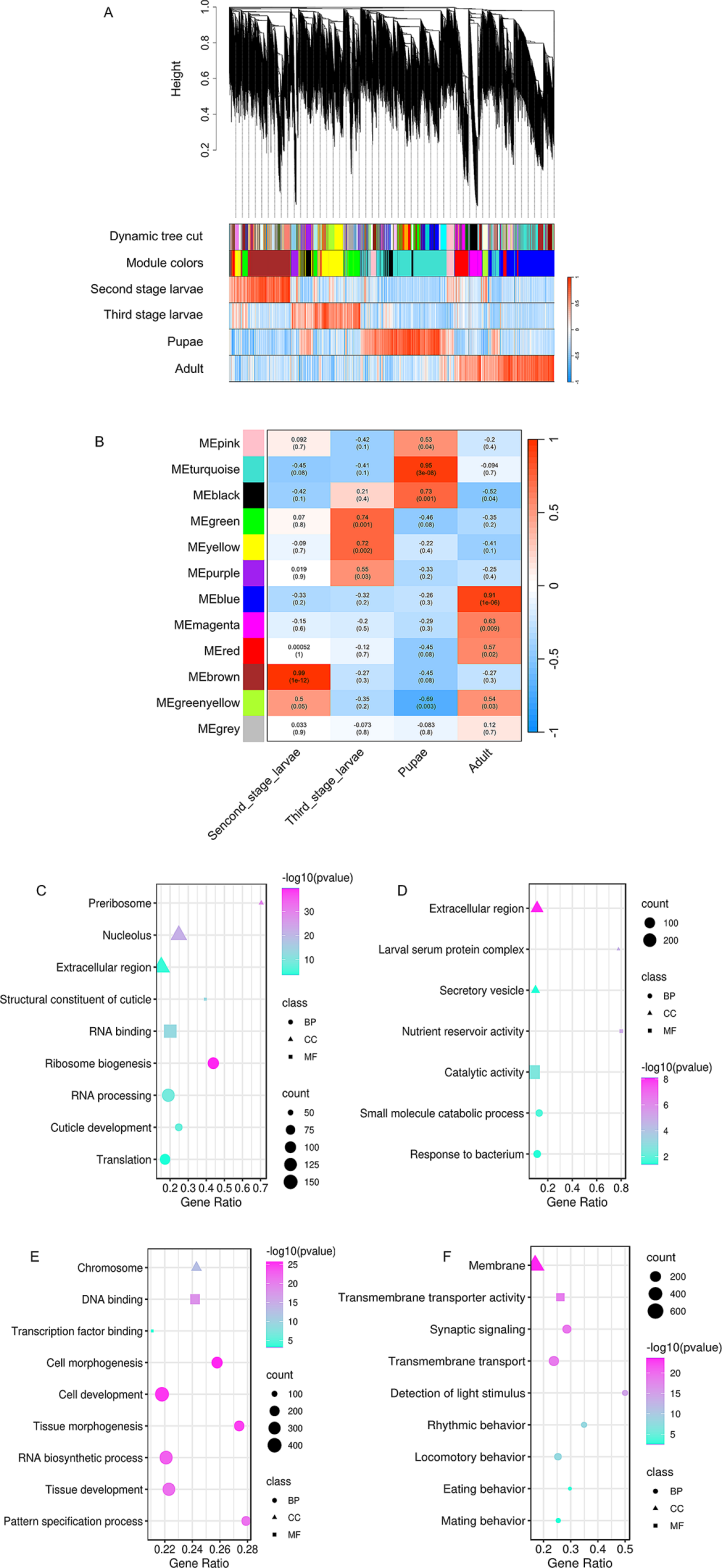
WGCNA analysis across developmental stages of *W. magnifica*. (**A**) Hierarchical clustering dendrogram of the genes. The expression distance is displayed on the vertical axis. The modules colored on the horizontal axis represent the branches of the clustering tree. The correlation of genes and modules, with red representing high correlation and blue representing low correlation. (**B**) The correlation of gene expression patterns for each module eigengene (ME) in relation to developmental stages. The rows correspond to developmental stages, and the columns represent MEs. Red represents a positive correlation, while blue indicates a negative correlation. (**C-F**) The representative enriched terms for genes in a module that exhibited a significant positive correlation with a specific developmental stage. Triangles, squares, and circles indicate that the terms belong to the Cellular Component (CC), Molecular Function (MF), and Biological Process (BP) categories, respectively. (**C**) The brown module (second-stage larvae). (**D**) The green and yellow modules (third-stage larvae). (**E**) The turquoise module (pupae). (**F**) The blue module (adult files)

A total of 11,506 genes were allocated into twelve modules, each designated with a unique color to facilitate differentiation (Fig. 5A). Among them, a subset of twelve genes was assigned to the grey module, which included genes that could not be classified into any other specific module. The correlation between MEs and different developmental stages showed that five modules were significantly associated with specific traits, of which the brown module was correlated with second-stage larvae, the green and yellow modules with third-stage larvae, the turquoise module with pupae, and the blue module with adult flies (Fig. 5B). The GS and MM analysis demonstrated that the genes in each of the modules were correlated with the corresponding stages, confirming the fundamental importance of these modules in the network (Supplementary Figure S2).

Expanding our investigation, we performed the GO enrichment analysis for genes in each of these modules. In general, the results of the enrichment analysis of WGCNA were similar to those from the pairwise comparisons. However, there are some categories that are specifically detected by WGCNA. Similarly, the brown module related to second-stage larvae is primarily involved in cuticle development. We also identified a high number of categories associated with gene expression and protein synthesis (RNA processing, RNA binding, ribosome biogenesis, and translation), which were only observed to be enriched in WGCNA analysis (Fig. 5C and Supplementary Table S7). For third-larvae stage, the most enriched terms in the green and yellow modules were nutrient reservoir activity, response to bacterium, and small molecule catabolic process (Fig. 5D and Supplementary Table S7). The result of the pupae-correlated turquoise module is similar to the pairwise comparison, with numerous enriched terms implicated in the development of cells and tissues (Fig. 5E and Supplementary Table S7). The genes in the blue module related to adult flies were engaged in transmembrane transporter activity, sensory perception, and behaviors (Fig. 5F and Supplementary Table S7).

### Hub genes within each module

We identified hub genes within each module. In the brown module associated with second-stage larvae, the top five genes in the core positions were Woma_00012859 (orthologs of TweedleG (*TwdlG*) of *D. melanogaster*), Woma_00004141 (DNA-directed RNA polymerase, mitochondrial (*Polrmt*)), Woma_00005556 (Acyl-CoA desaturase 1 (*Scd1*)), Woma_00007337 (3-hydroxy-3-methylglutaryl-coenzyme A reductase (*Hmgcr*)), and Woma_00003225 (Larval cuticle protein 65Ab1 (*Lcp65Ab1*)). Among them, Woma_00012859 and Woma_00003225 are involved in cuticle development. The remaining three genes may be responsible for the growth and development of second-stage larvae.

There are two modules, green and yellow, related to third-stage larvae. The top five hub genes in the green module are Woma_00001427 (uncharacterized protein), Woma_00008050 (G-protein coupled receptor Mth2 (*mth2*)), Woma_00007519 (Aquaporin (*AQP*)), Woma_00005609 (Lysoplasmalogenase-like protein TMEM86A (*TMEM86A*)), and Woma_00013846 (uncharacterized protein). In the yellow module, the top five hub genes are Woma_00005806 (60 S ribosomal protein L37a (*RpL37A*)), Woma_00014656 (PRADC1-like protein), Woma_00015588 (Transmembrane emp24 domain-containing protein bai (*bai*)), Woma_00005411 (UPF0587 protein CG4646), and Woma_00009113 (larval cuticle protein 65Ab1-like). These proteins serve different functions. For example, Woma_00008050 is predicted to be involved in the G protein-coupled receptor signaling pathway and to function in response to starvation; Woma_00007519 regulates the permeability of cell membranes to water molecules.

In the turquoise module related to pupae, Woma_00006124 (Serine/arginine-rich splicing factor 7 (*SRSF7*)), Woma_00010385 (orthologs of CG18766 of *D. melanogaster*), Woma_00005980 (Protein neuralized (*neur*)), Woma_00009621 (Lamin-B receptor (*LBR*)), and Woma_00010498 (orthologs of CG6163 of *D. melanogaster*) were in the core positions. Some of them are involved in gene transcription. For example, Woma_00006124, an RS protein, plays a key role in precursor messenger RNA (pre-mRNA) splicing; Woma_00010385 is predicted to be involved in the positive regulation of DNA-templated transcription; Woma_00010498 is predicted to be involved in the regulation of transcription by RNA polymerase II. Woma_00009621 is a protein present in the nuclear membrane that interacts with nucleoskeletal proteins (e.g., lamin proteins) in the nucleus to maintain the stability and morphology of the nuclear membrane. It also participates in functions of the nucleus, such as gene transcription, DNA replication, and repair. Woma_00005980 is involved in the Notch signaling pathway. This pathway is essential for coordinating cell differentiation, tissue patterning, and organ development.

In the blue module linked to adult flies, the top five hub genes are Woma_00011427 (orthologs of CUB and LDLa domain (*Culd*) of *D. melanogaster*), Woma_00012405 (Guanine nucleotide-binding protein subunit gamma-e (*GBGE*)), Woma_00010769 (Sodium- and chloride-dependent GABA transporter 1 (*SLC6A1*)), Woma_00009321 (Carcinine transporter (*CarT*)), and Woma_00002878 (orthologs of retinophilin (*rtp*) of *D. melanogaster*). These genes are related to the response to light. Woma_00011427 encodes a photoreceptor-cell enriched transmembrane protein, which is required for the endocytic trafficking of the products of neither inactivation nor afterpotential E (*ninaE*) (functioning in light detection and vision) and transient receptor potential-like (*trpl*) (functioning in the response to light in photoreceptors). Woma_00012405 is a subunit of the G protein family, which serves as modulators or transducers in various transmembrane signaling systems. Woma_00010769 is required for the uptake of Gamma-aminobutyric acid (GABA) and other small molecules, and it likely plays an important role in establishing the excitatory/inhibitory balance of the central nervous system (CNS). Woma_00009321 encodes a transporter that is involved in the photoreceptor histamine-carcinine cycle. Woma_00002878 is a protein associated with visual function and is primarily found in photoreceptor cells in the retina. It plays an important role in maintaining visual adaptation, light signaling, and retinal function.

### Expression level analysis of parasitism-related genes

With reference to the functional distribution of ES proteins of *W. magnifica*, relevant literature on other myiasis-causing flies, and the understanding of *W. magnifica*’s parasitic nature, we selected four gene families (peptidases, cuticle proteins, heat shock proteins (hsp), and immune response) and conducted an investigation into their expression patterns across different developmental stages, with special attention given to the parasitic larval stages.

### Peptidase genes

We identified a total of 480 peptidase genes from *W. magnifica*, including 13 aspartic (A), 68 cysteine (C), 182 metallo- (M), 205 serine (S), and 12 threonine (T) peptidase genes.

**Fig. 6:**
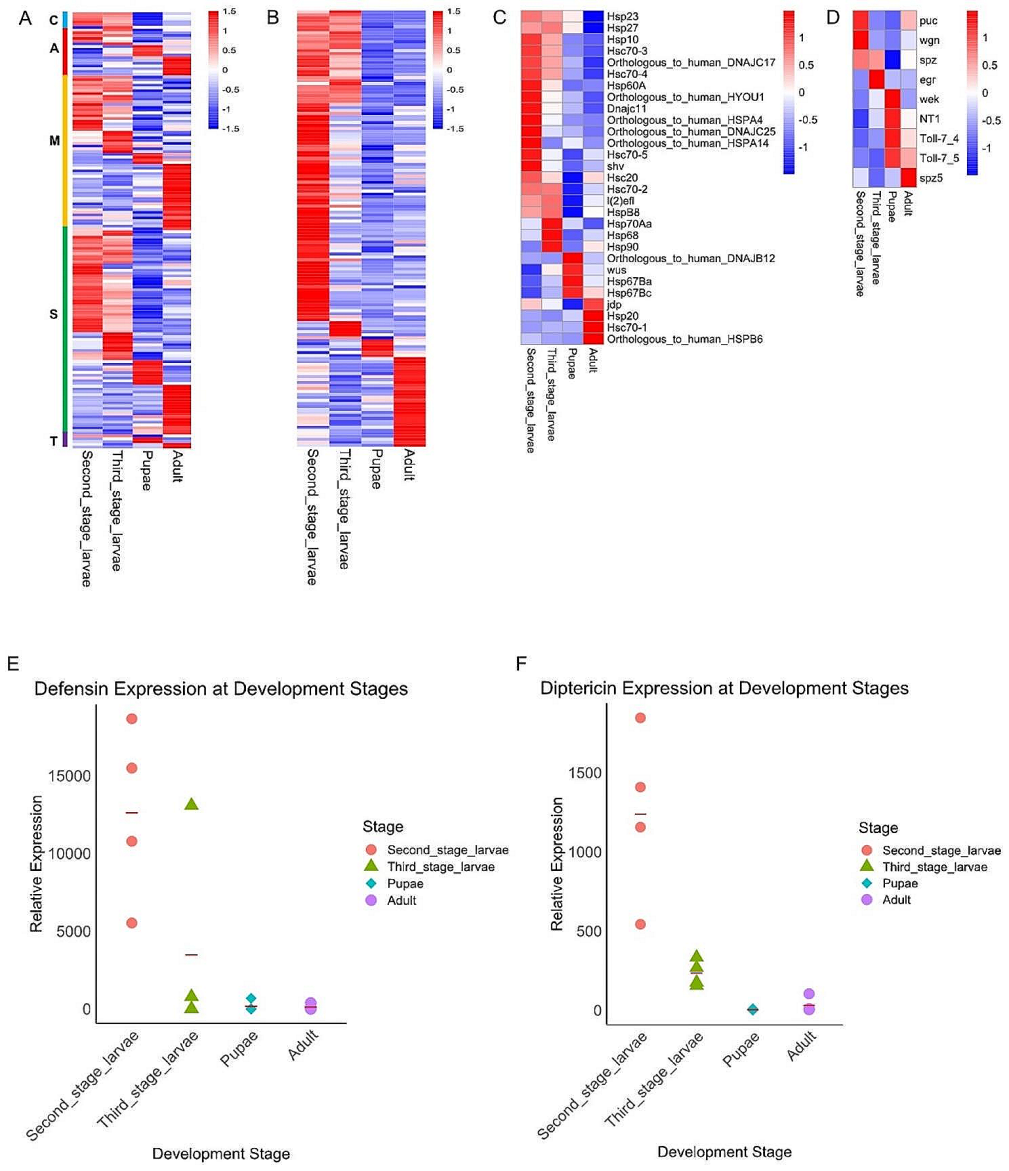
Expression levels of genes associated with parasitism at different developmental stages of *W. magnifica*. (**A-D**) Expression level heatmap. The horizontal axis represents the sample names; the vertical axis represents the gene names. Blue and red colors indicate low and high expression levels, respectively. (**A**) Peptidase genes. C: Cysteine; A: Aspartic; M: Metallo-; S: Serine; T: Threonine. (**B**) Cuticle protein genes. (**C**) Hsp genes. (**D**) Immune response genes. (**E-F**) Expression level dot plot of antimicrobial peptide (AMP) genes. The horizontal axis represents the different developmental stages. The vertical axis represents the levels of gene expression. The horizontal red line represents the average value. (**E**) Defensin. (**F**). Diptericin

We further investigated the expression levels of the peptidase genes across different developmental stages of *W. magnifica* (Fig. 6A). The results showed that 88 peptidase genes, including 6 aspartic, 6 cysteine, 28 metallo-, 47 serine, and 1 threonine, were upregulated during the second and/or third larval stages. At the pupal stage, the expression of only a few peptidase genes, including 0 aspartic, 4 cysteine, 4 metallo-, 9 serine, and 2 threonine, was upregulated in comparison to other developmental stages. At the adult stage, we identified 0 aspartic, 7 cysteine, 24 metallo-, 18 serine, and 2 threonine peptidase genes with upregulated expressions. Specifically, a number of studies have shown that serine peptidases are involved in parasitic activities, such as nutrient acquisition. Interestingly, in *W. magnifica*, up to 47 out of 205 serine peptidase genes were highly expressed during the parasitic larval stages. In contrast, 9 serine peptidase genes were highly expressed during the pupal stage, and 18 during the adult stage. For the remaining peptidase genes, expression levels in any of the three stages are not significantly higher than those in the other two stages.

### Cuticle protein genes

A total of 215 cuticle protein genes were identified in the genome of *W. magnifica*. Interestingly, 196 out of 215 cuticle protein genes can be found within the ES protein collection.

We examined the expression of the cuticle protein genes across different developmental stages, and the corresponding expression heatmap is shown in (Fig. 6B). The results revealed that up to 51.16% (110/215) of the cuticle protein genes exhibited higher expression levels during the parasitic larval stage (second and/or third larval stages) compared to the pupal and adult stages. In contrast, only 2.79% (6/215) of the genes showed higher expression in the pupal stage than in other stages, and 13.95% (30/215) in the adult stage. The expression levels of the remaining 69 cuticle protein genes did not show a significant increase at any stage compared to the others.

### Heat shock protein (hsp) genes

We identified 10 hsp genes in the HSP60 family, including 9 Chaperonin Containing TCP-1 (CCT) genes, 14 hsp genes in the HSP70 family, including 2 atypical hsp70 genes, 3 hsp genes in the HSP90 family, 1 hsp gene in the HSP100 family, and 16 hsp genes in the small HSP family in the genome of *W. magnifica*. Furthermore, in terms of co-factor chaperonins, we found 1 hsp gene in the HSP10 family and 38 hsp genes in the HSP40 family.

Subsequently, we examined the expression patterns of the hsp genes across the larval stage (second and/or third larval stages), the pupal stage, and the adult stage of *W. magnifica* (Fig. 6C). In the HSP60 family, only one gene, *Hsp60A*, exhibited upregulated expression during the larval stage compared to other developmental stages. Among the 14 genes in the HSP70 family, 9 hsp70 genes showed upregulated expressions during the larval stage compared to other developmental stages. For example, the expression levels of the *Hsc70-2* gene during the second larval stage were 390 times higher than those during the pupal stage and 21 times higher than those during the adult stage. Furthermore, 1 hsp70 gene, *Hsc70-1*, displayed higher expression during the adult stage compared to other developmental stages. Among the 16 hsp genes in the small HSP family, 4 small hsp genes displayed higher expressions during the larval stage compared to other developmental stages, 2 small hsp genes during the pupal stage, and 2 small hsp genes during the adult stage. In the HSP90 family, the only gene exhibited higher expression at the larval stage compared to other developmental stages.

Moreover, as for co-factor chaperonin families, the only gene in the HSP10 family, *Hsp10*, showed higher expression during the larval stage compared to other developmental stages; 5 hsp40 genes in the HSP40 family showed higher expression during the larval stage than during other developmental stages, 2 hsp40 genes during the pupal stage, and 1 hsp40 gene during the adult stage. There was no clear indication that the remaining hsp genes exhibited significantly higher expression at any stage compared to the others.

### Immune response genes

We identified the core genes in four immune response pathways in *W. magnifica*, including the immune deficiency (Imd) pathway, responsible for Gram-negative bacteria infection; the Toll pathway, involved in fungus and Gram-positive bacterium infection; the c-Jun N-terminal kinase (JNK) pathway; and the Janus Kinase/Signal Transducer and Activator of Transcription (JAK/STAT) pathway. In the Imd and JAK/STAT pathways, all 18/18 and 6/6 core genes were identified, respectively, but none of the developmental stages exhibited higher expression than the others. In the Toll pathway, we identified 11/12 core genes. 1 gene, *spatzle* (*spz*), showed higher expression during the larval stage than during other developmental stages, 4 genes, *weckle* (*wek*), *Neurotrophin* 1 (*NT1*), and 2 *Toll-7* genes, during the pupal stage, and 1 gene, *spatzle 5* (*spz5*), during the adult stage. The JNK pathway includes 8/8 core genes, of which 3 genes—*puckered* (*puc*), *eiger* (*egr*), and *wengen* (*wgn*)—exhibited higher expression during the larval stage (second or third larval stages) compared to other developmental stages (Fig. 6D). The core genes that remained in the four pathways did not exhibit significantly upregulated expression at any specific stage compared to the others.

Additionally, our investigation revealed the presence of 21 AMPs in *W. magnifica*. The analysis of expression levels revealed that 1 defensin and 1 diptericin showed higher expression levels during the second larval stage compared to other developmental stages (Fig. 6E and F). No significant changes in expression levels were detected in any one stage compared to the others among the remaining 19 AMPs.

## Discussion

Excretory/secretory (ES) proteins are a class of proteins that some organisms release into their external environment. These proteins are of particular interest in the context of interactions between parasites and their hosts [51]. As a myiasis-causing insect pest, the identification of the *W. magnifica* ES proteins will facilitate the understanding of complex parasitic molecular mechanisms and contribute to the potential identification of drug or vaccine targets against the insect pest, as ES proteins within the extracellular environment may be more readily reachable by medications compared to other proteins. In our study, a collection of 2049 ES proteins from *W. magnifica* (accounting for 12.26% of the entire proteome) was identified using a pipeline that integrated a variety of bioinformatics approaches. The transcriptomic analysis in the study using RNA-seq data from different developmental stages showed that 95.22% of the ES protein genes have reads mapping, indicating that the ES protein genes are indeed expressed and suggesting the potential involvement of these ES proteins in the *W. magnifica* parasitic life cycle. We examined the functional distribution of ES proteins. The GO functional annotation and enrichment analyses revealed that the top terms are associated with cuticle development, peptidase activity, immune response, and metabolic processes. Consistently, KEGG annotation assigned these genes to immune response and digestion-related pathways. These functions may be closely linked to successful parasitism of the larvae within the host.

We analyzed DEGs globally using both pairwise comparisons and WGCNA to more fully understand the life cycle of *W. magnifica* at the molecular level. A large number of cuticle protein and peptidase genes are highly expressed in the second larval stage. These genes are involved in larval molting and nutrient acquisition, respectively. Using WGCNA, we also identified a number of genes in relation to transcription and translation. In the life cycle of *W. magnifica*, growth and development of second-stage larvae are relatively rapid, which requires a large amount of protein synthesis. The gene expression and translation processes are key steps in protein synthesis, and therefore their expression may rise to respond to the cell’s demand for proteins. The nutritional reserves of parasitic larvae are critical for the subsequent utilization of the free-living pupal and adult stages. For example, in the case of *O. ovis*, low larval weight (less than 280 mg) can affect the viability of pupal and adult stages [52]. At third-stage larvae of *W. magnifica*, genes related to nutrient reservoir activity and peptidase inhibitor activity were highly expressed. Therefore, the high expression of these genes indicates that nutrients accumulated in third-stage larvae for subsequent use by pupal and adult stages. The pupal stage is an essential stage in the transition from larvae to adult flies. As expected, during the pupal stage of *W. magnifica*, the pairwise comparisons and WGCNA results are consistent with a large number of genes associated with cell and tissue morphogenesis and cell and tissue development. In the adult stage, a large number of genes are involved in light perception. In insects, sensory signals are processed in the brain by dedicated neuronal circuits to guide behavior, such as light perception [53], which in particular has been examined in detail in *D. melanogaster* [54, 55]. The high expression of these genes in adult flies may be involved in adapting to the environment, finding food, mating, and regulating the circadian rhythm. In addition, there is a high expression of genes closely related to adult behaviors, such as eating behavior, mating behavior, and locomotory behavior. These behaviors are essential for the survival, reproduction, and adaptation of adult flies. In the study, while we successfully identified DEGs in second-stage larvae, third-stage larvae, pupae, and adult flies of *W. magnifica*, the acquisition of other earlier stages, such as eggs, from the field is challenging because currently cannot be reared successfully in laboratory conditions. Our next step involves adjusting the rearing environment and the feeding composition to establish a laboratory-rearing protocol for *W. magnifica*. This development would enable us to explore the gene expression patterns of other early developmental stages. Moreover, this would allow a comparative analysis of the DEGs between larvae from wounds of hosts and those from laboratory rearing, which would provide a clearer understanding of the specific genes involved in wound parasitism.

To further understand the parasitic mechanisms of *W. magnifica*, with reference to the functional distribution of ES proteins, relevant literature on myiasis-causing flies, and the parasitic characteristics of *W. magnifica*, we choose four likely parasitism-related gene families, including peptidase, cuticle protein, heat shock protein (hsp), and immune response genes, and investigated their expression patterns, especially the larvae collected from the host’s wounds. Peptidases play an important role in the external protein digestion of parasitic myiasis-causing fly larvae, such as the establishment of infestation, wound formation, and nutrient acquisition in *L. cuprina* and *O. ovis*. In the study, we found that 88 out of 480 peptidase genes were highly expressed in the larval stage compared to other developmental stages of *W. magnifica*, while 19 and 51 out of 480 were highly expressed in the pupal and adult stages, respectively. In particular, for serine peptidases that are actively involved in parasitism in myiasis-causing flies [20–23, 56], up to 22.93% (47/205) were highly expressed in the parasitic larval stage, in comparison, only 4.39% (9/205) in the pupal stage and 8.78% (18/205) in the adult stage. These results suggest that a higher proportion of upregulated peptidase gene expression in the larval stage may contribute to tissue invasion and nutrient acquisition from hosts for the growth and development of larvae. Cuticles have a variety of key functions in the biology of insects, not only structuring their tough exoskeletons [57], but also serving as a barrier between the living tissues and the environment to protect them from dehydration, mechanical injury, predation, and insecticides [58, 59]. In *W. magnifica*, up to 51.16% (110/215) of cuticle protein genes are highly expressed during the larval stage, especially during the second larval stage. The large proportion of cuticle protein genes upregulated during larval stages shows the importance of cuticle proteins in growth and molting and may reflect adaptation to specific environments within the host’s wounds. Insects defend themselves against viruses and bacteria using an innate immune system including cellular and humoral systems [60]. We investigated the immune pathway genes and antimicrobial peptides (AMPs) in *W. magnifica*. Interestingly, 1 defensin and 1 diptericin AMP genes were more highly expressed during the second larval stage than during other developmental stages. In insects, defensins are primarily active against Gram-positive bacteria [61], while diptericin is active against Gram-negative bacteria [62]. As larval parasitism in wounds can cause severe damage to the host’s tissues, making the environment susceptible to bacterial growth, the upregulated expression of these two AMPs may be involved in protecting larvae from both Gram-positive and Gram-negative bacterial infections. Moreover, we also found several hsp genes, such as 1 *Hsp60A* and 9 *Hsp70* genes, exhibited high expression in the larval stages. The body temperature of camels ranges from 34–40℃ [63]. In the study, samples of the larvae were obtained from the vaginas of female Bactrian camels. It is possible that the body temperature within the camel’s wounds is higher than the temperature at which pupae develop optimally, thus leading to higher expression of hsp genes in response. Generally, our results demonstrate that *W. magnifica* responds to the complex environment within host wounds by employing multiple strategies involving the differential regulation of many genes and pathways. In the future, we can further explore genes within these four gene families that show increased expression during the parasitic larval stage. This will allow us to gain a deeper insight into the molecular mechanisms underlying the parasitism of *W. magnifica*. For example, in the context of peptidases, we can investigate which peptidase is involved in nutrient acquisition and which peptidase is responsible for evading the host’s immune system.

## Conclusions

*W. magnifica* is an obligatory parasite of several warm-blooded vertebrates and causes health and animal welfare problems and substantial economic loss. In our study, we identified a collection of ES proteins in *W. magnifica* that are closely related to parasitism. Functional analysis indicated that these ES proteins are involved in cuticle development, peptidase activity, immune response, and metabolic activities. An exploration using pairwise comparison and WGCNA methods revealed that during the second larval stage, genes closely associated with processes including peptidase activity and cuticle development were conspicuously upregulated; after molting to the third larval stage, gene expression patterns seem to be geared towards nutrient storage for utilization by pupae and adult flies; the pupal stage predominantly featured genes involved in cell and tissue morphogenesis and development orchestrating the transition from the larva to the adult fly; forwarding to the adult stage, genes exhibited a distinct tendency for signal perception, many of them implicated in light reception, and the behavioral activities of adult flies, such as feeding, mating, and locomotion. Specifically, when analyzing the expression profiles of genes tied to parasitism, a significant increase in gene expression was observed during the parasitic larval stage, including 88 peptidase (47 of which were serine peptidase), 110 cuticle protein, 21 hsp, and 2 AMPs genes, which may be targeted to engineer vaccines or pharmaceuticals to control *W. magnifica* or myiasis. Our findings further the understanding of the parasitic mechanism of *W. magnifica* and provide valuable opportunities to engineer control strategies against *W. magnifica*.

### Electronic supplementary material

Below is the link to the electronic supplementary material.

**Supplementary Material 1: Supplementary Figure S1.** Principal component analysis (PCA) of RNA-seq data from different developmental stages of *W. magnifica*. Red, green, blue, and purple dots represent samples of second-stage larvae, third-stage larvae, pupae, and adult flies, respectively

**Supplementary Material 2: Supplementary Figure S2.** Correlation between module membership (MM) and gene significance (GS) of the genes within each module. GS is plotted on the y-axis, and MM is plotted on the x-axis. The brown, green and yellow, tortoise, and blue dots represent genes in each of the modules. A The brown module to second-stage larvae. B-C the green and yellow modules to third-stage larvae. D The turquoise module to pupae. E The blue module to adult flies

**Supplementary Material 3: Supplementary File S1.** List of genes for excretory/secretory (ES) proteins of *W. magnifica*

**Supplementary Material 4: Supplementary Table S1.** GO annotation results for excretory/secretory (ES) proteins in *W. magnifica*

**Supplementary Material 5: Supplementary Table S2.** Assignment of the KEGG pathway for excretory/secretory (ES) proteins of *W. magnifica*

**Supplementary Material 6: Supplementary Table S3.** GO enrichment analysis of excretory/secretory (ES) proteins of *W. magnifica*

**Supplementary Material 7: Supplementary Table S4.** Statistics of transcriptome data at different developmental stages

**Supplementary Material 8: Supplementary Table S5.** GO enrichment analysis of differentially expressed genes in pairwise comparisons of different developmental stages of *W. magnifica*

**Supplementary Material 9: Supplementary Table S6.** GO enrichment analysis of overlapping DEGs between/among pairwise comparisons at each developmental stage of *W. magnifica*

**Supplementary Material 10: Supplementary Table S7.** GO enrichment analysis for genes in a module with a significant positive correlation with a specific developmental stage of *W. magnifica*

## Data Availability

All raw transcriptome data in the study have been deposited in the National Center for Biotechnology Information’s Sequence Read Archive (NCBI’s SRA) database (https://www.ncbi.nlm.nih.gov/sra), including four second-stage larvae with accession numbers: SRX21712892, SRX21712893, SRX21712900, and SRX21712901, four third-stage larvae with accession numbers: SRX21712902, SRX21712903, SRX21712904, and SRX21712905, four pupae with accession numbers: SRX21712894, SRX21712895, SRX21712906, and SRX21712907, and four adult flies with accession numbers: SRX19591857, SRX19591858, SRX19591859, and SRX21712897.

## Abbreviations

AMP: Antimicrobial peptide
HA: Hypodermin A
HB: Hypodermin B
ES: Excretory/Secretory
WGCNA: Weighted correlation network analysis
HSP: Heat shock protein
TM: Transmembrane
GO: Gene ontology
KEGG: Kyoto encyclopedia of genes and genomes
DEG: Differentially expressed genes
GS: Gene significance
MM: Module membership
Imd: Immune deficiency
JNK: c-Jun N-terminal kinase
JAK/STAT: Janus kinase/signal transducer and activator of transcription
