# Supplementary material for: Transcriptomic profiling of different developmental stages reveals parasitic strategies of Wohlfahrtia magnifica, a myiasis-causing flesh fly
Source: BMC Genomics. 2024 Jan 25;25:111. doi: 10.1186/s12864-023-09949-3 (PMC10829477; doi:10.1186/s12864-023-09949-3)
Supplement: Supplementary file 1 — Supplementary Material 1: Supplementary Figure S1. Principal component analysis (PCA) of RNA-seq data from different developmental stages of W. magnifica. Red, green, blue, and purple dots represent samples of second-stage larvae, third-stage larvae, pupae, and adult flies, respectively [file 12864_2023_9949_MOESM1_ESM.pdf]

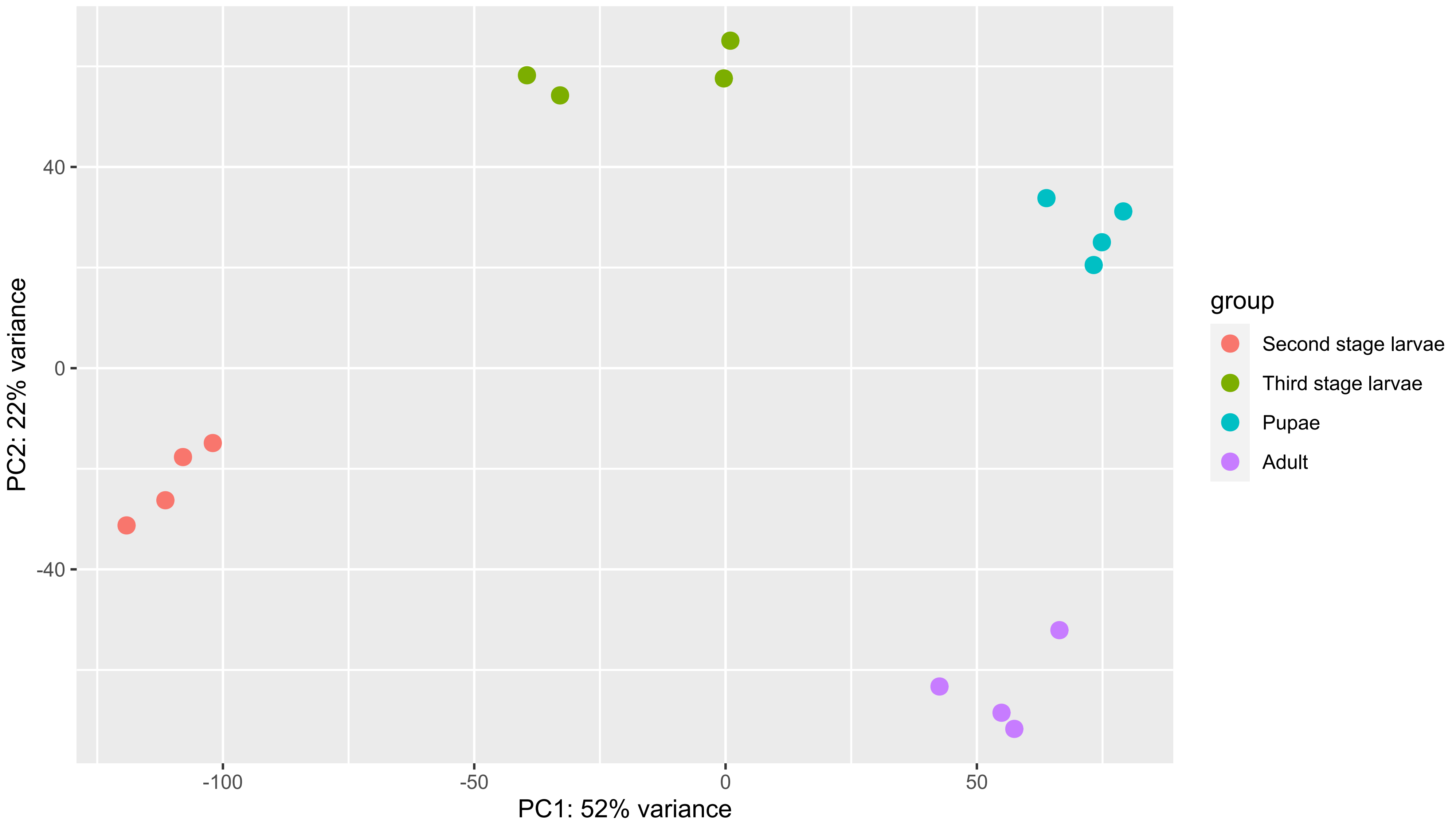

Figure S1 Principal component analysis (PCA) of RNA-seq data from different developmental stages of *W. magnifica*. Red, green, blue, and purple dots represent samples of second-stage larvae, third-stage larvae, pupae, and adult flies, respectively.
