# Supplementary material for: Transcriptomic profiling of different developmental stages reveals parasitic strategies of Wohlfahrtia magnifica, a myiasis-causing flesh fly
Source: BMC Genomics. 2024 Jan 25;25:111. doi: 10.1186/s12864-023-09949-3 (PMC10829477; doi:10.1186/s12864-023-09949-3)
Supplement: Supplementary file 2 — Supplementary Material 2: Supplementary Figure S2. Correlation between module membership (MM) and gene significance (GS) of the genes within each module. GS is plotted on the y-axis, and MM is plotted on the x-axis. The brown, green and yellow, tortoise, and blue dots represent genes in each of the modules. A The brown module to second-stage larvae. B-C the green and yellow modules to third-stage larvae. D The turquoise module to pupae. E The blue module to adult flies [file 12864_2023_9949_MOESM2_ESM.pdf]

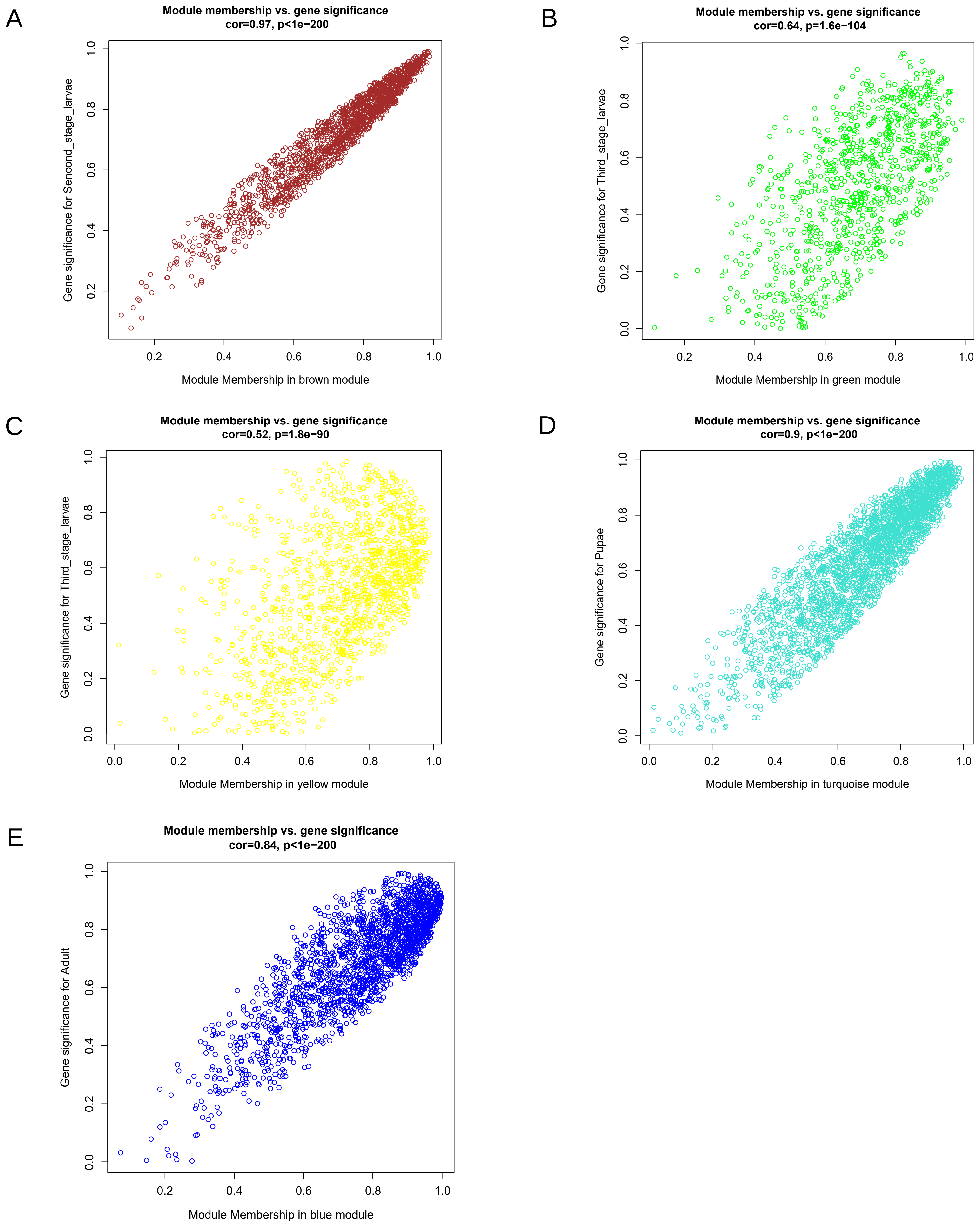

Figure S2 Correlation between module membership (MM) and gene significance (GS) of genes within each module. GS is plotted on the y-axis, and MM is plotted on the x-axis. The brown, green and yellow, tortoise, and blue dots represent genes in each of the modules. **A** The brown module to second-stage larvae. **B-C** the green and yellow modules to third-stage larvae. **D** The turquoise module to pupae. **E** The blue module to adult.
