# Supplementary material for: Transcriptomic profiling of different developmental stages reveals parasitic strategies of Wohlfahrtia magnifica, a myiasis-causing flesh fly
Source: BMC Genomics. 2024 Jan 25;25:111. doi: 10.1186/s12864-023-09949-3 (PMC10829477; doi:10.1186/s12864-023-09949-3)
Supplement: Supplementary file 3 — Supplementary Material 3: Supplementary File S1. List of genes for excretory/secretory (ES) proteins of W. magnifica [file 12864_2023_9949_MOESM3_ESM.docx]

>Woma_00014417

MERIFINFMHPLTLSLFLLVVVIHTSWSVEVKTGENLQSDTLHRQSWQNPTGVSHTVNPVSSFYYRQPKQIDERISNIIAENVLHFGHNLGLELATLDPYAEKSEIFSPLSIMTALSLLTLGAKGKSYQELKQLLAFDSDSELIESPGKYHEEFGMMLNDLENSNVNNGGINKRPNANWRFTKLKTAPVRSSGRTQPVVDHIIRVANGIFIQSGYTLNPDYRQVIESIYKSELTQLDFQNHNREARDYINGWVNKATFGKIPQIINGDIDKSTDIIIASALYFKAFWETSFFPRATTEDYFYPDGIHSPPIKVQMMPTGGIFPFYNAKEYDCRIIGLPYKGNETTMYVIQPNNSTRQRLRELQQVLNARKISDMIDNMERKTTVMVFPKMHFTRSLNMKKILQKMNINDIFVNGLSDLSLIGGKSVTNYASTPVPVPVTTSNKKTLLFEAISRQAPIQQISHLCDRFNEPALVFSSRFGEIGESNTTTNGLEMPTTTTTTVKPPLTANETKSINNREKRQVTDLHRNPIQAALFKLEADRAKFNSYQSNHFVDDIIHKVDFSVDEQGTEAAAATLTYLHRSGTDVVFRGDTPFLIVIRHDPTKLPLFYGIINKPEL*

>Woma_00014069

MVNIRELIVIILLLVSTNGHPLLPTCLGLSKWLNFIVQQPSKPFCPLDIKNKNNTIYLLYDVNPPEGFNLRRDVYIRLAVFMRQLHEQDHLPWEHFFNIESLRRYAPVLDFPEFLREYEKFGFENNERLPVHKVLQLRHFKDMFENGVFIDKWKFTDDCPQSSDVLRGSFLEEKPLWLKDSKIQCVNYQGSANLLKQVINRIVSRFEMDQMPKVIAVLNAEIVLHEHWADREFWRARRSMRFAENLMKISEEFRISNLNSTNESDKVQRPPMWEFESSQHKSKALGGSYLCVHLRRGDFIYGREKTTPTLKSSALQIKYHLQNLNLSTVFLATDASAFEIKNLKSYIPRYRVVRFTAENLYQKSVIKDGGIAIIDQIICSRARFFVGTYESTFTYRIYEEREILGFPKESTFNSLCKKSTMENCQQNSVWPIIY*

>Woma_00014072

MKHLLILFSLLAGFIQSSVYGLQFTDCGSKTGKFTKVVVSDCDTTKNECILKRNSSVSITIDFSLAEDVTAIKTVVHGKVLGVEMPFHLQNPDACVDSGLKCPLEKGETYEYTATLPVLKAYPKVNVIVKWELQDQNGEDIVCVSIPAKIQ*

>Woma_00014054

MIRHNKCGFLLIVLTTTLISLSHQQVKAELFPLSIIHINDFHARFEPTDIYGGNCDDGDSCIGGYARTVHTVKRLLEEQRSTNAVYFNAGDSFQGTLWYNIGRWNVTSQFLNMLPADAMTLGNHEFDHGVEGLVPFLETLNTTMLVANMDSTNEPELTGKYQKSMIIERSNRKIGVIGVILETTYDLANTGKLVFRNESEVIVEEAEKLKAQGANIIIVISHCGYEVDKEIARHAGSEIDVIVGSHSHTFLYTGDNPPGPDRPGGDYPTQITHSSGHRVLIVQAGAYAKYVGNLTVYFDDKGDVVDFEGAPIYMASNVPQDVEVVKALQPWKEEIDAKGKVVVGETLVDLVKNTCSYSECNLGNYFCDAMIHAYLHLAPYEDPIWSKVPIGLVNTGALRVPLNRGNLTYSHIVTMSPFENILTAFDLPGERLWEALEFGASKIDYANGVKSSYIFLQVAGLKVTYDFNKPVNERVIDLKVRCSNCSYPRYEPFNANSIYRIVAPNFLQDGGDGYYMLRDYASNVQPYITDVDALLDYTKDVTPIYHGVEDVDILSALPNWKSVIETYANKKIGTTMVNLDNSDCGYKECNIGNFYSDAMLNKLLSLSSYQSENWTNVSVAIVAAGELSLTLPEGDITYGQIYSLCPFEAPLVAFDIQGIYLKEALESSVSRIDFAGNRNTTNTFLQVSGLKITYDLNEPVNQRVLKVKVRCSNCKLPLYEKLDENKLYRIISTHYVARGGYGFAIFKKYGINKIQYFTDMSSILEYVEKMSPIYLGIEDHNFVVEALKPWRHEINTVGNRVIGYNAVYLQQKECSSGECNLGNFVTDAMLYAFLEEQNYDSPNWSNITIAFTAQSHFRVPLEPGNLTYKHLITMCPWENDIFVVTLNGSAIRQLLEECIKPLTAESNYSYRFPQVSGLKIVFNITNPVGHRIQEIKVRSQDFAVPKYVELDINRDYKMVVMDYLAHGKDGFNIIKDNAKDFRRGPGDLDALIKFIQISEPVTAGIEGRIKIVR*

>Woma_00014018

MKVFTLTLVAVLLVSATSAVKIKRGEKRVVPAEDSKTSEDEVKADITEADSDTKEKEVHKRGIFHYGGASLAASNSFWPRTYAAHYGYGWPSASGYSAAHIHSPSVLKYNTGFHYKSLAAPTVLSSGVHGFSGALPTYASLAPAVSHVHQSATAQPFIIRPGGAVVESYSVTYPRHRAAVAVKPVHAFASVAPVQPVTPVQTVTQVQAVQPVQHTFQTVAAVQPVQHIQPVAAVQPVQPVQPVAPVQPVHQLHPVPPVQSVVQVQHFKPPVHFDVTQEFPSYFQTSFVQPAPVPVHPQRPTVAFSPAQPAFPAIPTFPNPPAFPTIPAVPASPSPSLPPNSFFPVNVQPPLTQRPPSFGNINPAAQFPGFGPIAGSRPDSGSAEAAPQIPVPPSPSPEPEFSTPQPEPTQQPWKPILYQPPTVQSPVNRPSNTLLPPYGNAPAEGYLPPSHSSNNQKLQQDYTIDSSLFDNLSDADIQEIFAQANLAAQRDHQYHSRF*

>Woma_00015660

MLQMRFILCFLLVAIVAVSAITVKPLNEQGLQQSLFDADGQGHENAGERIARQYYGRRGGYYGRGGRYRGRHG*

>Woma_00015661

MKNIRKICFTFLVITVLCYVRADESITEDDSFQLRFGRRSKQPQNTSESVKKQKSQQKQEDLTAVTRSQLTPSSANELKTLQTDVESVRNLLLNEPFQVRFLDNQLNGGILQSYAFNYPYQQGYPLQQIPAMATQGYLYGNPAVVPPVDQVNNNLLGQQPQLMPHLMNGQQSIANSYAPNFIGGLSGHTFMPQQTQLPHQQNLNPAYGNNLIDTNGFPIGQPGLQNNLGLHGQPGIPPAGYPNIFNHPGQLPGMVFGPLPGATSRLPPHPDQELQFNYPLGVQQQQLLNPILGNLPSAVPPQNGFNNGIGGMLSPFIRSQRFLEQKADEE*

>Woma_00015662

MKYLCCFWLCLLWLVCNEKCGVFGLHVQQAPEIVQRITRQATRYGNAGSNYRPVSGSTSGFNVRNAASDLTASLSSSHLSSVSGNSGQRTVGVGAYSFGGMASGRPSNGNSFAVTSGSKSGALRGAQPNTVQTIVGSGSVAGSGVSVSRPQQLQQALLQAAQYQLHQQQQQQQQQQQQQQQFRRQIPQQQLYRQRPASAYQQYN*

>Woma_00015663

MKFLQACCVLFILVCSSVNIQALPVSSELAQHQVAILLPEYIAADLPASTYNPHESIVAPVPLTLLDIEKDDKSTVDAAHESQRSARGLGLGGLGGLGGLGGKFGGLGLGFKGFGGLGGWGLGGLHGLGLGGFKPFFGKHFG*

>Woma_00014139

MIWGCMSANRMGKMCFIENTVNAARYINILEKNLIPSIPNITTFGECTFQHDGAPAHTAKITKKWLSDNNINVLDWPSSSPDLNPIKSVWAVMKRRLINEPQTTVGELKEKISEIWNSITPNECKTLTNSTNKRINAVINVKGDVTPY*

>Woma_00014176

MSNQLVVSDLQRILLWNMPHALLSEQEKNPSTKVNDDGCDFSALNDTLDEIEVENYNSLETESYSDVDSEDITIVPRNRKRSKRLSISKNVIVDTSRNLSEKWIWEDKGNIREISQFGDVPAEALVNDLEHERAAEESDEIEPFADEETDVTFRMIMLHPTLKIT*

>Woma_00014195

MPKKFEGSDIVELEDEISDNFDILNDLNQSQSDIDNNQTFNAEEDNLLSENWTDTAPKNDNRPVEFRWKKNNWTEIGAHFEESLLKKNAKLS*

>Woma_00014223

MLLKLLVLVGLSHTLAALYDEEKDTIVTLPNLGSIQGKIIETAWTQREVLQFVDVKYAEPPSGKFRFKPPRPIEPWDDVMDATAEKTGCPSIVSMESIQQLDDIIDIEDCLSMTITTPNISGSYPVLVYIHGEYLYEGSNSEAPPDYLLEKDIVLVVPQYRLGPFGFLSTKTDEIPGNAGVLDIFLALQFIKFFIKYFGGDSNKVTVAGQVGGAAIAHLLTLSPKVEKGLFHQVIYHSGSALMPTFLEENPRKFAQEIAEKTDCKMKTVRDLNKCLIDMSPMKLLEAFMEHGIEKSELGVGHTGGIQFTIGGPSGVLPEHPYDLMLKSNYSYPAMGGCPKNVGSRTLNEIVENDWEGNIPDETYNSYDYIDHVIRQAVGSDKTMLLTSFVTHDFFNRKLMENGTFSTLIPRLIDVAGTLLHKLPVLLALNMNNKHVPENTFLYSFDYAGEFNRYRDMDEEHNMQSPFKAGVSLTDEALYLFPYPDHVKKLSPPDESMAKRMVDVWTSFVINGHPFGSHRSGYWPPMTTLYGPYLKLDETFTIAGNYFKEFSATIMDEEMGHSLVREVYYLRRSYTRKQQQQTSRQHISNFRKKFILNRRTPRPKLTKEIPIRKSNTKNSLIRRHLNRNKVIFRF*

>Woma_00014222

MDGIFNRILLSLTLLVLSLVIQQCWAQHNFNSDDSYDTELHQRNYGSNLNDDISNGEEGHIFIDQNQHESRIYKPYPEQPPPPPQDPNVILVPRLGQVLGHYNFKVIKNRPISAYFGLRYGHVTPGLGRFQKALSPDLSSNFINATEIPHNCPQFPDLDMIMQMERRGENVDDCLTLNIYTPAKSGSFPVMVFVHGEMLFDGSAEEGQPDYFLENDVVLVSINYRLAPFGYLSTLTEDMPGNVALSDIQMALEWIQQYIRSFNGNPEQVTLLGQSGGATLIHALSLSGKAEGLFHKLILQTGTALNPYFIDDQPLNTLRSFAHYARCPPGRAVENLLSCFESMSTSDLLNYFKRYFENNELRGLQFVSGFKLIVGDKLGYLPQHPASMVANNTKPLIVGVTKDAGAFILSRFYDQLIQLRSRNISDYINIVLKHTTKPKYYTLWKQWALQHIFTDEDVRNPSVGGLVQKLLELINLILYRGPIIDSIRFTSKNYSTYIYCFDYRGEYRRFSHLRNPLPFEIDATLSDDNIYLFPYPPEVSQLNPEDKSMARAMVTMWVNFAQYGIPNLNAGIWPNVSSEYGPFLRFTNTKESKLELDYHFGEGVPVPNLYPEYFNSTKTNTTSITTTSTTTIPPQNYDKYPQHYPRVSYPNYRQYPNYNTQPQITYRNYRSHGTALNSQRMKFPQSL*

>Woma_00014224

MFKSRIVYLILLILVICSFYVDAQQQFNNRKRGSQKIRQQQQKQQQFNKVQNNKQKGNKINKIRKEKPFLKDKNVVTVEIPGLGSVRGRTIKSEWSGKTVLQFFDIPYAKAPSGSLRFKAPVPALPWTNVLNAEKPHHGCPSLQDLVSYEKLKIKNVEDCLRLTVNTKSVTENAPVMVYIHGDFLYDGSSLEAAPGYLLEENIVLVGVRYRLGPFGFLSTKSDDIPGNAGVKDVILALQWIQDHISAFGGDPTRVTLFGQVAGAALINVLTMSPSVPDGLFHRVIYQSGSALSPAFVTDNPLPAARDIGQKAGCKNVNKIESLNKCLRKLNATELLEAFRLYSSSKQGLGAASGVIQFVVGGPSGILPQYPAKILMSGNFKTYPTMGGSVKNAGTFILKEIYFDSFNETIIDDAMNGSDYINLIISYTNGGDPTEAWKKYAEEEIFTSEIIRNGSFYAMAPGLIDLCSTIAFKNPVLLAMQGNAQKLPNSTYLYSFDYESEINRYTTTDDGDDEEEGDVEVNEMPFELGVSLTDDNLYLFPWPRYLLLNSNSDIKIAKRMVAFWTSFAATGKPTAPNAPEWPPMNDETGPYIKIGRTVSIEDNYIDEFTAAVKETKQGFNLVNEEFFDSLVEITHDSDTDDLDADGANVDGDEGRGNIVLIAKKSKH*

>Woma_00014216

MAKTTEIYKIGVVAILLLLITTSQAQRPPYAGSRPENGLNQKDKYSSTTPTNTDFQNRFGSADATPISSQIPFGQTQKPPVGYPIVYPESVYTVLTPSSTSSSGNANTGVVFEDRFGGADNTVISSNSSSASSSTTRQPGIPLDANGDQLLIDQLNRIPIDRRPFWFINYQAIESQRNGTSANFAGAQSARGSFFG*

>Woma_00014217

MAKFQFAFVLLLVVLAVFGCEAQRQRARPARYQRNRQLARQEVEEEPITPYPSADELKPVIPFDETVSADEPAIAGEDEKVVPGEGAAVGAANEPIPDEVYGPPEEAVLFNSPDEIYGPPEVEANQLPVDATLARQRQRQARLVQARRQAAYRQARLAKLRQAKPKRSA*

>Woma_00014240

MKLLIIAYVIGSISLCQSLVLSNKTLEYKNLPEVKGDKFYYFGYGSNMLTKRIHIQNPTAVKIGPGLLKDYRLDFNFYSSRWQGSAATVVEHLNRSVQGTLWEIDLENLDDIDDQEGVHQGIYKPLSVNVIRLTNNGTIPARVYVLVKQPLSDLKEFTPENIPFDRQPSKTYLQCLVKGAIETGLESPYIEWLKSIKHNGNVEKNLEKLLELENVELMS*

>Woma_00014219

MAKFVQILFVALLAVALCSAEAPRSRTSRFQAKRNRFLARQEAEEPAPVTPYPSADELKPEVPFDEAAAAEEQPSQPDEVYGPPEDGTPVETDEIAPAADEEQAVAAEEEPAAAEDEEPTPARLTARRTASIRKSSVRPAKLRKAAPARLQLQPQFVPQPLFYYMAQ*

>Woma_00014214

MAKLYTIIAIIAITLAFVVAEPPRFRQSKRFGQLRIQNNKKQFSRQEEASITTPSPAPYPPAGVTPEIPFDLPTETEKPVAEPDQTYGPPDSTPDETYGPPEQTPDQTYGPPEPTPDQTYGPPEADNVPAITEPDNTYGPPTNDDQTDNEKPEVEGLTPPEANVVSENLIQPRNNNIQSRSKSASLRSRLPSKRLRSAALRQGIVPVQPRFNTVEIIHSDPVLIYTLH*

>Woma_00014215

MAQFKKLSLVILILAIVACVVMAEPPRHRQRLAARKLQPLRQRSKVQQKRVLARQEAVTPYPTADELKPEVPFEEGNQPDEVYGPPDETYGPPELDQTPADQLPSEEAPEEFAPNPDAEEFQPTAEEEEVVPATEEQASRLTSRSKSNKKRIASRLVQKKKPQKSQRLVVAPVTAAAAPAVPVALPFTSPYAARSHQYFFLNQPFAYTAHYQAW*

>Woma_00014354

MEKVLKYLVFLFPFLLRSQAFWWGTTTTTEAPPPPTVMRQIPLTYFRTYTYQQPIGSHQLPLPLLSSTDYIANQQTLGTTPYFNPMMPLMPMVPPSLPFATSNEIPLDVRPAKSYASVNLVRSFNNIVPATTVITPEPTTTTTTTTTPAPTTTTTTTTTTTTTEAPTTVEETPITVAPFIKTDSMVNIEAAYPAFNRKVFTNHFNINYNRPIYPSPEYNTYYNTNAEDNLAAGGPNVEFVPCMCPINIAAHTGFPAAFQGASHLIGQPTNSIFLAQGRTNIDAETPNLDEPIQQQQQHQTTANVDFKAQAEVTNLLDDLHKLESRIRNNNNLAAGGPNVEFVPCMCPINIAAHTGFPAAFQGASHLIGQPTNSIFLAQGRTNIDAETPNLDEPIQQQQQHQTTANVDFKAQAEVTNLLDDLHKLESEEQESNTSQNV*

>Woma_00014349

MRLYWPYLMALIIIATNAIAWAQDFNVFAAEEEREKRAIDFGQFVRNLLLNSAAASNIKSDITRNVANLKTTTRAPRRRTTTTTTTTTTVAPFFFNKGPFKFPFDLSAPFNNKINLNSNKLSNSNNNRGYINFDYNDYGGTRQTVRPGRRRRPTTTTTTTRGTTTRAPLPTPTPRQRQPPPQPPTRRGPIPRRPYGLFGKQKVPQFQYYNDDDADDYNYEDDDDDYDTDQTNTTTSAPATRRPPPPPPSARPQRPPTAARARAQGRVPQLRNRLVYQFRQPDDTSTNNKDDNNAQPPAAAAAATAAPTEDSTTSTNNNDGEESQTENSENDDAGNNEETSVNTNDDYTDDSAGDNGDDVNNNSNTANSAQQDDNVDTNNASDDTNNNNFNFNLPSFPPFPTRNQFNYAGGNYVPNIANFGPSASFQYPSYNDYGTQYHPSAPADYSLPYY*

>Woma_00014459

MSRPHQKISTENVKANYEEFKEFGNQVLGEYLNQFTQMWSNLKPEHLEPFGKILMEHENIALKGQEELTETIRNNLQLQTQQAFNNFWHSNSVSEALISLEMCKKKFKSYEGNKWNMDRKTPWERTRPIRMHFKENRLRYLQAQLNFQNKRLEQEMEENIAQRERIQDVRNKRIFLMEAMDKYREKAQAAKSEILELQDQAFPEGNENIEPNLEVYDISLK*

>Woma_00014468

MSNDSEEEIENEIKRQKHDCGGRTTVNSNIMSGQHPNLADNSIDFDTQNFLLNSQSNCFNSANNQKNSPSVSAGRYQEDDDLIYSASSVPKEYDAVMPKIS*

>Woma_00014366

MSPESRKYTAFTVSRRGLFQWRVMPFGLHSAQRALDEVIGTELDSFAFTYLDDIVVMGAILDEHLQNLKAVFDQTKRNAPLYRQTFGIYAMSRFIPDFSMLAGPLNNLLRKWQKWEWKMEQKMAFETIEQKLMEAPALTCPDFNLLLILQKDASSVGLGVVLTQEFSDGEKVIAYASRTLSAPERNYTVTEKEWVAIVCGIRKMRHFIEGYHFIIMTDHQSLKWLNSIQLPPGRIARWALELMQFDYKIRYRKEKRQSKNRKRRKAILIEVSKPKEKNEITAINVNLPRIDIQDIVTPSVARIDPIENLTHTLGIDKEEESYSHNQIIQEILSSIQPIQPCKRTRFQTTYNGQAIEVHIRRSLDVWMIFKGEKLVYPKPKNDREKEPYPS*

>Woma_00016081

MQVKDKWDNFTELLNALVPPIRNTKEWQRVSTGGGPFTEVSLSPLEQSTVELVCMQSQVNPNGSAKGLEIQEDSDEVESIFVPLEPSTLPEQHSPERELSKSRKRSKNKLEEDRLEILKNQATNQEEILRILKRMEKNSYKNYEINKKCMN*

>Woma_00014530

MVSLDRKKIYIKRKYLQRLQQTHLRLQSEHFFLECNKYQTDIYRSRDILLNIFESCQDCKKMATLPQGAFVACKLREQCRERDLIVSRLKKSNGDKTDAKFFNGNKKLLEYSPLKYNEKLMNSIWGFYNRYSPHNIKSNEIVSYNHFYNQQQPSAVANQAIAHNIAMAVTTGKEWNLEGKK*

>Woma_00014544

MKVSLKLFFHLAIVVIIVILNCLATVQAKPKANLFNLMGMDEHPEKVPEFDSAGLRMRRSLDPRVADEGDVPKGELNSDSHLRNRRQAPPGESDAPKGLQQEIEKLRHKRQMPSPPGEMPMPPM*

>Woma_00014596

MPQDTFLERAGKEERQCTGGTTTSQGQDASAVNVEEDSLGPDDEAMPRTKETSIERQRGNCTGKFSIARKGHGSRCEKILTQILGEWVIKLLWGNSALEAPLQ*

>Woma_00014589

MRPTTGETLRAWLLSALMVHGAVAGNPDAKRLYDDLLSNYNKLVRPVVNTTDVLKVCIKLKLSQLIDVNLKNQIMTTNLWVEQSWYDYKLRWEPKEYGGVHMLHVPSDHIWRPDIVLYNNADGNFEVTLATKATIYSEGLVEWKPPAIYKSSCEIDVEYFPFDEQTCVLKFGSWTYDGFKVG*

>Woma_00014606

MLSPDMSQVASWHTPSVYSAASSFRSPYPTSLPINTTLSSDFPFRFSPSLLPSVHTSPHHMLNSHSAIVASGAKQESQDNTGNHRFSRELVGQNMYDSYTVS*

>Woma_00014626

MSNPNNLVQPPRLVVPGTSSQAANESTPRPSVQPVQSIPLTQMTALISIRLMPPRSSMPKPQERPEQMDIDMSTRSNRVNYMNRPHPNDLYGKRPVPPSFRSNGNKMQRNYHMDVNHTMDAPCDDECPLTNTEFEQAQRYFRDVPKEVATQEEQPVDFNDLHF*

>Woma_00014671

MKIVLVIIKLLFICLKGIKTDPDWTFEILELDCLNNTPDILNISFYLVRISRGVFAVSGNFSVEQDLTDETTTVGDLFYSATGHNFIRTAFHIPEVVLSESVNTFYRDLLLDSLWNCTENAPLIATDDPFVAPLTKRVIKADKCFISNSNMPSFMRPGYYKGVYAFKKQADSVITGIFKIDKK*

>Woma_00014669

MKIVIVIIKLLSICFQGIMGEPDWTYYIDELDCTNKTPHILNLTCTLDRISRGVFAVSGSFLIAQDLTDETMVSGELFYSAMGHTYVKTPFHIPEIVLSEFINTFYRDLFLDSLKNCTENAPLIATDDPFVAPLTKRDFKLDKCFFSNSNMPSSMRPGYYKHIYAFKKQVDSELTVITKIESK*

>Woma_00014668

MWSLISVIILLFEYSLSPVQSEATWTYDLISIEVENHTPEVVNFDLYETRVSRGVFALSGTLDIKQDITDDLIVSMTTYYSSSGQNFAKAAFFIHDTKLSDCLKNYYHLVMADLERCSENLPKYDENFKLPLKPCTIKFNKCLLNTDDLPPKIRNGFYKVVLEFKALAKTSITYTIQVFDK*

>Woma_00014731

MQLFVRGLECVETLEVYQDANIADVKAQIAQLHGYNTEEITLNCEGNALANETPISALSSFELDITVPIMGGKVHGSLARAGKVRSQTPKVDKQEKKKKKTGRAKKRMQYNRRFVNIVPGSEHRGPNSNSKL*

>Woma_00014734

MQLFVRGLECVETLEVYQDANIADVKAQIAQLHGYNTEEITLNCEGNALANETPISALSSFELDITVPIMGGKVHGSLARAGKVRSQTPKVDKQEKKKKKTGRAKKRMQYNRRFVNIVPGSEHRGPNSNSKL*

>Woma_00014725

MRYFILILFVSSVLTDDKVSKIDEKPSDAANDLKQSVEEPKSKTEKRDSSVHTSSFGINSNAEPFKPMLNPPVKYETAASSNQQVYYNKNQVTAGNAKAAVNTQTANAYREQYSQQQQQQQQQPPLPPPPSPNKNQLRAAVKNAGAQQQIQQQVLYAVPQNPPNHYERIPQQQQQQQQQLQQQQQQQQYQQQSHQLPVPRAPQKQIKYVIAIPMSYLRQLQQELVHHQQQQQEQPSNLQLYKGQQQQQHQFQLHLQPPPQQQQQQPIYSIAGPLARDQQGAYRPFHRFALTAVNDVSTGGIQHHQIGSPAPIPPPALAGAPSSPGYVTQYVQIPASVLMSALQSAQVQQQQQQQRPVHLAQPVYPQQHHQPQQLPQPLQQQQQQIQLPLQYQQPIQQQQHIPVPASQIFYLHHQPQQHLQPIQLKQSLPSQQLTSPPVYAYHVNPLHQQQQQLEQSNQQHQSYLTEQQQQQLNVGKQPLRVRGVRPNVNLAPSQYDTSAATYEHEGKIQLVSIPTPIQTHAHTYPNAHGQQYIAQDPIAQQAQAHQVVQAQQLQAPEAYEQVVHHYSPQYVQQHHVGQQPQQQQYQSHQPQQHRLQVQSPPPAPPSSPTPPPPQHSTPLVEHLNNLPFSQGIHGPTPLPFLRLHQSTGVAPFYNFNVVPPTVPQPFALQSPLNPLGIPFGGHPSQYPAQEINSAALANSADTVPYFHQIGGIRYGTQVFHPPTSGAIFLPGSKPTTSTVSHPSPRVSNVANEQKIAASSAKSAISSVAGSPTIVKYP*

>Woma_00014728

MQLFVRGLECVETLEVYQDANIADVKAQIAQLHGYNTEEITLNCEGNALANETPISALSSFELDITVPIMGGKVHGSLARAGKVRSQTPKVDKQEKKKKKTGRAKKRMQYNRRFINIVPGSEHRGPNSNSKL*

>Woma_00014756

MVVVDNEKALNSESILFILDDQLNTKDFQTPPYISTVNGQVERFHSTLSEIMRCLKTEKIHRSLEELLVRAVYEYNYTIHSTTGRKPIEMFFGRRVSTNSEQYEKARIENIEAVFKKPKNDIEYHNKSTF*

>Woma_00014767

MKIGLLCGSLLVIVGLISCALGEVNVVTRLEWSAKEPVNELTTLELPVGRVIIAHTAGNVCNTKDLCAQQVRNIQNFHMSKYHYDDIAYNYLIGNDGNVYEGRGWKYQGALVKGYNAGSFGIVFMGNFIKELPSQNALTAAKELLGELVNLQKLKEDYKIYAHSQLMATISPGEALYAEIKTWPKWSNQV*

>Woma_00014765

MEANLIKKMLCSCRSKITNKTQIQHLVTIMEANPGIVCGFYKGNKDTLARFWKNAEIDLNSMGSPSKCAGVGRPKKQNEIGKIKSTGGGLNKENTFSPTEEYRT*

>Woma_00014814

MLINFWNFKLFQVYLLLFVSRVTEISADKKLPLVINTWAFKEANEVAWATLLQGRSALDALVAGCTTCEEQQCDGTVGYGGSPDENGETTLDAMIMDGTNMNIGAVGGLRQIKSATKVARMVLEHTKHTLLVGDAASNFAEMMGFRRESLTTPKSKKIWQDWRNNNCQPNFWLNVIPDSSKQCGPYAPHHKWLYENVERYSYKVDQWHHDTIGMIVIDSSGRIFAGTSTNGASHKISGRVGDSPIPGAGAYADSEVGAAVATGDGDVMMRFLPSLLAVEFLRQGQGPEEAAVRSMQRIVKYYPDFSGGLVVVDRWGNYSAACVGIQQFPYSVAYGNTNTVIEYKLCIHPEIKRTDLKPIYRYCI*

>Woma_00014833

MNYLFTFVAVFSALTATAVGTTFTRCSLAKEMFALGVPKSELSQWTCLAEHESSFHTHLIGHANSNGSRGYGIFQINNLYWCQANYGPSFRNGCKVSINASVTCARKIKAKQGWKAWSAWKYCSGTLPSINDCF*

>Woma_00014836

MFFNIILLLALDLRILAFNYSPYGEENQGNPVVVIDYNTMGQGVCPQIKQPLCATNGQQFSYFGNRCQLEVKNYEQLLQGLQEFYECPLSYCSPQCAPCGLVDEPVCAIDIQTQQRETFANPCEVERKTCTTQKVYSIIASGACPQISAYELKCPQFCTLKYNPVCAQYQYEIREFPNECELQKEKCETQKNWQMIDLSNCAFTKGNDVIEPLELPVTKSECPEFCTLEYKPICARFMSLEKEFGNECELQTTICQTKQEWTIIRQGPCVQREKETALISTTKQQCPLFCTLEFSPICAIFNNELRQFSNRCALESTICQTQEKWEILFNGTCLAFVELNNTKCPEICPLVYRPICARLEGVLREFGNDCELQATIYQTQEQWEVVYNGTCLSYNAECPQFCTLEFNPICATFNGELREFGNNCELESTICQTQEKWVIVNQGPCLVKTHAKSSNCPQFCTKEYVPVCAEFNKELKEFSNKCVLLQTICETNQKWEVVNLGPCSNECPKFCTKEFEPVCAVYNNVLQQFSNKCVLEKTKCELDQDWKIVNMGLCPLKSEKSSIQLPTSCPLFCTKELNLICAKYGQELREFDNKCLLTKTICETGNEWEIVKQGSCSTEASCFKICTDDFVPVCAEFGNEFREFTNECDLERTICETNTPWKIVSLDLCSSNSGNSKTVILGPVCASNGKENKTFKSILELPGVDWIVIQQGECYATIDTKTTTGTTPKTTTQKSAVKYSTKTSTTRATPETTIKNKYTNTTTTSVPKTTSSQKTTQHTTKKPSVSTQITTLITTPNSTTTTSTYKTTKYTTTSTEKPTTTAKTTTTTKHLYDFIKSSEDCSNEHLNCCNCKSLEEIEPYSEESFEDDLDLSWESEEDEEDNIYIRALMNNKTDANVNSYTQKQNNSNDNGYLKENNKEKLLEENTYKENTANSTETSKPEEYVTVYKKSHSEKSSQEDNENLKHYSAEGRGSLTSTSFIIKNPVSYNNKPTDIQQTSAINVVEPIISNKDKIFHSDGKVEEHNTLQNQATFKKVESNDIQPKLPYKPNYDSAVRKENEPKFGVFSNEAYSLNSPYQENSHKNENIQVLDYAKSSSLVNDKVDAEEKFLKAYEVLLTDSDSEEDEEDDIALVNKVLTTLATEKTSAAYPIFEEHESQLINHTKRQEYTAEYNTASNSKKGEIYNGAVYKAPVYDAPTYNFIYTAPNEPIFSWLAPGILTNSHHLQHKTAEAVAEPNVSFNPMQFLNSLASAQHFSSSTEIRNQEPNKHLSVFGQHQPLYPYTGTSPFTFSYILPQMPPIAPQMMSPPITNAFLPTKSAVSKVKFPLAATEYQDNYKISQQNDYQKLGNSKYLPSFTFSSNTKPYQTFHPLMSASEQKNIAISPLLQRDLKHVPYDFLNSHNSAFNSTVFKQILENLKTLPKQS*

>Woma_00014837

MKVFLLFLGFILFTAAQNSKNLIEDLNNIEICATLNNVNRTFTNIWEIYEEFKKTGDVWTPVARGTCVTNNLDCMILNPVCAITNNFFQKTFPNKCVLEADQKNSGIQWHVIYNGICRGDGFITPPTAPPLPVPQQSHKQTPDPNLPYSPSRSLNAPAALPTNKYSSQALVNPEPLPLKIPAPTTNAYSPPIATPPAAPVPLANNYEQPSNEYLNSVPPQESSQSSAISQLYEALVNYYESLPKQESLYPGKNDKNLLPVPSSAPTVTSPPSYINVIPNYSNDVLSAQPNDASSKGYKQPEAPSYSDLIEIPSNNYENDFQQPPAEQSYAAPVNQPEYEISSKNYGANADSAYVPSANSNNKVNTPAKNYETAPYSGPVTNPFPLNPSNSYEVPYAAPDTQISYANSGNNEDSTSSVKNYEAQSYAVPSENSSSIPAKSYEPLTYAPASVDSVAGEIPSPTSCTNLGKNYETLSNPVAVENPLPSYTNPINSYEAPSYATPIENPPSSYPGSALNYKSIPNENPSYSYSSRDDNFEVPSYAANIETLSNSHKNYAAPAEEPSPSAPSYEEIESLPAEAPCSNADSTYASPVLDYNSLPSYSQKTYEAPLISDSDYNSKYENSDASQPEAPISDNAESAYNSPAEESEQNLHADGQTVYNGVVYQGPYHYEPKYKHINYGNFNFLPAQSPKNDEVAPTAPALIPAYTYANALPASQYNAFSSNPFEQLLQYIASRNK*

>Woma_00014835

MKVLYILLAALALAAPALSRTLTRCSLAREMYALGVPKSELAQWACIAERESSYRTHVIGRTNPDGSYDYGIFQISSLYWCQPSNGRFSSNVCRLSCNALLTDNISNSVSCARRISIPSFPALLRLDFTSIGKRFAKVIGQQGITAQFAFIVGETAIGWLTPVVVVDLENAIVVRAIRVDWANNIDAALAAPALSKTFNRSLAREMHALGVPKRELPQWTCIAQHESSYHTNVIGPTNSNRFNDYGIFQM*

>Woma_00014801

MLKFVILLSAVACALGGTVPEGMLPQLDGRIVGGAATTISSFPWQISLQRSGSHSCGGSIYSNNIIVTAAHCLQSVSASVLKVRAGSSYWNSGGVLVSVAAFKNHEGYNAKTMVNDIAVIRLASSLTMSSTIKAISLASSAPANGAAATVSGWGTTSSGGSSPAQLRYVDLKIVGRTQCASFTYGYGSEIKSSMICAYTVGKDSCQGDSGGPLVSGGVLAGVVSWGYGCAYANYPGVYADVAALRSWVISAAGSV*

>Woma_00014798

MLKFVILLSAVACALGAAVPEGMLPQLDGRIVGGVATTISNFPWQISLQRGGSHSCGGSVYSNNIIVTAAHCLQKVSTSVLRVRAGSSYWNSGGVLVGVAAFKNHEGYNPYTMVNDIAVLRLSSPLTFSSTIKPIALATTAPANGAAASVSGWGSTAFGSSFSPSQLRYIDTKIVSRNQCASSRYEYGSDIKYSMICAYSVNKDSCQGDSGGPLVSGGVLVGVVSWGEGCAFPDYPGVYADVAALRSWVVRNAQSV*

>Woma_00014793

MLRFVALFAFVSCAFAGTIPDVLDGRIVNGVDTTIQAHPYQVSLQTNSGFHFCGGSIINEDTVVTAAHCMQKYKAHEFKVRLGSTEYNNGGELVAVKAFKYHELYDSETMVYDVAVIKLATPVRESSKIRYVKLAEKTPATGTTAVVTGWGATCFLFCQTTPKVLQKIEVDVIDENVCASSEFKYGDQIKETMICAHAVRKDSCQGDSGGPLVAGNELVGVVSWGKGCAFKGYPGVYCDVATVRSWIEKTAGQL*

>Woma_00014792

MLSFIVLFTFISCAVAGIRSNDFDARIVNGVDTTIQAHPYQVSLQTNWGLHFCGGSIISKDIVITAAHCMQTYKAHEIKVRLGSTTYDKGGVLVAVKDFKYHELYNPRTMVYDVAVIKLANPIRESSEIRYVKLAKKEFATGTAAVVTGWGSKCFFFCPTIPKILQKVEVDIVDEKLCASSEFRYGDKVKETMLCAYAVKKDACRGDSGGPLVANDELVGIVSWGIGCAFKGYPGVYCDVATVRRWIEKTAMEF*

>Woma_00014775

MLSNGLKQTRILKALYLLLIVLTLNRSTEAALFDGCDHIYNISPGYSYLESPYYPKSYPAGTSCRYKFVAPLDYEISINCTIKLDKGPNACSTEFFYVAKDGDHLLRDSEQFCGSGTFIRKTLFRSGVLAYVSGGSFGSFRCQLYARPQPCDCGWSINTKIVNGQETAINEFPGMVAFKDTSTNLPSYCAGAILSHRHVISAAHCYAIQPNPGYIRALVGHHNLKAGSNTKYAAIHEISKILVHPQYTDKPVNNDIAIYVTKTNIEWSRGVGPICLPPLANANTDFAYTSVDVIGWGTTSFAGPTSDTLQKITLMVTDNQACAKTYANEAPIYQTQLCTYDYTGSSKDSCQYDSGGPVVFKTTRQFLLAIISFGKICGEGGYATGVNTRVTSYLQWIYQNTGYSTCNKNFYS*

>Woma_00014777

MFVFLSTVICLILSFAFALFDQCNHKFYMKAGEKLYVNSPYYPNEYPVGTSCRYMIKAPPDYHLKFSCDIKLNTPYAATRCHNEVFSFNREGSELLTGSEYFCGSGKIERKSFLNQAAISYISTPSNKRILTSAIKEAEQNEQTQEKVTTETITTTTTTTKTITSTLKPLKENQLTSETNITKTTQIPIKTHIKKSPLIPISLSSTPVQTTSLLNTTSDQQHYKNKNVGNAFAYVVALLSDKIDLKVFTRKITTTTTTTESPLMSTTLRPTSVSVNKIDFLTSNTWRNGSIGQHKISLQRVAKVIVGQDYTTTSSPTSLPFFSSTSTSLFTPSTRLSEDGGGGGGSFSCLVEVLEPQCECGWSSTVKIAGASGVAGINEFPSMAGIMTIRNQKIFCGATIIHRRYLLSAAHCYTTPETSRPEFLRAVVGEHDLSTVLESLYTRHYDLVSIITHEQFHVSTAKVHNDIAILKTHLPIEWNRSVGPACLPFYSLVQGEGTIPYAGQRVETAGWGTTSFGGQQSAVLLKTTLDIITRTECQKIIRNLPFGAFCTYTPGRDTCQYDSGGALYVRGERMYAVGIVSYGFACATNQPSVNTRISSHLRWVRNKTPDAKYCWK*

>Woma_00014776

MLILFIKVYGIWILLLQFKLSQAQQCFSMDYQIPSERDILNITSPNYPQAYPPGSNCRHRITAPMDHVVIITCIFEVVPNSCDTESFVLSLDGDLQFRDAQRFCSSTQVTRISHYRSLALAYVSTRPNTMLRGRFYCQARARRVPCNCGWSSPTRIANGKEAMRNEFPSMVALRDITSPQRVFCGASIVSHRHILTAAHCTRSHSNPSAILAYVGDHDLLAENETPYAAQYRIQSIINHPGYRDTGNGIVNDIAILVTRIPIEWSRGVGPICLPWRQQNEPFAFFNVDVAGWGTLSFAGSKSNTLQKVQLLTMANRVCQQQYNDTINPAQICTYDYRGLGQDSCQYDSGGPVISRQPKLTLIGIISYGRSCGQRYGIGINTRITSHLGWLWRYIQNDVCVL*

>Woma_00014805

MLKFVILLAAVACALGAAVPEGMLPQLDGRIVGGVATTISNFPWQISLQRGGSHSCGGSVYSNNIIVTAAHCLQKVSTSVLRVRAGSSFWNSGGVLVGVAAFKNHEGYNARTMVNDIAVIRLSSPLTFSSTIKPITLATTAPANGAAASVSGWGATAFGSYSSPSQLRYIDTKIVSRNQCASSVYEYGSAIKSSMICAYTVNKDSCQGDSGGPLVSGGVLVGVVSWGQGCAFPNYPGVYADVAALRSWVVSKAQSV*

>Woma_00014803

MIKLLVLLNALHSNMAMVGSQMKPSMICAYAIGKDTCQNDSGGPLVSGGFLVGVVSWGVGCAVENYPGVYADAAVLRSWSIALATSAPANGADALVSGWGITSFGPTEVPAQLRCIGTKIVSRSSCASSSYYNADMISDNMICAYTVGKDSCQGDSGGPLVSGAVLVGVVSWGKGCAKPNYPGVYADVSTLRSWVLSITPSV*

>Woma_00014795

MLKFVILLSAVACALGAAVPEGMLPQLDGRIVGGVATTISNFPWQISLQRGGSHSCGGSVYSNNIIVTAAHCLQKVSTSVLRVRAGSSYWNSGGVLVGVAAFKNHEGYNPYTMVNDIAVLRLSSPLTFSSTIKPIALATTAPANGAAASVSGWGSTAFGSSFSPSQLRYIDTKIVSRNQCASSRYEYGSDIKYSMICAYSVNKDSCQGDSGGPLVSGGVLVGVVSWGEGCAFPDYPGVYADVAALRSWVVRNAQSV*

>Woma_00014834

MKFVIVLLAALALAAPALSRTLTRCSLAREMYALGVPKSELPQWTCIAEHESSYRTNVVGPTNYNGSNDYGIFQINNLYWCQPSNGRFSYNECHLSCNALLTDNISNSVTCARKIKSQQGWSAWSTWKYCSGPLPSINDCF*

>Woma_00014780

MFSKCSLLKAIICCLTIALSHSHNDLLNYRASPKLMAPLKMHHQHKNDITLNKLDDSLDQISTIYMQALFSGTHTPDLETRMRKLEIELFDLLDMLYKQERLSEFMRYEAEVTRQMILYNMLKKLFGYTTEEDNEKVK*

>Woma_00015083

MASRRNNCLPSAQYRRLTNEQRESYVQSLFDEICDDDDVIYVSETEDEQDIQLNEDEQDIQLNEVEQSDIEDFSEIQQCEEEDLAEYDDSDDEEEVAVTGSSEKFVARDEEDMEKERSQLHLVITIWSYAIWIEECPLYFLEAYEFRTYGIYK*

>Woma_00014856

MTMLEADSDLDEPTGADDKVTFDVPQVRSIDIQAREHEAVSEENIDDTEYIEDTSKDHQDIGTSSETEPEISLKYTHKSNISWRTVRVPENYRGITFEPILETDDVCAAGHRSHILVNI*

>Woma_00008426

MAKLWILTVLAVVALVQFASARDVAEALNVNIRQQLNLYGKFQKEADSAKYADNLKKLIEITEGALKTESADEKNQILTSTPSHFPEDFNKWIGAKLEEAQVNDDIQDSIEFYNSLLTANAAHEAEIKTTISTLENLLKEADLKQKEEKFLNLGQAFSPEFVKFLKDNALPGVNRQLQKTSEFFENLLHDKDIKFASEIEALKKKTDAALGSDVSIDDKNKVLQEVTFTDNEQLNEFLQKKNIEYS*

>Woma_00008427

MFFKYFMLIIFLLFMLVEKIASETITSEEQAFRIDNHLKFFIYRLQLIQAKQFTQLDDDRDKFAYELDLLKATFELALKNHIIREKVNLLNNFNKILCDYFSTFLMDELDMQYVNQKLCTDYKYLQLMQDDLKNLGLANETSSLEIKLEEILSLENLQEKILKYSEMLANLSPELCFILDKKPMHEIVMKAHLNFCKEFLDVLAENDIEEFRTDLNVLRRQFELAQGEEDQEDKLKFLNTFNDQSTKFGRFLN*

>Woma_00008482

MKFLVVFVALLACALAYETEILTEEQWQKLMEDESIEPETKGLILNGQATKVVKNALKQMPCGWPDLGIPPLAPYTNPDLEVHLAQSVVEALLQFIRFRFDGLNDMEIKKLKVSYTLNKKVNFHFNFKQLKASASLLNTDAFLDVMQQLGLSVRYEGSGPLEFALENLSIEGQFKYKLPLLIGSITIYKFQAVVTLGAVRSNIGGILGNGKMNRFVNDQIEDIIPAFINGHQAEISAMIEEKFVPRINEMMKGKKIWYLLDKMGSSSSKCEPTPAPWLANEVY*

>Woma_00008481

MKTVFAVLAIVAFVSAAEKPDPFASRSISSSVVEGIEAIRDQMPCGFPSKGIPPLAPLDIEHREINIDTPALRVTGVVDNFRLNGLNDFDVAEMKVNVITSKVTFRFIFNNVNVDTLYDLKVFLKKAGFTINLVGAGPAKFAIKDMQIWGTMKYSFSFLSGKIKLKSMDVRTRIGEVDSAIKGILGEGAVNHKLNEILPEAVIMAVNENEQLLSEIIENMALPKVNGILEKYSLSDFLGSIGGGVGEKVECIPPAKGM*

>Woma_00008444

MPCLDDVSVTTYAGYTAFLATHSSAIEASGLIQNQIDKLQIWLNYWNINVNKQKCTHVMFSLRRNDCPAIVMNGSQIPKSNHVKYLGM*

>Woma_00008469

MKSMLIFGLLALFVLANAEDVKKEVEAKSVESQPAASEDKSSVSTVEKKQEKRGIIHGVGYGYGHSGHGYLGGGHGGGAVLIGGGHGGGIGGGGLIVGGGHGGGIGGGGLIVGGGSAYGVTGGGGIAENIPTNVQVNTVVRNVAVPYQVERAVPYPVERTVTYPVKVPVPQPYPVEKIVNVPVKEIVKVPVHVPQPYPVEKTVHVQVPVHVDRPVPVKVPVPAPYPVEKIVQVPVKVPVPQPYPVEKVVQVPVKVPVHVPQPYPVEKVVQVPVKVPVDRPYPVHVVKPYPVPVEKPVPYTVEKKVAVPVQVPYDNPVPYPVDRPVAVHVKVPVPRPYPVVKEVPYPVERQVPYPVKVPVDRPYAVPVEHNVPVAVERQVPYKVPVAVPVHVQSQVGHGVVFGGGHGHGHGHGYLPKKK*

>Woma_00008428

MFIKFAIFFIFLQLNKLICCQPITPEEEEEHLNNRLKHYLKRIELLAQNPNSKFANELQNLMDKFKHSIAKESFKEKSLLLEQHTKLPDFVDYLALEFDKQYINEDILTESWYLNEILPHLPKDLYEEVTTILRELKEAYRAEDLEKKFNIYADVGESLSERFLDYFYGEDEPVELLNAQLKYYYEYLQYLLKECQVGGEFEKPLEELAVKVADGIANAEKTVKENIIDIFDDLTTKFGRYLDEHFVDFKMYKFGE*

>Woma_00008425

MDLKRILSIIFIISLMHGLTLTDKATVERIRKHQEWLKQRNQGRNSLLSTAKPKKSIELLNLWRFDKKENNSVNDVYPENDNILSSEAATSTTTKPHSTTTMTTSTTSQPTTIKTTFPPTAITTTTTITRKPTLPPTAATTTTTIAAALTTTTTLKPSVAPFTSTIPRKDSNFHFKATNTTSTTTNDWLSNDILETTTPFTITTTTTPAPTTTTTRKPKPRPYPRWGNWGSWSDCSRSCGGGIKHQLRKCINR*

>Woma_00008421

MTTSYKICLLLMVAVHSSGAIRPATTKDSQEKCGSVLCRKVSEIFSESKLNHGYHYIATIPMGAMNVTLKQMAKSNNLIALKTTDDIYIINGNNKASEGGIFLYDDDVYDYNKDTSTIVAKGPLHKPVKLMLFVRSLNPGISYTYTLPVASASISEPEELQSQWNELGLPLEESDENYIRGSASTQISRLRERKRRRFSWELLGFGPCNRSCGPGIQLPIFRCIRDSPTKESLSRYYSPKRCAFIEKPQFSEDIYHCNRGLCPAYWRPTEYGDCLCPEGEEEGVRSRKIECVQEQANGLIEVVDEAKCLSEKLPDLKETCNCQLIAKRKIYARNPDNPPKMYSKLIGSPPIIRSVMGKRHLNASLSTSLRRFHRDDRFDKAGVWLMSDWHPQCSNDCHVNYEYRTIYCDRTAPYTDLCDARLTPEQKRSCPTSSCRHGQWFTAAWSNCSVDASHCSLETKPLAVENCTQYDVEFCGPKWHYSEWSECSRTCGEGVQRRYAKCLEFDWKQNAMIESNKCKYLEREPVYGACSLAKCEELKMQQFNDNNNNNNNYMEHLQNDDNRSYDDTGNKAINGGVNSKTIRDLAAGKEKLVIAAAPKSLQNCNDELNNCKRINRERLCKLDFYKTYCCLTCHGYY*

>Woma_00008484

MRKFGFFIILTLLWTNLGTAKPVNSFNEVLLPESRTKFDDDLREVVEFLKMQMACGYPPLGVPPLSPYELEFKNFEYQDTIWWLKGNVSDFEISGLNAFDVVELHWNNILAKITFDFKFPFIEFNSLYKMNVYAPRKLFGNGIFRLQLFNLRLRGSLKLKPLSITKGLAVRDFKVKVDLESKRSKTTGIMYNRWSTKLFNAWLEEFISLTLEQEETVSRVVEQHAVPVLNKALKHISLVELVALITGLANDIVPNGPKCDF*

>Woma_00008483

MRFLLVLAFASVALASSIKIGHPMTEKIDAYVLDLVENNGELEFAVDHEDGTVEIAPQFIVSWQARRFIRKLQKEMPCGFPQYGIPPLAPLKIREGEISLKKDIFETFDKVLRFRFDGLDGFVIKKFKLNMITSKVTFDFLFPILQASADKYETDTLIDLLLKLGLSVQYEGEGSLEFGLKNLRIAGVLRYKIPILWGSIKITSLKTEISVGECSSNISGFMGEGSLNEFINRQIENVVVKGVNENSQEISDFIEDNLVPKVNKILKGNDFWTLIDLIFSSNEGKNEDDPIVTNCVPPTDPWA*

>Woma_00008524

MMKIPLQVLRQDIYNDIYDAMKSIKNITGGMNFSNDLTEEDINDWATEEIIQRAKRNESSVVLTESEDDFVIMDAKTKISNADAKKSFSVCVRSVGWGEYCLTIE*

>Woma_00008433

MAFNWKLCVVITMVASQLSHAFIILDTAECHYSDNELSPPNPKCSWIEKALKSLPKYRNFFASLLQDASGEFAYELQEIFNTICVAMDESDRRKQREQLMVAKWLMDTNKKLNEYLLKKGIWKI*

>Woma_00008432

MKFFGTCAGILCIFLIHAPRGVFLLENNDQTADPALVQFLNDEISKLLQANPEQDIRDFPLAGIFSEERSGLRNATLTNSDTVDAAAKAQDAIIRSRKFLHKYNMISSLVETAIKDLEQIVNDPKINSKIRDEARHLKERLRTSGSLEDAESALRSYDDVLRNLTIGQTPLLDDKYEQYLKEIIEILRKHYGNMNDPKLQALLKELINNPEQLKKLWKALSELSEEDDYDYSYDYFESLFSFFW*

>Woma_00008430

MKLFGTFVGILCIFLIHAPRESILQDPAPEPTPAPAPAPAPAPAPAPAPAPAPAPAPAPAPAPAPAPAPAPAPAPAPAPAPEPAPAPANMKVEPALVVFINQRINELLQANPVRDTTNFRLANLFNEERSRLQNAILSNSDTVNDAIKSQDAIVRFKKFLEKYNPLAYRIELAIKNLEQIVNDPKINYTIRDEARRLKERLRTSSNIDDAESALRSYEDFYKNLITNPMPSLDDKYEEYLKEIIAILKKHYGNMDDAEIQALLKELLNNPEQLKKLWQALSELSEEDDYDYDYDFFESLFSFFW*

>Woma_00008435

MLNNFFILILAIHASGIITGLSANSGAVSCGGLLCRPITGLFTRDPLPDDAYIHVASIPAGASNISITELKNSMNLLVLSTDEDSYIINGDNSMSESGAYEAAGAIFDYHRLDGLQEHGEGVTEWVTCTGPIRDNVELMIYSKTMNPGIKYEYLLPITSDSEENEVSLESDGFLKSGIEETSSSSSHTNRRRRYNWKVVGFSTCTKSCGGGTQTPIIRCVRENPLRYYSQRRCIHAEKPSINENLLRCNTQPCPAYWRLDDWSECRCNHGEGFKERELSCVQELASGIIIHVDDSACMEEKPVVKKACDCPKNRRRSNQRYRLHGLSSNASHANIRRNERAGTWLMSDWNQYCSADCGKGVEYRTIYCDRSKPNIERCDFRSTPDITRSCESSDACETGEWFSGPWTPCNGNCFNLTRTRQVLCIQNQLIVEDEDCRPELRPSNLEKCSHEEVEYCGPRWHYSEWSECTKTCNTGTQRRTVKCLEYDGHENALKESNKCRYSIREPIYRSCNTHKCEETRFERLQNDAASPTCSDGIANCQWAVKAKLCSYEYYQHKCCYSCSIAG*

>Woma_00008438

MITGLMAKVIAVLCITLCLFQNVELRPQKSMPNVGTNCGYASCPKTKPHMLNVHLIPHTHDDVGWLKTVDQYYYGSQTLIQKAGVQYILDSVIQELLKDPQKRFIYVESAFFFKWWREQTPELQEQVKMLVNEGRLEFIGGAWSMNDEAATHYQSVIDQFAWGLKRLNDTFGECGRPRIGWQIDPFGHSREMASMFAQMGFDGLFFGRLDYQDKDERLMTKKAEMIWRASANLNEQAHLFTGALYNQYQPPPGFCFDILCADEPIIDGKHSADNNVQRRVDEFFDFVKKQSEYYRTNHIIVTMGGDFTYQAANIDEFIAIARNMSQGYRTNHILVTMGEDFHYQNANMWYKNLDKLIKYANARQANGSDINLLYSTPSCYLKSLHDAGITWPTKDDDFFPYASDPHAYWTGYFTSRPTLKRYERVGNQFLQVCKQLTSLAPNLYPELIPHLGFMRETMGVMQHHDAVTGTEKQKVAFDYARRLSVGMRTCGANTRAVLNRLTQGDPPRPKRHAKPRPAQFEFKTCSLLNISSCEVSEKSDQFVLTLYNPLSHVTFDYVRLPVPDVNYVVKDYKNVVQEIQYVPVPDPVQNLTFRQSQAKYELVFLASDLPPFGYKSYYIKKTNGHHMKPVPEPDNISSLVDIGNEYIRLTFDTNGYLSAVEADGMTRIVSQEFLYYEGAIGNNLEFRNRSSGAYIFRPATDKPRTVTVAPEITIYRGNLVEEVHQKFNSWISQVVRVYKKKPYAEFEWLVGSIPIDDGVGKEIITRFNSDIKSEGIFYTDSNGREMIKRLKDHRDTWRVKLLEQTAGNYYPITTKIALEDEKARMAILTDRAQGGSSLSDGSLELMVHRRLLHDDAFGVDEALNETAYGQGLIARGTHYLIMGSSQLDHSPTTQSLERFTQLERTLAPWRFFSDTQFPYEQWRQQFTNIFTGLSIPLPKNIHLLTFEPWHENEILVRFEHILEKDEDPEFSKFVQFNVMDVFRGFKISNIRETTLDGNAWLDENRRMEFEPDPEQAWYENYAVYAKSANHVQLLKAQKPLVSTDYYEELLPIGNLGSESNRLKRSPHSEHQELNRMRMEKLKLLQLSVAELPLQYDDDRKFMVELSPMEIRTFVMYL*

>Woma_00008462

MKNLRNFVIQIILSVIFLCCCFQTIQTEYHTTKYTTTTTTKQPIIRTPGLSLPQRHTNPPKCTKEVPAIFFQFEKNIQVIGNSSLNPYFNIIETCCEGYKRYDFDWTKCVPDCGEKCQHNGFCLEGGVCQCFDDFVLNHRNDCVPTCPLGCPNGQCYLNGTCLCNKGYELDATKKYCIPVCTLSCGRNEICDAPDKCICAEGYARSQQPQNSQMGCQPLCIPDCGFGHCVGPNKCECFPGYQLKANTSVCESKCYLRCENGFCANRTGCICQNGYKYDVNTTTCLPDCGDDCENGICITPGNCRCFNGYQRNGAKCEAICEHGCGFYGKCIAPGVCGCAVIDGPFKSYQKCELGYCTSKGRCRCQIGTTRFINQCFAPDKVTTYASMKHQQLNEALIDEFNLLIGRHFMFGPYSSFKSQQEPQVLRFSYQKARCVPQSLTRSKTTVPQFWKMNALTEKDMFILDVKSTMSAGICYKQVPAKVTDPELRMRTLSFCCPGYKRSPLSPHSVKCDPICTEDCSNGICSAPDVCECYPGYERNGGRCEIVKKAATNTHVCCH*

>Woma_00008455

MYFNAVTIFNIIVILSTAVTLLAQTTCGDYECYEHTTCVIDQDIKMKCLCKENYTESRYGNKIYCCLGKGDLCATNRWCNEELECVSKTGEILKPEPADTESVEFLNSNCSSEQKCAETCCAENEICSSDQKCVSKATANCTDTQMCSQLNDFPKEKVSNNKEQQNNIDLVTVNFGNDTNTKKCSENKLCGEHCCNDSEICSKDNKCVSEEFSNCPPSKLCHQQCCAANEICNERSECVNEKIFVDVSQLYDLPKERVSKKTEQQNDLDAVIELFDFPDDTNTKKCSDNKLCGERCCNDSEICSNDNKCVSIGLSNCPPSKLCHQKCCAANEICNERSECVNEIVFVDVSQFYDFPKEKVSNKIEEQNDFSAVMGLFDFGDDTNTKKCSENQLCGEHCCNDSEICSNDNKCISEELNKCPPTKLCQQKCCAANEICNERSECVNKKLFVDVLELYDLPKEKVSNKIEEQNDFSAVMGLFDFGDNTNTKKCSENQLCGEHCCNDSEICSKDNKCVSEELNNCPPTKLCHQKCCAANEICNERSECVFENEKSDKNATSSVTPRGYIIIGKRRVIEPFMPYVEDPISCDLLNQLLTLDPKERIDVDRAFNHDFFWTHPMPSGLTKILAQH*

>Woma_00008456

MHLIIVTLLLIKLIRCTSALTITTGHCYRNVSVKYQVPITKTRMKLNTQLIEYYVEFEERLRIDSIRMCCPGYRTIIFGLCEPICSERCPINSYCGEPEKCLCARGYEESHNHHVRSLTSKQPKQLRCRPICSGGCPLHSQCVAHNQCACRPGYRDTSGWFNSLRCEHIQCPMDQVYDVLQHKCVKVDMNLEELMQRVGQKLTKGFNALDYGDEDLEEDNDTSRFSRED*

>Woma_00008468

MKLFICLATLLVASAYAETAAKKTAAVAAAASEKVPLEKKLDKRGLLDLGYGYGHSGLDVGYIGHGSVAHGHGYGIGGHGGGGAIITGHAAPQEFLISKSANVQKTITITKGIPVPHVVDRPYPVEHVKHVPVHVQVPVPQPYEVIKKVNVPVKEYVKVPVHVPQPYEVIKKVEVPVQVPVDRPYPVKVPVPQPYEVVKHVQVPVKVNVPQPYEVVKHVQVPVKVQVPVPQPYEVIKHVQVPVQVPVDRPYKVNVPKPYPVTVEKPYPVVVERKVPVQVPVRVDRPYPVPVDRPYTVKVPVDVPQPYTVEKRVPYTVDRPVPVPVSVPVDRPVPVPVTKHIPVTVDRPVAVPVAVPVVAGHSVVQHGAAVASGPAVVAYGGGGGHGGYVSSGHGFSSGGHGFSSSGYGFTSGHDATVVSDAHGYHKKK*

>Woma_00008477

MFSLQILKVLVFISVLQFTLASVIIRPPSPLINDETREEFERQVRLETEEFLNSIFKSQIGFFNKVKASLQPDSQRYKDIEAFVEKLEVAKQEQDLERKDEIYWNSFKKIHKSTLLLNDPDDTGLDNDEYQKILEDNGIKDLLKNFLADVAAYFWKMAKHSGKVVETTVDRLVDNMQKDKVVF*

>Woma_00008475

MKAIGLLTLLPCLRLFTVTLATDTCSANNPEARQFFTRQNELVRQRNRASYLAAYEYNTNVTEANRLHMIAVSAQNAIENKKLSEAIHKLHYDHLDDPCLQRQAKFLAEIGDDILDSQDYQALQNAISNMQTNYASTKERLSNSRDPQELAHYWREWHDKAGTPMKDHFAKYVELSEKAAKLNNYPSYADYWIHFYEDPDFEKHLDEVFKALLPLYREIHGYVRYRLTQHYGSDVVAPKGNIPIHLLGNMWAQNWDEVIELLKPYPNVSFVDVTGEMRKQNYNIKKMFELGDEFFKSLGMIALPESFWKLSVLEKPTDRTIVCHASAWDLFENSDVRIKMCTEVDTHYLYVVHHELGHIQYYLQYENQPTPFRGAPNPGFHEAVGDVIALSVSSIKHLNAIGLAPKAELDEQSRMNELFKMALKKIVFLPFAYTMDKYRYAVFRKQIPESNWNSAFWLMRSQFSGIEPPVARSDKDFDPTAKYHIDADVEYLRYFAAHIFQFQFHKAMCIKAGQYVKGDPHKTLDNCDIYKSKAAGDAFGRFLSAGASRHWKEVLQEFTGESDMNPAALLEYFEPLRVWLEKENRKLKVPLGWELTDKVPHTYKVPVLDLAL*

>Woma_00008474

MKLLIVTLLASLALCHAAVKEEISAAEYLKNLNKEIATRTNLETEASWAYGSNITDENEHIQKFNWRTYQSDDLRRQFKMLSKLGYAALPEAEYAELLDTLSSMESNFAKVRVCDYKDKKKCDLSLDPEIEEVITKSRDPEELKYYWREFYDKAGTPTRKAFDRYIELNTKAAKLNGFNSGAEMWLDEYEDDTFEQQLEDIFEDIKPLYHQIHGYVRYRLRQHYGDDVVSENGPIPMHLLGNMWAQQWSDIADIVSPFPNKPLVDVTNEMVKQGYTPLKMFQMGDDFFTSMGLKKLPQDFWDKSILEKPNDGRDLVCHASAWDFYLIDDVRIKQCTRVTQDQFFTVHHELGHIQYFLQYQHQPFVYRTGANPGFHEAVGDVLSLSVSTSKHLERVGLLKDYVRDEEARINQLFLTALDKIVFLPFAFTMDKYRWALFRGQVNQSEYNCAFWKLREEYSGIEPPVVRTEQDFDAPAKYHVSADVEYLRYLVSFIIQFQFYKSACIKAGQYEPNNPELPLDNCDIYGSLAAGEAFEKMLSMGASKPWPDALEAFNGERTMTGKAIAEYFEPLRVWLEAENIKNNVPIGWTKSDKCVSA*

>Woma_00008471

MQLMRTASLLNVVLLLLLLTHTSDVRGEEEKSAGKSETEEKKVEPKEKEAEKSADAKEEDSDKPKRGLFHSLGSYHHSFDDHKFHHHVHHYPIHKEKILTIIKKVPAPYPVEKIVHYPVEKEVAVPIEVKVPKPYPVIKHVPYEVKEIVKVKEEVPAPYPVEKKVPYPVHVKYDRPVPVKVYVPEPYPVEKKVHVPVKVHVPAPYPVEKKVPYPVKVHVKVDKPYPVEKIEHYPVKVHVDKPVPYHVHKPVPYHVEKPVPVPVIKKVPVPVHVPYDRPVPVHVEKPVPYEVKVKVPAPYPVIKEVPVKIEKHVPYPVKVPYEKPVPVVLEKHVPEYHEKHVSYKEPEFHYKKHDDHHFDEKPIVEHAEGDEYKHQEHEHEYKHAAQEDYKFEGHEGFQHEVKHEDFHGHEQLGHELGHIEHGHIEHGHEEQGYVEQGHEEQHGHIEQGHEEQHGHIEQGHEEQHEHIEFGHVEHGHEQHGYEQHVYEQHGHEQQGHEQHGYEQQGHEEHIHHEYQHNHHEFQHEEPKVHHYQNHNYYHGHY*

>Woma_00008470

MKLMRTAYLLAVVLLVATTYACAEEEKQAAGAEEKKVEPKEKEAEKSADATDETTKSKRGLHHFEDYHHEQHYPVHEEKTLTIIKNVAVPYPVEKTVHVPVEKHVPVPVKVKVPKPYPVVKHVPYEVKEYVKVPHEVPAPYPVEKKIPYPVHVPYDRPVPVKVYVPAPYPVEKKVHVPVKVHVPAPYPVEKKVHVPVKVHVPVDKPYPVEKVVHYPVKVPVDKPVPYHVDKPVPYHVEKPVPVPVIKKVPVPVHVPYDRPVPVHVEKPVPYEVKVPVPAPYPVVKEIPVKVEKHVPYPVKVPVEKPVAVHIEKHVPEYHEKHISYKEPEFHYKKLEHHEPAHHEEYHHHGGHEEIHEHH*

>Woma_00008479

MKFIVLLVALMPLVWAAEFQEIAPAKQRSISSQAVDAIVAAKEQMPCGFPSLGIPPLAPFKTEHQEININNQDLTAFGEANNVVVYGLNDFDIKKFKVNIITSRINFEFHWNKIFATADYEVFSEFGNKGLRRAGLAKIALKDIMVRGAIKYNFRLIGKKLSLKEAKIYVTLGEVQSEIEGLSKINIINKKLNRMVEKWIMLAINDNTDKIANMADSMIVPFVNKMLSDKNLSELLGGGGNGKPSEKVPCVPPEDAADYMKYFM*

>Woma_00008478

MKFIVLLVALMPLVWAAEFQEIAPAKQRSISSQAVDAIVAAKEQMPCGFPDLGIPPLAPFKTEHQEININNQDLTAFGEANNVVVYGLNDFDIKKFKVNVITSRINFEFHWNKIFATADYEVFSEMGNKGLKGAGLAKIAFKDLMVRGAIKYNFRLIGKKLSLKEAKIYVTLGEVNSEIEGLSKMNIINEKLNRVVEEWIMSAINDNTDKIANMADSMIVPFVNKMLSDKNLSELLGGGGNGKPSEKVPCVPPEDAADYMKYFM*

>Woma_00008461

MLEKSLVLKIVLILQYLLSHQFCKAQFDKHDIDEDISDTYILQRPPYKMDPPYRNPYQPIRKHKLHKKPQSFASTTESLCVRNLKKKKAEEEAENPTKKVVPSGNRWHGPVQYSEYLWRSKKSAGRRTKIRDITPVERKLHKCYKWVSSQELKHYEWPAIIQTIEDRNLFHIEICCPNYGPIRYMGNTLCRPFCSNCRNGDCVAPEKCRCFDGFVLTDNGECVFTCPISCLNGRCNLLLGNNCLCNSGYKLDATGQFCRPICHTGCGVNPLHNCTAPDVCGCMKGYSLTDNDCQPIAKQINP*

>Woma_00008463

MKSFIFLYLALLAAAVCQGQYKNLGIKTRYPPPSSGNLILGNATQSHNETYNYVYSQQQQHSEVPLSQPNPYEPYNGNFKPAEYYPGGAPVVVNRPQPVILDATAEFINKTRAGMASGVCYKEVPTASLVHGSGAVPVGNGTKPEMSKIQVCCEGYERNPHVFKRCDPICEDDCPNGICTAPNTCVCMPGHVRNEEGKCITTCPIGCGNGVCSDNNECKCKPGYKLEPTQQKYCIPHCETDCQFGHCVAPNRCECQKGYRATATGVCEPVCENCENGKCTAPGYCSCHEGYIKVGALCEPVCARGCDKGTCIAPDTCSCSQGFELDRSGTKCVPHCDAVCVNGFCGGNNQCVCNAGYIASEDQPYLCKPHCPLGCPNGYCSGPNFCICKPGFIKTGIKGRQSCTPA*

>Woma_00008431

MKLFGTFLGIICIFLIQASRGVFLQVPASAETSAHAEDQVPPQPGDQVPAQPKAPLEAQEPVQLGAQAPVQPGAQAPAQSGAQVSAPPEAQASAQPGAQALASPGAQVLSQPDAQASDQPGAQVSAYPGPQAPVQAGVQTPTRAEVQSQAQLRAQASAQPRAQASAQSGAQTPAQPRAQASTQPEAEVPVQPEAQASAEPVAQAPVQVEVKTPAQAEIQPQAQLGDQAPAPPGAQAPTQPGSQVPAQPGTQAPAQSGAQAPVPRAPQVSVQPGTQAPATPGAQKPAQHRLQAPATITIDPLLTEDHNRNINYPKTQDLLKELLNNPEQLMKLWKALSSLSEEDDYDYDFVESLFSILSKENWWELKR*

>Woma_00006616

MLSMKTFVVISIALVGLACVCAQIAPGSNQYLPPTKNGYDYSEPKKPFKPQGPAPGPGPRPGPRLPPGEPGPDHVHMPGNPFDFEYAVNDIDTNNDYAHKASSDGDVVTGEYRVQLPDGRMQVVRYTADWKTGYHADVSYEGEPRFPQGPGGRPGYKY*

>Woma_00006524

MEYSLKNFVISENLKDSSKTFYSSNVIGRDILSSYDLVMDHFKNCHSHSVHRHNHSTVTNDVNTTVIYENQEESDIYFNYNSNTAISNHFCEYKSESSTYQSDRQKQRRMRTTFSSAQIKELEKIFQETHYPDIYTREEIATKIDLTEARVQVWFQNRRAKFRKQERHSLHISKSTPSY*

>Woma_00006480

MCNTPLHQRNINNSVEPFSFSTFHFLRTRNRLMIPGAVADSTSCANLMKNLQIQPQLDAVLSQNYETERSSQASPILDVIENSTTEHLPNELDHTTSSLAQDGSMLNKPQFAFKPIELDFVTKTPSTDNVVSLPNLRVLPLPGISSNNLSHQTTLPAFEYLTPAVRNLSPIDFPLMELNRVGGMFPSFLHRRPRGEKRPIPEEQKDDKYYERRKRNNEAAKKSRDARKIREDRIAFRAAFLEQENSLLRAQVIALRDELQTMSQIIGRSNLS*

>Woma_00006558

MYRSAFVLLAVVVTCTQGSVLMRRIPELDGRIVGGEDTTIENFPYQVSVRLFGAHTCGGTIINSRTIVTAAHCIYNFLGASSYSVQYGSTVVGGSNNVIPAIRVVKNEKYDSSAINNDVALIFLSSDIPLGASAEPIQLASVAPAPGTPAVVSGWGTLQEGGASSSILQKVNVKIVNKNVCEEQYRGVNSITDKMLCAGTVGGGKDACQGDSGGPLVANNQLVGIVSWGVGCARPEYSGVYTDVAAVKDWIDANLA*

>Woma_00006556

MLNILAIFIVLTLLAGVSAAPEARIIGGRETDIHKYSYLTSIRYRSQADDPYIHKCAGAIYSERVVITCAQCVVDIKEHEKVIVVAGANSRAGVDGLPYPALKWVSHPSYSSWTVDYDIGIIIINDVFDFQHLKVKPVSIKEVRPADGRLATVAGWGYREEYGPSSTNLEEVQVPIVSTADCLKAYGAGEITGRMICAGYLKSGGKDACQGDTGGPLVVDNQLVGLVSWGRGCARPGYPSVYTYVKELQKWIDDTISATNIYYIKNKLNTYVLKHMQIKES*

>Woma_00006554

MGRVIIWLVFGYALASSLVVAIPPRLGGRIVGGYEADIKDITFQVSLQGPNHFCGGSLIAKSYVLTAAHCTDGKSSTNPNFNVRLGSKYSNKAGIIVKPLKIYQHEKYNPLTIDYDFSVIKLEDYDDTALPFKIKYAKLPRKDSLKDGTVLTVSGWGSTQNSAESRDVIRAVKVPKVNNSDCAIAYKDFGVITDQMLCAGQQEGGKDACQGDSGGPLFKDNIIWGVVSWGYGCARPKYPGVYARVTSVLQWIKSTINE*

>Woma_00006555

MIKILKIFFLIITFIIIFVPFSQSLETRIIGGETTEINQTPYLVSIRYKKYNNTAFEHRCSGTIYSAKVILTTATCLIGLEINRIHIKAGSSYRSQNDGLLYLAKKYVLHPDYNIWFTDNDLALVILAFDLPTNFPKQIAPITLASEVPAEGSLATIAGWGLTDSSSNNNFSEILQIANVHLVNDTVCKRSYGENRISRAMLCAVGTHTDACLGDAGGAIVYKGTALGLISWGNGCANTDFPGVYTNLVHFREWIQNEVGKI*

>Woma_00006552

MFALTLIIVTVESVSQGRIVGGCESDAIYGFERINDGMLCVGKDSGGKDACQFDSDGPLVSYNKLVGIVSEHPGVYANVAYFENWIDSVAQK*

>Woma_00006553

MKLLVLAALISLSQGFLLKHLKEPEKRIVGGNEVHIGKHGYQISLRKKSIFSPQNAYTHICGGSLYSDNIVLTAAHCIIATVPSQLKVVAGSNFKTGSDGVIVPVKEIIMHENYNPDTYNNDVAILVLAIPLPLNKFTIRPIELINSAPLAGAPTTVIGWGALSEGGPSPARLQEVQVPVVDHDDCNEDYEGFITEYMLCAGLRHQSGKDTCQGDSGGPLTIRNKLAGIVSWGNGCARPNFPGVYANIWTLRPWILEKIAKMSINN*

>Woma_00006473

MCKLLAIVLIAMILGYSASLPQIEHGSVRGPSGRFERTRNQAAPPVDLSLPPVFLPESIPIHEDVKEFYADGGSYGVHS*

>Woma_00006472

MNKILICLIGLVVLSTGQRITTIHLDGVQYFISRMNPYSPELNYFLAYQYCRSLGLQLASFETKEKAESMTTYLKNAGYGNYDFWTSGNRLGTGMFLWMSTGLPFNATFDFFENTADAIQAGLLDPVDHNSNTSPQRTARDSSSGAEKGCVILKQPTLKWMPEDCSAVKDFICEQTRCYYYNYGSIPVSSAQGRPITTTTARSNIADVNKSEIKITTPLPFLMSSYTTDKKTNQPSYVSLKLNEERALTTSSKNDDNHLNDQENEEEHEDDDESNNSQGDEIQGRDLTNEEFDHDIDGSQQENSEELHEDQDNIQPNGSNKSNDGQQSDENSKINSLHSQEESEESTVKEKSLESYEIENTPELPAVEVRLKEISQEVEKRKNEENRQIPSLQPDLDDIGRQSFLSLTDLIRTLHPNDKQIIPQIDSDYSNAMRVLGEKSVVVKTSEE*

>Woma_00006669

MFKFVVVLAFVACVSALGPYDGHSVSHGSAEDAHAEIKSLDSEVNEHGFQYSFDTTNHISASAKGDEHGNHYGDFEWISPEGEHVAVKYVADENGYQPSSDVLPTPHPIPVAILKSLDYIRAHPVHEEHYGHH*

>Woma_00006665

MFKFVVVFALLATAAAQRHGHHGQAHGFARPSSPVRATSDDVHAEIISAKSDVRADGFDYQVETTNAIRAQASGDAYGNVHGDFEWVSPEGEHIAVNYVADENGYQPNSAVLPTPPPIPEAILKAIHYIETHPQYEENQYKKQQGRRF*

>Woma_00006666

MFKFVVVFALLATAAAQRHGPAHGFAHGPSHATTDDVHAEVIAARSDVRADGFDYQLETTNSIKAQANGDAYGNVQGNFEWVSPEGEHIALSYVANENGYQPNSAVLPVPPPIPEAILKALRYIEAHPHKEESHNHGRRF*

>Woma_00006667

MFKFVAVLAFVACASALGPYGGSSEDSHAEIKSLDSEVNEHGFHYSFDTTNHISASAKGDEHGNHYGDFEWISPEGVHVAVKYVADESGYHPISDALPTPPPIPVAILKSLEYIRAHPAHEEQHHGYY*

>Woma_00006660

MHWPAVAYFFTFLTVALSCQTLPLQQESEWWENASLYQIYPRSFQDSDGDGVGDLKGITSRLEYLKEIGITATWLSPIFESPMADFGYDISNFTRIDPTFGSLEDFDEMLAKAKQLGVKILLDFVPNHTSDECIWFEKSVKRDPGYEDFYMWHDGKIDPVDSSKRLPPSNWNSIFGGPMWTWHEERQQFYMHQFLAKQPDLNYSNPLVRQHMLEVLEYWLQRGVDGFRIDAVPHIFEKRNEDGSFPDEDISGVSSDPTSYDYYTHKYTKDQYPTIELLYEWRAFLDDFRNKHGGDSRILLTESYSPIDFLSLAFGNATYKGAQIPMNFNLMDLVSTSTAKDVEILCHNWMDTMWSQHKIANWVVGNHDSSRVANRIGKNKVDLLNIIVHSLPGTSITYYGEELGMSNVDIPCTPTATTTCEARNPERTPMQWDDSLNAGFSQAHTTWLPLAADYEFYNVKTEREIGRSHLQIFKGLQDLKKTAAFKNTKGEGGFSYKALTEQIFQIVRALPGKEEYMILVNMGDELEYLESLNDKMYEYILMNTYSPHNKGDKADFRGRVYLMPYEAVVLKWLA*

>Woma_00006661

MFKLLSLLMVGLALQAQARDWWENGNFYQVYPRSFKDSNGDGVGDLNGVAKKVQYLKDLGMDGVWLSPIMKSPMADFGYDISDYYQVQGEYGSVEDLLALIEKAHAVGLKLILDFVPNHTSDEHEWFIKSVNNDPEYRDFYIWHDGVINPVTGKREPPSNWLSQFKFSAWEWNDKRQQYYLHQFGVKQPDLNYRNPAVVEAMKQVLVYWLDRGIDGFRIDAVPFLFESEIDPQTGTYPDEPLDESNACPDPTDWCHLLHPYTNSLPEDIEMVYQWREVIENWKREHNSETKILLTEAYTSFENLMLMYGDGVRNGSHVPFNFEMLAGLTKISKAEDYKYFIKHWLENMPEGTFANWVLGNHDNRRLASRLGTERKDLINILLQTLPGIAVTYNGEEYGAEDVEVSWEDSVDPQACNADPLTYQKYTRDPARTPFRWDATKNAGFSTADKTWLPVGEGYEQENVASQLQEDNSHLKIFKRVTQLRKEEPAFSGRQIEPVVQGEVLIYERTAPENSEADSFLIVLNLGGLDAKVDLSTYAHKLGSQVEVTVSSKQSGYKQGELVDSDSFVAKAYVGYVLRLK*

>Woma_00006662

MELLGKFFILFSLVATGFARDWWENGNFYQIYPRSFKDSNGDGIGDLNGVTEKLQYLKDIGFTAAWLSPIFKSPMADFGYDIADFYQVHPEYGTMEDFENMIKKAKEVGIKIILDFVPNHSSDENEWFIKSVNNDPEYRDFYIWHNGKLNATTGEREPPSNWLSAFRYSAWEWNDKRQQYYLHQFAIKQPDLNYRNPKVVETMKDVMRFWLGKGVAGFRIDAVPFLFEKDLDENNMYPDEPVVENVATCPDPDDYCHLQHIYTNDQPETFDMAYQWRALADEFKKEHGGDTRILLTEAYTSFENIIRFYGDGVRNGSHVPFNFDFLSNIFNDTSAADIVSHITKWLNAMPKDVIANWVLGNHDNKRLASRLGINRADLFNILLQTLPGNAVTYNGEELAMHDVFISWEDSVDPQACNGNPDTYYGLSRDPARTPYQWDASNLAGFTTGDHTWLPVADDYQTNNALAQLRAPQSHLQIFKKLLKLRKEPSMQDGELQIKAIADDIIIYSRQKEGSDLYVIVLNLSKNDQTININQHYNMGSDAEIITTSIQSKHRDGQIVDPSNFLAEAEVGTVLVKV*

>Woma_00006663

MFLFLIITVCILFILYMELKAYYIRQKIKHTSGPKILPLIGNAHQMGKTPSEILNKLFTWWYQFGQDNYQLWIGYYLNIVISNVKDVEYILSSNALIQKSDIYDMLHPWLGQGLLTSFGAKWHKHRKMITPSFHFKILQEFHEVMNLNCNKFVEKLKQVSKGETIFDFQEETHYLTLDVICDTAMGVHISAMDNHDSEIVNAFKEMCYNINMRAFHPLKRNNSLYKFFPDYKDYCKALKILQDFTYEVIEKRIETRKLEESSKTETPNDEFIKRKMAFLDTLLSSTVDGRHLNTQELYEEVSTFIFEGHDTTTSAITFAVYLLSRHLDVQQKVYEEQKKVMAGNMKRDATFQELADMKYLDLVLKEAQRLYPSVPAISRMADKEYNINGKIIPKNSALNIFIMALGYNDKTFPDPYRFLPERFELSNRADKNPFEYVPFSAGPRNCIGQKFAQLEIKTVVSKIVRNFEILPALDELASKDGYVSTYFGSHKQNKLPNKYDPILSAVLTLKSENGVFLRLKERY*

>Woma_00006606

MFKNLICFVLIIKLTQARYGYMPKFYGQDSTIIVDANKPPPINNNVAVNYRNKLRLPAPFPLWRHYPRYHIIPSPPLPAQVFSVNTWAKAANKKPLTITTVEPTTTSSTSTSTSTTTITTPFSENSFGRNRAVTTTPTTTTTTTTPKPNIFYITSAPFVTDDEFPKELLNIAQNKLGLKSLDEIPSISELGQLLGTNTPKETLNYIKQLTSNEQGVAFMKAYIESADYTDNSGDLDNRNEDEEDEDVMGDMEEATKINDLDLVVISDEDLNNDYDLETTTLMPRKLPKIMQKLKKIPQKQQSFLEHVTNFMHLNNLVDKKSAEQQEIYENYDNELTRMTEMGKNVIKKLKPADKDVLENQTEAEMLEEPEKNMNQNFKTPILVRESALPYNYPVPIRIASPTTNTALPSTSLNSPNGNSTSPVPVHLNTPKILIPHVQQLSRVTKIPTKKIEDFLASKPKLIEFATKVSRFPIGYDRNSPVEAQVIAAVQKAIEQDEDLKKLLSATATLK*

>Woma_00006621

MKFLILLFALIAAVAARPGGLYGGGLYGGGLYGGFGHGISYAAPSYSYAAPAISYAAPAVSYKAVAAPAISYAAPAVSYAAPAVSYAAAAPVVKAVAAPATSYATFSTVVSHAPAVKAVAVAAPAPAISYAAPAISYAAPAVKVAAAPAISYAAPAISYAAPAVKVAAAPAFSYAAAPISLGYGYGHGYGYGHH*

>Woma_00006622

MKFLILFALVASVAARPGGLYGGLGGFGGFGGGISYGAPAVSYAAPAVSFAAPAFKAVAAAPAISYAAPSISYAAPSISYAAAPAISYAAAAPAISVAAAPAVAAAPAVVAAAPAPAVVKAVAPAASSYTQVSTIVNHPATIRYALAAPAVKVAAAPAVTVAAAPAVTVAAAPAISYAAPAVAAAPSISYAAPAVKVAAAPAISYAAAAPSISYAAAPAISYAAAPSISYAAPAVSAVKVAAAPAISYAAAAPAISLGYGSGFGYGGGFGGFGHH*

>Woma_00006626

MKCFTTIAVLACLSFVTAEPPAPQNSYLPPNQQQPNSNYLPPSNTYQAPSNNYLPPNRGGGASAPQNSYGAPAVTSAIFPKQGGGAGGYGGGNQNGYGAEENYGPAKYEFKYDVQDYESGNDFGHMESRDGDLAVGRYYVLLPDGRKQIVDYEADQNGYRPTIRYEQVNGGQGAGRGGYNNGGGYDSNAQQQGKFGGYQ*

>Woma_00006627

MLKLFLLSTLTVVSCLAEAPYAPAGFRPTIPFDLPNEYYPNDDVYDPNQGYSFEISKQRVDFAGQEIQRVPAVVSEPQHQYLPPNQFGFNNNRDTTNNQRPQAHPFFNHRHNHGRRNNGRPTQRPSAQTPQNRPPQFQPNFPPTNIPLPRPTTDERFETQQFFPKEQQQQPALEYGAPNYNIDPRNNRQQQQQQQQQNQYNFNYQNSISQQQQQEPALEYGAPNNNYETSNQQQQQQQQQQQQQQQPALEYGAPANTQSLKEEELEKQFALEALNAAVENYNRLQENENDRIAQGQYFVVNPDHSIQKVKFTTKKSKDESRPTDFTAELNYTKVGEINDPLYKYTAEGQLVRIVKK*

>Woma_00006498

MMKLFYLLSVLIYIASAIGMPQKIALTPLSSEEEFGTMQRGSSLQNFGSIRDRYPIYILGPYHSEDKQKPNLNTIRAYLNFINADSVILRKLLLSKLIAQCANQTKSLTNHICNHVFDNDYDHIYFTVSPELQLSNDSSKIKKNFDEIDKTSASFSTLNQLI*

>Woma_00006547

MLSSDRQIRAGLTQGSVLSPTLYSIYISDVTVPRNHETAFYADDRAYICRGKVSNEIVKRMEYSSRHQQILRLVDKAYSYETYLL*

>Woma_00006657

MWQYCIIIAALVSAAYAGVYPNYHAHFDFVAWDKEKTLEDYAKATKWWQTASFYQIYPRSFKDSNGDGVGDLNGITEKLPYLKELNVTAAWLSPIFKSPMADFGYDIANYYEIDPIFGNMKDFEALIKKAHELDVKIILDFVPNHTSDECEWFIKSAAKDPFYKDFYIWHPGKMVNGKRQPPTNWVSVFRGSAWAWHEGRQEYYLHQFVKKQPDLNYRNPKVTAAMKNVLKFWLGKGVAGFRVDAVPFLFEKAPNANGDLVDEPRNPSVTDPDDYGYLQHIYTVNQPETIDMVYQWRQVLDEFKHEFGGEDRIMLTEAYAPLEVVMQYYGNDTMEGSQIPFNFLLISNLNNNSNAYNYADVINMWLNQMPEGRTANWVLGNHDQNRVGSRLGTDRIDMLNMLLLTLPGCSVNYMGEEIGMTDVWISWNDTVDPSACNSNPDIYEKLSRDPERTPFQWNRDKQSGFSTANKTWLPVANNYWDVNVEVEKRQPRSHLDIFRNFQEFIRTQPAIKYGQTDVRAVNQNVLGVKRYLLHEFTYIFLMNIFDNVETVDLQKAFLEIPNEMVYELVTAKSKAKKGDLVKASSVVLQPKDAVVLKTMAKLTTTAGYFTYYKAEDKNTK*

>Woma_00006656

MFRLKILIFYILIITGLFKRSYSCIGNGGIQQTIASKDWWQTSQFYQIYPRSFMDSNGDGIGDLKGIISKLQYLKDIGVNATWLSPIYVSPMADFGYDIANFFDIQPEYGTLEDFDELIKEANRLGLKIIMDFVPNHSSDENIWFKKSVNREKGYEDYYVWHDGYVSENGTRLPPSNWLQAFRGSAWEWNEQRQQYYLHQFAVKQPDLNYRNPDVVAQMKRVLTYWLDRGVAGFRIDAVPWMFEVLPDENGRYPDEPLSGYTDDPEDSGYLLHIYTQDQPETIDMVYQWHQLLEDYRRIHGGDTRVLMIETWSSIDQVMLMYGNRTTEGAQIPFNFQFISGGNSNQINTDLKAPGFSKIIKSWLDNMPAGKTANWVMGNHDKRRVGSRYGEKRIDLMNMLQMFLPGVSVTYMGEELGMTDLDISWEDSVDPAACNSNPSIYEQFTRDPARTPFQWSSSTNAGFSTAQKTWLPVNPNHDTINVETESSQTKSHLNVYKQMSQLRQTKTLQDGKIQYGNKGDNILAIKRSLPNEKTYILLANVLDEGTSADVSDIIKEAGTFKTKISNVISTRKQGDLVTLDNVYLEGNEAIIVESL*

>Woma_00006653

MDEELRRILEESDFDFNDSSSSVGEEYDVTDFDSDDYEADSDFLPDDAQLISATNSDYDDDSNIAINAVDQAPTTHRNLFGDPCPISCANSQESDMVQKNCLRCYFWIKIP*

>Woma_00006652

MRNNYNKVLQIILATWLIQQLSTSTAWQPAIAMEATVMPALMNITNIVLKQKTLMKTADSLEKPSKSELNQSAIPKGTTNSMYTTKSTSSNKFDVNNTDTQMTLKLKDATARNVYTAIPAVILAVAADNNNSKTAVSISYGSQRRATENIYTETHKTGQQLTSNTVIRSQETERNNQTERKSSTPHSAPGNLTALTLKSTDAGDKMGRDFEMLQQTQPTVHDDKTENTGATDTMATKTIVNNLIRNNILINKTVSTNGKVEKNVERKEIVKLKDINEGLISRTLKDSSRKHYAEWEAVTAQNAKTSNVNGIAVVLLKTNNSIKNKEEVKEQDKQGKQETHFNTTNYIKKQTENLLVTHHRLESKEDREEMKEKIKSFNFRTKNNGDVIILSQKQTSFSSKPALKAPTSDIKKSLKNLKVKAANITAFHTAHRTIADNKSFQKAESLNHTKAYFNESLSQDKSLLMIDTKAFVGPGFLDNKILKLSDIKSLNKTQSSSSKQTNKNSNETKAKRNNNNESFSESKVSQENSFSQTSNMALNTSHSKLKPLLVTDKANDYLVKHKATESLKVLPLQDLHNKGNANRNLRHYLRNITIPAKALIEKKIKVLFEKEMELEKSFEKLKQNETKIYKNFEMNMKEIHLELQSLKEGHNVKQKTQLNRTKLQNFTKQKLLSLSDTNINKKFNKSKEKFDTGSFLKKNNTKNYTSLWQRQKRLNIKSSQSDGLAKQTAIDQLPPSPKNYTQITKSMPKINTKTRHDKQSITDSTEKIQNITMNESVEMETADVHKLKYFGDEQKQTQFNRNETTDAGGNTTTAINDVMNNKQLKSEVGGDSGDSDKQQYVDNETQKKQKEQIIRGKYNDAGIKQDIGTTTTQTETSLSITPTVADKAKQDIWELLTTLTLEADHVLTSTTADAGIDLITPTKQIFIDNMANISSTHIVDNSSLSINGLVNATLNPPTLTASLTTTADLMQLVAAATAAAASATTALPTLLTLSPSLNATSTLTTSASFYSTFTATTSSTTTTTPSWPVKHASVVEGNVILGGLMMVHSREDSITCGPIMPQGGIQALEAMLYTLDQVNKLQLLPNITLGAHILDDCDKDTYGLEMAVDFIKGKW*

>Woma_00006659

MEWRAVVFVLFFLASGLYNTEAFAKSEWWESASLYQIYPRSFQDSDGDGVGDLKGITARLEYLKEIGVTATWLSPVFESPMSDFGYDISNFTKIDPIFGTLADFDEMIAKAKRLGVKILLDFVPNHTSDQCEWFRKSIKREQGYEDFYVWHDGKIDPENSTKRLPPSNWVSVFGGSQWSWHEERQQFFLHQFQDKQPDLNYSNPLVRQHMLEVLEYWLDRGVDGFRIDAVPHIFEKRNEDGSYPDEPVSGWSSDPNSYEYHDHIYTKDQYATIELLYEWRAFLEKYQKRKGGDIRVLLAEAYSSVENLNLYFGNGTHFGTQLPMNFNLMYLNGYSTAKDVENSCNYWMNTIWKTHQTANWVIGNHDNVRVANRMGQHKVDLINIIVHSLPGVAITYYGEELGMSNAVTACTEISCDDRDPERSPMQWDATENAGFSNGSSTWLPLAQDYQTFNVETERKIARSTLNIYKELQNLKHTAPFKNFKSPGGFSYKAVTEQVFQIIRAIPDKEEYMVLVNMGNKLEYVENLSKNTYVYKILSPHSTQQKGDKINLSGRIYLKPYEAIVLHWLKR*

>Woma_00006658

MRSKWTLLATIVLISCVAIKIEARTKAWWETASFYQIYPRSFKDSDGDGVGDLKGVTSKLAYLKEIGMAATWLSPFLKSPMADFGYDISNFTEVDPLFGTMDDFEELMKKSKELGVKIILDFVPNHSSDECEWFIKSANRDPEYKDYYVWHPGRIVNGKRTVPNNWVSVFRGSAWEWHEVRQEYYLHQFHKKQPDFNFRNPKVREAMTDILRFWLRKGIDGFRVDAIYHAFEIAPDSESNYPDEPRNDWTNDPDDYGYLHHIYTVDQPETPHLVYEWRRVLDEFRKENGGDERILMVETWSPIDIVMHYYGNSTAEGAQIPFNFQMISYLWNDSDAYHYEKLINEWLDLMPAGRTPNWVVGNHDKNRVGSRFGADRIDIFNILLLTLPGCSVTYNGEEIGMTDVWISWQETVDPQACNGVEEGYEYRSRDPARTPFQWNDEMNAGFSTGNTTWLPVSPRYKTENLKRQRGISLSHLNISKKLQKLRAENTLRDGGAEIKAYNRDVLVVKRFLPLDYTFVSLLNIFDSVQKVNLYDIFVNIPTEFEYVLVTDRSIRRVGDKVTSDSITLWPKEAVVLRSTKKA*

>Woma_00006463

MKITIFGILLIFVAIIFCCHPMHCKPLDDVYNDDDGYNGNLDKKTIEQLRQCDMDLETMELCMRCAKITKSTIVYPLCCANEDKIKDWCHDYVFFGLL*

>Woma_00006673

MFLIASLIAYAAAVSNSDDVHAEATLLKSDVRADGFDSALDTTNHIHQVASGDVYGNIHGEFEWVSPEGAHVAVKYVADENGYQPSSDLLPTPPPIPEAILKAIAYIQAHPPKTEHYH*

>Woma_00006671

MLKFFIIASLIAFAAAVGNSDDVHAEATLLKSDVRADGFDSALETTNHIHQVASGDVHGNIHGDFEWVSPEGEHIAVKYVADENGYQPSSDILPTPPPIPEAILKAIAYIQAHPPKEEYHH*

>Woma_00006670

MFKFILIAAFVAYASAAAVSSDDVHAEVTALKSDVRADGFDSVVETSNHIHQVASGDEHGNIHGNFEWVSPEGEHVQVSYVADEHGYQPSSDLLPTPHPIPEAILKSIAYNAAHPQKEEVHH*

>Woma_00006676

MFKSFILFALLACVYAVSNEKQENVEVKQLESDVRADGFLYRLETSNGISMGAEGDEHGNIHGHYEYISPEGEHIHVDYVADENGYQPSSDILPTPPPIPVEILKALEYLKTHPSVEDKKY*

>Woma_00006675

MFKFILITTLIALAAADGSKSDDVHAEATLLKSDVRADGFDSALDTTNHIHQVASGDEHGNIHGDFEWISPEGEHVKVTYVADEHGYQPSSDLLPTPPPIPEAILKAIDYIKSHPSKEEVHH*

>Woma_00006674

MFKFFLIASLIAYAAAVGNSDDVHAEATLLKSDVRPDGFDSALETTNHIHQVASGDVHGNIHGDFEWVSPEGQHIAVKYVADENGYQPSSDLLPTPPPIPEAILKAIAYIQAHPPKEEYHH*

>Woma_00006430

MSLTWLCLENTSKIVLFLIALTVFSASATKTDSQTDSETHINSLQDELEYEEFMDADSQQPHSRRKRLVWITDDGRLALPPGTSLTFTPTIALPLVRHPPEGFFSNMSISFPVTIDFDKLGLTDNQNPLGDLPPIFSRTFGHTAGEMLGNYMSKYLHFKSKRDLSAQQQQQQQQQINPNFNINVQKHVSGEQQPEFPQLPERFKHIFHGGERVILYGVVEDFLSTFGMNGKACLLRTICEVHSRKIDHYGVFGEMAKLFLTVTRSPFSDLIPEYVKAQEIGEGRTAPGECFPYHKECPRSLFKPLQNHKYRETQKGEYHEQEVEEITKPQLPENPSAAEILDQQTVDEIENLQDNEIRMNVKKIKFNDLYSM*

>Woma_00006425

MKNVQQWMFIASACLVLAIVVNNNVDAAAPFKKVSIATTSKTTTTTTTSTEAAAPAEGESVAEAKEEKPEHHHNSTATTKDSKLTGIPQIDYIWDPNLPRELRGYNLSSYPFLSTTPADDEIHFKCDGLHDGFYASIEYKCQLYHHCVYGIRHDFLCANFTAFDQRTFICHFVSDVDCEGSMKYWNRNDDLYMATTTTSTTTTTTEPPPTQRRRPVRPLRPLRRPANRRPIDDYYYEEEEYDEDYYEEPVNRRRKQRPRHRKPVADYDEEYEDEKPRRHNGRDRVRDEIEPEDDYDIDRRPLEKPKSRGKPTAAAERRKLSSRKPSTFTGRSSADERRSFSDDRPLTGRRKAALPAVADDSAEPVETPRRSTGAGASHIPDQEEAAADEDAPPKVKTTPKPLEEFVTPKAAPSSVYARPRAPPRIARPVPVNAKGKFQYPVQRTATTSAPPAGRAEDDDYYADEEYDDVRPRRGHSKKRIVDDAHEEEDEVPAPKRGILGRRPTKTLRKPLTEKKSVPVDEEEDYDADDRYTRRGTSRRRNPLTGRTRSRGAPALHDDVDFVDDDYDERPAKRSRPNSRKTSKKPIIPEPSEELETEEIEPEVEQKPLTKGRGSSLNSRRKSVPAIPSRSRRPAAQAAALVHNDEYDAEPAEEIDEVPSAASKSSLPISRPASVRVVKRPFLPSRGGSPYLPRGLQPVGVAHSKSHDAHTTDSSMIDMFSTNSGVHLLEHGAPLLRDSGPRTTLPPRSAIPVSQPRPEPQPSSKATLDELYENDYDVTLNDALNPTLKPLSPHQSNHYHQHHHENNNDNNHIRRRTVISPPAPQARRSNYHRNNSGSGTRIAPNYYDDDYEY*

>Woma_00006437

MKSNKNCIGGCRHHFNKTRFLLLSIIWTLLFTETMARPNLETLLSNKPNELEQQQQQQQQQQQQQQQNIFLDEIITNTNGDKLFNKLRKYNRNQATNYLNKRPGLLDLVLAFDYDDNNGQEDSNEFWNTKFFDRLRQFASLKTDSYDSDDIMRDNSFDSYNDRLPMKSLKSSSSSSSANQLGFKDHNIKKNVQCK*

>Woma_00006439

MLVLRTFIKACLALLITVNFVTVSQAYAIGQSKYGRIEKFPYQVMLIGKQLWRKRILCGGTLLNSRWVLTAGHCTMGVTHFDVYLGTKSVNESLESGKVILRSNKFIVHEGFNPVTAAHDIALVKLPEDVIFTTRIKPAILPYHFKNDQFIGSRVVATGWGAVGETSKSDPMQYTELKVISNAECSNEFDVITAGVLCAKGLKDETVCSGDSGGPLVIKGTPIVVGITSFGPANGCETNIPGGFTRVTHYLDWIDAKIGNSQQTQSLQFVQKQQQHDQHPNQNIV*

>Woma_00006350

MWKLIPIFLCIFYINNGQATYNTGIKTRIYTTREQPITQVVQHHYQSTPTHGNYHQQHNSNCARCDNDYQTSITQQQQYVSSYQQQPPQQNEGGYYQPTQNQAHFQQQPLHRYYHQQQQQQQQQQQQQLQQPQPQPLPIYNRGEVNSQAQTTGNWQNSKNSINEFYTGCPTGYTGQLPYAYDCRRFLNCWHGRGHIQTCSVGTVFNAETLECDRPDKARCEVALGVLGQTNNNQLPTQGGFKQQQQTQPNYRAGRYTDVSTDAVEVLCSPDAKGLQPHPSDCTRFINCANGNMHIQQCGPGTAFSVAMKVCDFIDKVDCSGRDGGGGGRAFVAPTQDKNEVVCPADATGLHPHPYDCTKFLQCSNGVTYIQDCGPGTGFDRVRSLCDYKEKVKCCSGCTWGYNTTETTIIPSNNDLHTQESSTVNHEQHEQLPHTNDILCPKGVTGLFAYPFDYTKFLHCKNGNTAIQNCVPGTAFSISKGYCEPLDDIQQSDHVIYIVSQVSYEYSQTLITCPPGTEGLHLYPFNAEKFVKCVQSQMEIVPCSVSQVFSITRRHCLPTDQVLKHERVRLLSELQNLNDYSVTDEHYTLQKKVYCPGGLTGTYPNPFDSTKYLKCVQGRLYSETCASGQFYSLSRKVCSPKEEIDVTDRVPLAKLTQGLEWNINDMEITYSSIDGLTYLMCPPGLNGYFLHPFDCTKYIECRSGATVVDECAQGTVFSISRQECIPRDKVEAYDRVEYMITTTNEFSNESANHQNQELGITCSPGTFGIYPHPQDCHKYMRCSNGRLSMENCPAGQVFSVTKSFCHNENEVPTNDRSSYNRGHTLTQSHYAGPVTTNTNTVHQTGLYNPINIPPTSLMPPPRPGVISPPTAVLTPPLPSKPTANVINEIACPEGSTGRYPHPHDCTKFLMCTNGETHVQDCGPGTAWSRAMEVCDFIDNVKECSTNSLSSSSSTTIVNSAQSPYVCPAGIEGLYQHPYDWHKYLVCNHGQTTIMDCAPGTVFSISRRICDLEANVLNNDRCNNGAYSSNYDYSYGQNTNNRHHSSTQWSYAGTQNQPTTHTRVTESRWINMNVPRPQSPTINWQQSSHTHHVHSPSTRPDVTPVWSGTAAHVDQRSPTHTIIYEEGSFEPNRNYKTQINNTPLAPLKPPTTSTSTTSTVVYAQPIGSQEDSKEIFSNAPPKTTNIYYPPPPQPTSVNRQNHTRHINPWTPISRPIDIPRHNLMPSQPAQGSVNTNTGVIVPVVTHFKPPSPLPQPPRNHERPLVFRQPIGEEEVLSGQTPLAEGDFKITTINIDEIPGQRTYTFDDLDQLPAASSKHNVNDNSNLYGGLQPPLSNKPTTAKNFPPPYEQTTQIPEPAKTTTPTTHIPGYPQLSNATIQTFPHQPHYSPPYAEVTHSRNATWVGRKLPPLSSIKPIHGDHSTNDNKNIRISSPKPPPHYYGLNLPPPPPANKQHEEKDNVDEDEEQAMKQALKLLLKPYLDPEAKVDDVVAKKAQSHIMSLVTTPTTTTTASSTHKTSSNRNTLAPITSTTTTHQPDVELILAGEQHSLVSVSTSKTGNHETSTAYNLYQTPLVSERSSALPNQLDHDHNPNWHKHHNPYHTHSRHHSKLQHSREYHEKHPNLPNPFADTYNNPKTESTTPHSYDIDLRAGGPCPFECGNGKCIDQHQVCDGVNNCGNRKDEQQCDHLGYELRLTGGESSHMGRVEVKILGKWGYVCDDKFGLRDADVVCHELGFKMGAMEVRGNSYYPPTDVNLHFVMDEVECKGNETSLKECDFKGWGVNNCGPDEVVGVVCKVPKLKCPDNYWLCSTSKECIPTAFLCDVTPDCEDGSDESEQVCNAPIKYRLEGGRSSNEGRLEVFYRGEWGTVCDDDFGLKEAQVVCNSFGFYGKPEIIKNIYGPGSGPIWLDQVSCFGNETKIDECNHWSWGENNCNHTEDVGLKCTAGPKPAKKLNNPSTALAPAKDSSFSLEFEQTELDDLGLYQGLWQRSSKAINNQKKCGHFKTNLIDEYEHPEERVVNGSIAKRGRHPWQATIRTRGRGGISSHWCGAVIISKKLILTAAHCLAGYPKGSYFVRMGDHYANIAEASEIDVNIENWYIHEKFRDQKHMNNDIALILLKSPIRFNDYIQPICLPEKGAILETNRMCTISGWGSIKSGTSTPSNILRSAQVPILADEVCSQKNVYNNAMTEGMFCAGFLDESADACDGDSGGPLVCSDKDGETLYGIISWGQHCGYANHPGVYVRVEKYIDWIYDKINLMLQQKKL*

>Woma_00006371

MKIFVCILALALASAQAGLLPHHANEVVVAPHHIDHGFHHDDVHHHADVHHDGGIHFGHSAQIVIDGGDHHAVEHHPVYHHDDHHATVEVHHDDHHAAPAAQIIHHEPIHKVEPVHHVTKVVHHEPHHFLHYTNPVVDHHVVHQNHHVDEHQHAADLHHTELHHGDAHHHGDLHADLHHHGAVDVRHIAAGIVHHGDLHHHHAAGLIHHDAGFGRHYHTDVHHHAALVTHTVPAVHHHHHNAAIVHKVYPAHHPTHADILAIAKHHLHGKYGKVKITEKHY*

>Woma_00006331

MLRLLLVFWIFTSLDYAYANQCFAKHQKEINNDNFNILIRNITDIIYSGLGRLPEAIQLKNNGKTIVWKYKYDTPEPVLIEGGPYGKNVYQFDFIYFNWLNESDINEKFLPIELHGVFYNRKYVSFEEALQKNFGITVWSFRYTSEMRNLTEDMADKIYNALQYVREPGSKTTILPMERLLVPLNVIFNLPSEYFVYQGSLLPFFNNCKSHVIWIESPNTVSIQQAQYDNFRQLLSFNNQPLMSQVLEYATPNSTVYQNIDKYKYQWNNLPATNFEYFDASFGEEINEEESTSVAVSNKRIRLFLLKIVVIFIINGNSK*

>Woma_00006318

MRSAIILLTLCVIGIYAESSEDLLVRKVREFEGYSYPPPKEPFTYPPPVRTTPPAPVTTPKPTTRVPATYLPPTNKPQTPATTRAPVTTPKPTTRVPATYLPPTNKPQTPATTRATPRPTTRAPVTTPKPTTRVPATYLPPTNKPQTPATTRATPRPTTRAPVTTPKPTTRVPATYLPPTNKPQTPATTRATPRPTTRAPLTTPKPTTRVPATYLPPTNKPQTPATTRATPRPTTRAPLTTPKPTTRVPATYLPPTNKPQTPATTRATPKPTTRAPVTTPRSIVTTQRPTQAQTRKPSTVGPTYLPPTNKPQTPATTRATPKPTTRAPVTTRATTRATPKPTTRAPFTTPKPTFVPSTYLPPTNKPQTPATTRATPKPTTRPPVTTRATPRTTKPTFVPSTYLPPTNKPQTPATTRATPKPTTRAPVTTRATTRATPKPTTRPPLTTRATPRTTKPTFVPSTYLPPTNKPQTPATTRATPKPTTRAPITTPRPTTRAPVTTPRPTTRAPVTTPRPTTRSTPGPTYLPPLNTPETPATTRATPKPTTRAPITTRATPRTTKPTVVPSTYLPPTNKPQTPATTRATQRPTTRATPKPTTRVPVTTRATPRTTKPTFVPSTYLPPTNKPQTPATTRATQRPTTRATPKPTTRPPVTTRATPRTTKPTFVPSTYLPPTNKPQTPATTRATPKPTTRAPITTRATTRATPKPTTRAPVTTPRPIVTTQRPTQAQTRKPSTVGPTYLPPTNKPQTPATTRATPKPTTRAPVITTRATPKPTTRPTFVPSTYLPPTNKPQTPATTRATPRPTTRPPITTRATPKPTTRTPVTTRPTLRTTKPTFVPSTYLPPTNKPQTPATTRATPRPTTRAPVTTRATTRATPRTTQPTFVPSTYLPPTNKPQTPATTRATPKPTTRAPVTTRATPKLVTTPRVPQPKPTEKFEGYTYPKPAIPFSF*

>Woma_00006381

MKLFLIVLSAAALANASTFIPTARLRRDVSHLNLNYLPPPTNNYLPPLPLTLPTGTQPSNEYLPPVVIEEQATPEIEISNEAPVAEVQAAEETTELPQPSNENLQPTNLGGEDISEIPAEPLAEAVAEPVEPTVPAEPTATAEIVEALTESKQDTAVFKEDGYHYKTPSETVNLIPTH*

>Woma_00006366

MLTFNLIAQVFPICCTFIASVVCWGECDLNADLDNMRSKLLHYNNEQYHEIFNLMSDMKAQSQECSKLIVPRMLVTESDIQQITTELENLTTQIENLQTTAQADEQKMIDNQENWKRQLEMIINIFQSEAQQIIPDQQNWKIELEVLNYITKSETQKIIAEQENYASQLIDKILEKINDMNAKLQSSIEMISESFSKN*

>Woma_00006363

MAHSVDISMISPPTSSSISTDQDPLGQLPPLLPPLRSTQVLKPLTGFPVSNLSEDSYDYVFGGRRKTPPSTTALKLTSPPVRLRPEDAYNNTRNISGGGFGSNTTNNGSTGGGRFYRHSFSYVPKRSRHTQNERERNANYESRLRCHGEDEATLRQLLLEYVLKTFVF*

>Woma_00006368

MKTSVCIAKAFLIFYVLQNSFGCLGIDLYDLFDYSSEDDSYEDNSNEERNYRGDNIMWKKDNNDDGCNLKQTVNDIIDKFLDLDRTHLYEYSARADSIEYWFNQCKKNVDAVPYVTKDLVNRIMDNINVCKQKIQEIQKLKNNNQNTDWNMKIENLQDVIQSEANQIIANQGKWKVEIHNLQSLAQSELQKLFADQEKSANKVIDKILNKLENVSDQLNKLMKKLKESLNKNPKQINKLLEKLKENLK*

>Woma_00006367

MKSFNINTQVFLISCTLIASVTCWEEAEECNPIKELDKIQNELRNNKLILNYFFDSSLEPLTFNSDACITGITSKKGAEQHEIDQILPNFNSLKNEIQKLQNIPQEAGQQNNQQYFENELQKLEIIAKSEIQQIIANNQYWKEELEKLENITRNGVKETIVNQQNLEIKIITTIMKKVNDINNQLRDGIEMIIESLNKM*

>Woma_00006734

MATLAEATFATTQCPIPSVGVMNSLVTLLVQSILTAHCNISRTEYWPQDYAQETLQKGLDAYDFIVVGAGTAGSLVASRLSENPNWNVLVLETGGDPPQESEVPNLFFTIQNTDFTYPYLVEPKLQSL*

>Woma_00006723

MKYLVVIFAVILSAIFQNLQTVDLFVLKGFTTGNAFETRLDYVDSLLQQLFDTVLGKKEELQDPQLWPKDYAEDFMKGGPMTFDYIVVGAGTAGSVVASRLSEDPKVQVLVLEAGTDPSILSEIHTLSLQLLNTSYTWNDYAEPNPACCQAMKDGRCYWPRGRMIGGTGGVNGNIFLTGYPSDFDEWQQQGNEGWSWNDVYPFYEKATHEHSQNIDKPVGSLTLNYFERLDSYHILTELMRNATAEVERSATVKSPGYLDNILGTMDRGKRMSTGKTYLGRVAPTRSNLYVIKNGVATKVLINNGHKATGVEFLVNGTHLLRARARREVILSAGTFNSPKLLMLSGIGERNHLKSLNIPVVKNLPVGDNLQDHGMMPLVLKFNKNIPTTEDEEPLPLSLYDYLIYQKGPLASSPTLIGLINTLKEQHENKSDIMLVTHFSKPTKESNVFEFLQFKPDLVDKFLDRLENQTILEIQGLIIKPKSRGSVKLKSADPLQGPLIHSRYASDPADRQTLLRFIRFIQKLAKTSTFQHYGLELITVPLEECDKFPHDSNNYWFCYIKYFYISAWHGAATCRMGPASEPSAIVDQRLRVHGIKGLRVIDASIMPNITSGNTNGPTIMIAEKGAQIIKDDWLNNI*

>Woma_00006774

MAKLFVTLAILCVFGTVLVKAFDKNEAMASFLAKMDECKAESGAKDGDVEELMNKKLPTTMEGKCLLLCLMQKYEVMDDQGKFVKDTALSHAEKFTDGDEERMKIAREIIDTCVATDVDSDHCVAAEQYEKCFQEQIVAHGIKDHFEF*

>Woma_00006828

MKSVIFFALLAVVVACALACDRDGNNQPDCNTKNLNVPVRNFWDPTHYWLCKQAGVAAESVRCPDAQGFDSAKGACVPFSEWQWTDPCPSADN*

>Woma_00006826

MKSIVCLAIFAVALTCVLACDRDSNNQPDCNARNLNVPIRNFWDPTHYWLCKSAGVAAESVRCPDAHGFDSAKGSCVHFSEWAWTDPCPENSAN*

>Woma_00006827

MKSAIFFTILVVAVALVLACDRDSNNQPDCNTKNLNVPIRNFWDPTHYWLCKQAGVAAESVRCPDAQGFDSAKGACVPFSEWQWTEPCPAAAN*

>Woma_00006820

MFFKYFKFFILNLFFLHYGRTSETCDLSEFYQYFYNQIESVKKSMEILNTRLDIVANGYTLGSHNEKIIEKRDENAKQTTPPIFDIRYDSFPKHCDADVKASTCAEATACTRSSGIYNITVTRYNNKPFQAYCDNDNFEGDWLYILRRLDGSVNFSLPWMDYVEGFGDVGGEYWIGLENLYALTNFNGPQELLVFLENFEGRQVFARYDNFVIGHANERYKLKSLGRYSGTAGDSLHFQLGAMFSTYDSDSDTLANRNCAIERYGGFWFKDCTKANPTGKYLRGTYTKLYEGSYWRTFVGENYSQKTIIFMIRRQRPMLVDANKENLKK*

>Woma_00006821

MLPNKVLFIILFVIICDFNKAWGRGHGHGRGHSSHGGGRRGSSSSSSSSSSSSSSSSGSGSSSGLGSSHSHHTGNSASRSSTRRKSYSSSSSDEQIVSDRYEPITYKPPSYDSINFSPPSYSLNEYKPPTYEYRTPARYVPPQRISVNELDDFISGYITGNVLGSSGTHFNNFYSQPYQPPIVILPGYESRHYEESKPKEEKVITTWRADNSPSSFPTANNFESLNLNIPLPGISNSLRVHCKQQMNSDLNERGEIVDSVKQICGPSTPVTTTPYPITSTSTTTTTTAKTLETSSLTSKPYAMKKKRYERIMASTTRGINCRKVMNSEFNELGEIVDSVFEVCDPPHTTTTTTRKPTMKTTTRRPTTTPKPLMKVCRPVMDSYLNENGEIIDSVTEVCTMVPVK*

>Woma_00006746

MFRILCLFGLLLQVTDCQQNIQHMLNLGLTNLIKQSLYNDAPRQSAEYDFVIVGGGPAGCVLANRLSENPNWQVYLIEAGGLESLVHQVPAMAAFLQGTHSNWGYKSVPQKASCWGMHNNQCSLPRGKVLGGTSSINFMIYNRGNKRDFDRWSEYGNEGWSYREVLPYFLKSENAQLQGLEDSPYHNHSGPLNVEDVRYRTKFVHAYVKAAQEAGHVRTDYNGESQMGVSYVQANTLNGRRQSAFRAYIEPIRQQRKNLHIVTLARVTKVLIDPNTNRAYGVRVLYRNTLYTIKARKEVILSAGAFNSPQLLMLSGIGPRDNLQAIGVPILKELPVGKLLYDHMCHFGPTFVTNTTRQTIFTSRLTPNVLKSYATGDPSTVLSSIGGVEALTFAKVPGSQEPDDMPDVEFINVAGSLASDEGTGLAQGANFKPEIYTKVYKTLTETQQDHFSFLIMQFHPKSVGRLWLHNRNPLEWPRLNPKYFHNSEDVEVLLQGIKEAIRITQMPAMQQVGTRLHNIPIPGCEGHLFGSDDYWRCSIRVMSYTLHHQVATCRMGPANDSTAVVNAQLQVHGIKRLRVVDTSIIPFPPTCHTNAASLMIGEKAADIIRSMWSS*

>Woma_00006745

MLKYFRKWPLTGFIAVMCSLMAVSWAQERNVILETIDFLRRGQTDVNLENYDNTVNIESEYDFIVVGAGTAGCALAARLSENPKWKVLLLEAGGPESLIMDVPIVAHFLQLGEMNWKYRTQPSDHACLAMNNNRCNWPRGKVMGGSSVLNYMMYTRGNRRDYDRWAELGNEGWSFKDVLPYFKKYEGSSVPDADADFVGRDGPVKVSYVNWRSKIADAFVESAQQDGLKYRDYNGRIQNGVAYLHTTTRNSTRWSSNRSYLYPIKGKRPNLHVKKNALVTKVLINPETKTAYGIIVQSNGESQKIKARLEVVVSAGAINTPQLLMLSGVGPAKHLKEMGIKPLVDLAVGYNLQDHTAPAVTFTTNATSLHFEDFANPTWLNRFNRQEGPYGSPGGCEAMAFWDLDHPHLEDGWPDIELFLVGGSMSSNPAISRAFGLKKSIYDSIFAEIEDKDLNAFMIFPMILRPKSRGRIMLKSTDPTKYPLIYANYFNHPYDVDISVRGLLKAISLMDKPGFQKINAKLWERKIPTYRAAVVDPRLRVYGIKNLRVADASIMPEIMSGHPNGPVFMIAEKAADMIKQDHGFIP*

>Woma_00006744

MKYLTVIIQLVLFYFRYNAAQQTNVVSFVLDFLRRGQNDLNLENKDNEVKFLKEYDFIVVGAGTAGCTLAARLSENPKWRVLLLEAGGPESLAMDIPAMAHFLQLSPEINWRYRAQTSDKYCLGITNNRCNFPRGKVMGGSSVLNYMMYTRANRRDYDYWAKLGNEGWSYKDVLPYFKKFEGSTVPDADRDFVGRKGPVKISYTDWKSPIADAFVEALQQDGLKRRDYNGRIQQGVSYLQSTTYKAIRWSSNRAYLYPLKGKRPNLHIRKYAFVTKILIDPQTKTAYGVIFESQGKSYQVLARREVILSAGAINTPQLLMLSGIGPAKHLREMGIKPLQDSAVGYNLQDHFAPFVTFLTNATSLHLTDFFDVRNILKFGSNTGQFGSPGGVEAIAFYDLDHPGLEDGWPDIELFLISGGLNTNPATIPALGLKPDIYQALYSDIIRKDGNAFVIFPMILQPRSRGRISLRSSDPHKYPIIFPNYMADPYDVDIVVRGVIKSIELMDRPAFKKINARLSHNTIPACRKYGSIKTRAYWECYVRHLTFTIYHYSGTAKMGPKTDRSAVVDPRLRVYGIRNLRVADASIMPKIIAGHPNGPVFMIAEKAADMIKQDHGFIQ*

>Woma_00006808

MKIIFNLVLVTLLSALLCCSAQESSKQLPAEYVESVQRSTHTNNWAVLVDASRFWFNYRHVANVLSIYRSVKRLGIPDSQIILMIADDMACNPRNPRPGQVYNNANQHINVYGDDVEVDYRGYEVTVENFVRLLTGRTQNGTARSKKLLTDAGSNVLIYLTGHGGDGFLKFQDSEEITSQELADAVEQMWEKKRYNELFLMVDTCQAASLYEKFTSPNILAVASSLVGEDSLSHHVDSSIGVYMIDRYTYYALEFLEKVQPNSDKTMGEFLTVCPKRLCISTVGVRKDLYRRDPYKVPITDFFGSIRPTEIITNRVNITMAEEEDFIAAALEAEENHAKIDSFYIQFESQFPKDIFK*

>Woma_00006749

MNFNFFILLIDFAFITKAAYAFQNIMSSFAQLGITDLAHQIFSPQIPRNNDVFDFIVVGGGTAGSVVANRLSENPNWRIALIEAGGTENIFNLVPALCGYMQVVHGVWDYRTVPQKRACFGMYNNECRHPRGKILGGTSSINFMIYNRGNRRDYDSWAEAGNYGWSYDEILPYFRKSEAAQLDGLEFTPYHNTSGPLSVEYLKYRTEIVHAFIKGAQEAGHRYTDYNGESQLGVSYVQANTRGGRRHSGYRAFIEPIMNRRKNLKVFTLARVTKVLIDPSSKAAYGVEFLYRKQRYVFKARKEVILSAGSFNSPQLLMLSGIGPEDNLSRLGINIIQELPVGLRMYEHASHFGPTFIVNTTNQALFASRFGLNDVLGFLAGRPDTRMSSLTGVEALTFLKVPGSDRPKDWPDCEIIFASGSLASDEGTALKIGANIKDEIYNRVYGPLQRSQQDHYTVLIMPFHPKSVGRVWLKNKNPLQWPVIDANYFDNPEDVEVMLEGIKEAIRISQTPAMQRIGTRILDTPLPGCEAYTFGSDDYWRCSIRTMTYTLHHQVATCRMGPASDPTAVVSPELKVHGIRKLRVVDGGIIPFPPTAHTNAPTFMIGEKAADMIRGDWGG*

>Woma_00006725

MLLTRLNDSIIVLILLLSISSGWSATLSSITQSEITQTKQLKKHLEVLKDHLEDAIQKLDHNIKARQTQYQRDILTPDSEYIEAALASQGNSDFIRAAAVPAIPLPSQKMRINQNSGLRRKRYT*

>Woma_00006858

MKSMLLFCLVFYCINDAVIGKKVLNYDYEKSFGNLDIVHYQPEQVHLSFGENSHDVVVTWSTRSDTGESIVEYSDGNNFANQVVGIREKFIDGGDKHKKQFIHRVSLKNLKPSTKYIYHCGSKFGWSAKFEFRTVPSGVEWSPSVALFGDMGNENAQSLARLQQDTQSNMYDAIIHVGDFAYDMNTDNARVGDEFMRQIESVAAYVPYMVVPGNHEEKYNFSNYRARFSMPGGTENMFYSFNLGPVHFIGISTEMYYFLNYGLKTLVFQYTWLERDLTEATRPENRAIRPWIIVYGHRPMYCSNENDNDCTHSETLTRVGWPFVHMFGLEELFYRFGVDVEIWAHEHSYERLWPIYDYKVLNGSYKEPYRNPKAPIHLVTGSAGCKEGREPFLKNIPEWSAFHSQDYGYTRLRAYNATHLYFEQVSDDKNGDIIDSFWIIKDQHGAYC*

>Woma_00006829

MKLVIFFTLLAVACILACDPDGNNKPVCDSSNLNQPIRNFWDPVHYWLCKAAGAEPESVRCPDFQGFDSAIGQCVPFDQWQWTPPCPSVAAA*

>Woma_00006752

MKFLIVIAFVTLAIYNVRAELTKEEAIAIATACKEEAGASDADFEAMVKHQPAETTEGKCMRACALKKFGVMSDDGKMIKEASVELAKSLIKDDEKKELVVGIIEACEGLEVSSDHCEAAEEYGHCIKKEFESKGVTSAEDLIS*

>Woma_00006753

MKLFLIIFIAILAKQSLQQSIEGLREIAKASVVDCYEDEENTKKIEITEAGFEDIANGSRDALRNAKCIRYCIMRKHNLFSADNSLNESEIIPFFAYLFNDTIDRNHLKEIIAACNLKISRETDKCERSHMANTCILNKLNESGMKDI*

>Woma_00006755

MRGVLICSLLLISCLLCSAQYGRGGGGGGGGGGPIGGGGGGGGPLGGGGGGPYGGGGGPYGGNGGGPYGGGGGGPFGGGGGGGPYGGGGGGPLGGGGGGNRPYGR*

>Woma_00006756

MKLSVFFGILVLLMCSLICSAQRGGGYSSGGGGGGGGGPGGGFGFGGVGPNGPYGGAGGNGGYGYSSSDGQRGSGSSGNGYGYSY*

>Woma_00006757

MAFIKLIIFFALFIVSTNAQIGEVINNINTAVDANADFLTTLVSAIVKLPFALLTPLMPRKPPPQNDQIQQILAALLALQNEKAETNNMNKKIVKKDKSPKDNSKHNPEDNKENGEHDQI*

>Woma_00006758

MKYFVVLTIIVCALLALARASPLPGGGGGGGGGGGGRGCNCEPGGGGGGGGGGGAGHGSGPGGSGGPGGPDCVRPSWLPPCP*

>Woma_00006759

MQSFIKYILALYVVLLSEGEIHWMVGASESFSNSGDYEGSGGHSFNFVTPLYRFSSGDGELSSNSDNEGYNFKENYDNSGDYSGFHCGNIFFDTDFDNNFDKNFVYSGDGNNDKSV*

>Woma_00006760

MKRNMKFEIACVLIFFVVVFCEVNAHRDSSIADGLRPEHKHDGFSQPLYYGELSDGVNNNRWVFRRPGQKRLPGYGGNDGIADKPGKPGNGYPAQLERPVYGNNGQLKQFSYGGNGQSGQTGYGGINQPTNVYGGRPGQNWQSMFTGDAVYGFPLQSWHSGYVGSGQLEQPNKPAQTSQTGRPDNGQQGHHSFDYLNYHPGQLGRPGLGNNGKLKHGGKPGQNGQSVYAGSEVYGLPGQYWHTGYFGNDRPGRPNKPAQSSHPGQHANCHQGQHSYGYLNFHPGQLGRPVYGNNGQPGQTDYGGNNQPNNVYGGQLGQNWQFVFTGDKVYSLPGHNWHAGYFGNGQPGWPYKPAQSSQAGQHENDQQGQLNDGINGQTGQSGNSGNGPSGQQVNGGNGQPGRPGKPEHPGQPGQPEKPGNNAGNDGNDQPEQSEEPGYNFDNDENGQPEQGEEPDNNGDNDGNAQPEQAEEPVNNGGNEGNGQPEQSEEPVNNGGNQGNGQPQQAEEPGKNGDNDGNDQPELAEEPGKNGDNDGNDQLEQLEEPVNNGGNEGNGQPEQAEEPGKNGNNDGNDQLQQLEEPVNNGGNEGNGQPEQAEEPGKNGNNDGNDQPEQGEEPDNNGDNDGNDQAEQSGEPGNNVGNGQTGQFGNGQFDQTANADNGQPGRPGKPGQPGQPGQSIIDNDGNDQPEQPEESGNIDGNGAHVTNGNNQPGQPGQSDGNVVGGDYNYNAYEQYAVPVYEVYYEYEY*

>Woma_00006764

MKVSITIIVLISSTLLNGAQSKKVKNYYGNAISDAKGFQKPLASPLGNSINDIFKRFKRDTTKSMQELQKMLDMAKSECINELKMDPQIMEKSVMHAENPTTNEKCLMFCILKKTNLMENNQLSLKTIGRIASIVSQENPLVISLVVAKANNCNTMIQPNKPACEVAYEINRCISDELKARKLNFNY*

>Woma_00006766

MILKQKIAFILGAILCLNEIMEVFGGATEEQMFAAGGLMRDVCLPKFPKVTKEIADGIRQGNLPNEKDAKCYVNCILEMMQTMKKGKFLYEGSLKQVDILMPDHFKDEYRAGLAKCKDAANGVKNHCDAAYAIFTCLRSEITKFVFP*

>Woma_00006809

MKFVVIVCVLFALIAVNEAAIAMARFNNPTYPGKCTIDENTILAPGQKGKAPNNPCAGVTCMDNGYVEFRTCPAVAPPKGCKLRDFVNVNRNYPECCERTYDCTKMI*

>Woma_00008672

MVVRYSYCILVTILVGLALARDHDHPKPRSTEPHPVKYEAERKTDYWINNAQDILAEKLRQTETVTTNKAKNIIMFLGDGMSVHTVTATRNFMGDSSKQVYFEKFPYFGLSKTYCVDRQVADSACTATAYLGGVKGNYGTIGVNSNVKRYNCFDGQNPANWVDSIAKWAQDAGKDTGLVTTARVTHASPAGVYAHIAQRDWENDAKIMAANCSPKDNVDIARQLVEWDVGRKLKVIMGGGRRNFLDANIRDEEGIPGERADGRNLIKQWLDDKRQQNVRGEYVWSRKGLKMLDLDKTDYLLGLFSSSHCPYHGDLQRSHIEQADPSLSEMTEAALKVLKKNDKGFFLFVEGARIDMAHHETRARKSLEDTEEFAKAVQVAREMFSEDDTLIVVTSDHSHTMTINGYPFRDQDITGLANGPADDNLPYTILSYANGPGYYSTYGPHGRKHITDKDTADAKFEYLATVPLDSETHGGDDVGVFASGPYAQYFSGNYEQTNIPAMMAKAANIGPYANVGK*

>Woma_00008699

MKSITLVCILAVSLLLVSVLVQANNVTWGYVGIYDRLLATDYVQKSGSFFVKQTATVVYPPKGHISYVTLTAIKAWDWGGRKAFGYATLLQGGPGHRNSTIYLKSQSGKGLNFTIQYFGR*

>Woma_00008698

MKSLTLICILAVSLLLVSALVQANDITWGHVGRYDRLLATDYVHTAGRLLSKQTVTVVYPPKGHISYATLTAIRVLDKGGRKGFGYATLQKGGPGHRVATIFLKSQRGKGLNFTIQYYGH*

>Woma_00008701

MKLLIAFCLLASLATVAWTSSASWGKLLPYDYHLYTENVVLSPIKNNYLSVNINFPKPGQINTRNITVVYVYDRFTNNSGAQPTLLSGGPGFKFATINLKSPISRGINSTVQIYGR*

>Woma_00008700

MKFTLSFVLILALATNTLANTVVYGKRSSEHQLLYRANVIRPAIEGFSHDVYVSYPKDGKPKHSKHIAVILFTNNNNSTAQPVMAYDVDKDWSHLDSASIIVRGQLSQGIDSTVEFYGYDGNQSRGVNVTAIYVYDNLTNSSGALPHVIKHLRPTTNARFNFTIVLLRSQHAYVTITTVTLSYDMKWGKYSAGDTLLYRQRAIFYPVKGKKLNVLVAYPQLNSSFTPKDYITYIHVHDGFSNSSGAVPILATTAKLNKKIAQNSTIIILVGQRSNGINSTVKIYGRKIQK*

>Woma_00008702

MKFLITFCLLVSLATVALSSSASWGKRLPKDYRLYTENVLRTPIKNNYLTVNVNFPKSGQSNTRNITAIYVYDRFTNSSGAHPALWSGGPGFRFATINLKSQMSRGIHSTVEIYGR*

>Woma_00008721

MQRDRISCEVRREKENIRNKSEKMKNLLILLSLGLMLQLCWAEEKLLIRVPVSKIKSARTHFHEVGTELQQLRLKYGADVGGITPEPLSNYLDAQYYGPISIGTPPQNFKVVFDTGSSNLWVPSKQCHFTNIACLMHNKYDAKKSKTYEKNGTEFAIHYGSGSLSGYLSTDVVNIGGLDVKRQTFAEALSEPGLVFVAAKFDGILGLGYSSIAVDGVKPPHYNMYEQGLISQPVFSFYLNRDPSAPEGGEIIFGGSDPNHYKGDFTYLPVTRKAYWQIKMDSAAMGDLQLCKGGCQVIADTGTSLIALPPTEATSINKAIGGTQIVGGQYVVSCDAIPNLPVIKFVFGGKTFELEGKDYILRIAQMGKTICLSGFMGIEIPPPNGPLWILGDVFIGKYYTEFDMGNDRVGFAEAK*

>Woma_00008653

MLKNILKISVVLCLFAFTQAGNRTQITTHLSPEYSDEINTAPFVRVCNKLYYFGTSKASWFKSFLICRLIGGYLASFGTAEELNDLSKYLVANYPTDRSWWLSASDLDSKGDYHWYSTGESISYTAWSSGQPDNAGGNERCVHLWFQKTKYQMNDAKCDHKAFFICEAANPKTIVVTIFQRR*

>Woma_00008650

MKRIGSLIILFQALLIFCSSADEDDEEIFTDYCRPDLCSSGFGMHSGCLDGIRHNGKFQEPPCKPGTSELITLTHAEKNMIIDWHNYQRNYVACGGLMKYYNFKPACRMGAAKWDRELEATAYLHVSYCLMAHDKCRSTPKYPLSGQNLGWYSTNETGFNFTKAIHHHLISWYMEYQDVWEEILYNYTSESWHPPIGHFMISIHEKNVRLGCALAYVFELSDQSMSSIFTCNYAFTNVLYSPTYSYCTVPASKCETGTDHKYPYLCSTKEKYNWGEEVKLFPYRGDGTEAYDCGPSPDVVQNAPKYTIKERTTLMPSSTLEVPTLFTNIKQNLSEDAIKKTKTVKPSSTLDIPTKSPDLRQHSPEDIIKETKTLKPSSTLKIPTLITDIKQNLSEDAIKETTTLTPAKHSQITSKSNPYQPSQSKPRSGKPSSNIPRQKPPFGKNDRENYVEGIATLDPLIITGEPTQSHLRVDPSKPVPRQKPPFEPEKRANIDYEEYEMDLPISSSTQIWKQFFYQKIPKLAFLLLELKLIRIINY*

>Woma_00008624

MLPFKNIALFLGLLYSAHYIKAAALESLEKPNYNLFANELFSNIVDGKSSRNIIYSPASIQTGISLTFIAAEGETAEHLRKDLRLGQGDKTQVAKNFADFLRTSFKANHKEYAPQLKLANRLYISKDLVLNPEFDRISKEYYETETEAIKFDEIDLALEQINRWVEQATDNKIRNILQSSDIDADTTTLLVNGIYFKGAWEKPFNALHTQKAAFYLKEKQEQDVELMYNKNKFRFAELPDLDACALELPYNNSDISMLIILPHDKYGLEQLESKLKGQDLNEIASKMSEEILEVYLPKFRLEYELNLRETLEKMGLSYIFSPHSNFEGFFHEPLKRPRSERLKHKAYLNVNEFGSESAAATAFDNSQLSADLNAARTFRADHPFYFAIRCKSAVYFTGHLAQI*

>Woma_00008641

MKQFMQNHLNSNNTHQTLQQQQQNLSATTTQVKNYTNFLMSSTTPAATALMEDDVVGSALNHNLTASSLLGAGNTMAMDNMFIYQQQLKQF*

>Woma_00008668

MKRIGFFLIVFLALLLIPSSFADDDDSEEIFRDYCRKGLCPKKTQEHTGCKNGILHDGKFQEPPCKPNTSTLLNLTQELKDNIVDWHNYQRNFVACGGLMEYYHFKPACRMGAVKWDRELEATAYLHVTYCLFDHDKCRNTLKYPMSGQNLGWYQHDDPTFDHYTTPAIHTIGWYMEYQDTWEEVIYKYITKPGHP*

>Woma_00008684

MAKQFQIIFFVVFVVLSFVVVALARGKQCPPNQEWTTCGTACPDKCGQPAARFCTFQCIIGCQCKPGYLLNSRGDCVSPRNC*

>Woma_00008685

MAKLLKQIILLLAIVLMVVAIQAEEQCPKNQEFNNCGTACPPKCSDNDSNDGFVKPCTLQCIIGCQCKPGYLLNSNNECVTPAEC*

>Woma_00008686

MAKLFKQFVLLLAIVLMVVPIQAEEKCPENQEFTNCGTACPPSCNDDLLISKFCTKQCVIGCRCKPGYLLNSYNECVTPDEC*

>Woma_00008639

MGPLLLLFVALQLLYTLTWSKVVNDPQYALNTISDKLSDYSIFLPPDLSGDNDEFLDDDEDDITDVLPLVTTDEDKTEMKFHQPIESVNIDYNRNTIVAKFYKNHSKSMAKNGASGVKSKQKALQDNFIRFGKRSYEEFPLDNSLDGEFVDPNRRMRLERSQQTARDARGDNFMRFGRSVNTKNDFMRFGRGQNDFMRFGRAAAAGDNFMRFGRSAGQDFMRFGRAAAGQDFMRFGRTPSKDFMRFGRTPSQDFMRFGRTSNQNFMRFGRTPPSKDFMRLGRAPSQDFMRFGRTPASQDFMRFGRNPHHDFMRFGRSSPKIPVNTNFMRYSRPDNFMRFGRAPPQPHDNFMRFGKSLEKSNDKNSSQPMMGKNELKQAVKLIHEADKNADNENPVDKAIKVLFDKQQHDENDTDDSEELKKSLP*

>Woma_00008718

MKVSIFIGVFALATLANAQSLIDTVQQYNRPFPPVKKPLQQLILKPAEQIVEPQPPVVYDDEHHTPPLVVIDEEHPVTPPVAIEEEHHATHQVATDEEHHATPPVAIDEEHHVKSPVEVIEEIVQNIANVIKHKPDIDYYNGKMVPTPKYPKAPKKE*

>Woma_00008697

MLLKFSILLLICSIFTHNLSLAAKSENSDSHNAVWGAHSQRDVHLSRQIVMDKAKTMRIISGDYEYRPKPGDLNYGKTITQIQVTDQYTNGNGGYATLKDGGPSTNYAVIHLKSQRNHGYHFIIDIYGQ*

>Woma_00008696

MKTLLILSVITISCLVAVQAGNDYLWGKIGPKDTLISKKDVSKAFFVGLKVTTKYVYKQDALDAYTITAVKVTDTTKKNGAKAELLSGGPGSKGATIKFTSQRGKGIKEEVEIWGRK*

>Woma_00007000

MQVKIVEIVLATLCLLAFCQGRVIQEDELLEKMPSEFLEPMAWLPETTEAEVLEEEQELAELPEIPEEPQDFVNQTEVEIQARSGCSISIRGGLKAKQPLYIKPGTTEFYPFTNTGHIYVAAGKSIEIHCTSGFARPLGGKTHKATCVSGSTFRVNGVKHYLPSLLCTSWPAFVAKRSGKSCNGGTTLIKVGFELSASRFLKQYEVCFNEHEEVTRYVKHKLYPGSNHYETGVDRITFGTGGWFAGKNVDNLYTQAKQKETIDKALGYNADKYFESSKNIYLARGHMAAKADFVYASEQRATFLFVNVAPQWQVFNAGNWARVEDSLRAWVTKHKKTVDCWTGVYGVTTLPNKHGVQTPLYLAYDHNNNGLIPVPKLYFRVVIEPSTRKGIVFVGVNNPHLSLDEIKKHYILCPDVSDKVTYISWKKHSIKEGYSYACEVHEFRKKVTTLPSFSVSGLLV*

>Woma_00007028

MERAVIFSVIFVSCLLSLITAVPTPVNDDNTNEDIMPLFSNQNVDYNAMMSHVKDFFMYLPVMFTTFRETMSDFPKLAQGIRILTSPNFESTADDDKNCKCNSKPTQSSNEIDKSSHLF*

>Woma_00007034

MNIFFSVFIFFVYIANVQSIVKKCICETGLPGLPGNDGEKGYKGDKGVQGNQGLMGPKGQNGVAGPFGDTGAVGEMGFNGGPKGDRGAPGRDTACPPECQRFRSLIGEELRDNNYEPYTVYKVMPDGEFKAVKKLEPTKALKAFIQMIKMKNYEYSSYVDEMY*

>Woma_00007035

MISVLWTATLLGTLLHTTWASSTNCGCLPGLEGHIGYKGNRGEDGNNGDKGEPGIRGNQGVIGDRGPQGGRGPQGPKGPRTNLPLTFGPAGEAGPPGRCLCNVNRAFYGQPVIAGDYFLTDEDGEPNYAGDNIEEPFQEYETTNMPPVPATSPLTPDINLYMMSPEGVLIPVGNTKTFDVYAAANAAATTTTTTRRPTTTRPPTTTRPPHNYDDHDYHPPPKTVYVMGPKGSLIPIDILGITHKFTQGYGHHHHHSPPDHYHHHHHSPPDHYHHHPYPRPDRDHSYHHYPSKYGQQYHPGKGSTSFTKWFRSIFLGENDEKEEIYAEHDEDDDIDEDDVRSDDDVSIDDTFDDQLSTLVGKKVFEE*

>Woma_00007032

MVALKNIFVIALSLALCDNLTAHSVEDLEEQHMHPDFKDELNTKNRFVLRQGEDTNEFWLNLGKDFVKQQKQHKRNTSVAKNIIMFLGDGMGLTTLVAARNYIDAEHTKLSFEEFPYTGLSKTYSVDKIVPDSATTATAYLCGVKGNYGTIGVNAAILRDDCAAMKNSSNHVYSIAKWAMDSGKAAGFVTTARVTHASPAGVYAHTADRDWESNADIDEACGKEHGLADIAQQLLYGEVGSKLKVMLGGGKSAFVDKSLYENGWRTDGRNLIEEFQKLAATNAYVETKEQLENLDLTKTERILGTFNDGHIDYHLNTLNKPDNTQPTLKEMTKKAIQFMQQKSENGYFLFIEGGRIDMGHHDTKVRLALDETKEFSEAIALARQLTSEEDTLIVVTADHSHTFSVAGYQNRGSDIFSVVKSGTDKLPYEPLSYANGAGYYKHFNAQLSKRVDPQSVLTGDANDVFPATVPLDSETHGGEDVAVFASGPWSHLFTGVYEQNTIPHMMAYAACVGEGLKACDTA*

>Woma_00007033

MKIFYGFLLVCAWSCTVQAVIKNCYCEPGPPGPQGTKGDRGYKGADGKKGETGLTGAQGPQGPPGIKGEKGTKGIKGRAFGYKGQRGPKGPAGSCTPCPNRQERTIEYFPPNRTYIITNKRQLIEVQRIEPTPEIRQAIEELKKTNKWKDF*

>Woma_00006971

MHKLALILLGMASLAASGIITDRVRNGLDIIGGLGAQLQHIHGLHDIGLSGGVGLVNIGALNKQELMRQKFILDILQQVNQPLQKQELLMVINRNDVINEDLYMRPLTEEMLMVLDLLRQQQVLGKQDICTISNDEHIRQMVGLYRLLVTARDFETFKRLVIYGRQHINTEMFVNALILALAERDDTKMLVVPALHEILPHLYHQDSVKQQVEHLDTGVSTLKPDLVDIVGLGRPNRFLNPHWNTEQQQGGGLWGVWNRSQMWMPWRGLHRQLAMRRMMGGNVVEQNNSQMDKLVIQLPGQGLLTEDIGLKAYVNVLLDELIVKQDVVRNVGINTGRNMDISGIWGRRGSGNTRNRLFGLNKNDEDVEDNMQLNVNNQRRTMMGHGMNKNMDWDMDMQQRDVLGRRNIVESDNIMGSGRVGRVGLLRDDRNMRRGMWNMNRDNDDDDDDDVRQTIRNRLGGGQRGSNTVSVNDARLLFVGRRRKNPNNVINLGNKRNMNNMDDMEMISGGRRVDRNERRNEKEQQQRWKNNNRYDNDDDQEEQQQWNKRYTNRRNNLMNDDNMRMGGNQWQRQGQGQDQGIGSRGLDRDEFLNIVTRGNYGRKQMWQNDNDDDDDVVMQNRGRTNIKGGRHSSLDMNLPTTTIDDERLLHVNRRKLNDNVNVNMNERRSIWNNNNNNDDDDNIRNLNSWDIKRRNMLDDDDDDMIMMNPHLGRDIHTGGHRYRRSLDYQQVMSGVGDVSTNEGRINGKLLLQTLQQLVARLNVERIALGLPQLIDDQLTGIGRGGLLGNTLNQQQLTINRNLVNTDSHLSTQTVQTIEDIIQRIDGVLQQNKRQILSWSTLDNDYNDKLDQMGLMLAGQIQEIGLLDIIGDILKQSSSQLRKGQVANLIENENIQVLLAGIVKVIDENVQQIFKTRQDHISNVRDITINNVDVDKLQTYLEQTVVDLSNLSQDNIDLSVHGDGGKKKMIVGQVPRLNHKSFHIDIDVTSERQQQVVVRSLLVPKVDGHNNVLPLKQQRQNAILLDITTVNLKTGRNLIKLKSNDITLTSCDTTPYTKIYERVMRALEGETVMGHDQICGQTDLLPHRLLVPRGRVNGLPMQLVTVITPVQNPAMLGHSVTGLDVLLLDHLPLNYPLHCDITTSLEQIAASMANVLVKDVKIYHEDNIKMPLINNIMY*

>Woma_00006972

MHKLALILLGMASLAASGIITDRVRNGLDIIGGLGAQLQHIHGLHDIGLSGGVGLVNIGALNKQELMRQKFILDILQQVNQPLQKQELLMVINRNDVINEDLYMRPLTEEMLMVLDLLRQQQVLGKQDICTISNDEHIRQMVGLYRLLVTARDFETFKRLVIYGRQHINTEMFVNALILALAERDDTKMLVVPALHEILPHLYHQDSVKQQVEHLDTGVSTLKPDLVDIVGFGRPNRFLNPHWNTEQQQGGGLWGVLNRSQMWMPWRELHRQLAMRRMMGGNVVEQNNSQMDKLVIQLPGQGLLTEDIGLKAYVNVLLDELIVKQDVVRNVGINTGRNMDISGIWGRRGRSNTRNRLFGLNKNDEDVEDNMQLNVNNQRRTMMGHGMNRNMDWDMDMQQRDVLGRRNIVESDNIMGSGRVGRVGLLRDDRNMRRGMWNMNRDNDDDDDDDVRQTIRNRLGGGQRGSNTVSVNDARLLFVGRRRKNPNNVINLGNKRNMNNMDDMEMISGGRRVDRNERRNEKEQQQRWKNNNRYDNDDDQEEQQQWNKRYTNRRNNLMNDDNMRMGGNQWQRQGQGQDQGIGSRGLDRDEFLNIVTRGNYGRKQMWQNDNDDDDDVVMQNRGRTNIKGGRHSSLDMNLPTTTIDDERLLHVNRRKLNDNVNVNMNERRSIWNNNNNNDDDDNIRNLNSWDIKRRNMLDDDDDDMIMMNPHLGRDIHTGGHRYRRSLDYQQVMSGVGDVSTNEGRINGKLLLQTLQQLVARLNVERIALGLPQLIDDQLTGIGRGGLLGNTLNQQQLTINRNLVNTDSHLSTQTVQTIEDIIQRIDGVLQQNKRQILSWSTLDNDYNDKLDQMGLMLAGQIQEIGLLDIIGDILKQSSSQLRKGQVANLIENENIQVLLAGIVKVIDENVQQIFKTRQDHISNVRDITINNVDVDKLQTYLEQTVVDLSNLSQDNIDLSVHGDGGKKKMIVGQVPRLNHKSFHIDIDVTSERQQQVVVRSLLVPKVDGHNNVLPLKQQRQNAILLDITTVNLKTGRNLIKLKSNDITLTSCDTTPYTKIYERVMRALEGETVMGHDQICGQTDLLPHRLLVPRGRVNGLPMQLVTVITPVQNPAMLGHSVTGLDVLLLDHLPLNYPLHCDITTSLEQIAASMANVLVKDVKIYHEDNIKMPLINNIMY*

>Woma_00006973

MHKLALILLGMASLAASGIITDRVRNGLDIIGGLGAQLQHIHGLHDIGLSGGVGLVNIGALNKQELMRQKFILDILQQVNQPLQKQELLMVINRNDVINEDLYMRPLTEEMLMVLDLLRQQQVLGKQDICTISNDEHIRQMVGLYRLLVTARDFETFKRLVIYGRQHINTEMFVNALILALAERDDTKMLVVPALHEILPHLYHQDSVKQQVEHLDTGVSTLKPDLVDIVGLGRPNRFLNPHWNTEQQQGGGLWGVLNRSQMWMPWRELHRQLAMRRMMGGNVVEQNNSQMDKLVIQLPGQGLLTEDIGLKAYVNVLLDELIVKQDVVRNVGINTGRNMDISGIWGRRGRSNTRNRLFGLNKNDEDVEDNMQLNVNNQRRTMMGHGMNRNMDWDMDMQQRDVLGRRNIVESDNIMGSGRVGRVGLLRDDRNMRRGMWNMNRDNDDDDDDDVRQTIRNRLGGGQRGSNTVSVNDARLLFVGRRRKNPNNVINLGNKRNMNNMDDMEMISGGRRVDRNERRNEKEQQQRWKNNNRYDNDDDQEEQQQWNKRYTNRRNNLMNDDNMRMGGNQWQRQGQGQDQGIGSRGLDRDEFLNIVTRGNYGRKKMWQNDNDDDDDVVMQNRGRTNIKGGRHSSLDMNLPTTTIDDERLLHVNRRKLNDNVNVNMNERRSIWNNNNNNDDDDNIRNLNSWDIKRRNMLDDDDDDMIMMNPHLGRDIHTGGHRYRRSLDYQQVMSGVGDVSTNEGRINGKLLLQTLQQLVARLNVERIALGLPQLIDDQLTGIGRGGLLGNTLNQQQLTINRNLVNTDSHLSTQTVQTIEDIIQRIDGVLQQNKRQILSWSTLDNDYNDKLDQMGLMLAGQIQEIGLLDIIGDILKQSSSQLRKGQVANLIENENIQVLLAGIVKVIDENVQQIFKTRQDHISNVRDITINNVDVDKLQTYLEQTVVDLSNLSQDNIDLSVHGDGGKKKMIVGQVPRLNHKSFHIDIDVTSERQQQVVVRSLLVPKVDGHNNVLPLKQQRQNAILLDITTVNLKTGRNLIKLKSNDITLTSCDTTPYTKIYERVMRALEGETVMGHDQICGQTDLLPHRLLVPRGRVNGLPMQLVTVITPVQNPAMLGHSVTGLDVLLLDHLPLNYPLHCDITTSLEQIATSMANVLVKDVKIYHEDNIKMPLINNIMY*

>Woma_00006997

MFKITLAILVAVLLLGPAYARVYNRCSLAREMHRLGVPKHELARWTCIAEHESGYNTKAVGSLNHNGSRDYGIFQINNYYWCSPPSGAFSYNECHIRCEDFLVDSIEPAVKCARLVLRQQGWKAWSTWRFCNHRLPSIDHCF*

>Woma_00006894

MARVWPVLGLFMLCCALAASQETNDKLEGIDIEEACADRPADEYFRLDTEGDCREVYRCDRGETGKTRLASIKCSGGLAFDITRQTCDWRTNVKNCDEVEKPRKVKPILKTDEPICPEGKLSCGDGECLDKELFCNGKPDCKDESDENACSVDDDPNRAPECDPTQCALPDCFCSADGTRIPGGIEPQQVPQMITITFNGAVNVDNIDLYEDIFNGQRQNPNGCSIKGTFFVSHKYTNYSAVQDLHRRGHEISVFSLTHKDDPNYWTSGTYDDWLAEMAGARLIIERFANITDGSIIGMRAPYLRVGGNKQFEMMADQFFVYDASITASLGRVPIWPYTLYFRMPHKCNGNAHNCPSRSHPVWEMVMNELDRRDDPTFDESLPGCHMVDSCSNIATGEQFGRLLRHNFNRHYNSNRAPLGLHFHASWLKSKKEYRDELIKFIEEMLGRNDVFFVTNLQVIQWMQNPTELNALRDFQEWKEKCDVKGQPYCSLPNACPLTTRELPGETLRLFTCMECPNNYPWILDPTGDGFSV*

>Woma_00006895

MAKLFVVFAVFALAVTAATASNSERVKRQATTEEPKKEESFEKELCKDKDAGEWFRLVAGEGDNCRDVIQCTSSGLQAIRCPAGLYFDIEKQTCDWKEAVKNCKFKNKERRVKPLLHTDEPLCQDGFLACGDGNCIERGLFCNGEKDCSDGSDENTCDIDNDPNRAPPCDPTVCVLPDCFCSEDGTAIPGDLPAKNVPMMVTITFDDAINNNNIELYKEIFKGRKNPNGCDIKATFFISHKYTNYSAVQETHRKGHEVAVHSITHNDDERFWSNATVDDWAKEMAGMRIIMEKFSNITDNSVVGVRAPYLRVGGNNQFTMMEEQAFLYDSTITAPLSNPPLWPYTMYFRMPHRCHGNLQSCPTRSHAVWEMVMNELDRREDPANDEYLPGCAMVDSCSNILTGDQFYNFLNHNFDRHYDQNRAPLGLYFHAAWLKNNPEFLDAFLYWIDEVLANHDDVYFVTMTQVIQWMQNPRTVSEVKNFEPWREKCVVEGKPSCWVPNACKLTSKEIPGETINLQTCVRCPNNYPWINDPTGDGFF*

>Woma_00007031

MRLYVVLSALVWALLATAHDDGYHPKFPTPRSYQPLAEEQTTEFWINKAQDILAHKLQKLNQLNTNRAKNLILFLGDGMSVHTVTATRDFLGDSSKQVSFEKFPYLGLSRTYAVNKRTPDSASTATAYLNGVKANYGTIGVNAQVPKYDCYKANDTKTHTESLAKWAQDAGKWAGLVTTARVTHASPAGVYAHTAARDWENDQAVKDSNCSPEVNVDIARQLVEWPVGKELHVVMGGGRRNFRDQTMHDEEGILGYRSDKRDLIQEWLQEKSRENKAAEYVWSKIGLEMVDFNKTDYLLGLFSPSHCPYHGDLKRDHLENSDPSLSEMTKAAIKLLSKNDKGYFLFVEGAKIDMAHHESRAHKSLEDTAEFARAIELARNLTSEADTLIVVTSDHSHTMTINGYPNRHESIFGLAPKMADDGLPYTILSYANGPGYSKTYSSKKGRRDLSTADLNDPKYRFMATVPLDSETHGADDVGVFASGPSEHYFTGNYEQSNIPALMAHIANIGPFADDKL*

>Woma_00006943

MTMSLQYSLVVVACVVLAVQADVSHLGYNRFNGNRNAPLRLQRLESRPTPYPSAGYRPNREFNLPREQPLFPLANPQQSSSGFDNLSEPQQKPQLVYGAPGVDSQRFPQRQQQNSNGFNNFAEQGNTQQQHTSSSGFDNLSEPQQKPQVVYVVHGVDNQRFPQQQQQQNSNGFNNFAEQGNTQQQHISSSGFDNLSEPQQKPQLVYGAPGLDNQRFPQRQQQNSNGFNNFAEQGNTQQQHTSSSGFDNLSEPQQKPQLVYGAPGIDAQRFPQQQQQNLNGFNNFAGQGNTQQQHTSSSGFDNLTEPQQRPQLVYRAPGVDNQRFPQQQPHGQRPQNSNGFNNLAETQQQDFNVAEQNQEFENSNGFNNLAEPQQRPELLHAAPNSPQAEQNNGFDNLAAPQKRPQYNYNSPARLKQLLIASLKQARAQLQNQPQTFYSASAPQQPQQSQQIETQTQAQAESQTSRLTAVKPDRLTDNREDREEETAESALDIETTTEKDSIAQNATNGTAVAANSVAAPANGVVAYYPATSFTYVQPYSAAYVAAAANYHTVQPVNAAYAVGSHFLPQQYALAAAPAQLQAW*

>Woma_00007131

MSSKHIYSTSNLTDLQLKEQGNCLFSARKYDDAINCYTKAIIKNPNNATYFTNRALCHLKLKRWELSCQDCRRALDIDGNLLKAHFFLGQCLMEMDLYDEAIKHLQRAFDLSKEQKQNFGDDITSQLRLARKKRWNVMEEKRICQEIELQSYLNRLIKEDMENRLAKLKLDETTNEEQLKDKQQEIEQECDDHVKELNNIFAKVDERRRKRDVPDYLCGKISFEILTDPVITPSGITYERKDIEEHLQRVGHFDPVTRVKLTQDQLIPNFTMKEVVDAFIAENEWALDY*

>Woma_00007040

MKYFVYLYALSLLCFWKNFVRAAVSHTTTTSDDNFLLKESLLCDNTHIETKLETLELRIEMMEDQLNNLFSSLDKKINDLPRKFEIMHEKLLKENKFYFEELKASLNEQIQNQVRAFVQPPVDDKIFILSDIKEMLKDYDKALDNIVSNQKQSEKLLKDANCERHTTTLANTMSNKLAILHKLDGKLATVESSGSKTETEQLKFRCDLQKYLDRKEVLKGTCKDDYNNLFCTQPYPRSCADLSASHCVDDKCCISNHLYGPESFLVSCNNKDQGGGWTVIQRRINGSVDFYRDWSEYKNGFGDVNGEFFIGLDKLHALTTTLKPTELLIQLEDFNNTTKYAKYDDFQVGNETEYYKLIKVGSYSGDAGDSFSYHQGYNFTTKDRDNDVDKSVNCAVDKTGAWWYEKCMWSNLNGKYYANGQPLNGKRGICWNNFHGYTYSLKFVQMMIRPKKL*

>Woma_00007048

MMMLSTNERSQFRKALLSFTILFVICCAPGKSQQLTSSKSFPITTLINAKWEQTPLYLEIAEYLADENPNLYWDFLKDITDLETTLSSYDTPSQSYNAAVNVVRRRLTDLQMPLMKLIVSMHSLTPRIQTHFQIASEILGHGACADSTTFAQVGSELACTLDELRQKLNLPKQDTLDAEVEIYAFDHVYPGSENNTRTVVLYGDIGSKNFAQYHYLLSERALTGDIKYVTRHYLHKNEQRSQRRVRLSGYGVELHLKSTEYKSQDDAPKPADDVGNKSAEDAGLESEVHGFDFKVLKNRFPTLSHSLDKLKQSLLQGNEEIAQLKAWEFQDLGLQAAQAIAEIDGEEALKILQFTAHNFPMQAKTLLSHKISEQLRNEVKHNTEAFGRTLNIIPPDGALFINGLFFDADTMDLNSLMETLRSEVRVLESLHNSQVKGSLASALLALDFAGSGSKEFAIDIRDSSILWINDIESDPQYRRWPSSVMDLLRPTFPGMLRNIRKNVFNLVLVVDPLKPTGRSLIKLAESFVVHSAPVRLGLVFDARKATDDTQDDYRSLVCAFNYVTQKKDPRAALSFLTDVFAAVDSKQEVRLKHIKMQLKKTFDKLSGSVVDDILDEDSDYDYGRQLALEFVDRLGFTESPQALLNGIPMPQNILSSDSDFEEAIFSEIIQQTTTLQKAVYKGDLTDSDNLLDYLMDQPHVMPRLNQRILGNNENAKFLDLTGEPHTDLNNVKALAQLSNRDMTSTLLNNIKYFEGKHSYETIGESKLHFLTIWLLADVETKAGKELLTNALKYVKAGSSVRIAFIPNAEGSDMSRKDNFNRLVWAAQQSLKPQQATEVVLKWLKQPTDKWEIPSQVNDILSSTELHMKILRVYAQRVLNLKAGERLIIGNGKIIGPITDNELFGIEDFGLIDRFNAIQYGDKLRKVFKMKSIDMDESEFGSDTLLKLYASLIPRQSKTRFKIPDDVKEAHSVVKLSAKNKELPHFEINAVVDPASRSAQKLAPILILLRNTVNCNMKVYLTPVNQHSDMPVKNFYRYVVEPEIQFLPNGELADGPIAKFTGVPANPLLTQNLQVPENWLVEAVRSVYDLDNIKLSEIGGPVHSEFELEYLLLEGHCFDASTGAPPRGLQVTLGTQDNPTMVDTIVMANLGYFQLKANPGVWTLRLREGKSADIYDITYAEGPNTVHQEGNTQVVISSLKSHVIKLRVTKKPGMQQADLLGDDDSQHQSGIWNSIASSFGGGSSSSDNEVETINIFSVASGHLYERLLRIMMLSVMKHTKSPVKFWFLKNYLSPQFTDFLPHMAKEYGFQFELVQYKWPRWLHQQTEKQRTIWGYKILFLDVLFPLNVRKIIFVDADAIVRADLKELYDMDLGGAPYAYTPFCDSRKEMEGFRFWKQGYWKSHLMGRRYHISALYVVDLKRFRKIAAGDRLRGQYQALSQDPNSLSNLDQDLPNNMIHQVAIKSLPDEWLWCQTWCSDNAFKNAKVIDLCNNPMTKEAKLTAAQRIVPEWKDYDGEIKSLMSRIEDHENADHTEQMLISGSGEMSSKSRDQNQDDDNEGDAFKHTEL*

>Woma_00007105

MKLKYKILITFCVVLLLCNQNCWAWRAFKVILTSFPHEFNDKLLNVKFQIQNTSNTESPAINVQLNVLEDIDDISLTYAVGIEGNQANNYTIVLNRTVHFCKFLMQRTIDNTLRTIYEDILKRGRFIKMCPFKRGTYTLHEYHIDEELLPQYVPEASFYVDIKLEKANNGDMILKGRLNGKIDKSKGFNNLKMFSLG*

>Woma_00007155

MRFLTSVGIVMMAICGLASAGLQHHGHHQNYHQHDDHHGCSYAVISKHEEPAVKHELSHKAWEEHGHELIHHIQHKSDEHEDYDAGHGHENSHDYYAPPKYEYDYSVKDEKTGDSKKHWETRDGDKVKGSYTIKDADGTTRTVEYTADHKNGFNAVVKTDGHAHSEHESHSSGGVHESHGYAHYNH*

>Woma_00007154

MRVLSVCVVVLATSCVALAGYAHGGIGGGSSYTVVTKHDAPIHQIKYVGGLHDGGYLGGGAVGLHGGYLQKGSDVGYGYGHYDKYDYHHKYPKYQFDYGVKDLKTGDIKNQWEHRDGGLVKGGYSLKESDGTTRVVEYSADDHNGFNAIVKKIGHAHHPQIYAKGHLGGYGYGSGLEYGHYGAGYDYDQGHHGHASSYVNVKQL*

>Woma_00007156

MKFLAVGTFLAICGLALAAPSYHHGGHGTSYAIITKHEIPHHHHGWEEKSWASADGGWHGGSSHYDQGWDKHDYDDKHAKYEFDYGVKDLKTGDIKNQWEHRDGDHVKGSYSLKEADGTTRIVDYTADGHNGFNAVVKNIGHAHQPVHYGGGWEGGYDHGHGHASSYIKVNQY*

>Woma_00007152

MKLFFAFLCLIGTVWAELTPKSIPDCIHALNAAYGYTPPEVKFSISPGTTSLKTSPSLSTYSENGKLQYAAVGSSSSSYESSAGIAGYSHGGPLQVTKYVEPVKTASYEPAVTYAKGGSITYADKSPASKFAAVVPSGIEFKNLVTPAISTKQFSNAYLPPPQGISSKVETYQSPGYSYSHSTPGIAKSATYTTTGGVDLSKIAFNAPAVTTKIESFTAPGYTFSKQTPGYSKIETYTSGGYSKSGNLESIFSSGAAYPFASAISYAPKISYGAPTVTKLATITSSNKLASFSPQPLFSKAFAPTPAIYAPTISKGYLPSIETSGYTKSLVGGISHQYVSKPAITTQYVSSPAKVATYAAPVASHYISSPVKVATYSTGSAHLTAPAQAAYVAPVVAKAVATYSGSASHYAASGGGGGAVSHQYVSKPTAHITGYSAPAIAKVATYSAPAVAKVATYSGPSVTHISSTGGALSHQYVSNPASHITAGYVAPAVAKVAYATPAVAKVATYSSRGSGAVSHQYVSKPGSHVTTYAAPAVAKVATSYSGESLSHYSSSGGGGGGGAVSHQYVSKPAAPAVAKVATYSAPAAVKVASYTGESLSHFSASGSSGGAVSHQYVSKPTAHITGYAAPAIAKVATYTAPATVKQASYSGESSSHYSSSGGGGGAVSHQYVSKPAAHVTAYAAPTVAKVATYSAPATVKIASYSGESLSHFSSSGSSGGAVSHQYVSKPTAHITGYAAPAVAKVATYAAPAKFASYAGESSAHFSSSGGGGAVSHQYVSKPTAHITGYAAPAVAKVATYAAPAAIAKVASYAGESSAHFSSSGGGGAVSHQYVSKPTAHITGYAAPAPAVAKVVSYAAPAVAKVATYSEGSSSHFSSSGGGGGAISHQYVSKPAVIAAVPDVAKLSTYEAPAVASYSGGSSGSSVHYSSSGGAISHQYVSNPAQATVIAAAPAIAKVASYEAPALVKVSGHSGGGAVSHQYVSKPAPQSIAYAAPVVAKAVAYTAPAAAVGNVGSYSAGSSAHFSSSGGAVSHQYVSRPAAHVTAYAAPAVAKIATLTTPAVSQYVSAPAVAKVATTYSSSSGGAISHQYVSKPAAHVTAYAAPAVAKVATYATAPAVAKVATYVEPSVTKYVSSGGGGAVSHQYVSKPLVHLTGYAAPAVAKLSATPVVAKVGSYSGESGSHYASSGAISHQYVSKPVPAVTHYTSGASTISSTSSASKPAQTAYVAPVVAKVATYAAQPAVAKVATYQSRGGAVSHQYVSRPQVLGSIVSGPAIAKVANYAAPAVTHYSAGPAIGTKLTTISGGGAHYSSGAISHQYVSKPNLVGSAYAQYATGPALHPYHALHTPNVNPLFTSSSITRYGSSGAVAQQYVAKPVQLAGPALSKVTHGAVSHQYVSKPIASAVVAGPAIAKVSSYGSLSYGSKLSSIDAAHIAQPLQAVHLTAPAALHGAGYAKIQTSSLHTASGHLSGGGAISHQYVSKPLQGLALDKPAALTAPIVVDGSAHLTTGSGKYSSLSAGYTTSSHGGSISHQYVSQPSLVKGGSGLATGGHLIAKPLVAPALQISSSGHGGYGGHGAVYTTGGGAISQQHISKQGLGLLSLHGHGIGAAQHGLAGGYYGAIALGHGATSPALGIHGVLGLSSAGHGISGSVHGLKGLALGHTSSRHGNLAHGSLSHLGPLSGYDHGIGGIGPLGAGFYRYAPAVPALSSHSLSPAAFLKSAPIIKPAKIKIMTEKHLEYFNDHPRYAFEYGVNDPTTGDIKHQREERDGDVVRGEYSLVEPDGNVRTVKYYADWETGFHAEVINSRDSVKTLTKRTAAVSNKS*

>Woma_00007038

MDLRSLTYEEMCQMELMHTGSNCGELPLPPPPHQHQQHQHHHLASTNPLNTVTDSAAVNNQTTLTALTSTGCANTNPTNNTTTATMLPIMVIPTTMMPPGTQFIAATAAGTTPAATPLTESDFSNDSQQQQQTQQFALSAATAQPTHFYQLQPTHILAAAAPTNTEFTLASAPTTALFLQPTAAAAAANVNTNTNGGEQLPHSVHQQQIPQQTVNILYTGTLTKTPQLTAAAVGATTPLTRATLECSTLPRNLQSKTLNAAALANTQDESDFEGSECGASVSTTTCIPHFRFGGCGVILQPAQNFNQQQHGEPQVIGCASLSHMMGSFASAAGDAMHSGSRNLAGTLSRNRRSQTTSADGGGGGITTQTPKRVSFKGVDMPTPPPPPPTAEELQMLDAGNHHGPNMDAFNNCSYMNFDNFMDYQTRDGSTMVTIVLLQRNFFSEFELNE*

>Woma_00007037

MKYYFFSSALSLLCFAISQNFVQAEVNHITTDHNLLKESWKRMETLGSTISNKLATRDKRDDNLANSESNSVTETLQLRFIRDAGAEGENQNLLCTCQSYPASCADFDRTNCADNKCRISNHLYGKRSFLVSCNDNDEGGGWTVIQRRINGSVDFYRNWSEYKDGFGNIDGEFFIGLDKLHALTTTLQPVELLIQLEDFNKTRKYAKYDNFQVGNETDNYKLLKLGSYTGNAGNAFTFHVPYSFTTKDRDNDNEDGKNCAVVKTGAWWYNACAMTNLNGKYYKDGVVPKDERGIYWYNFHGFTYSLKFVQMLIRPKNFK*

>Woma_00007111

MMAKNKLKFIFTLLTTLICQVCQGAKQRSPRAAYFLCSEISYNPLYLTEHFMVIAPNRLTVNITLNLAQDFLNDPWADINLATKNRKDNTYLTIFQYDVNVCYILGTHDNKNPFNKFLRGWINNVWKYGNLPAECPIKKNNYSFINLKPEKLYIPHFIPLGKYRGIINTYFRKDLNKDSIANFTIYIDIK*

>Woma_00007110

MAKFVVILKVFIHVLFFNLLPPSLQARSIEFRNSTSKWNPSYFSNFTIYTSNDSVSCEFDLIRPIINDFKADIELEIRLANSKKYQKLFGYNVYVCSMVIAFKDTMFKRIFKSLLKYSNLMQNCPINEGHYYLHDWRMESSLIPSYLYAGDYRFKSRGYLHKTLKNRTKVDDFVISVTMDTVIVDK*

>Woma_00007071

MFKLIGFLVLIVQQVQCQIGFTSVPCGTQRNPKIVGGLEAERHEMPYMVSLTRRGGHFCGATIVHEKWILTAGHCICNGLNKFMKPSQIQGVMGLHSISQYINGIDNDRNGDGPVRVDFKSVIPHPNYKCANAKNDIALLELIQPIRFSRHIQPSCLSSADSVRNHENEVATVSGWGWTQENQILGDRADILRKAAVRIWNNNVCERSYQLNGRPNSVITDTQMCAGYENGGIDSCWADSGGPLMSKENFLIGVVSTGIGCARPGLPGIYTRVSKYVHWMESVIFPHN*

>Woma_00007158

MKSPITFLLIILLNCKQHFQVKAKDFKVVAALANEEAKTAKFSTLKLAHVIFRHGIRTPVNTYANDPYRNETFQPFGWGHLTNDGKKDLFQMGKWLARRYQLFLPAYYKPDSLYARSTASPRTLMSMATVLAGMLPPNNTPMKWNAKLNWQPIPIFSEPLDKDIELRMQTDCPAYDELRENITQQQTEFENLYKELTKLTGENVTRPSDVNSIFITLWAEQLYGLKLPTWTRNYYPDKMRFLAEQSYADLVATTEMQRIKAGPLIKLMIEQIQAKINGTLEPMERKLFLYAAHDWTITNFLIALKVWKRQMPNFAALILFELHQRAENEEHYVELYYQNGPKSPLEALTLPGCTMRCPLSRVLELIQDVLPTDTYEEMCNSF*

>Woma_00007153

MSIDIPRKWLNSLLIISLTFTIYAKAVNLQPIHVAAEDEGEEPEETEMMEYAPQFDHPEYAFSYGVKDLHTGDVKSQWESREGDGVKGHYSVLEPDGSIRTVTYTADAKSGFNAVVKTVGASSHPITESPNGLESKNDDTSQSKINHYSRDQEHIVLSSDIKPIKKPIEDLTHSHPKIPSLIEFKPHARIRQVPMELEPGMRERLKEAREEYYQKYVAKETKEEEPDSDFQPSGGVFASPYENEENEWRAVVKDNTDYSSHQNYGLSPGYYHKPEDYALHQPQYYESKYQHKHNQGNSHSTHSSGGHNSHSVSSLNTDFIASKPLNTHRPTSNVGSQADTHINSIPFHNSKKTVLKTTPGLKHYASLPNKYLNNKPRRDYSSYFQRSKSKKPRKYETLKKGAVALAEFEEVQSAASASLIQSMIKRDKKHLTPMYAKNYGINSAEGGFRPFRRL*

>Woma_00007102

MKFSATLALAVIVGLVASGSALPAQKRMIYYQRPAQYHVAPQRMMYVQYMHQSSPFARSTQGAEALVAGQSVATGTYLKDCNHAEADISASFPAVAFEAPASVDIVAEHGEDILSEAGATQAEVETYPAAGEAAVPGFNAEVPADVDIVAEENEVPDDDAKVPREFNLDGAENSNAAGDVFPAQEEEQQAHELHAVEAEAPVEGPVEAAVDGSGVAPAEAGVAAELPAPAAISPVAKPVNRYLPAKKKVYVQLDQSVESAEETGLAAADSVQEEEEEDDFVAPVAPARRPARRPVGKKPAKPAKKEPKPLPVGTFFPVHFGGTKGGAIAVANSFSTGEGGSATSHAIAYGSPDAASLRGCVSCKKH*

>Woma_00007104

MTLVKQIIALSLCILLCINCIQAEKVMRPKFKDVKLWSDDKHLSHTITLDANDPHINFTFNVLQELHSVDIHTEARLKQKLNPDYFLNLNTTMNLCNILNWKLKSPVGSFVRTFLKEYGHILEKCPIAKDKYYLHKFWLPEDTTLAVIPEMDFEIDFSAYHVDAKKQRTMFLNDHFTGELVVQDVTNVKPGVLSLLPKMAG*

>Woma_00007203

MQFYNRHSSTHSAPAKVSTLDVQKEETPSCYSTTENFSPPEEETPEYIDITQQPIQHLETGIDNWHIIGMYLASQMREIAKTNRRAANELHIQLVKTVIEATEQAERY*

>Woma_00007192

MKLFVLLACTLAVVAARPEPPKSRYSAPAPQKEYLPPIPAEQYGPPAAPQQVTQPLPVYGQPAPFYGPPAPETIITKNVYVHVPPEEPEPEPYPPAAPIQTAVPKKHYKIIFIKAPNPPTPVRQVVPPPVQDEHKTLVYVLVKKPEEQQPLIIPSPAPTEPSKPEVYFIKYKQQQQKPAEQYGPPPAAPASEYGPPPAEKF*

>Woma_00007160

MTRDLVLLSRSLVTSGHQVVCHMQNSTELSLSQQVQLEFISFRRTLQLSSKALTRSKPLQHVKDIRYDIQSENIKERIHLLPLRLPFTWGLKLRNI*

>Woma_00007184

MTTNTKGMTFYLLAAHLPVCSLAQQQKFRVTPHDLQVLEGAEAMLRCEVTNLAGAVQWTKDGFALGFSAVIPGFPRYSVLGDRKQGAYNLRISNASITDDAEYQCQVGPARLNSAIRANAKLTVISPPSSIEIKGYANNAKVEVRENQDLTLKCIVSNSKPASQIVWYRGNVEYKPENRNDKVEETTPKRFTTTSSLKLKPTADDDYTEYTCQAKHKALAPDMPMRSTVQLSVLYPPGPPYIEGYTQGETLRRGQTVELICRSRGGNPPAQLIWYKNGSQIRMAYRTSGRLSENIYTFTAEASDNKARFRCEASNVMSQTPLKAEVELTVLCK*

>Woma_00007357

MKFLIIVFAICSSLALVAGQFLFGQLPNINSHSLDFPQPTQVRDPRQNRGPVVFPPSPPDAIDESSGVVVGASGYGFVPPQQSPSYFTTAALRALSQSTSYAEFNYYNFPYLQRFRF*

>Woma_00007281

MTFKVILTLSLFGLILNNVKAEDTEITKFMKHLEVIPDVVDEGPKNFLKVTYDNGIQADKGVELTPTQVKNQPKVEWQAEAGTYYTLIMTDPDAPSRKEPTMREWHHWLVGNIPGDQLSKGEVLTEYVGSGAPKDTGLHRYTFLLFKQPKKLEFDEKHLTKTQAAGRENFSTKAFVKKYNLGAPLAGNFFQAQYDDYVLELYKQLGL*

>Woma_00007280

MFLKIFKLLFILHLTAAEDSEIAKAFKEHELIPDVVNKGPKEILKVSYDSGVSIDVGKELTPTQVQNPPKVEWSAAKGKYYTLIFTEPDAPSRQDFSMKEWIHWLIVNIPADGDLSKGDLVYDYQGSGPDKASGIHRYVYLLLEQPDKLQFDEQYSAANSIVGRPKFSTMKFLQKYGFEDTPVAGNFYMAQYDEYSPILWAKIGLSE*

>Woma_00007267

MGRITFWFLLGLTLFAVSSTDVYASSDVGPTESECRPYIEKAINDLKTGEEKEELVPSPDTTSGEEETTKETDKHDHDDDGAASGEMLGDQPEIDVDGDGIPNEKDNDIDGDVIPNEEDDDMDGDGVPNTQDDDIDGDVIPNEKDPDIDGDGVLNINDTDLDGDGVLNVVDSDLDGDGVPNIVDGDIDGDGIHNVSDNDLDGDGIPNDEDNDMDGDGIPNDEDDDIDGDGIPNDQDDDIDGDGIPNNEDDDIDGDGIPNHEDDDIDGDGIPNIDDNDMDGDGIPNTEDDDMDGDGIPNTEDDDIDGDGIPNDQDNDIDGDGIPNDEDNDIDGDGIPNNEDNDIDGDGILNHEDSDIDGDGIPNDEDDDVDGDGLPNEDEPKSLDVDKDGIHNDEDSDIDGDVLPNELDSDMDGDGVPNKLDKDIDGDGIPNVHDEDGHEPADIQPGAVPVKDDVDKRSKRSIEPISDMDNDGIPDDEDTDKDGDGIPNENDDDIDGDGIPNHEDNDIDGDGIPNENDTDRDGDGLLNEHDDSDGEADDKKTTKEEDDIQPEDELLWASKKHIDLDGDGIPNEEDEDIDGDATPNEQDEDMDGDGILNIHDDDIDGDSIKNERDLDMDGDGVLNIHDSDLDGDGVANILDDDIDGDGVKNIHDTDIDGDGVHNIHDDDLDGDGIHNENDDDIDGDGVPNHKDTDIDSDGILNHEDNDIDGDGIANEHDNDYHKFKATSRIVDIDGDGIPNEEDDDIDGDNKPNEQDDDIDGDGIPNIEDDDIDGDVIPDEEDPDMDGDGIPNIADDDIDGDGIKNIHDNDIDGDGIDNVHDEDLDGDGIPNDHDEDIDGDGIPNKNDLDIDGDGILNHEDHDVDGDGIPNDKDHIVHRSKAMPKIVDIDGDGIPNEDDDDIDGDVIPNEQDEDMDGDGILNVHDDDIDGDVIPNEEDADIDGDGVLNIFDSDLDGDGVANTIDEDIDGDGVKNVHDIDIDGDGISNVDDTDLDGDGISNDHDEDIDGDGVPNMNDTDIDSDGILNLKDHDIDGDGIPNDQDDDRHRVEALPKEIDLDGDGIPNERDTDIDGDIIPNEEDDDIDGDGILNIHDDDIDGDVIPNEYDLDLDGDGVLNIHDSDLDGDGVANVIDDDIDGDGVKNIHDPDIDGDGVHNIYDNDFDGDGIPNDQDDDIDGDGIPNAKDNDVDGDGISNVEDSDVDGDGIPNHREIFKHKLEALPKVIDLDGDGIPNEDDEDIDGDKKPNEQDEDMDGDGILNIHDDDIDGDVIPNEQDPDMDGDGILNVHDADLDGDGIVNIKDDDIDGDGVKNVHDPDIDGDGVHNVKDNDLDGDGIPNEQDADIDGDGIINSQDNDVDSDNIANHEDDDIDGDGIPNHQDNIKHESEPPKHVDLDGDGIPNEQDEDIDGDVIPNEQDEDMDGDGILNIHDDDIDGDVIPNEKDLDMDGDGVLNIHDNDLDGDGIDNVVDDDIDGDGVKNVHDADIDGDGVHNIHDNDLDGDGIPNDQDDDIDGDGIPNHEDNDIDGDGTANHEDDDIDGDGILNHEEADRHASEGEIKRVDVDGDGIPNEEDDDIDGDVIPNEEDEDMDGDGILNIHDSDMDGDVIPNEKDPDMDGDGILNIHDTDLDGDGILNVDDHDIDGDGVLNIDDHDIDGDGVHNIHDNDLDGDGIPNDRDDDIDGDGIPNSKDKDIDGDGIPNDKDDDIDGDGISNHADNDIDGDGIPNHQDHDGYESEGLPQLVDLDGDGIPNEQDSDIDGDVIPNEEDEDMDGDGILNIHDDDIDGDVIPNENDPDMDGDGVLNIHDTDLDGDGVANIIDDDLDGDGIPNIIDHDIDGDGVHNINDSDLDGDGILNIDDADLDGDGVPNVVDTDLDGDGVLNVADADLDGDGLLNVADTDIDGDGVHNINDNDLDGDGIPNEQDDDIDGDGTANSEDDDIDGDGIPNTEDDLINIYEFEEGEIPENRRSRNHIYELLQLDDQSNAREKAVDNVAEIILRDIKRIYENAIKPLEALYKYRDLSNRHFGDPEIFSKPLVLFMGPYSGGKSSIINYLTDNEYTSSSLRTGAEPSPAYFNILMWGNETEVLDGTQLAADYTFSGLQKFGQGLEDRLRGLKMPNKLLEKVNIVEIPGILEVRKQVSRLFPFNDACQWFIDRADIIFLVYDPAKLDVGPETEAILDQLKGREYQTRIILNKADTVKPEELLRVQSALIWNISPLMSSAQPPLVYTASLWSHPYQEGSPARLLLAQERAFLRDLRTAVDKRIENKIASARRFAVRVRNHAKMVDCYLNTFYNHKTLFGNRKRIADNIIENPQNYHIYEGLSTLTNISRYDLPDPEVYRDFFRLNPLYEFKKLAETCSYFRGCPITKLDVAISYDLPDLAGKYKKMVESALVKVEGKESFTSTTTASEQDDKNKKTKS*

>Woma_00007269

MFKYLLMLIFYVTMTQRIRGKTLFDYNINPQYDLVAEESTSYSNFFPSSEFYVAPVDRHKHHAKRGYNGIGNGKRGRGPPSVHGPYHHFIDSSEEIYGSAELHGYPPYPPYSHHPHGLPPGQAKKQINPHGHPHEPPRRFPPPPVYDKNFINPWFIPGPTPMPITVQPPPVTLPPLVYVAPKLTTRRTTTTTTTTPAPVRSSTTSDDQLIYDIDVRFGRD*

>Woma_00007268

MVKSLVLSLLLLLSIALFVSPAKASDQPARIVCYFSNWAVYRPGLGRYGIEDIPADLCTHLIYSFIGVDDKSWEVLVIDPELDEEQNGFRNFTQLRQTHPHLKLQIAVGGWAEGGSKYSQMVAVRERRLSFIRSIVNFMKKYEFDGFDLDWEYPGATDRGGTYGDKDKFLYYVQELRRAFEREGKGWEITMAVPVAKFRLQEGYHVPELCDLLDAIHAMTYDLRGNWAGFADTHSPLYKRKHDQYAYEKLNVNDGLALWEEMGCPANKLVVGIPLYGRTYTLSSSNKNYNMGTYINKEAGGGAPGPYTNASGFLAYYEICTEVKDKSNGWIVEWDEEGMVPYTYRDTQWVGYENEQSVQIKMDFIKMKGYAGAMTWAIDMDDFHGLCGRENGLMHIIHDNMKNYRVPEPTRETTPRPEWAKPPSTPPNPDEGSLVILETTTKKPKPVTTKRPTTSIATTNKPMKATKKPPTTSATTTTLRPVVSTTAITIPSREPTTEHQPAEPMESINIDCAHRDFVAHPDCRKYYRCVHGKPVEFQCKEGLAFHTVSNVCDWIENSDRYYCTRLKDKQMGNNA*

>Woma_00007346

MIKEKFYLQFAILLTTATYIGSVSTPCNLRETSNGGVCVCTIDYCDYLEDPTPLEETEFSLVSSSKAGLRFEITKGLFNLFKKCYVFDYDQRIEFQAKSETIQTKERYFPPRTVRLEINRDVKYRNIVGFGGALTGAVSYLLQKLPQELQDHLYKSFYSSKGIGWNLLRMSIGGSDFDLEPWAYNEQPENDAALTNFTKLDPRDEKKAEQIQRLKSVSKLNNLKIKGAAWSPPKWMKSNNAWTGFSHLKKEYYQTWADYHLKWLEVMENNDLPVWAISTGNEPMNGLFFMYFVRFMSLGWSPFSQAQWLSEHLGPTIRNSKYKDLIIFGNDDQRYTFPKWFQVMKFKHSKYLDYIDALGVHWYWDEIFKPSLIDDTLEQMPDKMLLVTESCIGDKPWHKSAPVLGSWERGEKYARAFLQNLQHGCNGWIDWNLLLDQKGGPNYVDNTVDAPVIVDTANLHEILKQPMFYTMGHFSKFIPENSIRIDAVRSNVNIDSVAYLRSDGTISTVLFNSGNANVEVNLVDSVRGSIVLHLPPRSIHTLLYR*

>Woma_00007256

MLISLQYPKCIIIFLFLNFHLNVDSMELSNFTPKIKIIYDNLITAYDALRNKIMEAERMSSLGGNLWLSSAEEKANSILMNAKKEEITEGFKDPTKYPPAMHFFQGKQYLRQSEVFRIIQKMPKGAFLHGHNKGMVSSKWVIGNLTNLYNLYTCRDVSGLLIFTYDQSKCHSEVQNVCLERVNAEDKRLYEKQLEKHINMYSMHPESLMTDRRKIWKRFEDIFQTIDQFYKYQPAFCNYHKRLLEELCEDNIIYAEIRASLSPLYGDNNKTFNSLEVATELEKIVESFKAKHPDFVGLKIIYAKRNKATVDEMTQRITTFKQLHNAKPNFIIGFDLIGNEDSGDPLHKFANELTDLPPTANFFFHAGETNWYGRTDWNMMDALLLNTKRIGHAFALPKHPQLWSTIKKRNIAIEVNPLSNQVLGYVWDLRNHPASFLIAENFPIVISADNPGIWNAKGLSYDFYYAFMALAPAEADLKFLKQLALNSIKYSILTSEERRKINRIFQKKWEEFIYNIINMKF*

>Woma_00007257

MHSSHTCLIIITLVTLIKASSSRQSNYLPYQDAREAVIQAEDLLSTGGRLHLNSRESKVDDILLKYKYDELARGFREPDKNAAGMHFFKAKPLIDNSKVFKFLQKMPKGAVLHTHTSATVSSSWVVRNIFYMPGLMRCTTKEGVSILTFRKLPQKHKCVTQYVSVSDERKKSLSSRDYDRALEKFINLYTPMPELEYASINSVWTKFQNMFSTIGDSLLYIPAYRSYHWQMLEELYNDNVMYAEVRLNFYELYDISGRIFPVERGVTELINVVEKFKLQHPNFLGIKMIYSPHRNAESGVIRKHFESFKKLHASYPNYIIGFDLVGQEDKGKPLYNFVNALGDRPKTSKFFFHAGETNWYGASTDFNLLDAILLNTTRIGHGYALMKHPVLWNAVKTRDIAVEVSPISNQVLHLVWDLRNHPGTFFISQNIPIVICNDDPGFWNAKGLSYDFYYAIMSLAPNNAGLKTLKQLVWNSIKYSMLQPGEKQRAYALLQIKWDHFIDDVLQGVVI*

>Woma_00007315

MLKLLLTILIIFAIGGKHVAVVGQYLNNCQMSGYGMNSKKELTGPLLMEAEHELRTALMKIAEEDEGPFYRVGIIFGGSEQLVAGSLKTVAAELVDFNNIKHNCEVDMWTRPWLTDGTEVTIRCECMRTVFKHIDY*

>Woma_00007314

MKYVSIVCLLALVTLAYAHPSCPGCVNQLQGDDLKKSEETLNKSLSKLAAGDGPVYKLVKINTASTQVVSGSKDVINADIKDENDKIKTCDITIWSQPWLENGIEVTFNCPGEEKVVKKHSA*

>Woma_00007313

MNKFLIFLYLALVFISTASFGKPIEEQNAGAIFKVRGGIRPLLDAGLKDAEEALQLSLNKLAAGDDFKLKLIKIHSASKQVVSGSKYKIKADFLDADEKTKTCDVTIWSQPWLENGIEVTFECEGQDKIVKKHSA*

>Woma_00007278

MSKQLVAIFVVLALFAWAEASNEVETVFKDNEIIPDVISDAPMEFLKVSYDDDLKVDGGNELTPTQVKLQPNVEWQAEENEFYTLIMTDPDAPSRADPKFREFRHWLVANIPGNNIDKGDVIAAYIGSGPPKGTGLHRYIFLVYKQPGKLELDEVRVGNNSRKDRPNFKAAKFAEKYNLGAPIAGNFYQAQYDDYVPILHQQLSEEN*

>Woma_00007230

MWLKNFTYAQLLVALLLVQYNVMLTSAAVNIAQLKSKQQQTLTQESTAATSTQHQQQQQHQSPQQHSLHVRTQTQHHRNIPSQHQSVQPAIAAHPSNNDNSDNAIHDATQALSSSSGSSLSYAPPATQYVVNDKETAAMQEVLKHFQADDDSYQILNEFKREAQANTNHGQTVAAPPPPPILSSPSTANNVPYPWNFYDRPYVSAPAISGVLANMFNPFETFTSPYPVTPRFLPIMTYPQPVYVPYPLLMPPEMFYPSFSPMAPHITDDYEDSMSRGAATSRRPASNQHLTSYSRNSPIYYVRLPPTPYMFLPSLGLGTPNAGIPGYPTMFPYQSLPPFPSYSSIFNVPINFLANGKPSNIYQMSGAPPTNEMQNHLQFANIPSSFNTRPPPPPPNHYRPPALATNSYFSQQSPSTSSSNYGASSSSSSSPALNLPAPPSSSQDSKLTALKRPYFFNGRPEEIYVLPNNFNALYSSAENSYY*

>Woma_00007231

MFSLKLIVLIAILAILLNLDTAEAVAKKQRRNIKATKKSNQNGKLKPTLKQVPASKTNQRNGKMNFYNSHLWPTVNTFYRPATTSSSSYMRSPLSTSSMFYPMHWGNSYINSSPLMPSSTLRRADHEFSASSPVSSSSTMGNSPIPTLYDMSAFTGNAGLVENPFGSSNQNLWSMGAETITPPTPKSKYNLFNGFNYNDDGGLFSGFLNHFL*

>Woma_00007236

MHKILGALLLLALAVTNAQRVAPGVPPQHYQQPPQQYQQQPPPQQYQQHPQQHLPPGVNPQQYQQQQMQQQQMQQQQQQQQQMQYQQVPVQQQQPVQQVPQGHGGGHHAPHGHHGQQQVLNAANIQHEREHIQEHMQVPIDTSKMSEAELQFHYFKMHDSDNNNKLDGCELIKSLIHWHEQGSKDAHAGNGQQPTVEEKIFTDEELVALIDPILQMDDTSKDGYIDYPEFMRAQQKSQQNQQQQAQKIDGEEGEKKENSGTGEKKSGTTYTDENLADVIDYVLNTMDLDKDGYVDYTEYRKSEENSTKGNKQ*

>Woma_00007526

MKLKFIFIIFYGCFVLALPFAKPIEAYNEERSKDKTSSLHLTKNLKRFQRQNFKEMPNSDKMAYSDADYAVDSNRNEQEEENEKWLQQFQETSNLFANLKHNERTAVSLFANDNDGDGDDYDQEKGESEHASVDKDDAPTNSGEKSEERSVVSRKFLQLPKISGTGEKITLSLRLPKGRNSNQQPNVDGNFLKSKYFIGPEQYAENCNTDISGCQQCQQQQQTCYSKNRNRTEGMKSFESGDNNFVLPKCNKSLKINNSAGYDKEKIARDEDDDDGNWALLKFSLANEQNNNKWSPKPLNDQLDYTKNTLSHEADYKLSKIEQDSSADKISNSLWKWGIFKRSRSFNPMHVIKLKTSERLMKRGLSASLPSFKLEYQVQNFGNQPPYNENEFKSRQRKAIDARYDRYKREKETSMVNGLVEKSLDAAKKSLSSTKSENDKVSVVKDNNNSNNNDNNNNNNQAVDLQSENIPQSLELNNAQELSKVLEPSKEEMSLEKNIKAFEDNIDTFGVRSRRRKRSISSSSINALETQGIKELQKENRALKLQGPLKALNTSPTQTKLTENEKTSKAFEHQSSSKKLLQKSVKTKLSTIKQNKNLRKKSYREILKSYQLRKPSFLLKLPLKRQNTFKASSKINLSFNSHQSATKRSIQSLKTKSNNNTHNDGSTLKENMKKAFASLAKLSKKEQNNTKFFENLSENKSFIGETAKENSFKKRLQKKKFNASFVKIVSKNKDLLWKNYLKKAQENNNESAKKIRRKRAIDSYYNENLMQHTQAKESPINKRFSTYLKGANKTQFNANKAKTDFGLYTNDESLNKFSKFNYKKYKDKAHRTKIAPMYESSLPKPMLSLHDYYGNPLKNRKDHLKNHKKSQKISKMYTETLKDFKNSEPDLFRDQHNNNKPQSVEVMKNRYLSDLKNFNQNYKFNEYPIEWSMTPLIRNPKDNNTISQDDSQALKLRNEILNCQAFSTLEDFLKSLSQNTTPPALTNNTLGTPNITTTHVPPTETAFTFSSTNTTTEQPEEYQVPTTCITILATDEATETCTEYVMDDSTEILPESNEEEEVYPSYDEEATEAKDLKLNISINAKVHGNPLGKDFYGNGSIEMMPGFVQGKKRRDLLLQLHKKLPEKSLHKRKCAQMPTVKPDFIEDSEDIGNLASGKLSSQMVENIFRRVQNEPTLQDLWPSLKSNERTTEKSTGFYALRTMGSEAEVRHAEELFSESIAAINSIIEGKLNSQACVTLRPDLQAFYNLILRTRQEQELFRAKRDMARKIKKSDFSQDIRLLNANEIQEKTSTVKKLLKEYDALPEHEQKQMLAVKEELLKNLSEFQQLNAVEKRRHRDLATRLQRISDVSVENVMKTLPNSYTPEFIKLLKAANFYRAIQS*

>Woma_00007612

MLKTVTSFLLCSVFLASTLAAVVIDCQRPPQLVDPVRCCSDGGRDDVTENCAQRMGITGQPTDAPPSVETATCLADCILSESKYMTKPETLDLNNIRSDLHTKFANDTVYAETMYEAFRKCQPTAHKKIKAFKQIQMSSSDAAATAFKGGCSPFAGMVLGCTYMEYFKNCPAHRWTDNAECALAKQFVVQCSLGA*

>Woma_00007657

MCSRNIKISVVLFLVLIPLFTALPHNHNLSKRSNFFDLECKGIFNKTMFFRLDRICEDCYQLFRETSIHRLCKQKCFTHETFGDCLKVLLIPEEEVTQLQYFLKVINGSPLSSLDFTSLF*

>Woma_00007678

MWKSLTPSPTMVASSSSVLNSLKLISKSSLSLICLFLLLMDNTEAVGFGRCPKYPSMPKFNMSRVLGQWYEVERSFYLPEIASGCTTLEFQPLHQDEVSRFNNFKLEVAIKTINRITGNPSVNLGYATPESRKSSIMDFKFNTRFPDVIARLLPGSGKYQVLYTDYDNFAILWSCGSLGSLGYSDQIWVFGRNRDFPVEVRTKIYDALKKLGLEPDRLVLSKNKNCPKTL*

>Woma_00007670

MAKALFVFIVLTALMHNTFAADCKKKPPRVNLENCCPIPELFTEENTEKCKEFLMPPSPSSTFESMSIENPPTSKSIKPSPTPAVQRSGAPLHLCFLNCVFNDTGIFQETGKINESNLSALVKEALKDTPEFIPEIEKSFKTCSNRIEKFHAKIQEEMENRSTKDAKMEANNRMYQQPAPSSICSPKVSHLMTCVFTETFVNCPAKYWTKSKECNELREHMKNCSPNQVNSMEDSSSRTSEETM*

>Woma_00007609

MKLFVALTVLGLLSASLAFETPQQVQAAITGHVETKIFNLLSMPGIGRAGGGIKCYDKYLPLITKATENSKTDTEACIERAASRRNDELNKVKQERENLNLQVRNVENTLTSCLNKTDDLEYLKCLRENSAILQSTISEVGKSSTLLADNLSASYKNIDATEDNCIKNVIAKSKSESDELLAALQNCLMNGLDSGSDGDSEGGSEGGSEGGSEGGSDGGSDAGSDNGSDGNSDAGSDGNSNGDSDNSPNNGNDDDSDSDNNGDSDSDNNGESDNGNDGDSDSGNDSGVIPDERQNPEEDIFP*

>Woma_00007610

MASLRSCSILFTEIIIIFVLIEATCSLRCRSDDGPSDAELKRVVRSCMRKFGDAVYAQNSQQQQQIQQQGRQRSRFGNREYFGYAGGYDDSDDMDDVDNDDDQQQYNYNNNNRNYNRQQNGYNNNNNNMNNNNDNERSRNGGGRSYSSYDSYGSRYSGSNNRTNYGNGNGNNNNNNWSYNQRNSPNNRNNANNYNNNNSNNSNRSKNNNNNNNNNNQNNSSSNDAACITHCFFSEMHMLNNNNYPDRHKVMYILTKDIRDRELRNFYTDTIQECFQYLETQRRRDKCQFSRDLINCMTEYAKGNCDDWNEFNMMFN*

>Woma_00007528

MPKTNSILSLTFMAILIVCLMTKPSTARPWDLYDDVSAFFDTMSLDDVPGAKEADSESKDFCLMPARKGVCRALIPRWSYDAQKKECVEFKFGGCDGNKNNFPTYKACMETCQGM*

>Woma_00007574

MKFLIFITIICTLTAINRGAVMQGAFKDPSHPGKCVYGDLILSPGEIGQKPNGCARIMCGKDSWTTIHTCGAILPPEGYKLGEPIKPQADYPDCCERAYVPII*

>Woma_00007556

MHFMCIKLNLLILFNFIILITAQNCHDEPVKLDEEFVGCCRGRPKYTSEPCIDELLENNTLSSECIVNCIYRQFELYDGETIDMASVKGFLETEIPHEEFGLMYLNAFEKCSKLDKQAIAEKFDFFEIPNEHGCDEYPPLVDVCVWYHTVANCPTSYATDSESCSNKMQWVNECLFES*

>Woma_00007598

MLSCQHIMLFLVTLVIVLLAATQQSWAQPVAEPLADPLAEAARTEVPKLLLIIDDVIPDTVEKDTKKVIRVSPLDLPTIREDPNVLNMDSSLYKIQSIKPYNGKRKYVNMHRTKPQTKDNLSVDSTTDTPIIRGLSVNTAEKFVKPKHQPKKKNLHFLYNQKDLKGNLLSKTMRVHIAPGAYPVYYVISKTIGRFGKFPLKTFDSPQAFMKYLRKTKLEDLPAL*

>Woma_00007597

MKSFLLPIIFSCLCLTTMAMPLKDSNQEAADSPISTIVIPYEPYTKSQLMNSQGKLQGVYVPLNMEIVPKVSLKEGEIAMASPKGLVLNVAEGAYPVYYMPGNANGKFQDRKIYLLE*

>Woma_00007590

MNKNFFALAALCSLSALTTGQFVLKGACPDDIKVVEDFSVEKYLGKWYELAKYPVYFEMYGKCITAEYTLNENGLVNVKNSQINERTNAYSDILGNATLVSSGKLLVKFPVSPALNVSSNYWVLDTDYDNYSVVYSCTPIAENGHATIAWILTRNPFPGDEVVEQALKVLKDNNISLLPLQMTNQISCN*

>Woma_00007592

MHRQAVLTSLFIIQLLFLVTNAGKCSDDQQHSVSTFYLIKSLHRSLQERLYVDNVLTLQECIELAIKYKGLALNYAAKNRFRRVHKDIFIESKISRDHYWNQPQLYFNCHILRCPENKTLKTLVNDYNFNYYSMYQPPVDSSNYICLPEVGLFVLQTIPEAFENASLNCRNSKSFCGSLPHVASRMRTNALTQLLLAHNVHQTKTHSKARVYLAFVGLYYNRSITYTFDMLNMNRESLKCFPYRVWEPGNPKRSSQFVNTSCVALTTQRTWRTVNCKRKLPYICEILTTCGEEKEDYLE*

>Woma_00007669

MKTFEIYLLGILIILAGFTTSNAEVDCHKPPPHLIFESCCEIADVITEEIETKCETKGISYPHHCFMSCVFNESGILVDGEFQEDNMNIYLNQVYDDSEIVDLVGEAFRKCNEKRESIAIRQPKNSHGSLHCHRDHGQMLYSCIHMHAFKNCPDSVWANTDYCNAAREYYRECDPPM*

>Woma_00007668

MKTFEIHLLGTLTILAGLTTSNAEVDCHQPPPYIDLEKCCQISDVITEEIKTKCETESISNMPAMGEEGGPHPHFQCFISCILNETGILVDGVFQEDNMNAYLNQVYDDSEIADFIGEQFRKCNEKRESIATRQPKNIHATLHCPRYYGDMLIGCAYIHAFKHCPDSAWTNTDECNAVREHMRECKPSHHPNNDEDEDDLDEPFEEDVDVETEK*

>Woma_00007667

MKTFELYLLGILTILAGLSTPNAEVDCHKPSPSPHLDIKKCCQISDVITEELNIKCETESITKMPAMADNGVFRDDFGPPGSENQGPSGHQREPHRDGPYLHCYISCVLNETGILDDGEFQEDNMNAYLNQVYDDAEVVDFIGEQFRKCNEERESIAIRQPKNIHRPLHCPHNHGDMLFACIHIHTFKHCPDSVWSNTDECNAAREYLRECEPAHPPI*

>Woma_00007666

MKTFVICLPGILTILARLTTSNAEVDCHKPPPHIDFGNCCQINHIITEEVKSTCEGKIHNMHTMGEVGEYRDHFGPPGGEHQGPPGHHKGPSEHHRGPRHHGLYYHCFMSCVLNETGIVLDGEFQEDNMNSYLNQVYDDSEIVDFIGEQLRKCNEKRESISIRKPKNKHGPLHCPNNHGHMLFACNHIHTFKNCPDSVWSNTDECNAAREYFRECKPPHRPPKKEDEEEIEEDIDEGLDEEDVEVEAEEEI*

>Woma_00007665

MLFYIINILIITLTTPAWGATIQYSSMPNPDCNRPNYMPDEKCCLIPSIYTEKLMEKCGELIEGSSTNMDLSNSFFENCFINCVINETKIMADDGELSEENMSNYVNEVFADTPDMIPIVEESFRECFGVTNISRQYIQPNDNCPMQITTIMDCLYTRIFMFCADSVWTNTETCNDARAYSDKCMPKFLYMKIE*

>Woma_00007664

MKTFVIYLLGILTILAGLTTSNAEVDCNKPPPHLIFESCCEIADVITEEIETKCDTKGISYPHHCFMPCVFNESGILVDGEFQEDNMNTYLNQVYDDSEIVDLFGEAFRKCNEKRESIAIRQSKNRHGHLHCHHNNGQMLYSCIHMHAFKNCPDSVWANSNYCNEAREYYKECDPPME*

>Woma_00007576

MQIFFLLTFIFCWIYYSLAAASINFYKDPAHPGKCVLRDLVLAPGDIYNNPNKCEQITCYERSQASIFSCGVHQPPNGCVAKKIQPKAPYPHCCTLETMCNNILTGENAVAPIIL*

>Woma_00007575

MKFVTLLCVFVLITIVKARPDVSIGRFISPEHPNKCVVDQMILEAGSVAKHPAKCAKITCHANSKASIVTCPAMIPPVDCEVKEPMDPEANYPQCCRRKIVCKDGKVFNS*

>Woma_00007579

MFKLLCLLLLSALMAELAYARPQPQNDVQQPNYLPCTDHDYLAYPGSEYYGQSHPQTYAQHYQQSYPSPLYTPPGTYFDQRRGILRPFPGGGVPVLVDYHKRCSNDFVGLKAHPEEHQYYYVCKPDCVIFGKCQNLQIFNSTNGQCVQHAPPEYMPTCTSVGRFPLFSDCHLYYKCEEDLKAKIFSCPSHMIFSPVSQKCISGNKCTPTQISPESSYLPEYCAYKFPPCYENGVFRSPSDCSLYYSCELQNNGIYLQTRLRCPSGQFYDLKTSNCLPAEQVPCDCIQVAELVYPQTNGLPAVYPAQFDLDSLEEKIYPIEKTEAENSVNKSEEDDEVASDIPEPTTALVHFKAEPRIGDIQLSALLQEHPEMSKEITISSKEATSTEGSGLTTLELGTTPTDEDVTNQFVTTTLSSATEIQRIGEPDTLRIAKAIAANTGLRLLQFDLEKSTIVPEISTSIPEEPTTTIFSEPSAIPDIMWSSEIQTTTDSASHTIPDEVFSSTSFSNIMPKEISTTSESITIPNETNKTEVVITAVSQPLHCVDTNEINNDDDNFGTKDTLTAQSLAKSCKQTFTIPSQDTTKSPDTDQQQTKAITHVSEFTRGSRSTLVKQKITPTTFTTIHLKAPANLTTHIANEIKQIEEEEELFSPQTIENDTNNLKQNTFQNDNDDDGGKIKSNIFSEEEFLPVHSKTTAIEKQLEQTTDFDYITSNPEEDSSFNIKMDYDYTTSISEIELNTRPSSQLSPTEGIPRKTDSVEFVENLIKVPKAKDFLHSIKVDNKVEAITATVLKHRQTNLAESATTLETTTAIAQENETFLTLGEDLLLDLKDVAKNEVTVSILPTDSTSPLSHTLSNTTNENIPHLEIIAERPLDVHFKICPTNCDKPHQHNFSPAGNRAVFVRWFANKSNQTLGFEAANEVNTHGDEEKDY*

>Woma_00007560

MSILCLLSLLFNTVLDVALSNVSGILQAEFGGDCTEKWVIWIMQMYFGVEKGPFYLYKTFNYDYDYNYNYDYDYDYDYDYDYDYDYDYDYDYDYDYDYDYDYDYDYDYDYDYDYDYDYDYDYDYDYDYDYDYDYDYDYDYDYDYDYDYDYDYDYDYDYDYDYDYDYDYDYDYDYDYDYDYDYDYDYDYDYDYDYDYDYDYDYDYDYDYDYDYDYDYDYDYDYDYDYDYDYDYDYDYDYDYDYDYDYDYDYDYDYDYDYDYDYDYDYDYDYDYDYDYDYDYDYDYDYDYDYDYDYDYDYDYDYDYDYDYDYDYDYDYDYDYDYDYDYDYDYDYDYDYDYDYDYDYDYDYDYDYDYDYDYDYDYDYDYDYDYDYDYDYDYDYDYDYDYDYDYDYDYDYDYDYDYDYDYDYDYDYDYDYDYDYDYDYDYDYDYDYDYDYDYDYDYDYDYDYDYDYDYDYDYDYDYDYDYDYDYDYDYDYDYDYDYDYDYDYDYDYDYDYDYDYDYDYDYDYDYDYHYDYDYDYDYDYDYHYDYDYDYDYDYDYDYSYDYDY*

>Woma_00007562

MANILKLLAYFTLLFTLCGADSYYDQTKSIYLSETSAQQLLQEINDLYWKSATAYRKFADDTLSPEILMREKLRPFYQTMQKFDWQNFNNPLIRRQFEVILRGAKYPPLDYAFKRATHTLKNMSRRKYVCEKNMPHKCQMAFVHQIKTIFTNSDDLELIKWYWKEWRDNMPQEIKDAFHYYVAYYQNMSTPEMPASAIWYDEYEDPNFIVELEDLMNTILPFYREIHAHLRHVLKVRYGNNVIPPSGLIPHHLMEQAMYQAWKKDSVLRNPFPQRKLPNLQVEIDGLGIFPYDIVNMSARYFSVLGFENMTDEFLRDHFVEMEIGEGGPDCKSRIFTYGEIEMQYCPKVYYKKILQTHGDITQIQYALMKNTMRVGLNQEACPGFANALGEAIILSISTPQHLQKCLGLLKDYDYDDILNLNRLYRLAVHTILTIPNYFVHEKLWVDIIDRKIEPSQYNCHYWNLMEKYMGVQRPVDTKPGAYDMPFKFYEGIVDHYRSTKKLFGEFLGYQIYRGVCLKTGEYQRGNAYKILHNCDFDNSKHAGLILKEMMQAGSTKSWRELIKPLTIQKLSKLSATPLLEYFEPLHTWITEDNLEKNLHVGWLTPDQCRHVNAANATAVFIEEHY*

>Woma_00007564

MAKRLIFSVLLVASSSWCYGKPTFGKEHVHIRIHLPESGGDHGGHHIEHHDHGHGHDDIIGPLHGGLLGGGGHGGGHGGGHGGGHGGHAEEHTVIIQDAGGHGHGGGFGGGHGGGFGGGHGGGHGGGHETVIIQDAGGHGHGGGGGHGGFGGGGHGGFGGGGHGGGHETVIVQDAGHGHGGGFGGSGGYSAGGHGGGHETVVIADAGGHGGGHGHGGGHGGHGGGHDTVVIADAGGHGGHGHGGGYGGGGHGGHGSAHETVVIADAGGHGGHGHGGGYGGGSHGGHGGGHETIVIADAGGHGGHGHGGGYGGGHGGHGGGATEHTVIIQDAGGHGGHGGFGGGHGHGGGHGHGGGAAHVNTYAVIQESSSGGHGGFSSGGGHGGYSYGGSSGHGGYSSGGYSSSHSAANIAAIAATAGDSSHGGGYSSGGGYSAGGHDDAAKIAAIAAASSSSSSGHGGYSSGGGYSSGGHDDSAKIAALAAAHSFSGHGGYSAGGHDDSASIAAIAAAHGSAGHGGYSSVGGGYSSGGHDDSAKIAAIAAAHGSAGGASGGGGGYSYGGSAGGGHDDSLKLAALAATSSGSGGHGGSAGGYSYSPSIGSGWSSGGGLSSYSSSSGGWSTSGSGGGGW*

>Woma_00007734

MKMFRFSIILLILCSYLRLLLGNPAEHCAFLEDNLLHLYCRGVYPQEVHLKYHDEWIRIGEPFLIYRSKPKTSEYLFVFRKTELHKCKIITVLSAIKFKCILAKSETDEPIILRLRNATGMCYSMNLFLTDDLVRICLHQNKRKEHIKPSTQNAFLYDLEADPDNAVKCHAFIKEIFFVLALLVYMYPTKYWTWTINSGFH*

>Woma_00007755

MTDFYFSRIVLNRSQMYFHLSLLTVLLACSTQAIQIRPSYGPPQRQPHREYGVPQQIPFREYGPPGLKYGPPKLNFIGGGSSSGLHEQIKTYYGAPKPFYGPPHKPHQQYGTPPQQYGPPPQKPAATYGPPPPKPAATYGPPPPQKLQHKPAPAYGPPKPQYGPPSLPPPPQSLPLFQPPPQFKPQHQPSTSYGPPPSGPLNIPPKQIYGPPPPNYGPPPLPNFNGGAPETTIIVTGGQNSQSQSHHQHQQGHQQQQQQQVQIQIDASGHTHSVAGSQAPFHTACEGWKPIPAPAGAYIENNHIDTQAGYSQVSQGSAHASAASSFSNTQYSNGISSGVGASAGGSNILISGNSGASGNSIIDGLTDQQLVAIALQASGDNSAPQQDNLPSTAPANDFNQHSLNSIEAETLQLSLGSSDDSYSRPPSDSYAPGSKYSHKFGHQSSSQSSFGITGPGGNFGPPPPPNGKYGPPPPNGKFGLPPPPPNGNYGPPPPPNGNYGPPPPPNGNYGPPPPSSNGNFGPPPSSLGAAAFVHHHAQASLGIQYGKQSGSIGHYASHGGTQPSKPVAFRPPVPQGLLETIGNTVQHLDKFGAKPPVQAPTYIPPAANEIPLTGPGNLNIEYGVPQQQQQQQHQVPLSIVEQQLPQQQPAQVFVQIEQQQHQQSHSLQQEFANAHAEYGVPPPPPSTPTVPEQYLPPPPPAQLQQQYLPPTGPQQQQFNSGASYSSSASATFDNTAYAASQYSHDIIHTQGLPLQQEQPRFHDCGHGPNLVGAGGFSYQQQQQQQQQQHQQQYSSSSGTSIAQSIPIGEQHTQFITGPADSYGPPPSGGNEIDHIGYASQKSQVTAFPDGTIPDGLPGLDGLNVLSTQKSEGIQLSSQQETHNTNTYQVQYSANGQESTNHQEILSADLLQTILNAVEKPQQQHQPLPHNHKAESRSDIDIHDDEPIEAEDENEPGVAAQETKHVEVKVQPDNEKSEESPADVKEIKPIVVKEENEAKH*

>Woma_00007754

MSSVIMLTLTILLVLSTASIITSAWAESEINDLNTIETTQKPLKNVSAVAADRKMQAETRQYPFNGFMMPLPPAQPIPPLPPSTTSAGCPLCDSSVYSYCSHKLIHDSCCCDFPGSTIYQKPLQCLYTDCSLLYAKSCYEHSLIKNCCCNNPY*

>Woma_00007750

MRISITIMGFIIGLIMLQLKAQQLENVAENLSFNKTNANVTNITTIQLKIKDEIVQKSKTNVKDEINTEIVTQTTTNKPFTTATTMNPVTSATPLNKETNYTVTATYAYPLTTTVSPTTSSTPSASMSAQLELNNTSTATTATTTTSATISATPTSISSSSNQYCAPSLCEFFNGTHTTIKPHIACKNSGNFGPACGSQPHLLKMSERRRNLILNLHNLARSRIATGQVEGYKSASHMPQLKWDHELEYLATLHVKRCKFEHDLCHNTPRYPYSGQNIGYFWKGANITSHSKRMKNFIINWYKEYVDANQSFIDSFQLHPENKLIGHFTAMVGDRVHHVGCGAIRFYESNLTKLLMTCNYDYNNFIDEPVYKTGPAASKCAYKISDKFPGLCDWKVPIYEYNESDDEVFENSSNVVLRV*

>Woma_00007684

MKFLLLLIVGLCGTIQAGVYTQRYFRDENFPGKCVVDGKVLRPGQSIKHPTMECAEVTCDNSIGMATIETCDPISALPPPLQSFKSCDLLHPRDCVWGDFKNTKAAFPKCCERHLTCTF*

>Woma_00007725

MWFLIMLTILLSHIHYARPTNHYSLKDDDVIKRRLDSPFHLREPDNLQMFIEQIHYNENAEEAFRKMQRKRSTLDDHIVITAQLLQQILDRSKCVRMYVKMAGLSDYLLTTAASMKRLKFPEKFEKDFKGGEPICPNYAHLELERDKYANLKSFKDDIIKSLHIEQEENLLKQELDIKDAAKVTATIYEISVKGLHSNPSAWKFHMLGSYYWRLTGQAKNAIDCARIAVILAPAEEKDIPLLSLGTILVRAEQWDDAETILNDAIKYGPSHGENYIVLATLQALKHDFKRAKQNFKLVEKLDVNMYNNSLKMRQYIECLEPLDTNATKLFGFVKYMLREIKEVSKLRQEISQYQSKIIQQQTPLASRYTDNDPQLKADLLRRFQYCSTRKATDGQEPVLFCDFYSDLQMQLESKQFDAELLDWQVNRYISHLFEKFPFEYKKQLETLYNNVKAVAPTKLGTTFTM*

>Woma_00007768

MKFLIILALLCFSYAEEHSTAAPQTGGRLPPQHIKEFVEQHIKQVKQQIPIIQQQIKEGQIHVEIVKPIAKEQNQVPEHIQEFIISQINQVLQHVTQQKQEKQLLVPLTTTPTVGIPAFVAEKIKKLQEYVQNIMQQVEKHIPEIINQQIAYKQKIKNKEPVPEVIIEKKPENVVQHIPQTETHHHHVKTRSTTDDVKVSEDVKKQQNVAIKPVVVPPVVQEIVQHVHQHVPQVVSQVQKNDVVIPAKVQALVQQIHQHIPQVIKQHMAHVNQQVPQVTTHKIHTRSIKDDVKVSENVKEEKDVVDIKPVNLVEHGHQHVPQAVSHHVVQAQKNDIENKQVVVPEPVHQHVYEHVAQGFYKKVPQNAGQHVTDTKIVVPAHVQGIVEQVHQHVPQVVNHISQKKTDQEIPVVPLHILDLVQQVHHHVPQVIQQHKDQIQKKIQPISQTIHTHVVMKPNPKVPSDIHSLVTRIHTHNPQIIAQHVDKHNTDKVLKEDSTIESSRVQEIQKVVVPLRTTEVQIPPKVQHIVEQVSQHIPQVITQQVAAAKKDDKA*

>Woma_00007769

MKLLTFLVILTFFAFVSCKIVSIPTANPPNRCAFSCTSSDITLCATNGQCIQEFQGECMMTAYNCEHPHKPFVIIEDWKCKQSGAAKCTQIELELY*

>Woma_00007748

MKMLSSKLAQILLVVILPLVFTQTNYCDKSLCGDNRKHVACNNNGAFQAACGENPKVIEMTPELRKQILDKHNELRSQVAQGFDKFKPAVRMATMEWSTELANLAEFNVKQCWMGKELSVTNIIDSQIQSWFDEYKVSTIDDINKYPSNTDGRVIGHFTVIVQDKSTHIGCAIVRHKNSNDFTEQFMTCNYAVANIIDRQVYKTGPDASQCKSGNNPNYKSLCSLEEQYNF*

>Woma_00007749

MFQIKLILIALISFGLIELSLSKTDYCKPNLCKNNKKHIACNNNGAYSKKCAKNARVLKLSKKLQQVLISKHNQLRNQIAQGKLKSYQPAKRMATMRWSTELAKLAEYNVKQCVIKHDQCRNTAKFHYAGQNAARKKWRGLKLGVAEVARAQIQSWYNEYQKCTMAIIKNLHSINKCGHFTAMVQEKNVAVGCAILRQTRNGRITQLMVCNYAYSNILRKPVYRAGKTASLCKTGANPRYKGLCSVKEIYDLRSTNF*

>Woma_00005140

MGKVVCALIAAVLVLSCTKLAFSKKVSLTSKDFQKEFLQTVNNAMRQTAKDYLDNFLKEIKKPAFVAENFDNELKFALESQRLEPKVVFIKQYLDMAEHKDTSRANMYGLRNVIFLKKLKDKFYALQEQSFEEIHQLRYFYLCKQFKVQQQATTPAYHNYYSRQSIMAPLQEEHMANMLNKLGLKDNHTVTVNVAKFGEDLYEKVKESGAEVIDDYLVIIRKLLIEVLEPESEEEVISHSEALRKILIEIDALLNTHDFYKKRQRLYDYLQNHLALDYEQIQNSNVSQHDLKEISEKLEAKGLDLVVAYLFSNFEFIDHLHDHWKQQLPLAPLSLYDTDSRPLKHIQQLYEEFKKDFENSTKYDAYLEAVKELYEKTQQENTDSTHIYEMLYSAAQNVGSSRATLMNSKCNELFNE*

>Woma_00005143

MKSVVIFYIISLISKISGYYEVFFSPYYKAYNYNYAQKRNIENSINKTVEPCENFYEYACGNYIYDNNHENYREITQYLDSKFNKKLLEVIRPEGLVEEFIKKVTNFFAQPKQTFEEKMQNYFVACSCERKGLLHSYNCRNLIMDYQINRNVDGFLELALKPILYEVSNILPEDEFIYAFLRLLDVADSQAVVQQLKLTQKNWLEISKNHTQAEEETQELSCVEFLAEYSYFNDYFKSLLGRKISDITKVTLIYPAYFKYFLDIEWSGKEVEQQLTFLMIQFFYYLALDGTTGYTPLACVKDMREKFDLASNYVYYQRVLRNEKRSYKLALKEMKNKILTTFEHYLQESSLNISSLEVLELKNKFFNITFNLGNLPYPMDFQTIDNFYLQLPNLDDRNYFRNHLLLLKHRFLTSLQPPTDLNIYLAEDMKPLLASSPYYVLEQNMIVIPLASLQYPLFHYDYTNLYQLSLMGFVMSHEVGHVFDNTILITPELTKCLENQQSTDDMDERIADIMAERVVYRTYEKYYADADNQMWQRLFYLNMAQFFCNKGDLEFRNHDADVVRLQQIVKNSPQFAEAFECALDTPMNPKEKCRLF*

>Woma_00005144

MLKLSIVFGLLASIIWRTTPTNCRQTNNQTQNINQLAALNIERYMDATARPCENFYQYACGNWYQQHIDEMDYGDVLGLMDYELNRKLEHLLRKTLTQQLAAGNRTEENLTAQYTTTLTLADAADGGDGSWGIGESSSLYDKVRTYYKSCKKVKPYNLKKYLQLVQPGRGVHWYVLAKQGNRKWLPEKFDWLETIARLRLYGLNGVLLKEQVLPRWDESSSMSIYLDKPDLAETLAMGEGVIIELLMDIGQTKRVANEIAREVNRFEFQLHKLQELEDEEGPKEMQLSYLQEYMPQIDWLSYLKELKGPDTDLATTVIIQNIPYMRSLVKLLNTVDSSTLCNYIMLKFLLYLKAQGPNEISRQECIASLRRVMPLAMSYIVGIHYYKDQEKNDLQVEKIFNHLKLTFHNTIQDNRLQLPQPIVQTQLQKIDTMKMRIGFAPRNLSTEYFDMYYERVTLDANNFYANQLSLLRLTVEHSHETLDPLSNPDLLADEAVISAEWLGSSSSPLYLKSRNIIIIPYGFLHMPIYHRNLKDIFKYSVLGFAMAHEMLHGFDSSGIDYDSLGNIMGPSEEISANQRFMQGLRCMQNELATGSRSLNEKLADYEGIRLAYNAYFTLNSSNDVNQHSAKAWRGKFLSQFTPQQVFFLNFAQFFCGKVHPKLTHSAYLDHALDELRVLQTLANFEDFGRVFNCEKRDKMQSRHRCRLW*

>Woma_00005146

MQYWCLNKLFILTIFISESLANSASRKNINKKLLEDMKAIMNEKADPCTDFYQYACGHWDYKLPYHREYKHIVGSLEYKINIKLKQILEQRMWQTQQETNDIYNKVRLYYESCEKQNSFNVTEYLEILKPSDRDEWPKEGEYWQPNTNWNIWLTLARLQTVGLNDFLLTMNMKRYNSTHFYIYLNKMENLNYSTYEQRILDDVDYMYDMEAYLIYKKVENFLHKLFLLNRKHQKNSNIFREMSTTELQRLVKNIDFKTYFSTLWGKKFFKDLPLKVANVDYLRDLGDLLNETENSILIHALKLRFLGFMEKEMPRSTTEHLNCVQHIRDLMPLALDYIYEREFYQPLKNNNNRLITKLFKNIKNTFFTLIAQHSKELSLEEYNFLKAKLKNMKLNIGNMPLLANQSFYEDYFYTWQVHRNLFYWNHINALQHFYTERFYLLQQPWFEPLHTYFPWPFATPAYQPTTNLLIIPYAYLQYPIFHPDFHQLFLYAELGNTLGHEMLHAFDTNGLQYDSLGNPSSVFMKSLPDKKSLMASLECFEKFKSFSLNEIIADINGFRLAYDVYFQRKPVHKFRGTSQFSNKQLFFIKFAQFFCSSYSYHDETHDSPSYRVRKVVTSHPEFEKIFKCQRNIYKREREKCNLW*

>Woma_00005147

MWLLIMQLLLLITLLLTNCDFAESKPLNFLINAKAPLILNEEAYWEQVNKTCLSHDCDEHVNLRHLQHIATHMNRAINPCENFHAYACSYWQQQHPKQKTAMDEGEQQLNDKYEAIFRDEQNTLLRNDAIMQKLKKYYGVCLLSESEEKASWLNYINTLRSMGYLQWSNNTVWLQTLRELNVFASSRFFISLQIQTSNHTGYALSVDPHCIMERVKLTQDIYNILRIYSNASFTQIETDFKNLELDLENILNTSCLNAIVDENNECDLSQWLTYEQLLNSNSSLDWKYLLADFALEPTDQININEFQNLEKIKNYLDQANQKTLFLYAIARFVNYLQTHSHNIVDKGSQSQNCMRHMRKQFPLAMNYLYEQVYYKAKRQQSDMVIDQVFSQLKKQFAVILDSNEMQFSTESLQYLKKKLLHVQLNVGNLPKNASLEFYQNFIKDLNVSDNFYENHLLALKHYFLKKRLLASPSKFEDFWYTFNLHMPHFSDGMDSTPYYFGITNYIILPFAYLQLPLYDHRFWPSLLYGDLGNTLGHELIHAFDSKFLQYDFAGNYNDKESLNIINNPNFQQHISCLSKEPTTKFLTERIADISGTHLALKTFMRDPLFLKHNGKLFFLQFAQFFCGSPTPEAFALDNKTHDLDSLRLNYTLAHMPEFAEVFQCPLGSPMNPEKKCKLW*

>Woma_00005162

MHFIKISFILFITMVLFIALCKAGNKGSKMVDEPVKPANNALKNKSPKPTGVRPLAGAGTGNSYVSNKPTNRPRSPKKD*

>Woma_00005163

MHFIKISFILFITMALYIILCEAGNKGSKFLGDVTKQTANAFKDKGPKVTTFVAGAAAVNSTGNLYVNTKPTSRPSSPKKG*

>Woma_00005018

MKLLLVSVVLCLSLHSTLASKTDAKQTGRLISVSPAVPAGPAYHSPPIYYPPHHSHCHCPPGPPGPPGPPGPPGPKGDTGYRGRPGRPGYRGDRGPPGHGHHGHGYPVPPVLVQPAPVYGGGSHHHHSPIYSHPPANGPIIVVPGGSSSGGGGGGGGGGGGGGSGTSSSTTRTTTRRPNVNNQNNRNRNFVYQARFSPEQYQEVLEQLQNYGDQDQSAVLELNNDLDGQNESVSDADSQDYSDNDNEQSTDAEYGEEGEAGEGYQDTLARFYQAIKSTHTVKRKHHDKKR*

>Woma_00005016

MKLHIAIVVLCISLNCGLARKSDDTERSFSLTNSDRLGLTGPVGSPAYGYDQSHDHGHGYYPMPPEVVQPAYAYGRSSGGDGGEEKEQQYHSRDGSQRQYYGGYHGYSHYPHYPHYPYYPHYPHYPHYPHYPYYPQYPPPVFPSAPIIIGGDGIGGNFNISGIQAPYPFYPFYQAAQSPIIVAGGGNKAGGGGGGGGGGGGGVDNGSNSSAGGGGGGGGGGGGGGRSIRGGSGNNANTNSTTTKTPAMGCQIRFEDDITQQEGQKGPEEQKVHEKQKLQEVQQLQEEILEQKQNETIEQFNPAEDIVAEVPIEFVASDMISEKRTEDNQS*

>Woma_00005017

MKLHIVLVAICFSLCVAESAKLQKNTPGRLISMARSAPTAPSDLVYYPPPSYHHCHCPPGTPGLPGPPGEKGEPGHPGIPERPGPPGDYDRDHGHCHGYSQYPVYPYPPSPYPYPPVYPYYPQYPHYPQYPPYPYYPYYPYYPHYPDNGTNTTKKPKDAPIEEQTFGFTRTITDEDYLQSLAKPMYEDDLEESFNDDGQEVNNADNIKESYNDNTINENFSVEQSPSARFPLIIRAIPVRRPKAN*

>Woma_00005015

MKLHIAIVVLCISLNCAVARKSDDTERSFSLTNSDRPGRTGPVGSPAYGYDQSHDNGHGYYPMPPEVVQPAYAYGRSSGGDGGEEKEQQYHSRDGSQRQHYGGYHGYSHYPHYPHYPHYPHYPHYPHYPHYPHYPYYSHYGRGRTGRGGSSNSANTVSITTKTPAMGRQLSFEDDITQQEGQKGPELQKQLQEEIVDQKQNETIEQFIPAEEIVTEVPFDLVAHDIILREAPAESIITEVPIELVTSDMISEKRTEDNQS*

>Woma_00005050

MKPNFLFLYTIILFASSPCSGAGYKETHVDYFLKRSGIEKTPFSFKDAHNGRMLVTNLDNFRGLMVLIRQILLKRANLQWQNLLKYNWKMGKIQKPRPLWEAFPCSLNNTRSKQPPKSVHELRPGDIDVVAAIGDSLSAGNGIISKDILHIGTEYRGLPFSGGGLEDWRTVLTLPNILKVFNPNLYGYATDEVLAVHRSSHLNIAEPMIMSRDLVYQAEILIARLKRDPNVDFQNHWKLLTIFVGNNDICSDMCHHDNLVEFLRKHERDLRNAFSLLRDNVPRLVINLVAVPNMVTTLYAMEGLPLSCQAIHRVGCHCIFSERYNNKIVKRAYNYITRWQAIDQYVAELPEFQTDDFIVLYQPFTANVSMPTLANGKTDLRYFASDCFHFSQLGHAVMANALWNNMLQPKGRKQSVLLDPFVQFECPTEDRPFIVTGKNTRERK*

>Woma_00005051

MAYFKVFSLALIFGCLAVLAENQQQQPNKADKQQQPIKPGNEPRFIRAQDIRLLFNPLISNILTPTTTRAPHPVVVVPQYPYYPYYPNYPYDHEHLTHPTQPTTKPHTIIRPHYPHYPHYPQPPVYPYPSGYPHNAPVHPYYPPYGYDWYGSQYPPFVIVHPPINRTRSIGISNAQFEDEEPQYDLVDGMSARNIKVDGISITSGNSDDDEDSVIVDTKDIKLPYGITLGGFSGTSASHDSSHKKHNEQEALIYLRNYKKGRQLRESKTA*

>Woma_00005070

MSFWLIVLWTVLSTILGYLKVKFSYWELKGVKQLNTHFIFGHFFKLKSINHCDLLQETYDRFKGCAKLAGIYIYTKPMAVIIDLDLVKAILIKDFHNFTDRMPYKNEKDLLSVHLINVEGSVWKPLRQKFTPTFSSGKMKFMFPTINSLTGELKAAYERSIIESKEQMVDIYYLNGRYTTDVVGTCIFGIECNSLKDPTVEFRQICSKIHGRTNFNIRWFLFKHTYFWLTKFIGHKRYPQFIEDFFRRVTYEAVMEREKLGIKRNDFLNILIELKNTNDDKGKPVLTYDQISAQLFAFFTAGFETSSTNISLVLYELAKHPAIQEKARQEINKVLEKYNNELTYEAMMELTYIDQIITETLRLYPAILYLQRVAINEYAVPGTDITLDKGTSVFIPVRSIHHDPEIYENPSDFNPERFSPEQVAFRHPQSFLGFGDGPRNCIGLRFGRMQNSIGLVTLLKNFKFSPCSQTSDKIKTSNHSVVNIPVDGIKLKVEKL*

>Woma_00005139

MRLTFCLTICLMQIFSSAYGKSEGKYTVIAPGTIHSGDKYTAAVAVHHTSKPCQIKVGITGPSYNETKVVDVDANEIKHVDFDVPELKQGEYNLTAEGVNCMDNFKNTTELYYAKFYNHVRIQTDKGLYKPGDEINFRVIFLDKDLKPGQPEKDAVIWIEDGKRNRIKEIRNFTTTKGVYTGKFKISEFPVTGRWRMGAKNGAPFDKIVYFEVDKYVLPKYIVKVEATEAVSVKDGDMQVVVRANYTYGKPLDGKATVILTLGSGYYFDHSRNGGSNTEEPKHPPTLIKTADMVKGKAKIDFNVKEYEPHLPYKNSPSYMNVEATVEEQFTGVKINGTASTTIYPYRYTMNCVSYSTCSTFTAGKEMEVLFQITYIDGTTLKDTKSEIELIYKEVLNKNHWWHRQRELGGDDDDTEVITTTEAPVSEDRIVQFKSHMNETGYVNFKVSLPDLPDYEGYTHYYSMEMKYLDEQREVYSAYQYREPKNVDQPEEEDNKPKEYFELLDKYAFEKKLYLNKAEPFTLNSSQPLPYIDYHIIGRGNILKSGRVDLPDKPKVYNLTLTPEYMWAPNFVVYVYYVDDEGEYHYAEQRYHIDFELMNQINVTAAEQVKPGEEVALKIQTEPNSFVGLMAVDQSVLLLRSNNDLNANEFNWVLGSYSTNTRHQGGYSYYPGTIAGCVTLTNADYFYNLTKPVYLKRPNIASADRGFVSNDQFHLKTAVQHSAPAAAGFSGAAAGGGGGSANVQVRKDFSETWLFADIENTEAKEFTWSKKIPETITSWVLTAFSLNPTKGLGVTKDSTKIKTFLPFFISIRLPYSVKRGEVINVPALVFNYLDKALDVEVTLDNTDDEYEFTEFTNEVISEPKRTKSIRVPANGAAGVTFLLKPKVIGNVMLKFTAISPVAGDAIHKSMKVVPEGITQYANRAFFVNLKENPEVKNSFELDLPEDLVPDSEHVEVGVMGDILGPVLNNLDNLVRLPTGCGEQTMSSLMPNYLVMKYLKSTNRLTPTLETKLHYNLETGYQNMLKYLHKDNSFSSFGPSLIKDKPRNGSTWLTAYVIRSLKQIQEYVPVKDAIIEKGLEYLKQQQASNGSFTEKGDYFYNSQRNHIALTSAAVLAFLESDSATQKYSAEIKKGLDFIADNIDKTDSLHAKSVGTFTLSRAKHSAAKEQLSKLNALAKSESDRMWWSEEKNVKRGPRWWWFVFRNDVEITSYNLLTLLEQDSTKIDDILPIIKWLIAQRNSYGGFASTQDTVVGLTALIKFAKFADYEPAKINVDIESKDKKETIKLTEENGILYQNVEMPSKTKSVDFTAKGTGTALVQIAYQYNIFEKELKPSFTIKTTKLDKNQNPSHLDMLVCVNYAGEGASSNMALLEISTPSGYVIDDEIIKDLTKLKGISNVELKNADSLLVIYFDHLYKDDETCVMIEALRTHAVAMQKPASIVLYDYYDTDKKATAFYEVESKLCDICEGEEECSKCK*

>Woma_00005137

MKGFSLVVVLVVASVHYSTGFNAYQRRNVDCSYGACPYTPGGGSGKYSASYTSSSSSSTASSSSYGYSSCCNLNNWAYAHINDIRQFANRLRQEYNSMSSGSTSGYSTTYTPWSESIISLTGKTSGELDAICRLQSEQLVNDMRKGLVTYTTIAQPNFFEWKSADILEKYSVAGVDYGQQQSLDLNLENFDDVKTYNYPAEVKVVDGKTFVVHKNITEAHKSSGDGTFENPYVTVVKRNRTIITTNTVPVYTGSYVTPAYGSSSYGTTNAGYTTGGQAYAPAGSTTTVTRKKTIYDWVNQNMEPSVVGYSPVVNVNTNWGASSSNLYKPPVHIPVDNSAKSEVETFGTGYQPHAYRPGSTVTIRRYNKTTITNADGTVVSGSESHKKWVDGKLVYDNERPFGEWSVPRDEEWKREERKRFFWFLTSGDITPQKLEEWQRQQEERLLAIAQRYNCTLEEIQEWHRKELERYRVLLGQYRAQTTDDTNWKRLERGRLDWLIHQNSLTREELERWQRENNDKLAQLARQYRITVDELKNWQTEELERLYVFFNDQNNSMISRVPITDSLTRTSEQERLEELIRQHNATIDQLHNSIKMDQQKLSDLSLKYRGNVGDLEKWLKSELARLGGIITEQRNEITRITEWQQMERKRLEDTVKNHRGSVGNIDEQIAKDRGYLQTLASKYHISIEELEKWQRQELERLQKEGQMQIELGIKEWQQREHENLKKIIAQNDLTVEEFQTQIVNDRHRLQNLANTYNVQVTEIQEWLRKEINELKNEGLLNDIKKELVEWQQKERERLMLIVQQSHATVQDLELKIKTDQSHLNQLAANYNVKVSEIEDWLKKELMRLQSQGLVKNEELQEWQKAERDQIILLLQNNKLTIDEFERKLLSDRQKLADLARMYNVKTTEIETWIKKEGERLQTLGLLQIQEQLNNWQKIERERIMNLIQQNNLSIKELEEKIKNDQTHLYSMAHQHQVRVEEIEEWIKKEIERLQAEGLVELEKLKDWQTEWRGNLTNMVKERDFTVEEFHKWLLEDRARLQALAMQHNVKIEEIEQWVKNEEQRFVAMGLLKPNEKLTNWQEVERRYLERITQEQYQSTEQLEQRLRQDRELLEKLARDYQIQVEEIESWMKKELARLRDEGQLQIDNLTAWQIAERERLDALLKQNKQWSVEEFEEVLKQDRDHMQKTAFQYHTSVEEIEKWIQSEINRLQQQGKLNVEKMTQWQKEQQQRILNLLQQQSSITVEEFERKVQKDRTFLINLARQYHVNVNEVEEYVQKVIQDLKEKGKFELEQLKGWQLAERDYIINLIKQHKNEWTTKEYEDRLRSDRDHLNQLADFYRISVKEVEEWMIRELLRLRKDSSEQIAKLAAWQVTELERLKQLIRENNRLNFVQFEVELKKQRDHIQQLSQQYSVSVTEIEQWLREQLLNLKTTGEVQVENLTKWQEDEQKRLIALSLQQQREITNEEFEQQLRRDRSRLQKLADEYNVSVQQIEQWMKSELQRLKNSGLVQVEQLSYWQKIERERLQDWLKAQNKAATTEELDEFIKRDKARLQRIAQDYHVTVEEIQSWVEQEGARLQLLGMVKGPDNKISDWEFHESWSSKQPQNWNNQGMYTNEEIWKDQTKAHLLAVAQVKPMTWQEFEQYLKNERMRWEQFARQYHITVEEIETWLRQAAKELVPQGFIYGSTTVEEWEIKEQSHLQQLINDKLRKQQQWSIEELERQLINDRNYLQRLAQQYHLTVEQIIEWYRQELQKLLNQRKIVTENLTEWQKNEKERLYNLVSRNPYKNQQQLEQEILKDQTTLNRICDQYHVTIEEVEIWIRNEIKRLVNLGLIKGYNQGSGATGSQYNDWQEQERQRLRAIAADIPITEEEFLEFIAQDDYFQRQLTRMYGCTLEELAPFQKVQIAIMNKEGLLDKTALLKVEPWQKVERDRLYSFIKDKSYTLDNLRNWQKQEMLFNSLAANIGITAQELKDWQIQEFERIQNLAQYYQMNLNQLQDFRDKELRFITYVMHKKTASEQERMQWGANENRRYGVLQRKTGLTGDNLTQWRRKLYLIAQARLPFNYGGYGGWENGPGTTNRTALPNTNISKDRGDQPPNVFDENMDNDEPGVAGGKYMLPSPPSAAMKMEPMGHHTGGSYGGSYGGYHAGSHASSHYGGGYNQHYGHDDYGQQQVQMDDDFGQQQQQPVEVEDLTGTFTHRQPVQSEKQIEATGQIEVQAEAEKPGLLSKIKNRIFG*

>Woma_00005156

MCKFNNRLFVFHIYCLLGLQLVQVLAKPLDHRAKNGVEDFNALGELFALFSPQDVHYDQRQKGDENYRIKLDGFFIGLPQEDSTALLLLTDDIFESLDDEYLAAFGKNKPTSGPAEAPPTSTILSTTTSASLDSNDNLTSSTENSSNNSQARLVEIPAPKRFMENRENLGSSSTRPKTYVSHFLKLLKRGRKY*

>Woma_00005154

MRCLILYAALLSAVSGYRATSYTGSLNKITTTPHIDIQTLKSDSSSTTTRAEDDSTSSSTAQSSSSSSSSSTTSSSSSSSSTESNLIEEVISELPVSLPPVLEEVDLPSLPNRRVITYDQRQEGQYNIRADLDNFMIVLIPPGPSEGLGLLDLLTRSSLRRTSHASKGKKKHYTTATSLKPEALKHLNYLGVRSAEQMHMASSRPGEFLEGRTPYHVDITSLNEDEVQPRLNPDRQVDVLPPSYPLAYPHLMKPYHLEAESSLLPPSEMSLQHNAEAAQTQNTGYYRFARYIRGENFLDSNRVTSSNRLNPQSSLLRRQQLPVYRSELSMGAAQLYPPLDVPQFNFHVARNFDSKSSSSSDANSSAAEELDSKQSLDSDVSFSEIIIPPAHLPLEDDLDLALNDDSLLEGEAKALLSDGIERCAPGKRRDSYGVCREIEGY*

>Woma_00005041

MKTALVAFCLLVAIATTSIDAATIDNSLLAKYMCRTRPDGFKTLVPGSCSQYYMCLGGKTKIRKCPQYYDSVKQACVATNPGCEETAVDPVSCTGPCCGISNGGYAVDSKNKQIYYLCRNNCISVTKTCSNGEMFDLVKKKCSTPVNCPSCTTPKPPCEKPTTTTCTTTTTKPCEKTTTTTTPCTTKKTTTCTTTTSCTTTTTTTCTTTTTCTTTTTTTTTTTTTCITTTPTTTTTTTCTTTTTTTTTCTTTTPTTSTTTTCTTTATTTTTTTTSTTTTTTTTTCTTTTTTTTTTTTTTTTTTTTTCTTTTTTTTTCTTTTTTTTTTTTTTTTTTTTTTTCTTTTTTTTTTTTTTTTTTTTTTTCTTKTTTTTTTTTTTTTTTCTTTTTTTTTSTTTTTTTTCTTTTTTTTTTTTTTTTTTTTTTTTTTTTTTTTTTTTTTTTTTTTTCTTTTTTTTTTTTTTTTTTTTTTTCTTTTTTTTTTTTTTTKTTTTTATCTTTTTTTTTTTTTTTCTTTTTTTTTTTTTTSTTTTTTTTTTTCTTTTTTTTTTTTTTTTTTTTTTTTITTTTTTTTTTTTTTTTTTTTTTTTTTTTTTTTCTTTTTITTTTTTTTTKTTTTTTTTTTTTTTTTTTKTTTTTTTCSTSTPTTNTSTTTPCITTTKTTTTCTRTTPIAITTCHTTTKVPCVTTTTTTTCGCTEANKLKDNIENYMRIACRDKPNGFVMASLRRCTEYYICSFGEAVRVDCGEKYFNALKGICDLPENTNCNQAHYFLN*

>Woma_00005040

MDEDCIFQQDNAAIPVSKQSKSWFNEHGNPLLDWPACSPDLNPMENLWEYMASKVYANNAQNKGIKTVTELKLRTKQVWEEIDSDLLKKLVESMQRLIHTLLIMLQWPKH*

>Woma_00005042

MMNSKIVVSCLLVAFFVSSSEAAAVNNGLVAKYLCRSRPDGFQTIVPGSCSQYYVCDWGKAIIKTCPNFYDNVNNKCVNNNSGCVVTISEPLSCKGPCCGIPDGSYAVDSQNQQIYYKCQGNAIQQKSTCSNGLMFDLVKLECAKSAVCVGCATTRKTPVRITTTTKKPCTKPTTPPCGVTTTTPCAPTTTSCTTTTTCTTTSSCKPTTTTCSTTTTTCTPTTTTCTTTPTTTTCTTTPTTTTCTTTPTTTTCTTTPTTTTCTTTPTTTTCTTTTTCTTTPAC*

>Woma_00005045

MKTVFVLGCLLLAWAISTTQAAKLDRNLYAKYLCRSKENGFRALLPGSCSQYFECQDGISYLHDCPRFYDAVKQICVTKDTGCMRSLAQEVHSPAGCSGPCCGVASGYALDPLNDQVYYECKDNCVVSKQQCESNLMFNLETLECSAPENCGEVTTTAKPPCTESITTTTSAPCTQTTTTKAPWTTETTTTTTCTTTTSTTCTTTTTTTTTTTTTTTTTTTTTTTTTTTTTTTTTTTTTTTTTTTTTTITTTTCTTTTTTTTTTTTPTTTTTTSCTTTTTTTCTTTTPTTTTTTCTTTTTTTTTTTTTATTTTTCTTTTTTTTTTTTTTATTTTTTTTTTTTTTTTTTTTTTTTTTTTTTTTTTTTTTTTTTCTTTTTTTTTTTTPTTTTTTTCTTTTTTTCTTTTPTTTTTTCTTTTTTTTTTTTTTTTTCTTTTTTTTTTTTTTTTTCTTTTPTTSTTTTCTTTTTNTTTTTTTTKTTTTTTCTTTTTTTTTTTTTTTTTTTTTCTTTTPTTINTPTCTTTAPTTTTTTTCTTTTTTTTTSTTTTTTTTTTTTTTTTSTTTTTTTTTATTTTTTTTTTCSTTTPTTSTTTTTTTTCTTTTTTTTTTTTTTTTTTTTTTTTTTTTTTTTTTTTTTTTTTTTTTTTCSTTTPTTTTTTTTTCTTTTKNPCTPTTTTTKAPCTAITTTTKAPCTASNAVEIVKPLLSRQTNYERSVCRGKSDGFKFSSLTKCNEFFICSKGVAMPLNCGNKYFNALKGKCDLPENTKCNIPN*

>Woma_00005044

MNSKLVVLCLLVAFAITTSRAASIDNSILAKYICRARPDGFRAMVPGSCSQYYQCSGGKSVLKSCPRFYDSIHQQCVNTNPGCVETISKPLNCEGPCCGIKDGGYAVDPKNQQIYYLCKGDCIAKKNECPNDLMFDLSQLKCASPSNCVGCTTPKPPCKETIPTTTCTTTKPPCKETTPTTTCTTTKPPCTKTTTCTTTTTCTTTTTCTTTTTCTTTTTCTTTTTTTTCTTTTTCTTTTTCTTTTTCTTTTTCTTTTTCTTTTTCTTTTTCTTTTTCTTTTTCTTTTCTTTTTCTTTKAPCKGTTTSTCSTTTTCTTTTCTTTTCTTTKVPCKTSNEKPAASSNRLLIKNPTHYVLPLKMTQEEMDDYIRYACIDKPNGFLLPSMTRCNEYYVCRNNLALSVKCGRKYFNAKKGICDLPQNTGCIQAYHI*

>Woma_00005046

MVHKVWIFILQVLNLIPPSTHCSMFDMMPSSLRNVYGEKLNKLTWNTFIIIFYYNTSFNGSPAICLLDI*

>Woma_00005048

MSSVAANQTTIAAATTITAPRNSLSTKDLNIYEFSPLKKNCDLEHNDDYYMPSSMEILGIQKSLQHHVNDDENDDNNDDNENDFKDNHDDDLSHVELRRISSDNAVNNVGCGIIVETNGFARLKLC*

>Woma_00005084

MYKMFLKKFLYLLILFKLQLPCNSQQTKPIKYFLNKTHISVKPFSMQTARNGHLMTSNLDNFRDLLASTKLLMVQEYYRHIKINYKYNADIGRLQPQISPDQPFPCALNNSRSAETPTSVHRLRPGDIDIIGAMGDSITAGTAMMAKILPHLIIEFRGHTSLGGGLKDWRTFLTLPNILKVFNPNLYGYAVADTIAKFRPARFNVAEPIAITQDMPYMAEVLVKRMQMDPKVDMKNHWKMISIFVGANDVCNDMCFYDNMEDFLNSTREHFYRTLTILKENIPRLLVNLIAIPNLDSTTRTMKNIPPLCQIMHKTECHCVIGDSIKGRKFNDTVKTLRRFQQLYFDVASLPEFQLEDFAAMPRMSFVNKTLINLKNGNTDLRYFAMDCFHFSQRGHAVFTNMLWNDMLQVKSKSLNRLLKPFEVFGCPSEDLPYLSTLKNS*

>Woma_00005086

MAKILISAVLCLAMFGSMALAFSCPEPNGRFASPEQCDAYVQCQDGVAEEKLCPDGLVFQQRSKTAGDCTYAPFASCKERARLQPANATEECPRQFGFYSTGDPANCGVYRNCAHGVSTLTKCPEGLAFNVDSYQCDWPDLVKECNAEAFLGFTCPPAEIVDGVAVNVAVTPEGDLQYFPHPNSCKKYFVCVNGHPRLYNCGRYLAFNPDTKLCDFYRNIPECYARLKEKKERKF*

>Woma_00005161

MNFSYLFLLITSILLITTVQIDARGKGGGGGGGGGGGGGGSGRGRGGGGRGRGDGDVAVGGGRGSGGGRGRGGGRGRGEGGVAGGVAGGVAGGVAVGGRRGSGGGGGGGGEGGGGGSGRGRGNGDVAVGGGRGSGGGRGRGDGDVAVGGGRGNGGGRGRGGGRGRGDGGVAGGAAVGGGRGSGGGGGGRGSGGGGGRGGGVAGGIAGSVAVGEGRGSAASNLFKEIGIHAAGEFGREGARKLFDQGAKFYNDIKETVNLREILNKNTLFS*

>Woma_00005166

MKLNLIIILLASICALVFLNTIEAREIDKYVDDEDHKVIIYKNGDKYIENKRTGTVLIQRGKTTIVQAN*

>Woma_00005167

MKLYSALLALLVVVTVKLNYGQAKNGLYDVDGFIDDIINNPKSTDNIIRGNNIHNRVQSSSDIHFELFNNAEKPIKVAETEKLAPVLIFSTTVSAVRHDHLRNRYYTDNLNQNSIYKDNNHIKIEAYEGHKAYGNLKKDISRVIVKDNNGNVQQSTRISIGD*

>Woma_00005164

MHFIKISFILFITMVLYMILCEAGNKGSKLATDAVKQTANAMKGAASKLPGVGVGAVAVNSTGNLYINTKPTSRPSSPKKG*

>Woma_00005165

MHLKTALFFVFIISVTLLQSFAEAGNGVSKGGQMLKEGAKKLPYVVPATVSVNKTHDLTINTNPPSVSYKSRPNSPKKENKS*

>Woma_00005721

MEEQQFLIGLILWCLCAPVHLARIPEDLEPSAVKALQLERARSLTACGDDFDKDSSSTDSTIYDVCPPSKYRLPTGECNNVSHRKWGARGDVFLRLLSANYADGISQPRTSLETHALPDAEYVIEELQKHIDPELRHPHITAMLPAWGQLLANDLYEVSQLPIEGKCCKEVAERKPYEVEQCYVRLGADCKEYRRSAPGYDSETCSKHKREQMNVASAYIDGSGLYGSTLQDFQNIRTYISGGVKVDACKYCRVPGATGALHRALLQEHNQIAEQLAHLNSDWSEEDVFLETRRIITAQIQHITYNEFLPLVLGQETTAKEGLRLAAEKHATNYSSVNRGGIYNEFATSAMPAFLTMYPPEMLSRSMSSSQLLSVAALQKSLVPATPNEEGWSDLALAIHRARDHGIPSYVEALDLCDKRFADNSNITFDNLNKYSNIPEEHITSLKDIYQSANDVDLLTGALLEDPAVGALFGPTISCLLTLQFEHLKKSDRFWYENDLPPSSFTLEQLKAIRQTSLSGLLCASNQVQKAQSKAFIREDNFLNTLLKCDQIHKFDLRPWLNNPEEDAAIEHVEIEPEVKEVISELNPDLLEAAVERAKIELEHRKRFEYESWLAQGGISAKSPDGIAASFSKANRNALLLANSSLMYELASNEILNTLTSINRRKRQIFDGGLNFNRNELTETLQTIDINSFLQNKPVKSECDDPPVACDTTTPFRTITGYCNNLRNPNWGKALTTFSRLLPAQYDDGISKPRLVTVTGTPLPNPRTVSTIIHPDISNLHTRYSLMVMQYAQFLDHDLTLTPIYKGFHESIPSCRRCDSRQTVHPECNPFPVPPGDYYYPEVNVTSGERLCFPSMRSIPGQLTLGPRDQVNQNTHFLDASMIYGETVCLANQLRGFNGRLNSTIHPIRGKELLPQTNANPECKSRSNLCFIAGDERSSEQPGLTAIHTIFLREHNRIVEGLRGVNPHWTGDQLYHHTRRIVSAQVQHITFNEFLPRILSWNAVNLYGLKLLPQGYYKDYNPSCSPIIFNEFATAAFRIGHSLLRPHIPRLSPQHQPVDPPILLRDGFFNMDPLLQPGMIDEIMRGLVATPMETLDQFITGEVTNHLFEDRKIPFSGVDLIALNIQRGRDHGLPSYNNYRALCNLKRASNWNDLSREIPTEVINRFKKIYPTVDDIDLFPGAMTERPLQGGLVGPTLACIIGIQFRQLRKCDRFWYENQNPEVKFTEAQLAEIRKTTLAKITCENLEITGDMQRAAFDLPSNFLNPRVPCHSMPQIDLSAWRENVQGCQIGSRHVRVGESAFPSPCTSCVCSTEGAQCASLRITDCGQLIRQWPKEAILRDEVCNSQCGIYLSGQSGSFSAQSRQSQIPPPRVQRSRPQNLFKFPDLTPFIASL*

>Woma_00005822

MWKYFIVTVLLLWKDFCEANPFVGLFDPKCVVVDNLNCPSENVTFWLYSKQTQDTPLKLDPLKLNATDFQPRKPLYILLHGYTGDRDFSPNSYIRPALLQAEDVYVISVDYGSLVPYPCYFAAVKNLPAASRCLAHLINTMVDQGVVQNDDIHLIGFSLGAQMAGQTANYLKRRLRRITGLDPAKPLFITVDFDLKLDRTDAEFVDVIHTDVLGRGMLRTMGHVDFYPNIGPYQPGCQLENMEDPGSCNHDRAPKYYAESIHSDRGFWAYSCPSWLHNLFGLCNTNSNDEIMGHHVNRSASGSFFLTSSSTPPYALGRLNNTCLTYKSQLPSLYEYRDIYDFEPQLLKAFVDWTGNEFKEIRKLTDFDEKLEKIKPLDKK*

>Woma_00005821

MKIFISLIKALVLLQNFCYNSCGDNAAEYTDYSRFTCYFAEDQCPNKDIQFYFYTRETENNPILLDLLDLTADLFPQRLPLKILIHGLTLNHNLSPNKELRPLFLHQENSYVISIDYAPLTRLFCYFPWDVQNVRVVGKCLAVLLNNLMDQGIYASEDIHIIGFSLGAQVAGLASNYLNNKIGRITGLDPAGNVFDNLDPLDRLDASDAEFVDIIHTDPRFFSTRMALGDADFYPNFLDIVQPGCNILTGLNYCNHFRSSIYYAESIVTKVGFWSYNCGNYTEYLFNQCAKYAYKPQVEMGYFVDQSARGSYFLETNAEPPYAQGPFAQIELEVVDYIDEKSIND*

>Woma_00005828

MAKFIILMPLWFLLFGIKSGCETKQENNIVLPDLPLEHLKRYLNMFSIAKQHCQRTSKCDSSIFNTSLEADTKACWGYETDCDKKNRFQTPSCPGNHAGWVRNKESQMNTFYEQADFGYIKHQINELMLMCEPKLITDSSLECTKYLRFCRGRYLMLDFRDLAKRNERIRYHMDVLGPGQVKGYCKLNETRLKNEMDHMGALQSWSPELRNFVQASNTLDADNEECDMFINTPTFLMKIDATYNMYHHFCDFFNLYATLFVNQTHYSAFDKNTQIIVWETYPYDSPFADTFRAFSDNPVWTLDQVKGKRICFRNVVLPLLPRMIFGLYYNTPIIQGCHSSGLFRAFSEFILHRLRIPFEHPPQQKIRITFLSRRTKYRRVINEDKLLEEISKNKNFIIRKISYERDLSFLQQLEVTRNTDILIGMHGAGLTHLLFLPNWATIFELYNCEDPNCYKDLARLRGVNYLTWEHRDLVYPEDEGHHPEGGAHAKFTNYAFNVKEFKRLVEKAAKNVRSHEEYQSYQEKEAVTMLKDEF*

>Woma_00005752

MRHSLKAFLIVALLFSIEVLAKKHKEESSEEKEEEGKKYEEDESEEKKHKNEKGHKEHKEWDEDEKKHHEEEDHKHHHESKGGKKKKEEDEDEKYGEEHKHGKHMKGGKDHHKKKYKKGHKELEHHKKFHKDEYIKEKKFYDDEDKGGHHKKYGKEHKHHDEEEGEHKKNEEHEAGKKKKHKKHEGHYKKGHHDEDHKKYEKEHKHKESEEEHKKWGNEEQKKSNKKKGHKKESEKKD*

>Woma_00005739

MLINDRLKCILFLSVCLACLINSQTNAGKSARKAYYIENEKFLAWKDAVDYCKNLSMNLFSIENFDEFDYLKSIIKLKYHRVLPYWLGAYKVGGVFKWIATGENIKLFLWHAGEPNDNSGHEKCIHTWENDFDWNDNDCERELPFICEMLE*

>Woma_00005738

MKFIIITLLMLSSVHYLLAASSPKSKDVDSSDSYDYSDEQDYPSAELKKREDNNDDWENALEDENDDDEYCEWLHCDKDLRQERGFRRYFDFPFLKKLVRNLSPFSRPRLQMHFYLFKREFPDCGRELIVGNDNSLLESGLDASLPTRIIIHGWMSQSRGSFNRDVKNAYLQKGDYNVIVADWSANAANLNYFHVVKLIETFGTYVAEFTKYLYERAHIDYDDIYLIGHSLGAQIAGAAGKRSRPHLYNTIFALDPAGPKFRKKSADFRIDPSDAKYVESIQTSGNLGFLEPTGNATFYPNYGSYQKKCFYVGCSHIRAYKMFVESINSPLGFWGVRCQRREPEWQCDDMDTASYRMGGEPSIPKTGIFYVNTNSRQPFALGKVNVKGNE*

>Woma_00005815

MNYLILLITLALCLSSSVHAGHNNNYYVEPNYNFKWFEAHIECYNRNMTLFTIENEDAFYDLYNILISGVFNRKPPHLWLGGIGAQRKFTWIANGKPVIWKYGSPDNARNSENCIQLYENTKDINDRNCNDQLGFVCEENKYQRKCVQGGEKIADKYVVLNIYQQNKDKK*

>Woma_00005799

MLPLPRGLWGLLGSSTPNRHSLLLTTVVAILLIFALFENTADAKQPDEKCNGVVCDCKGLKGRPGDIGLPGFQGYEGPAGDLGPLGPPGRPGEWGDPGEYGGPGEKGHRGDPGFAGQPGLPGPRGRPGDDGPQGPRGIDGCDGKTGQQGAPGLPGRHGGRGDVGKPGPPGPQGDAGEGGINSKGTKGSRGDRGTDGYDGQQGYPGMKGYKGDTGFPGLEGPKGEEGPKGYKGEMAESVDLQLQEQGQQGEKGEPGDVEEGAWIPSEIQKGYMGDQGFTGNKGYRGMQGEKGEIGRQGLPGVRGDTGEPGERGKPGKPGESGKPGSKGVKGAPGYNGLDGEDGPTGEVGDDGYNGIPGVQGPPGPPGIYDPNLDISFPGPIGPQGDIGETGPPGLPGIPGGAGRRGQRGPTGPPGDAGLDGLPGRRGASIKGDEGDFGFMGPPGPMGPPGNPGPIGRVGNQGEPGKNVLGPKGFAGQHGVPGLPGYRGDRGEVGLPGEKGLPGLGVDMVGPPGADGMPGPPGQPGVDGFPGLSGPIGDKGIRGDDCGFCPAGPKGITGIRGDAGFPGTYGARGPQGITGERGLKGLPGRPGLMGFKGIRGTDGIPGESGQPGLPGPPGKVLKHGTQRKPEDGDRGDVGEHGMQGEKGERGVDGFPGMRGAKGETGLRGDYGDAGYPGRDGAPGRPGKDGRPGRNASTPKIYLIGQPGYDGRKGEMGDPGDVGPKGEKGTRKPGEIYDNKGQRGDEGYPGEPGPEGPKGEAGTDGIPGIRGDPGLPGPSIAGPVGFKGWPGIIGDIGLHGSPGIPGRNGAPGIDGRAGVKGMRGSPGPNILAGEIGPDGPPGIAGISGDVGFNGFPGPSGKPGVKGVKGLKGDDGPLGLQGLPGNKGMRGDTVVGYPGMAGEPGRDGRVAPHGRKGQKGETGLPGPDGVQGAKGNIGYTGRRGAVGDKGFAGAPGLMGMQGFVGIPGEQGDRGELGEDGRHGDMGPRGNIGLMGPKGQSGEIGGFGRPGRDGSHGRKGQLGDKGFIGPVGPKGFASRSGIKGEAGEMGYRGARGFDGLPGEKGIQGAPGDQAYGEDGLPGRKGETGAPGIPGLDGLPGLKGARGEYGLMGLMGETGDQGDEGVPGYPGKPGAPGIKGAVGEIGRMGEVGEVGEIGEMGFAGYNGPRGDRGDTGIPGAFGPQGEQGDEGFPGRPGKLIMAYAERGQKGEAGFRGEVGPMGEPGMIGVPGFPGRKGERGDFGLAGIPGVDGYAGVKGERGDKGYPGAPGKTPDFAEPGDEGDAGYDGLPGRPGRVGPKGAPGDVGEYGLDGETGEMGMSVMGVKGVTGDVGHPGPMGRNGLHGMAGLKGMMGDIGPRGMLGEPGMVIPGLRGDKGDTGPQGVAGFDGRKGEQGMSGRPGRTGPVGPRGPRGPPGDAGWNGGDGLDGLKGPTGEPGVTFPFNMARKGDRGEHGLDGFKGAMGDIGVEGEMGFQGARGLKGYQGDQGFIGLVGADGPKGERGMTGPPGLTGLPGMPGPKGYTGEPAPPPPPPKSRGFIFARHSQSVYVPECPANTNKLWEGYSIAGNIGSSRTVGQDLGKAGSCMMRFSMMPYMTCDINNVCNYAQTNDDSLWLGTAEPMNAAMAPIQPHEIQKYISRCVVCETTTRVIALHSQSMSIPECPGGWEEMWTGYSYYMTTTDNTGGFGQNLVSPGSCLEEFRAQPVVECHGQGRCNYYDPIFSFWLAVIEEHEMWSMPRPQTLKKDQTSKISRCTVCRRRNDSFVRRLPTLDSAAREFRRGHERFVPAPNAAPPPPPPPLRRTNTYYNRRPMPGSQGYGRVNRYRYPRADTTAP*

>Woma_00005798

MEPLWKRLLFAAVLAGALVGINAQFWKTADTGAIYNSAKHYRPEVEAPRNPIDESYAILDSATTTYTGQVPSNCTGGTVGCIPKCFAEKGNRGLQGPIGAPGPKGMPGYPGPEGPPGDKGQKGDPGPEGPRGLKGERGIAGIPGMAGVPGIQGIAGNPGAPGIPGKDGCDGEKGLPGVTGLSGMTGPRGYPGQPGAKGEKGEPAKENGDYAKGEKGEPGFAGRSGNPGPEGPTGPKGDRGDTGPYGPPGQRGDRGQKGEKGAPCFARPQPGKKGEKGEKGEAGGAKPFRGGKDTYTGQKGDRGEKGETGPPGEKGDIGFPGEPGRDGMKGEKGLPGPSGDRGRQGSYGPPGPSGQKGDRGETGLNGLPGKPGQKGEPGRPGSPGERGLVGPPGPPGGGRGSPGAPGPKGPRGYTGPPGPKGLDGFDGPPGPQGLPGAKGGPGVPGHNGVEGPPGEKGEKGNRGRDGSIGPVGPMGHTGPPGPEGQKGEPGLPGYGERGIKGDDGIPGTPGAKGQKGERGFPGNVGAPGDSKFGRPGTPGRVGAPGQKGDAGRPGTPGQKGDMGPKGDAGGKCSSCPYGQKGDKGDRGANGAPGLPGLRGPPGERGYPGERGADGIAGPPGLPGDKGKDGLPGAPGPYGIPGKDAILDMNLMEVIKGQKGERGYPGHQGVKGERGESGTPGYPGEKGEMGDKGDKGLMGPPGSDGAPGTPGRDGLDGMPGQSIKGDTGRAGFDGAKGDKGFSGPQGEKGEPGSCEVDYLQIPAKGNKGDRGLQGNPGPQGPQGPKGDAGRPGEKGDRGPQGPVGPTGARGLTGPRGEKGNQGPIGAPGNPGKDGLNGPPGRTGAPGQKGEMGIAGVGPRGPPGRPGLPGDKGERGPTGPYGPPGKDGAPGYPGEKGDAGYPGAPGLPGLDGAKGDLGPIGPPGPQGPPGKPGVDGVQGRDGAKGEPGNPGLVGMPGLKGERGAPGNDGPKGFPGVTGQPGKRGPPGPAGIPGAKGDRGERGLTGRDGLTGPRGPPGPPGLMGIKGDMGPVGPPGADGLPGIDGEKGDRGFTGDVGPRGEPGDAAEKGQKGEPGAPGLRGEDGIPGAPGPQGEKGVAGIALHGRPGAPGEKGDRGRDGINGRDGAPGPKGDQGLTGLMGAKGAKGEMGLPGIPGAPGMDGKPGEVGAPGPVGYMGPKGDKGERGYPGINGAKGDKGDQGLPGFEGAPGPKGERGLTGPPGLDAKPFTIKGDKGEMGEAGLIGLPGPMGLKGNQGAPGFDGPKGDRGLTGPPGAPGLNGVPGAKGDMGHRGEPGPAGLTIKGEKGLPGRTGKNGREGAPGPAGLKGDKGLPGLPGTPGAMGPPGLPGPQGPKGDRGPMGAPGRDGHDGMPGVQGIKGDMGYPGPKGEMGPPGFQGQKGEKGELGFTGAPGLPGRPGPKGDMGMNGLDGLPGPIGAPGEKGYPGPAGINGRDGKPGAKGDKGEPGMVPPPGPKGEPGYPGRDGQKGERGPPGPRGLIGLQGERGEKGDQGLPGPMGREGVPGALGPKGEPGLACTAAQDYLTGILLVKHSQSEEVPRCEPGHIELWTGYSLLYVDGNDYAHNQDLGSPGSCVRRFSTLPVLSCGANNICNYASRNDKSFWLTTSAPLPMTPVPASDVSNYISRCTVCEAPSNVISVHSQSLDIPNCPNGWESLWIGYSFIMHTAVGNGGGGQDLSSPGSCLQDFRTTPFIECNGGKGHCHFYETMSSFWMVTVEESQQFRRPAMETLKAGSLLQKVSRCQVCIKNSS*

>Woma_00005814

MKLISLFIIVTIIGTKYASTTGAPLTKWFKTDNNTYYIEPEAKYTWQQSYEECQKKNLRLITLKAYKEDQLLLQYLKQTFDPQPEFWLKANDNIDLSEINKLLGSMLTGDFLNLKLDGVNDGQVVQSNNPDNCALTLDGTVKACETLHGFICHQNTTSGNGGHNVQNIVLKFN*

>Woma_00005812

MKFFGIFLACCAVLLLSAVNLTESAESLNQLTDGLQDAFGSALHEPVELNRQKRLTCDIDRSLCVLHCAVKRFKKGYCSQQKVCICRYPF*

>Woma_00005818

MKPPASYPEQTEFLSYKPTITHVCHCSMESLCLSIKVVESIVKKTTNELENCDTYIPPPRQFNNKIFTHQETSQIKKKILKNIC*

>Woma_00005819

MRAWLLFLAVLATFQTIVQVASTEDNNNNNISSSVITAIQPFEKDLTATPTTTTAIKQKPPSSSTTSTALINLRDFQNTVNTEQELNKQTIVYETHKQSDKLSEIQQKQEQEKQQQQQLDYQEINTNIINQDQNNKNIIVEIKNIEDNEVLVEKEKSYKNSQKNNYNKSDKNFNNNHNLKHINKMALKESELEQQQQENINQKSQEEDTLSNKNTESSSSHRHHQPQHKHHHQHQHQHQHQHRQHQRHQQQNLQPASQQQQTTTSTTTSDNLESKAASTTSKSSSSSSSSLSPALSSSSSSSAPTKLDLDVTVTENDDGAIVVESQQPQLDAVIPDEGPRTFSNQEIIAEKVKPDPSALVEIENSLLSLFNMKRPPKIDRSKIVIPEAMKQLYAQIMGHELVSVNIPKPGLLTKSANTVRSFTHQDSKIDDRFPHHHRFRLYFDIKSIPADEKLKAAELQLTRDAISHATLNPRLANRTRYQVLVYDITRVGVRGKREPSYLLLDNKTIRLNSTETVSLDVQPAVDRWLATPKKNYGLLVEVRTSRTLKPAPHHHVRLRRSADEEHEHWVHKQPLLFTYTDDGRHKSRSIRDVSTRSKRAGGHHRRAHRRKNNDEICRRHSLYVDFADVGWSDWIVAPPGYDAFYCHGRCPFPLPEHLNSTNHAVVQTLVNNINPGIVPKVCCVPTQLEGISMLYLNDHSTVVLKNYQDMTVVGCGCR*

>Woma_00005744

MYFFAIYLLLISTSLANAAPQSLEPITTVKPLYKTSYLTSYLFEEQAPRIAGSGMGFISILHSSNNNDGEGTDDVELNIIVDFLKDIEKPLSNAIEDLVREAETLNGNLDDGESEVPVEDENGN*

>Woma_00005745

MYFLLPFWLVVIGTSLIKAAPQSRIESNTTPLTKSYSIDTTPHPGPDPYNPEANNFSVEPSTKAAGIGFILILQSYNSQSKDDDNNFSDQVSESNFNVIIGFFNGTEETSSDTLEVLVRKAQTLLEPNENFHDGQLESLLGDLGTVRIYKISEVMKDIVPSSTVSSQAIE*

>Woma_00005742

MYFLTLWLAFIGCICLIKAAPQSNSYGWDSYYYGTEYPPFETTTLPTTSSGIGFILQIQSANHKDKTQGEDMTDQNEYSGFNVFVGFINGKQDISPDVIKPLARKAQMLLKTNETLTDDEQNSK*

>Woma_00005268

MKQQKIKWFFAIIALQLCLIFVASNAEENTSNIKIAYYEEIKDLPNHPEILLIDVRSPEELEQTGKIPTSINIPLSIVEQELYPDLKPEDFKNKYGRDKPVADTPLIFSCRSGRRAQQAAEIAVKLGYKNVKNYKGSWLDWAEHEGLPKE*

>Woma_00005189

MTEEWKSFCETLNKEGCTINSPRKQAEEIRKKRAKFAYYINKQGVFSIKISFLRHHNYWSNKSYQQKP*

>Woma_00005206

MKLFVLYSTVILLVVTEIQANIVVMPEAMYSTASPTAKVDNYILQHQRQFEEQINTLDRYLEDFRKKFDIRLDMIEYYDAIVDVKLRDMKDKLDPLEMLGDINDQCVEKYRGKIPADNVLKESLKTCIKKAKTALTPLLNNPENTLKNIKNHYNNNFANAIKECKRKFEKDETKYHNCATNAIKTTNNYFITNTNTFYTQMSTAECSSTTKIDEAFDCYSVNVYQTFTSIGEVNGLVENCMAGHDYCPPCKDDADDLSQIKGRCPYQMAWHLADDDLMGDKIVNPFKGINSTTPCLQINFITKSFSNSVKKM*

>Woma_00005204

MYKLFALLFFILAVTNAEVQNISLYVISSYNSSVLNTDAYINLNKQQHEKNIQDYDNQIEIFKQSYAASLNTIQMEDDMLIDSLNQANERWNSLQVLSEFSRKCVNKYIANIPKISNIKISINGCINTAQSQINTMLSPALSSKTYLENYYNNNYEQGLKSCKNKFNLDNYYLNYTICVVEVTNSAQDYTINSQKTFASYMDTARCSAETNIKKALDCSYSIQNKTISQIAEAKTLINKCLFGQDDCKQCSAGYQCPYITYMAKKDVSSTSRTMENPFYGQPGMRDCVMIKLY*

>Woma_00005203

MYKIIAFVLYIAVSLTSGQKHNVTPYAYNNLHNITLSTETYIGITKQLHAYILQDYDNQMDAFKQTYTDTLNTLEIQGDMLIKSLIETNDRLDYLQASSATNTACVNKYRSAIPTIASSKVDISNCINTARTQMYGAYGLNGMLLHAEYTRSYLENYYNNIYAYDLKTCKTKFSSAYYYLNYTQCVVDVTESAQSYTFQNQKTFATQMDEARCTANGHIKKAIDCTYSVKNRTISRMAEANTLINKCLLGQDDCKQCSSVNTCSYTAYKNRYNVSYTRKAMENPFYGMVNTPIEDCFMIKIY*

>Woma_00005202

MYKLFALFFFILAVSNAEVQNVARQAITSYNSSVLHTDAYINLNKQQHEKNIQNYDNQIEIFKQSYAASLNAIQTKGDMLIDNLSQTSERHNSLEILSEFSRTCVTKYRKSIPTISSTKISINGCINTAQSQINTMLSPALSSKTYLESYYNNNYEQSLKSCKNRFNLDNYYLNYTVCVVEATNAAEDYTINNQKTFTSQMSTAHCTANVNIKKALECSYSIQNKTISQIAEANTLINKCLLGQDNCSKCSIGYSCPSVTYVDRDDVRSTSRTMPNIFYGRTDIKDCLIMKLTY*

>Woma_00005200

MYKIITVLYLALAITNGEVLSNFSNETYANTLQSYDMQIEMLRQPFNAGLNIINMQLNLLINSLAKMEEFLNIFQSLNSVNQACVSKYRSSLPTPNAVMSSIGSCIINAKIDLNQTLSIPLATRNYLAGNYTYVFEKQMDYCAQMYGANSNNLNYTACITKVNEAAKSYTTSNQNTFHSQMSIARSQADIYTKKAVNCSFEALDNTIFAMSKTDTLINSCASGINCVKRNMDYSCPNINYISSSDVNPFSNIMQNPFYGRTDITNCLMLQIIG*

>Woma_00005388

MIKLLLFSILVINFLYVSPSTDLATQNDPNGPVTHLMENLAAHTSSVTNELLAQLAAKIERLAKTLIGNIEVVITSALHQLAQPFEDARKKINSPQCQTAFSYEELKRRVDNDLSNCTQNLNLILHTFEADTNAFNAALKENVKEIIDLPKECQNVANETDLGSNVSCYVEKIAEFNQQVAVVFNRASSTLLHTRQLGEQAMETARSCAENVVASTIDYLDKILDNCQAIEN*

>Woma_00005312

MKNFVYLTIAFTLLVSSSMATTVRNCPKTKARQLEQGDVTISNCPKSKCILKRNTDVSIEMKIIPNRDFKELNSDIQGIILDVPLPFPGYYGTSACPHIYDAEGKNQVGCPLKAGETYIYKNSFKILPIYPTVSLDIHWGLGDKEGDAACFQVPAKIKA*

>Woma_00005199

MYGNKLIATLVLLGVIAANAQVQNVTTLSINEQQQPITRPDDYINSNRRTYANNIKDYDQQMDNFRKSYAERLDTIYTQMGMLLDAIEQCDERLNPLELLSDFSKDCVRRYRSSIPTTSATKTSISSCVSTANNNLNGMLSSALSTRNTLQNYYTNTYDKGITNCETKFKDHAMNYTLCITGLTTSTNSYTVSNQKTFATYMDSADCSAKANVKRALDCSFAVQNRTISLIAEANALINKCLLGQSECKPCVGGYSCPDVYYVKSHEIDYTNSTMPNPFYGRHNNSSCLMLEIYDFNRIYS*

>Woma_00005287

MINKILQFSIITLWLFLDLPSKTQCRHHNRNNGGGGGGGGYHSSQMQQVPNIDVRDKILELQCYAKCHESLRGNVDYEPCQTKCQIDMVKAPRRGYCPAMQNAIFQNINVQPLQKLSCLDNCSYDFDCPEVQKCCNSACGPVCMKPIGVRDDSMLPPIPKILKCGLIPREQKVEITLQSNSSYYFHVEVRYHIGTLLSPRKLGTWQYQPVQKLAEILDLNVLLTTVGFYLRPGRWYQVRVAAINAYGFRGYSEPSQAFTLPNHPKPPKAPADLKVVSSHFDGKYVKIKIVWCASKSNLPIEKYKIIWSLYVNNNVRDESLISNEAFVKDRHQFEISNLLPDSSYYIQVQARSINGKRRLKSEKHSILYNTTLSSIQAFNPLKCGKEQQILRNGISNAFNDERYGGGYGKKHFFNDGDSLMSSPPSYASSSSSGSTFISNNNLNTNHHNSNSTYVTTRTEKFDVKYRPNRKLGMLVIISGFYPRNERIYELCPIDTNCEEGEYNAIRVNKDSLVFSKLSYNTTYSFKPRSNSVTIDENMKGITFTTPKCESFRKGHPKANIKC*

>Woma_00005309

MFSYNVAGFAVVTLLVLSCVQALPPKCQRDDEDCRDRYMHPDLPEIPKQYQRVVSGENTNEYWRNIGEDFVFKQLMSKPNKNQAKNIIMFLGDGMGLTTIAAARNLLGGEEQSLSFNEFPYTGLAKTYSVDKIVPDSACTSTAYLSGVKAQEGTLGVNGNVDMGDCQAGNDRNNWVYSIAKWAQDMGKSTGVVTTTRITHASPAGIYAHIAHRNWENDQEVNAECGLNSGVQDIAYQLIKGEVGSKLDFVMGGGKKHFIDANLYKNGKRRDGLNLVEEYKKQSSKNLYVETRDELMSAKLKEWDRVFGLYQDDHMLYHLETNETTTQPTLEEMTSKAIEFLSKNENGFFLFVEGGRIDLAHHQNMARIALDETVEFSKAVAAGQELTSEADTLIVVTADHSHAFSYSGYPYRGNDIFGRTPANSADKIPYMTLSYANGPSFVKSFDSAKGHRIDPTTVINGDKYDQFPSSWLLEDETHGGDDVPVYASGPWSHLFSGVYEQNIIPHIMGYASCLGNGLQMCHRQSDNQ*

>Woma_00005362

MRTSSVLLLTVIVVVAIYGNYAKASIIPLGGVVPPYVVLQHHPHVVVASSGNDGSAVIAAAAEAAENFANTVQEAGNQAVVSAQVAADAIAVSNQAILDAAQAANDKAAADIEAIANAQAEANAAAVANAQAAAESAAAANIEAANAANAQAAAANAAAAANAQAAADAAAAANAEAAAAINAQAQANAEAAANAQAAAVANHQAAAAANAQIAAAANAQAAAAANAQAAAANAQAAAVANNQAAAAAQAQAAAIANAQAAANAQAQAAANAQAQAAANAQAQAAANAQAQAAAIANAQAAANAQAIAAQNAAAQAAAAQNAAAQAAAAQNAAAQAVAAQNAAAQAAAAQAAAAHAAAAQTASASGIVLSIVPPGLPGSPGNPGAPGGGGAPGGLGGPGGLGLGLSGAGGAGSAGGASGGAGGSGGSGPAGGGGGGAGGAAAGGSGGGGGGAGGGPLGGGGAGGAGAGGAAGGAILSGASGGSGGNGGLSVGYACGADGPDGLNGADGPNGPDGSVGGRGGKGGKGGKGGCIGAGGAGGGAGGGGAAAIPAPAWPPIVLNTIQW*

>Woma_00005363

MKPFLIVCALLVATCTASYHGAVSTQYAHLDPHSHSYSYGYADPNSQKHETSHGGVTHDSYSYVDAYGHKQTVSYTADPHHGFHAVGTNLPKGPAPAPVHAVAAYDGGHGYDGAHGYAAGHAYHGPQAHITLTHAGVPHDTPEVAHAKAAHAAAHAAAAANAYSHGGDYGHHLYKRSLWGHGGYAHHAVAAPVVLTHGGVPVDTPEVQHAKAEHYAAHAKALGAAVHAHGVPLDTPEVAHAKAAHFAAHAAAGHGGASHHAIAAHGYHVPVIHNGVPVETPEVQHAKAAHFAALAKASAEAGHSGGGDDGSWDGGHGGWDGGHGSYGGAPTHYATSAHHGHYGPYKGPLHIPVIHNGVPVEPAEVQHARAAHLSALAAESHKSAGYGGHYAGKW*

>Woma_00005358

MRMKQIILILCILWASSSAARPFQPTSFDYDYEIESRRAPTRRGRERATSTTPRPLIQQYWQNFVSNLPTLPPLRWNMINPLGNLFTAGTQQIIETPVAMHYLTEETNQKRINSRRKTAKKSKNRKKLSSSLRNSYDNSYHENQLQLHLQPLGVDRNRVLHLYDPASGQFYALQTETQPSFYNQYRQNNGYDGFYDNKEDNDDSYHKHNGYEDENRGNGEQDDGYGEDYEPYGHEYISDESDNHSAYDDDADVEVVDNWGYNDEEVFADRKLKQETYNLNTLASNIIKVRKDLLKPETAEDVVNQLSTDKISEEGKSTVKIKKDDAKKTDTELGRNVWGSSIKDYGNTKRKQNIKRKSSLKLINVSKARGKFYIIPGNIFS*

>Woma_00005190

MSFRILTPLTTNRMSRLMFITLLSVVLILTPRNTQAARRSRLDLTAASDANDSLSSLVNADALEPALVAATNEAAKTQESLKTDQSSSTTSSPASAAESLPLTQDDTKTKDSTATLAANVNTGKSAGTDVSAEDDKALQDAEELPQEQKEVPTSSFEEVEIVDSATQLASSGSGFSTTQQQHEECDSDMLGFEIITGYVFSAPGKLLESMPGTLMLTDCLEACQSNETCQAVNYETGLCVLFSANADQLPGALTKSQFPVFTIYAQKSCLGIRPCARAWCIDRVQNYKLNGHAKRSVSVSSRRDCFELCLGETEFTCR*

>Woma_00005195

MSLLLRVLIFQVIYAAIVSKQDAGGFTEDFRYDYGIVENLYGPHMESTSLSNGYEFDVVDLLDLGEKYQERLERKPSLRNSASKFLLEVYNEITENEHELDHVREHLAHGLRQKRALREDNFITKFDRMEIEKCNNIITFSGKKTLSLDEGHLIPNITIPSDMQIGFKTTDVPRDLQLIHAALRIYQKPSCGKYMQDTVNQKAKISIYQRIMYRGEYTGHLRIITSANTSTTYKGWLEFNVTGVLKRWLQQKSGRINELVVGVTLITSNSITAEILTSDLGLITPDISEDIIDLQPFVIGYFNEPELMVKIQKLRFKRDVLKRKRKAIHNLPPERMREIYETPKTCERLNFTVDFKKLNMHNWIIAPKTFEAYFCGGGCNFPLSSKMNATNHAIVQTLMHLKQPDLPKPCCVPTVFDSILFLHYLNEDSVNLRKYPQSVAKECGCH*

>Woma_00005198

MYGYKLIASLVLLEVIAANAQVQYVSTLSINEQQQILTRPDDYINSTRRTYDNNIKDYDQQMDYFRKSYAERLDTIYIQMGMLLDTIVQCDERLNPLELLSDFSKDCVRRYRSSIPTTTATKTSISSCVSTANNNLKSLLSNALNTRNSLQDHYKNTYDKGITNCESKFKDYTMNYTLCIIELTTSTNSYTVSNQKTFATYMDSADCSAKANIKLALDCSFAVQNRTISFIAEANALIGKCLLGQSECNPCAGGYSCHDVYYVKSHEIDYTNRTMPNPFYGRHNNYSCLMLEIYDFNRIYS*

>Woma_00005231

MLRLILIALSFAICSAESDHKTIDVETSLGQLKGTQMESRLGQKFWAFRGIRYAEAPVGELRFQNPQPVKPWKPQIYDATKDGPICPQVTQNLTYLSEDCLRLNVYTKDVNSRKPVIVYLHPGGFYAVAAISSYAGPENFMDRDIVLVTLNYRLGSLGFLATGTAEAPGNMGLKDQVVALRWIQQHIEKFGGDPNSVTLWGYSAGSLSIGLHMMSPMAKGLFHKAIMMSASPLGQFKYKTDQLNLAEKQARLLKCPEKPIKDMVKCLRTKPMIDFVNTTEEMFEVGWNPVLNWVPVIEKHCDGNHESYLIEDPYTTMSKGNIYKVPLITGITEYEFYYLGYYTLRNETQRKYFNEDFAKYTPIFFLYERETPKSKEVSAAFRSFYLQDKPLEFPQSLIPFGKLYSDGLIGFEYHRFLKMVSKHTPVYTYLFTYKGRFSHFVDPETNQTLGAMHHDELLYLFNAPLFTPQFKKTDPENDTIERLTRLCYEFAKKGDPNNSTDEYLKAVKWPLYSEDKQQYLEIGQNLNVKSNGIYPDRFQLWDRLFPINEMLNSNIEQCKD*

>Woma_00005216

MLKLALRLSLAFTLCLLCTATALSSAQLPLEARGGESSEEEEVISTSYVLPNQIFNNGKPYYASKDPISGQLDFNIKKPAGIDSAVNEVIDPNEKAIITSSSPNIHDFLNLPVKYSSSKFVYPLVSSSYANLKYQGSNKNYITNKKPTQVVQSTSAPNYHTANYYTVPTTKLTPVSPTTPALVSTVGSSTTSSTSTTTTKKPTEASSSSSSSTTSTTTSSTSTTTTTTTKVPVVQTVPTVQSSSSSTRKPMSTTTTPASKTSTTRKKFVPSKKYTPTPPQTFTSPVPVNTTNTPRPTTTTNVFTTQPAKTTEPVMFTEDPTAPPPFSPFPSSTRIPNMDPADVYQTLGQKNTENKFEQKPSMTLADIFNSLVEEESAVVANNPMHQGLDAQGNLMEPLDPSKPSPFAMHNVEEQRQPQPTKPSVETTATPLQQQQQQQQQPQQRQPSVVAGSQETLNNEYVSYQVQQPNIMQYRPAPGAINNVVISPGQNLASFVLGSQQQVGQNAFGSVEKEPLFSKEHPVQYGTVINEDISGISRQPVPNAPSPYQEMPNKPTISNFHQNGNYALDTNRPETQRPLAPGPSYQELPPVPPSPYQQLPLIGNIRNKPSAPPHPKPSQTQTNQVLPSAKPIGTVQPVNPQQTADNSITLDNSQNTKELLVSTNIRFPANEEQSPEATVANGPPVGPQVNGHAQPISLQQIQQTSNPIVFPKQEEGKLQQNEAMYHNGPNSNTDGVQIQQHEVLTMSQHKQKLTFPSTNEPHTPAQHMVPPPPPPPNSIAAEHELQKPHRPQYSYNEYTRKPIPGITRPNPDRNLPNILPQFRPNAKISSGHPQPGNFNQEPGNIRVTGPQQPKRGPINGNPQFAHRRQPLGGQPKRYPLNRMTDYPTGLPPSNEMTNRRVYRLPPYGGSGVQYLDRPGSHLRRPMPHNLRPAESLNVERHSLAPVLEKLPAFEEEDLVINDPPQPVTPNKEAGLFSQTKLEPVVTLQMLQSQKKAVSLPGDDSGDGEIQVPSLDNDEAETGSEAQNNQNGLYVVYPIKGGKKSENLEEGPENQNALPAVAHVETPTGSDYQNTPFSIIRDQQQEPVLKNKKPLSLQQQQKSKSQQVKDSSFPYPIEKPDPSYSELNSASRVPGVVIAPKIRNGAYGMATEAPIAIAYTPTEPSRYKSSAETLQQKKPQPSYSNVNLATPVIEEIRPDSQTEITLNGHDFDLRAQNFEKNFMAPFYPSVSLDSVTSTPKNGWNVLPSSTGQNLYEKNNIDRSDVGGTHSKNDEAVESSTKSFEMDKFQPELQGGFKPIYPPGYKLDNEQDEQEQLQQHESNNMPLALASRMELPQKLQQLSEGLETSSTTSSTTSTTPKPLLNDSESTEKPIITTKPTKRTKSKFETSLAALLFGEDEEDAVEADEAAEQTQARQEPTKAMMSGPRSIPRMGPRSLKI*

>Woma_00005390

MPKFHFLWALLALIMFIEPTWCYREISGLLDEAKGFSQEEINALRPLFAELQLQYDFVISQTANHLPTANEDETFKQLYENTLQDFEQQLNQMFSQEANTLYDTTLPSVEDIMGKLDLSLMSKEQQMEFLDLQILLQDTLDEAQLNLNELVMRALLIESNLLKLNKPKIVMFIIKALKMGYKVAARVGRAAYCTYSHLPQLNSSLHSLYEGVDCYLYTTTLIVRIKNETYQTVKQVKKNIYELAGIYKKIVEKNSLIGKIISVILNVTKIARNILDTISTAKHVMDDVENQLPLAVKEAEKCGSNFVVQIPQMIKTVSNVTECILFVDEVKTEYDFMKPEGEYNKTVQRMLLLNVDEKDFSEIDDS*

>Woma_00005297

MFINGRENTFYLFAIILIIAVIKTEHCNGQPFKYETKEFEVPLDHFNFAISKTFKLRYLYNDSYIDKQSSKSPLFFYTGNEGDIESFAQNTGFMWELAKRFRACVVFAEHRYYGKSLPFGNETFNVSQNYGYLSVEQALEDYALLIADLQDKHNYKPVIAFGGSYGGMLSAWFRMKYPHLVYGALAASAPIRQFNGMTQCDIFAKITTSVFATAFQNHTCSENIKKSWDLFKQMVSTEDGKKKLNTEMMFCEPLKTADDLEKFLDYLEDVYGSLAMVNYPYSSDFLSPLPGYPVRQFCSYLQNSETGDALFESMKKAINVYFNFTGSATCLNYNSAFDPNSVGGDAWDIQTCNQMVMPMCNTKDTMFRAKEWNFKKEAEKCKSKFHIEPKLNDITLRYGGQNLQDTSNIIFSNGLLDPWSGGGVLQSDNAKIYIIIIPEGAHHLDLRSSNPKDPASVIQARHTESEIIGKWIEEFHW*

>Woma_00005319

MLKVNLLIIAILLLSAGSFADKKAKKYSKEANEPHFKEVKSETYDPDFRTLQRPFRMAKLNLVWAKAQNRLTEPKLKSLYMELKIHDKEEIAWKQLNSQHKDKDGLKENELRGKLIGIMSSYDLLEHFEETEDKEKVKPYKKFHDPEERHLNKSLFKDKKLNKLWEKAEVSGFTAEELNSLKQEFQHHQDKIDVYYSLLENIGSNVDVNKHENAINEDELENFNMISHDPNENEIETPVSAAKKYENHINEVREHHRGLKDHYDRLERLVSSGPHSQDFIEPKVQGLWRVAQASNFTDKELSSIKSELHHFESRLLKLRHLHAEHALQKEKYKNEKHQDKSNRFEDMEDTIKKQSRKVEKIQENIEKTIFKHTEL*

>Woma_00005333

MLFKFFGFLLIVFYFNESLANSTKVTIGESSVRATVKRKSRVDVVSFEALGEHIALGLDFLMPFIKVPIIRKTDAYGNEPALLNINTAALVSTGLLATTSALISYVFRKYVVLGREDTSSEKIRRSDNDDFENELWSALNNFKLVYRNSSGARVDTSLAGIFNTIDETFQKNDIDLNSCLQKAVCSRIHLSSRRVNEGNPSGVDKIIDGLMGLKWFREFLRRTYFKDSVELLEHASFSNHQYKYNNRSHCDVKYPKCKWSMPEDNLKDIIGTYLKFT*

>Woma_00005416

MQLSIVFSLFCLVATAWANTGPAATPAKPGEKEKQLVCYYDSSSFIKEGLAKLIVDDLEPAFQFCTYIIYGYAGIERDSFKALSTNQNLDLDLGKGLYRVVTRLKRKYPNVKVLLSVGGDKDIETGEDAKDLPNKYLELLENPTGRMRFITTAYALVKTYGFDGLDIAWQFPKNKPKKVHSGLGSFWKGFKKVFTGDHIVDENSEMHKEQFTALMRELKNELRPDNYLLSATVLPNVNSSLFFDVPSLINYVDFVNLAAFDFYTPERNPELADITAPLYPMADRNPEFNADYQVQFWLRNGCPASKINLGIPTYGRPWKLTEDSGETGVPPVAKVENDAPLGVNTQTLGLYSWQETCSLLPNANNMYAKGADAPLTKVADITKRSGVYAFRAADKKGKNGVWVSYEDPDTAADKAGYVKNHNLGGVALYDLSYDDFRGLCTGDKYPILRAVKYRLLH*

>Woma_00005414

MLFFLLLTALVGHISCSSRIIGGELASPGQFPYQVSLQLRGRHHCGGSIISETFILTAAHCTKGQSPSLMRVIVGTNDLQAGNGQSVGVAQFIIHPQYNPQNQDFDMSLIKLSQPLTLGSATINKIELAEPNKVVAADTLATISGFGAINGNLQLSHRLKFAQVQMWSRDYCNPQNIPGVTDRMVCAGHPSGQVSSCQGDSGGPLTVDNKLYGVVSWGFGCGAHGKPAMYSYVGAFRSWIKQNAGV*

>Woma_00005446

MMLKVALLIIMGTAVSEAARIRRDDQPPISTQALLESGLNIAQEGVKKLEEIAGNLINANSTDELLNHGNTILDQVKSLAKTITKTTKEWSDNTSVQELKDIATNALQGFKDEHPDLTTAVSDSIESLNKVANEIILKLKLLVDDKDVKQVSDTLIDTASSGITIVKDQLKETTQALAEQLKKDAAVKDKVTERK*

>Woma_00005461

MLYTYILFLLTVLLLGSRTHSKVQHFTHFTITSEAHIGGRRPIPANPKRVGRFQLFSDSLTKFSDRCVNTVAEADDLPKTEIQVMWVAPEAGSGCVSLSAMVYEGPRSWFSDDGQLTQVICEKKPDAATVHKECCACDEAKYSFVFEGIWSNETHPKDFPFTVWLTHFSDIVGASHDSNFSFWGENHIATDGFKLLAEFGSPSGLEAELRAKGPKLRTLIKAAGLWYPNVNANTSSTFRVDRKHHKVSLVSMFGPSPDWVVGINGEDLCTADCSWKESVDFDLYPWDAGTDSGITYMSPNLETQPRERMYRITTMYPEDPRSPFYNPQSREMTPLAKLYLRREKIISRNCDDDFLKALQVEVSDNSEDQDTRVECRVTEYSDWAPCSVTCGKGIRMRSRQYVNPDKAEKAKCSRQLVSKEMCVADIPECGNQQDEDDEGENLAKSISLVNENGDGAGVCQTTAWSPWSDCSATCGIGITMRTRSFLNHLGRKRCPHISVVEKQKCMKPECTFEQIELPDPLCPTTQWSDWSPCTATCGKGVTIRRRILLLEDDALKENCTKRMELDQQKECTVAQDCTITYDMAKDICVERFDSGPCRGNYMRYAYNKDTGRCETFTYGGCRGNRNNFLTENDCMNTCSILGSSSGSNQITTSRPINTLSLAATQRPRTDQDDGIPEACVMTEWTNWSECSVRCGLGYSERYRYVISEPKNGGQPCPKRTVKRRRCTMTDC*

>Woma_00005465

MFSRKFVIICIVISIQTPCKATKKRYALEYYTTNCSKYSETLTRFECVFNSPTKGVYLFSTRLMLSKTMDQDFYMRFYIEAASKRKKPVKFFDIKTKACDVLQNDFGNYIIKEIMRELRKTGNYPYECPFKKNFLYSVNNITYTDKNLPVSLPFLKFTFGIELFEKVELIGKMMVTGAIVPSK*

>Woma_00005469

MFSRIFLITCILISIQNSGQAAKKRFAMEYYTTNCSKYSKTLTCFECELKNPTKGVYLIFVRLMLSKTMDRDFNVRFYIDTTFKDKKPVKFIDIKIKACDALQNDYGNYIILQIMRDLRKMGNFPYECPFKKNVLYSVNNITFNEKSLAASLPFLKFTFVVELFEKEKLMGKMMVTGAIVPNN*

>Woma_00005403

MKFCAKTVSIKLLFVALLLLPATTLSWPHDANSHQLHHGHNTHHDNEHNASVDALSSSSSSTSASSTKGVHHNDWHRLEMMDANGLYLLEWWTKAKDIYFRVTVNTQGFIGLGFSRKSGRMSGADMVLLWVDDRTAKANALDCHGSFTQQNAAPIQDDTQNYIVLNGFQNATHTQVEFYRQIETCDPYDIPLGTDTIKVLWSFGDTDPIHGNLKGHGKNRGSKPLHLLGPMFRKPHGLNRNRQDIQKWDVTVQNVTIDASMDTLYWCKIIKAPDLMDKHHIIGYEALLTRDSDDTQPLVHHMTLFECSTKSYPGSDPASWDVWVKSSGAVCNSNLLTPRDWDACITPVAVWSIGSSGQFLPPHVGIPVGGKKGAKYYMLEIHYDNPLALKSSDHSGFRIHYTKHLRKHDGGVMISGVSVSDTQLIPPGQKLYRNVGICGPSCSNEMFPKDGIKIISGTLHSHRAGRKMALRHVRNGKELSRIIEDDNYDYNYQQVRQLENETVVLPGDYIITDCAYETSSRKRPTFGGYSTKQEMCLSFITYYPRIDLAGCYSMTPVREFFETFGVYQFYSLNMTDVENLFLYNGNVLDYIPNTIYAKTKPNKANMTEEDILYEESLLNKLIISDPVEFHDRTFLSHLNQLPWSEPLFTKRVEQSMINGKHMTFCRVSNDAISIPSEIIRYPNFTTFVKPPSICPYQMLMDSAPLSSASVILNFKANNLTAQLKLYKKRKVIHCK*

>Woma_00005426

MFYFKIPFLLTTTFAVYANSDTIAANLQTNHNYELSSSSLNSQHNEFTDNFYNAKDDNNSNSNNNYDDNVYNESNNNDNSKHNTKNSNYGGGSDDNDYNKSVANIKKETTTARTKKKPPHIIFILADDLGFNDLSFRGSSQIPTPNIDALAFSGIILNRYYVNPICTPSRSALMTGKYPIHTGMQHTVLFGAEPRGLPLNEKIMPQYFNELGYSSHIAGKWHLGHYKRVYTPLFRGFHSHVGFWTGHHDYFDHTAEESHEWGLDLRNGLDVAYNLHGKYTTDLVTDESLRVIANHNPSKPLFLYVAHAAVHSGNPYNPLPAPDDAVSRMQHIDNFNRRKYAAMVTKLDESVGLIVQQLQKQKMLNDSIIIFSTDNGGPAEGFNLNHASNWPLRGVKNTLWEGGIRGAGLIWSPLLKNTRRVADQTIHIADWIPTLLTAIRGEKPDIQNSSTSLDGISIWHALSNDEISPRKSTLHNIDDIWGSSAMTRGDWKVVKGTNYNGAWDGWYGPAGDRDASSYDYKAIQNCYTGRALQALNMLPTTADITRMRSESNIDCTVRMTYLKKGAVCKPLERPCLFNVKDDPCERYNLAQKYPNILNALLEELDYFNSTVVPPSNQPLDPRAKPSRWNYTWTNFGDYI*

>Woma_00005425

MFKLVLVCLVLTTVVVLARPEPPVNTYLPPSDTYGAPGSSGGGGGSGGGSGSGSGSPLAPSDTYGAPGFSSGGGGGSPSSTYGAPGIGGGAGGGGAGGRPSSTYGAPGSGSNGNGGGKPSSTYGAPGFGSGGGGGGGSGSAGGRPSSTYGAPGGNGNGGGRPSSTYGTPGFGGSSNGNGGGNGGGRPSSTYGAPGFGGGNGNGGGSGGGRPSSTYGAPGAGGSNGNGGSRPSSTYGAPGSGGGSGSGSGRPSDSYGPPASGSAGRNGGGGSRGGQPNQEYLPPNQGFGGGGNGGARSGGPGGNDGYDYSQNGNGGGGGGRGGGSGSGSGGYNGDDGSNEPAKYEFSYQVDDPPSGLSFGHSEMRDGDFTTGQYNVLLPDGRKQVVEYEADQEGYRPQVRYEGDAIEGGQGGGAGGAGGAGGADLGANGYSSGRPNGGGNGGGGNGYSSGGRNGGGGSNGGYSGGRPGGQDLGANGYSSGAPGGGGNGGNGGYSNGQGGGAGGRRNGGGGAGGQFNNGQGYSSGRPNGGGSGGSGGGRGNGNGGGGYRNGNGGRNGGSGSGGGNGGGNGGYNYNQQGSNGFGAGGQNGDNDGSGYRY*

>Woma_00005396

MFAFYIVLILTNCIGWNALGGGSGTVLASSLNKPKEKSSFLLHDGEKMTEKHYATAFSGSVSDETTTTTSKPAATSSFTSSASSKQDYLLHPLGSGVSGIAAAAATGSASSRFNVDLRRSSVSSTGAIQSPTTFIGGNGGAGAGAGVGGIGHHHTQQQHQIRRKPQHLSPLSSSSSSLSAAKSAFDEKSGKIGWWIVNCVVNSI*

>Woma_00007485

MLESLKLCLILWLISAHLCYGNEAETNMANHKLSGLIQWRYEKRPFCNAFTGCGKKRTSSHPPYPIIKRAELDDKSAYNSNDYISEGLSDLIDINAEPAVENVQKQIMSQAKIYEAIKEASKEIFRQKNKQRLHNLQQNQLLKDEQAKLEMDNDNDNV*

>Woma_00007483

MFKIFLIFLIINGLITKNNLFIKANECALNTECKHLRDCPLILHSIHLIRYKPYCNLNQKGTYVCCLKPPEKYAQDKDEDIRSVRECKDIQKYRPSCEKSLIVSGKNAEPKEFPFMALLFYKDESTVNYLCGGTLISNKYVLTAAHCFFVYSPPNTVRLGELDFSTTTDDASPIDIEIKNSITHPQYFTTDIQEKYNDIALVELAKEVTFNDYIMPACLPLVDGRDFNKYYAAGWGRPNDTTTELTPHLQKVKLDKFDDATCISKVEQTEELKDGVNKRTQMCAGSFNDKRDTCMGDSGGPLFIDHPDYKCLYLVLGITSFSHGGCGNVGHPAVYTRIQLYLEWIERIVWNEAVRLS*

>Woma_00007482

MIIFLLSGIILLQFLSISAIPCDSCGNECASACGTKHFRTCCFNYLRKRTDPNAMKMVTNKRLIDFIMLEGKAMFTQDEMSHDKKQQQQRQQKQQLYEHQQKLLLPRTTFPFNDEERRNGTFILEDLQAYYD*

>Woma_00007506

MFHFKISSNLFYLLAILFIIKFCAAELEECDSLIIEGSSSSLKKIFTDFNAIKDTMNEIKSRMEECPSTAEKINPQDQPLFDIRYDALPKKCVTEFLPKNCAEATSCLRRSGIYKILPHNTFEPFYVECDATTENGDWIVIQRRQDGSEDFYRNFAAYEEGFGDVDSEFFIGLKKLYALTNYNGPQELLIVMENANNKSFFAKYNTFDIGNDTEVYKLKKLGKYSGTAGDSLTQHLGMKFTTKDRDGDISNPSNCAVKYSGAWWYKSCHHSNLNGKYGDTTYGKGVVWYTLTGNNSSLKYVKMMIRRRRTVS*

>Woma_00007507

MFHFKISSNLFYLLAILFIIKFCAAELEEHDSLIIEGISNSLKKIFTDFKTMKDTMNEIKSRMEEYPNTAENINPQDQPLFDIRYDALPKKCVTEFLPKNCAEATSCLRRSGIYKILPHNTFEPFYVECDATTKNGDWIVIQRRQDGSEDFYRNFADYEEGFGDVDSEFFIGLKKLYALTNYNGPQELLIVMEHANNTRAFAKYNTFVIGNDTEVYKLKKLDKYSGTAGDSLTQHLGMKFATKDRDNSDNCAVRYTGAWWYKSCHHSNLNGKYGDTTEGKGVNWFAFTGNNSSLKYVKIMIRRRRSAS*

>Woma_00007504

MFHFKISSNLFYLLAILFIIKFCAAELEEHDSLIIEGISNSLKKIFTEFKTMKDTMNEMKSRMEEYPNTAENINPQDQPLFDIRYDALPKNCVTEFLPKNCAEATFCLQRSGVYKILPQNTFEPFYVECDATTKNGDWIVIQRRQDGSEDFHRNFADYEEGFGDIDSEFFIGLKKLYALTNYNGPQELLIIMEHANNTRAFAKYNTFAIGDDTEVYKLKKLGKYSGTAGDSLTQHLGMKFTTKDRDGDMFNDSNCAVRYTGAWWYKLCHHSNLNGKYGDTAYGKGVNWFAFTGYYSSLKYVKMMIRRRRA*

>Woma_00007505

MFHFKISSNLFYLLAILFIIKFCAAELEECDSLIIEGSSSSLKKIFTDFNAIKDTMNEIKSRMEEFPNTAENINPQDQPLFDIRYDALPKKCVTEYLPKNCAEATFCLRRSGIYKILPQNTFEPFYVECDATTENGDWIVIQRRQDGSEDFHRNFTDYEAGFGDIDFEFFIGLKKLYALTNYNGPQELLIVMEHSKNTRVFAKYNTFDIGNDTEVYKLKKLGKYSGTAGDSLTQHLGMKFTTKDHDDDISNPSNCAVKYSGAWWHKLCHHSNLNGKYGDKTEGKGVIWYTLTGNNSSLKYVKMMIRRRRSVS*

>Woma_00007503

MFNFKMSSNLVSLLAILFIIKFCAAELEECDKLIVEGSSKSLKKIFTDFNEIKDAINEINSRMQECPNSAEKINPPDQPLFDIRYDGLPKKCVTEFLPKNCAEATFCLRRSGIYKIQPHNTLEPFYVECDAITENGDWIVIQRRQDGSEDFYHDFADYEEGFGDVDAEFFIGLKKLYALTNYNGPQELLIVMEHANNTRAFAKYNTFAIGNDTEVYKLKKLGRYSGTAGGSLSPHLGMKFTTKDRDNDIEQTKNCAELYIGAWWYKSCHASNLNGKYGATTYGKGVIWWTFTGHNSSLKYVKMMIRRRRSAS*

>Woma_00007508

MNLNIFSFISICFIVGLKCEEIDALTYVDEIDILKNIFDKLNELKNELALCNSRFEEYKNNNETSTDFSIFDIRYDTLPKKCTSNTLPKSCAEATACTKRSGIYKILLEKFSSEPFLVECDVKVDGGGWIIIQRRHDGSVDFYRDWAEYRKGFGEIDGEFFIGLDKLHALTNYNGPQELLIILEDGGVIKHAKYSNFVVGGESELYALKHLGVYSGNAGDSLQSHLGMKFSTKDRDNDKYDGNCAESYTGAWWYGKCHMSNLNGKYGDTSYAKGITWQTFRGYNVSVKHVKMMIRRRRFERD*

>Woma_00007509

MIKTNSINIFYSILLGILLIQYTLGKPIDDDKIKNSSQTIGPLDDDKINNSNQTIAPLIKRTHLFDDNSTEYYVNRTVNTTRHILEDPTVIANHNMEVEEFKLNLTTIMNNYEQLRKVVETNGGIKNDTATEMAYLDKVVRRFLNITEYYFELPEKRMTTDSKFIVDILKKYGGYDIYIELKIVGEFIAHIFARALSEALFGWIKPITDRT*

>Woma_00007459

MNSFSVFLVFALAALVVAEPPSGYNYQRGGGGGGGGGFGGGFGGGFGGGLGGGGGGYQAVSGGFQTNEGQNVDPQLLEQVRQILLQEESKSGGGGGGGGFGGGYPGTPSGSYGAPSPQYGVPSFGGGGGGRVVGIDLEGIRQAIQVAQFAQQSTQAGGGGGGGFGGYPSAPSGSYGAPSRPSGSYGAPF*

>Woma_00007431

MKYKMNYLILLAIFAVLCRLPEAFGAKTNFVTCGSIIKLLNSDYGMRLHSHDVKYGSGSGQQSVTGIELKEDVNSHWIVKAPTNKFCDRGEPIKCGDTIRLEHLSTKRNLHSHFFSSPLSGEQEVSAYGDDGVGDSGDHWELICSNNEWLRDAHVRLRHTDTGAYLSMSGRSFGRPISGQMEVVGVGNPSHGTRWTTAEGLFIVPKEKETEYSHTEL*

>Woma_00007436

MWFLVSFCVCAIGFALAGRKWGRWLYLRYGRFILPRSMLAQKNRAPTPPLSPSVVKNDETLEGLLDESGEMHETEINHASRKEILLSNIYHSSFYENNV*

>Woma_00007366

MFKNILKISVVFCLIAFAYAGLKTQVATHLSDEYLDKLNTTPFVRVGNKLYHFGQSKVTWFKAFLVCRLIGGVLASFDSQEELTELSNYLIANYPIDRWWWLSGSDLDNKGDYYWYGTGKSFGYVDWSSGQPDNAGREEHCVHLWLKTTKYQMNDWKCDRQAYFICEADTPKTVVISVF*

>Woma_00007367

MFKNILKISVVFCLIAFAYAGLKTQVATHLSDEYSDKLNTTPFVRVGNKLYHFGQSKVTWFKAFLVCRLIGGFLASFDSQEELTELSNYLIANYPIDRWWWLSGSDLDNKGDYYWYSTGESFGYVDWSSGQPDNAGRDEHCVHLWLKTTKYQMNDWKCDRQAYFICEADTPKTVVISVF*

>Woma_00007510

MFVPKIILNLFNLVVILSVSNLSSAQFQECDKLTIEGSSNALKKIFMDFNEIKSTIAEIKSSIEEYSKRPEIAHKDQPLFDIRYDILPQKCVREVLPKNCAEATSCVRRSGIYKIQVAEYSTEPFYVECDAETENGDWLLIQRRQDGSEDFYRNWEEYEKGFGDVDKEFFIGLKKLYALTNFNGPQELLIIMEDGNDTRASAKYDSFSIDNNMEAYKLKSLGKYSGTAGDSLQVHLGMKFTTKDRDNDTHQTLNCAVNFTGAWWYKGCHHSNLNGKYGDNTFGKGLNWLAYRGHTTSLKYVKMMIRRRRINS*

>Woma_00007448

MLKCHHVICFFFTFLLTIRTRAEQKKERKINPLKNTTIIFKFNTSKCENNITILMSITLLRGGKQKTKSCVGMTVMSKTSIAEICENAKLARDMALTGNYDSASIYYEGLQGLLARMISSTNDPLRKGKWTMVKQQITKEYSQIKEIQRTLSEMTLDLQSSQFASKLRTQTSETETKDPSSWFRPDPDIWTPPPKDPDVWGPPRPEKPAVPNRNRNVAGKKSLPTKSTIPTRNSASSNGFNKKSNTATASSSARSTNSRSSTTNRRSTAASSGNAANNKDDNSSNKDNDEARDEESPEQEEKEEKKFQPNNHIEAELVDILERDILQRNPKIRWNDIADLHDAKRLLEEAVVLPMLMPDYFKGIRRPWKGVLMVGPPGTGKTMLAKAVATECCTTFFNVSSSTLTSKYRGESEKMVRLLFEMARFYAPSTIFIDEIDSLCSRRGSESEHEASRRVKSELLVQMDGVGSEEANKVVMVLAATNFPWDIDEALRRRLEKRIYIPLPSDEGREALLKINLREVKVDDSVDLGDIAKKLEGYSGADITNVCRDASMMSMRRRIAGLTPEEIRQLAKEEVDLPVSNQDFIEAISRCNKSVSQADLDKYEKWMAEFGSS*

>Woma_00007442

MRFYAVSATVALLLIISAAGQDATAAEQEYETQTIQPVSEEPVKYVHVLNPGEREYLSPNLIGVQNIAMTFLPLSMNFINIVDAFREIVNGVRYEIILNAVNTEDNNADIVCRLVILEKPWLRTEWGDKVRELQHSNCTAEADDGSIANTTPLPSNIVNDKYVKSTVFNGGTRNELTDDDMKRLEDQIFSSAISYKRMPTHKFVTPMTSTTTTAKSLTAADEQGEVDTATIATTTLENNDYSNGQEEDDKTITITTPSYSVNDEETTVANNEKTIEEEREQNPTELPQYQITTSTTGVPELTADEMKWLDDIFSVGALNFENNLNSVPTGGVGGGGENGEPDNLQQQEQIEEPFVRRIKRSLKNDIVGGVEKLSNEDAEKKLQNSLDKLTTGEGPSYKISKIHAATKQTVSGSLTKIDCDLIDADGKIERCEVQIWARNWIKDGQEVTINCPNKELVKRRHSRSLEHVEKKSHKKQNHHSLNKVEHLFSKFQIKYNRRYHTSMERQMRLRIFKQNLEIIKQLNENEMGSAKYGITEFADLTSTEYKRRTGLWQRDPVKAASTPKALIPDIELPKEFDWRSKGAVSPVKNQGSCGSCWAFSVTGNLEGLHAVKTGKLEEYSEQELLDCDTTDSACNGGLPDNAYDAIEKIGGLELESDYPYHARKEQCHFNKTKIHVKVKGHVDLPKNETAMAQWLIANGPISIGINANAMQFYRGGVSHPIHVLCSHKNLDHGVLIVGYGVSEYPSFKKTLPYWIVKNSWGSKWGEQGYYRVYRGDNTCGVSEMASSAVLDI*

>Woma_00007420

MRKEDYLQILQANLPNFMDKCAYPEKEIDFQQDGHPKHTAKIVKEWIGKQNFQLMEWPAQSPDLNLIENLWSVVKRQLVQYEAAPTNTSNLCDRVNAEWNRIPKNVIENLVESMPKRINQVISNKGLWTKY*

>Woma_00007422

MHFSVKILLMLLSLAKIISSLEIDYCDRELCPYQGIHEACNKTLSFGKNCKGKMKVEDMKPYTELILEEHNYYRNLVASGKVKCFYPAVRMPLMVCSAVILLKKWDWNLAATAEHNIRSCVFAHDECRSTTEFSLAGQNICTARTRRLNVTVESFITEAIFLWFKEQFHADHELLMSYHKTKPQTGHFTLLRMVIFTCNYSSTNIRGMPTYKAGIAASKCKKRNNKFKALCEDPIDPNDLSWYNR*

>Woma_00007424

MLLKTDGSRPIMGSKFFCLLLITLTAAQNAVAYAPISKESIFADVLLTELLNRMDKENDPLSNYFDMDTDLSKGLDMVSRSSSQNIPEDYEYRNVFPGGPNPSIRDQEFLQHSSLWGHQYVSGGMGEAPNHFPTLIKTDASLPAYCNPPNPCPYGYEENEGCISKFENTALFSREYQAAQDCTCDSEHMFDCAGQDTSSGNSEMASTMEKFLMHQFQNDNGIDNSNAPVKKLYGFAVAPAAAVAVNPFLEGDKLPIAAKKGNHVKV*

>Woma_00007409

MSGLNASAPTVLSGLTSVGPTIVSGLFSSSSNTTWPSASFLNSLGNEPLSINTTSPARIVKIRTSVVQIPHSLQLIQVAKPLSLAQPASLMDTTTRKD*

>Woma_00005497

MKSFTAVAVIALAICALVSSKHIESKVADKDFLVKQKFMLDILQHVYQDDVFVKKYDESYVNYKPWEHLEDYHDVDMVKSFFELWQHKPMHDDEIFSPVYLRHAEYAKGLVHVFYFAKDWNSFTHAVYWARTHVNKQLFVYALTVASIHRDDLRGIVLPAIYEIHPWHFFDVDTLKLAEKYRMHGFHNVKKLDNLYNVVVKSNYSNVYGDVNYEHNLAYFLEDIGYNAFYYYFNVDYPHWAKGPVGHELNKDRRGEFYLFLHWQLLARYYLERLSNDLGEIPYFNVYQPYEHGYFSNLHYYKGVSFPDRDNHYNFYYEDNYEYAKDLEYYGSRIAHYVDTTHEDYKVAVNKLGNMLQGNHDSVDYHKYGALDLIYRELVNEGRPYGKYGETLPAVLMHYETSVRDPLFYSVYKDIVSYYWRLMETYPEYKPQDLVFPGVKIDSVHMPEGLTTYFEYFDADISNAVNVEIPNTEGPVDGLYKFGRNSQVHGQSFVIKARQLRLNHKPFEYTLDVNSDKSQKAVVKVFIGPKYDENGHLIHLEDNYMNFFELDHYVVDLAPGVNHLKRNSNDFTWYVSDRTTYLELYQKVMDATNSDYKFPLNQDEAHCGVPQRIMLPKGKKGGMPFQFFFIVYPYHAPEVHQYSTYDPVISCGIGSGSRFVDDLPFGFPFNRPVKHDYYFNVDNIKFYDIKIYHKDDHTNVV*

>Woma_00005494

MDLSRRFGLPPLILFITLLLCIQSSQSRFFGERLKRQNGANMYLSASSIVPGGEGDDPNEWSEWSSPSECSRSCGGGVSFQTRECLRTASNGAPICKGGNRKYFSCNTQDCPEDEPDFRAQQCSRFNHQAFDGIFYEWVPYTNAPNPCELNCMPKGERFYYRHKAKVIDGTRCNDQGLDVCVDGQCQPVGCDMMLGSDAKEDNCRKCGGDGRTCRTISDKFTTNNLAAGYNDILLVPSGATNIRIQETAPSNNYLSCRNLSSHYYLNGNWRIDFPRPMFFAGSWWNYQRKPTGFAAPDHLSCRGPITESIFIVMLVQDKNVSIEYEYSIPQSESSSTPDVYTWTHMEYGPCSASCGGGIQTREVTCNNRFNLEQVDPSFCDEKAKPAESQACASEPCAPHWISSDWGKCSKGCGSDGIQNRTVTCERISPTGERTVEDDAVCLEEVGNKPANQQECNRDVENCPKYHLGPWSPCDKLCGEGKQRRKVTCYIEENGRKKVLSDEECIEDKPETEKTCMLTPCEGVDWIISQWSGCDACGQTMETRTAICASKSGKVYDDEFCAPETPLLSRPCESNKCKTQWFTSEWSKCSSSCGKGIQSRIVLCGEFNGKIISKVTDDSQCDAATKPEAEQECEGKEKECPGEWFTGPWGPCSKSCGGGERQREVLCLANGTKSDSCDESKIESLSEKCNAQACTEDEILPVDSTDKPVTDEEYEDDCEEDEEEEEDYIKLVTDKMSEKLKISDGIDLDEESTMSSLMTDDLMLSDSTPAIETTDDEISTDLPLSTVEGSGDQTESTELSSVVTEGSGDDIESTSDSSFSTDKSEATDDTTESSKSTGTSTSDVITESTLSTAISEATEKTETYDHTDISTDTSESSSTEESTYSSFAVSDITETTDHSTVSSSTVATDSPQSTEISASTENFESSTTDTSISTDKSEYTESTDSATDITTDITTETTETTETSLFTDISTESKYSESTFTTEQTPSKDTTDTSESTSDSTDSSLSTSDSTESSVSTSDSTESSVSTSDSTESSVSTSDSTESSVSTSDSTESSVSTSDSTESSVSTSDSTESSVSTSDSTESSVSTESSASTEPSISTDSTTEFSTEPSGSTDSTADISTQSSISTSSEISPTVDSSTSTIDSSISTEITSSTEVSTSTDATISTDESTSSDGSTSTDSSLSTEFSSTATTTDDSSSSSDTSPSTDASISTEISTEYSDTTQSLPTDSTESSLSTDMSKSTDKFSDSTDSSTISVTDIDSSISTDSEISVSTDAGTSVSNDSSTEGDNESTDVTTESGSTVDAISGETTDETESESTGKTTSVFDSTSKATEEDFSTSSDIFDSTTDSSSDSSLSADEFTTIDIWTTEDELEKSTPNTLEAVVTKETKPKKCKAKKPKDCRGTAYGCCPDGKHSAKGPFDEGCPIAKTCAETEFGCCHDGVSPASGKNFNGCPKSQCAETLFGCCSDKFTPAEGEDNEGCPEPTTLPPTSTTEISEETTEVYETESSGMPESTDTSDVSDVPETTEAVLPEATEKSCSFSEFGCCPDGKNIASGKNFAGCDDVIDPNNCRNSLFGCCNDGRTSATGPDGEGCPACTFEPFGCCPDNETPAHGPLGEGCCLNSVYGCCPDNINSARGPNFEDCDCKYAPYGCCPDNKTSARGPNGEGCGCETSEFGCCPDKLTAAQGPKFLGCPCHTLQFGCCPDGITVAQGPHHYGCHCSQTEFKCCPDEKTPAKGPENEGCTCLESKYGCCPDGVTSAQGDKFEGCENVKEPPQLSCKLPKETGPCGNFSIKYFFDTSYGACAKFWYGGCDGNGNRFDTESECKETCQEFKGKEACLLPKSQGPCTGYITKWYFDAERGRCEDFKYGGCYGTNNRFDSLQECQSLCTVSDSIPPCEQPMDQGPCEGRFERWFYDNVTDVCRPFTYGGCKGNKNNYPTEHACNYHCRQPGVHKDYCSLPKKIGDCSEKHPRWYYSEEEKKCMPFYYSGCGGNKNNFPSLETCEDNCPKEVVKDICEIPAEVGECTNYQPMWYYDTKDQRCRQFYYGGCGGNENKFSSEAACLQRCEKKPEPEPEPEPEPQLKREPEPEPEPESISESEKPHVSVNVCEEKPDPGSCSNYTLYWYFDKEQSSCRQFYYGGCEGNGNKFVNEDECKHQCIGDSEPEPGAALVPPEVPSEQNICLLSHDTGNCDNYTEMWYFDTATSSCSMFYYGGCGGNGNRFASEEECNYRCGQEPRTNVKGSPETEDSRKCFLPVETGNCLDNEVRWYYNSGDGVCDQFVYTGCGGNANSYATEAECEDECFPAHETCVLPPLRGNCNESLIRWHFNEETGSCNEFEFTGCRGNRNNFVTEQECLSRCGNGEPSAPSYSVCDQPLDAGECENSTTAWYYDNENMICVAFTYTGCGGNGNRFQSREQCERQCGEFKGVDVCNEDVSSGPCRQWETRYFFNKQTRTCEPFTYGGCQGTGNRFENRGECESVCIIGQEPTYSHNKDICKQQVDVGRCNGPSVTERRWYYDDARGNCISFIYSGCAGNQNNFRSYESCYNFCSKPDITNEVLPNRCETYENECNSITCPYGIQKVPVDEECHRCECNNPCKDYNCADGQQCAIEVSSEVSGQFVPVCRLINKPGICPQLRADDGVCGRECYTDADCRSDNKCCSNGCGFVCVRPTPPTIRTTAPTTVAPVVVYPGEVRASLIPKQKPELDVQTPMGGIAVLRCFATGNPAPNVTWSRNNVVVDTNQGRYVLTSSGDLTIVQVRQTDSGSYVCVASNGLGEPVRREIELHVTEPVNIPAYVYGDRNVTQVVTLNRPAVVRCPAGGHPAPMVHWWRNRSRLPLISQRFELARDYSLTFRSVQLSDLGPYTCEAWNRLSRRPASIKVTLVAVGPARATTNHDAQYLQYIIEPPKAPVTQRPSFPYRPTRPPAVPPPYVVPSLPAVPVSAVIGMDPNNQYTPGSTIAIGCSIQGYPKPNVTWIKDGLPLLPSERVQISVGEPHRLIINDISTADSGRYGCKAANAVSYSISEESVNIESTIPLNPECIDNEHFANCKLIVKGRYCKNKYYARFCCRSCALAGQL*

>Woma_00005495

MPFLEDSGIDSEDKTSLLIEESEPHFNRMRNDNAEHMADLEVTFSGATPIITELQPTSLKTSDSSPVHISGDTEDTVRPPNTCRILQRKLELKVERAKRNYSHYQETNKRKSQSAILIPISRLHIPGDQERKPLVDYKSDSDECEDISFFPEQKHKERTKRHAELSDTFSIQEMTIDSDLESNDSQNLELLTPRIKRNFLEYLATCFCFQPSS*

>Woma_00005490

MQPPSVVNHQCILIHRSNDVNDSTEVSEDDPTTSQQQEPPLSQTPDSSSEYEPPGQISPTYQKPETIQLPNTNRTSSSERKAPRYLEEYILD*

>Woma_00005546

MIFSSVIQILCIFSLIIVVKTFPDGAPADTCVKKRPNQPNHGQARSQSLDTNPYEVVANSETFHPGQEVTVVIYPHSKQELFRGFFLQARDADSNEWIGEWQRSENTNTIPECSAITHGDNRDKLGAKLIWKAPQNKRGRVYFTGTVLKSYGKFWSDIVGKVQSRQ*

>Woma_00005599

MKLKFNLICIIIILLGFVFASKAGTFKPFIRSRYSLRWRKTTETPKIWASSTISSNTKEETPAKTLQNLESSTENSILVTTIKNLLKTSTHPPDYDYYNNEVEEAEHKS*

>Woma_00005488

MNPKNGLFWQLALILVATCARLGSGRESQLRIGKALNVFVRFGYLGISMRVIPTNDTNEDVRWLFKEPTRNIYKDVNHLSENQEQNTPGIFHGDFHMEFCDNRRQLYQAYFRDFTIERLDRPWEAFTGGWFPDNAAKKLGINTSFIQGDYSYVLVRVVRFRETGKFKSELPENLTLENDIRERMNEIAIGNVTTAVKFFENVGTHYINSYTTGNSLYQVFVYNRKNYQMIKEQIKTKGINSLSKQDLYHLFAPWVAEHLGQIRSASANYTVERWARRKLQYEYSLEKYVTLLKLHGNGTLLRTLDNLLGNDAILQLDLKSLNVLFRHEPEKQNWFHEVLDNHMKLWEANMPQN*

>Woma_00005639

MKTFVNFLIIVAVLSCNNAYDFDYRYQSKWYDVNDECSFDRQSPIELNSDEAIITTDIPALRFINYNLPLTKPVIVSNNGHTANIELPKPDGYVESLWKIYDRFAPSISGGLLLNGRFVAESVHFHWGSKDTHGSEHVIDNQRYSMEMHILHRHTKYQTVEEAKKHEDGLAVLAVFFHVKPSQLIGFKGLENIINALASIKAFNSSSQITNFTLSELFGDISTAEFYTYQGSLTTPPCSQAVQWIVFPQVIHISQAQMKHFRSLSDNHGEILENNYRHLQSRGTRRVFYSQQRNPIDAIISSPNFWSILDKLKPRPLKVEN*

>Woma_00005637

MKFLLNLLILTGLLAIIKGQDFDHKNQAEWTGQCHLKSQSPIELNRFRAFPAWGLPSLKLKNFNISWNKPAQLWNNGHSADIQLPSDSTAIITGGPLLFSKYATESLHFHWGSRNIFGSEHVVDGERSSMEMHIVHRNTKYATMDEATQHPDGLCVLAVLFNVNPSQLEGFNGLNQIVKFLPAIVQYNSSIALNNLVLVDLLAGINIQNYYTYQGSLTTPPCSEAVTWIVFPGRIPLSAEQIRAFRSLFDMNGNNLENNFRFLQAKGNRKVYLHDGNISGKDDTKDECGGSPDNVCLAQLKDGCEIVMTGA*

>Woma_00005611

MKFYIAICFLFALTTAEFVVKTHNDLLQYRDECVAELDIPETQVAHYKKWQYPNDVITHCYLKCVFTKLDLFDTTKGFNIENIHQQLIGSQAEANHDDILHAKIAACIDKNEHGSNACEWAYRGATCFIKNNLQLVQQSVAPVKA*

>Woma_00005670

MARNFNKQSLKMLAMLFLVWIPLCYAYPSPQSSDDMVHNNNYEMIMPDDHEPAESMHYQNFDEFFNAADGGDAMAEMEMAESHNSAARIRPPQPLSPKERELRQTLEVFDAQREKHNQRMLTKGSARPRKDAHDWDQFDYEMLVNHS*

>Woma_00005577

MLFTIIITILSVIMIPVELTPIVGGDIIKNDGIIKYPYMVSLQERLHKAYNKNSSTTLYLYKHFCGGSLLNLNWILSAGHCLWHKNIQNVFAVIGHENIANVLPHDRLALQRVEFIYFQPTNLQNDIVLLKLQQTYHPPSNWINNMELFGKLPSYGMKAYRKNPCKIIGFGAEKHAGLLQDLLHEAVVYVITNKQCRYLLGHVWAPQKGDNTVCALGSEQDTCQGDSGGPLICQYNGRDYIYGIVSYGLTCGLKGMPSIYTVTGPYIEWINLIINEL*

>Woma_00005693

MLRIVILPLLLFLFSMACMGQTFQYSRGWTNGKRAGGGASSNSFNSLLRKDEDGITDLFEVQEANERRLERCLLQLQRFIHNPLLLHTSANTLTLNPSTTNLAGSIGNGNNNNNKNNPFSRNHQSNEVFEELGAADSGAIIDANDYAKH*

>Woma_00005482

MLAPPIYMGNVTDDLVVSLAPYVDTACGGKTLFPEYCSTLFFLLAGHENRNLNKRSSSCWNFYKSRNSLYTRIC*

>Woma_00005689

MNLNFHNPTTMGLVLLCIWLSLLDGTMSFPSLVMPRLHRSVQAQMETNKNMGFPQTDDDVLQLEQLRDVHPDENVHFSHQKKDKLMTDDTAAYQADESIPELEQQLESAKPDVQSTQGPAAATVENVGETPEIPVVPEVKGSGAHRPPTLQINDGGKVENNGEKPKAKCRLGGMHGAARLVYGQNPEVLPTTTTASTTSATTTTTTTTTTKPAPEANSAGSNEDDDKDEDDDDDGASTDGGGSEDEKSLDTVNEGVDNVEPPVDEPSENQEPAGGDEKPQETPEVPETQEKPADIPATAAPGPSQLQPMKPIKSNKKQNKEEEPERQLIGDVIVDEEAALRKSTSRRSVAHMTPKTAESLLRHGNGQYSSFDMAQYVFWTGDEAGVARAVEELIEQDLMTREEALKFLHEIRVGIEYLQKSYANRIFPEEIRHNTIKKSYLPKPVPATTTTTTSTTTTTVKPTLAYLNLEQNLEDADNINLAKSLDSISLWHKLQALNNAEGRQDITEYESETRRPKIIDCQHKEYNLEEIIYKLARIMFTESLQHGDEAQREIQKLMDFFEREHELGIIPLDLEQKVIHVLLQALSDTLTEKPELWPAAQYPYNRLLRSYAHMNYARPSPTNV*

>Woma_00005476

MLYVTNHGHLNVMGTDLMGQLWSYPIESICYSISILEIQQIKSKFPKVFVRQPGLCSPFKVHLKVKTSATPAFSPKRPVPYAAMKLVDERQRLERANIITPVNYSKKD*

>Woma_00005564

MYSSNSGSGSGSGVVGSSGNYIIQHLSPASSHPLNTNTGISDGGTPSAVATISPSNTPHRAAGGSSSSSSSAIIPTEGRTVPADLPQGLLEGADLLPKYRRDLVGKLRALCTELQALQPQSGHCRLEVSRNENFEERYRLIMKMRSKDMRKRLMVKFKGEEGLDYSGVAREWLHLLLSFKLKENPVGVMQMYK*

>Woma_00005605

MFRTIFLFLCGVLGILSHVPNSHMNPKEVNDKCLIDFPLPSDSLEKFRNRQIIDTDQMRKFLYCIATSLDIYSDGDGFDVDRLCNRLYREPEWDCKKSLVQNCVDLYGEEKLDAGRVLKTLKCIADNENKINENL*

>Woma_00005629

MTVIDLVNRLFLLLLLAYSATALLSEFEWSYDNQDNWGTNSPQCRGEWQSPIHLTISAAPFVPIPAIKFGNYDISLADDITLTNNGNSVEFDVPVTINGQRPFISGGLMKNKFEAVSVHFHWGSAKSKGSEHIMDNRRFDVEMHIVHKNVNYESVDEASQNKDGLAVLGIMFKMVNESDHYYPGFNQIFNKLPNISNYGESVNLDDPLTLGQLLGDLNTKEFYTYSGSLTTPNCAEAVIWSVFPEPLPISQEQIKKFWSIRKKNGEILINNNRLIQDSNNRPIFYRIATRAFGKEYFNGGESIYGSAEYQ*

>Woma_00005601

MKVFFVILILTAVAYADHHEGHGHDHHVHHDNHEYVVKHQEDLVKYRDDCVAKLKISPELLEKYKSWDFPDDADTHCYMKCIFEHFGFFDEHKGFDIHKVHHQLVGEHGTVDHNDETHTKIESCADKNTQGSDACTWAYRGGMCFIRNNLQLVKGSVHKH*

>Woma_00005600

MDMKLLIILVTFMVITTTKCEGKAALRRKPKVYNALITTDEDLVQSRAYPVIQPTIHESGITHLGLYDPYGLYSSHVVRLAQPIAPALTTKQQLPYSLASPQFPPQLMQQPLIPQPQPVAGYPLVQNPPTAPAAEKLQESSEPPTDLEEQPQPTVQPATMPTQPTEIPKEPNQPSKMPSEQLPIPLNQYGLPPALIPLRQPQPYQPFPYPPPQPFHLPPYRFSQFSPLYYNTLPGLRQPFLPPYNYLPTGPQFPLSNTIPTDKAYPSPPVMPPSPLPTTAAPVTPALTEQPTESSLPSEPQPSFEDIKNGSKSKDSNVPDVPPPPIPSGAKSKQS*

>Woma_00005607

MFKLLCFILLTFSVDLIYCQDVSKEYPDFILNNAHARHARLRCLQQYPLADIANFSNTSDTHCYLQCFLYRMGLMDLSTRGLNSKRFMDIWERIDEAFVEDTCMERFYFDEPLTGNCIDCYRKLMEFRNNCLELFDYTFSINSTWKSENTKYSKKLGQSASEFCDSLEGRDNIEAKETSKETDVTLLTQYKDKLKCIFENINYLDAYGRVDESEIILSYQEAQEDSEQIKSIISICSHRANTRYQHNYFGDMVLELQRCLKTKSPVYAKVYKLRDKTSRQY*

>Woma_00005606

MKIFVALLVLVACVYAEEEWSVKNGEQIKEIRTECLKEHPLSEEQKNKMKNFEFPNEEPVRKYLLCTAEKMGIFCSHEGYHADRIAKQFKMDMEEEEVKKLVEECLAKHPKADKPNDVAAYEAHSCFMNTAIGDKVKNYIKKRQEAAAQKTE*

>Woma_00005667

MPHNNIVCEWLRALGLQQYAESFLENGYDELEICKQIGEIDLDAIGVDNAQHRSKLLKSVRTLREKGAALVYVMINDPKALSSSNEILAAECDTPTTMKELESVIKRHLEADGIRLTAHPYSTPEGKRGYLEGLAAHYSKEVNMPYEDVLDAIEGARSAKWRERHMRMATSGSGNTGTLRRGNSSGASGSFGMGVSAMITGNIANQAILPSSHSQPLYVPGKYSPSSCLSNREENEIYSYAQNELVNRMARSVIDSKSSVNLSSGMQHSYPGARTNFFYEFAATEGRNKHKRRTVFARFINGLRQAGDELNPTEGMQLKTSEKIKSCGTMKRVEPHQNFEETIQRLKNQKARKKMQNDKGIHDRNKDITSHAMTTNDMSLNNYTKHP*

>Woma_00005669

MKKLIKLIVFLAALQIHSREYHCDIPEKPFHNEDIRQLLPTVINGDNGKNYEVTVLASRKIFEEEQAASDPLPSRQYLPVHQQTGGNTSPNPRYYGNLQQSLLNNEQNLHVNSQQYANKAAISTVNNYNDQYRFSQFTSNVNNQQQQQQLFSQQQQLLNQQSMPQQQQHPANLLLSTQDQYQHYLQQPQQHNNNLPNSQFLLDALVKQQKYVSQSEAAQLDVYPYKQINGNTRPFKRVITKCDAKGQCEPQTASDDVHGGSDFLHQQVLKQTKAKIEHNSNKCQNKFNYQYTGNALVDNVAKAQRGIPIKKGTDLINRRLIPYTEVVIPQYPHN*

>Woma_00005668

MKFPATASLLQLSILFLWRFTTDAQVQATVGLYGPDRYEGLPEICLMPMDFGHCRAKVQRYYFDIRRMKCTMFYWGGCAGNDNNFKSLDECNNFCGSAYEEQLHNEIVAQKEPTIRETTRGSSVKYNAISSRDSSITKSVPKFALKANVSSKETGYKSQFKASAKSFNVTPKSNTNSNSDSDSNNNSKSNPLDSTEDEDYDD*

>Woma_00005648

MFGGQKLMFGASTTGTGFGGFNNAVISPPFGQSNFSKPATSAFGATSTFGTQQTAPSLFNSNTAQLEAGGSLGGASNSATFGSTTTTQSTFGNFAQQCTAIFCATQPSTSAAAQPCLSFTFTQPVPITSTATASFEQLANAAAPANQGSSIAK*

>Woma_00005643

MWFKFSLLALVALIGSVSCFPESRVVNGSDSDVTRYPFIVSLRGPSGSHSCGSSIIAPHWILTAAHCVSGREANQLSIQYATTNISPNGENVVGVKRIIVHEDYAPSRSYANDIALLELEGSLIYNYKTLAPVTLPDPYFEIPQTPEGVPGVLAGWGLNATGGYSQKHLQEVNLKIYSDEECTARHNGATSGDYHICGGVDEGGKGQCSGDSGGPLLYKGSVQLGIVSWSIKPCTVAPFPGVYTKVSHYIGWINSHIY*

>Woma_00006296

MDIKLNILLLASFVTLLLALASASSSIGHDTEIGLMNFMARSNALLQQNPARSLDCFNFYIPLINQIAQDYETNFKACQNESLAARSKADEATYDQRKDLATAAQNSCDLLSQCSDGESVEGIFQCYIDGGASNIKTMYGINANASQQLADLREQYYLIVAEEYKCTNETKRAYEQKSAAAYASLNSCILGTSEVPTNLPPTAAPEQPTTQPQPEQPTTQPQPEQPTTQPQTEQPTTQPQPEQPTTQPQPEQTTAESSVESSPAEPESSPAALEATTPAAESTGEGGEVSPNEPSIEESSTVAPDNSENYDY*

>Woma_00006290

MKVFVCVVALMAALAPAQATFDKLGFLIGATGVPASYGRSLNSGYGAGYGTGYGYGGGYGSGYGTGYNYGSGYGGGYGAGYGSNAINKVIKVTVIPSSGGYTGGYGGGYAGGYAGGYGGGYNSGAVAYQPAVSTSAVVTPVVTAVSTPLVTPAYTGSIGASYVGGLGYGGGFGGGYGASLPVSYGAGAGYGYGGGFGLGFGQPAGFSDLC*

>Woma_00006224

MYRLSIFPDISFSFFISSNSTGGADAKVDLTHAIGNPDHNLIGNVFAAGNINKGPVATGGTLAYNNNGFAAAIGEEHIPDVRDTVIKSLNADLFNNGVHKVNANVFKSDNTLAYGFKFER*

>Woma_00006277

MKPFASVLLVLVALVACGQAHNIQKRSLGGLLQAKLSLLGGGGGGGGGGGYSGGGYSGGGYSHGGSYGGGYSGGGYGHGGSYGGGYGGGYGGGAAQKTVVVKVVNDGGSYGGGYSDGYGHGGGYGGGYGGGYSGGYGHGGGHGGGYSGGYGHGGGYGGGYSGGYDGGASIVKVIKVIHQNGGSYGGGYGGGYGGGYGGGHGGGWSQGGSSW*

>Woma_00006274

MKIVVVALASLAVVSAGGGGGYLPGGRHVSIGGHTAGGFASASAGAVSGGFGGGYGGSITRHASAGHHGSYAAAPAVLYSAPKVSYGGGYGGYGGARSGGVWAGKSLGFGGGSYGGSAGYGSGGLGSWAGKTAGSYGSGGYGGGYGAGGAGAWAGKSVNVGGAYGSGGSYGGSAGYGTGSYGGLGGAGGWAGKSYGSFGGLSGGYGAGGWAGKSIGGYGGGAGGYGYGAASAASSAAASYSGWWKQ*

>Woma_00006273

MKIFVCLLAVCAVASAGFLGGAGGGGGGFGGGGGGWSGGGGGGGGGWSGGGGGGGGGGVVKIIKVIDGGHVGGGGFGGGGGFGGGDGFGGGGGFGGGGGYGGGHGGGGGEVKIIKLISQGGGGGGFGGGHGGGFGGGHGGGYGGGHGGGYGGGHGGGYGGGHGGGGGVVKIIKVISEGGGGGGGGGGWSGGGGGWSGGGGGW*

>Woma_00006272

MKIFVALLAVASMMATANAGFLGLLKGGGYGGGYGGGYGGGYGGGYGGGHLGGGYGGGHVGGGYGGGHIGGGYGGGYGGGYGGGYGGGHYGGGGYGHGGGGRVEVYKIISTHGGGGGYGGGYGGGYGGGYSGGYGGSYGGGYGGGYGGGHGGWGGWGKKK*

>Woma_00006284

MKILQSLVVFWCIIEGSRVQAIPSGEELFHASKKILFGLQEVEIFKTINQLCSVFLASDIIKPKVTPDMDSIAFILRTPCQELSFPLSKAEQLLKSSEFDASKKAVIFVTGWMSNPNEQYVFDMAQAYHCRGGYNFLALNTSDAIDTLYTWSAFNTKEIGRQLALGLQKLVKKVPAENIHLIGHSLGAHIAGYAGRYFTEYTGLKRGDATFIDVIHTNPGVLGKSDPIGDVDFFAEGLAPIKPGCKIFSCSHRRAYQYYTESVYPGNDENFLGIRCNSLSKLKDSFCTGDSYPMGFAVPNNIMGNYFLEVNSKKPYGKNANAEKEISDECAFQCLAQSRFIFNPGHFIESARSVVTGIGSQVAQKAFSPRDLFRGSRQLLFGLPEVAVFRTLHELCSLYINSGSIDPSITPDISQMYFQLRTPCNSLSVPIEQSELIFEAAEFDVEKPVVLFISGWTSTIESDSIQHMAKAYNCRGGHNFLALNASYFIDTLYTWSAFNTEEVGRIVGQGLVKLHARIAAKNFHLIGFSLGAHIAGYAARYFEEMTGEKIQRITGLDPANPCFGEGQSLEGLHRGDALFVDIVHTNPGAFGKRDALGDIDFYVQGFAPIKPGCMAFGCSHARAWQYYAESVYPGYEDNFLATRCSSLRKAENGLCNESTILTMGYAAPQGEKGTFMVSVNAQVPYGENASEDGELILNECVIFINGKCVLRPSHLYIAH*

>Woma_00006295

MNSKQNLFVALIAFLGISMSIAIGEHDLSWAHKENNLSLMSLMSRSNAIMQRDPTRSLECFGYYIPLINRIAEEYQKNFNACLKTSKESREQVEADTLDKRNDLAGRASTSCELLSQCTDVETAEEVFECYVNGGSENAKSMYTINADASEHLAELRENLRLIDVVEYKCTNETKRTYEKDSADAYKDFNDCVLGIIEVPTDPPATNPPATDAPSTDPPATDAPATDPPATDAPATDPPATDAPATDPPATDVPESSEVAPEDKLPQEDIDMLRRTLKSRLQRLRF*

>Woma_00006259

MKVQLYCALAVFILILTINNGVCAFTLKLTKILCHVHDKTFATFEKCLVKAESRTLQYVVLYIKFLKLPITDIKVRFTFLKRGNGYKPFLYDFIFKCDFLKYMNPIKTLVWDWFKDTSNLNHSCPLVEDFEIKRIENRYVHGKLQALPLPPGEYCFVTNFSAYGEYKFDIQFFAKISD*

>Woma_00006258

MTTNKISRLFLLLVLSALASSQELVEPFSPAINCNGKVAVCVGPLIYKRCVKAGDGEYYASGNAIPCTANSRCVNDGVEICVKLKTSTDAPVTEAYVNSTTSAAIAGLPTEGTLTTSNSTVYSSSAPTIEPSPLPVETTSAPVLATLAPELPVETTNVPAEITTDPNLPWETLVGSSDPVHTTVSSVNPTTNPVNPTDPVATNTPIDMTLSPVEQDVTVNPTSSVVEPVNPTLGPVDPTSGFVDPNATADPTSTTIDRTSGPADQTTIVASTSTPIAPTSGLVDPTDPVATNTPTDTTLSPVQPIEPIDPTPSPIDPEIPNTTIDSTSVPVDSTSVPVNPTSGSAVPTSVPEDPNATLAPTSAHSTINPTTGTIDPVDPTAPVDSNATVDPNVPVDPNAPVDPNAPVDPNAPVDPNAPVNPNAPVDPNAPVDTNAPVDPKAPVDPNAPVDPNLPVNPTAPVDSNAPVDSNATVEPNVPVDPNAPVDSNAPADPNTPIDPNAPVYPNAPVDSNAPVDPHAPVNPNTTVNPNAPLDPNAPINPNTSVDPEAPVDPNAPVDPNAPVDPNAPVDPNAPVNPNAPVDPNVPVDTNAPVDPNAPVDPNAPVDPNVPVNPTAPVDSNAPVDYNATVEPNVPVDPNAPVDPNAAAYPNAPIDPNAPVDPNASVDPNAPIDPNAPVNPNAPVDPNAPVDPNAPVNPNVPVVPNAPVDPNAPADPNAPGDPNATVDPNVPVDPNATMDPSATVDPNAPVDPNAPIDANAPADPNAPVDPNVPVDPNAPVDPNAPVDPNAPVDPNASLAPNAPVDPNAPVDPKAPVDPNAPVDPNASVDPNAPLNPTAPVNPNAPVDPNAPVNPNVPVVPNAPVDPNAPAVPNAPGDPNAPVEPNAPVDSNAPIDSNAPIDPNAPVGSNAPVEPNAPVDPNAPVYPNAPVDPNAPLHPNAPVDPNAPGNPNAPVDPNAPLDPNAPVNPNAPVDPNVPVNPNATVVPNAPVNPNSPVDPNAPIDSNAPVDPNAPADPNAPVNPNAPVEPNAPVDSNAPIDPNAPIDPNAPVGSNAPVEPNAPVDPNAPVDPNAPVDPNAPLHPNAPVDPNAPGNPNAPVDPNAPLDPNAPVNPNAPVDPNAPVDPNAPVDPNAPVASNSPVDPNAPVDPNAPVDPNAPVDSNSPVDPNAPVDPHAPGITPVDPNSAHHRPNGPSNTDGPNSEDGIHSNGHNKPDRPGRPGRPNKPEKPETAEAESTESKEPVKEKPVSNEDISLEDKSKERKEKEEKEKERKEKEKKEKEKKKEEEKERKEKEKEEKEKKEKEEKEKERESERKNREKKDSSEKSDEKDVKKQEEEARKELIKKIVKTVKSHKCDEDDIYPDVRDCKKYYRCIEKSEKSKFVHLHCDDDKRFDFKENKCVRKNKAHCLLKDD*

>Woma_00006301

MDEDDNNSANEQQLQEEEEEEEEEDVEDAEEANEEDFDDDDDDLLDTIEITVTTDGAPLPRDDAEDIEIINLSPQQQLHLAGATAISPTTNNINAAAPPLLNLSEEFPNWPWETLSNNDETDDAMISGSSSGPSTSTRANALLSVMSSSAHSGGDGSVMDEVASGSSGGPSAGAVPSYCFDTSSFCSRTRKEVAALIVAQCCSGGPTPDLNYIMDRFFNPSTPIDNPDNISWIRWLIAGGRTISDFVKIEIFKQ*

>Woma_00006261

MNIKLCSTIIFLITIANTPKVSPFTLKLTKIICNSYDKTFAIFKTCRLKAVSRTLQYFTMYLKLLKVPITDVDARFAFLKRSNGYKPFLYDFPFKCDFLKKMNPVTKLVWGWFKDSTNLNHSCPFVEDFEMKRLENRYLEEKLQVLPLPPGDYCFYANVTTYGEHKFDAYLCGIIS*

>Woma_00006294

MFIKQNIFFVLPCFCLVLAMAAAYNPHVFTNKLNMNVMNLMARSNDPQQAADCFEKYNPIIEELSKTYEREYNECCKTADDQRNDIDERTLISRQTLANKTADSCEALYECEKKEKVGNQYHCYVNEGNTNSKVLRSVSNEASSDWADYQEENRVVLFQEDACKNKTRQTFEQKTFEEYVNLGDCLLGKLVTSSTTAKVTSTTSSTTSTTTSSSNENTTEDDD*

>Woma_00006293

MQFEMNFLLLTILCIFGSSLAQQDFNSRYNYSSNSFKFLQSSPVRSMSCFDYYIPIINGIAEQFERSYRECLEEAETKYAAVEAETMVQKKDLSNRSQATCELTNKCKSEIFSSNAFECYATAGLTNAKSLYKISSDASIHLSDLRGAYRLIDREQQICISRAERKYNEDSDKAFDDLNKCILGQLPIPPGVSSKGVSIHAKVLK*

>Woma_00006298

MIPENIIIKRIPNYFKTQTQCMDDSTLPMLCSHANIASVNEDCVTCRSKGSKLVTISLDIEYFIYLIEAGKNQILSINKCNLKAKKKKNVKETVEEKSKKFERNLLNLEGLPESAIKDTLSLHIYPNPLLLLVKL*

>Woma_00006302

MFPKFSLIFISISLMGGLRADFTGYSDQDEFSEAVDILVNNLGGINCMAETLENLKAAAKEFSYQINNSCKPKVVNALKTELADAERLIKLIDKFNDYNLRICKNSHYDEENDADTLPTTACSDNVKTLMKGKYWRTHSKTREDIKTSLENSYGNKDVCRRVGLVSFNIQINNFEVLFDQCMQITQSSD*

>Woma_00006289

MKVYADLLIVILAMAAPAQAFFGAKLALLGLGGNIGGGGGVGSGSGNAGGGYKNIGSYGGGYGGGYAGGYGSGYAGGYGSGYAGGYGSGYAGGYGNGGGYSGGYNYNRPVYTGYNANNGWGQQSYGGGYAGSNEQVVKVIKVIIPREQAQAYYDSYARSSSSASAVSTASVSSSSSGWTPIVASAPATVQTYTAAQPVQTVQVSAPALAPVAVPVQNVQVQPVQVQTPVQSVQISAPAPAPAPAPAPVQIVQPQPRPVYVQPVQVQTPAQSVQVSAPSQVQVVQPPPRPVYVQPQPEPQPQYIQRPQPQPVYQQQVSVPRTIQYTVQQPVYQQLQPQPQPQPVYQQPQPLPQTIYQQPQPVPVPAPQPAPQPAPQPLPQSHEIVKTFKIVVDGESQHVPAQTYSPPPPAPSYVAPSTNNNVAWNSGAWGTASSSSATSDNSWKSGSIWESVGC*

>Woma_00006250

MLKFTLISLLLWTCLTWVSTSPTDVIDAAVDGEQQEEFSTGDFNTTIDDPDRPQPRYAQLNVNNKIYTVYFSKLNWFTALEYCNFHGQAIASIRNGEDNALLIETLHQYGLQRSEPLWLGATDLGHFGSYVWTSNGQPITYFTNWRYGEPNNDCGNEHCIELLNDGQWNDVRCDEKRSFICENRIPNTSYDSPIYG*

>Woma_00006286

MSPLTVLLSLSLLSTAIQAGVISGHYGGHLDANAEAHDFSEGLVKSESSNYESLGSLGDHLQQSHFPSDYAEAYKGAEYQEAAATSEHIEYGGYEHQHFPQYEHESFGHEYHHHSDLHDFDTKPVPGINHGKGALSYSTTYEFKDPKP*

>Woma_00006287

MTSSVIRIFEIIIVCLLAINLEGKSLQSTENINSNEQQNNRIKRGYGHNGGNDFSSSIQLSSPVLQYDDSGYQHSSSSLDTSSHLSTATDAVGADPWAASGLSPGYEVWLEPSAHISSSSGFDTAAIHASSQVQGDYIGDLHASSLGSSHPSGDYVTNNVFSHAEPSSQLHTQISSSSSSSSYGSGVGAIHFADIHDHGISAISDNVDFNNVADLKSIHYSKSYGK*

>Woma_00006257

MQVQLHFALTILVLILTINSEINTFTLKLTKLICNSNDKTFAIVKTCRLKAESRSLQYYTVYWKFLKLPITDMDAHIRFLKRGNGYKPFLYDFHVKCDFIKKLNPITSIIWGWFKDKSNLNHSCPLVEDFEIKRLENRYIQDKLQALPIPPGDYCFVLNITMYGKYILYGQYCGIISA*

>Woma_00006285

MKILQSLVVLWYFIESSRVQGNPFFTRFNVGRLFTDIVNGIGKEIPNIIPTPNELFHASAQILFGLPEVAIFKTINQLCSVFLASDTINPMVTPDMDSIAFILRTPCQDLSFPLTQAEQLLESPEFDVSKKVVIFVTGWMSNPNEDYVSDMAQAYHCRGGYNFLSLNTSNAIDTLYSWSAFNTDEIGRQLALGLQKLVNKVPVENIHLIGHSLGAHIVGYAGRYFTKYTGLQISRITGLDPANPCFNEGEALSGLQRGDAFFIDVIHTNPGVLGKSDPVGDVDFFAEGLAPIKPGCEMFGCSHGRAYEYYIESVYPGNEENFLGVRCNSLSKLKDGFCTGDSYPMGFAVPSNLKGNYFLRVNSKKPYGKNASDENENSDECGVCES*

>Woma_00006283

MHFKLLLHVFMAICTFGAIYCGDFGFTNVFKSAVDVTSGVAKDVTNKLPTPTGLFETSKQLVAGYPFEFVSSSINKICSSALASKTIAPQFSPDINKMQFQLRNACERYSFPLLKANEMWTSPHFDPSKKVVILATGWTTTVNETEAIDVFAKAYSCRGDVNFVAVDAASFVDTLYTWSAFNTENLGMHIAEGLKQLIKIVPVENIHLIGHSLGAHIVGAAGRHFQYITNKSIPHITGLDPAKPCFNEGESLSGLLRGDADFIDVIHSNPGVLGKRDPMGDIDFYPGGLDPLPTGCLSVVCAHERAWRYYAETIYPGNEQNFIGIRCSSLTRVREGKCPSKEVPMGYAVPTDAKGNYFLEVKGEEPFGERASKQRMLGLESCGVCKEKKN*

>Woma_00006205

MFLTKINVSIAMLLAITSHLANAQFDPNFVTGRTTIVHLFEWKWDDIANECENFLGPKGYGGVQVSPVNENVVSPNRPWWERYQPISYLLVTRSGNEAQFANMVKRCNNAGVRIYVDIVFNHMAAGQGSVVGTAGSRADPESKHFPAVPYSSLDFHHTCSINNYNDVYQVRNCELVGLKDLDHSKSWVRDRIVEFMNKLISLGVAGFRVDAAKHMWPGDLKVIYSRLNNLNTAHGFPTNSRPFITQEVIDMGGEAISKYEYVDFGTVTEFRHSDSIGKVFRGKDQMRWLINWGTDWGFMPSDRSLVFIDNHDNQRGHGGGGSNILTYKNAKQYKMATAFMLAHPFGITRVMSSFAFDNSDQGPPTTDGNNIRSPIFNPDNSCSGGWICEHRWRQIYNMVGFRNAVGSSNIENWWDNSSNQIAFCRGNKGFIAVNGDSYDLNQILQTCLPAGTYCDVISGLKDGSKCTGKSVVVGADGYATVIINAHEEDGVLAIHVNSKL*

>Woma_00006282

MKVFIILFALIAVASAGYINSGWNSGGYGGGYGYGSGYGYGGGWNSSPRVVKVLRLGGGYGGGHGGWNYGGYGGYHGGSSGWW*

>Woma_00006280

MRRSIVILLVGLSAQALAGYLGGGGGGWSGGGGGSGWKSGGGGGWSGGGGSGGWSGGGSGGGTKIIKVISLGGGGGGGGWPSGGSSGWSSGGGGGGWKSGGGGGGGWSLGGGGGGWPSGGWSSGGSGGGWKSGGGGGWSGGNSGWSSGGSGGGWKSGGGGWSSGGGSGWKW*

>Woma_00006281

MRLIVVTLVICLAVTVVSGGFLGGGGGGGGHGGGGGYGGGGGGGKRGGGGYGGGGGKGGGGGGYGGGGGKGGGGGYIIISSGGKGSGGGYGGGGKGGGGGYGGGSKGGGGYSGGGGGKSYGGGGGGYGGGGGKSYGGGGGGYGGGGGKSYGGGGGGGGKYGGGGGGGYGGGGW*

>Woma_00006246

MLFYPIICVVLINLLWPSQAQQDGITINTGSKYYYIETAETANFFKAAHACAKLGMKLATIETETDLRRIKSTLILNDISANIHWLAGTTNGLATPTKFIDFLTGKKFKLFSTLAPAATQCISTAASTFEYKADVACTVANYFICDKPLLPYCGKNGQCRYRY*

>Woma_00006247

MSPSNKTFLLPLSLLALFILASTVNGQNGITVDLGDKRYFIINTQKYTWYTANHACTSKGMALASIETETEYQNLRKYLLAEDQLSDEFWLSGNSLDGTTTFTWLSSGNAFKLDKFASGQTPTPSKCIKTNAAFKWEQEDCGDATKTFYAICSRPLIPDCGLTGGCRTDLSFFF*

>Woma_00006248

MSLNLKFVSLVLSVIVFKLLWSVNGQSTTIGDKYYFVMTSKKLDWYKAYQTCAGFGMSLASIESESENKSLKDFLYEQSILSNTYWLSGTNLADKGQYSWQSTGKPMSFTKWSPNQPGTLANRCVHTNSNGEWVTADCYTAKYFICSKPVTTTCGPSGSCTPNYQLAF*

>Woma_00006268

MKIFVALLAAASLMATANAGFLGLGGYGGGYGGGYGGGFGGGHVGGGYGGGHVGGGYGGGHIGGHVGGGYGGGYGGGYGGGHYGGGGYGHGGGGRVEVYKIITTHGGGGGYGGGYGGGYGGGYNGGYGGSYGGSYGGGYGGGYGGGHGGWGGWGKKK*

>Woma_00006269

MKVFVCLFALVAAAQAGFLGFGGGSSGGGYTYSVGHSGGYGGGYGGGHGGGYGGGYGGGYGGGYSSGGSAAVVKVISEGGHDHGFHGGFDGGYAGGFDGGHAGAVEDVKVVKVIHQGSAGGQGGFIGGGGHGGYSHGHAGGDVKIVKVIHEDGGFIGGGHGAGYSHGGYAGGYGHAGGDVKIVKVISQAGGDGGYGGGWSSGW*

>Woma_00006288

MKVYMCLIIAMMAMAMPAQALLGAKLALLGALKGSISGGYGNRGGYGGGYGNGGGYGGGYGNGGGYGGGYNYNRPVYTGYTSNNGWGQQSYGYGGYSGSYAGGYNGAYNGYNSGSQQIIKVIRVIEEPQVSYENYARSSSAASAVSSSSSSAGWVPVAAPAPVVQTYAAQPVQSVQVSAPAPVAVPVHSVQVQPVQIRTPVQSVQISAPSPVPAPVQVIQAPPRPVYVSRPVQYPVQHQVYQQPQPLPEVRTVNVVVEGQAQHVPAQTYGPPPPAPTYVAPNAGNNGVWNSGSWSNANSNVGSWKNGGIWERIGC*

>Woma_00006220

MQFLKHLLLIAFACIALASQLVSAEPQFGSHSPSGLNRPRPQTLPPQRPIQPDFNGPPVAENSRYKEKSKTKDLLKRKDKS*

>Woma_00006223

MKFYALAIVTVALLGALIEALPQRPHFQKLPYYPPPTRPPRPIRVRRAVLGGQLTSNPAGGADAKVDLTHAIGNPDHNLIGQVFAAGNTNKGPVATGGTLAYNNNGFGAAIGKEHIPGVRDTVTKSLNADLFNNGIHKVSANVFKSDNTLANGLQFERNGHGLGYSHIDGHGASLTQSNIPNFGRQLELAGKANLWSSQDRNTRLDLTGGASKWTSGPLSGQRDFNTGLSLTSMFG*

>Woma_00006195

MHLKFICLLLTACLIIQTVTADANTSKDGKKIEKPAKRIRKVKVAKRLQERPQAEEPKPAEAELDDLENYDYNNYDEDVDTDADVDKLDTHEEVAVSSTPRSRAVDNVIAAFSCHFEGNWYRENDEFTSERDNCMHNCVCVEGKVICTASTECKRNVESVSNQPSDERPLTTTAKPLTTTTTSQPQTPAYNPYDASVQGPRGERGFTGAQGPVGDPGYPGSPGNPGVPGNPGPPGPIPDMSYYHQQLAAEYANTDKGPTAPMFAGPQFIQATAGQPGPRGPPGNMGPTGPQGFQGVRGEPGEPGLQGPPGPMGPRGLPGPPGKDGLPGEDGETGPQGLAGTPGPRGLPGLPGQPGLKGHRGFPGLDGAKGDQGAAGEKGSSGAMGPVGPTGPTGPAGPRGERGREGAAGQPGARGLDGNPGTPGQPGAIGKPGPPGFPGIPGAKGDMGHMGPKGDQGLQGPRGEAGRPGATGENGLPGAPGKDGMPGDKGSMGAPGIAGPQGFPGPRGPDGLPGSPGEPGIKGAPGQPGERGYKGDQGIKGESGLPGPRGLPGPVGPEGKRGKRGMRGPTGPAGPTGERGSQGLPGLPGPDGPIGPKGVPGDRGIQGPVGDKGSPGDIGPPGVPGLQGIRGLPGRVGPVGRQGAPGERGIPGADGKPGEQGPQGLQGLPGPMGMPGDKGIPGEAGKSGEPGAIGPPGPRGDNGKDGLPGAQGPPGPPGLDGARGEAGPTGPRGFQGLPGVAGSPGAPGKDGAQGPPGPQGPIGPVGLRGERGYPGERGPIGQPGTPGERGEPGNPGFDGPPGAPGKPGDKGHMGAPGMMGLPGHKGLPGPAGVKGERGSPGQLGPEGPPGRPGDRGPQGNVGAPGAPGETGERGEPGNPGTPGEQGSPGVPGDRGPIGPAGPPGFPGAPGLAGLPGAKGDRGLMGIKGEQGHSGPQGAPGEQGLPGPIGLTGPKGARGETGLRGEPGLVGLPGRPGDTGAPGQPGAQGLQGVAGLPGLKGNPGEPGRPGMPGTPGLQGPPGIEGQKGETGNDGPPGPAGNPGPQGPQGERGITGLPGPPGAAGLRGIRGAQGEPGQPGKPGKEGAAGPQGVAGPPGNPGPLGEQGPEGPPGKQGPPGVAGRPGDKGPPGQQGNQGPAGNPGLPGPQGPMGPIGPVGERGPKGETGLPGVEGPIGLRGKAGPPGPEGPKGETGEPGKPGEMGMRGPQGRDGNPGPPGQTGPPGPRGYSGNDGKPGPMGPAGPPGPPGPPGETLGYDVAALTALLNQGQTKGVDVLGDQPMIEQFTEGLTEEEKHALVLKAFEHVRASFERLTRPNGQQSAPAKTCRDLFAAYPDYNSGDYWIDPNEGDPRDAILVYCDKETRGTCIKPQPKETKVISYKGEGQEVWLAEMPGGMKIHYKADSYQMGFLQLLSAKASQKIIFHCRNTVAYRDDDKNSSRNGLKLLSWNDAELTPEGPKRLRYNAEIDECRKHSNSWAKTIITYTTEKSQRLPIVDIAIRDVGQANQEFRIELGPACFY*

>Woma_00006192

MFCKLQIFIAIALIIGIIKAQKEPNFADNRSTIVHLFEWNWPDIAQECEDFLGPQGYGGVQISPPNEVSVIDGNPWWARYQPVSYILISRSGNEEDLADMIKRCNDVGVRIYVDIVVNHMTGYKEGETVGTAGSVANYGERSYLAVPYGPEDFHAACDIQDYQDSQQVRNCQLSSLLDLNQTVPHVREMIVNYLNKLVELGVAGFRMDAAKHMWPQDLEAIYAEVNDLNVDFGFPEHSRPYIYQEVIDLGQENIKKTEYNHMGAVTEFKFSSEIGRLFRNKNSLKYLRNWGPEWDMLPAEDAIVFVDNHDNQRGHGAGGADILTFKTPELYKLAVAFMLAHPYGKVTRVMSSYDFTNTDQGPPHDEEGNIRKPEFLSSGLCDTESVGWVCEHRWPEINQMVQFRNAVGEEPVENWWEYGEHQIAFSRGNIGFVAFNLETEKELQALIQTGLKQGVYCDVLSGQKVENKCTGLSVQVGADGLAAIELANGPRALAIYEDSKLD*

>Woma_00006193

MKMLSVIQLAARLLLLPQAKSQKEPHFKGNRSTIVHLFEWPWRAIAEECENFLGPQGYGGVQISPPNEVREIENNPWWSRYQPISYILESKSGNQLELEDMIKRCNKAEVLIYVDIVVNHMTAYADGDSIGTGGSRCNYKKNLYPAVPYGPGDFHAACNITNYQNPSEVRNCQLNTLLDLNQTHSHVRDMIVNYMNKLVDMGVAGFRIDAAKHMWPNDLEIIYGRLKPLNDKLGFPKNSWPFIYQEVIDLGGESIKKTEYSPLAAVTEFKYSLEIGKLFHGKNSLKYLRNWGLEWSMLPSQDALVFVDNHDNQRGHGAGGADILTFKEPDLYKMAVAFMLAHPYGKVVRLMSSYAFNNTDQGPPLDNQGHIRAPTFLTNQLCDTANTGWICEHRWPEIHQMVKFRNAVAHLPLENWWEYGENQIAFSRGNSGFIALNMEKDKKLQATIQTGLSAGIYCDVLTGSKENNKCTGAEVKVRTNGEALINLDKGPKGLAIYENVKL*

>Woma_00006270

MKKIFVCLLALVVATQAGHLVGYAKGSGGWSSSNGYSGGYASASSSSGSFANHGYSGAGHNGGFNNGFSGNHGAVEDVKIIKVVSQGNQASHAGHTGNFGGYTSSHGGYSGNNAGYAGNHAGYSGHHGGFSGSHGGSDVKIVKVVHQDSGLLAGGNHGFSSSGHNDFASNGYSGFANGGDAGYTHVHGGSHGGFSGNHGGYTGGHGGYSGHHGGYSGNHGGYSGNHGGYSGSQGGFSGNHGGYTGSQSGFSNAATTSDIKIIKVVKPAGGSAGYTGGNNGGGWSALGKGSW*

>Woma_00006260

MKVQLYCALTVFILILTINNGVYASTLKLTKMLCHVNDKTFATFEKCRVKSESLTLQYIVLYIKFLQLPITDIDVHFRILRRSNGYKPFLYNTHLKCDFLTKLNALTNILWDWLKHSSNLNHSCPFIEDFEIKRLENRYIQDKLQGLPVPPGDYCFVANVTAYGKYRFDIQFFAKLYR*

>Woma_00006180

MLKTTLSSKSKLYIISVIKNSDANQEQRDSVLSLASSNDSVCDPKLQTNSNKDSAVTDIFKYADCVEACLENDFRDSAIYSDDTERRLDKNNSFRSSTSSNSNSKKPPLKSKPAYVPPPPTLNPTSTRSWVLKQIDNFNK*

>Woma_00005863

MTDEDSDAEELQLPEYDPVDETDSNEEFEEDAVEFFQNTFTARNGTIWLNQTPQNRKFLSHNILRFCNTGPAQKTIG*

>Woma_00005847

MDFFSDGGFQQLFCDLNEQIEQDYNSCSYENIKEKSSAPWPHQELTKTPIYEDHIDYRPQLMDLNLNEKHLQTSLDYLLNNTSTDTQEDSDLASISSINQELADWEYKFLDNYIEIPELVDFLPEKTPLCTDNCNHFLQESAENLKLFPSTPNSPLMRAEKCYPCTFGDCRKIYAKPAHLKAHVRRHMGEKPYVCNWPNCSWRFSRSDELARHRRSHSGVKPYKCDFCSKCFARSDHLTKHRKVHERRLLAASKAGKTLANGELPASVLTVRPGRKRKNQF*

>Woma_00005873

MFFTRQKICLYLLIVASGTDHTLSWPVFKPTALSEAETPVAYSKESLRSPRQIVQPTWLNPGQQEAPIDPIVHVNKEALEEADMIRKSIQKAVDEGTYAVTGEEVSSCF*

>Woma_00008123

MLGNIKCWCLLLAAFLCMVIDSTIAFDNRIVNGSTALPGEFPFMVSLRRASSGRHSCGASLLNRVWVLTAAHCVAKTQPNHINVQYGSIELDKNSTDVANVSTIYVHEGYEPANQYIHDIALLRLKKPVNVETDFVGVRLPELNASTDSKTPVTLIGWGLNATGGVVQKYLQKVNLEVFDDEECSSRHGLQMHSTTICAGVPDGGKGQCSGDSGGPLIFNHTQVGIVSWSRKPCTKPPYPGVFTEVSAYVGWILETILDAEDDEDENTEGLEEILSGNLIIVHKGRPQFLAPLQVNKHEGYNPTVIIHDIALVQLKDHIDLQAVKLPDEINGRYDNISATLLGWGINETNGVLMNQLQKVNLQTLTLSKCRSQLKSFIHNSNICAGGNRKGQCSGDSGGPLIYVNKQIGIVSWSLKPCAAKAGVFTNVSFYLKWLENKMKE*

>Woma_00008140

MNTFILFLLGYQTASNPYFGATVGRVCNRIANGRFVLKGQEIQVSRNSNNKHQLHGGFIGFDKVHWQVEAIHQDGITLKHSSPDGHEGYPGQVTAMVKFTLTEDNCLHVQMQAETNKTTAVNMTNHTYFNLAGHNAGRDAIYEHSIMIKASKITETDLESIPTGNLKSVDGTLYDLRTMINLGEQIKKLLPAANGYDDNYCIDANTDDSTTINIIAKAYHPKTGRWLVIASNQPGVQFYTSNYLPDISKGEAPLQGKNHSQYVKQGAFCLETQKYPDAVNHPNFPSIILNPGECYNHQVIYKFGTCRSGGCTH*

>Woma_00008187

MLLKYYCLPVILCILFELNYGQIEGILNPVVPPRPRWGPNMLLLRQHNSPAFEYWNERHEDNIWEHSGLFEGDIMLHREYLRNGLLNEKATWPDATVPFYIDSQDFNETQMMTILKAFKEYHDKTCIRFRPYEKGDKNWIVFKGNYSGCWSSVGRRTGGQVLNLNTPKCVTHGVVVHELLHALGFYHQQSATERDEYVKINWENILPGHTHNFNKYARTHVSNFGVEYDYQSVMHYSSKAFTKNGKNTIEPLDPNASLGQRRGLSEKDIEKLNEMYEDDCNTFNLFNIDRFGNSLDQIIDYLQGTLDKIFT*

>Woma_00008202

MPKTTFILVTVLFGVICGNRLMDNRFLRSAATTSYEAPSYKIIGDISANSFKGQINDDIKLKNQEKQPCVSSSIDFESDLTARNYNENLISLLKQTPPNYMVNDITSDDKINNVNIPKSYQTIRDFEENDDVFDVDERIESAIKTIPRLNSFPAANYKDFYQDLVVETTKQEMAKRNGNSLAAALGSPPPRGCDCKIRNDLVDLGQQHFPRYLLNAVCHSEHSRTDLGPKCWSGSVCRPLEYKVKVLTFRTLMDNMQKDPTLAWLPDDLRQIWKFKTVTVTAGCFCS*

>Woma_00008205

MMPEQKSLVTVGSSSLDTENLDEINLNATSEENQPTDIGDTNANPLLDPSIQIQSTNDSILSQAASSFSALPSVASNVFSTFSKRITGISSRETTPVSDQYIQDSNANLTTPQIDIPSTYIQQQQQHSNPGVSSQAPPPFYAAPPQGGNYFARDSEDTILFVMKEIYSGLGISPDSQVPQQTMTIERPSSLLFLFL*

>Woma_00008082

MSTKLIILAVVAISVVVMSTEASPSSRILGGSDVSTNQFPWVVSVRVDGAHACSGSIITENYIVTSGHCVSQIGNTPIESSRVTIRVGSINQYAGGQIVNVNNTIIHTSYGNFLHDIAIIKVMDSLVFNDKVNKIELANEDDVFEEGTLVHVTGWGLQANGASPYKLQQTEMSVLSSPECELQAGYGYDSVICLKHAENSGICRGDDGAGVLSDKKLISIASFAFGSCGTKYPDVSTKISYYRSWINSVIA*

>Woma_00008083

MASARLVFLLVNIFGISALAQPTSRILGGEEATKNSFPYVASIRLDGAHICGGSVISRNSILTAAHCFFENGEKVSENRIVIRVGSTNQFSGGQLVSVSGITIHPGYSGVKNDIAVLNLKNTLQWTERINKIDIATSSNEEPGPGAFVTVAGWGEQSSAVSSHKLHTIKFLITNDDECSAAYSENDKSTICLAHELKKGSCIGDAGNGAVYNNKLIAVSSFVVGACGSRFPDVFVSVPYYASWIESVLI*

>Woma_00008080

MKAFVITLCLLLSASGIWSAPQGRIVGGDDALLGQIPYQAALSIAGLRICSAVILSENYSLTSLTCVCSAGSDRPWPPQMFSITVGTTDLYTGGRDVTLEEIIINPNYKDLTTGIALLRHQKPLVFHDYIKPIALGKTNPPTNAEVEISGWGRIKENESEMQRSLQINEATVIEASECSKILGRTDNEVICLGHPRRNGICRGDFGGPAVYQNQLVGIAASVIGECGSFLPDVFTSIAFNYNWIMNQIIN*

>Woma_00008081

MALLLKVIAAVLALVASVNAVPTARVVGGVDAEVGQFPHQVSLQRSDGSHTCGGSILNERYILTAAHCVVVGNGVEPYPPQYFQVRVGSVQRTVGGKLLQLKRIIVNKSYGNLLNDVALLELEKPLVWTDKIKPIEMAAKDVPSGEDVIVSGWGRLYTGGPIPHRLQWNTLKALSTKECESAINWGFDSLICLAHEQNNGVCNGDSGGPATYQGKLVGVAGFVVGGCGTKNPDGYAKVSYHRQWIEENMTK*

>Woma_00008086

MRAENSLKVIFCMLWIFNVSALRVSNESDTDFKPVTKQPLKYFPQPRIVGGQNAKNNQFPYQISLRVDNYHICGGSIISKYFVITAAHCVTTASGDTVRQTPASKITVRAGSIDVNSGGVIHQISEIKVHPNYRNFNYDIAVLKLSKPLEFSDGIQAIALATHIPGLGVDVVTSGWGRLRTGGPTPRILQYNILKSISNPECRRRIGNVPTSILCLSHTTGNGVCNGDSGGPAVYNNQLVGVTNFVIGGCGSNAPDGYANVAFFYNWILNNTYMN*

>Woma_00008084

MLNVIYLLLLSILIGVVCHTCDGSILSKRYVLTAAHCVVTIGTEHHPAEQFTIRAGTINRIAGGVIVPVKRILVHPSYVVYNELTLLELEEERAIEMYDGEVPANSEVIITGWRLSEHAGANLPIFLQWYTITALSKTGCASKMGLYTDAILCFNHPSGTGACNGDSGRPETFNGKLDGVAGFVVIKCGSSRPDGYAKVAYNIDWIKENMVKHGLFE*

>Woma_00008085

MGNLTVFTFIALSLLCVSRSQPTGRIVGGYDAAEGQFPHQISLRRNGAHICGGSIISRNYILTAAHCVGVEDENGVYYPYKANLFTIRAGSTNRLMGGVLKQVVEITVHEDYGNFINDVALLKLDSSLIFSENIQPIDLASSEVPVGSDIIVSGWGRIKTGGDSPIKLKWNILESISKMKCASSIFMSSDALICLAHTEGNGACNGDSGGPATFNGKLVGVAGFVVGGCGTTNPDGYAKVSHHLDWIKKHSDV*

>Woma_00008134

MCNFVLSTPMLYKRNPDDKYERDLVPVSSTVIPLTALEVSYGLGGKPSEEYKEAYLKKLRKKVQHEKRGDEVQEDPDTLITIKKKHNDTNDKAKVKGIITAATIESNN*

>Woma_00008192

MVQKCVILLLTLINIFVKAEKYPVVLWHGMGDTCCLPFSLGSIKKLLEAELNNTYVKSLEIGGNIVIDYESGFFIHPNTQVEYACKELAKDERLQGGYNAIGFSQGGQFLRALAQRCPTPPMKVLITMGGQHQGVFGLPKCPSLRIKTCEYIRRLLNAAAYDTMVQLELVQATYWHDPLNEKKYRNRSTFLADINNEVFINGLYAENLNKLEKLVMVKFINDTIVQPKESQWFEFYKPGQDKEILPLKQSRVYEQLGLNHMAANQKLVFLEIEGDHLQFNKTWFLETIIPLLNQDVVGNRK*

>Woma_00008113

MISVSKLFYSTSCLLLAHQLYYPALAQNHSVCEKSSSFCTSTLSNQNMSDNHYAYEAGTSSVAFVTVPDDKIAKKLSRGLVERKLAACVNIIPNIQSVYLWEGKINEDGEYLLMIKTRTSHLEELTKWVRDNHPYSVAEVITLPIETGNFPYMKWLAESVPDKQ*

>Woma_00008154

MNKFVIFLCASVLISYTNARPDLNLGYRYNQGYSSGGGSFLQAPVAVSSSYTPTVSGNSYFASQPSLGQISSSGFVPSTQLGFGGNQGSFSSSNQVYTGGVGQTNFNNGFSGNTQYTLGSHTQNHQEVQQGFQAPIVTKHFYVHTAPSDDDQQDIVRHIQIGQPQKNYRVVFIDSSSSHSAKAKIVADLAPTVEKTAIYVLSKKTNALDIETEVNTPAPVNNKPEVFFIKYKTPAEAVHAQHAIQAQYDALGGTSDVSNEGLVPVSSVIGTLNQANGVGDIDHSASSNVGSVSADSTNAVKITHTGVNTQQTYLPPAHSYKIK*

>Woma_00008155

MHIIVILSALLVVACAAPQYGYQQPDSFNERAINSYNGLSPVNLQQPSVQRRGGEKYLPPGQTTVSPLVRKQFYVVSAPEDEDNTPKTKHLVLGRPQRNYRVVFIKAPNSDGGNVKYSAEFAPQEEKTVIYVLSKKDNELDASDIATPAPTPASKPEVFFIKYKTDEEAQKAQKEIQGEYDKLGGINEVADETTAPITSVIGSLDSLNPDGSYNYQQVSSPKALPQSPSSQYLPSAIKRTK*

>Woma_00008156

MYYLIVIALSLTALTSATPQFGYGYEPLNGAPISSGHRGGDKYLPPATTTPSPLVRKQFYVVSAPEDNDGQPKHKHLVLGRPQRNYRVVFIKAPNSGAGNVKYSAEYAPQEEKTVIYVLSKKDNELEASDIATPAPTEPSKPEVFFIKYKTPEEAEAAQKEIQNEYAKLEGPNEFSDEGVAPLTSVVGLLDGLTPDGAYNYQPISPGSQSEQKSVISQYLPPISK*

>Woma_00008157

MRALLTLCSLIALACAKPQYNYGSGISGTFGSSFGGGIGGGLSGISGIAGLGGGLSSGGQFAGPTLKFQGSRTAAGSVGDGSSFGGNSFGFGANGGSSFGPGAIGSGSYQSGSASNYNQQQQTGGHFEAPFVHKQFITVAAPEDNENLERTKHLVIGRPQKNYRVVFIKAPSSSNANVKLSAEYAPQEEKTVIYVLSKKDSSLEVGDIATPAPTVPSKPEVFFIKYKTEEEAQHAQKQIQAEYDKIQGSSEHTDAGVAPQQSVVGILDGVNGGNSGAIFGNSGNSIAAGSGISNSFSSGSTITHSAAKSSAYLPPAGH*

>Woma_00008195

MEYPILVFFISVLLAVALISNVHTAKLICDLSNACSRNHCLNSTPGCIANTRCLRNTYDYGAERGPHTWICTGKNQSPINILDEYIQKFPIRELLTWNHYDDLPCSVVLENNGHTVILRAFYSGNTPTLSGADLLASYTFVELCFRWSLLNNEGSEHMLNYKKFPLELQAMHRTGPLGECTSSYDLLMVSYFFEISANNPYLDPVIQNLHRVRKPGTKIHISPFPLSYLCYQFRTGFYSYGGSLTEPPCYEGAEWFLFPEPLAISERQLNEFREILSMDGKTRIVRNARPVQNINSSRIVNLNQYNPFDMNCLEKLNENGATCEEPLFD*

>Woma_00008190

MKIKNLFTTFIAQFLFIISVKAKDPEDSQLWICNTSDLCLLDTAPNIGDAKRFESQNDCRLSCGKYGAIWPMPTGECSLSKERIHFDPWKVRFNVVAPSAATTQFIRETNRIFLSNIIKECVRNCTLDNSKEVLVKATINSDSLSLDWNTDESYVLLIRTTGTATFVDIKAPTVYGARHAFETLSNLITGCITNGLLMVDAARIYDRPLYPHRGVLLDTSRNFMPLRQIRNTLDAMAASKLNVFHWHVVDTHSFPLEITRVPEMQRFGAYSPTNTYSRTDTINMIKYARLRGIRVLIEIDGPSHAGNGWQWGPMAGMGNMAVCINQKPWRMFCVQPPCGQLNPLNDNMYSVMKEIYEDIAEVGAPEETIHMGGDEVHIDCWNNTEEITKKMRETGFDLSLPSFYKLWSVFHEKNLESWDDVNQRQYPNIKEPKSTIVWSSHLTDPAYIENYLPKERFIIQTWVGSADPLNDQLLKKGYRIIVSTKDAWYLDHGFWGRTQYYNWAKVYNNKIESNPKILGGEVCMWSEYVDQNSVEARIWPRAGAAAERLWSNPKTSSLIAQYRFYRYRQRLIARGIRPDAVVPKWCVLNEGQCF*

>Woma_00008197

MKFFNITLLFVIFALAYQLSFTSAEEGGKRIALRRPVGKSAKPSSSTTSTQAPAEDEEGDYAENGEDNQYADEAEDAASTTTTTTTTETPKRIGPVIRPFRSNDDFLNSLKRRQMIAKKNKLEKPSPKPKPANQDSQEEAEESAPVAAPAPSKGFSKPNSALNRRKLNKPSKSEPVEDAAAEESEQEQKEEAKPKRPLGRLALRKRN*

>Woma_00008079

MLCRTTFTIVLVLYTSYKSVAQSEINSQPNGGETDSKLLEPRAYIPDTQYLDFVYHNHEELTKFLRSTSARYPNLTALYSIGKSIQGKDLWVLVVSSSPYDHMIGKPDVKYVGNIHGNEPVGRELLLHLIQYLVTSYETDQYVKWLLDNTRIHIFPSMNPDGFAVSKEGSCDGGQGRYNARGFDLNRNFPDYFKQNNKRGQPETDAVKDWISKIQFVLSGSLHGGALVASYPYDNTPNARICRSSALCAMFQTYSATPSLTPDDDVFKHLSLVYAKNHAKMSRGVACKSATPAFENGITNGAAWYPLTGGMQDYQYVWYGCMEVTLEISCCKYPPAYELKKYWEDNQLSLIKFLAEAHRGVQGFVLDQNNSPVERASLKIKGRDVGFQTTKHGEFWRILLPGYYKLEVFAEGYTPREVEFVVVEQHPTLLNVTLYPSTRTDNFPVSQFYRPLPGHLPNHHYRPAQSYADSGIISSITNGLNNLYSNIFG*

>Woma_00008215

MDISDNAFDSQQNAGQAQQQQQQQQQQMLQQQQHQSFVATTTPAGLQQMQCDSSLVDGTTTTTTTTHNSNNHGPLGGPIPLPAPPPHNYDKTPRTNAADNIDFDSLLLQQFSCLGTRDHEDLIEQFHSLMNNQMNKDAARFFLEMSNWNLQTAVGCYLDFCNGQTLPSMKIIQQTQPNNRQMAWRLQNIGSESWPNGCYLMSNSQARRIEVPTVRPGDTCEVVADLCTNEPVVWRLCTPYGEYFGDSLCLVAPGSFNSHEELNQRLAQLAVSEQNPAQFPQVNIVISERME*

>Woma_00008097

MSFLIALLSVLCKFLLLPLVSGHGRLMDPPARNAMWRFGYPNPVNYNDNELFCGGYAVQWEKNQGKCGVCGDAYHLKSPRPHEAGGEFAKGIISRYYTSGQEIEVEVELTANHYGRFEMYLCPNNNPRKEANQECFDRYPLYISGSREHRFLIPRDTKKKDIFRYRVRLPPYVTCTQCVLQWTYYTANMWGTCANGTEAVGCGKAETFRNCADIAIVSNTGGGVPPLFVNNRNPFLLYYRDYRAPKDKNVFPLIVRNTSRH*

>Woma_00006152

METRTDAVQHVEADNSSDTISTITSPQSNIHSQRNEDIELLSEELTSVLNNFNTAKAQFMGMVPGYRPHPPKRIMNKQMPRIIITFNATILPAEITSNIQYHQIIDLVYCSAYTILAIQDNYRNRVPQMNHSGNAQRETNRKPQWQIRLERKIERPWLPYEL*

>Woma_00006071

MKIAIVLLAIVGLVAASSISKQDVKIADKDFLLKQKFLFEIVYRVEDPLMFEEYIKFGKSFTFNKNDYTHFDMYMEKFYEAYKHGAVLPKGEFFGALVKTHLKQAYGLFEFFYYAKNWEVFQRSVAWARMHCNEGMFVYALTLAVIHRDDFHGLILPSIYEIFPQYFFNSKFVYEAEKFDYDVWSKYIMYEKEYKDILYKDYSTFFKDHYNQYYFYTKDFKTYQWWKLMGLGEHWYSEDRFMLRENFEKYNKDSKYLEIFEGTKMFFMPVDYTRDIDFYNKESALSYFTEDVGLNSYWYYLNMDYAFFLDGKTYGLNKDRRGEYWLYNVRQLLSRYYMERLSEGYGEIPEFSFFDKVEYGYDPQLIYYNGVGFSYRKNYYEIESYGKFDYYYKVLDFFNRLDEIITKGVYVTYDGQSIDLRKPESIEYIGNILQGNVDSFDKYFFKYWYMFAHMYFGDVDMNDMQVFPNVFLNYETMMRDPLFYMFYKKIATVYYQFFYYVKPYTHEELLFPGVTIKNVKVSELVTYFDLVDFDVTNLLNDKMTFVDGQFVWDKTLLARQQRLNHKPFDFEFTIESDKSQKVVIRTYLAPKYDEFGRVISLTENRQNFMEVDSFVYTLNSGVNQFKRQSKDFYWTIEDRTSYTELYKYVMLAFEGKYEFPLDISEPHCGFPDRLVLPKGWYKGMPMQFVFFVYPYTASYEPFSTYDYTYSCGIGSGVRHIDEMPFGYPFDREIDEYEFFVPNMYFKDVKIYHQDTFEKYYGKQYDKFGHFDYNYYH*

>Woma_00006073

MKITIVLLAIVGLVAAGSISKQDVKIADKDFLLKQKFLFEIVYRVEDPLMFEEYIKLGKSFTFNKNDYTHFDMYMEKFYEAYKHGAILPKGEFFGALVKTHLKQAYGLFEFFYYAKNWEVFQRSVAWARMHCNEGMFVYALTLAVIHRDDFHGLILPSIYEIFPQYFFNSKFVYEAEKFDYDVWSKYIMYEKEYKDILYKDYSTFFKNYDNKYYFYTKDFKTYQWWKLMGLGEHWYSEDRFMLRENFEKYNKDSKYLEIFEGTKMFFMPVDYTRDIDFYNKESALSYFTEDVGLNSYWYYLNMDYAFFLDGKTYGLNKDRRGEYWLYNVRQLLSRYYMERLSEGYGEIPEFSFFDKVEYGYDPQLIYYNGVGFSYRKNYYEIESYGKFDYYYKVLDFFNRLDEIITKGVYVTYDGQSIDLRKPESIEYIGNILQGNVDSFDKYFFKYWYMFAHMYYGDVDMNDMQVYPNVFLNYETMMRDPLFYMFYKKIATVYYQFFYYVKPYTHEELLFPGVTIKNVKVSELVTYFDLVDFDVTNLLNDKMTFVDGQFVWDKTLLARQQRLNHKPFDFEFTIESDKSQKVVIRAYLAPKYDEFGRVISLTENRQNFMEIDSFVYTLNSGVNQFKRQSKDFYWTIEDRTSYTELYKYVMLAFEGKYDFPLDISEPHCGFPDRLVLPKGWYKGMPMQFVFFVYPYTASYEPFSTYDYTYSCGIGSGVRHIDEMPFGYPFDREIDEYEFFVPNMYFKDVKIYHQDTFDKYYGKQYDKFGHFDYNYYY*

>Woma_00006072

MKIAIVLLAIVGLVAAGSISKQDVKIADKDFLLKQKFLFEIVYRVEDPLMFEEYIKLGKSFTFNKNDYTHFDMYMEKFYEAYKHGAILPKGEFFGALVKTHLKQAYGLFEFFYYAKNWEVFQHSVAWARMHCNEGMFVYALTLAVIHRDDFHGLILPSIYEIFPQYFFNSKFVYEAEKFDYDVWSKYIMYEKEYKDILYKDYTTFFKNHDNQYYFYTKDFKTYQWWKLMGLGEHWYSEDRFILRENFEKYNKDSKYLEIFEGTKMFFMPVDYTRDIDFYNKESALSYFTEDVGLNSYWYYLNMDYAFFLDGKTYGLNKDRRGEYWLYNVRQLLSRYYMERLSEGYGEIPEFSFFDKVEYGYDPQLIYYNGVGFSYRKNYYEIESYGKFDYYYKVLDFFNRLDEIITKGVYVTYDGQSIDLRKPESIEYIGNILQGNVDSFDKYFFKYWYMFAHMYYGDVDMNDMQVYPNVFLNYETMMRDPLFYMFYKKIATVYYQFFYYVKPYTHEELLFPGVTIKNVKVSELVTYFDLVDFDVTNLLNDKMTFVDGQFVWDKTLLARQQRLNHKPFDFDFTIESDKPQKVVIRAYLAPKYDEFGRVISLTENRQNFMEIDSFVYTLNSGVNQFKRQSKDFYWTIEDRTSYTELYKYVMLAFEGKYDFPLDISEPHCGFPDRLVLPKGWYKGMPMQFVFFVYPYTASYEPFSTYDYTYSCGIGSGVRHIDEMPFGYPFDREIDEYEFFVPNMYFKDVKIYHQDTFEKYYGKQYDKFGHFDYNYYH*

>Woma_00006075

MKIAIALLAIVGLVAASSISKQDVKIADKDFLLKQKFLFEIVYRVEDPLMFEEYIKLGKSFTFNKNDYTHFDMYMEKFYEAYKHGAILPKGEFFGALVKTHLKQAYGLFEFFYYAKNWEVFQSNVAWARMHCNEGMFVYALTLAVIHRDDFHGLILPSIYEIFPQYFFNSKFVYEAEKFDYDVWSKYIMYEKEYKDILYKDYSTFFKNYDNKYYFYTKDFKTYQWWKLMGLGEHWYSEDRFMLRENFEKYNKDSKYLEIFEGTKMFFMPVDYTRDIDFYNKESALSYFTEDVGLNSYWYYLNMDYAFFLDGKTYGLNKDRRGEYWLYNVRQLLSRYYMERLSEGYGEIPEFSFFDKVEYGYDPQLIYYNGVGFSYRKNYYEIESYGKFDYYYKVLDFFKRLDEIITKGVYVTYDGQSIDLRKPESIEYIGNILQGNVDSFDKYFFKYWYMFAHMYFGDVDMNDMQVFPNVFLNYETMMRDPLFYMFYKKIATVYYQFFYYVKPYTHEELLFPGVTIKNVKVSELVTYFDLVDFDVTNLLNDKMTFVDGQFVWDKTLLARQQRLNHKPFDFEFTIESDKSQKVVIRAYLAPKYDEFGRVISLTENRQNFMEIDSFVYTLNSGVNQFKRQSKDFYWTIEDRTSYTELYKYVMLAFEGKYDFPLDISEPHCGFPDRLVLPKGWYKGMPMQFVFFVYPYTASYEPFSTYDYTYSCGIGSGVRHIDEMPFGYPFDREIDEYEFFVPNMYFKDVKIYHQDTFDKYYGKQYEHFGYFDYKYYNY*

>Woma_00006074

MKIAIVLLAIVGLVAASSISKQDVKIADKDFLLKQKFLFEIVYRVEDPLMFEEYIKLGKSFTFNKNDYTHFDMYMEKFYEAYKHGAILPKGEFFGALVKTHLKQAYGLFEFFYYAKNWEVFQRSVAWARMHCNEGMFVYALTLAVIHRDDFHGLILPSIYEIFPQYFFNSKFVYEAEKFDYDVWSKYIMYEKEYKDILYKDYSTFFKDHYNQYYFYTKDFKTYQWWKLMGLGEHWYSEDRFMLRENFEKYNKDSKYLEIFEGTKMFFMPVDYTRDIDFYNKESALSYFTEDVGLNSYWYYLNMDYAFFLDGKTYGLNKDRRGEYWLYNVRQLLSRYYMERLSEGYGEIPEFSFFDKVEYGYDPQLIYYNGVGFSYRKNYYEIESYGKFDYYYKVLDFFNRLDEIITKGVYVTYDGQSIDLRKPESIEYIGNILQGNVDSFDKYFFKYWYMFAHMYYGDVDMNDMQVYPNVFLNYETMMRDPLFYMFYKKIATVYYQFFYYVKPYTHEELLFPGVTIKNVKVSELVTYFDLVDFDVTNLLNDKMTFVDGQFVWDKTLLARQQRLNHKPFDFEFTIESDKPQKVVIRAYLAPKYDEFGRVISLTENRQNFMEIDSFVYTLNSGVNQFKRQSKDFYWTIEDRTSYTELYKYVMLAFEGKYDFPLDISEPHCGFPDRLVLPKGWYKGMPMQFVFFVYPYTASYEPFSTYDYTYSCGIGSGVRHIDEMPFGYPFDREIDEYEFFVPNMYFKDVKIYHQDTFEKYYGKQYDKFGHFDYNYYH*

>Woma_00006143

MYCASFNSKVFCILVVSLLYEIVNAVPKYNTSGKTYDKNEWSRSFFNENKTQNLEQDNTTTKDRLQRLGYTTGYGTINGYPAGTGISAYNPIKLDLGGVVLGTLVGIGAIIIIPKILSAFHGGYAGYGRSENENELSSLTNFMNKLDDILGQNNIDSTSCMQRAICSYVRSTEYNVKMGASDQMDEIVHMISENSLVDYLLDGTAIKEALEHGKQINSKPCEEVYINCPLDRKKMSSMLIKLLPSKPSKTLSSSSKSNNNNQ*

>Woma_00006085

MRTVFDAILKPSSSSSEGNIQIEEQYNNHGTRNIIFTSKDLVYDNNFEGNKSYWTSGEIIQVGDRYIIVKAQANQLRSRTDETSLSSTTMPLDILLNEFQLPMSTFHEDFVTPPNSPFQLVQKRMTQRRQLVDLCPDHLNHIKRGDVNNVKSVVIDIKKNISRV*

>Woma_00006088

MWCYLMTSFLIVMVAAYDPSDKKIASVLPPEGMFEAFYPREMDGVPNSASRPAHAHGSFFKHRNPALVDTKNAAAYGYRFDGKRRFNFD*

>Woma_00006161

MYHSTLVILTSTVFLFNMSAGLYIKTTVDSQNLKTRNDDNNKDEWSVFKQKLGLTNQKVELLKSQKDQPGTLELLNRFVKENPMYKETTHKKQTKSSNNQFLTIYHNLLKQLKLSRSTLKTSLNFELADKTQKNVKRSRRKIAVRMVSDMPSEKIDDLLKMFNEKYGISNQDDNDDEIDEHAIDSYYSEFFMNLPSAMDPDYMYENLYEDEKDLAAPTKSNLSIQDLILQASRNQWQRKMEESKLLNRNNEHFI*

>Woma_00006069

MKIAIVLLAIVGLVASAGIADKKVKIADKDFLLKQKFLFEIVYRIEDPLMFEEYIKLGKSFTFNKNDYTEFGFYMEKFYEAYKYGALLPKGEFFGALVKTHLKQAYGLFEFFYYAKNWEVFQRNVAWARMHCNEGMFVYALTLAVIHRDDFHGLILPSIYEIFPQYFFNSKFVYEAEKFDFNVWSKYIMYEKEYKDILYKDYSSFFKNYDNQYYFYTKDFKTYQWWKLMGLGEHWYAEEHFMLRENMNEYNKDSKYLQILEGTKMFFMPVDYTRDIEFYNKESALSYFTEDVGLNSYWYYFNIDYAFLLHGKKFGLDKERRGEYFLYNVRQLLSRYYMERLSHGYGEIPQFSFFDKIEYGYNPQLVYHNGVGYTYRKNYYEIESYGKHDYYYKVLDFFNRMDQIITKGVYVTYDGKTIDLRKPESIEYIGSMMQSNADNLDKYFFKYWYMFFHMYFGDVKLYDMDIFPNTFLNYETMMRDPLFYIIYKKIATVYYQFFYYVKPYTHEELLFPGVTIKDVKVSELVTYFDLVDFDVTNLLNDKMTFVDGQFVWDKTLLARQQRLNHKPFDFEFTIESDKSQKVVVRAYLAPKYDEFGRVISLTENRQNFLEVDSFVYTLNSGLNQFKRSSKEFYWTIEDRTTYTELYKYVMLAFEGKYDFPLDISEPHCGFPDRLILPQGWYKGMPMQFVFYVYPFTASYEPFSTYDYTYSCGVGSGVTHIDELPFGYPFDREIDEYEFFVPNMYFKDVKIYHQDTFEKYYGKQYDKFGHFDYNYYY*

>Woma_00006067

MKIAIVLLAIVGLVASASFADKKVKIADKDFLLKQKFLFEIVYRVEDPLMFEEYIKLGKSFTFNKNDYTEFDFYMEKFYEAYKYDALLPKGEFFGALVKTHLKQAYGLFEFFYYAKNWEVFQRNVAWARMHCNEGMFVYALTLAVIHRDDFHGLILPSIYEIFPQYFFNSKFVYEAEKFDYNVWSKYIMYEKEYKDILYKDYSTFFKNHDNQHYFYTKDFKTYQWWKLMGLGEHWYSEDHFMMRENMDEYNKDSKYLQILEGTKMFFMPVDYTRDIEFYNKESVLSYFTEDVGLNSYWYYFNMDYAFVLDGKKFGLDKERRGEYFLYNVRQLLSRYYMERLSHGYGEIPEFSFFDKIEYGYNPQLVYHNGVGYTYRKNYYEIESYGKHDYYYKVLDFFNRMDQIITKGVYVTYDGKTINLRKPESIEYIGSMMQGNVDNLDKYFFKYWYMFFHMYFGDVKLYDMDVFPNTFLNYETMMRDPLFYIIYKKIATVYYQFFYYVKPYTYEELLFPGVTIKDVKVSELVTYFDLVDFDVTNLLNDKMTFVDGEFVWDKTLLARQQRLNHKPFDFEFTIESDKPQKVVVRAYLAPKYDEFGRVISLTENRQNFLEVDSFVYTLNSGLNQFKRSSKEFYWTIEDRTTYTELYKYVMLAFEGKYDFPLDISEPHCGFPDRLILPQGWYKGMPMQFVFYVYPFTASYEPFSTYDYTYSCGVGSGVTHIDEMPFGYPFDREIDEYEFFVPNMYFKDVKIYHQDTFEKYHVKKYGKFGHFDHKYYN*

>Woma_00010535

MFKENNFKILLICLAFGQICLAKPTNNEAKEQTNTPSNTRLCQCNNIWKDLLCENNNPKTPSLQLLEFVLSGLLDILETVTEKVENITDQILSDQELMQIANNDEVKIFIRKLQNSKEKSKNCRCRSQMDRMLNVFLTQCANYYKQEERDRWSPDGKAILNVIKKYDSEKMLDDFDTDVNKFIEKFDEKYESLKVPINEENTEMGEEISRWYRKLNTVRNTKKYGSKVRVFKQLVDMYKMEFIEKDSILFY*

>Woma_00010537

MFKENHFKILLICLAFGQICLAKPTNNEAKEQTNTPLNTRLCQCTNIWKALLCENNNQKTPSLQLLEFILSGLLDILETVTEKVKNITDQILSDQELMQIANNDEVKIFTRKLQNSKEECNNCRCPSQMDHMLNVFLTQCANYYEQEERDRWSSDGKAILKVIKKYDVEKMLADYDTDVNKLIEKFDEKYESLKVSINEENTGMGGKISRWYRNFDTVRNTKKYGSKVRAFKQLVDMYKREFIEKDSTLFY*

>Woma_00010536

MLKKNSLKILFIGLALGQICLAKPTNNETKDQTTSLNEWCQHKTTFSALLHCEEDNRKPPSLQLLNFTVSGLVDILVFTTEKTISVIDQILSDQELLQIAHNDEVETFVEKLQNA*

>Woma_00010534

MFEKTNFNILLICLALGQICLAKPTNNGAQEQTTSSNERNQHKITISALLHCEEKPPPLEVFKYVETDFRFILEATSVKAKPAIDKILNDKELRQIAHNDEVKAFIQKLQNAEEGCQNCRCYKKMENMLDIFLTECANYYKQEELDKLSSDGKEIVKVLKKYDIKKMLDDYDTDVNKFIEKFNENYENLKETIREENKKNDEKLKNWFEEFNTIKDGARY*

>Woma_00010539

MLRKKMFKKTNFNIFLICLALVQICLAKPANNEAQEKISLSNERCQHKKTFTALLFCEEDDRKPPSLQLFKFTILGLEGIFEITTVKAYPVIEQILSDQELLQIAHNDEVKAFMQKLQNAQEVCQNCRCFKRMERMLDLFLTECANYYKQEELDKLSSDGKEIVKVLKKYDVKKILDDYDTDVNKFIEKFNEKYENLNESINEENNNIGEKLKNWYEEFKAIKNCTMYGRKIKSFLKLFIIYREEFLENETTTT*

>Woma_00010540

MLLICFALVEICLAKPTNNEAKEHTIPPNGLCDYKTALAKLSSENDNQSPPSLQLIKFTGTGIFEFLANIAEKTKNMSDQILSDQELMQNANNDEVKTFIQKLTQFQEEYKNCRCLKKIGYIYGLFLYACADYYKLEELDQLSSDGTVILKVIKKYDLEKLLDVFENDANKFVKKFMEKYENLKESINEENNKIGERIGHWYEEFKTIKENGKKFMALNQLVDLYKQEFLENKTTT*

>Woma_00010541

MFKKNNFIILFLLCLALVQICIAKPTNDEAKENTTTSAGLCHFKAELSKLSLEKDNQRLPSLQLINFTLSAMLDYLLTFIERAKHKSDQILSDQELLQIANNDEVKTFIQKLQHFQAEYKNCSKNTDYILGLFLYTCADYYEQEELDQLSSDDGKKFVENFAEKYENLKESINNENNKIGERISHWYEEFKTMKDYKKKLNALNQFFDLYKQEFLENKKTS*

>Woma_00010547

MQTTTNKSTTTPTSPPISLTTNGHHHHHHQQQQHHHHPYQQQQQQHSHHPQQQQQQQQQQQLYPSITPTYWQPAPNPAPYLVPGN*

>Woma_00010545

MKLFMVVLFATLALNGVLSLPARSEDAEVIGRPRRVYTKADDDGDSNDDDFTNFKGVIDTGSGYPFLQPNPSVIRVGFFDSFDDLFRRFSARLFPVGLFGRAGTDSDEDDSSELGFPSIDSKKANSTSTVKVVDGHKVEVNETVYGDENSVFKVRVVNIRPLEEGETVEETETKDIRPVTQGPSDADAIKPANNNESAESDEKREPLEKKPLENEIQGNIDEPELSKITEKENIVDSITKETTTTYDLIMLPTTDDINAAFVTDNSLEHEQEHKEAEQELLDANGKGNNEFETNMSEDIEENAEDNVDNVENLEVVNAGEEVIDNLNKYSSELSSSANKAVENGDDADREEQEYNEELDNGNSVADIRNRERENEFSSDDEEDAETNTTEMHPTFNDEWGNLNEADKYDVLSNAISEEDDDEKFNGKPYDLSHDIDVNYVANLDINPNAEFIDGYSPNNPSNYMPRFEKLSLNNSPPAK*

>Woma_00014889

MISVWKSVMYFCIMIITIVNIEARPQNDLDSNQPLDTVVSDLEEIQNSYYDLPMPQQNVYSDIPLTRLQMLIARYNKPTFRPYLRSSSGGGVGELYRVPEAKRQVKYRQCYFNPITCFKK*

>Woma_00014887

MTAPIHRRLTRFFSNSFSGCFLLLLILQATTPLLCVQNNNDYKNVNKRLSSLGYNKNLPANKRTLISGSGIGGSHGQPEEIFSASDQLESNYDEYPMVVPKRSALLLDRLMVALHHALEKEHGGSMRLSEFYNNKNSYNDNNKDIPTEDGARMNDDNNADIIQDNKDSPYLAMYSDDDPTVMMDYDFKDLNSINRATGETLMAQSFQRRGNMGLDVVDDIGMEGVSSLIGTSSSSSSGLSRGILNGYNGGEVGSVGGVVGVGGTGRTASSYWRCYFNAVSCF*

>Woma_00014892

MTPCSVTNSSDSQLVMSGNTENEVETLEVPDNSSILEPDTANRNDEKFLETEHIIIPASTLTTPTRSEAGDVSSDPIGASRYSARQRRHRVFFSP*

>Woma_00014866

MFYELFSHHLIFILIVVPNIVFSGPVSSPDISTKDGKSNNHVNSQQQPQQSHYQKLNNNQDAQLNSLNVYASNFDVISSDQNDKFKPSYKLPEMETNFTPIHNGNSITSEFTPTLGSPTPILMQYLPQTINEGGVQYLQLIPTRPLMVPIAPYLAGNSGGQPITTYHQHLTPSNSIDYAARPLLSSLPVNMPPPAPASLIDLQSTTLPGYGIQPFAGSITPYKQNHRINRETKDKQLLGPISLNLNEYIPGPNSQQHSNTNIRSRP*

>Woma_00014880

MKSIWLFLFSLSGHFAFSHAWTPIVGQAATTAAAAAAKPAISGPKTTFTSSFSLQQSHPEFGSKTAYVSDSDVKEVKLPIIEDTGILTAKNRYKPFEMGPAVPSSINFNDAKFGYKIAGHEEDRCSVKKYAFNQDGVVHYDNKLPELAQFTICFWMRFTNHSGDHVILTYSTEDDNRQFQFWVANAKNSSFLSMAIKGQQMYRLNYPLRVRQWHHACTSWNGKTGEWQVWLKAERIGRGFHNSLVGHKIPANGKLFSGGSSVTGKICDGLHFEVTLVQVYRVALSAGKAHRDHKHHHVHHFDHEGQEIQITTKPPPPINRPQLMHSLLANGQIPTRVRINLAQPASSADTTASDVPVASPANAQQAVTIHTDFVNGQINAGSRLVAQQLIGLNHKLAIAPQTTQTVNRQAVYDPSNPSQIQFVDETETRIIFKRQADNSSLKEENEDNKKLQKRGLVYLDDGSIANDPLVEGTVSGFNKYNGLAEFGGQQFKNDLTDKHHNIEDEIVEHDREPAEEEVKAVMALCDACEIEPFQGAIVFAWKEANEKLDNVLKGYSVGGCGQF*

>Woma_00014864

MKIYSVVSYFILLLTITVMATAVSNSTSKETNTLKAAEKISYPKKVNSLKKNSSKISLKKIADITDKFKDLPADDLEFIKELDKQFKLHGDGELGYGYAHNGYEYNPPKFMFYPYSQHNIGNKASNNFLMGDGKTEVTIEPSYSYELKPQTYITQNEPQIDVPKPAQHQLQQHEHYEEPVIVLRIPGPAKYATHLQMLLQKYLEIRAAQYLRILEEAEQRNNQPQQVDYGQVNTAINEAHIEHTQQPQQQVAMPGELDYNQKVQYDHQIAVTPQPIQYPDIDNVYQNFKHIHSSQQKQFQQPQQDQQSYFGPSHSNIDQHQSQYNHHEQHELQYEQQVQQYQPQDQHQQEEQPQLYYSHQQQYAQPEGPPENIYHSDYNRANDLMEKQQPTYMQEQLNQNDHSQPIPITENNPRPSHTKVVFTKNNDHVNQEYQMYQLPSIKPNERHPPTSIPNYHQDFQQQQQNLQENEGQHYNSAEPATQEVVAITQRPFNYHAHNVKTRARGRTAQKKRVTTTKDADAGLQKIREYVREKLGAETGSAVEFKTTQLLDG*

>Woma_00014867

MKIFVCCLFTALLSSAFAGSKYEENSAKNKENNRALDKSSDSKLKRDASLLLGSYHGPSYKYLPPSSVSSTLDSYTGSSGPSSSISGVYNSDIEGNYRSDGAGKVHGHFGGFPHHGQQNSGAAHGHGYVSKNSYHNSGHHGLGYTSGHFSGNGFVPQKSSIKGTISSLSAGTLSQKGFSGGHVNSYALGHGYSTTNPHFINGGHEHKPMASGGKYHYSKVHSATFPSVASNGFSSGHSFSNSQGLGLTEVHHSDHVHNGGQEHIYIIPSGSSSFGSGLQEVSGIPNVSYLPPVQQHPIPSSNYGVPIINAGDTYKNNYHQYTPSAVDQGPTYAAGHKGLRHFSFTANKPQALHTSISDSSLKQPSFSKAPFKPSTFLGVKYEGSSHDNFPNQQYVPSSAIGTNPTGTGYDYPTPPDSLATYQQENYPQSLEVQSVAQTEPLSTYLPPSSNEGSFSEIASPNGNYLPPSNTYEVSYSH*

>Woma_00014916

MQTILEKEMQGLSLADIKNKKAAVEEVEYTTAKSGSTSRDESCYNIIIEPSEKRSNKSNLFFEIKRNFLSFKEMIPDKKKYVQKYGWLSNLVAAQMEEINVEHQQEAAWEI*

>Woma_00014929

MEEPTNTNDEKNNAADDSIDPTNPMQIASESFTSTTATIPIMEATVSPPGLGSNLHMTTSDTSAACMLENHKTEAKKLDDEIEILHRRYEILRLRKEIETMEHATDTTVPHRVVNFQGIENAIIKFSGEDRSFGITEFFQNLEEVLEHVKADELLKF*

>Woma_00014936

MTHLAKMCFTNSIIIILIFNTFNVNALVNSPKIISKDGHLIFESGLDRNITFLLKGKSRLNINDKYDVIDFLLQSKKGRITDPLSTQTEWSEEEDAILKQLMGDVTSLQTRVFGSNGLDFRFRLLQNRTSYAIQMLRRYKIRMQRVETRVQSLYDLLETDNCKSNPCQNAGTCMNLFGKYACKCPSNFEGPNCDKDVNECALYAGTDLGCQNGAQCVNQFGSYSCICQPGWLGIHCNQRKGDCLQSSIWELCGHGACVSSNDTFGYKCICDQGWKTNGLTPACTVDVDECTESHTACTTQCINLPGSFTCAPCPAGLTGNGINCRDIDECATQNGGCSMSPKVSCINSYGSYHCGECPLGWSGDGRTCTRNSNTDATSGGGGTAIGAITRCATSSICHPRASCHEISNTVVCSCPPGMIGSGIGINGCVIGTAKNCINQPCLNGGTCVDDGDGFNCICPLGFMGPTCLPAPSLCSPNPCRNGGHCHLITTATARRFICECRPGYRGEKCEKSANDCGGVLRGDSGRLRYPPGTQNLYSQNAQCAWIIRTNETLVLNVTFSSFLLEDSTECRFDWLQINDGPSAGSQIIGRFCGSHKPLGGNIISSSNQLYLWFRSDNSTSKEGFDLEWNSIPPQCGGLINVTTHGTISSPGSPGNYPKNRDCRWRLKANNDKRLKLTFFSLQIEKHDSCNFDYVEIIDAISGETLEKYCNSTHPQPVLLPTNEAIIHFHSDNIDADTGFQIFYSTEGRIQNCGGVYTAKEGTIQSPHIENEALSCEYEIRMSNSESISIMFQAYKMGEHDCLEIYDVNPVTNENILNGKYCGEYQSAPSIMRSQFNKLILKFYGKHGAQFKLNYKADCSYTFEAPEGVIQTPNYPQLSNKERDCLYRILTEPNTAIIVHIDDFDLRDSSLEDTDCAYTKLEIEDVSNKNRTGPYCGDRSPDADIVSKGNLLVFHLKTGVTTTGRGFKLSYKSVPMDKSECGGVYTKPGYSIRLPTDEEGLYAHDMICYWIIVAPRGKFIMIDWKSFDLEESTDCSYDYVELYDSLATEYKQALERYCDAHRPQSFLTHSRILTVKFVSDVSDAAKGFEFTYKFIDSSNQCGGKIYSSYGMVQTPDWPANYSANLDCVWIVQTSPSTQMELEIEVFDVEFAKNCSNDWLEIRNGGSNHSTLLGRFCGNITQLPRLMPSFTNQMYFHFHSNGFVNNRGFRLFWRVFSSGCGGNLEGQQGVITSPFYPEPYPNKAECDWRIHVPAGSAIHLTIEDLALESYSNCRYDSLAIFEGTTETKTHLASVCQLESDDKPMEYIINNNEALVQLKTDDSNRERGFLLKFKANCTVTLTKNYGIIESPNYLKPYDEYSDHVNCTWTLKAPKGNRIEAEFTYYDTVDKKAKLDIRDGNNVTYNIQGQGHIINSTSDTLIIRQSTNKLNFQLEYAMIGCINVYRNDEGEFQSPNYPKPYANNLECSWEIVTQPGAGIEVEINDLDIEDSVNCTKDALVISPHYHSNNVKERHCGRHESLTINTASHRLYVRFNSDSQGNGKGFSVNYKVKKSDCGGVLKSRNGIITSPNYPNNYPENADCEWTLTVTSHHAIIFTLEELDLEDFYDCEMDYVEAYEELGDSEPLQLFKMCGSLDAETNTTWRSFGNTVTIHFHSDDSVPAKGFKLSYVEDCGQRVVLDENDYANFEISKHAQINQTCMWQLIAKDPTKHIVLSITHLQMNPAVAQLYPTEGDCLAQGGKIYDGTNDEAPLRVKFCKSHPADVISNGHALTLKVPFGLIAEIDAYAYQMDNNCGNVYGSLTGRFASPNYPNSYPVNIDCTWYVDASPGNSIGLTFEFMDLEDSDECNNDYVEIRDDVGTGHLLGVFCGNRLPAAIDGSESLIIRFHSNDDIVGRGFFVNYYYQKHNEINGTSGTIESPAYPSKFHSDEWYSWRITVDKNYVVLLSIKHILDTDIPFIKFYDGYSDIGAPIDYALTHVVRSNTNILYFTAKRGPFQIEWEQLSKEVVRSNQTAERMSELCGHQNLRINSSLLFSSPGFPHGYGNNLQCSWIIVPNNPAMHTEIMFLRIDLEEFDECFADYISVSKSTDLQNWQELDKMCKKPADKILRYEGEPYLKLEFVTDAGINKTGFSSIITTKCGAELTERRGFVNITELNGRVGNFQQGCVWTIRVRPGRRIRLTFPDFWLRTPNTEGNCGTYFLVRNGMAEDSPFLGKGKYCDNDISDVLETSSNRAYVKFTRAGYPAFRASFHYEEISHECSKEIILSDAYKGDRIQTITTPNYPNIPNPHSECIWKVIAPLHKTITLDFFGDYDLIPVNSDSKQCELEYVQVNDGGTEMAPVIGRYCGSTKPNTIRSTGNILRILYFTDVSEPHKGFQANVSISRCGGSYYDSEGIITAPLLQLKPKEKELECIYTLEMPLGSTINISIDKINLPESFSDHNTDCKEETHLELQEIDAFSTDVENITDTLFLCGSEPNRYIVETNKLRIVLRIKDGFYTTDSFQISYSAIGDRCGETIRGMQGILQTPNYPKGTGVPIHCTWHLEAPKGLRIKVEFLDFDAGSGMESSGRVYRRLTFSNDRKLSSIIERVTDTIPTVIYSSDNTITIDALMLSFYKNRGFKLKYTADEFSLHCRNLLWFYDESQMSKPQTLTFKREENAKSIYCSYDLSPQYNTTWSVQITKYTTFNTSQQWYIYGCQYSSAVQLYAGEERILPLLLCRNETQPNFRLPYAAKLVLTGHRRNALRELDLQLTTYKCGGIWPLWYYENFVIGLPQMTNHTGHLECAWAVWGYSGDTSTMPSFADIQADIQLDVNLSTNFKGKCNEEYLMVYNGPNQNSPHLGRYCEQSSITGLVAVGGLFIEYITANYTSLSSFTLNVSEGSGCGGTLHYPYREIVFDYQYKNNVECVWDLATDKGFHLSAIFSNSFFIESSPNCTKDYLKIQQKSVQGIWEDITTLCGREPPPVINTTTTEMRLIFRSDDNVVGYGFTVAFERNCGGVFYATDTMQELTSPNYPNEYPSNLMCNYTIIPSPSIIKTESDSLYIRFLDFILEDAPLNKCMFDNVTLNVMNNNDEQSTTILCGRKINYQMRSKKTISIIFQTDGSYGRKGFRLEYGHNKCGAVITNSSIIESPKDSSTHMYPHSSICTWKLQAPENHKITIKFEYIDFESQGMCTYDAVEVYKGLNPVEDQRLMQLCGNITGQKEIINIQQNTGLIRSFSDDRDASRGFKALVKFMPNCDNHIYLAPNNFTYEFTRYSGQYDNNLDCSWLFTTTPDRQLRLEFSSFHVENSSNCMNDYLDVRDGSGTFADQIGQFCGHDIPSPMVSSKSVLLMHFVTDAQETSSGFIATIKAVPKICGQQNYDLSTQKVVNIASPDDGTGKYPNHINCIWKIKADNNIHLHFEKLDIEGPDANGSCSSDYLKIINSEDADLIDKGYGSQLVFNGLIASYGSYYITSEYAAEHIYCGSKTPDDYFTISKAVFIKFQSNSQVNKGGFKLRATKAEGCFRNFTAAQGRIKIAESSDHCDIYIKAPANTTLSLYYTDLMFSEYDCEKENIEVYDVRTNTSLQKLCEFVDTGKSLFSNTNELRIRLKLSGYYTQVDITYLASTNGPGCGGDFYNTKGLLTNPFYPQHVRNNSDCRWNIRVPANLKVLLKFPVFNMGAKSTCRTDYLQIIEHEDIDKGEEKVMRQFCGEDEPRYYLSTKNYVTVRFHKSVNYDGTGWVISFSGVHPSFNTNYIY*

>Woma_00015023

MYKNLEGPAVSIEVTAATADTAALGLEDAAANIEVAIHPMEVTDTLPKHQMQTEVTDGKINFELQHDITALENGEPTQHVVPPVTASFDASSRFTFRDLEHTIINFDGEWLDGYGWS*

>Woma_00015035

MVYDQMKFLKTHLRSRPSSSNISHEEIHNANDSAEIVIDAYDDDSVPGPYPSSNTSEILKLTDQINGVKKLISKRLEETSDYDWLSKQVDFGLKSIQDEEIRQETVWKIQDILREAVRADISKRNCTLPVHT*

>Woma_00014962

MKGLLLLAACVGIVAFSSITEAYPQRVYVQKLPYYPPPTRRPTYMRARRSVVLEDSVSPNLNGGQDVSLKLSKTVGNEHVQGAASVFAEGNTKGGNVVRGATVGVATHGLSASVTKAQDDIAESFRKQAEANLKLGDSNLITGKISQTDTKIKGVDFKPQQTSTSFVWKNGRVGAGISRDVDRGVSDTLTKSLSANLFSNDKHNLDATVFRSNVLQDNGFSFKKTGGILDYSHANGHGLNAGLTRFHGIGDQANVGGYSTLFKSNDGFTTLKANAGGSQWLSGPYSGTRDFGFGLGLGYNGWRG*

>Woma_00014963

MKGLLLLAACVGIVAFSSITEAYPQRVYVQKLPYYPPPTRRPTYMRARRSVVLEDSVSPNLNGGQDVSLKLSKTVGNEHVQGAASVFAEGNTKGGNVVRGATVGVATHGLSASVTKAQDDIAESFRKQAEANLKLGDSNLITGKISQTDTKIKGVDFKPQQTSTSFVWKNGRVGAGISRDVDRGVSDTLTKSLSANLFSNDKHNLDATVFRSNVLQDNGFSFKKTGGILDYSHANGHGLNAGLTRFHGIGDKANVGGYSTLFKSNDGFTTLKANAGGSQWLSGPYSGTRDFGFGLGLGYNGWRG*

>Woma_00014964

MKGLLLLAACLGIVAFASITEAYPQRVYVQKLPYYPTPTRRPTYMRARRSVVLEDSVSPNLNGGQDVSLKLSKTVGNEHVQGAASVFAEGNTKGGNVVRGATVGVATHGLSASVTKAQDDIAESFRKQAEANLKLGDSNLITGKISQTDTKIKGVDFKPQQTSTSFVWKNGRVGAGISRDVDRGVSDTLTKSLSANLFSNDKHNLDATVFRSNVLQDNGFSFKKTGGILDYSHANGHGLNAGLTRFHGIGDQANVGGYSTLFKSNDGFTTLKANAGGSQWLSGPYSGTRDFGFGLGLGYNGWRG*

>Woma_00014965

MKCLLLLAACLGIVALATITEAYPQRVYVQKLPYYPPPTRRPTYLRARRSVVLKDSVSPNLNGGQDVSLKLSKTVGNEHVEGAASVFAEGNTKGGNVVRGATVGVATHGLSASVTKAQDDIAESFRKQAEANLKLGDSNLITGKISQTDTKIKGVDFKPQQTSTSFVWKNGRVGAGISRDVDRGVSDTLTKSLSANLFSNDKHNLDATVFRSNVLQDNGFSFKKTGGILDYSHANGHGLNAGLTRFHGIGDQANVGGYSTLFKSNDGFTTLKANAGGSQWLSGPYSGTRDFGFGLGLGYNGWRG*

>Woma_00014967

MATKIVFTVVAVLLCATQAVLGINIISKSQWGGAPATSKTSLANGLSYAVIHHTEGSYCATKAACIQQMKNIQHYHQKTLGWADIGYNFLIGGDGNIYEGRGWNVMGAHASKWNSKSIGISFIGNYHKRKPTAAQITAAKNLLSAAVARGQIKSGYTLYGHRQVGSTDCPGNILYAEIKKWANWKA*

>Woma_00014971

MENKIATTLFVSCFSSLTFALTIGSDTFKNCYVNDNEYNLAAYSKKEKSENKDFFKTEDIQNDSKINQIFPSNTYIKLDASYNNKNSITTKDDMLFNISDENSYICKKKANSLLHKPNNKNIENKLTKNLRNKNIMQSVQKFGINNNLMDTRKKSTLTHKVIDASPMGKFYLSHMEKIRKRMMRRANEYRNKNISFQSLCPTKRIPILLETSDYEYRPKHYTEVVCANIFAPKYSNLDDKNKVCSEAGFSCMQLNRTIHFVRRNKSESSACWESEIRVVPSGCECMWPKHYYGDILAYHESEKRFNVGVNNAYIPFLSVKN*

>Woma_00015003

MALSQGTDENLVTTMPNVPRTSTEPIATTTAPMNSGAGMTTMANSTAIMTSAVQQLATMASNNSFSEASQITNMSIDSPESCSMTARSLKREFWHDLPVTHQPQATGAAAGGELLSFLCDEFTQQIQEFQRVMQHEIA*

>Woma_00014997

MIKKYEILSHIHSGGVSSSMDYNDVEDVSPIVSLTIPSEPYNTFGNNTSNLEARFIEEIGPNILSPVGYATIVT*

>Woma_00014998

MVLIRLEKNMALSQDTDENVIAIMPNVPLISTDAIATTTAPVVSSAGIPVMAITVQQFLTMVDNNSFSKASQLDNTSIDVSDRQECEAGISAGETCNSTASNNWIESLWGSIQYSAGRIYM*

>Woma_00015065

MHQNRSLQEFAAEDDLPLSTLLNFHTITEFNNVNFEEFVNIDNAACIENDSIEIKFDEVNNNEEMLNDSDEENVVQENNAINSYNKLLI*

>Woma_00015061

MMFNFGVSRMVIVDNEKALNSESILFMLEDQLNIKVFQTPPYKSTVNGQVERFHSTLSEIMRCLKTEKIHRSFKELLDRAIYEYNYTIHSTTGRKPIEMFFGRRVSTNPEQYEKARKENIEVVVKKQKNDIEYHNKSRKPFKDYIPGPKILL*

>Woma_00015074

MTVTSDNLSPNRLATTSEVNIYTNNTITSPAVISVNTAVNDFNVPSAYPVPDPSISKPAKHANTAAINTDCPFC*

>Woma_00015054

MPLRISYEELQSSQKTDDELKTSLEGNNSLKLQIVHFDSSSIYCDISTGWSKRKAVRRAQNIYAFSYVYSKKNVRYSTNKDIIGIILQRSSKKETKNIKH*

>Woma_00015042

MDPSECLIEEVKKYECLYNLASKDYKNVDRKTEIWNEIGSVVNLSGDACRKRWRGLRDTYKRYKRAAQLASGSGAPTPSKKWKYMKLLEFLNNYKDARSTIGSGYW*

>Woma_00015136

MENPSGNSLAASLLNGNSNSSSTEAAPLINSNVSLVSVNPLNTNASQSPQPQPQLSTALPQSASVPHIPLPSSVELTLQLMDGLVMKKPMMLNVEQMKLILDKVDTISDNEEIVGNVQLLNDEIQLPKEIPSQFKVEDIYVDDNINLPKEI*

>Woma_00015147

MKEISIRISKTIGKQQFGIDRGNTSLYSIVLMAVADADYKFILVDIGALGHNSDWGIFKNCKFGKTVLENSEDMKFPGAKALPGTNISTLCGIMPAHDQNFNKHLSGARRIVKNTFGILTNRFRIYRKPIIACKETAINIVKATVCLHNFL*

>Woma_00015160

MKSVHASSPQFPSHTDSQLMQSGQFGTMFLYERNERYSTPWNTCQECNSLTIENLSLSNNRHNRHIPHRCAIRCTSNEEPIVYDTDGTPAESQIISQYAPNLSNNVCQSMPNVSEMSLEAHTLYNYTEVPDLSHIANAVVTNFNE*

>Woma_00015190

MGICDATYFILAANAQFGGTAHDSFVWRRSRAYRFLEELYDAGERNFWLLGDSVYPLQPW*

>Woma_00015187

MGICDATYFILAANAQFGGTAHDSFVWRRSRAYRFLEELYDAGERNFWLLGDSVYPLQPW*

>Woma_00015255

MSYLLTKTILVIILSSFSNGVEVHFETADSGFFLKHARQPPVTHETSTTHSSLLSSSRSQTTYDSVLSVDRFTVVQTTQAVSIRASYGPFSTKQTVPARYIVPDVIEKQLDYLNNTTAALLELQQSNIHLDISAHLVSTVVSQDSPVLRVLFHAGADPGGHLQRQKICVLLHVSSNNHGPIKGRCMPEGEDGVCVAEVVIPFSWFHSLTPPSHKENSNSIPAKVPQRFAQVSYSVFEPPVRNPELCEPKVQIQPLTSFVQVPLVAAIEPFKEFHIDETLIILLPQQPLYSLSKFHVPVFLQHNSNKNISSFTLKARIKSGMKVLEAASSSDLWDISVERDNLKHPTVRVTAMKKKADNNTSLSINDERIYEIFTWLLEVADDTKDLIEGGKIVWSVTYVYDNLKDSSVELPNEDNKKRIITKLEVNKDDIQAVLPMAKNWELMNTAVLTGRQVAQAMKVFIVSQGGKVADVTLQSSCYAEDESVIKVSSSCSSVYVDGSEIRGSSNASVIVKYGTYIGLAKFIVWMPEFPLEVYVTDFRLSQIKGWKVVEDHQYIHKKSRRKKRSFFPGQHNTGFFSNVLLDKMMCKARYQQSPVEVYAKFVAIDQHTFLSWKTIFINTSICIGTYKK*

>Woma_00015266

MYAIGYLRCFKIEIQNNFGEDIETNNDVLEEVLDELTENMSIVMECRRGFFSSIPANQWISGDSAYPLSSTLITPYRDNARQLNKNAQIALNLRHSKYWVRVEHCFGILKEKFASLKQLRMRIANKNNHKLCCDWFLVCSEDSGECSIDPEVDISYDSGEIKR*

>Woma_00015280

MIQAPLNSLFKESKRNDRRQIEWTLELEKSFGACKTCLANETLPHFLEPEAPLALVTDASDSAVGAHLEQLVQDQWRPIAFYSAKLEPSQKNYRTYDRELLAVYLAIKHFKHLLDGRNFVVRTNHKPLTYAFRQKLDRASPRQVRHLNFIGQYTTDITHISGIKNTVADAITLQKIQRS*

>Woma_00015315

MNSTKIIFAVALIAFCCVNLSYTLKCFVCNSKQTCKNAQMYECNSRLANDTRLYLQQFHTGINPNSTSPYFECFREYLKTSTGEIYYKGCAYATVDACRLPLSPHIAYTVQKQVCNQCSNKNGCNPADRANIDVINVFAAIIVAGLLRTCLRCYVCDSQKSCKSPKLLDCTPKIANDTQEYLHTFHSGINTNTTFNSYNCFREYLKVDSTEYYYRSCIYANVKWPGWL*

>Woma_00015316

MNCFTVGLFLAYACCLNFAYGAYKLQERYSWNQLDFTFPSEQLKQQAIASGDYIPQNGLPVGVEHWGNRLFVTVPRWRDGIPATLTYINMDHSVTGSPALIPYPDWRANTAGDCANSITTAYRIKADECGRLWVLDTGTVGIGNTTTNPCPYAVNVFDLQTNTRIRHYELRPEDTNANTFIANIAVDIGKDCDDAFAYFSDELGYGLVAYSWEQNKSWRFSAHSFFFPDPLRGDYNIAGLNFQWGEEGIFGMALSPIKSDGFRTLYFSPLASHRQFAVSTRILRDETRVEDSFHDFIALDERGPNSHTTSHVMSDDGIELFNLIDQNAVGCWHSSTPYSPQFHGIVDRDDVGLVFPADVKIDENKNVWVLSDRMPVFLLAELDYNDVNFRIYTAPLSTLIEGTVCDQRNAIYGPPNAVSIPKPNPLYNKQYSAPLPQPKPPVQGTYVHQVAHPPPTHYLPSHVSQKVDVPKTFIYNQQNGLSYEASNGPHLFPPIQLVHHSVQPDGLKNYVTARSSNWWRNHQHRHQ*

>Woma_00016303

MRTFIIFCLLAVACADKLGYNYQPVGHSDNGLSFHPGGLGTGVSSNAPSYSAPSYSAPSYSAPSELEKEFYTFSAPEGDFDDANAAQKIAGSVKQGLRVVFIKGPENKGLEDAALALAKQAADQKTAIYVLNKQADIGDLANRLNALSRNNNNKPEVHFVKYRTPEDAANAQKAIQSQYDSLAGPSQSHNGGVAPVLNFASQAPIHTPTVQTPGNSYLPSSVLRRRI*

>Woma_00016304

MRTFIVLCLVAVACAEKLGYNYQPGGNPNAGLSGSGLSGIGTGASYSAPTYSAPVEFDKEFYTYSAPEGEFDDADAAQKIAGSVKQGLRVVFIKGPENKGLEDAALALAKQAADQKTAIYVLNKQADIGDLANKLNAINRNSNNKPEVHFVKYRTPEDAANAQKAIQSQYDSLGGSSQSHNGGVAPVLNFASQAPIRTASVQSPSSSYLPSAIFRRRF*

>Woma_00016305

MRAFIVLCLVAVVCADKLGYNYQPVGHADAGLSFQPGSGLSGGLDTGASYFGPSYTAPAEFEKEFYTYSAPEDEFDDADVAQKIAGGVKQGLRVVFIKGPENKGLEDAALALSKQAAEQKTAIYVLKKQADIGDLANRLNAINRNSNNKPEVHFVKYRTPEDAANAQKTIQSQYDSLGGPSQSHHGGVAPVLNFASQAPIRTVSVHSPSNSYLPSSVLRGRF*

>Woma_00016308

MRAFIVMCFVAVACADKLGYNYQPVGHSDSGLSFTPGSGVGSSLSGGSFGGSLGGGLSGGSLGSIGSGLGGIGGSSLGGIGGGSLGGIGGSGLGGIGGGSLGGLGGGSLGGVGSGLGGNNGGISYDAPSYTAPAELEKEFYTFSAPEEEFNDANAAQKVAGSVKQGLRVIFIKGPENKGLEDAALALAKHASEQKTAIYVLNKQADIGGLAQKLNAINKNNNNKPEVHFVKYRTPEDAANAQRAIQSQYDSLGGSSQSHNGGVAPVLNFASQAPVHTPTVNAPANSYLPSSIFRNRA*

>Woma_00016306

MRAFIVLCLVAVACADKLGYNYRPVSHSSSGLSFTPGSVSGGLTTSYGVPATSGFSVGNGVGGGVAGPVSYSGPNNYVQYEKEFYTFTAPEDAFDDKEAVQKFVGSLKKNIRVIFIKSPENKGYEDAVLALAKQAAAQRTVIYVLNKQHDIAALAQKFNAINNSHNSKPEVHFVKYRTPEDAANAQRAIQSQYDSLGGTSQNYNGGVAPVLNFASRAPIQVGNIQVPNQEYLPASVVRSLK*

>Woma_00015343

MEKSLFIKGFHVICILLIIQNLTFAQIVFPNEEADVPNFGRCITPNRERALCIVLEDCKYLYNILVTSPLRQSDRLYLSRSQCGYQNGKVLICCPDRYRQVTQPTLQPTPPPMYVNNLLPQPGQCGNVLSNRIYGGNRTKIDEYPWMALIEYTKGNNAKGHHCGGSLINTRYVITASHCVNGRAIPADWRLTGVRLGEWDTTTNPDCEVDVRGQMDCAPEHLDIAVEKAIPHPLYNPSSRNQVNDIALLRLERSVLYTDFVRPICLPINANLRSATFDGIVMDVAGWGKTENESVSNLKLKASVEGYTIEDCRSVYMRQNIILEDTQICAGGKEGVDSCSGDSGGPLISLDTTNRVRAYYFLAGVVSFGPTPCALEGWPGVYTRVGNYIDWITDNIKP*

>Woma_00015342

MLNSKIRINLNIYLTILLVNCATEVSSYHTHKNFSLCNCVLLQSCESFNNFIDNSPNDAHIKAVIQEAVCYFDGFNIFVCCPNKLNHKRYIKEKPKLDYLDELTGLNCPPSFGDEYNYDDYLKSHHEIEPKFYEANHHNIPQYDYDAVNNHHHHHHHHNTHVLCDELGNCSNAFAFPHDGNEYLTTSQQYPAYHHHEHDRECDHHTHIGQDMQVPFFPHSQNTHHNPVDPPERLTPKPLTALNSKCGMLPILSVSSMEVYPWITRLAYLNTTSRTIRYRCMGTIISAQHILTAAHCVDQLVHDLRLIYVRVGDDTNFDDFKILETIVHPNYNEPLFNNDVALLRLAITSQAKGPLIQICLPQNNNTVSTGDTGVVAGWSNNGSSTLKSSTIRYINLPVMNNTECAIRYAKYSENFENSIVITPTEFCAQAQPMNDVCEGDSGGPFMERSKAGRYTLLGIVAFGPKTNCGQSNLPGVYMRVSSYVEWIISNIK*

>Woma_00015345

MAKSLLIKGFHVICILLTMQKSIFAQIIFPNEEAEVQYFGRCITPNRERALCIVLQDCKYLYNILLTSPLRYSDRLYLSRSQCGYQNGKVLICCPDRYRQVLQLKVQPTSPPAQKYVNNLLPQPGQCGNVLSNRIYGGNKTKIDEFPWMALIEYTKANNAKGHHCGGSLINTRYVITASHCVHGKAIPADWRLTAVRLGEWDTTTNPDCEVDVRGEKDCAPEHFDIGIERAIPHPVYNPSSRNQVNDIALLRLERSVSYTDFVRPICLPIKANLRSATFDGIVMDIAGWGKTEKKSASNLKLKASVKGYSFEVCRTVYMRQNISILEAAQICAGGQEGVDSCRGDSGGPLISLDTTIKIRAYYFLAGIVSFGPTPCGLEGWPGVYTRVGNYIDWIIDNIKP*

>Woma_00015359

MLMLKILFCALFIALANCGVIGPYGPHYAPQGPLHYGPHAPYTPVAYAPAPKPVVPEPYDPAPKYSFGYDVQDGYTGDSKSQHETRHGDVVKGSYSVVDPDGTKRTVDYTADPHNGFNAVVRKEPLAYKAPAPLTVVHAPAPAPPLPPVPKAPAPVAIAVPLHHAGPAPAPAPAPAPGPAPHYAAYPALHYGPYGPGPAPHLGHYYHK*

>Woma_00015324

MSELSLANLKILLSKNSLKQLMEVSNAQAVKTTQVSVTFANVSTNNAASPPATILENLPQSIAPMQQQEITEYSVPFHHNLPQNFTAMRSFAPVLTSVVFYCPTIRANLYAFFTTHQYVPTPNSLPCT*

>Woma_00015425

MIERRQMPRESFDDFFSEMIAMNARLSQPLSPEEMIGLIKNNVRESLGSLLFATDLFSLDHLRDTARRAEKYITRQQHIRFQRKQISELETNKDIEETSDNDEEIEALKYHGNQKRERKQLDTSRFKCWNCDQMGHSFYDCSSKNEICFASDVVKRT*

>Woma_00015386

MEILLQMTDFNNVTNENNREKDAIDKVSTKSDDEDRSECEFESADDNENLAPISRGPARPRILRSGGPGRPKKEFNVLGLIYSEDDIKVPETVEEELSGKYGDEWWKAMQAEYDVLLKNCTWELMELPVGQKAVSNK*

>Woma_00015391

MEILLQMTDFNNVTNENNREKDAIDKVSTKSDDEDRSECEFESADDNENLAPISRGPARPRILRSGGPGRPKKEFNVLGLIYSEDDIKVPETVEEELSGKYGDEWWKAMQAEYDVLLKNCTWELMELPVGQKAVSNK*

>Woma_00015415

MIINNDNNNDNNNDNNNDNNNDNNNDNNNDNNNDNNNDNNNDNNNDNNNDNNNDNNNDNNNDNNNDNNNDNNNDNNNDNNNDNNNDNNNDNNNDNNNDNNNDNNNDNNNDNNNDNNNDNNNDNNNDIIMIIIMII*

>Woma_00015461

MNKDIILSACLAAAQTINIEAAVEELLEEERSGYYMLKAEHKKIYTSKSGQNVATYVSKYEYFASLHFLDTVRNRGSGGTESNFESLTNCEDWDDVEEQFQNSEDQCSLASAALENSTFAKRRKVQEVNEIDNKRSEVYEKLGQFLSQKVS*

>Woma_00015532

MQLFVRGLECVETLEVYQDANIADVKAQIAQLHEYDTEEITLNCEGNALANETPISALSSFELDITVPIMGGKVHGSLARAGKVRSQTPKVDKQEKKKKKTGRAKKRMQYNRRFVNIVPGSEHRGPNSNSKL*

>Woma_00015520

MSIMLTVNGDTTLLVANYFPPIQLNENYVCGLISFDTYHSIPNVDIENKLFHIGNQEIEITIGSYELSNIADLLTLKYTQLEPKENLCIKANYNTLQTHITRSKDPINFNKNRSIGSLLGFSKQVLKQKEEHYSITDQDGNLVNFRGERITIRLHLRPS*

>Woma_00015606

MKTFITLAVLCFIAGAFAADLKLSDEQKAKVKEHFAECVKQENVSEEDAARLHNKDFANPSENMKCFGTCFFEKVGTLKDGQVQEDVVLEKLGPLIGEEKTKAVLSKCKDIKGDSRCDTGFKIYQCFEQAKTELNV*

>Woma_00015604

MKTFVTLAIVCFIAQALANAVELSEEQKAKAKEHFDACVKQENVSEEDAARLRNKDFADPSENMKCFGTCFFEKVGTLKDDQIQEDVVLEKLGALIGEEKTKTVLNKCKDVKGEGRCDNGFQIYKCFEDSKQELLA*

>Woma_00015605

MKNFIVLAGIYFTAITFTEASKLTDERLFGSEELSVLEKKDILGEHDNKQISEKHAVIKGVVNGENDSETGTNTRDKTLKKNYNFIKPLLRLIFQICVKLRIVWLEEELRKFVIGLVYTIL*

>Woma_00015603

MKTFVSLAIVCFIAQALATPIELSEEQKAKAKEYFDACVKQENVSEEDAVRLRNKDFADPSENMKCFGTCFFEKVGTLKDDQIQEDVVLEKLGALIGEEKTKTVLNKCKDVKGEGRCDNGFQIYKCFEDSKQELLA*

>Woma_00015602

MKCLMILAFICFIAYCQASLLDLNEEQKAKAKTYFEECIKQENVSEEETTKLRNKNFADPSQNLKCFGTCFFEKAGTLKHNKLQHEVVLEKLGSILGEEKTKTILEKCKDVKGEDRCDTGFKLYECFEQTKTELVAA*

>Woma_00015649

MKIILWVLLNVADLTSTIGGSSALTVPKEFAPRVLFTISSTPFKCPNSHNHVSIANTQHTKPSSSKRRLVAAKVPQAKETEEGPTTKENDKPLTSLNETHIKEVTPISVANNVSLGLDISPNKQKEYNEE*

>Woma_00015644

MKIILWVLLNVADLTSTIGGSSALTVPKEFAPRVLFTISSTPFKCPNSHNHVSIANTQYTKPSSSKRRLVAAKVPQAKETEKGPTTKENDKPLTSLNETHIKEITPISVANNVSLGLDISPNKQKEYNEE*

>Woma_00015700

MRQSIFYPQLAYLPRPAMNVYRQNSFQRNQNRNIYDSAGNNFQRIQLAPPRPSMPKPQERPEPMNIDVSTRSNRVNYMNRPHPNDLYGKRPAPPSFRSNRNKIQRNYHYNYHDVNHTTDAPCDEECPLTNTEFEQAQRYFRDVPKEVATLEEQPVDFNDLHFLG*

>Woma_00015706

MKHCNEQSKQLITLWQKKESIRCVQVRRTLLPCSNDDISIEKENFITLAIFCVVKHSAYGIVMKNYSHNYVKRVVLGSPEYRNKFKRAKAWAEISAAMNVNETLCQLKWKNLRDRYVKLHQKKLQSGSESIVRESSWNLMSHLEFLSDHIQPRKIVSSVPVVDSLSEESTLFPPMEEIS*

>Woma_00013218

MIFIDKLKIYILCALSLVGYIQARSISTYQFGDIRIELTNNKWLCDKIECPADTYRCYVSKTNEKDPSVLVRTDICYSKDNKPLAEDVTSTAVDPNSKIKLVIDSDRNGVTTTSNYDSNQKIDEEQLKKEMAQLESDLENMHKEIQANLQEEMKHLEESLSDMQNGLAHMFD*

>Woma_00013217

MWKLIFRCVLIICVIFKDISSRPAEDNLSYIEGLNIQLENDKWICGDVDCPAGSNGCKIRLQTDADNKKLLLQSFTCFDENKTDLISAADVIEMPNEKSIDIDIESYKDAVSVYSTGFGFGGHESAKGIVAPGQWNNFKEESKRNRQGVGIE*

>Woma_00013223

MFKYICLIVLISSIYCAPLNNAVEDVEPVRKAAVEVSSHAVEEHVQDADEKKLTTKPPKVVEILESGLEHQTDGSYSFHYRGADGSFREETAVVKNAGTEQEYLEINGAYSFFDGDGKEVIVHYKADDKGFVPVGSNIHDAISLAARENSQLPAVKEDTDDDDVEQEVRSDEENVSKKKEHN*

>Woma_00013206

MAIPFYENEEGNQQQHNAEEVDQVDGPVLSSQNMSAAPQFLGQEAHHSNLSETLSENDLQAWKILALAMCKALKDFYQQNMSQSSSQLTSTAIIQILPNGEVKPKWN*

>Woma_00013209

MDFRQKNINFFNRITVLVVLLIPVFGLPDTNIANKYFLEKQKFLFEIVHKIEEPLQNEEWLNRGKSLVWDKSCYMEFNTLMEKYASKVNSSTLLPLNENFILTDSNQLEELKGLYYFLYNAQDFETLRQNICWARMHVQPQMFIYALTQNLMKREDFKDLNLPKIYEIWPQHFFADKYIRNLKHFNYVQWSKAEMYGFKDPIKNDTLQSTCQPQQWWCKENLSFKIYRENERTLWPKNIYGNFRNYTKWPQVLEDVSIYWLPVDFSREVAAPKELAARELTYLTEDKSWNSYWYYINMGLWLNERGESDLYSKPLRDWWYWNLQQIVARYKIESQRNFEHTTLNPYLINFQGKKYQSLDIIYSKNMQRFLKDLVAATEKALYEQTYELKNKTRLQLNNPIHFEYFLNDNFNIDKVLYAFMSLPNKQSSLTVLQNFETMLRAPEFYQYAEDILKLYQSLKSNFEPYKSQDFQSAGVVINDINITELTTYFEIVDTDVTNLLRSSNIYFESKLLWFKSIMARQANLQHEPFTLRFNLTSDKPQAVLVRTFLAADNRDDPVHFQMDTFVSTLAVGYNIIERKSKDFYGFMSPPLTYTELYHFTNLALNEEYEFPFNITTGNCRFPHHLKLPKGHEGMGRAVKFVFVVTPYNFRFHKGYNLDCDFSSGILAFDDLPPGFPFDRDVAENVYANENVLVKKMRIYHDEKVRFR*

>Woma_00014003

MFLRRQNVILIVIYFAFTIHTVYPSKENIKILLNYLNNETSTTLSPINCGEEAKCGKSHTNLTNKLTIENPIEYLKNVFDNEHFEEQEDESIEGIKTQPLDKITIKDDAETTTVVTSVNTNTQERSEDITIIKSDTHISNTTATSTENILTSNEDNISSTENILSELVPLTAYSDGELHIGQTIELQRSNTVAENEIVNEAGVAILKNMSVNCGSVLEKRNSTANGPVERTGVRLGEHDLSSERECRRDMKGKEICLTTVDAKVVKIIIHHDYSHLSFKNDIALLRLEQDYLSKQEEETIEGIKAHPLDKTTIKDEVETTTVVTSVNTNAATNTQERSEDITIIKSDTDISNTTATSTENILTSNEDNIFSTEYILSELVPLTAFADGELHTGQTIKLQRSKVENEVVNEAGVAILKNMSTNCGLVERISYPEPVFRTKEIYHCGGTLITTKYVLTAAHCVAQEFYELTGVRLGEHDLRRERECRQDIKGNQICLTTVDAKVVKIIIHHDYSHLSFKNDIALLRLEQDYLSRIYFIFAVRTVYPRKDNIGILSNYFNNEISTTPSPVKCDEETNCDISHTNARNKVTNKNPIGYLKSLFGKENSGEQKDERIEGIKTQPVDKINNNDEAETKTVITPVSTDAAVYNQEKSKDITTRKSVKAISNSMAMSTENTLTSNEDSMSSTENILSELVPLTAYADGELHTGQTIEVQRSNADAENEIVNEAGVEILKNVSSVCGSLLGKRISFANITTLMEFPWTVLLLYNSKDIYHCGGSLITQKYVLTAAHCVAQEFYELTGVRLGEHDVSKERDCELDIGGDEICSTTVDAKVVKIIIHHGYSRLSFKNDIALLRLEQEYFGRSFYPICIPTPEMTYTNTMAIVAGWGATEKALRSNVLRKAEVPILDMSKCRSIYRYSKFNDTTEICGSGVDEVDVCQGDSGGAIFYEQYIKNKLKHYQLGIISLGVRRCGDKRFLPAVFTKLQGFYKWIENNLEY*

>Woma_00014010

MKNHTFKYMMFSLCVIIVLLANCCAHNENYFKNELGYCEPLTVEFIAGLVTKQARQLLDPQPLNKQLRFDLYTRKNPYVKQMLYTGDEPSILRSNFNVMWPVRISFHG*

>Woma_00013313

MVLRGIFIIWLLLFGHLSWVFAGGYGVNVNLTDFLKHRRQKRGLIFNNGGAAKFVIGPIQPITLADPIVWRSLICSYNIHVGNYKIPTTPLYPWDKWENFYVRSAGREGRAQLKPDESREFLYLVLENYMTATHGHGKECLLRAICENAQVHHHMDLFAEILNVILTPGKGDVDAVYSQAYEMGKAGTDCFRYFSQCPKGLNFLDEFIQSL*

>Woma_00013346

MFNRRYFMTLCFMVCAILQIVTADEETTEFATYEEVKDIPNHPEKYLIEVRSKDLVEKDGSIPGSINIPFNELEKALMMNDEEFLLTYGAEKPPKDAVVIFSCLNGQYAQMGAEMARKNGWKSAKPYLGSWTEWSKREGLQ*

>Woma_00013366

MDDEKLAEPEEIYIAVPWGHIAGRWYGNRNVRPLLTLHGWQDNLGTWDRLIPLLPYNIGILCIDFPGHGRSSRYPNGIVYHMIDYACTIMTIVKKYKWQKVSLLAHSMGGVVSFLYATLYPHMVDMLIELDIIVAPIRTNDYLLKRLIIGTEKLLIANERLMDTTEREPPSFTYEELQEKIFLGSNKSVEKKFSEHLFKRSTKPSQLFNGKYYFSRDDRIKYYHEFNASMALIEDMTKRLVNVNYMVIRAKSSNYVTDEMLNVVQKIHTKMEFYEVEGTHHVHLNNPERVAEIIIPFILKNRPDMANKYNNSLTGSVISKL*

>Woma_00013362

MKWQLFLIAVITTVTQHSTTYAKVTSNYGQAIERNLFASDFYNAVASEKLNDNVVVSPAAVQVSMALAFYGAKGKTATEMQNGLRLGSSDPEVVVRQFGEFQATFPRDNNIRLANNIYINENLEFKQKFKDIAQRSFESNIEKADFHPPYNKRTAERINKAMEAKTGGKITNILAPEQLNDLTEGVIVNGIAFSAPWQKAFRLDKTSKRSFSAGRQSFKVDTMWTLNNFNYGEFSNLDAKAVEMPYQNTDFSMVVILPNRKDGLGDLLKNLKGKNLAAILDEGLSNQKVEIYLPKFRVTFGVSLKEPFEKLGVSTMFTRKGDFGNMYRMFVSHYINSANHKAFVEIAEDGSEQPLEAGGLKNIFSRTKKFEADHPFVFCIKHKDSVIFMGHIANYAYA*

>Woma_00013950

MHISYMKIAIYALTFLTYAYSGYGSSTIAHLRDAIIAAEAIFGDVFKNLITVLKKFHTVQEVFDAAVDENCVFKCPTLEDGPEVRPVQNKLYTPMADGCGSLGLRISTEYLPAIEMEACCNAHDICYDTCNSDKELCDLDFKRCLYKYCDSYEKSIASELMVKGCKAAAKVLFTGTLTLGCKSYLDSQQRTCYCAPPKPQRDDKSNYKNGYEGDKYYKAKKQKYGWKASNEI*

>Woma_00013946

MTRNTEYLSGRGNVDSDNTEWLSSTSTDIPALLQDPESNDELTELDEMNFDDIGILQEDALEYILGYIIRKKKNMNPMKIRLHGLIRCHRRNLHHHLIQESLSGTTFRQKSANTKSKAVSTTVFLS*

>Woma_00013949

MDFLKFLIILICYLTTFAQRTMAQYGGMGYFPYGYNGIGGIFGRRGSTYPTGIGGGYPSYGYYAYPYPIYGNYLCYQNIYCSGGYGGYPYYGNSLNTAFASSSSRSGFSTAYASSGGGFFG*

>Woma_00014739

MKITLAVLFLTGLTIYCKCVAVLSLPPSLIAQAAASLDDDDDIQKSQKPSTLKHNEIPKELQEQQFGRQQANEETSQEVTTDFYRNFVNLWPNNLSFFEQKRERNTFDDDIDNVDDVAIKNSSLQQQQQPIEAIKQNSNNIRQKSDLRLYDDTNKHKNNNSSNNSNNNNNNKNENMLLKVSAAILNNENIPTGHYDDTGLTNDFLDSSHTGKKSVTKREVLITPTSPELEILNSYLKPSKYWAGRCSLENEENCLQEYYGNLIADRNSALKKQLQFKQNIQAAQHMNNLNDDDIVLLLTAEQNLKKFLSWALSELYPFGKFKNASTDSEAADFYHPGMYLWKKLNLSGRVEPPLLVEEPQYVLVRRELNAEDDENDYKRGLNYGRDPFIPPRGRKHNLPDLDSLLHRYETFVPNRGRRDKIKDIFKYDDLFYPNRGKRQMIVTPTVTQSVIDNNNNNNGDIDDSQDDIKNYNEENANEQQQFPDPLLSTNELIEYIENLTTKMKRKLRQNQKENNEKIANSFDEMMSNGKEPTKMSTTYQEASVSWPMSSSSLLNKRFKSAFNILHNRQQQQHRQHQLQQHLPKHYTNRQQWWKRYATTAATAAANNNDRLLSEIRSPTMATNRLQTLRSMSPQQHLTKLYEQQQLPVDLSPPHVNGAQQFDPLSWHKLQMLLQQQQHSVQQQLLTMRHPLLTALQHLPLIQQLEEHVATPQETQLTYASDLFDHSITIDLDLNDNKMDLY*

>Woma_00014402

MKVSLAIFIALFALVSAEYKIRNQEDLMKARKECMETKKVTPELIEKYKKFEFPDDEITRCYIECVFEKFQLFDSKDGFKNDNLVAQLGQAKENKDEVKADIEKCADKNEQKSDSCAWAFRGFKCFISKNLPLVMESLKKN*

>Woma_00015626

MLLIKLFLISSFCLLSLSQRVQCEKTENSAVTGDGSVVAVAGKNNRKDTKVTAAAQSTIYKIKRLDPSKDKPNMHKQRIEKEKQKKQQPLQHQLLDYQHNSPQQQQEEKHQLQQEQKPRQQDVMHHQEFTGNYKVTQDGFDTTVSSKKYRTKKYRKSSKNAHGLNRKLLNKLGFTKVKAPNSHNRKRLAIESRRHASPDDSHMFIIKLPPNMYYYTNPKPSTSSTSSSSSSSSNAIATTDKKNSLKENTSMAKDANGKKVSFPFNSNGKPGRIYHWNLPVIKKMLEDKHRFPQVAHIEPKKVEQLIDIKNIPTWSKPWENDSIEKSYNQESMISYKKSLKRKSPTYYAPASTVNKNSFNKYFSGNGKPKGFYVIKENQKKPSYYKNIIA*

>Woma_00014511

MKAITLFYGFVLTATIFMVDTARSQKVEPRRDDTYPPPDFLLKLKPVHDTCVGKTGVTEEAIKKFSDEEIHEDDLLKCYMYCIFDEMDVLHDDGEVHLEKVLDMLPETMHDIAINMGKKCLYPKGETSCERAFWLHSCWKKADPVHYFLV*

>Woma_00014512

MKKRVLIEAALTVLVFLLLMPISIFAQKPRRDEHYPPPDFIMKFSIIRDVCVEKTGVTEEAIKEFSDGEIHDDPALKCYMNCLFQEANVVDDDGEIHYEKIVRVLPDYLMDYVKHIMDACATHKPTGETKCDRAWSLHVCFKQTDPEYYFLL*

>Woma_00013514

MAIKKPYEAVNSNGGAPAVTTGNYIEDYSCCYDLYQNALSPLDDKRKQRSRLMKGIIATYRCVMRVCSLIRRYVKHLVEHKYFQQGILLAILIKTLRG*

>Woma_00013522

MRLLTFVVACLCLAFLAHADDDIKVGASYGGPGGPGGLGGPGGPGGPGGPGGLGQPPNGGYNNGNINGYYPPPTQMHQAATRQNAPPCPTNYLFGCQPNIKPVPCIPNSLRGGFGGAGAFSQNHPVYALPVPQYAYGYQTY*

>Woma_00013525

MKTFTLLIVAAVITIGSASPTGGEERVNGDGSVSPIPCPQNYLFSCQPNLAPVPCIPGNSGGQRFMGAYSENVPTIVNAPHGLSQHLLHYPNYYQHQ*

>Woma_00013738

MLNFQRIICIWICCVSCMFGTTQLSTLLVQAVPYEYFDDHDQSFYDLDTEQIQAKYDTRLLSQQMLPTENLKGFHNSAAMAAFNEDDGGGDGGHRSALDGRKQNNDGSSLYGSTEKFKSNDGSAATSVDDDDDDEIHNDWLEQQEETATMAASSTSTFTEPLDELHLPRAASCFTNGQKYTHGQKVPRLDNCEVCLCMDGEIFCWWEKCDKKSVKASITYDDFSTPYGESSTTRMQLNPKTTFKTTLKPIKKPKTRNEYYKYDDDDSAEIREMQQEYEGLKEQLLRDEHEYEQESADSHTQKQAKTRNMQMVKKKIYQQQQEEEKHKKKIKEPNQNRKQQEQEQQQQKQLKSNEHYEQHHHAHNHNSNSNNNNNRHHDKSSSGSSSKILNFPENLPSVLYYDYKTEEHHQHQHQHREQHLIHQKQKQQQHEKELKQRQQQQQQKLLQQQQQQLQGEQQYHHNNRHHEYGQDFHTYKSPNGLELGMIANDGSGPSTTFKYFSNGNDNIFNEDLRTDSDILPEPPTKKPKFLTSIADTMATTETPLSQLYRSSTVTTAAAEISTTPNGKLTKPPTILTSSTPLKKQILSPWDSDKPKSGKDNGNSKSTTNNESTEDLMDTEDQDDAFHRWLTSTETNNKNNFTLKETTSRNRGFDSSNSDYNGDERIYESNGFDTDAGLSSTIGNTDNTITAMATGSTKNTEAAVALIYDDIGAKANGMYENIQHFSDNNRLSGNSNEIINSNGYDSNYNSAFSQTFQNSNKNNNQLSTASGSSSGGLISSTTSSEDNSVASVVNIDIMLTASSAEAQQQNINFQTINQQQQQIEQQLQNFEHEKQMAKTHDKFMQPPHLISNTNDAAATSAVDNNNRLSFIENDNNSRKNKDSNFTNIVGGNPTKEIQNMGVNLPNNNNSSSINDTNNNSATSYSSSDSFKFKNSPTNTTSTTVVTSDTTSTTPPTLSTTSSPERQCNVMGTMYKIGDVLPQDTGNCLQCICIDGSTSDDTPRVTCSPHNCPPLVLPDLFDATGY*

>Woma_00013723

MRRTIFVLCLAIVGLVNAAEQDVLELSDSDFSSTLAQHETTLVMFYAPWCGHCKRLKPEYAKAAELVKDDDPPITLAKVDCTEAGKETCNKFSVSGYPTLKIFKGNEVSQDYNGPREANGIVKYMRAQVGPASKEIRSVEEYEKFLNAKETTLFGYFQDIDSKLAKTFLKFADKNREKNRFGHSSYSAVLEKAGETDRIVLVRAPHLANKFEDSTVKFDGKSDSDLLTFVKENFHGLVGHRTQDTVRDFQNPLITAYYAVDYFKNPKGTNYWRNRVLKVAKEFVGKLNFAISSKDDFQHELNEYGYDFVGDKPVILARDAKNLKYALKEEFSVDNLREFAEKLLEGELEPYIKSEPIPESNDAPVKIAVAKNFDDVVINNDKDTLVEFYAPWCGHCKKLAPVYDEVAGKLQDEDVVLVKMDATANDVPPEFNVRGFPTLFWLPKDAKDKPVTYNEGREVDDFIKYIAKHATNELKGYDRSGKPKKTEL*

>Woma_00013598

MHDFSTYLLVYILLLLANLPVKAKIPVLLFPPTAPTRTQWICGIGIPLDNLRFEAMTTGYVLKAEYFLPETVNQLRIKTPMAFERKLTSSSTKFENFLATEEEVPVRSKPSLWEGSKKVFTSSYRWSVYKGLEGLANRMGLSGRACVLKSICETAETPFHYYNGLFAELFHILLTPSSSSDELSTHSDNEYFQAENLGRSGANCQEVFKDCPRSLIDHFSNIQELLGGVMKIMG*

>Woma_00013482

MTNNKGLAAGKLQKIHGELETEKQWARLTITLNSLGSAVKSVEQWRTVRRNLKSRASNKARDRKRQQALTGNKPVTIDPLRELGKHVVSIIGNEYIEGSEDVTENVPKIKVS*

>Woma_00013479

MATLLCSMLIGAGFPAVVVSGVARANIVENNQRNEPYPYEITDINMDETREEVVQKSQTYKLRPLPDLESHLEENMAKVFKQKADEEQRIREEIEKKQLEELQLKPADKYHYSRSHAWVAIIENAPWSVQPRKTYVNENGDVTDEPPKARFIEPSTGLFYESDCKNYIIIDSVWNNSQYFVNRQTYSVIGEIKWDFRNTEDWEHLLQGEPIEMRVQVTGSEENIISSDQNIGEEKHLDTVRSWVSQLHIGEKEFEERFPELQKTVLYKGAKYERFSPYLQRDGKVMQLTLYEDDDYQQPTIRREYFKNRADLLVQLKFNYETMDIIEMFEKGRDDNLKLESPKELDFHSIQTNIVDRNVPTLEINVNGLTGQTYLGTAARASIAVKDDVKVNQLPVKSISVTSKDSKINDFISWLELKGKDDPQKTPKLRTLYSDTCDYSPSGILKIFEDLLPYNFEMPKEKEINCLHL*

>Woma_00013507

MMALVDANYKFLALEVGINGRSSDAGVFSQSKLKQKYDENFLNLPPPKKLPFSEDMFPFIIVADDAFPLLENFIKPYSRHNLTPEEQIFNYRLSRARRIVENAFGILANRFRILRSTINLCPEKASTITLACCYLHNFLSKKNSSVYCEAVRMKGLICKIML*

>Woma_00013506

MKNFKFFILNIYVFPLCFRFVMPTNGTLQAINTSICKHNDKSCGNEWITSGRLGSRDAVLVFRTSILSRRPKLSDISPNSFECEAAEKPDRFSKTLETYNENVGPSTSKYAHYISKTPVNQEPIEELSDEIDDKKKFLTKRPKNVLRIVRSGKKLYDSLSSSDNSISSEGDELDEIEKQVESVTVTGIGRIDDTDTAGDGEDDDNENKIEDSTDEYSPANGMPAVNLRLTYKFVQTETKLLRKIFARHGLTEAEENENFSILWTGVHMKPDILRNLAPYQRVNHFPRSYEITRKDRLYKNIEKMQHLRGVKHFDIIPQSFVLPLEYRDLVTAHNKHRGPWIVKPAASSRGRGIFIVNSPDQIPQDEQVLVSKYVANPLCIDGHKSDLRVYVLVTSYDPLIIYLYEEGLVRLATVKYDLNADNLWNPCMHLCNYSINKYHTDYIKSNDAHDEDVGHKWTLSALLRHLKTQGCNTESLMMSIEDVIIKSILSCAQSVISACRMFVPNGNNCFELYGFDILIDDTLKPWLLEVNLSPSIGVDSPLDAKVKSCLITDLLTCVGIPAFSPAMRAHYDSKWTRYRNVSSYRRTSSAEPASSSSSKRNGGSISNNAVGKQLTLEEKRIIRNVKLQNARRGGFVRIFPTEDSMIRYGNFLDTNTGIPMSTPILQGQGFQSMLLLHNYNQLLFNNLYGKQSIDVDTQDNNSFKERMIQYERALETETPLLFAAKQIEPKCEEEGKQLRKQVLKLINEGSELTQLQARQTFGLYLEAILKRLTQDPRDCHEKIILKFLNRVGGSVKAPIFFRNPQNIRVVSKARSAMVAKLLSDFLEQYNRDTEAYVDTFDHIGMIPSNLFVDFLTQAQEADLEAVLALNTSLTGTMPYLYNRCGLSVPPTPPIPSGVHGFLKALPSMVLSGSGARDFSKHDSSKK*

>Woma_00013563

MRRSPSNNSEKRAIPLAPQSLPNSPSAKNSVLVQDQYKDARFNGGRNTTKVEDFIAPILAYKAYNVSDPMALSVGMYQYNVNINLTGVNDEAYLDIAARTSVAGYTLFQKLEQKKGDIISGDGIPRKDIAYSTIVNILIEKRFKRIRFHLSNARTNRTLIEKDEFLTNEYSPVDINRTFYSSVLKDLDTDVYDWDLFPSPKRMKTDNNQSEKMVEINSFD*

>Woma_00013552

MGSFGSQSDGGIFHLSSFEESVISGDAAFPLRSNLMRPILGSNLEPSEKVFNNRLSRAGRLIENCFGILCAR*

>Woma_00013631

MSGSLVILVGALGLTSLEIYNYTCDTPYYNFPKFWTNSGLCPAGKIMHSDIKESLLSDAMQMNLLHVAALPKGSITHLRIHWLLELMRYEQFTQSGVPIYDFSVLDKFLLDLDVMNVYPVVEFMANLSDIFMKNPNRNDFLWENLSYQFGKHYLNIFGAKKVFNWKFETWNEPDLLTYNKLNFTVEDYLTYAQSLRLGLENAAEDHENGSHFPLRGPAGLFKSQSKHPLCWSVLDFCNTDLERCPIEVLTYHRKGTGENASTVITNSKILLAKIYEKYPKLYQLPIANDEADPISGWSTPREFHADVRYAVTLVNIILDHWKAKLTNYLFTNLESVSHDNAFLSYHPHEFTQRTLLAHFRMNNTNPPHSQFIQKPVYAALGLLSRLGDMASDVSIIETVTGKPLKVLKTINAGKNPFYFSWLIMPLEKTTYESHEKIILHQNYLCSNEVYGYILETIDQMNSNPYNVWRSFNSPAYPNATVRANMRRRQGLKLHSTGLLNNATLSVDANILSEPCLILMRVCSFLKSRLKTPENLLITPVTSNEVLLSWQEFDAASVQCLKTYEVWFKMNDTKSWINISFDWHLPFPSFHFAPLNGIIVNVLRSNYDAL*

>Woma_00013696

MQWWRYFFLILFSVGFCEFLADFIKLWDCRWPAVIRTKTYNAKETEDGTINVMILADTHLLGPLKGHWLDKLYREWHMRRAFQAAIFLHEPHVIFVLGDLFDEGDMVDHNDFKEYVRRFRSHFNTPPSVPVISAVGNHDVGFHYKIHPYFVERFKESFNNTGAQLYTFRKNHFVFINSMAMENDGCEFCENAKNELQNIAGKS*

>Woma_00013656

MDYSYRLLWLFGVLLGPIFFAVVSLQDIASPVFQNHKTKEWTNLDNITFSFDCRNRPVGFYADMEYNCQIFHMCDEEGNRIPHLCANETSFNQEYRICDWDYNFNCTESPKWFYLNELTYATEPPDEDDDY*

>Woma_00013855

MGSFGSQSDGGIFHLSSFEESVISGDAAFPLRSNLMRPILGSNLEPSEKVFNNRLSRAGRLIENCFGILCAR*

>Woma_00014361

MITHRIILFNLGLILLLSLISADLDAKEGRIIAECLERFGGPTLENAERLQRFKHWSETYEEIPCFTNCYLNKMFDFYDEDNGFNETNVIKHFSKAIYKACESKLIEGKDKCELAYNGFHCMVNLENDPFIVIEGMQGLNDDAKLAMKDCLHKFDPFEWQRFGDYSNYPVKEPIPCYTRCFVEKLQLFNHRLRKWSVVNMREKLGFPDENTNISGCLAMGHKRTRNSCAWMYREFLCYLMSRGITEIDNQL*

>Woma_00014362

MKLQIVLILVTLALVSAKYKIKTKDDALAFHDNCREELNIPDEIYDKFLNYEFPDHKRTKCYIKCWVEKMELFTERKGFDETNIISQFTYEHPNFLSSVRHGLEKCIDHNESESDVCTFAHRVFTCWIKSNRHAVRKILN*

>Woma_00014360

MKFCIFFLILIFSVKRILCNLENEKNNDILRQCLQDNNHNPEISTSDLLDKFKNYANWTQEEIPCFARCVVAEKGWFDIDHHRWNKQKIVDDLGENLYNYCRYEFSRPIKNVCTYAFKGLKCLKDAELNVFITYTHLLECINEKATSMSQLLEYYHFPNRERIPCLFQCFATKAQLYDENFNWNIKKWLKAFGPPRDENLANIAVCRVTDERRKTMNVCAWMYEEYNCWERFNYNTNGSVAYRKALRKIFGRKMF*

>Woma_00014359

MKKFMIVFVALFGAAVAQDSFKCPDDFGFYPHELSCDKYWKCDNNVAELKTCGNGLAFDASDPKYLTENCDYLHNVDCGDRTQLEPPISTPHCSRLYGIFPDESKCDVFWNCWNGEPSRYQCSPGLAYDRDARVCMWADQVPECRNEEVANGFACPAAGELANAGSFSRHAHPEDCRKYYICLEGVAREYGCPIGTVFKIGDSDGTGNCEDPEDVPGCEDYYGDLDLKSIRKSELLAGLNSEGRKKSKTKAVPSTSP*

>Woma_00013783

MENCGVSEPSSSTWSSPVVLVKKNDASCLILRAWQQAYMSLQRNLRSIIGESPDPKRPIVLCTGFGIFSDTSGYGIGGFLSQVVNEAGATIAARCQSLNSIIV*

>Woma_00013799

MFKNIFKISVVLCLFAFAQARHSTQIAAHLSREYSDEINTAPFVRVGNKLYHFGTSKASWFKSFLICRLLGGFLASLDTQEELTELSKYLVANYPIDRTWFLSGSDLDSKGDFHWYRTGESISFADWSSGQPDNKGGNERCVQLWFIKTKYQMNDVKCDHKSYFICEADNPKTIVVSIF*

>Woma_00013806

MKIQQNVQRFIIIVILICLTNFQEVRTACGDSEWECDDGQCIDDDNRCDGIVDCRDRSDEIAENCIHYHDTCQKFAFRCAYGACINGQSRCNGVQECADNSDELICTKKDDSDFQGTCDDGEIQCVTSKECISSTYLCDGHADCRDGSDESLPLCVNFGCVANGFQCGYGACIDSNARCNGTTECADGSDEAWELCNKPRTKNKLTTKPTVPTPTPNPTTYSKCFIPKTTQTQDMIFKLHPLNETIPAGSLVDDFETIHIECKNRQTLRGQPSLLCRNGAWFYDFPFCQGYCDGTLLSGVSIHPICDYRTRFVECPKNIKAGTEIQINCAYGYTRTPNNPQYIKCLPDGTFDKNPIRCKQHCGRIAPNAVPLTKNGILIEPSSAPWHAAIYENASSNFVYICSGSIISSRIIMTAAHCFWDDATWTLRNYSQYQIIAGRSSSNYTIEQNDYSQLVDVDEIHVPSVYQGKTGRQQDDISFIKLKSPLKYSETISSICLPKFVRNTGSKYVPSNRKGFITGYGLRNQLERLRMITLSFHDCNSKSPPDAPVATDKFCIYNNEGASVCRGDSGGGFFQDIRNEEDNEIWYRLLGVISNTPSTTNDCTRVSEDAYVAITNIYDMESKFQEKLENAIQEDVALY*

>Woma_00013816

MYIKIFILLINGLLLFSPICWAQSGYNYGRNEIGGGNIQATAATPIRPLTSGFSAAPTATAAFPTAPTAFSGVGSSAGNPSGPFGGFPAQNQRGIGSLAANSPTAFGVTGKRPNLSQRPTGAGATGAGGIYAPNTQFSSSGSGRGTGGGNAIDDDYDGDFSAIPGVPGVDYPIYSQVPQTSFDCAQQALPGYYSDIEAQCQAFHICALNRTYSFLCPNGTIFSQEVLVCIWWNQYDCASAPSLYANNAFIYDYGNERIPTNAGYQSSSTQQIGRPQTNILSSSAGRTPSTSTGLFSSQRLQPSVAGGGGNIAVQPSSLTGNPAPFGAVIRPASPTVGATPGGGGGGTGFNANIPETAFGNAAIATPTSKAREYLPPPSRRQ*

>Woma_00013805

MHRYYAKFVILILSLRMVYSDESSAGCPLYTWQCNNGQCINFEELCDGNYDCEDRSDETVDECLLRPCPSYAFQCAYGACITGNFKCDGIQNCVDGSDETQFLCNKKINYEEEIQGRCMFPSSMQCKTGECISNEKLCDGKEDCRDGSDETVEMCSFLKCPEYSFRCGYGACITGLTVCNGYKDCRDGSDEIASICSNITSDATTTTIRPDTHTTTALTTTSTTTALRPTSPTTTTTTTTTTATPNQSDNYCSSTTLTRGISSVATCYYQKREVSCLQGIRAGTNAMVSCAPGYISKHMKNNVTRLQCDLDGEWTRPKAKCIPECGMLSATQKDSVSKPWEVTVFRRAYIPIYEPICSGVIVSPKIVLTVGTCFLSSASGYNTDHVFYNLVEGYYNASYSSDDHLNARVHNISSISIKNIHKSQDLAVLVVVNYFQFSELVKPICLPTAQSNVFPFYTNNNLNIGRPIIRDTSKIYLEYLIASVEDKPNGRKTLTYVNVYHYIPFINREIKKYDYIL*

>Woma_00014121

MVKLAAQLFSNTTANSIIRRYPLGMDLYEPIQNAEFIQLVNDWFDVLNSNLSSYPGKEPFGQDLKNQLDKNMNSESPHTRSVMKSMENMGGNLGLEYKRERILSESDLDNTVRNVDEKLLVDDKCSLEKMC*

>Woma_00013890

MNVDDNIIDLEKEIGVDFSTIQSQHSNELLLSTPPNNSVGNTELTPCSSLYKRFPSLNVLITPNTLRNTMPQTEDVIGE*

>Woma_00013985

MMGKLILVFLASCSLIDYMRAEIVYAAVSGLPMNLTPPPADQMQHNFRPPLPQEGQLLRPQRSLYGGWHGPPIHPHPIGLGHITKQELITLLEAAKEAARPAPEPEPEPEPEPEPEPEPEPEPEPEPEPAAPPAGVPLTVSLPAFVPIQLSAMYTAPPPPAAPEAPAAPEEEPEEEEEEEDEEEEEEEEEEVEEEPEPAPAPIIKPYHPVYHPWSSRGLYGGRRVVQYQPRYAKKSRSRARSKYRSKSRKSKSRRSKSKSKSRSKKRNGRKLRSRKNNLRYAASQQSIQALPPPPFLSRPPAPLKLVPSNQLLSNWK*

>Woma_00013898

MPQNNVIRPSVLGNAGVQARSESPVQPEPASVSNVRNATPEFRTLIRKLITSILETDGQDLFKDVLHPKQQVMTDLIIDERYRNNLTDMDKVQDVVRSLREFKGNPAEFSSWKKSVESILKIYEPSIGTPNYFGILNVIRNKIVGNADAPLESYNTPLNWIAISRCLTMHYADKRDLSTLEY*

>Woma_00013928

MNEDCIFQQDNAAIHVSKQSKSWFNEHGISLFDWPACSAELNPMENFWGYMARKVYANNAQNEGIKTVTELKLRIKQVWEETDSDLLKKLMDSMPDRIFETIHNKGSSTHY*

>Woma_00013906

MGVCDAKYTFTSVSVGSYGSQSDSGIFRLCSFGKSVMSDTVPVPQSKPLSSATSDPFPHFFIGDAAFSLRQNLMRPYSGSNTDVKKTIYNYRLSRSRRVIENSFGILSARWRILHITIEMSPNNIDSTVLTCVKLHNYLMLNDHKRWYCPVNYVDRENENGIVLNGEWRNEIAAWGRRPLRSFSTHIRRSSQTAMELRDRLTDFFSNEGSISFEDRMINRF*

>Woma_00009295

MLTIARHILIASVVGVTLAGSLRLNEKLDNSKDGFTTEMAEDDNCMPGCPRIFDPICAVDERHPEEYKYFHNECLMEYDSCRQSRAWKPTQMLLCINDIDPVLRADCMRACPMIYDPVCATDGTNYEIFGNQCSFGMENCLASGKWLEVLDEQCGL*

>Woma_00009290

MAKLFLIVTLALFAFALAQHGHGSVGQQGGFEGHQNGHSHQVNEHGHQSGRPSGHEGHGRQNLGYGGHGQQGYGHGQQSTAHGSHHEFAERFAFI*

>Woma_00009283

MNCEKTILHILIVTIALLSLCETRGGRRGGSFGGLFGSWRKYKKPTSGGGSTRRIVSSSPVHTPITKAMHENKKSSASDKFQKTNSYYNNYNSPHLPAGGSYYQNTAALPGNAIYVAQPPPRGAEIGDFLTGYLTGRLLTMGFSSPYHHHVTHVYRDNNSANDQNINNSGSKVVIENGQPDTSTAGPPYTAPLETTTVTATSTITPTGIRETRSTSTQLPMDTTETPTPPPYGVICVPMLLNKTNEKGEQVQVERIVCYPAPAPPMPQKDLKAKPVEIAGDIIEP*

>Woma_00009281

MHNTGICIFLLLAVVSFKICDAKGGRGGGGGRSGGRSSGRGWGGSRRGSVSSSSSSSRGYGSSHSSGGSSWFHGFFHSAPQYSYRPVYIIRSGGGSGGGSHYTPKEEYSSSSDDNYAIGYTISRGLSGNFFSIFNMVRGISKYPDKIRNNEIIPATTTTESPVILYR*

>Woma_00009280

MHYTKICTCLFFTVVLLTICDAKGGRGGGGGRGGGRSSGRGWGGSRSGSFSSSSSSSRGYGSSHSNGGSGWFHGFFHSTPQYTYRPVYIIRSGGGGGGSGSAGASYYTPKEEHSSSSDDSYAIGYTINRGLSGDFFSIFNMVRGIAKYPDKIKNNELIPATTTTESPVATYSIHDVISTYPKKNFVYDLGDLEKTLKDTLDNLNKVMAEYEDIASTEVSSKSTTPATQAESKEN*

>Woma_00009285

MLIHQNPVQAAVVYPAAAEIRYVSQQKSHQEEFIYYDKPSHHLAVLRAICTKCKDSNMSLDPIDNSQQSSYSQITL*

>Woma_00009289

MSKRLIIFFALFAIAFAYPKAHGGGYGGGHDNSLDGGYGGGHQGGFGGQGGYGQGGLGGHQGGFGGQGGYGGSHGSHQGGLGGHQGSLGGHQGGLGGHQGGLGGHQGGFGGQGGHGGSHGGHQGGFGGHQGGLGGHQGVHGGSHGGHQGGFGGQQGGLGGSHGGLGGSHGGHGGNAGYGHGGYKKHGY*

>Woma_00011852

MKYFIIFLISISLLTTCYADCEVFGLLMKKGEAKTVPGKCSQVVCQGNRYDEYTIEGCPNAYGAKDCNFVPVDLTKPYPKCCPHWKCPEK*

>Woma_00011845

MNFFKFLAVLSVVFGLTSAGSYHHDHGHHGNDGGVAVHYSVVTEHKADHQAHEGHHGHDHDHAHGGHDNGHAIVHTWSYDAGHSAGGHDGGHHDAGHGGDGHADDQHHDHHAYPKYDFSYGIKDPKTGDIKSQKETRDGSTVKGSYTVKEADGTTRHVEYTADKHKGFNAVVHTTGHAHGGAPAAEHGHGGHGHDSHNHGFGQASSFIIVKKQEEKKH*

>Woma_00011813

MLILLQICWTVTPIWAAFKDFVIESEKDLQRFGMANGDTYYEDDVDDLEFYSEYQNAYDYFIDDSCGAYADMMTDPHMQFDYDINVEGNGGTIDGRIIKELLHKLAKDPRFRHLGVQTLEDFSVLGYENNFDSVIAGRPTSYDKFYEYDDQGNCNENF*

>Woma_00011812

MLAKQLILKILILTFLNAFNGTIWTYAAVTEPAMGTFNCSQPPNFKDFDIGKCCRLPAINLGQAVEKCHQQIKSLKSYNDKYPVYAHVCYPECIYRETKSLKDDDGDIKLENVEKFLKANVEKHDKMIVPTIVQSFQSCLTNICQHMQTKGIQTFSKLPDLGCSPYASMVYGCVNVETFLHCPSEMWQQNEHSCNLARSFVQQCNPLPHIPL*

>Woma_00011798

MEQPLQQQQSRSLKINSAMVTEDLYAPLSPCPIPDISDDELVSISVRDLNRTLKMRGLNREEIVRMKQRRRTLKNRGYAASCRIKRIEQKDVLETEKSHEWSELEQMHEDNEQCAREVETWKKKYAALLQFAAHNDIPIPPELETC*

>Woma_00011801

MSSFTQYLCLTALLLSLSTLQSSTAHVLDILRLNELYYPTGPEYPVSNLPNGDSEFLLWHLDNPINFYSEKYDQIYINTNGILTFNIEFPDYINQPFPFEYPSIAGFYSNVDTTGANESTSISIFSSQDPEQLEKVSELVRYAFDEKTDFEATDIVVATWENVGYFESKTDKLNTFQVIIISDDQDTFVQFLYPSDGLNWLQGENGPLGLPDIRAQAGFVSEDGRYFNLEGSGTENVRFLSKKSNVGIPGIFLYRVGPFDYEHNVQAPTNLDTLTESPLSETCYDAGQRVCSHLAQCVDKEYGFCCVCQPGFYGNGEVCIKDDFPVRVTGSLVGELNGEMINERSKLQSYVVTADGRSYTAVTPISTELGSNLRLALPIISAIGWLFAKPLNATQNGYQLTGGEFAHKSKLSFDTGEVLSINQTYEGLNYWDQLSVRIDLYGQVPHVRHTSKLHMAEFTEEYRFVSVNELHSVQAHVLEVPEENRVINFQLEQNIYFERCLTDHQNSLVGSTYYQKISKIMLDYFERDQALRTSILTVAGVTASSNVCTDGTAACGENMVCIPYDDNYRCDCLHGYAPQATETGSEICVDIDECALGTHACSENAVCTNNEGGFTCVCLDGFEGNGFRCLLNDSVADNIESSTPNTWGTVVNMEELPVYTEEQPVSVLTESQPQPQPSYPAYHNDECHRCSPYADCLEGRCECREGFSGDGIYCASNCGPDEAWEEGRCVKVLYEEPDIEPRCNFFGDCTCDDGYELMEDIQMCRYVGNFHMDHRAEDIVPCDVEFNCHPNASCEWFEVELRHICTCKPGYYGDGYSCAIIDESCAVKPEICDPQAECIYNENLGHSECQCRRGYDGDGYRCTLAPECLEDLDCGQNAFCDRDVCQCQEGFERDMSDLCVVPGSCGAVFCGANALCKYDKHQGIKYCDCMDGYEGDALVGCKSKPVPCNVQFNCGVHASCEPTEDPANYECQCNAGYNGDGYVCVEDQNCLNTPQLCDMNAACLSTNNGLICVCKQGFYGNGSVCIERQQHDSGFLLVSQGVVIVRVPLNGKNVRPISVASMAIGLDKDCVEGRIYWGDISAKKIVSAKYDGTDIKPFITEDIESPEGIAIDCISRRIYWSDSVKDTIEVASLEDPSLRAVIINKNLVNPRGVAVDPYRQKLYWSDWNRESPKIEVSDLDGTGREILLGKDSVTLPNSLVVLQHTGEMCFADAGTKKVECIDAYTKQIRTISNELTYPFGLVFTHDQFYWTDWTTKKLESVDTFGKRQKSMQIPFFGSHKMYAMTAVEEHCPQMDSPCQINNGGCTDGRLCLVNRKAPSGKSCKCTALSKLCTAPNPFSF*

>Woma_00011803

MEHFISTVVLILGLSYIINPRYTREVCVQIQEYCARHYGERYSEKMQKPKTYARSSEKCCIIMKDKSTQVD*

>Woma_00011848

MNTLRCASILIVVACGLVAGHHGHHHDGHGYSSHGIHSKEVGYAVETKHGHNYGNAGGHDGGHHAEHHSVHEYSGNEGHDAVHFADYEAGHYSGHNEGHGYGHDDIHYDHHAYPKYDFSYGVKDAKTGDIKSHTESRDGASVKGSYTLKEADGTTRHVEYTADKHKGFNAVVHTTGHAHADHSGHYDHGYGHGGHASSYTIVKKQEEKKY*

>Woma_00011849

MNSLRSASILLVVACGLVAGHHGHYHDGNGYSSHGVHSKEVGYAVETKHGQNYGNGGGHDGGHDGGHHAEYHSVHEYSGFEGHDAIHFADYEAGHYSGHTESHFGDHGHGYGHDDGHYDHHAYPKYDFSYGVKDTKTGDIKSHSESRDGGSVKGSYTLKEADGTTRHVEYTADKHKGFNAVVHSTGHAHAGHAGHYDHGYGHDGHASSYTIVKKQEEKKY*

>Woma_00011846

MNTLRCASILIVVACGLVAGHHGHQHVDHGYSSHGVHSKEVGYAVETKHGHNYGNGGGHDGGHYAEHHSVHEYSAKEGHEAGYYSAYHGHGYGHDDGHYDHHAYPKYDFSYGVKDTKTGDIKSHTESRDGGSVKGSYTLKEADGTTRHVEYTADKHKGFNAVVHTTGHAHAVHAGHYDHGYGHGGHASSYTIVKKQEEKKY*

>Woma_00011847

MRLLCTVSLLIAMCGLALAGHLYYGSHGSEEKEVGYSVVTSHGSKGSTGGDGGHGDDIDDGYDYHAHPKYEFNYGVKDTKTGDIKSQSETRDGDKVKGSYTLKEADGTTRHVEYSSNKKAGFTATVHKLGVANAKQHGSHGGKYSDDHDGKATSYVIIKKHEDKKHR*

>Woma_00011260

MGLRNFYCITIVLFTATICTSSSLRKDIKPANLYVVNETPLTVPVYSLGSIYPYYYYPQPQSPTNTGVVYQLSNNGGQYPQSHFPNTGLVYQLPGSAGQYPLWPWPNTPTNKPANHPAAPTTPEISSIESSDENTNINNNNPKDEIEVFY*

>Woma_00011288

MKLFTTTAALLCVALFSLSVEAAKREAPISSSYLPPKNGNGNGHASGPASNGYAPSAPAGGPYASAPAAPASRPSSSYGPPAKGPSSSYGPPPPSGPAASYGAPSRPSSNYGPPKKNSRRPSNGYGPPPASRPSSSYGPPKSPSNGYGPPSRGPSSSYGPPKSSAPSFGAKTPSSSYGAPSKPSSSYGAPSKTPSSSYGAPAKTPSSSYGAPSAPSSNYGAPAKAPSSSYGAPAKAPSSSYGAPAAQTPSSSYGAPAKAPSSSYGAPAQTPSSSYGAPSQGSSNGFHPVQPPSTSYGSPAKTPSSSYGAPAASAPSSSYGAPAAQAPSSSYGAPAASAPSSSYGAPAAQAPSSSYGAPAVSAPSSSYGAPAASAPSSSYGAPAASAPSSSYGSSAAQAPSSSYGAPAAVPSSSYGAPAAQAPSSSYGAPAAQAPSSSYGAPAAQAPSSSYGAPAAQAPSSSYGTPAAQAPSSSYGAPAAAAPSSSYGAPAAQAPSSSYGAPAAQAPSSSYGAPAAQAPSSSYGAPAAQAPSSSYGAPAAQAPSSSYGAPAAQAPSSSYGAPAAQAPSSSYGAPAASAPSSSYGAPAAQAPSSSYGAPAAQAPSSSYGAPAAQAPSSSYGAPAAQAPSPSYGAPAAQAPSSSYGAPAQAPSSSYGAPAAQAPSSSYGAPSGPSSSYDVPAQPPSTSYGAPTGPSSSYNSPSAPSSSYGAPQGGSAPGGYSNSAPSSSYGAPTGPSSSYNAPSAPSSSYGAPATGNGGNGGYASNGGNGGNGGYNGKGSNGGGYEGNGGNGGNGGYNGNGSNGGYGSNGGYNGNGSNGSNGGYSGNGGNGGYNGNGGYGGNGSNGSNGGSDGYSYSQTKVPDTIPQSYSTDGGYTY*

>Woma_00011307

MKLSLLMLVGLLALAPYVKADEEDISELDDLDLFDLDESDEELLRQLEEKNLHKDIERENEIATKLVAETNQVLNPVIEIDPCEKMHCGAGRVCQLEGVKAKCVCIPECPEEKDVRRRVCTNKNETWSSDCSVYQQRCLCDTKASGCLTPDNSHLHIDYYGECHAQKECSEEDMKDFPRRMRDWLFNVMRDLAERDELTEHYMQMELEAETNMTRRWANAAVWKWCDLDGDTDRSVSRHELFPIRAPLVSLEHCIAPFLESCDSNNDHRITLIEWGACLGLDASDLNERCDDIQKTVPHLLG*

>Woma_00011301

MKFFQFVLSLFIMCILLNTLTRASPVPTAPPTPGYGTGIDGNNTPYDGPPTESTPIATPPLVTVTALYPQK*

>Woma_00011259

MKIFLIFFALLFCVSGKPTLYILRDLLKDILQIPDAPKTHDPVAIQTIHSTYPPPYAYNTPENYQFQNYPCERAELRPVPPPSGPAGIYDNNLYIQDPPLQIDRVSFMISK*

>Woma_00011279

MKLTLAVLVICCALGISQSQLNSGFGLGIGPKPAFGGLSPGQFFQTIVLQAEAGKLLANPDLPEDLRQRVLDTMANAEQGFNNCSTIATLPWLQIRCAALQLQRSKNELKAIENEATARAQAAATAAPAEGSGAPVA*

>Woma_00011278

MLKSYMIGLVLNYLLATALSQNVTINTAADLEIQEAFKFSSVVTTAIKEYINDALPTAAKPEGEQILQTVQEGLEYCEKLVNIPEDIWQYKYCGSALLRDGMAALAALQAKYRPYSSSASRIKLFW*

>Woma_00011297

MKIVFVCGLIVALAQFISAAQICDYDVTYTSVFVPDCTGNDILIDWPYYQDFTKYYSCTGFNTPILNNCPKGKYFSYFLQQCTDCGRFLPPPECQNLKTDGTPKCVASGTTSTPAPVTKTTPALATKSTTIGTASTKTTKKPVIVPTPPAEPPTPATADPSAPTPPLLPTSAPGIPTPPVEPPTPPNLDVTPPSAD*

>Woma_00011291

MKYLICLALLASMAITTVLAVAGQSACRDPSEVGKTFPNHWDPRKYWYCETLNEVAIELECPDGEAYMHLLKACVPWPSYIWKKPENPPTVA*

>Woma_00011290

MHKVSLIVLLAVVAFASAGYIQPHYEPQDIYAEPNCAVVKDHSRMFRNINDPTHYWVCPEGKEKADYIQCPANEAFMETLQKCVLWSEWKWVEPYQK*

>Woma_00011293

MKYLICLALLASMAITTVLAVAGQSACRDPSEVGKTFPNHWDPRKYWYCETLNEVAIELECPDGEAYMHLLKACVPWPSYIWKKPENPPTVA*

>Woma_00011292

MHKVSLIVLLAIVAFASAGYIQPHYEPQDIYAEPNCAVVKDHSRMFRNINDPTHYWVCPEGKEKADYIQCPANEAFMETLQKCVLWSEWKWVEPYQK*

>Woma_00011299

MKVAGLLFATLSFVAVAQASYKLSCAKSELGIRWPSYESNSEYYVCPRLFAKQATISCNPGEIFTFALQSCTSPNRYIPPPPLEVLPTASPLLSKPNNEGYLTPLEIDNDVHPPVLGHFNPNVPDIIVSEHQMPGTVEIPKAPAVIVEEPKHEQLPALKEELPIAVPVETVQKPLPLAPLPPTPAPTPPVVEKDNHKKPHHGASGKKPSSDKKKKSSAKPSKNEKKPAISKNPKKPAKNPKTPNKKTPKPK*

>Woma_00009547

MKLTICQFGKLFLLLTAFLFVTKSLKTFVVDVAATNITSPFSTSTTTTTLIPTPAVLSQATTVTVNTTAVKASGILNLSLNANVVDAEQDIERKFKIVTQQQKQQQEQQQQQQLILAKSIITNNKVNIEEQLQEQEANKLKQQSTIKNIFKSILLNHEENLQKTALKGNENQTVATTAANALYKSASKAQQYVSNKILKTFHKNQTKNNSDVTDSRNINYVGNSKIINTPTTSKTTNENKIAILSTDTYVTLANRESLVPTHIAFEDNATATTTQNEFFFRQQKSKQQQQTYINSSTKDETLLRHSATAKSLKLPTTLPVSASIPLLVLNSQYNQEEYQPITYNSNQPSIQAQQLQQQQQLKQKIITKIAKSAATSTTNSTPITASSSSSTIPHRKRVGILMASRLLNKMHVQQGFNNFVEFFQVNLNNVNVDFIADDDLATFIRLLEQPNYTTLVKTLNAGINLDDDDLAEGVFPKPLQNHQEQHSTASHSYRNQKENGENDEKKSEKLQTKSVKVKAIEKINNENKKNYEDLQNQKHSTSYLSPTNDDDSQDVEQKLKKEWQQQQQQQQQQQQQHDKNLNIGYCHLAKVLERDFNKTVLLWPCPHMKVSTNGNIIL*

>Woma_00009534

MMTLNNDDDDDDDELLKFLLTHAESKEIREPIKQRNQKHWVIMSRFYGFSTLKWKQKFQETYDFHKSIIMSSVKE*

>Woma_00009578

MLTAFLPAAAALLTSTATTTYTTTSGVRQHLWPQLYTDQQLEQLEQQHQPETAFYQQLFAAALPATKHTMLPQLEIIDSVFMNTTTTPITETTTYLPSAFSHNVLSAPSKTTTNSPTNANPPQQSRPLPTAFPASTILMSPSPSSSLAWLAGTSTTASSVFFENDLLPAVFVNANTNNNIMNNSDNNENNSHISSDRNTTTLGLGGIIAGNGIDEDDDNLNSFYFYEVSQAYKWLAINKNSNNNKSNSSKHDKR*

>Woma_00009389

MQIKYIFCLAVLLVFMATTTLNVEAARRIKLRPLSAKEVIRILKAAGREDAIPEGRVVTQGIAAITGFALGLTKGLGGVMLLDLATSNATTEFINNFNYTAMMGDFQGINMTALIQMLQGSSATNSSGITYVTTEICFDTTVAKIEPETNTVARSLHGNSEDDCGDEMELARQAIITSPPATSPTNTGGTATDTNGATNTATGTGTETGTGTNIDTGTETNTNTNTITETGGGGQTCIVIQKPKLRKRRSMRGRRRRSPLAF*

>Woma_00009355

MTLKFLLMLLIPLTYSVLSQLVEDRFTASVCTANAGCMIGRLMYGFNIPFFEAFLGIPYALPPIGKLRFSSPVVYPKWEDTLNAFEAKSDCIQKNYLLPTHPVSGSEDCLYLNVYRPLNRSSSDKLPIMVYIHGGGWFSGTANPAIIGPEYIMDTEQVILVTFSYRLGAFGFLSTGDASISGNFGLKDQLLALKWVQRNIADFGGDPSKVTMFGQSAGAISAHMLMLSTQSQGLFHGVIALSGTANVPFAITETPLEQAMQTAELCGLENARNMTTLELLETLRNVDAYTLIRAGDSLKFWNVDHITNYRPVIESKDVKDAFFTKHPVDIMKSGNYQAVPFMLGTVPNEGAVRVVAIMESPELRHSFNQDFYNILQAFLELPSRFDNETLSKKMSKIINEYFKGEQELNDNTIKGFMDLVTDRGFHHGFYNTIKNFVHTINTKLTPIYLYSFNYTGPYTFSSIYSGGQTSRNYGVVHCDDLIYLFRSPLLFPDFKKDSLHAKASRLYVKNFVHFAKYLKPSSDVLLKKCNQMTFHRKPETICDYHLFENDRTGSSHILKTDNKFNVSRMKFWDDILSD*

>Woma_00009416

MLLLQVPCNHLQQMRHKPMWTFLFLLIIVVVFLDKTSANAETEPPQTTTPEPDASPGCKAPDIRFTVIKPETTTQQPYAKFITTQVYPPDDLTTGDVEYVERINIDLTGRHNGNQMGGIVNPYQVNINDHRAGAGIHANDMAAGDEQHDGDDDDVDNDGNDDEDDEEFTDTDEAAAKRKQMQARKLHEKNRRTGSGRRRRIENENGQTRGRGSRYKRQVIHHDPDASPDTDKWSGGKLAAEGDVYYIHIDDILKSQSPNEHLKLKLYKMKQKVRKTKKSNCTENHCPKRVKPLVKKSTGLEEKEKRKLAIKNHHRRRGDSHETQKDFKTNHPDLKNATTLSINPRKKRDLKQNIDYAHRKENDLDNDNDEVSQLTTKAISSERNERKLNETGPPLEHTTDLEDDARVYSNVSHNNNALQRVKRKSGKTTGALSRPKGGGDSSSKSTSRKDKGIFDEEGTHYPIHTDEGESDEEEEEEDEIDIQQQFTEVSEIRFPGEIGPLGDRRLCKIRCIKGKWVGPLCATNEEDENGNVKFQPLYKSCHVQRIPPYLLLSYRNISVNPITRARRYRTVKSNLISNTQINVGWDLPHGHSLQARCKDLGMYKLLGESRVLCSNGLWAPRMPSCVPTTLLTNFSDDSAPSIKYRIINGSGSFEPSGVLAVLPNSTILLDCIYPRARGIPDWSWTSWYMQYETGWTHEDKNFRYRLTIKQMGIGDSGTFTCTSPRGLTNSVAIVMATSTCPQLPEPALPLSLRLEGDKLGQRALYRCPPGFRVDGIANATCLASGNWSSPPPTCQAVQCPRLILDDPHLSLVELNTSAWGRAVFKCQWGFKLTGPPGLECDPTGVWSGPVPRCKAIQCPTPVAPLNGRIGGTNINQRRLTVGALITFSCNEGHTLVGEPSIICTESGLWSHPPPFCKSQCPYPGDPPNGLLAPLKFNYDSGDYISVQCRPGYVQYSENGPPERPKCQPDGNWSGPVPKCRSYEEV*

>Woma_00009395

MRLKLISLTFATLFIHSAWTEPVRRRYELKNDAAYPLRKLQGRVSNNNLPDNIVILLRFDQFSNLFNNKFGIPATTKATKKRTHKPKPSKYPPFYPPIYYTPYYPPIFYPPPYYLTYPTTKRPTTTTTITEKPTTTTTTTTEKPTTTTTTTTEKPTTTTTTTTEKPTTTTTTTTEKPTTTTTTEKPTTTTTTTTTENPTTATTTTEKPTTTTTATTEKPTTTKTTTTEKPTTTTTTTTTEKPTTTTTATKTTTTETPTTTTTTTKKPTTTTTEEPETTTTTRRSTHPTPCWPLPWCLFFPPLPLSQTIPGPNPGPIPGPNPDPNPGPIPGPIPGTHWQLVFDSLARKRSLKLNKTNGTETELIFKNNLKQNE*

>Woma_00009394

MIVSKCLGILFVAIMAATVAGKGNHQKLIRVRPITREQYESILKLNDGKSVISEARLINSTVTGLAGLAAGFQLSRLWPNFFGTTSVVQDSAVLGGYPLCTINGDQASGRQLEEPSYLDVDELTNSLVRPENGSSEHTLIHCILVLDDGKHRAENPATFIIPTTPATTTTTTTEPKTTKTTVVPDYPHYPHYSHYPPVYPSIYPGYPFYPYPPPVYPLPPAYYPITLPPYIQYPISNRNRNEQPQTVNSVKLRTVLLDKFPSIRGLTTTTTKAPKRRTGRRTTPGPQAYLQSQYYSPTDKKLRRQEPKVDLDEYDDSVDYIEVSKKSSH*

>Woma_00009405

MFKLGIFLLPILLIIVCFLRIANATVQPRAPNFQYFERPKYRYPYYDSNGRGKLLYGYGGPDLYQYKTYTPLEGIH*

>Woma_00009404

MSRKYFSNVAKLAFLLLFVLQIQHSESFIYRLLTETLQNNVAGEPITHERTNWDFDPEISKKRRSLFYETHGFRAAKLIERIGVGQDGLEEQRRFEQQQRDIGRLNGEHFINYPALS*

>Woma_00009407

MKVQICLLLFTLGSLASFSCAILNDIHDAIGWTNVDLVNFKYFKNLPSQDPKRAAQANLFLEHFQKKKAVLDYIENLFFGNSPFTQESEIPFDPHYGRAWRPYYVRKFGWKGERLIDALGKGYSIKQLRHFGAIPKDYGSKYYPN*

>Woma_00009366

MSTRNFPPSFWNSNYVHPVTATTHPQMSDLYSSDGGYATDPWVPHAAAHYGTYAHAAHAHAAHAHAYHHNMAQYGSLLRLPQQYGHSSRLHHDQQTAHALESAAAYSSYPTMAGLEAQVQESSKDLYWF*

>Woma_00010885

MNRYVLHVLIITILCYKALSVPLTPTDVPQDDEDDDWDDDDESSEADDDGRVYKNPRNSPSSDCPRDEEQATLLGQKCLRKCSSDEDCKSKKKKCLCDGVCGMSCIKPDRECPELAQPTLGQVTVGGRHFGARASYSCPHGYHVVGLQSRLCQADGNWAGAEPACKQNIYCLKPPQIEHARNSALPEQETFDLDSTVQYHCHTGYVTNGFPRAKCLAIDGQASWYGPDIQCEPRSCGQPPDPANGWHSGECYTYGCKITYNCGTGYELVGKHERTCQSDGSWTPKELPTCVLVTSVVCPTPENPKNGKATYTTLAYNSVVSYECRYGYTLVGESSSRCGADRKWSGTLPVCKEINCGHPGNLYNGWIENIDAGTGLGASIIFRCQPDMLMYGHASSVCQIDGRWRNALPECLAPCVVPAISQGYVLPIEIVIDENGTTIVTPTTTTQSSILLTANVEKVKHGTALDVKCDENYEFPVSLLSPPTCHNGTWSIIPRCVPARCKSMPKPPKHGMVLAPKTEHGMKARFKCKDGFKLVSPEGKDVMDPNDYVLTCSFGNWTGETPHCQEVFCSFPGYIPNGKVLLVGNMGLYDYRPYVKKIVNNKQIMYDCDKGYVLDIGPPGATCVGGKWRPLELPQCLLGQHPRLRWNRRRRSIQIRYLRSSYLMKHKRNLERSLRNEIYNHFPNWKNISAHEAHKSDKKDRIKRSPTDIDEAYLKYYERIKQKYQDFVKYLLGYSNGLYSSMDFRHPAKKFKVQNGRWYYINNDTEQLPKYKATHKSRNLINDYNLNEYDDLENKAHRTVGKIPIPNGYDNSQYTFLRRNNETNLLRSNSYNYISDTPARIYKSSNDSDFIEQLKAQIIRKRRKRSLLYPSETDSDTTDQFENKEGRKRLRGPCEDLDWDSFANITVVRPGKVPGRNSVGIILSLECNAGFKLNIKGENATARCIRGIWKPETPKCLSAPCLVPSVENGKYYKVEPHTTELSDKPSLTPLSTYEEIQSNEFITLECEEGYKIEGSAQLRCAHGSWSVKAFSECVSVACILPNITGLIYEGGYRAGLTIGHGSSVYVRCNYSSSTLPIEMTCSKGSTHPNNIHCETGMRKSREEVIPASIATESKETEDNSIELSHTLDSSDMEEESNSTDDHSATDESKMCGPPTMNEGALIYVNDKNNNKIERIYESGTEIYFNCIPSASGERNTWKIICENGQWIGRSYNCANGTCLFRNNEPNVVSFYNDLEIREDIVEFPPGATITSRCTDIGKFELIGSIERTCIHSEWTGTKPQCFGLNQENDYAMEKAPTILFRRQNGPIAQSNDGKLIVYPGTTLHMECLWMRRFGNPKWSVSHDYKNYSEGWVTEEGRDATLEYRLTIGDAQTDDSGVYSCETPARHKHSVEVVVKSINCPEIPTRRGLIVSTNETKLSTRVLMSCSNGNSLIGASELFCLPSGNWSAPLPVCESVECGDIPLPNNITSPRVSVLSREVGGRAAFSCSSGYGLRGPSEAICMPSGEWATPFPTCVEVQCENPGAPQNGYAQGQAPYRAGDVVQFNCNPEYMMQGQPIIACQDNGRWSGGLPKCVQACSYPGTAISGHMSSVQFYYAIGESITFTCDAGLELRGPKMVKCLKNGKWSGAIPQCVAAD*

>Woma_00010869

MVSLNPRLSVTHNGHNTWKLHISHVHLNDSGSYMCQVNTDPMKSQSGYLDVVVPPDILNHPDQNLEESITNEGGTITLMCSATGVPMPTVQWKREGGKDIILRTESRERQVVKSVESKKLVLGNVHRTDMGGYLCIASNGVPPSVSKRFDVHVNFTQA*

>Woma_00010894

MFAFFLLVYLSLSSALAVEIPTQLLINRPSKQTQLKAVKDLIYRTILEKASYFEVDINSNLEFRSFQILKTTANKIRITGYDGISAAKGFHYYLQNHLNRSVYWYNKVIDIGENIEFPYVNLTSKSASEIIYYQNVCTWGYSFVWWNWSKWTTHIDWMVMMGISLTIAPIQEQVWYETYSEMGLTEDEIDSYFSGPAFLPWLRMGNIHGWGGALGPTYRRIQTILQQKIIKQQRSFGMIVAIPSFAGHLPDAMEKLYPTSSFSRIDSWNKFPSSYCCSVFLDPNDPLFQNITDIFLRKIIHYYDTDHIYFADPFNEIQPKKADSAYLNLTAYNIYKSMHTVDNETIWLLQGWMFVKNIFWSNDLKRAFLTAVPSGRLLVLDLQSEQFPQYEITQSFYGQPFIWCMLHNFGGTLGMHGSVNTINERIGIARSMQNSSMIGVGITPEGINQNYVMYALVLERAWLKDDLNLMQWFNKYSDIRYGIVDDRLHAAWQLLRNSVYSYYGLRKIHGKYVLARRPSTRLKVWTWYNFTDVYLAWSKLLQANLSIPNTHYYTYEYDLVDITRQFLQITFECLYVNLIDTFNRKEIERFYELSRTMINVLDDMELILATHKSFLLGQWLEDAKNFATTPQEKYQFEFNARNQITLWGPSGQIVDYATKQWSGVVKDFFKPRWHLFLKQLHISLVNNEPFNNTLFAKKVLYEVEKPFNFNRLSTNKHIYALKRDDIFNASVVRVQSEQSVVLARQGFFEITPQELIFISSDHEPIAWALQHLRRYGLTGNIFSFEAGRRCRTGPGIYTFRCCNADQFYAKFQRCINSMPVFGERSNSSIHPSHLFGRLDSHQQNTNHYLEPTSVQLTSFNDERTLLTESISNGLNDFNLNSPDSLQSTSLTAEIVNFTSTAPSYSNISPNQNLFFSGSNIYMEQPMRHNQEHNNNISTQEGTEPLNSNKKHSEVISLLKSSSLDMPPDECAPIITTNGMESQRVYANIDSLVGTSTSQSLCTNSTDRCYANVEVSSRLDNALTPSVQSKSFQLDLVNEQLTQSTFNTPVVNYIVLDLDQPRSPSQCSPKNDIGRSHSLAGSSENMDMKKEDISSITTNAISCGSSTMPKGSTIHNDSGALSSMESSGSYTRIDFLKTFALMKSSTNYTDFEFDNDQEESRITRHSKFVRKAYSISE*

>Woma_00009480

MKLLQIGLPVLLILLLFADNAIAKRRRSSTTDENEYDVHHHHYDEEDISSSKDDLHAQESGRMEFNVHPDANGNVGRGKRGSQKYRQTALVKPVNRRKKHVLLKIQPRHKKPAHHNQVSESIKNFSTSPSEEVKVYHTFEETHEHIEEKDETPTPSADENVPSPEIQTSEATEQLEIQAEEQFNDPITEETQHYHSEEEKHEKHKKVKVKHHHHHHHHNHIKEIIKKIPEPYPVEKIVHVPVEKIVEKIVHVPKPYAVEKIVKVPIEKIVHIPKPYAVEKIVEKKVPYPVEKIVEKIVEKKVPYPVEKIVHVPVEKIVEKVVHIPKPYPVEKIVEKIVHVPVEKIVEKKVPYPVEKRVPYPVEKIVHVPVEKIVEKIVHVPKPYPVEKIVNQVVAVPRPYPVVQRVPYPVEVKVPIHVEKPVPYPVEKVVPAPYKVEVERKVPIYIHSKEPYKYERPKQDDSNQKHIDDDFIDHEHKGYEHSHEIEHSNHHSHDLSVFPTNHKPRVTTQPKVASQKQTPAIRTELLQQQIHNYGFKKHEQQHQIQLQQQQIKQQQQQIQQLQHQQLQHQQLQQLQHQQHQQLQNQQLQHQQHQQLQHQQDQLQLHQKSVTNSPAPEASPQESRQVEVSIPPKTSPKFQIKVEEIEPSASEAMSVSVPASSSDMQTQASTNSYQVVAPFPSIAIPLNILQYHHMPFQQPLGFSLAASDY*

>Woma_00009470

MAAKLSLFVFALVVANVYGQLSLDDLIGQVFTDKNAVESTTKVFRPEPTKTTVLNPPNRIETGGYRSCGVEKECVPRHLCVDGAISTTGENFLDIRINDNVCNYSELCCDIPNKRTEPVIPAVPKEQHDGCGWRNVDGVGFKITGGTDNEAEFGEFPWMVAILRTEDAGGEIIHLYDCGGSLIAPNVVLTAAHCVNNRQARQLVVRGGEWDTQNTDEILNHVDKPVREIISHEKYNKGALYNDVALLILEEPFVWQENIRPICLPEVNANFDYNLCYATGWGKDKFGREGKYQVILKKIDLPVVPQATCQNYLRQTRLGLYFNLHESFLCAGGEKDKDTCKGDGGSPLVCPIPGVKNRFYQAGIVAWGVGCAEENVPGVYANVPYLRPWISEKLAARGIPFNHFTPSDF*

>Woma_00009490

MKLICFSFVLAAVCVLVNASIIGHPTIELEEHSPPHYEFQYAVHDSHTGDIKDQFEHRRGEYVTGRYSLVEPDGHRRIVDYSSDPLLGFSAQVRRELPGATLVTGHAAVAPVIEEWPAPTHRR*

>Woma_00009491

MFKLFGLLLIGVSAVLAIDYPLLHQPAVIKPIVKAIEVEAPAHYDFAYSVHDEHTGDIKSQQESRKGDVVHGQYTLIDADGYLRTVDYTSDAHNGFNAVVRRDPLGHKVKAVAPVAKVLVGPPKFLAGPKLGLPLAYH*

>Woma_00009458

MIKYSNILQILIIIMVMKVVMCNANQPSYQVSLYVRLHPRSVPVHLCNGIIVESRLILTTASCVHFKFTSSNSPVVAIEPAMITAVSGSSTAFSDELITQVIEIFIAENFNYTTGENDLALLRLSDKLPLDTRNDISWIILDDAENYDGPCLANFYIRNSIHGAPSYIQSERLPLLDSKACWALSKYPGLREVDICSYYYLPAGFNLNKFETLQHYNGDRGTGLLCDNKLVGILSTILPPQNDTDNTEKPIKAFYTSLAPHLTWIFEIITAEDLKLLEEGDYISSSPYAGETNAFVQPEASEDGSTLALGTNNLTNATEMPYRRNSAGLSVKEQNYLSIIFVIYLMTQLFN*

>Woma_00009494

MASKIVICLLAIAHLAIGIELKHEEEHGPVAYEFHYSVHDPHTGDIKSQKEVRKDDKVEGIYELIDSDGHRRIVKYKADHHNGFEAVVLREPTDIKIPIPEPYHGGKLLHATKLISAPLVHYSAKPVIVAKPDLSHSHEYGNYVSYSGPSHNYKY*

>Woma_00009493

MKVIFILITYIWAYLKLGLALHVPLRSEQQRSHDHQHNRHTNHEYLHQLQPYYSDVNLQRATSHNHGLGVYDHYNNVDHQPFYHHATLHLNNDDESTKLAESAKNPNNLLNGIYHDGVVTYDLGLPKYEILNPYDPPEIITAGHPQYEQHLNHIKLTKNFTIENFGVISMLDGSDTIYKITDPITTTTTATTINEPTEPTTTKTLRRTTSISTSPPQPSMEKYKNMNSDIERHLEKTKEKLQKLNDELEFNNKNRKINATAKKLNKHFKSTSYTSSSASSQPATLIESNNGYSQSKSHINIPIEAEMFDKDHRFVKTVHGHSVLLPAIAMSPPIDSQLHGNPLSPTRYTDRAHFSYSTAHQLSSHLKLPLEEDDNNFDRLYRSATENSLEESSPSTDGVE*

>Woma_00009459

MKMAKFKFRAFLLIFVNILQICYSAPSNNAKKSLAVDYAALTEDVKWLFRGVSRIADTVEKMLPNVTVTDVKSRSFSENTKLTFLIGLHHLLVGIREISMTFEQNFNTDHIIQLQQLKNATNFVNEILIALKMWFSQIRIVVLHLINLYNRYIPEVLFGKCVGNYLITAYPDRSYYEYPFILVAEMYSSIASRIPFSTKVEKSTESVLIDHDTNEEQNEILHSDSDDYSTSSYGHQLDEGNTSNAENFLDFSLVLNENKDQIIKSAANQQSWQICLKLYTAESISRSLKSYFLGL*

>Woma_00009432

MQIKIIYNLLQLSVIIAISCHYAKGQQNEVSPKLDFDDTEDKNNTVPVVPGVKQGRNDERDTEKDKESETEKGSKSGPKIHGVRVTVDTGDTHKSKESKESVEITDLGKNKKRVGIHTDITFEITAEGGDDHTNKTSREQDVDKEDASVPIYKGRDGSTHKNRKPYDPKSHWNPNFSSERRYDHSGRANYPGYYPEYYPNNVPVYIGSAGARGAGSPIYRQNDGWTSYIPRTSYWTTERSVHVDAYNKPNDVSAYRTTGWKPCYCLTNSNEYRKRRDNNPHYHNQQESGINAVKPTSSLIEIIDGKLEIPFSRK*

>Woma_00009433

MKYFIVLALCLSAVVAEELHESGVGFHTLHYSQHQQTLEEAKKKIADVLDGQHAEFEEVGSYGGVGVGTVGLSSSGLSSSGLSSSGLSSSGLSSGMSSGLSSGSEFNSGSSFGSTAGSSFGASHSANFAASSQFGSSANFASGVSHGAAHGADAHGGSSVVIPVAGGSTGSTSYVESSAAENVQQVVPEVLVHGGIGGGQESYVRHDERRTENQVATPIIYSSPSGGSEKYFHHEERRVSNAATPVVYTVPSAGGSENYVHHEERVQTQTSAPIIHFAPSTGGSENYVHHEERRVQSQPVPIIQYTAPSGGSEKYVRHEERRVENQAVPIIQYTAPSGGSEKYIHHEERRVQSQATPIIQYTAPSGGQESYMKYRKVTTTATQPQVIVHTAPATSSSYVHQSSAGSTTGSQLGSLVTGALTAAPYTAPTYTANSGNSLFKTGFGASHQASTNFGAAHQAASNFGASSTFGAGHHAGFGTGVNSAYTDGSAFGSKFGAQSKFGSSFGAESSFGSKFVAGSSSGLHGYMQEAERLAKLQAQGMSVGGARAGGSQFGSANLESGVGGYAGLSGLGNAGYKTKSWEKASKWSSQSEFGPDGQVKNYKELSTGESENYDINGRRAGYRAATTTLDDNGKLSSYSLHT*

>Woma_00009506

MINKKFLIKKIIVFGIILNFTTCLKAETTKPEALTSPMTETTAASSAQATNAITPTGIFNVNPQSFDTGFYAYDDIDDYVPFNSDAHDHFSWELLRSILDEEQTNVVISPFSVKLLLALLAEATGNNTQTQRELIKTLEVIKSPDNLRGFYKKILTSLRKENPYNTLNLETKMFTDEFIEPKQRYAAMLATYYGTSIERLNFKDTQASADHINEWCKNVTNGRLQNLVTNENIQNSVLLLTNAIYFNGLWRRQFNETYEGVFFKTPVNQTRVQYMEQTEYFYYYDHSSMDAKILRLPYKGKKFAMFILLPKTKKGIEELTHVLQNDQVKRMQFMMEETKLKVTLPKFKFEFNKNLKSTLSSLGIHDIFTDDASLPGLDRGAEVAGKLKVSNVIQKAGIEVNENGTEAFASTVFEIGNKFGGDPVIEEFTVNRPFIFFIEEEATGSIIFAGKVLEPVL*

>Woma_00009620

MQTPKLTLENKFEQESTTAQTLTNPNTNCTAITTTTALIKPASPEVATTPDTVYGTYDAKTNSITIVMDDVAVPVSEAVEEIYCDGANNVAMDEVVCNNTSSSGMASPSQVFLNVTACSDDEADNDDDSVNFDPIERFLRPKRPLVSPLAKSPAPSLHSATSDHGYESIMGSPSPPSHHFEPLAEDISNDDFSTWPPGFNELFPSLI*

>Woma_00009611

MLSFNFLLASLAVVCIWSTLPTATQCDTSYYEKYYSARRFLPLKKPSQVQVNFTLNLEGEKLEEFEKVALVGDANALGNWKANGAVFMNKTEQYGKFQASIWLNVNTTLNYRYFIAAQDKITKFIQVRRWESSIQSRFLNLSTTSVKRSDTFGLIGSASTKEIQRGWLNSGHIVQFKLFRNPLHINDTAALKNQRLRLKLTPVDPQWRTAIVPSARAFAEYVRFEYGNSFLRKQPEYGVLYQDKDIIMFHVSVSDLKNVAYLWQVYVEDNETNVARLIGSQYIYPDDIKGTDGDVLVNLLSSTWLNTVGSLNLKYVVINPLTTSNVDFRTSFTDYWRKEWSGLEAGHRGLGKSLKETTNAAPILENTVATMLAAAELGADLVEFDVQLTKDLVPIIYHDFSIWVCMESKTPTSKDDLTEVLIKDVTYEQLKDLTTYQVVGNKIVEYPSHNTMTNETHRLFPTFEDFLTKVNKTVGFDIEIKWPQLQSNGVWESKQTIDKNVYVDRILDVMLKHGCGRLSFFTSFDADICSLLRFKQNMYPVMFLSSSKEAAFVDPRSDTIYDTVNTAQAFDYAGIVPNAVFIKKKPEWIDVALKQNKKIFLWGGELKDRESIDWFIAQKPTGVIYDRMDLYITPNRTSAFDKFPDLPSFFELQCEPPSQASTENDTITNILSNVNML*

>Woma_00009593

MKTTAAAIILLINAFNLVLLVTAQSKTSDEFPEKSEKCFQRESVPQSFKARFQSFQYPNEEIVHKYIHCVSTELDIWDDASGFNVEKIAQQYRGRANDEVVIPVISKCNQDNQNRNKALWCYRAYLCILNTQVGDWFKDDVRRKQQQNSLPNGHH*

>Woma_00009613

MIWGCMSAKGLGNLCFLESTLNASRYIGLLKENLIPSLESITSFGEYIFQQDGAPCHTAKITKKWLENNNINVPDWPFSSPDLNPIESVWAIMKKRLRSDPQTTVAGLKKNIQEIWESITPEECQTLINSMNKRVTAVVKVRGDVTKY*

>Woma_00009654

MNYSIFLIFLLILIKLKNSLILAQKDLVRQVLKMNDYDCETHKVQTKDGYILTVHRIPPSPTLTKPSNITLLLQHERPIILMHGLIGSAADFVLPGKYKALSSLLHQQNYDVWIPNARGTTYSKKHITYDSSQPEFWNFSWHEIGVGDLPAIVDYVLNYTQQSSLHYVAHSQGSTTFFVMLSEMPEYNDKIISGHLLAPVAFLKDLKSPPFRVMATKSDQIEALLNHLGLHELFPSTALNQLGGHLLCGNGVPTQNLCLLLTFLTVGFSDYQMDRNLLPRIFETTPAGISRKQFQHFGQLITSGKFQQFDHKSKSENYRHYQSKTPPEYNLRNVRVPLQLFYGSRDLLLAKQDVIRLTNKLKNTYFTLNEIRGFNHIDLLYSTAAPQLIYKNILKELQKFYQ*

>Woma_00009812

MVCRLLGFKSMEYERSTGANIELKVLQILEEFSCGPSGKLNIGTDRGTYMISAFKKQNRINCIAHLLHNSVKKAMSSIDEIEGLCDACAKLVK*

>Woma_00009811

MAHFCGFIKLIFLLLMLARIPSNASRPLKGNAEANSDLIDGNEFLKLFMEREQLDKDFYSSEYVGGSGGGGDESNRRKGLTSATKKNETYDKKSAETGRRSSSGRSLRDKTPLLNQAEGQLEDSKQVVKLVTSGSKIVFLEDYDENDAILDAEVRDIDLKKEKINKNKTHSNSNLDDYKVVAVSISSSSQRGTTMARNHSKSSSTTTTKKPSTTTTTTPKKPLRAASTKTDNIVGDNFINRSHKSEFSIEEVEPLTTAQHKTRKIHTRQRTSIPVTSVADTLEALDSENSASINIQNILAAPLQQAASARSASTITSTYHSNQRNMTINHEQHRHKIDNDNSDDSEAQHMSSGIEDQLLPFQTVIYHDTKAGHGPQSRSISYSSISQNVEDLKKWQQSGILAEARRNVSDSLKPMPRTNISLSLQQPESAKFYSQPSKIYSEPSKFYSEPAKVYSEPSKVYGEPEKFYSEPAKVYSQPSKFYSEPSKVYSEPSKVYSEPAKTYWPLTATTTPTTNSVNTLIPPTNAPIVTTIMPPMQQQQHQLLEQQKTKRPQQTLTYNQQQQQTQRHNINPCLESAVSSLFSSSSLSAHPAIVKQRQQPDSQQHSNQNAKGLPNGDLVRCVAEISHTPSAGAAADDGTFTTERSRTEELFTSTPEQHYEIEESVSVMTNGRAHGRNGFTQPTISALHQQQHHGFKPNRQRTRIHFGTNVDNQQQAEVQQQHHRQLAQLPSYNTNANGNVETGNRHNNNARDYNDNGGDEGEGDDGSYKNNDYEDDDSGTGRDSPANGNNGDDEDSKVAYVVEGRNYRKYRVEEKTDDGFIVGEYGVVDHNDGNLRGVRYTADSTINPSLIQKALLTFLKLK*

>Woma_00009820

MFRFLALTSLIALGASQGYHQDPKTAAIISEQRYLSGDGKFGAAYTQEDGINFKEETDSDGTRHGSYSYVDPTGQRRTISYTAGKNGFQASGDHLPVPPPAPPQPIPQSGYVPQPQYQPAPAPGPSYHNNDYGDDGSYDPRYNDPNFGQNSQSYNQPQYRPQPPPQPAYNPPPPAYNPPAPQYNPAPAPHYNPAPAPQYNPPPQQYTTPNPHRFSPPGKLSLNRTPDGFTYSFNKVR*

>Woma_00009830

MKQFNAGLCAIVAALAVFGLAVVDSSPLNQNRQLGAEKCTWGPSYWCSNITNAKGCHAVRHCIQTVWETHQVPVDNDSICQICKDMVSQARDQLRSNETMEELKEVFEGSCNLIPIKIVKKECCKLADDFIPELIETLSSQMNPDQVCSVAGLCNNAEIDKLLMKYYTSALDGSLKEEDDASAELIVPTETKELSAAAAAAAAPKQLLLSCGNCYNVAKLMTDKFQSSNRDDVLENILHICGEMSSFSDACANIALTYFNQIYDHIKENLQPAGVCHISGVCAAKYHQHSEDPKEEEAIDLMAPGNDDIPCQLCEQLVRHLRDVLIANTTESEFKQVMEALCKQTKGFRTECLSIVDQYYDVIYQTLVNNLNANGACFLIGVCPKGLDALFQGEIRPLLPVLPVLPPAEIKVTIRKKLGAHEPKFSQDELKAMTLPIDHLMGAANPGLLVKGATPATEDEIKHLVNGICDKFPNSIQGQCHNFIDMYGDAVIALLVQGLDPRQVCPKMQMCPPNHENHNDMEVFQPVPVDEQDKPTCPLCLFAVEQAQEKIKNDKSKKNIENVLNHLCSHLPSKLQNECVDFVQTYSSELIDMLISDFKPQEICVTLKLCPKSKNYLDDLGISLENENSSEDEEDFALNEINDSEELPIEIVFQQHNEGSSPNCLLCIEMVKLAEKRLDKHMTKDDIKKALEHSCLKLRKNLQPKCHQYVDKYGEIIAELLLKEMDPKLICTEIGLCLFSEQEDLEIDEALKYDVIAMPNENAVWINKNDRFVGMDEAMTMQEPPTCVLCEFIMTKLEMELKNKTEQDEIKQAIKNICNIMPKTVSKSCDKFVEQYANAIIALVGTIPPKEICQKMQLCFAGLSEVITDEVIECGVCHGASMALLPYLRKHTEHNNIGSVEMINVACENLPAKYYQICSEMVRIYGKSIMNLSERIALDETHICAEIGKCFDNEKSNLAFARISV*

>Woma_00009800

MHGFIIFLCFAFSQAEKLGYNYGQSGSHNAFESRFKNHGNTVSRFSSSSTTSSNNVHHRIGSGLDNGSRGYAADSIRSEIVASPVETNYNKEFFSFSAPEEDSNDVAAAQRIAAEIRKNLRVIFIKAPENNGLTNAAIQLAKQSAESKTAIYVLTKQTDVADLAKHLQNIQQNTQSKPEVHFVKYRTPEDAVRAQQLIQHEYDTLGGTSRVSKEDVAPVIDFASKTEEETGTRNLSNNVDSTDSFATAINAYLPSKIRN*

>Woma_00009803

MILTACVYPITALLFICTFSIAGVCSEDIKTGRSVEGDINHARPVAGGNDVFTSSFLVRFRRSVDNDFAHKVANKYGFENLGAPKIMLLCLTF*

>Woma_00009821

MNFLNILFLFGSALTIIKAQSASYGRSYYSSVLTPTSHYSYHHGGPGSGSGGFVPLAFKSDLISEESKHRSPSKYSKKPTYKGYGSDEDDISSESYTSDEYSNEPKHHPFVSKENDSREYTIGTHIRVQHPITSPKKTTIVYPTKSTKYKTLANVGSYPLDVSQEERGSDTYLPSKSSKTHKYSKQYEPDPFHVIPAPKSKSPSHYHHSSSSSIGSSYNTYEPEPETYENSDLAVKHLKANLRDSYRDVASKKQIEKYIEDQGKLLDEALKLQLLNSPKFQKILKTAEKEHQFNQPDFEDEYIANVPPPFRDNFHKSSPSKLSRSRRRPTKSKPLASSKPNRKYRSGFVIEV*

>Woma_00009862

MFLSLIGIFAIILNLVLHTNSDELIEVFKWKQMDYYNRGNNPISTSPSNQPSGIIYFPDQYHGTRSRRQLQNASYIPYNNVPMGVTHYKGRLFVTIPRRRVGIPSTLNYIDLAKDGRQESPKLIAYPDFETNQLTPNSGNLVSVYRTSVDNCGRLWFIDTGMLEYPNNFMQIQKPSIWIIDLQTDKVIRRFEIPPSIVAEGRGLASIAADTDKGCSKTFAYVPDLLNNRLYVYSFEQNRMWAFEHNYFHFDPIAGDLNIGGQSFRWNDGIFSVSLGPKEQNGTRNIFFHAMASNNEFVVNNQILQNESNSQRGYHGAVFYAEIQRSGVGCWNTRKPFTANNHGTVARNEQTMIYPSDLTIDEDGTMWIMTNSMPIFIYSTLNANNYNFRVWKQNTAAAVQNTVCA*

>Woma_00009795

MRRLIVMCLLAATYAADIGYEYQPVTQSNDDLLHYLSAESSNLLSQTAEIPLSHQAIESSSQQSASLNREFQKEFYTYTAPESSFDDKDATQNIANAVKRNLRVVFIKTPENKGFEEAALSLAKQASQGQTAIYVLSKQADIGALAKRLQNLKSDNKPDVYFIKYRTAIDAINAQNAIEKQYKDLPGSNQNINGGNAPVLNFSSRDSVSTGHIPAGPSHQYLPAHPISTINDYLPPSMRRFRYRV*

>Woma_00009796

MSNESSSIKEQIKDILLKYRTAPPKNAETSTNAHSSPEQLIFQHASIYNANLNNNSKDGPVASQHQHSPGACDDTIHIPHEDENRDLHQIPRSTRGSSLQRKTPGYLEDYSWTRLFYGIILRVVELLYHYVHL*

>Woma_00009791

MRAFIVTCLLAVACADKLGYNYQPVAHSDSGLSFTPGASSGSLSGFGGNLGSGVTSTSSLGNGAGLGGSYGGISGGVSSGFTGGLSGGVSNGFTGGLSGGVSNGFTGGLSGGVSNGFTGGLSGGASNGFTGGLSGGASSGNSGVSGSYGGSSVNYAPQAELEKEFYTFTANDDDFSEPAVQNQVANSAKKNLRVIFIKGPENQALENAAVALAKHTAEQKTAIYVLQKQADVSSLSQKLNTINNNINNKPEVHFVKYRTQEDAVNAQNAIKSQYDSLGGSSQSYNGGVAPVLNFASKAPVAAHVSHGSLGGPVGSIGGGSLGSYGSVGSVGSASSAGSVGSVNSVGSVGSLRSVGSVDFSAPAASYIPPAIGASSSAPSSTYLPSIVARNRK*

>Woma_00009793

MRSFIVLCFVAVACADKLGYNYQPVGHSDSGLSFAPGSGTGGGLSSGSFGGLSGGSLGSGLGGFDGSSFGGIGAGNLGGIGSGNSGAGLGGNDGGISYNAPSYTAPAELEKEFYTFSAPEGEFDDANAAQKIAGSVKQGLRVIFIKGPENKGLENAAVALANHAADQKTDIYVLSKQADIGSLAQKLNAINKNNNNKPEVHFVKYRTPEDAANAQKAIQSQYDSLGGPSQSHNGGVAPVLNFASQAPVHTPTAHTPDNSYLPSSIFRRV*

>Woma_00009792

MRTFVVLCLVAIAYADQLGYNYQPVRHATENLPTYAGSQDGVNYAAGSNNAPSYTAPQGDLGQASQAPQAELDKEFYTYAADENDFNDPAASNQIANSIKQALRVIFIKGPENNGLEDAAAALAKQAAEQKTAIYVLNKQADLASLANKLNNINKNNNNKPEVHFVKYRTPEDAANAQKAIQDQYDSLGGRSQSHDGGVAPVLNFASKTPAVSHVAASSPSASYIPPSSGALASAPSATYLPASIIRRFRL*

>Woma_00009766

MQGTTTGKDLHDELKSVLDNFSMLLENIMGVSTDGARAMSSMESRALSHRQLKEFLKDLETEYVDVVFNTEVRWLSRGAMLKKSCVRVAISSIPTDIDQLVSRKQCQIAH*

>Woma_00009733

MFKVIVIAVLALAISVTADPIHQSRRILARQQQQQLAPAPTGYPPAGVIPEIPLGLPTETEKPAVAQEPDEVYGPPEVETQQPDEAYSPPETVAQKPDEVYGPPELQTVQPEIETVPEVDEKTVDEAADEEVAEDLDEADEEVISDENDSVIAIATTFKKPARLVYQRFPQRRQPPTAVPARFVKAEAVKPVNFIYTTSFQTFPQK*

>Woma_00009780

MTTATTASNDAPTHTASAPHPTTPPKVKSIGVSTVKRTYCHKSVNSSPIHCISTLTSPIDTQNSLVAVTREHVLKQEIEQLQERLKDTEERLELFRLQHDTVSQLHRKLRESNTQLQEESEMLKLDVQHLNECANVLRAELHADRHDRDEATNLQKVLQNELEETRAEKKRALEQKDRDAKTIQDLQRQCREMEGILMRKHPDSVSMKLKQAYLNLTVSINHLNYRIR*

>Woma_00009749

MEPWKLAIITIVALYYCFQVSFVAGLECYVCSNQSGNVEKCLKTIKTCEPGEDVCGTEIRWGSQPYFSLGAIKQYYVSKRCMTKQMCQAKRKRYMPYCTHIWYEDWSCHECCQGDRCNYYVIVSM*

>Woma_00009668

MLLKREGLWTAITENRSAENFANWDKIDQEAHASIALCIGDDQIHHIRNKTNARDAWNALKEYYERDSPGVKVRLLCEVMAKRANDDTDMEAHINQINELFQNLVAFGESITWEFLMTAAMLGSLPSSYDGLITAMEAYSKKNLISSLASPKIIEEYGRRQERDKFVDATGGVDALKVSNFKRKNGDSITCFFCRKKGLIRSKCKEYSTWKANKVREERKPNANIVDQDDTLENLFFVGSRNSDEWIVDSGATCHIASNRDLLDSFDASHTEDVSEPNGHFGCIWQGKHQYTTS*

>Woma_00009732

MAKRLTGLLVLLSIIFTVTLAEPGVHSIRIRFPGPSSDKIVLYKPAKQQEAPYPPAGLKPEPAFELPEPTYLPPAEPELTYGPPEIVYGPPAETYGPPAAEPELTYGPPADTYGPPDLTYGPPELTDNQSAAIVNAPLQFTLQFTPPRPNKAANFRPVSFQPQSAAIVNAPLRPVNYFTLPRPERIVAFRPRRPQKQGQNYFPATYLTLPRVERLIEFAPRRPSTSNRRPQQQQQSNGFSRPLPSSIFGKPNNKHSGRQLPTNIFSN*

>Woma_00009751

MKHFIVCVFVFAFAIVARAEEPDLVASASENSERAEELKGSATSLNPWQEKHQEKRYVGGYAGVGAYGTGLDTLGGYSNYGSYSGYYNPYQSKLGLDGYAGYPGYNGYTGGYGGGYGGYGGYGGYGGYGAYAGGYNAYPYNYYNRLGATYPYTRPSYAGYGSYPSSYYTSNYANSIVGGGLGALGTPGFVPPVAGAAGYQYGPGISGTIY*

>Woma_00009875

MKFLVCLAVICGLFSTIQAYSFLIPANMGLPGVCMYNGIMLQRGDNNMLSSCQNMHCNEDGSILVQGCGHHDMRDCRILDPINLHKPYPDCCKMNFLCTMANGEVINREISPLMHRGIYA*

>Woma_00009876

MVVKLLLLQLLALVCLLIMMPLVGVSSFKRNFEPKYTKAAIPLWKQRACEKVQKYKANSHYVCDDKGDVKCLPGWQGDLCQVPMCRKGCDPMNGYCQRPGECRCRIGYTGELCEKCIPLPGCQHGDCTKPFECICRPGWDGLFCTEPTCRQGCHNTRGYCEVPGECRCRIGWAGRNCSDCSVLPGCQHGTCTKPLECNCLPGYTGLLCQTPICNADCHKQHGYCKRPGECRCKVGWTGPNCDKCFPYPGCVNGDCEKPWECNCKPGWGGILCDEKLTYCKENPNICENNGKCISLTKEDGSYRCHCKRGYLGKNCEIIDEFLLTSTAAPRITPPPLVGWDDDDDDEDVILNDDNEIQSSNKKEIQIKLSKNETIELKKENRTETNVKLGITAKINTTQVQENVQGNLEEKPKPINTSAIEKADNKTLEFITLPTSSDIIGTATAVMNTTKTMGNKKTSKDPNKLAKVKTSTTTMEPNISSTTPAANKASTTTISPLSKLDEDKNLHSMPLKTTDTKKPETKDEDDDDEECTDDDNDDECEYVDDDDEDEEDAK*

>Woma_00009870

MTTKISFYLLLLLLHFNHCLRHVFAMSSESARYKEMNGITQLMETPLDNESCLEFDLYKNLFEEMNSQNHPKIQLYYNRVIQKFYDSMPATVLFLQIWKIYKRCDPSTFVAMVRAQLSLFWLHNMNEYPDYDYLLMVSNGLYKIKNFFLYPSLNARTRLLFERALKSLPSSLHHLFGHREFCLMNRKHSEMMYQTESDEFVLNHEIRFGFTWHMVKRYNDGKGKIIPLFENFNISFLYEGEEDGEKGASGRLIKHVMPAATSELTDIPSTLNVVLYNKHYKNFYSRWPVYNQKIGGNISEIPEYIWQQDYHVWSVQLVDDDHLVFFQNQYLMCATTFYDNKRRFIFGLKNRTVYDTDCQWSAGKCDRR*

>Woma_00009878

MLKFLIILTLCLHSCCGFGQFHMKKYTPRIDSSEEDNSVLRTYRRCLWEQSKSLPRRLVLLSLCSNLFCENNQIIPRSWSIFVVEKMTRHNDCLDILPDQCKQGDEEELMYKPFPDCCPVYCNLKRRMNRLKTMHYRHRMLLNMQRQQAAGATNLLLNEYGMDSL*

>Woma_00009946

MHNQNSKSFLIRDLLGDLINRQQQHQQQQHHQNDEDSDVSDNNSDIDIEDRSSPDSEALNCHYSNSQLNGSHLLFSNNESSLDSSHYLNDNEHSTPTQMNQNHSTAFISEHKLGLPKSGRKPRRRRTAFTHAQLAYLERKFRCQKYLSVADRSDVAETLNLSETQVKTWYQNRRTKWKRQNQLRLEQLRHQASMEKEYVAENGGANPLGCCPPGLSTFNATNPCNFLTSAAAAAIFRNVGYVHGCQL*

>Woma_00009893

MYQKQQVILAALVAVLVINSVAAYKLGYRGNMKHPDLDDHCYFTEHKLSIKVNETVSPTNLDKACISVFCRDDYVLEIKHCDKIAPNQACQYSAYDYTKPFPDCCPLVTCGDEVYRSF*

>Woma_00009890

MKFTLVLSTLLAAVFVNQISVSLAVTEPVCAYKNSQDETIFLKYFPYSKTGQEYVDFGTDGKCLKRATCSETFTTEIDECKQYPVTCKNKKDFNGVFPACCVKC*

>Woma_00009896

MKRFLVFFCLLLTIFPYAQAKICDLLLKMIKVGDKYSPPGKCIEYQRVDTHGAFTAKVCPDIQSLRPCFYIPEDTSKPFPECCPFIICD*

>Woma_00009894

MKRFLVFFCVILAVFAYTQADECKIDGHVVKVGEKYSQPGKCVEFECTGPNAFIAKTCPDVQSLKPCKYIPQDNSKPYPQCCPRQEC*

>Woma_00009895

MKSFLIYFCLIFTLFAFEADYDCKIDGVVIQAGEKYSPLGRCIQYQCVGPNALVAKTCATYYSMESCIYIPQDNSKRFPECCPDFIC*

>Woma_00009931

MTKRLIVLTLLLIACVQSSLSWNQRSPRDLGTRMKTFKEQPIEENKENFSGITKLAEMVDKDSPTDSSTEMDMLSEMLSSKLSEQTIELAVDKTKRQTVIKGNRNRLNNLINLPVRLKVLANVLSEINQGVVANTEMPLSQNTIIGNDNTVSNLVNLPVDVNAVVNVLSSVNISKATAENGKPSAGGVVGGLLGDSGLVGGLLGNTLGAGGESGSGLVGGLLGSTLGGVVGNDGLVGGLVGNTLDSVAGSDGLLGDLLANALGDTLGDTLGELLGALLGNNAVVDNVVGTVGGQGLVNGLLEGVLGAVINLVDNLLHLLEGLLDGLSNGLSHANVTTTTHSVTGTGGATSTGDGVHIEGNNNVVSSLINAPIRINALINVLSTLRQELSGNAIPGGSLSRSGTTIKGDNNKISSLVNLPLNADLNLNVLSDVTQTLLGKHPNKPETIQPANEKPGHPGSTHAGNEHPVIGHTGSAQPSSKSTNIVGIGNTISNLLNVVPDINLNLNLLSQITELLGGRVSESDPTEICFDIPISQADTSIIGNNNKISNGVNLTPKINLDLNLLSNIRRTGGTLPAHVPTQRVCIKKPPTEPVTTTKPSTAAPVTIKPPIVTTEKPSPITPVTVEPPTEICFDVVEPIPDSGTVIEGNNNKISQLVNVAPKLNLGLNLLSNVQETAPVSKLSIKRICVPIETTPKPPVTVTPSTPKPVIPHSTPYPDKRCFRRTCTNWKQFVDYNCMSQPAQYPVYTLTQNL*

>Woma_00009932

MKTTCIFLILFGVLLKFHLITADVVRFTRVECESLDKEFAAIQECQLKAMKRNKIAMNIHVKLLQVPVDNITIHAQLLRKAREYRPFLYNATENFCKFMLNTENLMFWKMLLDLIRPYTNVNHTCPYNHDMILKNFILNENRIKSIIFPAGYFMLRFNMAAYHRARVRVQIYFTLRDAMFHN*

>Woma_00009936

MLERLDQQRERISNVIRRKSLGNALDYYIEKQTTYEVQQQVQQPSLQRHHYMPNSYQSPNVNTVTSSTRFALAATTTSQTAKIQNKNLQQLTTATTNKKQVMKNTRSTVLETPATPQPYDDYVPRNIVYNNIHAVSSMLRRQDDESLATTATKQSKPALLNLSLLDESNVEDSFEQLIIETLWNGYDCAPPNYIFV*

>Woma_00010053

MFKLVVLSALFAVVLARPGLYAASTVYSAPAYASVAVHEPALAHVGSVVKSVPSAVSHQSQSVVHSSAHVVEDVLAPAVKTTPLVYKTYAAAAPVLYKSW*

>Woma_00009955

MANLIKIIALIALVLPACLCVTIKPRSSWGAASARSPSRIQGAVDYVIIHHSDNPNGCSTESACQRLIKNIQTDHKGRRSFSDIGYNFIIAGDGNVYEGRGWGLQGSHAPKYNRNSIGIVFLGNFENTKPSNQMLQNAKDLIADAVSGGYLKDNYTLLGHRQTKATSCPGTQLYNEIKTWPHWRNI*

>Woma_00010029

MRFIFLCAFATLAAASRNHPQLIVLKSETPTTEYREEDSKGFYSYGYSSDDSAKAEYKTVDGSAKGFYSYVDSDGKLQTVKYEAGRRQGFTASATNLPTPPMDNNRPPEPVRDTPEVEMAKQAHFEAYREAAIKAAMQPDTGDDGGEVDDTNAATLAASRSDGSLDGNLSAEQAAAQQLETELQQNTRELLLNRQSSREQLLQLLRKQQQLASQEGHSSGVLLAQQQQAIQYPQLMTTYVLNAEARVQPATLSELKTVYRLDNTDSKVELKLGGLDLANNQQRSLDLLQQNGHSNQLSAENTLGTTTFESIRVPSNSYYTVDNPDTHYTVETPTVLTTLPRLASVNLGNHRLPISLSTAFSSQRLSGGKSTSH*

>Woma_00010007

MILINLINILILSLLAATFYQNITATPIQCGFEANCTVTSNSEVCILDETDGACIRKYPSKCHMDIAACKQGKNITDYSPLYCAMDGYLCEEGYERWTIYFGHEKNIF*

>Woma_00010006

MICIRFKVITCFIILNLLTTTYARSHFDLREKLARLLLDPEYYDDDRNEIQKPRQTQGMLLKDLPSFLSSSEEKLYDDISSDNEAITSKVSNIMKTTEATTTRITSETMKLETTTMIPPKLRPIISSIERLSIMIANLIDLWGNHNHL*

>Woma_00010005

MKFFAIIFLALFFICAAQADDCPVICPALYQPTCAHNGRCFKEFSNICSMNVANCNGKDNFTQVELHKCSDNKLTKC*

>Woma_00010069

MYKLVVLFALIAVAAARPGYVHSSPVVYSAPTYVHEPVGSIVSGIPTAVSHQSLSQGHSSAHYVQPIVAPVLKTTYAAPVVKTYATPIVHTSYAAVKPLYHSYSAAPVS*

>Woma_00010049

MFKLTAIFATLCLVSTVSAGLVTTHHVVHEPALAKVGHVVHSAPSSVSHQSITQVHSKASVVEPILAPVVKTTIHTAPVVHTYHAVPVVHSAPLVTVHHH*

>Woma_00010048

MFKFAAVFATLLLASAVSAGLVATHHVVHEPALAKVGHIVHSAPSAVSHQSITQVHGKTSVEQSVLAPVVKTTVHAAPVVVKTVHTPVVHTYHADPVVHAVPVVHHSAPVVSVHHH*

>Woma_00010047

MFKFIAVFALLAVAAAAPGFVTETHHHAVVQPAVLTKTAIVDNSASSAITHQSFTNLEKKVPVVATYTAAAPVVKTVVTAPAVHTYAAAPVVKTVATPVEHTYVAAPVVKTVATPVEHTYAAAPVVKTVATPVEHTYAAAPVVKTVATPVVHTYAAAPVVHHSVPEVHSAPVVKTIVSAPVAYHAIH*

>Woma_00010046

MFKFIAFFAFVSVALAAPGLLTEQHTVVQEPVLAKVGSVVHSAPSAVSHQSFTRVHNKAVVTPVVQPVVKTTVHAVEPVVPVVKTVEPVVPVVKTVETPVVHTYHAAPVVKAVHTPVLSHAVSHQSHVQLHSNAALVTPVVKTVVAPVVPVVHAAPVAHVVHH*

>Woma_00010040

MFRLVILVLAFIWLNEAAISSLQLVVPDDPYNVLSAGKSSTTILGHPISTLSNAISDEDKSSNIVSEESNESANTSAATATAFSSPSPSSASATAPAAITRVKPSSSSSPLGPITPSSSSGLSSRGTLLISSSIQASASTGSAGSSLSSSSPVASARSSATIQHRILPAESIPLPITISTRPGLRTVIAPETLTIQQPGIAKVGDIVQQIPTAVSHQRQTVVHNHARVVTPIVAPAVRTLNSQIVRAYHTPLVYAPHVVEN*

>Woma_00009996

MKLLNKLKDVFLEALLMISLINLTTCHIQEASTTPPATQTHWQRPAQMTRNTVTRWKANQTPVAATSHNCDYKQLITSLHDRVSLLVTVDEDLRKRLDNIEKK*

>Woma_00009994

MVTSSLLTRYHIGGTNFSTTNDGVILASTNVKINNNNLGVTMREKKGGALQKLKKRLSHSFGRLSKCEKFKIKINLFRYLKNLFSMQSKIT*

>Woma_00009992

MHYFNALVGFLLVATVCQAAVVQKRTYRNNAEILVPVAIIQDGEEFKGQDKLQQQEAQPEAITYLETDTSDAAIPEEKKLETVQTIVEAQRRLDEEIVEEEIKEAIEEEQNKELINALEAEKQPEAVEDSLAQESDIQAEYKKVPEVEKQTSTKVFVIVQANNEAEKPAQSAQPEQTAQPAQPAQPAQPSQPSSPAQPPQPEQSAQPAQPAQPAQSAQPALPAQPAQPEQPAQSELPAQSETAAQPEQQPEEQPADPEGTLRQATQATPSQTTTQQNFVQQLIQNSPLGQFFNQITGQNTQNQVANDDASPGSPAPTLPGLFNPVTAVQTAAQTVVNSTSQAFAGLQQFASSLGNQFQNTLSGLTGQQITQNDETTTRPPGPIQQLVNTFIGGNTQQPAPTQQGPLQGLLNIFQGNRPQASTAAANDDIQPAKEPTAAEQTDENDAQAAVQADVLSNEIRDSAEANDSFEQSVQPDEVIVVNDDASQTSEQNEQDNENVTQ*

>Woma_00009999

MPYSPYPAAAANPMLATAMPAMSLYGHYPYGQYRYNPYHIPQKTQPVAHHVAHGMPPHMAANSMTTAAAIHLL*

>Woma_00010083

MFKFFIVCLFAVIASVMANPTYGYYSYSANTPYAATYSAYPYADAYYAYPYAASYSSHPYTTSFVR*

>Woma_00010084

MFKLIATTVLIAMAIIVPPATEARPGDFTDAAENVDVPPEHRAAPPSGPPQPHPAQPPYQPRAIYPVAAPPGGPQYPLFFTTPNGFVPFGSIYNAYQQPQIRAAYTILPQDQPIVSPIVVAAAL*

>Woma_00010089

MFKIFVFLSLFALAFAKPGYIHSAPVLTSYQHTATVPIAHVVRPLVSAHSVVASSPVIHAQPALLHGSYHGSYGHHLY*

>Woma_00010106

MKYFVLLTVIVGVVLALAIASPLPGGGGGGGGGNRGRGCNCEPAGGGGDGGDGGYGHGSGPGGLGGQGGQDCVRASWMPPCP*

>Woma_00010008

MFHLKIITIFSIIFFNVILTESVTSDDCDIPCDTDEERPVCVLDKDEICFTKFSTECIMERMACVFGKNYDIYSDAFCELENFLCSDIIQSPQATESELHSYNEDSKESIEVVLQNKSDTQDLTVNTIEKD*

>Woma_00010004

MGSRRSTPLLLATIAGVVVLLSQTNAELTPPYFNLATGRKIYATATCGSDTDGPELYCKLVGANTENDHIDYSVIQGQVCDYCDPSVPEKNHSPEHAIDGTEQWWQSPPLSRGMKFNEVNLTIDFGQEFHVAYLFIRMGNSPRPGLWTLEKSSDYGKTWSAWQHFSDTPADCETYFGKDTYKPITKDDDVICTTEYSKIVPLENGEIPVMLLNDRPSSTNYFNSSVLQEWTRATNVRIRLLRTKNLLGHLMSVARQDPTVTRRYFYSIKDISIGGRCMCNGHADTCDVKDPKSPVRILACRCQHRTCGIQCNECCPGFEQKKWRQNTNAHPFECEPCNCHGHTNECVYDEEIDRKGLSLDIHGRYEGGGRCLNCQHNTEGINCNKCKAKYYRPYEKHWNETDVCKPCQCEYFYSTGHCEEGSGRCECRKAFQPPNCDSCAYGYYGYPNCRECECNLNGTDGYHCEAVNGECPCKINFAGHYCKQCAEGYYAFPECKACECNKIGSISNDCDLQTGQCKCLSSFGGERCERCKHGYFNYPKCQYCDCDIQGSEEEICDKVSGQCICREGFGGPRCDQCLPGYYNYPDCKPCNCSATGSTATTCDNTGKCNCLSNFAGKQCTLCSAGYYSYPDCLPCNCDVHGSVGVSCSADGQCSCHPNFNGKLCDSCQEGFYNFPSCEDCNCDPAGVIDKFAGCGSVPVGELCQCKERVTGRICNECMPLFWNLNISNPDGCEECDCYSDGTMATLDTCHTKSGQCACKPYTQGRTCNDCKDGTYDLDGSSLFGCKDCNCDVGGAWKSECDKISGQCKCHPRVTGRDCTQPLTTHYFPTLHQFQYEYEDGMQPSGAQVRYQYDENVFPGYSSKGYAVFNDIQNEVRYELTVFKSSVYRIVIRYVNLNEFNVTASILIQSENPLELDQNVKVLLRPTTEPQFVTVSGDKGKKPSAVVLDPGRYTFTTKSTKNVMLDYFVLLPAAYYEASILTRKISNPCELGNMELCRHYKYASLDDFNPAVTPFIVNAKGKPTNPMEYYNDPEHLTIINHVGDIPLLTFNQPELNYIVDVPHSGKYIFVIDYVSERSYAEPGFIKLRVGNDEENYGSTPVYPCLYSMACRTPIIDDQIREKAFYINKEDDKPIVVFADFEEDQKVAIISVTAIPVEQWSIDFINPSPVCVIHNNQCSTPKFRSVPDSKKIEFESDHEDRIAIHKPPYAVLDEKVKLIHLDNKEESSIVIESKVSEPGRYVILVKYYQPHHPTYNVLYTLTAGKNQYDGKFEISHCPSSSGCRGVIRPTGEEWWFDIEDEFKFTITNNRPQGVWLDYLVIVPVDQYNDDLLEEETFDQTKEFIRECGQDHFYITHNATEFCKKAVFSLTADYNSGALPCNCDYSGSTSFECHPFGGQCQCKPNIIERQCGACKTGYYGFPDCKPCECPSTAMCEPHTGECVCPPNVMGELCDKCMPNTYGFHQIIGCEPCDCNYLGIANGNSQCDAFNGSCECRPNIEGRACDECSNGFFDFPRCDQCTCNIAGTELEICDKVDGNCFCKKNVVGRDCDQCIESTYNLQASNPEGCTKCFCFGKTSRCESAFLKVYNVSLLKKVSLNTAVFEPKSIKFNIWGLDQEELLINEDMLQADFSLSEINDDSIVYFGVLDYLGNQNSHISAYGGELAYTLFYTTGLSGKALFAPDVILYSDDHVLVHQSYEQPSSNQVFKNRVQMVESNFLTQDGKPVSRADFMMTLRNLNMIYVRANYFEQTLISQLSDVYLTLADEDDDGNMVDYEFLAVERCQCPPGYSGLSCEDCAPGYYRDPNGPYGGYCIPCECNGHAETCDCATGICKNCQHSTTGEHCEMCIEGYYGNATYGSPHDCMICACPLPTPSNNFATGCEISESGDKIHCECRPGYTGAKCEFCSNGYYGNPTIPGDYCKPCECSGNINPSEPGSCDTVTGECLRCLNNTSGVACNLCAPGFYGDAIKLKNCQSCDCEDLGTMKCDPFEGTCQCLENVIGERCDMCKPDHYGYDSGLGCRACDCGTASNSTQCDEHSGKCACKPGVTGRQCDRCATDHWNYTKDGCIPCNCNQGYSRGFGCNPYTGQCECLPGVIGDRCDNCPHRWVLKKDEGCFECDNCHHALLDVTDRLSYQIDPVVEEFQSVALAFFTSQKLNYYDKLADEIAPEVQSLDPKSVNLEPALLLVNSLETDAKAYTKQVNFTLDNAKDSRDMASKLLQNITGLNDKVDLTVSEAHEAIRAVDNLSRNLETAASTKIDSALSEAQSLLEEINATSIDLERNELVLKKATDLLDEVSKAIMPIKAQNKTVELLKNDIGEFSDKLEDLYVWSLQSSNRSTEVERLNALNKLAFENSQFDTVSDQQKATEKNILEAANLQTNGAITLVEIDDKVHNLNDILDTLRGLNQQIDAELPEMEDEQLEASNLTNQAEFKAMELLKRAQDLSEQYTDMTASAEPAIKAATAYSGIVEAVVSAQQLTKEAKYAAGNATEKTDGIENRAGVSDKESAELLQSARGSLHKVQSELEPRLNSSAAKVEAISKMNENTEKRLKEINIAMEALPVDSQRDMWQSSNENATEALEIMNDVLAILEPVVKQTPKELDKARNISKDIDSTSKDILHVQNQLNSVEESLPALLEAADDIELQQAKVEEMAQNLGDDIESLRRQIETARQIANDIKMGVKFTPSTILELKTPETLPLLATKTKVSTYFKTDNPNGFLLFLGNDNKTASVAPAANKNNDFMALEIVNGYPILTLDLGNGPERITSEKYVADGDWYQAVVDRTGSNVKLIIKEELENKTVVDHVKEEALVGANNVFNVDRNSRLFVGGYPPPADFTPPEDIHSSSFVGEIEDLKIGDEHVGLWNFVYGEDNNLAAYPRTKLIAEESPPTGYRFNGNGFVALKALPYNFKQRSSIQFSFKAAKDSKDGLMFFYGRNNQFMSIEMVNGAIFFRYKLGDHIVSTGSSDQYNDNQWHRVVAERDGRKGLLKVDDVIIVQEEAPPHVEDTMPALKRMFFGGYPNKRNSSLIVEENFDGCIDDVSISGNKVDLGQHINAVGVKPGCPKKFSTVLAYPHDGYGYLRTANISSDNNLNINLKFKTRQKDGVLFYGANYDQSSNIGLTLEDGYLVLQSMNSKLVSDMRKLNDGEEHVVTVLHDGNQLRLSIDDMEDKRSVTAPAALVITSGDIFFGGLPEGYTTPRNTLSNTAYFIGCISDVTVNGEIINFADSTEKKNGNINNCPSDILAYDTTLVPIHYAGGEDEYVPPPAPIVPAPKPPTTTRRTTTTTTTTTTTTTTTTTTTTTATPFIEPKRKWEVDESENVIDSEQHDIITTKPEVMTFKPEVMVTRKPVIIHELHKPEDPRCKLPTNPNYDVDFIEAGYRFFSMREQRLEIPSLPHAKIRKHYDLSLSFRTEYPTGLLFYAADKHHTDYVAVYLNDGILYHKVRLGELVVTLNSTGEMNDGNWQTIQFIRTQRKVSLLINQTQQESSYEFEERNTHLINIDFPLYVGGIPKHIEEMVRANVDLGEDAPFYNGCLRDFKVNGQTQEPELQPFLVVPCSDQIEPGLFFNKPTGFVKLFERFSVGTDFSLNFDFRPREPDGLLFSVHGKSSYLILELVDNQLWFTVKSDAKNIVTTNYKLPDNGSFCDGNWRNVQAVKSKFVITISVDWISAKPGVGQEGSTVTKTNRPLFMGGHQAFNKAPGLKTRKTFKGCIRNIQVNKKTVRVTPNLIYGEIWQGVCPLN*

>Woma_00009960

MFNLLKLLICCGLLIDAVYCYPDSRHNPNLEYELINPKDPQPNKPVLVPITTPRNIVINTPPPPPADSKTFAYDPVSKTWTKVKDSDPKPAEDTLLWNQSNDKWLTMALRY*

>Woma_00009961

MKSLIIFAILIAVTRARPQRFPTQQFPFQQFPIQQFPGQFPNNIGNVVTSTVAPPPTAGTVTTASPQYLACLQSCPSTMEYNPVCGTDDESYHNSGRLSCAQRCGKNVAAVRIGTCRPL*

>Woma_00010061

MYKLVVLFALIAVAAARPGYVHSSPVVYSAPTYVHEPVGSIVSGIPTAVSHQSLSQGHSSAHYVQPIVAPVLKTTYAAPVVKTYATPIVHTSYAAVKPLYHSYSAAPVS*

>Woma_00010060

MYKLVVLFALIAVAAARPGYLHSSPVVYSAPTYVHEPVGSIVSGIPTTVSHQSLSQGHSSAHYVQPIVAPVLKTTYAAPVVKTYATPIVHTSYAAVKPLYHSYSAAPVS*

>Woma_00010063

MYKLVVLFALIAVAAARPGYVHSSPVVYSAPTYVHEPVGSIVSGIPTAVSHQSLSQGHSSAHYVQPIVAPVLKTTYAAPVVKTYATPIVHTSYAAVKPLYHSYSAAPVS*

>Woma_00010062

MYKLVVLFALIAVAAARPGYVHSSPVVYSAPTYVHEPVGSIVSGIPTAVSHQSLSQGHSSAHYVQPIVAPVLKTTYAAPVVKTYATPIVHTSYAAVKPLYHSYSAAPVS*

>Woma_00010064

MYKLVVLFALIAVAAARPGYVHSSPVVYSAPTYVHEPVGSIVSGIPTAVSHQSLSQGHSSAHYVQPIVAPVLKTTYAAPVVKTYATPIVHTSYAAVKPLYHSYSAAPVS*

>Woma_00010066

MYKLVVLFALIAVAAARPGYVHSSPVVYSAPTYVHEPVGSIVSGIPTAVSHQSLSQGHSSAHYVQPIVAPVLKTTYAAPVVKTYATPIVHTSYAAVKPLYHSYSAAPVS*

>Woma_00010068

MYKLVVLFALIAVAAAHPGYVHSSPVVYSAPTYVHEPVGSIVSGIPTAVSHQSLSQGHSSAHYVQPIVAPVLKTTYAAPVVKTYATPIVHTSYAAVKPLYHSYSAAPVS*

>Woma_00010038

MTTHTLSWALLATIALCAVIHIQAEDQLSWSGSWSAPDSQDSMLSKRNADADAEPKQGEEDVQGRNYAVNEPIYSNDTDINEIIDQIINSRREGRILNEYDRVYEDGNVDQALMQGNDMQARNLVRDKLCALGLMSCEEAKRPYYSNIYAQGPPKYGGGPYGAPKPMPPPSYFNGRPHMGPPPSSINSFGPPRKVGYETSYKPGYRPSGPGPNYSGPYLESPPPSAIDGSATNSFNRPPPGAIIYGSKPPGPVYTGGNGPSSPPYAFENPEKIQVAGSQQTNFYSHQSSASAASSSTKVVVSGGLPTAAVSSNAAGLQQHVHHHYHHVEGADSAKVPAVAIPVGAGQTINTDFSALAQSSTAFTPQTQGSFQNPAFAAGFNGASQGGLLTQGASSLGASGLNGFKPNPDLYSKPSGGLSSFGGISSSTSGFGQSGFGQSGFGQSSSPSSSYHSQNPDYYKKELHNLGGIAASSNSNFNSLTSSYGNEQQFNGAYDTSRNQYVDCQCVPFNQCPAADRIGRKEDLLLPIDPRNLDKDIEALSDEAAANITVSATAEKKSDDNSKPEDKKDDDKQRSKRQADQNDENKPDDAASDLPLDSQGRLQFPALQAIELIRRKILPTYGVSFGLPYPNHPNGYPTNALGDHYPAQNPYFGAIGPNGLNLGLVNVNPLVSVQVQKTEFGDKVVKPLVNLHITPSANIFDAVGKLFKPKPVVVHNSHYHHHEHYTGFEHDHDHGEIISDPGPIHHFPSIGPSFTKYEIEMIPSKPIFEYHSGPHIYTEPSPIHHFEEDHHKEHYEHVSSHSSSSFGHTSFASQPSYPTTGSEHNSQFYPTTGSSHDNNPDYYAQPETNFGNSAFPQFPEAGNGLTPQSYPTGFGLSGFENIYDRSSQVNQSLTYSESKRYRKGKELSNPNVDNTHPIPTPAPGIAGGSSYITFPKDRRRRSTEEHEDAIEQRSGSVLAEALKLQERAYYGNRPVQKTCRANEVCCRRPLRPPQPPQHQFGRCGVRNAAGITGRIKNPVYVDGDSEFGEYPWHVAILKKDPKESIYACGGTLIDAQHVITAAHCIKSQNGFDLRARLGEWDVNHDVEFFPYIERDVVSVNIHPEYYAGTLDNDLAVLKLDHPVDFSKNPHISPACLPDKYSDFTGARCWTTGWGKDAFGDQGKYQNILKEVDVPILSHHQCESQLRQTRLGYGYKLNPGFLCAGGEEGKDACKGDGGGPLVCERGGVWNVVGVVSWGIGCGQVNVPGVYVKVAHYLDWIRQITQYYK*

>Woma_00010039

MLKLLTFVALVSLAASAAISTPLLAELEPQPALAPANTKHAGYVEGITPIFKTAPIVATAAIVPTALTTVHHLPYAYASNYLTYASPAELSHYRVAF*

>Woma_00010037

MYNRIKIHIIACVILALHCSQETVGRLTKRNGYSTYKTQIGHAHNSGGAFFEPQEPHNAPDHEGPSGSDGGSSSNKYDGSYYEYKITSPNGVSSHTAIAPPYHLASEPISHGPKSGYSIGSGLRSIAQGSANQAFTAVASQHAAGKQAAFLAKNSLAQAASQAAATAQAALEGKQVLLQELQQQAASAQKALSRELEQLQAAKIATKLSQQTAQAAHNHISVLTAALNNAKAVAEHADQASQEVSNQLASQSAMVGQAKSRLEQVEGQLQQASVDYEATKEAALKASSAAAEAQVNASKAAAHATSELHESANNNANDDDSYEDAQPFEQQHSPDRHHKKSKRSPLLKK*

>Woma_00010030

MQFVIVLTSLIALTMAADSYLYPMPLQQLQTPLWLVVKSQQKAQHPSYQQHQEIQQYFSQDTLGQYAYGYSEPLSRKQEVRTLDGVTIGSYSYIDANGLLQTVDYTADHDGFHVVATNLPKDTQTVPENIKETAEVAKARGEHLAAYKAILDGNPLASSILPKPVEDTTEVAQAKKEFFARFAAEEEKQKLLRKSSLLKNQYVNTNSSPLKQARPISTSINEKSTTFPSLKTSFDYGAGAISYGYQIGGNPVKTSRYYLPVV*

>Woma_00010031

MKICLTFLVLYGVSHAASYSLLVAPALRSYPLVYGRQQTSSIITPVQQQYHTQDELGQYAYGYSDPLSTKQEVRSLDGITRGSYSYRDANDILQTVDYTADDSGFHVAATNLPKPSQHSTFRITAGGPSVSHTISSAADDTTNTISASDTTVESNTSAGAFGETVYSSGGLRMAESSATNTKSFADPANVRSVELSKSLADTPKVALTSANAVLVNEHPHKLVISAPLAVASSVPVVKTLVPSKLYTYGYPLTKHFYSSGYYY*

>Woma_00010058

MLKLIILVLAAIFTVAVARPSHLSVIKTVPVVKAVPVVSVPVVRTVSVPVIKTVAAVPVVSSAVLGTSTIYGSSYGV*

>Woma_00010059

MLKLFSFLQLIWIFATLLAIASARPSYVAAPIVYSHYPVIKPIYTAPIVKSYHTVPLVLPYAPLYSAYHWGK*

>Woma_00010050

MFKYIVVVLAMLLGLACAAPNPAPVASPNPNPSIVPLGLPLPLGPVVVAGPRLIPGSVVVGPYGPVVVG*

>Woma_00010052

MFKLVVLSVLLAVAAARPGYLSAHGPLLYSAPATVIHEPAYAKVGAIVKSIPTAVSHQSISQVHSSAHVVQPIVAPVVKTTITAAAPLVHAAPLVHAGPVLSYGHGLHGW*

>Woma_00010012

MASQSTGVVVPRNFRLLEELDQGQKGVGDGTISWGLENDDDMTLTYWIGMIIGPPRTPFENRMYSLKIDCGDRYPDEPPTIKFLTKININCINQNGVVDTRLVPMLSRWNREYTIKSLLQEIRRIMTCKENLKLAQPPEGSCF*

>Woma_00010073

MNKKLSMLLVFALACALVQAGYLGSQLAYNSGFSTAAVPFAAGNFATAYGSAPFATPLPVAPFKGSHHPYAFGYSSQPVVKFRSFGPFTTYASVRAHHKFAYPAPVPSFYNTPPGVACDDAAPPVFYNDYPAHNTVFKFGPPITTTTTTYSTTAAAAAPVAPVAVANSFNGVAGW*

>Woma_00010070

MYKLIILAALISIAAARPGYLASSPVHYSAHTYVQEPAYASIGSIVKSIPTAVSHQSISQVHSSAHVVEPIVAPVIKTTYAAPVVKTIATPIVYNSYAATPVHNSYAAAPILKTYAAAAAAPLYDAYNTGHVIKSYAPTYGASYSQW*

>Woma_00010074

MRKFCTTILLGLTMALFATTTTMAQVVDYSIKPYNHVTYYHYPAPPKALQYNAPLPQTPRSVNGAAQAYQNQYQTYAESASPATKNNYQTTSTLTQKFISSALDNAAFNLALANQGFTIGNGDLTATSRSSKTSTQSVAAASSTPAPAPAPASDSSSSSLSSSTSPSPSPSPSPTPSTQITSLNVKLPVPIGITPLNIAPLPLQGGAPYTALPDAVTSYGTTYPQRKKR*

>Woma_00010071

MLKIIVLCLIISAVKARPGYLTTSLALPSYEHYTYEPSVVGHTISHLPTAVSHQSQTIVHEKRPYLRPIVDYQPAAAAYVKAHYAPAHTQIVAPLTYAPVAAYTSELYPAAGWDTGLAYAKGWNGWPLK*

>Woma_00010075

MFKFVAVFAACLAYASAGLIQPTAFAAAPAGVAYDNAPLYAAGGSQQVDVRHNYDGTFSSYTTNPFGYAAPYSSRYVAGAPAAYAAPARYAAPLTATPFAVGPADFAAPLKYATPFAVASPYTTPYAAHLAAAAYNAYANAYAASPVSAPVA*

>Woma_00010078

MHMKVAFVFLALAVLCAQAKPSLPFGFHGIPDHLDLVPPVLPPAHFHAEPLLPAPVPAPLLPAPAPLLPAPAPLLPAPAPLLPAPAPLLPAPVVHHHTHVVPAPTAILPPVYEPLPLVAPVPRYRAIPGPKTTTHHVSVGYAFPQPRLAKLHAHVHAFPHVHAAPLTLAPHHHHHTLF*

>Woma_00010079

MAVTFVILAALASLASQAQATNFQTFASTYNAHSINHAVAYPVGGSKVPVPAVAATPAVAAAPAIFAAPTSPGAATTSGAATPSAASTPSAASTPSAASTPSAASTPSAASTPSDGSTPSAGSSIPNASQSPSSGQSPAPANINTPAQVPFAGPAQIGFAATPFGFAPGKVTFAAAPAYGPAYHVGPAYAVAPAYAAAPAYAAAPSYSAPVYAATPAYAAAPAYAATPIFAPGSIAFATPNRVGSINAAAPASVAFAAASSKVAFTAPYNPYAVW*

>Woma_00010051

MLTKVIIVILVVFVALTAARPGYLHGHAHAHLDYALPHYHEPLHVAKYVHVPAAISHQSSTVIHSAPIIKPVVVPIVKHVLPVVKTYHPAPIIKTIHPAPLHYDPFYHHHFDHHDLHHFH*

>Woma_00010054

MFKLVVLSALLAVAAARPGLLESPVVYSAPAAATVTVQETGLAHVGNVVKSVPSAVSHHSNSVVHSTAHVVEDVVAPAVKTTSYTTKSIATPIVESYAVAAEPTVVKTIAEPAPIVKTYAAAAPVLQTYAAAAPVVHSYTPVTTYAAASPLTYTATGVW*

>Woma_00010055

MFKLLVLFALFTVCLARPSLIASSPYAAYGSAPVATIAYQQPALATVGAVVKTVPTSVSHASHAVVHKSSHVVQDVVAPVVKTIYTAPITYSSYAAPVASNVAPSPVAYTSAGSW*

>Woma_00010056

MFKLVVLIFAALLAVVSARPGYLASQPVAYTSHVVQPVVAAHYAAVPVVKHVVAPVVHTPVVHAVAAPVVHSYAAVPVVKSYASALVPLNKH*

>Woma_00010057

MFKLAVLVFAAVFAVATAGHSYLASQPVVYTSHVVQPVVKTHYAGVPAVKHVVTPVVHTVAAAPTVHSVAHHTVAAPVVQTVQQHSYVAIPILKSFAPAYVPLKKY*

>Woma_00009981

MERRVKLKSINPHITCKICGGYFIDATTVTECLHTFCKSCLVKHLEEKKTCPTCENVIHQSHPLQYISFDRTMQDIVYKLVPNLQEDEMRREREFYKSRNIACPKDLPQNHDDDNEKVMEAHAESDFHRLDEQVNVCLECMSNNFKNLQRRFIRCSSQATITHLKKLVAKKILNGIDKYREIDILCNEELLGKDHTLKFVYVTRWRFREPPLRLQFRPRVEI*

>Woma_00010093

MWKFAVLLIGLSGYCLAGPVGLKRNMPIVQQREDVMTIDNMAVEAEPAVASVDPNVIMSTLNLPSNATSIRADITDSFSCENKTYGYYADVDNDCQIFHVCLPVTYANGKENTFRWSFICPEETIFSQDSFTCMRPDDMTISCEDSYQYYELNRNFGMMESQADKEENNVVAASENVSSNEIVAEAENVPQAKPVILEQQEEEEKIDIKPIGQPRPIIPIRRKPGVAFATQKKPLTTNYPLRKMPQVQPAMPEKPVEQVPEEVQQSVKPVEEEEEEIKPLMQKFSRRPVPVMSNTFKDKIQNSKPETMIGVRTELFNQKRKRPTMFNKKPVEAQEEEKAEQAKIESQPEQQSFSAVESEMSNANTETQVADSEIQASEINHIFVKPEIDNETPQIIAEAEMNQKPVQVLEALEEIPAVISEVVEIPKAEEQEIKNEETLPIAAAEQHVESAAKPEVAEETKMAQEIQTADETPEEAQIVEEEKQTAEEEMQTVEEIQPVEAAEETHAVEEIQKVEKETPMVEETQMVVENPESEEETQMAEETPKSEETPEVEVTQNLRSSAEVTKTVEEVQTAEEHQAVEESPAVEETQNVEQSAEETQFAEEVKPVEEVQAAAEAQTVEEVQAADKAQTVEEVQVAETVQNADETQTVEEIPEDADQVQVIKDVPVVQTAEDTQTAEEVSVAEDVHTADETPQETEKTEETQSVEVFQNNEQVPEKQEDEKTEEQEQESEVSQPAQETLMVEEPQPIKVAVAMEDTKTSQESNTLNEIKPAEVSESLEEIKVAEQAPEETEKMQSENNAEPAIFEENSEEAANLAPAQTIEEMESEKPAKAPESLQATEEMSQPDAMVQQMYEDITDNKHSIGGFKPVDPVMAHEAEQLIADFINTLRKNDFTNETPGMHMEHHIDEEPEQQSQEGVAELEESEKPAEVNEAVAVKDKMNEEENHESEPQILETQADSVENNEEEAQNVKEIEEPTQEVEETQNQLKSLEAQSVENMEDTSKSEEVQPEIINMPQEMYQIPVQVITTPLEEVVQEQAKETADEAEPTQISENSVKKTQEEMPAMTMGGYKPLSIDDIVELVKERLDHMPKNEMETPMQLEQVLGNTQEDKAPQEDEEEQKVKKTEQQQQQMAPSEEQPQLQEEVAQQSEEEIVVPIYQRFIEPLKVDAGVQTESENEQTDDNEADVQEKSSRSYSSRSIMKKLDPRKRHFLFKTESS*

>Woma_00010045

MYKTIACIAIFCLAAVSAGLIETHHVVQEPVLAKVGTVVHSAPSAVSHQSLTRYHNKAVVTPVVAPVVKTTVHAAPVVSVVKSAPVVHTYAAAPVVHSVPVVHAAPVVHAAPVVHKTVYPAAYTLHH*

>Woma_00010044

MYKSIACIAIFCLAAVSAGLIETHHVVQEPVLAKVGSVVHSSPSAVSQQSFTRYHNKAVVTPVVTPVVKTTVHAAPVVSVVKSAPVVHTYAAAPVVHSVPVVHAAPVVHKTVYPAAYALHH*

>Woma_00010043

MFTYVSFFALIAVCVAAPGFISETHVVQPVVTKVAHAVPSAVSHQSISQVHSKAHVVTPVVKSVAAPVVRTYHAAPTVVKTIEPVVPAVKTVHTYAAPAPIIKTVASPVVHTHAATAASVIKTIESVVPAVKTVHTYTASAPTIKTVHQSLPVVHTYAAAAPVLKTFEPVNKTVATPVINHAVSHQSHAITHQSHVQIHQKATVAPAVVPVVHTDPVVKSLYVH*

>Woma_00010042

MLLFILLLCAISCQAGILYSEVPVTTSITNIHSPTAFSHQTITQIHNKKHEFAVKQPPSPIGYLTKRNLINTELISPLDDSSEQIDKKELNSTSVSSMSFTDKTPPVFSSDTHTPAHLTYAARPVVYKMLPLIKSNPFTSYYSSLESSLNIKF*

>Woma_00010041

MKKLQYLHLLCGLFLATLSGFFKPISTQPLANPSALVIDAAPSVVSYQGSSQNYAHLVISPRGRALGYVAPTNLNHTVHHTEKVKDSLDFLDIRPIYVPNN*

>Woma_00010110

MFKFLAICFLAILANVLAKPHLLTTGVSFATPAVVAPVVAQPVITHPVVAATAVRTPLVAAAGYYPAYTAYASYPHLVRAAHFIRRKRKFFFCIFFKLKLFFG*

>Woma_00013538

MKFCTEVFLLILITLSKGYNSQTLKTEFNEPRQSSFVEWLISLFEFTPATVAPEHIRPPFTVRPPFTTAAPTTKCQTCSCGSPNIIKRIVGGEETQEIRYPWMTLLKYNNRFYCGGSLVSDQHVLTAAHCLRGFSPLAISVSLLVHDRKANTSREINRKAKKVYVHERYSPYNIDNDIGIIHLAETVQMSSVLRPVCMPQQNETFEGKIGIASGWGATSEGGPLAQKLMQVSVPIITNEECNKKYGENRITENMLCAGVPEGNKDSCQGDSGGPFHFHNEGLDVYHLAGIVSWGEGCARPDRPGVYTRVNRYLDWIENKTKGACRCDMPSKKLAAEINWE*

>Woma_00013537

MKFCRETLILILITLSKGYNSQLLETELYEPLQSSFVEWPATAGLTAPLTTETTNCQTCFCGSPNIIKHIVGGEETQEIRLPWMAMLLYNNQFYCGGFLIFDRYVLAPAHCLSGFSPSALSVNLLIHDHTTNIFREINIKAEKVHVHEGYSPYNMDNDIGIIQLEKTLEMSNGLRTVCIPQPNETLEGKTGIASCWGATLNGGPLTQKLMNVPIITNEECNKQYCENRITENMLCTLVPEGNIDSCQGDSGGPLHVRNEELDVYQLAGIVSWGEGCARPDRPGVYTRVNRYLDWIENITKDACRCDMPSKKLAADIYWE*

>Woma_00013535

MLINLKYLTLLVIVLTLIANVHSVRHKTTPINEEQEQTLRQSQNTFVQWVLSLLPQRPTLANLTGTFTGPAAATSTTPATPSETSTTAQPEKTTEQPSPEPTATPSSTTSTTTTTTTPKPSPTPVMPSTTPLTPPDLTPPRNCSECYCGVANTQKRIVGGQETEVHQYPWMSMLLYGGRFYCAATLINDQYLVTASHCVYGFRKERISVRLLEHDRKMSNFLKIDRKVVEIITHPKYNARTYDNDIAIIKLDEPVEMTELMRPACMPTPGKSFKGQIGTVTGWGALKVGGPTSDTLQEVQVPIMSQDECRKSRYGPSRITDNMLCAGYDDGKKDSCQGDSGGPLHVVAPGTREHQLAGVVSWGEGCAKAGYPGVYARVNRYGTWIKTLTKNACLCQSETKKIKRELV*

>Woma_00013546

MTLPATSALCGIFTFLLAGSPIFYSPTPRGFCSNCCEIKERDDIKFMLYTRKNPFKFQYLYVSDEKRLRKSNFDFNYPLAIYLHGFSESALGERQSSQEIKDAFLWGGNYNVILIDWSPMTAVPWYSNAVDNLPVAARYVARFIRFLVKRGYQVQNIHLIGFSLGAEVSGFVGKQLQEWGIYLPRITGLDPALPLFEDGSSNRRLSPKDAQFVDIIHTDGGILGNPEAMGHADFYPNGGHALQPGCARQEIANNRWLGIIIGCSHQRAWEYFIESIRRPFAFPANRCEPSKLFGTCRDGNGKAFMGMGADRRLRGKFFLDTNDEPPFGRNSLGAAITKATKKNNQRKSSTSSAYTVLPSIQKQLKQELQYQQQQDIQDDEQQANNSNNVTTENMQETDTLMDVTAAIKLQHDFTTATTTQTIVTSIEAIANKLPTADSWNSVGHIVSSRSKTNLET*

>Woma_00013544

MANKVFYSCLLASVFAVALITLTQADISEKPVTDVCLGCICEAISGCNQTAYCGGGVCGLFRITWAYWADGGKLTINNEDPASETAYANCVNDPYCAANTIQNYMRKYSQDCNGDNAVDCYDYAAIHKLGGYGCKGELTYQYANVLNTCLSNFGGINVRSN*

>Woma_00010162

MIGTILAFFSLISSAMAGNTERTFIMVKPDGVQRGLVGKIIERFEQKGFKLVAMKFMWASKDLLEKHYADLSARPFFPGLVNYMSSGPVVPMVWEGLNVVKTGRQMLGATNPADSLPGTIRGDFCIQVGRNIIHGSDAVESAQKEIALWFNEKELVDWKPAAVDWVYE*

>Woma_00010114

MVCKAFVALAILVSVVSASYVPIYHSTALEQHVVRIAHKILTKPVEKFDHHPQYKFSYSVGDKLTGGSKSQYEERYDDVVHDQYSLIDADGYKRTVTYTADDHNGFNTVVHREPFVKHKTSEMDISSVVIIKLAYKQQVKFNHDT*

>Woma_00010137

MKSQLVYFVLVGIAVLAQASLALKEEDCEVCVKTLRRFADTLNDDTKKDYKLIETEFKKFCKTQKNKEQRFCYYLGGLEESATGILNEMSKPLSWSMPAEKVCEKLKKKDVQICDLRYEKQIDLNAVDLKKLKVRDLKKILNDWDEECDGCLEKIDFIKRIEELKPKYVKTEL*

>Woma_00010130

MQPQNLNKLAIFASIISMFLLNLLLKTAAAKENIPTYIKDDGELQGREKKDNYRSVQQQQQQAHFSQHRTQQNHHYHNQHNHQHQHQTHHTQSASHHRQQRQRNSGPSSSQAYKRYSRDSTAYNSRKPNIILILTDDQDVELGSLNFMPRTLRILRDGGAEFKHAYTTTPMCCPARSSILTGMYVHNHMVFTNNDNCSSPQWQATHETRSFATYLSNAGYRTGYFGKYLNKYNGSYIPPGWREWGGLIMNSKYYNYSINMNGQKIKHGFDYAKDYYPDLIANDSIAFLRSSKQQNQRKPVMLTMSFPAPHGPEDSAPQYSHLFFNVTTHHTPSYDLAPNPDKQWILRVTQPMEPVHKRFTNILMTKRLQTLQSVDTAVERVYNELKTLGELDNTYIIYTSDHGYHLGQFGLIKGKSFPFEFDVRVPFLVRGPGIQPEKVVDEIVLNVDLAPTFLDIGGVPTPAHMDGRSILPLLLNKQKSIRDQWPDTFLIESSGRRETPEQIAEARARLQMERSNMKLANSTLIEDLMTNVTTAPNTHIDVVDLESKEEEQFNENDKVTDTDTDTEDDFEFDNEDDLDTSTGNLEDDDEEEDLDEELEQQDQFDNNLPLAPYITKMMRLNSECSDSKLLENCRPGQKWKCINENGRWRKHKCKFHLQLQHHLAEITKYPKHQNKRKCACFTPDGVVYTKIKQENFKVDQRRVRKRTQSFGKSNRRHKRDTEHFDIEELYHTELPYEMEELLDLHGNLNNLEKHLLQENEERAKHLRVKRELTANLFDNPDTLNSDSDESKKNSSNDAISKVIQEIQDTLETLELKFVEHDWSVNDTVKTSTKLTSAGFVRGSKFPKVGGRVGTRCYIESETGKVNCSDVIYDDEKTWRKSRSQIDMLIKVLKDKITHLKDIKKQLRETKQQQLQGRYWNNEFVTQSRDDVSNDDLPYITNEYHGPKGNRRRRPYNHHNHHHHGRHYPYNVGPSTNGGVGGNKRKFNNNTQHNTFSHISKNIHTEEFMDSQMKDKQFNNVPNILSTTTTTTTPQYEEDVTTVNSYEQFNQTFQSEEYEESKTRNNKKHYLDSTTDFNVATETAVRNPYNSKGQKEVIKNTYHNGRQPINDPYQSNNNNNQFSGPAECYCEPEEDIYPDSKEIAREARRKLKEERQRKKERKRIKKARLEKECLSEKMNCFSHDNSHWRTAPLWNDSPFCFCMNANNNTYSCLRTINATHDYLYCEFTTGLITFYNLKIDPYETLNRASSLTSAEKSYMHDSLVKLKSCKGKSCTLKKPLVSPQNISTTINGIVRGTKRKHAGAISTPLNGNAGFVTNHMDNYEGPQVKRRKIANRWSNTNNLRRRPWKQQQPPHSNPHHSYYNRQQYGQHLRPLYNPAHNQNRLSASSQLHTNIEQNISLRRNRFHQDIQHQQQQQKYLQPQYKSENDKRQQQQPQKSITMKNNTIDLKVTHQNPIEIKTHHQQYTQQHDNNNNTASSALVSSTSYSSSSPSTLASTVTKPTRTTTTQLYVSLPQQQPTAKSSSSASGPSSSPPSTLLERNL*

>Woma_00010139

MDLHLKSIKNCKFLLVLLLLSYCSLATIQGLTFINEQSETKQFTFASDDNNGAQQKQQQSTNDATGLTRQLITGITPNIVNTASHCICVPAGSCPNPLPTPPTDGSGQIDIRIVNNPVVVTTPTPFTCNYGLAVCCQAGPYQCGRRYPPPPGSTPAGPGQAEFGEYPWQAALLTTNEVYLGSGALITAQHILTVAHKVYNIAMTSFKVRLGEWDASGTGEPIPAEDIKVSNIYIHPNFNKDNLQNDVAILKLATPVALGTKPTVGTICLPTVSFVGQRCYVAGWGKNDFGPTGAYQAIQREVDVPLIPNADCQTALRRTRLGASFVLNNTSFICAGGEAGKDACTGDGGSPLVCETNGAWFVVGMVAWGIGCATAGVPGVYVNVATYLPWIQTILAS*

>Woma_00011759

MIKVTLLFGGLLVLCTAAVIQTSDSNIVKGCESETELWGSPDGTKFLFCLGDDMALVMSCDPGTFFVKNATVSGCVPLDEVDDSCVYHVERPTCDGESLSQPQPHTDPTQFYLCPGKDAQPVALPCPSNKAFVKQNGYLGCFEWTMWRKIRGCIDTK*

>Woma_00011761

MNILILIVIISTLIACSQAQIVHGNPLYTGQPGCQTEEEITVALYPHFRNKRAYWRCSVLGQPAAFELCPIGQGFLAPEKACVPWLSWYWTPTEAPPSSPLHQAPASADATEQQQ*

>Woma_00011763

MKIFILIAIVAAAVYADVENTSFNGRPGCKDRTEVGLTFANFFDSSRYWLCVSQGTQAISKRCPNQFGYQASIRSCIPYKIWKWEEPIMPITCPDFEQECIPSGVAINNNW*

>Woma_00011762

MKTFIVILSISLLVNLAFCNPLNNGEPGCQTEEELTVKYYRHFQNKTVYWECVALGQPAVLQSCPVAHGFLDPAKECVHFSLWYWTPTVAPPSAPAAEAATTVI*

>Woma_00010245

MANKYLIFVTLCALSLIALTLASGDCPSSTKVQNCTPKCLHDSECSAIGGKCCPNLCNGRSCVQPNLLSNAGSRDTSPFSKNSGSSGSYCGNVKCSAFERCESDRSTKRPKCVRA*

>Woma_00010247

MNLAVLLILLAHVLAIQANECPSSTKIYSCSPRCYKDSDCATIGGKCCPNLCNQKSCVAPHMLSNSGDKYGTKPDKYGSGSVYCGNVKCGPFEKCEVDRTTKREKCVRA*

>Woma_00010241

MAFKFITFFALLAAANAGFVSPVTYAAAPTLVKYANPAVDAVATTQHNVVRSFDGTVSSYSKSVDTPYSSVRKVDTRINNNVYTPALAKTVTYAAPAVTKTVYTHDAPAVTKTVVTPTVYTHAAPDVTKTVYTHESPVVYAQPAVTKTVSYSAPSTSYNHGPAATTYTHNAPAVSAYGSSQTVHYSPAEQVSHMSFDGFGTHWGF*

>Woma_00010240

MAFKFITFCALLAAANAGFVPPVTYAAAPTLVKYANPAADAVATTQHNVVRSFDGTVSSYSKSVDTPYSSVRKVDTRINNNVYTPALAKTVTYAAPAVTKTVYAHATPTVTYAAPAVTKTVVTPTVYAHTAPAIAAPAITKTVYSHESPVIYGQPTVTKTVSYAAPSTSYNHGPAATTYTHNAPGVSAYGSSQTVRYSPAEEVSHMSFDGFGTHWGF*

>Woma_00010243

MASFVYAVWSITNVASVHCSVRPEISPCTCEPTYAHNYVELSCEKVDSFHTIVDALSNKFEPYVNISLKITHSQLEDLEMRSFTDMKLNLVKLRMLWNGLKSVPELPFRGLSNVEYLGLGDNELEEIPKHMLNHMPVVKTLDIGRCRIRAALQDDFKGTQMLTNLFMPTNNLTRLDRATFPPGLVTLHLGRNNIENLNGTLRHLNKLESLFINKNRITTLEDELPESNRLKLIMAQNNMLVHLPERMRFMDKLETLHVQHNHLVSLDRVLKNAKNLNEFYAFNNRIESLARDEFQMAAKLEELYLAGNNIKSLNGSILPLQRLCKANFSFNEIDEFSMDEIRGLMSLKQLDLSNNRIRKLAGSQENNIDKNSYLIEFYLDFNELTTLDGALMGLNSLRILGLTHNKIERILPEDFIGLEKLEILELSHNKLLTLREMQMTVLPSLKILKVDYNNLTILDKDFHGLPVLCQAHMTHNQIISISHELVAKTNCMNHNVPGKLELHLEDNPFNCKMLLDEYCPLIGNSEKRLRLRTKCFEAYEDVCKNRQFLLMVPTNDLTQQVIPVILPSTDLLKKPDKFLPPLFAPLASGQAKINPLILAPAIKILEPTTTTTTTTTTTTTTTTTTTETPIITINTTTAITHPPTTLDHQNETLEHLDVTINNETVIQALNINDHLHMETQPISNQNSTQLPINELNEQQGTTTQKPSILVLNIEDSQPSVITANSSAGDIQPNNNVPKTEYATVEYIANIASKGNHEMAASPPDVLMDAVDTKSNSIHEEFHTKVLTDYDNAPQSLLQPDEPPENP*

>Woma_00010201

MFASTKVLLLCMALIVVVKAVPKPDEVPKHNPLEHDSLHAAHFANGHHNPQYDHEQFLGEDEAKTFDQLPPEESKRRLGLIVDRIDEDKDGFVTISELKRWIEYTQRRYIDEDVDRQWKQHNPDGNDTISWESYRHDVYGFMDALTQDEKEYEENGISYQSMLNRDRRRWAVADQDHDDALTKEEFTAFLHPVDHPTMRDVVLTETIDDIDKNKDGKISIEEYIGDMYTAPEDGAEEPEWIISERETFAKFRDVDGDGFLNREEVRAWIVPNDFDHAESEAKHLVYEADNDGDEQLTKEEILDKYDVFIGSKATDFGEALGRHDEF*

>Woma_00010246

MAFKYIVLSTLLAVASAGYLAPVSTHYAAAPAVIAKVANPAVDAVATTHQNVVRSFGGTVSQYSKSVHTPYSSVHKEDTRINNNVYTPAVAKTVTYAAPAVAKTVSYVPAPTAAVYTGHSAPVVTKTVYEHHEPAHNVYAPHMPHTVYTHEQPAHAVYAAPAPTAAVYTHHHEVPTTATTTYNHGPAATTYSHNSPAVSAYGSSQTVHYSPAEMVSHMSFDGFGTHWGF*

>Woma_00010227

MSHRIVLLTLIILVIITSTWALKDYKNYKIYEIIARNRRQLNSLYDIVKPNRDINILNLARNKKELSKVLIPPYLEKNLKRTLENYALNYTLLVNDVSKIIEKERRENRENIPPNYPLNVFKRYYRHHEINENMDYLSRSYPSRVFVKTFGYSYEKRPLKIITITNGDGRSDKKTIFIDAAMHAREWITPAMALYIMHELTVNYELHKHLLKEYNWIIMPIVNADGYEYTHEENRYWRKTRRPNKLEPECYGTDPNRNFDFYWGREEGASLDPCDDTYAGEKAFSEPETQVVRDILKSYAEHMAWYLSLHSYGNYILLPYGHTNVLPDNYFDMMDIADAGSLAIVMSTTSIYTYGNTYSVMYPTSGDSADYAIGKLKIPLTMAMELPAGGFSGFDPPVRQIKDIVMETWIGIRAMAERVVEKY*

>Woma_00010221

MKIFLRDILLLVPIICLTYKLANAVCNVCQENNAACVNLTSYHLCFGGVYDTEQLFTCPDGLVCTNLPNICFQRSTLPASCGDTSSCRKCNSNQVFACTSRTTFAFCFGATVPSDVVGTCPWGKICDASSAEFCVEQLTPTSIVCDLEDPSLRTDFFYYDEDDDDK*

>Woma_00010229

MLRLGIILLALAAVQTLTNAAGYDGYKIFEVVPKTQQQCDFLYELSKIEEYYDFFVLSKLPNHLARVMVNPNEEENFLSALARHNIFYRVVNDNVGRSVEHEFLMSQINRSRMPFTGKGRLSTDRYYSHGEINAYIEDLARRYPSRVAVKKVGVSYEGRDIKTIAITNGDSRRQKNVIFIDGGFHAREWISPAAVLFVIEQLVKNYKENEELLLDYDWVILPMVNPDGYEYTRSGTEHRFWRKTRRPYPYNGRVCYGADPNRNFDFHWNEEGSTQSPCTDTYAGPKGFSEPETIIVRDLLHSLRGRGIMYLTVHSYGNYLLYPWGWTHDLPDTWPDLDEVARTGRDAIYNATGAKYTVGSSTNVLYIAAGASDDYAFSIGFKISITIELPGGGIMGFDPPASSIDNFVKETWIGIRAMAAKVVEKYPTERVGFL*

>Woma_00010228

MTTKAIQVIVFLSSLFIVSAYTLARNTRTSQNYYKKYEGYKIYELHFQHQNQSEKFFQYFPNFINDSSSIITTRHHETTHSLLVLIASGNETSVIDYLQTNKITFKIVNHNVGKSVRKERRQSRTAKKFSSTSNLIDFQSYHRYDVIINYMEYLKVQYPKRVNIINMGKSYEGRDLKAILITNNTHRAVDEKPLILIDAGIHAREWISIATALYVMQQLLENSSSNEEELNMYDWLIWPLVNPDGYEYSHDYDRLWRKNRRPSNSSTNCVGADLNRNFAYQWSYSGVSTNPCSEIYAGPVAFSEPESQALRDLLLKIKQSCRFYLTLHAYGNYLLYPWGYKKSLPPTWPYLNAVARAGARAVKNFSGSFYAVGGAGKTLYPASGGSDDYALAIAQIPVVLCMELPSGDNGFDPPSDKIQSIVEESWYGIRAMAIEVNDYPLRNGAKCNAFSFIVILSAIYSLWT*

>Woma_00010210

MSSLRYATLFVFVLLNNGIVFIKAETFDECDGVDPESYVISADTCASYIYCNGDDSELFECDEGEYFDVEAGSCDAKENVFCPLDEEGSGDEEPDEPEEPEEVTTAAPVVTTTTTPEPITTVNTPVEVIDQLPVVMDTCPSIDDPNRIILTASSTSCSDYYVCYHGHALEMHCMDHLHFNSLTGKCDFPENSNCKITNQPTTYVNKCLPHVTTFYPHPHNCNYFIYCIKGYETIQQCPFYYGWDIERRTCVHMKHAKCYVNTN*

>Woma_00010211

MLLIIRISIVLLIAAALGNALTISQICKSKKFGYKIQNPENRHTFYVCLGNLGQFSLDCQSGYHFKETEQKCIKESYPTCPCLVGSSYNEVQLTGFIPIIPYKLLFLPHNVTQQKLAIPKKIAKAVGEGLNQDIMQTLADSKRETTDPGKYSDISTETKSSNINEKLLLGKSATLNVGEYKSTIQFRENVLVEETTVGATATTERTTESIIDFTTEQTTTVYTTETATETTSESNTENWPDSTLASTVEILTTTQLPTETNTTPTVEITETTILTTTTDIPTTITTSEAVTESTTHPTTINESTTIEAEPTTTVTESIPPFEVTEASTTTIASTLTTTISPDATTESTTGTYTTSESTARTETTTNIVGTETTSTTQTLSTTVEPPDTTTAATTEATTEPTTAESTIQPLSTTTTEETTETTTATGTEATTEESTSPSSTTVAPIETTTAHTPEMTMKTTTVKTAETTTEGSTTQLPTTTLEPSTTAMESTTTTIVELTTTVKPTTTIGPTTTVEPTTTVEPTTTGEPTTTVEPTTTAEPNTTVEITTTVEPTTTVEPTTTIEPTTTVEPTTTVKPTTTIGPTTTVQPTTTVEPTTTVEPTTTIEITTTVEPTTTVEPTTTIEPTTTVEPTTTVEPITTVEPTTTAEPTTTIEITTTVEPTTTVEPITTIEPTTTVEHTTTVKPTTTVEPTTTVESTTTIGATTTLEPTTTVEPTTTIEPTITAEPTTTTVCITTTTRICTTTTSTCTTTTSTTTCTTTTKTTTFTTTTTTTTCTTTATRCTTTTITCTTTTTTTTCTTTTTCTTTTTTTTCTTTTTACTTSTTTCTTTTSTTTCITTSATTCTTTTSTTTCITTTTTTCTTTTTTTYIITSTTSSSSTTCSTTSTTTACISQTKSTSVAKESESLCNNKPNGYFVKNPKSCRNYYTCYNKRAYPQVCPGKFYFDDKLKMCNYPELVDCLEEKENSRMKGPFTKGRKYMAENTQTQPTMLNYSPNDCKALKSGTFIREPGNCNKYYVCLNGNAIALYCPRGQYFDIEGYVCNFKDLVKNC*

>Woma_00010189

MVWAGFSSHGTLELYFPSTRMDSLEYQGILSERLLPYLAQNADKTFNFQQDNAAIHASKSTRQWFAEHNIKVLSWPGRSPDLNPMENLWGLLVRNADNKQYRIKDE*

>Woma_00010184

MSTVLQWPFVLALVLIASLCRVADSKVFERCELAKTLNKRHRLDLREVAQWVCIAQHSSGFNTAVQAFSPSGGSHGLFQISDVYWCSPPGKGAGCNLPCHKLRDDNISDDLSCAKRIYDEHQRISGDGYTAWNAYQQFCRNNAFSYIQDCFASETRITPSYAAPAKGYHSHYSGNAIQDYSLKKRGKVYGRCELASELYHKHKMPMEQIPTWVCIAEHESRFDTAAVGRLNADGSADHGLFQISDLYWCSHDRYSGGKACGLSCSKLLDDDITDDVRCIKRIHNEHTAISGDGFNAWTVYRPYCQNQRYEQIASCFKNTQQHKASANKLSVANSDIPFFQNVVQQIPQKTHYTTSSTSTYNNIYKTSSNTNFNSASSRVPSHKGKVYTRCSLAQELYFKHNFPMQDISTWVCIAKHESQFNTAAIGRLNTDGSADHGLFQISDKYWCTHDDYGGKACNLPCAKLLDDDLGDDVQCIRTIYEEHTRISGDGFNAWTVYRPHCQNQNIDHIKSCFDNRTLESNEVVKQKPFQDVVSSKGKGKVYSKCELAQELYHKHHMPMEQIPKWVCIAKHESSFNTAAVGRLNTDGSADHGLFQISDLYWCTHDQYGGKACNIPCDKLLDSDITDDVKCIKIIYEEHTRISGDGFNAWTVYKPHCRNIEINHVNDCFTEQELKLATTTVSTSNSNSLIPHSSSGTKYSPKGKIYKKCELAQELFHKHRMPMEQIPKWVCIAQHESSYNTAAVGRLNTDGSADHGLFQISDLYWCTHDQYGGKACNIPCEKLLDSDITDDVKCIKIIYEEHNRISGDGFNAWTVYKPHCRNIEINHVEDCFTEQELKLATKTVLTSNSNSLIPHSSSGTTSSPKGKIYKKCELAQELFHKHRMPMEQIPKWVCIAQHESSYNTAAVGRLNTDGSADHGLFQISDLYWCTHDQYGGKACNIPCDNLLDSDITDDVKCIKIIYEEHTRISGDGFNAWTVYKPHCRSVEINHVKDCFTEQELKTYNEVSTTVIGNSIPPYYATSTKNIPKGKKYNKCELAQELLSKHKMPMEQIPTWVCIAQHESSFNTAAVGRLNTDGSLDHGLFQISDLYWCSHDQFGGKACNIACDKLLDSDITDDVKCIKIIYEEHTRISGDGFNAWTVYKPHCRNRKMEEIRSCFTPNQIEEYDKKQTFSYKTSNYQQAQQTPSKSSYSNNPFLSGIKTQNSQPSQKQAVKQDFQLVSANQKPSYANNPFLQNIKTPPSNEVHKTTVKVTNNLNAAVKQDYKQNPFLNGSLKPLATTTLQNYQQEDNQSKTTYNHNPFLNGLISSTTNSKIVLKPTEDISNEPYKTNPFLSVVKPTQSSLQLDTFSKATTSFNKYENKYINSNAFRSTTTTTTLKPKTTATIKTTARTTTRKPTTKNPITTTTRKTTSSSTTSTNPTSWTTTKKPATTTKTPTTTTTRKTTKPATASWNAYRTTTKTPTSTTTRNTTSTITKSTTTKKPLTTTTTSKKPLTTIKTTTKKSFTTEPVTTKRTAISTTKAITKPPRSSTTAKWNYQNQRYTNTATIPQAVTSLNAYKLTATTSKPERTSWNWKQEQSLKTTEKPTVIQKPTTWNWKTEQNSKATTTQRTTTIKPSIKTTASWQKYDKITITKPSSTTTTLKPTTKLTTKKPTSTTTTPKPITKTTTKQPNIATTTLKPTTKATARKSGSSTTTTKPKPSTRKPAINTTTPNTTTRTTTRKPNINSTTSKSTTKATFSWWSNTAKTNTAKTTKSSALTTTKKPITTFTPYSNITTTKPTKTSKLFTTTTLKPLTTKSEFKNDYEKYKKDPFSHPFFAKVNKQIEQLKGNTTTTKQPTFTATKLQSLPHSALYQDYKNSLSNKTKTILAYNFEVTKPTTTRRPRL*

>Woma_00010230

MFDTRRRLIVAVVATILFVTSKAESYEGHKLYQLKAKEVDQLKFLQNLAKTEKVYDFLNPHTVIVGPKSQPGLESLLNQHAVNFEIADENVGKTLARNFEVNRLKKSLNPYKGQGRLSTERYYSHGEINAYLEYLAQTYPERVFLKTMGYSFEGRPLKALTITNGYGNYSKNVILVDGGFHAREWISPASAVYVIKQLVEEFHQNKELLEDYNWVVLPVVNADGYEYTQLSAETRMWRKTRKPLFHQGGICYGTDPNRNFDFHWNEEGSSPDPCANTFAGPRAFSEPEAVVVRDLIHTHSVKGQMYLTLHSYGKLLLYPWGWTNALPDTWEDLHEVAMAGADAIYLATGTNYTVGPSTSVLYVAAGASDDYAFNAGFPISFTMELPGAGSTGYDPPVEMIDDLVKETWVGIKAMALKVIEKYPVVYN*

>Woma_00010231

MFLKIVLSGLFCSIALAAGNSYEGFKIYDINAKNAFEKDILLRLSSNENYDFFFLPKILGQAARVMIEPEDQVEFETLLNKHGIDFTVINENVGKSIELERAENQFLLSAMNTSSGISFTHYLRVGEIENYLEYLAKNYPSRVIVKTIGKTYEQRDLKVITISNGDGNANKKVIFIDAGIHAREWIAPAAALYSIHQLVENVAENEHLLKDYNWIIVPVVNPDGYEYTHTNYRLWRKTRKPSSIICYGTDINRNFDFHWGEVGASILPCSDTFQGPKASSEPETQVMSDLMLLLSGTGKMYLTLHSYSSLLMFPYGHTSQHLDNYDELYSIGINTAKALKSRYKVGSFSEVLYLAAGGSADYAYGVAKIPIVFTMVLPAGGSGYDPEPSQIEPLVQDTWNGIKAMAEQVISKY*

>Woma_00010232

MILKIVLSIALAAEVSLEGFKIYDINAKNAFEKKVLLRQAAQVMIQPKNQVKFETLLSKFGIDLFVFTVINEDVGINYWIGIKTMGEEVISKY*

>Woma_00010233

MYLKIVLSVLFCSIALAAGDSYEGFKIYDINAKNAFEKEVLLRLSSNENYDFFVLPKILGQAARVMIKPDDQVDFETLLSKFGIDFTVINEDVGKSVELERIENQFIRSAVDTSSRISFSHFQRYNDILNYLNELAKSYPSRVIVKTIGKTYEQRDLKAITISNGDGRANKKVILVDAGIHAREWIAPAGALYIIHQLVENFPENSHLLKDYDWVIVPVANPDGYEYTHIKTRMWRKTRKPSINNCYGTDGNRNFDFHWGEVGASSLACSDTFKGTKAFSEPETQTIRDLMLSLTGTAKMYLTLHSYGNYLLYPWGYTSALPDSWKNIDEVAQVGAAAIKAATGTRYTVGSSTNVLYAAAGGSDDYALAIAKIPVAITMELPAGGSGFDPKPSQIEPFVQETWIGIKAMAEKVISKY*

>Woma_00010234

MRYLYGIIFIILILQQLSWSYPTDNENSSAGLSNSQQDNTATVVIADNGVEEAATAGQQDNKHNPENLRGLG*

>Woma_00010239

MAFKFITFCALLAAANAGYVAPVTYAATPVVKYVNPAVDAVASTQQNVVRSFGGTVSSYSKSVHTPYSSVHKEDTRISNNVYTPAVAKTVTYAAPAVTYAAQAPVYTKTLVSSPVPTVNTYAAHHAPVMYAHATPVVKHAITYSPADTVAHVSFDGFGSHWGY*

>Woma_00010287

MRELVLFIMNYLSSCRVVIVVLVLCFSQTNSQNVMSSTVTVDIPYQGIIQGDYCMTLWSQQTFMRFLGIPYAESPCGPLRFKPPSKRLPWRHVLDATKYGRRCPVMTSVLQLTESELEQDLEDCLNLCVYTKNLTTQVPVMFYIYGGGFYNGSNIDHPPQYLLEKDIVLVVPNYRIGALGWLATIDEDMPGNNPIVDVLLALQWVQDYIHLFGGDPKQVTIFGQSSGAAISGTLLLSPHTPNEYFIRSIIQSGSIFATWAINRKPVEQAQRICLALKCRNCESKKELYKCLREVNVPKLLECTSTETFSPIVGDFYGIFPEEPENLLKNYNRSVSIMTGFTQHDGSFVLASYYDTMKLSIVDLSTLDVRDFAKGMMDMANDSTGLSNNLLTKLLFNNNLLNSNDHKNATPAYFDLANIVAMKSPVITMAKNMLYRNLASVYLYSFDYEGSNTRFGYELGNDHYPFKGGVHHSNDNIYLFSTHHLNSEDTKMAMKMVEIWTSFAIEGNPKTEKDIEIFPMKTDSGPYFRINSEITLGHDIYEELTITIDDPNNDKLIRRDIEF*

>Woma_00010293

MSSKKCVFKPLIICLSCIQIASAVTMEQFEQSLDMMRNGCAPKFKVTLEQLDNLRNGFFDESSDELKCYTKCVAQLAGTVTKKGDFSITKAIAQIPIILPPEIQNTAKDALSNCKDIQKSYKESCERIFYVTKCVRDFAPEVFKFP*

>Woma_00010295

MHIKIHLIFIVCLTLINWSGAIETKDYIDLLELPDNDEFQWSRHANGATDVEKSNVTRHNISKRQAIAAGQLQENRGFGACRTPSGEEGTCRHVIYCRIPELKDDVWRLVSHLCVIQGSTIGVCCPSRIVGRLGPQVITDINNADLEEPRIVNHPEQRGCGITTKQFPRVTGGRPAEPDEWPWMAAFIRPGIPYIWCGGVLVTDRHVLTAAHCIHKFKKEDLFVRLGEYNTLLLNETRARDFRIGNMVTHIDYDPLTFENDIGLVRVERPTIFNTYIWPVCMPPIGESWEGKMAIVTGWGSQSFGGPHSDILMEVNLPVWKNEACREAIVERVPESTLCAGYPEGGQDSCQGDSGGPLLVQLPNRRWVTIGIVSWGIRCGEPNRPGMYTRVDKYLHWIIENSDI*

>Woma_00010280

MFAVRFLLFVVLISFWYSYAKAEVKDLQNCEAQLNMYRKLILQPIPSFEDVCDAYNTRSSTPIDTGLPPQVTFFGRYQPAQENTDAWSFFKFLMAQFNDIEFTNIIRDAVIERCRMKLQLQQQRDEKRNAVVLGKKQKFHSWGGKRSGDGNSYANFINGLKQEHNDDINLGF*

>Woma_00010285

MNIFIIFAIFVGINLCNADLNVVNQENTQTVQLPSNGVAVDEVVVESSLYIKPKQQRSQVRRVRRSIIGDFNNQRTFVEHKRVKRQFGQFGQTQAASNAAAHNHFNNQFGFGNSAANAQAQAFNAQGPLGSFGASAAGTLTQSVNGNPFGVQNSAGASHALTFKLPNGQSVNFASTNSFANGAYGNNANSRGSAVSINRI*

>Woma_00010380

MLPKILLICATVLIIGQVAFGHRFGYSAPNQRRWARHHGHCAGKTQSPISVHSSRTIPLHMPAVDMIGYHNVLPYPLKMINNGHTVSITIPKLSDEERDNGEFMPYIRGAKLPGDFEVEGIHFHWGDKNNRGAEHVVNDIRYTMEMHIVHRNKKYSTLAEALDYVDGAAVLGFFFNLDEDEGTGLTTICRHLHLIPEANTEVALNVTFSISSLIHGVDIDKFYTYKGSLTTPPCSEAVTWVLYPDPIPISPKQISRFRQLSDNQEGALVDNFRQLQPLGNRRVFMRDGNTHHTQIAKLNNEVDYRKWDWFH*

>Woma_00010366

MKAFILMSCLALAAARPEAGYAYNRPGGSGGAGGGGFGGGSSGGGFGGGGFGGGGSGGGGFGGSGSGGFGGGSFGGGSGGGGFGGGGFGGGSGGGFGGGSGGGFGGGSGGGFGGGGSIGGFGGGGGGGTTLVQKHIYVHVPPPEPEESRPRPNIPVGQAQKHYKIIFIKAPSPPSYQAPVIPVQPQNEEKTLVYVLVKKPEDQQDIVIPTPAPTQPSKPEVYFIKYKTQKDGSGGGAGGGIGGGIGGGIGGGIGGGIGGGIGGGSGGGIGGGIGGGSGGGSGGYDLGGSSGGGGGGGAPSSNYGPPGKSGPY*

>Woma_00010362

MKLFVVLLFCSLSLAKGDSILDSTDGWNIPTVDGPFTWVPREDYNLCLNTIIPDDMEINFLLYTSSNPTEPQLIKTNDTTALASSNFNPQYPTRFITHGWMSNCDYGPNSEIGPALLKTGSYNVLCVDWSSIAINPNYIEARCHVAAVGQLLAEFIEFLYEKGGMSFRSLVLYGHSLGAHICGYAGKLVKGGVIGIICGLDPAGPAFDVTDCTTRLCSTDAVHVAIIHTNGDNLGILEPIGTIDYYPNGGKNQPCCGADLTGKCAHARAITYCAEAISKNNFPTFKCDSYTNAVNNVCGFTYSNVLMGSTSSYDAFGYYYVPINCVSPYGYGA*

>Woma_00010328

MICIVKLLSLLIVILCLMPESQVSAIFWWKYCYKRCSNYENTIYTTDGTNCILHRNSCHLRRDSCLRRRNGLPRLSVSPKADCQAKCAKDCGTTNSPVCGEYAGTYTTFNNECLLLKTACENGQSIIKVRNGACTTKG*

>Woma_00010360

MKLLLILAVGCLAVSASVFDEDDSRIHGENGWYIPHEDGSFNWIDKERAEAYLEDMENNENFDITTTPVKFYLYTQANPKKGKKITASAKSIDGSNFNPANPTRFVIHGWTQSHLSSMNKDIQNAWLSLGDFNIIVVDWARARSVDYASSVVAVPRTGKKVAAMIDFLVSEYGMSLDTLYVIGHSLGAHVAGYSGKNVKTGQIHTIIGLDPALPLFSYDKPKKRLSSDDARYVESIQTNGGQLGFLKPIGKGAFYPNGGKSQPGCPLDISGACAHGRSVTYYAEAVAQDNFATLKCANYEDAVAKDCGATYSSVRMGADKNAFMVSGEFYVPVNKKAPFGVMA*

>Woma_00010361

MKLILILALGCLTASATTFEVDEKRQHGENGWYVPQKNGSFEWIDKDVAESYLASMEKIDLFGLTTSPVNYYLYTKSNPKKGQKITANNKLIDASNFNPAHPTRIVIHGFIQGSYSLMNRQIRDAWLNLGDFNVIIVDWARSRSVDYVSSVVAVPTVGRKLATLIDFLVTNYGMSLDTLYIIGHSLGAHIAGYAAKNMKSGQIHIIIGLDPALPLFSYDKPDKRLNSKDAFYVESIQTNGGILGFLKPIGKAAFYPNGGKTQPNCPLDIPVCSHERSNTYYVEAVTQDKFSTIKCNTYTDAIAKNCGATYSAVRMGADTNAFMVEGDFYVPVNKKPPYAIMS*

>Woma_00010369

MARFITKLLLLFSLFNLIVCPYGQVYNERQNRALYIESNEHSAQYLTESSSPVGSYLRKGHYRESNANYRNQEQQVEAPRTSTQFYNQANSQQYNNDDSTSTDSAIEKNIRSVFSTNFGPGLHIFPNTAPFVHQPHLNSYTDQMIGDQPDPYSSSSTMSSSDKEFKVQRGSNGELTVVFKEDFVPKKETDTTLQGSDNDNGSSNNDSFENKYQNRFPSGHHLTDDKLEPTPPGFNRRRDTAVVNTVDCVEKDAKSGKPFCTQLRNYPEKSHLEEIIKTKFSNLESFFGEDLLQPLNISQRMNNEPIEEFLCRSRTRVIYPEAGLNKDYDWLFIINTKKYKQGVRIEECINEGSICGESTGLTLPNRYTATCKQSYIYRSLVAYANETIIKDQFKMPSCCKCVLTVS*

>Woma_00010359

MKCYLLLALFVGSTIAAAIPEDEHIHGENGWYIPRGDGSFEWVDLEEAEYNLKASSLMEGRLSFNPVTFYLYTRSNPNEDREIKLNKKSIEESNFNPKHPTRITIHGWTNSKDNYINTGIRKAFLSHGDYNMITVDWGRARSVDYVSSVLAVPGVGKKVAELVDFLVKEFDMDLDNLEIIGHSLGAHVAGYTGKNIKTGKAHAIIGLDPALPLFSYSNPAKRLSSTDAHYVESIQTNGGKLGFLKPIGKGAFYPNGGRKQPGCGFDLTGSCSHNRAVTYFIEAVERDDFVSIRCGDYNEAVDKKCGSTYSSTRMGAVTNAYMADGVFYVPVNKEAPFGKLA*

>Woma_00010358

MYLLLTMKCYLLLALFVGSAIAAAIPEDEHIHGENGWYIPRGDGSFEWVDLEEAEYILKASSLMEGRLSFNPVTFYLYTRSNPNEDREIKLNKKSIEESNFNPKHPTRITIHGWTNSKDDYVNTGIRKAFLSHGDYNMITVDWGRARSVDYVSSVLAVPGVGKKVAELVDFLVKEFDMDLDNLEIIGHSLGAHVAGYTGKNIKTGKAHAIIGLDPALPLFSYSNPAKRLSSTDAHYVESIQTNGGKLGFLKPIGKGAFYPNGGRKQPGCGFDLTGSCSHGRAVTYFIEAVERDDFVSIRCGDYNEAVDKKCGSTYSSTRMGAVTNAYMAEGIFYVPVNKEAPFGKLA*

>Woma_00010375

MQKQEKLHRHHQQNFCHPQNFRILFLVGVLSLMATCADADDYLKEAPAFLKVCKLSDSQIGNCFAKAVENIFINWNDEIPGLKSLGSIDPYDMKKCTSSITIQTSILN*

>Woma_00010377

MVQKQEKLHHHQQNFCHPLNFRILFLVGVLCLMATYAQADDYLKEAPPFLKICKLSDSHHGECLAKAVENIFTNWKDGIPGLKSLGSIDPFHVKKVHFEHNNPNLNIKLDVKDVTVTGLSQTKVTKASISDGYNFKILLKLPKMRAKGIYRLKGSILLLHLDGSGPLDSELADAEVAISVKAQLIEAKNQKFFDITHVQSSIKEISNFHIQFDNLFNGNKELEDSANALFNENWRELFELLRPALEETLDTFARERFKAVFSFVPANYFFEDLL*

>Woma_00010371

MLVLKRLILITILCALTNAQDYQDYQENTPRPAPIRLRPSSGQVDAPRPTPVPILKQINKHNEDGSYTYGYEGADGSFKIETKLATGEVKGKYGYVDETGKVRVVEYGANKYGFQPSGEGITVAPPTLVDETRKEEPEYEDEIVARPQRPQRINRPQTQYRPVPPPPPPRPQPQPQYVQYEDEEPEPEPPRRQQYVPQSAPPRPQPQLGPAPPRIQIAGAQRTTDVVYSPVQKPSRPDYSQQQTSEYSQTTSYGEGQSNIRISRPVYAPAPVTPAPSSARAQGFLGPASGGRPHLDPFQFGPSQAAPAPRPAPQSIPVQQPRYQQPQLQSRSSGGGGSSLLDQLARDYALPEGTSQPLHDISFGYY*

>Woma_00010370

MKFLYLLLLTLPLLTHSYPAEAEGEDKTILLDDVTVDSELAGSDKDSQVQKRSGGSADYALHLKQGFLSSLGHASASIASGSSGGSSGGGYGYKSYGDGHHSNSVEYNPWSLKKAVLNTIFQAVKAITGGVTALKGQLIKGSGYALSASGQVVAASGDKVTDVGKYIINSAHSKLQPIPSGGGGGHPFGKFASLSGASSGGSSSGGGGKHPSGPVVHTESITSYEIPSGHSSYGPPSKPPTYSSATHQYLPPSGSGGYTGGGAGYGGGHVAFEAPSSNYLPTAYGPDVSNGEEFNIYGRHQKLSDEEARKAAVQLQEILNMLPNGKTEYTASKTVTLDTSGGYGTGYSTGTGDSVDLSSYGLPNDLGPLESLSHTEQITAAYGTKAPYDLYHQMRKQKQTPEEIYIEKHPNEGYDYKSPSHNQGNNDIYKYNNYHATSGGESNKNSLKDLTPIIAALEVAKQKQPVKAQVSYAIEKNVHKIPAHNGPGQYLPPAVQKNKYIYYQPTPAKYEYHTQYNRPGAYNGPYKVNRRSVDVAAARPKRMAMLYEPQSQSYDEILYNPLMADLQK*

>Woma_00010373

MYKLFIVSCLVIALQQIKAQNQQYTTPVPILKQIDRQNSDGSYTYGYEAADKSFKIETKYPNGEVYGKYGYVDDQGNVREIEYGATKRGFEPAGSHINVPPPTLTASKATPLGPNEIDDGQYREDPAVYYKDQKYSRPVAASKFSLNNNYQQPRPQPAYNPPPPAFNHAPAFNQQPAYRPQAPAPAPAPAPQYYNPPPQPHYQPNYYRQPPAPQPQYNPYNPSSSPAHFHPAVKSLDIWSGSYSLDYTGRK*

>Woma_00010379

MKSFNISYMRFLVVLFWLSLINGSTCFDYIKEQPAFLSPCKIYQPGFTECSTKNTQNLINAVVKGIPEVRAVIGSTDPMKLDKILFRQENTEAASIRADLTNLMATGIKDLVVKESKVSKKDFSWLTKIFLPKFKLEGHYKMDGRILLLPLNGEGHFVIEIDNMDITMRTKTHLVEKGGFTFYNITSIKVDLDISKLHTQFDDLFGGNNKEVERSTNESFNKNWKEFFEALRPLINETVERVLFDMLYPVFLIVPANFMIEDIPSPEKLYGAKQ*

>Woma_00010378

MKVLLKLYAIVAIITVAVTIHTANAAKYLAEKPDYLVPCRLQDPNFEKCFKRSFGVLFHEWKDGIPGLKSVGPIDPLEVKRVVIAQDPKNPVALNADLFNVIVRGTSNAVVQEATFDPKTLKQKFQVSVPKLRFDSDYKLNGRILNLSLNGSGKAFFEVENIVMVFYLQLKLRDEHGFTFTDVDKLQLEIEDVGGFRIRLDNLFNGQEVLEESTNAVFNESWREFYEILKPAIRESVQAIMKDRLTKYYSYVPGNFFIEDLPSAAEHSG*

>Woma_00010357

MKHLWALAFCAVAGATAALIPQDERVNGENGWYVPQADGTFVWIDKEEGESYLEIASKIEGRLSSNPVNFYLYTTLNPYVAQEITASTKSIDASNFNAAHPTRITIHGWTSNKDDYVNWGVRNAWLLHGDYNMIAIDWGRARSIDYASSVLAVPGVGQKIASLIDFLVKNYGLKLNTLEIIGHSLGAHVAGFTGKYITSGEAHAIIGLDPALPLYSYDKPNKRLSSTDAYYVESIQTNAGELGFLKPIGKGAFYPNGGKKQPGCGVDLTGSCAHGRSVTYYVEAVEKDNFATFKCADYQDAVAKSCGSSYSSVRMGAKANAFMVAGDYYVPVKSEAPYGILA*

>Woma_00010356

MKCFLFFTLCAAAAIVSVIPEDEREHGENGWYVPQIDGTFEWVDMDEAEDYLENMQMLEGRLSTNSGRARSVHYASSFLAVSDVGKKVAALVDFSLNNYRLNLDTLEIIGHSLGAHVAGYTGKDIAFGQAHTVVGLDPALLLFSYNKPDKRLSSNDAQYVKSI*

>Woma_00010355

MRSFYLFSCLAVATVLARPEPPVYMGLQQQNQRALPLNAHAAKPHVYQQLPHEHSFANFAAPAPTYGAPLASPSPDTLAPVTSEVAAVADMPQEDSYNVAYRHSSTQHHQTGYDNGPSETQVHKHIYVHVPPKDFEEEDAIQPRITHQGSPKQKHYKIVFIKAPAAPIMRAPIVPPVPQNEEKTLIYVLHKKPEAQPDIVIPTPAPTKPSKPEVYFIKYKTKKDEAPVYGPPPATSDVQQPRQAGEESNADNNDTQQTAFEAEQPLAQPHFLNEEQISAVDNFINEEPQPTAAAEPLNQYLSPETTPAPIVVEDIEEPVHVPAATYGLPNRSRFFIKKRK*

>Woma_00010472

MKLLLTVAVLTVIAGAVNAQGAIQISEEQKQRVVEYATACAEKNSIDKDTVQALKTGKFDNTSQNAKCFTNCFLEKAGLFVNGQVQNAVIIEKLGSIFGADKVNAAISKCNGIKGADNCETAYELYKCYFRTNAALI*

>Woma_00010432

MKFFIVALALVACVYADVSELGYNYEPPAASQPQNSYVPPPPPLPPPAPQNTYIPPAASAPAPAPAPVEPLQNSYIPPAASAPAPSYEAPAQDGYHYKTVRRVVYRYRS*

>Woma_00010433

MKFFIVALALLAVAYAQSGYDYPAPGPAASEPQNTYVPPPPEPQNNYIPPAASESAPAVAEPPQNTYIPPAEPAPAPSHEAPAEDGYRYKTVRRVVYRHRT*

>Woma_00010436

MKFLIVALALVACVYADVSELGYNYQQPIPPFAPAFAPAPVAPVNSNLPPAFAPAPVAPVDSNVPPAPAPVPVAPVDSNVPPAPAPAPVAAINTYVPPATVPAPAAPVDSNVPSALAPEPVAPVNSNVPPAPSPAPAAPVDSNVPPALAPAPVAPVNTYVPPAPAPEPAAPVNTYVPPVPAPAPAAPVNTFMPLAPAPESAAPVNTFMQPVPAPEPAAPEPVAPVIQEPVNTYIPTEQVAPMQPIAPLEEIEQIPEDGYRYKTVRRVVYRYRA*

>Woma_00010434

MKFFIVALTLFAVAYAQSGYDYPAPAPLAPEPQNSYVPPPPPPPPPQPQNTYIPPAASEPAVPQNTYIPPAASVPQNTYIPPAVEAPAQDGYRYKTVRRVVYRYRA*

>Woma_00010435

MKFILIALAFVASASADVSELDYNYQQPAPAPAPVFPPNSYIPPPPLVPIEKPLNTYIPPPAHVGQHQNTYVPPAPVVVEQPQNTYVAPAVNEHVEGLAQDGYRYKTVSRVVYKHRS*

>Woma_00010465

MSIKNLALITICSIILHLSSAEDKCGKCMSNEVYCVNETSYYFCMNGQPLKSVLNTCDEGYVCAADDRICVSKKETGVTPVCSSSCNQCPARARYTCVSQTQYGRCVNNKVAMIGDCEGNSICSLELLDITKSICVPSCVSDFFGGSPTCTNEVATTTTTTTEAPVTPDLENLRQQCKSAGKTNNSKFFYIRNVGDTECKTYIYCEKMGENEYEAVLLRCKTGYFDGTNKCTDEKLDFCDDLASTPQH*

>Woma_00010461

MSIKNLALITICSIILHLSSAEDKCGKCMSNEVYCVNETSYYFCMNGQPLKSVLNTCDEGYVCAADDRICVSKKETGVTPVCSSSCNQCPAGARYTCVSQTQYGRCVNNKVAMIGDCEGNSICSLELLDITKSICVPSCVSDFFGGSPTCTNEVATTTTTTTEAPVTPDLENLRQQCKSAGKTNNSKFFYIRNVGDTECKTYIYCEKMGENEYEAVLLRCKTGYFDGTNKCTNEKLDFCDDLATTPQH*

>Woma_00010424

MSVFYKIFLFILILWCSEAAWRTPLNIELKQAEPCSQEKCQPPNCRCSGMELPSVEFKGREKEIPQFITVTFDDAVNAINYLQYQELFNNLKNPDNCEVKATFYVSHEYTDYTKLNALYNEGHEIALHSITHGAGTEYWRQADVDLIMREFGNQIDILEKFAKINRKHIRGMRLPFLQISGNNSYIAAKRIGLLYDSSWPTQQYRNPAMWPYTLDYLSVQDCQIPPCPTASLPGLWVNPMVTWVDKEGYSCSMLDGCIYLPEDKVESLFAWMKENFHRHYDNNRAPFGMYLHAAWFGRSPNYIKAFRIFLEYANSLPDVYITTPTSVIQYLRHPSLGKPFKGCFKKPQTSCRPTSCNLRKQSTGETRYMTVCDRCPKVYPWLDNPLGEI*

>Woma_00010426

MEYMKSTLCVLLTLTTIMMAFSAPNRKGDRIHFIDSSEADSYDTDVRPGNGNRNRNHRGRGKGHIQVPEFEHGIPGVYPIKGPPQLPFVLPTTVQPGQQFSTINTEKPIVSSTPNLIYQPAKSTINSNQYGNNPFLKNIQTSTKSGPVFTGGITNQLPTSDKPSTTTPTYQNPRRTDLLYGSNLLEEESNRDGKASKTDDIEFDGRGLFDTAPRCQDGQILVNNRCRLQV*

>Woma_00010429

MFQLCYKLVIILLLIYNRQTEANGHINNDGHNCDYKQLQAMMGQLAARILLGKAESTDNPDAAPPTLQSIFTLLPRCKPGFILVGTKCRKQV*

>Woma_00013243

MKNITVILQLFVLLLRSFMPDFFQRGQNDLNLENKDNKVKLLKEYDFIVVGAGTAGCTLAARLSENPSWSVLLLEAGGPENLAMDIPAFAHVLQLIPELNWLYRAQASDKYCLGITNNRCNLPRGKVMGGSSVLNYMIYTRGNRRDYDHWAELGNEGWSFEDVLPYFKKLEGSTVPDADSGFVGRKGPVKISNTDWRSPIASAFVEGLQQDGLKLRNYNGRIQQGVSYLQSTTYKAIRWSSNRAYLYPLKDKRPNIHVLKYAFVTKVLLEPQTKTAYGVIFESQGKSYEVLARKEVILSAGAINTPQLLMLSGIGPAKHLRAIGIKPLQDLAVGYNLQDHFAPFLTFLTNATSIHLTDIFDLNKYMEFSNNTGELGSPGGVEALAFYDLDHPGIEDGWPDIELLFLGGGINANPATVSALGLKPDIYQALYSNIIRKDANTFLIFPIILQPRSRGRIILGSNNPHKHPLIFLNYMADPFDVDIVVRGIIKTIELIEKPAFKKINARLSQNTIPACRKYGSIKTRAYWECYVRHLTLTFYHYSGTAKMGPKSDRSAVVDPRLRVHGIRNLRVTDASIMPKIIAGHLNGPVFMIAEKAADMIKQDHGFIQ*

>Woma_00013245

MFKNIFKISLGLCILALITQAEHKTQITLHVLKEFSNQLDISPYERVGNKLYYFGQSKVTWFKALLICRSMGGFLASFESPDELNELSKYMTTKFPTEYWWWLSGSDLDVEGDFYWYRTGERMGYSAWTTGQPDNHSGQEDCAVLWYQSTKYEMNDFRCDLQAYYVCEADKPKTIVVSIF*

>Woma_00013246

MFNKFLKISLALCLLSAITQAGHKTQIATHVLEEFSDQLNISPFERVGKKLYHFGQSKATWFKAFLICRSMGGFLASFDSQEELSDLSKYMASKYPTDRWWWISGSDLDHEGDFYWYRTGERISYADWSPGQPDNAGGHENCVMLWYQKTKYQMNDWVCNQQAYYVCETDKPKTIVVSVF*

>Woma_00013233

MFKTRSLSCCLLLLLIAVATLTFSHMAEAASRNCPENEKYYDCGNSCQTECATLNKPCLIRHIRCPDGCYCVDGYARDASEIIVNP*

>Woma_00010519

MQCTLIILLLGSCVLLSCALPVTKTKKDESKKEESSTPGSPDVETALEYERYLKEVVEALESDPEFRKKLDKAPEADIRSGKIAQELDYVNHHVRTKLDEIKRRELERLRSLAKKEYELENEIDREHLKINQHIDHANEHTFEIEDLRKLIKKTAEDLAAADRKRQAEFKEYEMQKEFEKEMIKKELDEEQRKKFEEEQNALKEKHKKHEKVHHPGNKAQLEEVWEKQDHMDKEDFNPHTFFMIHDVDGNGFWDETEVKALFVKELDKVYQSGLPEDDMRERAEEMERMREHVFQETDTNRDGLISFQEFLEQTKREEYNRDPEWQTIDEQPQYTHEEYLEFERRRKEEIDRMIAQGLLPPHPNMPQGYYAHDPNAAYQMGQQHQGGYQQQPQMHYQNPQQQHMQQQQQYQQQQQQYQQHMQQQSNPQHQYQGQQVHLNANQVYQVPAQQQQQQQYQNLPQQQHQPPPPQQQQYQPPAQHQHYQQQPPQQQYQQPQLPQEQPIANNSPQQVASNSVSPQQSVNNNIPEKQQPINNANSQQHINVVQQPVVQHQQQNIPSNK*

>Woma_00010500

MDLSVEENIRTLNANLPVEEILETQETGHNIDSSSSADMKSRTLKRYLIQDKTELFKEQINIKKLALDFAASLE*

>Woma_00013187

MKLSLITLTIVILTIVNYSMASKCNLGNVGARIALPTSCSKFLVCSRNLRVVVRECPKGLHFNNDLEMCDLPSRVNCKYGGLQAMSSQCQSCDCNACCNTVTTTPAPVTTTPCEIDTKPTVTPTVPTSTGETNTDTTTPKPVTKPTVTPTVPTTTCETITDTTSPKPVTKPTVTPTITATPSESGKDTTTPKPVTKPTVTPTIPTTTCETTTDTTSQEPVTKPTVKPTIPTSTDETNTDTTSQEPVTKPTVTPTVPTSTGETNTDTTSPKPITKPTVTPTITATPCEIGKDTTTPKPVTKPTVTPTVPTTTCETTTDTTSQEPVTKPTVKPTIPTSTDETNTDTTSQEPVTKPTVTPTVPTSTGETNTDTTSPKPITKPTVTPTITATPCEIGKDTTTPKPVTKPTVTPTVPTTTCETTTDTTSQEPVTKPTVTSTIPTSTDETNADTTSQEPVTKPTVTPTVPTSTGETNTDTTSPKPVTKPTVTLTITATPCEIGKDTTTPKPVTKPTVTPTVPTTTCETTTDTTSQEPETKPTVTPTIPTSTDETNTDTTSQEPVTKPTVTPTVPTSTGETNTDTTSPKPVTKPTVTPTITATPSESGKDTTSQEPVTKPTVTPTVPTSTGETNTDTTSPKPVTKPTVTPTITATPSESGKDTTTQETITKPTTKPTIPPPSTPTDCVGVCCDKPNGIKFSGNTCQQFQVCQDNKVIVFNCPNNLHFNAATGKCDFPEKAKCDKPYHPPSGPHAGPSGTTCENNGRCAGQPDGKTFTDPSNKCSAKYVVCQCECEVERSCTAPLKFNSKLGVCDWPSAFGC*

>Woma_00013189

MKVFIAFCLLGLIAVTRCQETTTAANDEDLSTIDSNNERGAVIITDAAVTTTDAAVATTDATNGKVTITTTTLSTKKLHSRPFYIKRHYGRHSFSSGKVKTTKTRPAQKPNSRGRKGNGNGSRRNGKSGNRGGGRGSGRGGSRGGGRGGGHGARRGRGGGRGRNNNSKRGKGVSVNRKGDYNLKVAGKSNK*

>Woma_00013188

MFKVRFALLLASVSISLTYALDICENQLNGVRLAHPADCNKFVVCKDGQTQSIRNCPIGLHFNRFKGVCDLKERAACDFEFRQFVDNELDCSTCCAVCGCDGFTPTPENPGTPTPENPGTPTPENPGTPTPENPGTPTPENPGTPTPENPGTPTPENPGTPTPENPGTPTPENPGTPTPENPGTPTPENPGTPTPENPGTPTPENPGTPTPENPGTPTPENPGTPTPETPGTPGSSCLNDSTICVDKPDGSVSYVNGTCRQYVVCIGGCGNILSCPNNLQYNLTTHQCDYPQNVDCPWESQPPITGPSGVTCAQHGECLGKPDGTMLPDPNSDGYIVCQCECEIERRCAAGTKYNATLEACVHINNDSGNDNGNGNGYGNNKNAICTNNDIKDCQDQTDGTLLPVNGECNKFYTCVNGCAVVNICPNNLIYNPTKDYCDYPQNYKCPWPYTPPSGPTAGPSGIACASGGRCIGKPDGTFFPSNTNQSKYVICQCECEVEMNCPVGLIWNMQQLACNWPPNLE*

>Woma_00013190

MFRVILLLLLLGGSLALSNRLDICEDQEDGARLAHPTDCSKFIECENGETVAIRYCPHGLHFNRYEAICDYKFRAGCDYSYSLQLLSNDDAGDFDCAVCRAKCSNYGGNSPQRPPPLEQPDEKPKPPTAEKPQQRPSEPCETSPEKPSTPSTPKPTVTPCVTKPSTPVKPVEPTTPCNMPTAPTLSTPKPTVTPCVTKPTTPVSPVEPSAPCNTPTAPTPSTPKPIVTPCVTKPSTPVKPVEPTTPCNMPTAPTPSTPKPTVTPCVTKPPTPVKPVKPTTPCNMPTAPTLSTPKPIVTPCVTKPSTPVSPVEPSTPCHTPTASTPPPSSKPTTAPCSTKPSTKPTTTPCSTKPSTKPTTIPTKTPCASKSSISSKTYNGRSGATGQSLQEAMNKPASVRLCENPGQCADQKDGTSFKDPLTNGFIVCHNQCEVKMACPTGLVFNPTIKVCDWPKA*

>Woma_00013199

MFKKVFYQLFAFLVVIFRLTRGEVEADDVASSSESTVAPRNYFDPYECYPCVYFDSPYSYCDYHLGKK*

>Woma_00010641

MKSFAVCFMVLTVIVAMASALPQQREGAAYTNEAIRQAQQTFLIPKDAQIQNVQEGIELGAYEQIPGNQRINLFEILGDQVPSEVINNLQAQVDQIGRN*

>Woma_00010575

MHHFKLTCFMAILSFLVSTESIILNQIDLQNFLRSGALDLNVLRCFLRNTNECPNPYIMHRLYTPKSSRRGIALNIHNPLTLYQGGFSKHRDTIFIVHGFNGTAIDRHLQFLRDAYLSRDCNVITVDWNPLTQYPCYLSALINTRLTAQCTAQIYSFLTHHGADREKITCVGHSLGAHICGMVSNHLTIKQHKIIGLDPARPLIERKKPDRFRLSPDDATNIQVLHTNAGVLGLEDNTGHLNFCINGGRQQPYCRGNPIRRARCSHFLSICYLASATFKHKKYLGLPCPNGCVDISGPKRLPITKNNPFELVAQIKEYYMGNDAPETARGCYCLDVPYVKHCPFTDIT*

>Woma_00010574

MLKMLILLLIIYLNYCSSVTAQRHIWNREDLNLFVQRHINIEPIYCQLLRTDLCPHKFLTFHLYTRQV*

>Woma_00010573

MFLFKNFCLQHVLLSFLLINICIHGINSQFIDEDEDDSNEDETEEEKAERKSIEEDSYLSVNKLITKYGYPAEVHTVTTEDGYILTMHRIRGKPGAQPFFLQHGLVDSSAGYVIMGPNISLAYLLADKNYDVWMGNARGNRYSRNHTTLDPDGKKFWEFSWHEIGIYDLPAMIDYVLKTTGFKKLQYAGHSQGCTAFFVMCSMKPEYNKKIIMMQAMAPAVYARETEEHPYIRAINLYFNSLVGSSITEMFNGEFRFLCRMTEETERLCIEAVFAIVGRNWGEFNRKMFPVILGHYPAGVAAKQVKHFIQIIKTGKFAPYSYSSNKNMLLYKEREPPRYNLSAVTVPTYVYYSTNDLLCHPGDVEDMYKDLGRPIGKYLVPQKEFNHMDFLWALNVRKLVYSRMLQVLGKVNKERRDNVSAYPSTQVPNTAKELSL*

>Woma_00010588

MRKLLFILIVLITIFQVSQGFWPFSRKKTPPPREEKRLSPVIFVPGDGGSQVEAKLNKSSSPFVWCPKQWKWYNLWLNLELIVIPAVYCWVDNVKLYYDNITRTTHNTPGVDIRIPGWGDPEVVEWIDPTHNKAGAYFKDIANMLVNMGYERRKNLHGAPYDFRKAPNEHKQFFINLKKLVEDTYEANYQTPVTFISHSMGSPMTLVFLQQQDFEWKQKYVARQISLAGAWAGSVKALKVFAMGDDLDSFFLSGKILKEEQITNPSTAWLLPSPLFWKSSEVLVETPNGAYTMAQMQNFFNDIGYPTAWEMRKDTLHYTLNFSPPGVELHCIYGDGLDTVERLRYKKDDVSDDTPKIIMGLGDGTVNRRSLKACHHWTGYQASNISTLALSGVDHMGILANKDVINYIQNVMKL*

>Woma_00010561

MNWFVLILILKLLTTTWSAPIDIEETKSDLAKFIKDFTVTHNAESLLQPAENTYSTQNESLKDEFQKVNDEVKNVSTKVQQEFEEKPAEAITEQTTEKNAEAIVQIFKSVESVTENLTENPTVAELNSNNENTSNISEQTSQSSVALNELPENVHLEAVTESNSDFKTESSTEIPLTGLTENEESKQEFVLSTTGFSREVQNVFATATDQAAKHYAPDSSEITTKDPTVNDFIISNNSDVSSNGSVNIGTTAAISKETSSASSKLGTNTATEINNNLAVRASEEVTVQIYPSTLIFEHSTLDNQEESTHTSAEHDTSSKSYSEIFANEPTPAQINPSTAAANENFSHKPTEEDFKQSIDTFAPQLGKTTAGLVNQAEESVTTISRNQEPDETIGESTEFSPNYPHISNDNFVEATEPIDTKPKHNPSFKEAVDSFIADVMGIGSSLGLLNKTEKTEHVQPTSESSTNDDQQSSSPVAKATESQSYLPESLEDRNAKNTDIFSSTQENTSNATPITGNSSETSSDSSNLGNTLEHLVGSASEFVGTNIGKTVVNILSDNENINEKEHEDVTEQQTDAKSHFNAESPNYSSSTVTENSRELSTAVPYGNMASAANTDGNFFEVNQEVEGSSAKKELILTTQISEEAENISHIPENIENTSSNQELTSTSESLHEVQNVENKPELSAADQSYLESENKSSNQELTSTSQSLYEVQNVENKPELSAAGQSYLENENTSFNPELTSTSQSIHQVQNVANKPALIPADQSYLESENSSSSQELTSTGQSIQKVQNIANEPELSTTDQSYLESTNTSSNMELTSTSESLQEVQNLENKPELSAAHQSYLESANTSSNLEFTSTSESLQEVQNLENKPELSAADQSYLESESTSSNQELTSTSQSQQEAKNFANERELSAADQSYLENENTFSNQELTSTSGSLQEVQNVENKPELSAADQSYLESENTSSNQELTAASQNISTNPTPREVDNIFINQQLMATNQEGRDVESFAKRPALFSTHHDSQSNLDLRNSKSEILEHPVHYEEYTTVTIATGLEEEESSTKENHVQNIESRKLEDDPTNASLYPNTEEVLGQTQHNLELKNPESSTLAQSAHYEEFIPVAIVTSPEEEEGRRANFNLFENTQPEVSETSTHFSQQTEESFQSHQNYPVTENPTSAAQTESTETNKKDDSSATVFTSMEHNENSKPDIVSKDSTNTEALAEEASQNTYNFKEETSNAFNKFKNTLKSIIENETLKKEEIHATNLELSQIETTTSTPAQHSSIIKTSLAADDETTVLPKKFEEELQMAVKALEQTTHKQFEEFQNNLKEAFKKFTKFGVAEHEKSEEESTALKITTESAETVEKTTFTKAVEETTAATINILLEDSVKITEPRSGAIHQQFVDVFDENPSTTQIPKETEEDLEDKRSITELPVIKHEAVILGNNVENATAEEQTTEISIGLTTLLPAAERILDTQNQESEETTKPTDIKEDLSVGGEESTTKPKESSTEFIDETTFSSSAPLIAVSLQENASHDGHNELKETNSEVAVTTTVHQQQQLNLQQINKTAEQESVKEDHTEVTYADQPMATVEEDMITTPSSETITPQVMVKFIEHTETTTEHILQQQQQQYNQHDNTLPTSQTQLPLTQASFMEEQQQQIESTTANLEQTAITSDKQNESLIQTTTTTTVEDTTNGQFNTTTTINGLQEEGEATTTAMPELESASVEAEARSLSLPPPKLEYLQNEDGVEVFYGYSIVRRN*

>Woma_00010562

MKFFALLCIVVLAIVSAVSGAEGAGAGISESASGSHDVDHAAGNVAHTAAGAAKNVARGGLKPKDLIALSG*

>Woma_00010564

MKFFVLLCIVVLAIVSAVNGAESAGADISGSASGSHDVDHAADNVAHTAAGAAKNVARGSPKARRIAAASG*

>Woma_00010565

MKFFALLCIVVLAIASAVRGVESAGASISGSVSGSHGGGHAAGDVAHTAAGAANTAAGVAADIVAADVGLKVAAANRLASIAG*

>Woma_00010566

MKSFALLCIAAIVIASASAGGYGGGGGGGHGGASAGASAQASASSGAFGGGIGGGLGGGHGGGRGHGGGRGGGHGGGHGGGLGGGIGGGAGIGGGSAGAHGGSLGGGVAGGNGGGIGGGLAGGHGGGIGGGLTGGHGGGLGGGSAGSHGGAIGGGSAGGHGGGIGGGHGGGIGGGHGGGIGGGSAGGHGGAIGGGSAGGHGGGIGGGHGGGIGGGHGGGIGGGSAGGHGGAIGGGSAGGHGGGIGGGLGGAIGGGSAGAHGGSLGGGLGGGLGGGSAGGYGGSLGGGVGGGHGGAIGGGLAGGHGGGIGGGLAGGHGGGIGGGLAGGHGGGLGGGLAGGHGGSLGGGFGGGYGGSSASASANAKAAAGAFGGGLASAKASANAVASSYGR*

>Woma_00010567

MKLFVFLAVLAVCAHTQAQRVGYGGANAAASANAVAGGFGGALGGATASAASKSSAGGFGGFGGLGGASAGASANANAAGVAGGLGGGFGGHHGGHHGGLGAGLGGGIGGASAGANANAAGVGGGLGGGLGGGLGGHHGGHHGGLGAGLGGGIGGASAGANANAAGVGGGLGGGLGGGLGGHHGGHHGGLGGGLGTGLGGGIGGASAGANANAAGVAGGLGGGFGGGHGFGGGLGGRGVLSGLLGGLANAAANANAAANAAANAAANAAAGGFGGGFGGGLGGHHGGHHGGLGAGLGGGIGGANAGANANAAGAAGGLGGGLGGHHGGLGLGGGFGSANANAAAGGVGGGLGGGLGGGFGGATAGANANAAGGAGGLGGATAGAAANAAGGAGGLGGATAGAAANAAGGAGGLGGLGGATAGAAANANAVGGAGGLGGATAGAAANANAVSGAGGLGGATAGATANANAKTAVVG*

>Woma_00010569

MKVFIVLAMLLAAVCAVPQFGFGSPYGGGYGRPGVGAHAYGGGFGHPGFGGPSYGRPAYGASPYYGGGGLGRPSFGHPGSFGGGAGGGSASASASASSSASASASAAGGGLGGAGAASASASASSSSSGGGFGGFRG*

>Woma_00010760

MLKLFIVVISLFLLTEATLVSVPLEKVKVQKSKANELMKLRAKYNVKDSCTIHKEKLFNYVDVSYYGKITIGTPPQEFLVLFDTGSSNLWVPVAPCNSTNLACQNHNKYYPKNSTTYEYNGESFAIYYGSGSLSGYLVQDTVTIQGMAIQNQVFAAATNEPGFTFVYEHFDGIMGMAFQEISVDNVVPPFYNLVSQNLLDSQVFSFYLARNGTSKDGGVMVLGGNDPNHYQGEFHYVPVSKKGYWQFEMRSAHIKDTSVCHYCKAIADTGTSLIAVPRDQYENIQNAIGATFNATTYEYLLDCATIDSLPDVSFHIGDGFFTLEPKDYVIESEGQCTTAFEDSGMNLWILGDVFIGKYYTTFDLRNKRVGFALAV*

>Woma_00010761

MLKFVVTLLSLALLSEAAIVVPLHKVKEPKSAANELYKLKSKYSDLIKIKARDGVQESLMNYVDDSYYGKITIGTPAQEFSVLFDTGSSNLWVPVAPCASGDSACENHNTYDPSASSTYVANGEQFSIAYGTGSLSGYLVQDTVTVEGLAIKNQVFAAATNEPGTTFTYSPFDGILGMGYQTIAQDNVVPPFYNMYSQGLVDSNLFSFYLARHGTSNEGGVLVLGGVDPSHYTGEITYVPISSEGYWQFEMNSAEINGVNVCDSCQAIADTGTSLIAVPNSQYENIQNAIGATFNYDVYSYTVDCSTVDSLPPLTLNIGGTTFTIEASDYILKSDGICSSAFQNIGTDFWILGDIFLGRYYSIYDLGNNRVGFATAV*

>Woma_00010766

MLKFVVTLLSLALLSEAAIVVPLHKVKEPKSAANELYKLKSKYSDLIKIQARDGVQESLMNYVDDSYYGKITIGTPAQEFSVLFDTGSSNLWVPVAPCASGDSACENHNTYDPSASSTYVANGEQFSIAYGTGSLSGYLVQDTVTVEGLAIKNQVFAAATNEPGTTFTYAPFDGILGMGYQTIAQDNVVPPFYNMYSQGLVDSNLFSFYLARHGTSNEGGVLVLGGVDPSHYTGEITYVPISSEGYWQFEMNSAEINGVNVCDSCQAIADTGTSLIAVPNSQYENILNAIGATFNYDVYSYTVDCSTVDSLPPLTLNIGGTTFTIEASDYILQSDGICSSAFQSIGTDFWILGDIFLGRYYSIFDLGNNRVGFATAV*

>Woma_00010767

MFKFVAVLAVLAALASAELVHVPIRKHDITIEGLTFKKEVFAEAMNEPGNSFTDAYFDGIFGMAYQSLAEDNVVPPFYIMFAQGLVNADMFSFYLKREVSSGNINGQIICDNCQAIANTGTSLIVCPEAAYNTLNSEIRATFNILEGSPDSLPVVSFVIGGKTFTLEPSDYIVEIDVKCMSSFTTMGTDLWILGYVFIGRYYTVFDLGNNRVDFAPVA*

>Woma_00010764

MLKFVVTLLSLALLSEAAIVVPLHKVKEPKSAANELYKLKSKYSDLIKIQARDGVQESLMNYVDDSYYGKITIGTPAQEFSVLFDTGSSNLWVPVAPCASGDSACENHNTYDPSASSTYVANGEQFSIAYGTGSLSGYLVQDTVTVEGLAIQNQVFAAATNEPGTTFTYSPFDGILGMGYQTIAQDNVVPPFYNMYSQGLVDSNLFSFYLARHGTSNEGGVLVLGGVDPSHYTGEITYVPISSEGYWQFEMNSAEINGVNVCDSCQAIADTGTSLIAVPNSQYENIQNAIGATFNYDVYSYTVDCSTVDSLPPLTLNIGGTTFTIEASDYILKSDGICSSAFQNIGTDFWILGDIFLGRYYSIYDLGNNRVGFATAV*

>Woma_00010768

MKVACGIFAIILLVLSNSITSGYVVEPNNYSNKDISDTVQAWNLLFDAIYKGYGLEKHFPQPGNVPFNSKFFQCYLDYKLQNIEALAQTGRDIWQCVNVSIWELQIQFRRHSWRRLKQHTTHEITRCKTKHDDEKKYMHCYAEVIFESFKYLDILSENSKEDSEHCIQSALQNLSDTLQEINKELQNCLLESLNPPITTVEPPVNSTLNPWQNTTLSPWPNTTWNPWTNTTAVTSTVNTTWQPWPNTTWQPWPNTTWQPWPNTTWQPWPNTTWQPWPNTTWQPWPNTTWYPWANTTWQPWLNTTWNPWPYTTWQPWPNSTWQPWPNTTALPPTVNTTWQPWPNPTWQPWLNTTWNPWTNTTWQPWPNTTWQPWPNTTWNPWPNTTAVPPTVNTTWQPWANTTWNPWPNNTWSPRPNSTWQPWPNITWNPWPNTTWQPWPNTTWQPWPNTTAVPTTVNTTWQPWPNSTWNPWPNTTWNPWPNTTWPNTTWNPLPNSTWLPWPNTTWNPWQNTTLSLPQNVTITTSLYNTTWAALQNTTDNSAIVATTVATEAPTTSSLHLSWWKKISGYVEDPKDQRKRNIDNTFQAWSLLFDAIFEGYVFEKEFVQPQNLPLDSEFFKCFLDFKLRNIQILSQGGRDIRLCVDVSTWELEYKLRRYSAKRLKKLSTQEIIYCKTKYYSDNHYLDCYADVIIDCFKYLDIRSRKLKMHRKSCIQSALLNLFKLSQEINEELQNCLSESVDQSESSTQVITVPATTKTTVPTTTQTTVTSSTTVKVTNQTTGIQTNQTTVTQSNQTIIIQLNTTSNTGPNIIVNTYPNSIWNIYQNSTFNNWSYTIYNLYSNVTWNPWQNITWHPWQSTTLSPQENVTTTTPSFNTTLVPIQNTTLSPPQILTTVTPPFETTRKPIQNTTLSPPQMVTTVTPPFETTKKPIQNSTLSPEEIITTVTPSFNTTLVPIQNTTLSPPQIVTTVTSPFETTRDPILNTTLSPEEIDTTATPPFETTWDPIQNTTLSPEEIDTTATPAFETTWDPIQNTTLSPEEIITTVTPPFDTTSDPIQNTTLSPEEIDTSPTPFFETTWEPIQNTTLSPEEILTTVTPPFETTWDPIQYTTLSPQEILTTVTPSFDTTWDPIQSTTLSPQEILTTVTPPFDTTWDPIQSTTLSPEEIDTFATPPLETTWEPIHDTTLSQEEILTTVTPPFDTTWDPIQNTTLSPEEIDTSATPPLETTWEPIHDTTLSPQTPLTTVIPSFHTTLRPIQNITSKPITTRFPPVWAVSKAPSVRSPSWWQKLWSKITSFFFFVIKCIRCK*

>Woma_00010774

MLKLQACLALLLCVAVTLGEAKRVKRIVGGHHAKAPPPDDPVVFTRTYNRDARVEGFRNSITKIYTFLGLYYAEPPTGQNRYARPVYKRLMGDINATLYGPPCIQPDPQNHNRIIGDENCLLLNVYTPRMPDETTGLPVIVWIHPGGFRYGSANQYDATPLAQQGVIVVAPQYRLGSLGIIGDGTKEFDGNLAIFDMAAALRWTNDYISHFGGDPKKIKIVGHGSGAASAMYLSMSRSARSAGDITGVVAMSGTALSQYATDKEPVQSVQEVAEINGCPSTNEIEIVKCLRKKTAKEIIENDSKIQTERLAGRAMIKGLSGSIGFQPHIESEDDGRALPSLIVGEPDQQLKSGNFTAIPLLTGVTKHETANAFTLENINKVYGSAEKFLGSLTDALKDLTSFLRVDQITGEVTKASLPGLSSALTPTLNEVLKVPETLNLNQILSKVVEATTDVLFNLPAVLTTQVWSQIAPAFMYSFEYNGTTSKGINFLRGLPIVADTKNSNSDIVAHGDELGYMFDCNDVFGNPLPDTRLTSEQDLKVRKNLISMIVKFATDFSENTKIGKFSDNLFKSVTGKGTPFIKVDTDLSTDSDFRFCELSLFGASLSPVTSTTCEGLGSVLSTLQGTLGGLTKSLGGGTNLRGNNLGGILSGSRGTSTRGNNGGGGSVGGTVSGGVQDLFGNVQLTGGNNTRRTGGGLFGLL*

>Woma_00010763

MLKFVVTLLSLALLSEAAIVVPLHKVKEPKSAANELYKLKSKYSDLIKIQARDGVQESLMNYVDDSYYGKITIGTPAQEFSVLFDTGSSNLWVPVAPCASGDSACENHNTYDPSASSTYVANGEQFSIAYGTGSLSGYLVQDTVTVEGLAIKNQVFAAATNEPGTTFTYAPFDGILGMGYQTIAQDNVVPPFYNMYSQGLVDSNLFSFYLARHGTSNEGGVLVLGGVDPSHYTGEITYVPISSEGYWQFEMNSAEINGVNVCDSCQAIADTGTSLIAVPNSQYENILNAIGATFNYDVYSYTVDCSTVDSLPPLTLNIGGTTFTIEASDYILQSDGICSSAFQSIGTDFWILGDIFLGRYYSIFDLGNNRVGFATAV*

>Woma_00010771

MKSLIVLITCLTLIHLSDSQVLSAGSCNPKIPTVKNFDASRYLGRWYENQKYPFIFELGGKCIYAKYGLMKDGDISVYNFNINQLTGKPNDIKGSAKIVENAKLKVQFSNIPAFIGPADYWILDTDYDNYAVVYSCTDIGGILNGKVVWILTRERHPNPQYIERARSIIKQNGLSLGPLHKTDQTGCEKAPSY*

>Woma_00010775

MQIFILIFSSYNIINAEPSTPKSGLINSLECASSIPCTNTTASHIATITTALPTTPSTPPTSPKPICPNCPNPDCSMVTDGTTFPYANHCRLFYMCIGGSAQVCTCPSGEWYDRITLKCAKCELVTNCIANID*

>Woma_00010755

MKMFCHLLLLFTLIIWSFAADTELEKLFKDTKIIPDVLKEAPKEKLKLEFENGLSVGDGKEYTPTQTKDAPKIDWDSSEPDSYYTIYMASPDAPDAANPKWGEFLHWLVVNVPGKDVDKGEVYCEYIGPLSPKQGGIMRYVFLVYKQPDKMAFDEPKTNNSRLEGHTNFKVEKFAEKYKLGGPVAGNIYRSQWDEYVPILHRNMGVTVDE*

>Woma_00010680

MLKLFIAVISLFLLTEATLISVPLEKVQVQKSKANELVKLRAKYNVNDSNGVHKEKLFNYVDDSYYGKITIGTPPQEFLVLFDTGSSNLWVPIAPCNSGNLACENHNQYHADNSTTYKYNGESFAIYYGSGSLSGYLVQDTVTIQGMAIQNQVFAAATNEPGYTFYYEPFDGLMGMAFKEISVDNVTPPFYNLVSQKLLDSQVFSFYLARHGTSNNGGVMVLGGNDPNHYKGEFHYVPVSIKGYWQFEMKSAIIKDTSVCQNCQAIADTGTSLIAVPYDQYENIQNAIGATFDLDTYEYLLDCSTIDSLPNVSFQIGDGIFTLEPKDYVIESDGQCTTAFEDSGMNLWILGDVFIGKYYTTFDLKNNRVGFALAV*

>Woma_00010724

MYNMQSLILASVLVLMLTVQQGSAIKCFVCNSHKDANCALDMPPDNLLKDCQEDYSSRGRGFPTYCRKITQIIEFSVNNLPPDSRVIRTCGFQNQTSNNFCYQRAGFGGRQVVCSCDTDNCNAATADFISGFSQANFYPKEILKQIKKPKSTN*

>Woma_00010700

MPITPFIRSPFTDLTGSLINHQTYDKCGELEMNMMDCLEAYGAERGKSMCKDLVDDFNECFTMQKQMLRFQAMRSERMKQYWNGQRKKEELFAEPPRVDAY*

>Woma_00010742

MKRLLLLVCCLALLSVGESSTFAARYDDFKVYRVNIKTAQEFDEFKMLGNHLPLRYLTELKGPGFSHDIVIDPAHQDQLEKHLKFLELDSECIIEDLQNVIETAQTRRFNKRNFEMDWEQYHDLTVIYNWLQKITIQNPSIVKPYTIGYSYEKRPIKAVKISAKPGNKAIFVESNIHAIEWISSATTTCFIDNLLSSDDEKMQNLLLNYDWIYVPVLNPDGFEYSHHVERLWRKNRRPTGFHNASGPCYGVDMNRNFGYEWGESGYNIHVPCDHWYGGSEPDSEPEIIALQRFVNRFPEDYIRMYLAFHSFGNFLLLPYGHTSTEFPPNYNEMMEIANAFADGARVKYGTEFKSGASGLLNYLVSGSAKDWAYGVKNIPFTATVELRDNGKYGFFLPPEQIKEVCAEVIDGMIAMVNRAKEKGLFD*

>Woma_00010743

MLKNVLLKPFIVVALLAHLVASAVLPTEKQQEQEEQPIRYDNYKVYKVTIENAKQFDQLMDLESELKLDFWSEVHNYGETCDVMVKPELQNKFEQIMRKFNIKNEVKIQNVQSLIDAEQPKKRSSHREWITSATITYIIDELLVPRNPGVRKIAESVDWYIIPVLNVDGFVYSHETNRLWRKSRLPSDPTGKCIGTDMNRNFDFLWMLTGASSDPCSETYAGPYAESDPEIKQMLQFINNTIPEDTIKIYISLHSYGQYGYAEALYRRYNTVFTYGSSATTLYKVSGSEMILPVAKEVLDGFVGMINAAKEINVI*

>Woma_00010694

MSASPTARQAMNVPQKTKKITISDPMQMPEIYSSTPGGTLYSTTPGGTKLIYERAFLKNLRESPLSQSPPANIPSCLMRGTPRTPFRKCVPPPSDLSKKVDSLKIEEQEQFQLEL*

>Woma_00010714

MNLQDMARFTKHFPVLLLFSLLDLSSEHDTQYLTDSTDPVLSYLKKRSTHKENNANYENQEQQVDAPRTTSVIYNQNDSQQYNNSDTATADLIAEKNIRNTFSFVPFLHVFENISHLIPYTGQMIADQPDPFSSLPTISSFTDKAKMETVATLQAMPYTVDTNDCVKNDEKSGKPFCTKLRSYPEKSHLDKIIETKFSNLETFFGEDLLPPQNINQRIDNKPIEETLCRSRTRVIYPEAGLNKNYDWMFIINTKKYKQGIRIEECINEGSTCGENIGLTLPNRYKATCKQYYNYRSLVAFANTTIIKDQFKMPSCCKCVLTVF*

>Woma_00011356

MSPIIRFLILILAIFGGMLYKKINDIFGDVNAPTLPLNQWWGDEAEPTNWQAYLANSSEVIGNKLLYPDQTIDDLKTQLNRTLRLTEPLEGVAFEYGFNVKQLQEIIEYWRDDYLPRWREREVFLWQFNHFTTDIQGLRMHFLHLMVYDEYTVDRHHYPVLLLHGWPGSVREFYSLIHKLHQTKKDKNNKYIFNVIVPSLPGYAWSQGTSKKGLGPAQMAVIMRNLMIRLGYKKFFIQGGDWGSVIGSHIATLFPDNVLGYHSNLCTSITPKAMLKGFIASLAPSLFAPKGYEDFFFPLSEKYKYILEESGYMHLQATKPDTIGTTLQDNPVGLAAYILEKYSTWTNPSYRSSTDGGLRKRFKLDALLDNIMIYHLTNSITTSQRIYKEHYAPEQRALQLERVPTRVLTGCARFKHDLMHFLDYQLKDKYPNLIHSTYHNEGGHFAAMELPTTLYEDFISFVKKVEKIKKISNYKREL*

>Woma_00011357

MGVLVRASIVALCLALAALIQNYRLLKQPLPAPKFDFQEYWGPGKAADYKENIAVTPFDIATKPEIIEDLKAQLQRPLKLHDPLEGVGFEYGFNSKYLQTVVKYWRDDYLSKWSERESFLKKYPHFQTQIQGLKIHFIHVKPKKTEGKKVLPLILLHGWPGSVREFYDIIPLLTTPNEKSAYVFEVIAPSLPGYGWSQGASKINFGPAQMSIVLRNLMLRLGHEKFLVQGGDWGSIIGSNMATLFPDNVMGYHSNMCGNMGPVSILKMILANFMPSLFYEKQHSGFFKSLYEFFANTMEEMGYFHIQATKPDTIGTALAHNPVGLAVYILEKFSTWTNGDYKHLPDGGLSKRYTMDALIDNVMIYYITNSITTSQRLYAEQFNKAQMSLQMDRVAVKVPTACARFLHDLWHATDCQLKDKYLNLVQSTYHEEGGHFAAMEVPKVLYIDFIEFVKKINL*

>Woma_00011351

MFNVKSNMKLILVIAGVLAILAIAQSRPQEAENENQGEAIEIPSEPEQEQPDQEEEGEIENPEVPHEEPHSRPRPRPHLKPLRPHFRPNGELQPGQQQPGRNEGQNPSRGPHSRQPPRPLRRPHFGQRPQPQPQPQPEQEPNQDDNENPEAVPVLLDGNGFLILNDSGNDNTIINEGADNDDEEENNQ*

>Woma_00011338

MKLFLLSLLAIGSVQSLTIGYPYRSVWTYNPGVAQLPVHGYLRHLDGSAEEPVYAVHQIKYFTSDSPVQKSSDTTKFDEINFCSWSLLQYQNLKRDIEAVRAGGRDPSTEILQKFQTLEKIIIAPNFSAIADLPEVKDIIQDYIHAISLDEAVQSSNIKQNQGEIKTYTGISNIPLVATREVRGAKQAHEDHLRIHAHIEQLQSELHKNITEAFEYVRHQEYAKALAGLERAKEEHFKLWNEAKLRDEKAG*

>Woma_00011339

MKLFLLSLLAIGSVQSLTIGYPYRSVWTYNPGVAQLPVHGYLRHLDGSAEEPVYAVHQIKYFTSDSPVQKSSDTTKFDEINFCSWSLLQYQNLKRDIEAVRAGGRDPSTEILQKFQTLEKIIIAPNFSAIADLPEVKDIIQDYIHAISLDEAVQSSNIKQNQGEIKTYTGISNIPLVATREVRGAKQAHEDHLRIHAHIEQLQSELHKNITEAFEYVRHQEYAKALAGLERAKEEHFKLWNEAKLRDEKAG*

>Woma_00011336

MKLFLLSLLAIGSVQSLTIGYPYRSVWTYNPGVAQLPVHGYLRHLDGSAEKPVYAVRQIKYFTSDSPVQKSSATTKFDEINFCSWSLLQYQNLKRDIDAVRAGGRDPSTEILQKLQTLEKIVIAPNFSAIADLPEVKDIIQDYVQAISLDEAVQSSNIKQNQGEIKTYTGISNIPLVAIGEVTGAKQAHEEHLRIHSEQIAHIKQLESELQKSITEAFENVRQDEYAKALAELERAKEEHFRLWNEAKLRTEQAGYNSAGKTVYDSPEKQQVSAVTSNQNTFNSEQNQEIQTAGQTESLKTTTQQKSFRSHNTPVDQTVVQPQQVVIRDEHSRLVDSSGSQTLKSNIHADRVVETPKVAHANEEHLHIANEAVSKSQAVHQEEEKQRQQRVEQGHQNVYEGQVITPNKDQQGSLNSPHNVEQQNLKSVLPVTSYGQQLHLSATSEVAKAREENLRLVQEANVRAQFVEQQESKYKHQSQTPILTTQYPHEQLVYQPEVVKAREQHLHLVNEIKPKTNKEFQLQEKQRSMVSPGIEETPEVKRAREEHLRVYNAIKSQVENEQTIKDERRTPDSSITTETDKYEAEEKRFLELERLREAERLLKEQQLLELERIREAKRLAEEKLEMEAELERLRMEDEQRLEMERLLEMKRLQQEQEELEKLKTDDYQEIVIQENSAYQHPQQIVYISNEQSIAKSNIPKVNVPLSQEYVYNPYLETSLVGNEPVANRLKHSVNYQQEHSTFSHNPFLKTSAVNVPDATHHQHTVSGHVNKQSSDNIVPSIRYVQSSQQSSGSKHQYIKDDHYVEDKYHNTASSQTAGTADAYERITKDHFRAYEIALDRYHPAKIHNPQYKVCNGCYR*

>Woma_00011335

MWIVLLSALVISSVQALVIINPYRSLVFYNTAQSPLRNQFVQRYLLFMNTNYNGRFITYSLPQPVYVIRVNNYSGSSAVDMQDKGINEFKKAQEAHFRAWSLQQYQALKLEIDVLRAEGKEPSVEMLLRFIPLQKIALTANYSTIAELPEIKQIRQEHFNIWKNVRISDLQYLSENESNAQEQLNTYNGKLDELLKLFENGKLEFDKLEKINADIKTLEEINTSNAKIDAMQKSFHRRSENVKNSEKDNHIGRPYLPHNKVKFEQLNNTEASEDFKVFKTDIITDIPKPEIIFVNRSKNSDDATDKPELLFVAGVECELGTTEIIKEEDLTTERQEIDTTTKKDNDENQEQTTMNQQTTEVTTQEVFETTEVQTIQQDHFLSIIDPKLKKDAKNKIDKLISDAPQIYVPQQFTDTREVERVRDEHLSIVTQAILKSKLTKRSKTQ*

>Woma_00011350

MFNVKSNMKLILVIAGVLAILAIAQSRPQEAENENQGEAIEIPSEPEQEQPDQEEEGEIENPEVPHEEPHSRPRPRPHLKPLRPHFRPNGELQPGQQQPGRNEGQNPSRGPHSRQPPRPLRRPHFGQRPQPQPQPQPEQEPNQDDNENPEAVPVLLDGNGFLILNDSGNDNTIINEGADNDDEEENNQ*

>Woma_00011352

MKLILVIAGVLAILALAQARPQEAENENQGEAIEIPSEPEQQQQNQEEEGEIENPEVPHEEPHGRPHRRPHLKPQRPHFYPNGELQPGQQQPGQNEGQNPSREPYGRQPPRPHRRPHFGKRPEPQPQPQPEQEPNQDDNENPEAVPVLLDGNRFSILNDSGNDNTIINQGVDNDDEEENNQ*

>Woma_00011368

MLNCIYINIYSLSNKKFISLAATMSIHAEGSCQKGAGNDYNVNLWQSTKGLSLPKRHTVCDHKFGIRYFEYTFSLV*

>Woma_00011364

MQYFNYALQTSSRFLTSTTTATTSSSSTDRLNPLKVNQTKQESQNLAYSSSPEQSKLRGNSTATEDILNANIELPIQASASDLDPTPNP*

>Woma_00011361

MTIKWKVVILIVCLLFVFNGIQEGLARTIESISDNHQFDQIMNDHTVENPVRIKSVSVWTLLWQKAQLSRL*

>Woma_00011360

MKFFIIFLAAIAIATIVKAQEKTNPDSIDEPSNSFQPPQFPFSIRRPFFSLPLYNAKTVLYDIIQNYYLGQPQAKDATTTTTPTIYTTESDYEDNIVI*

>Woma_00011349

MKLTLITANVFVFLAIVQANFQDGKGILVSDELEAKQNGKQYRPEFIIHTNPSRDEIAEDEPEPKQSIGENTEPLSRIPVLFDITIHENNHFVQSGKNPDGTTAAY*

>Woma_00011348

MKLILIIANVFVVMAIVQANFQDGKGILVSDELEANQNEKQNRPEFKTHTNPSGDEVAEDGPEPKQSIGENTEPLSRIPVLFDITIHEKNHFEQNAKNPDGTTAQY*

>Woma_00011327

MKIGKLCPLFLALYIWVANGGSIPHLTKSRFDDKRIRIDRNVIFPLENINTINNLVPETNQTFNTNNNTFRLGASFNAKLFDILSSSAHHKNLVFSPISLQTLLAFVFTMSGDKLYEELSELLGVPKNPVYVAKIFEGILQRASSKAPPTSLIMANKLYYDYHLGAIDRSVHILAKNSFDCELEEIDFLVSQKAANIINAWVSEKTRNLIQDIVTPSSITRDTSALLLNSIYFKSEWLIKFAIYDTEIQSFYVNKNQQVPVDMMYNEDVFRYGNFSKDLQASVVELPYNATNLTMLLILPNDVEGLAEMERKLRYYDLDEMAEKLKRETVTVKLPKFKIEFETDMIETLQKLGLKALFNNDGVINLFKNQQKPLKVDQFKHKAFINVNEAGTEAAAVSVAKYIPLSIPANMKYFIADHPFVFIIKDGNTILFMGHVTQF*

>Woma_00011358

MKGLLIFVVLLSVLIGIGYQKFDELTRPLPLPKFDTNKYWGPGDVSKYKEDKTIKPFQINVDAEVITDLQKQLNRTLRLTEPLEGIQFEYGYNTEAMTQVVEYWREKYLTKWSERQKFLNSLPQYTTEIQGLRIHFIHAKPTAETLKKKKVLPVLLLHGWPGSVREFYDFIPILTEPAEMSEYVFEVIAPSLVGFGWSQPASKVGMNTAEIAIVMRNLMLRLGFDKFLVQGGDWGSIIGSNLVTIFPDSAIGYHSNMCVLTSPLSIMKSIVASFKPEKFLPSRFFIEHHFPLSEKLLFLIEESGYFHIQATKPDTIGTALLNSPVGLASYILEKFQSSVGATYKQRFDGITKVFTMDAVLDNVMIYYLTNTATSAARLYKESVSKEFLGYQLDRVQSPVPMGCARFRYDLPAAMDWQLLDKFPNLVHSKYFNQAGHFAALEVPVMLYIDFAEFVAKIKL*

>Woma_00011359

MKFLMVFLAVIAFAAIASAQEEDNQQENFDEFRPFRFPFPRKPLYLNRSPVFYDIIQNFYLGEPQLEDSDSSDYTTTSTPEIFV*

>Woma_00011347

MKFILVIVGLLTILALGLAKEQKPDGEKQGETIEVPDESNQQIFQKEDHKRNTEGSYEIHHLRPLGRLQPIPQFEPKSKSQTQQEQEPNQTGNEIPIIQPVLLDRNQFSVLNNAGNSNSFINGDAPSRTTTLPST*

>Woma_00013390

MFKILLSFLIVAVALNVALACNGYKARIVKLENCAGPDGIITIDETSSIKLNKKCEIVPSGCVVNKPFKTAVSNYKITKDGIVVKEGKIDMCSIADHASGEVKDYLKLFGAPASCPVEDGKVCGNDHSVDLSKYKSLLSMVRGNIVIDSDIEHDTGKSCFHVELEITKG*

>Woma_00013405

MALISCVNILTSFLVLIVFLPSQLEAFYTPNTGCHHNGTWYADKSLVPTTEKCLNCQCNKKTLVCRLKVCPEMPMPPPRGCVVVQKKNTCCPYLSCARLDAFYKIPATRRIIAYLDHYERESIDRVVNENMLQRRSDDTDVELYVCVKNGTVYKSGSAMSSSNLCSYCYCIGGTEKCVKPKCMLPAEGCKPIFVDSTCCPVRYDCSTKQTGKSSQEVRYRKTSNKHYLRMSQRLQRNRGCTVGQQFYVEGQKMKSDKDKPCDICFCIRGQRKCAPKKCAPALRNCIPVVPKGQCCPSSYDCGSQRDYRRSQNSRQFNLFSLLFGKDDEQDENAATEIPVQYPHDRDPAMVRPVRQKTVTTEKSFFDSIREGLEFIDSNNNQMLKDNEDLLTDTKHVRPSPTPIPLGMLSMPSTTEVSFLDLLLGPSEPNNHETEKSSEDIESTEEKATPTSTALNWIDLLLGPDEEGDLKNSANNILDKVDETSTLESTTSIDRVDSENIIENEETLGELNAEEQENLLATTTESPTTMEELLANEETTLTYPTTLAETTEEIKSTQSTTLTTTTTTTTTNPKPSSATGATTKRPSKTENQPTKPVLEATKNATQQKPDFLAQFLDGLSDILAGENKTHVTTQKPLNIKSNLNKTTTTVKLPTPKPMPMFKPLPVDKTYTRINSKIANLTLKPPIISTKEIMKTTFIPLNSESKKNATKPLLDTTQKLESTNTVAISSKKPQDSTSSTTTTTTSTTGASTTGKPITTTTTTSTLKPVVIKTNPTILEAELLDESLSPTLPPSLPNLKIIPFLPTDAVKADHSPGSSYEFYHHSAAPISRIDYDLYDDANIYPSITEKYPLYPDLEDGSKAEYIYKFNVEGPDSLISSNAGKFDGTLGSPAFLKYDFASASALASTKGFSPPTKTEGGFVPKEPLILDDDDHDNVSVKVGDSYHVTHHIIDITTSSADILNTTKVIEITTPDPFKDVIRTEPPPDLTSLIEDKLKNLINKSSITAKITKTNNQVNEKPQNIPETKTHSKQTDTNPMNKKTETQIKIEMTATMNTNNNNDGNYNSKIGTANAGDTEEKTVGQHKQNIGGADMLDKANVDVNDVTTVPPFKTVPTAALVVQKQLIEGNTTRPLSVSLETATAIELKKKQTTTMTTTTKRPITAIKVNRNKTNATTTTGQRLKQTQTPQKPLKTKNPASLHGSETKLLASTSISSSASTLSTSSTSSGSASTMKTKTNATRRTASTKPTKQNNKTATANINNTTSTTTNSTITKTMNQVKGNTKPNVINIDRTSVTTSRRTAKPTTNANTPIAHKKKIATGVAATQTTSRPITTTTTTTTTIRTTNIQTTPSPTPSPYAYHVEAETTMKPFFMSPFDKLPFMDASFEVKRNSATPSTTTDVANFAPPLQQHFAVQASVADANYDQDVVDTDITMSSTSNSRNKLMGLVHYKHTLNQLEGLEPSASATEQQRRPQHSSLSDTQFSQVSSPSSSSTTSVSTSSSSSSLNNLIDPTGILKLAGCNIYGRMYRVGRIIVELSSPCLECKCTEIGVKCGPLDC*

>Woma_00010796

MKAFINFILTCALLLNIISLIQCQTALNCSTNELYFNTTLSPICEANCQDLQRQCSLTTLRPVAGCYCKANYARNLQQICIPRQRCDSDCAQNESPIAPLLRGCEQLCRTSATAFRVTICYRLQERRCVCSLQYCRNRRGHCVKKIY*

>Woma_00010798

MVSFHFIYFRVMTSKLLVFIAIACLICIFPATAQNRCRMTSRLVLSGQLVCYHFCLPTGTLPNHRCYRSNLNQRVTVCPPNCCRIGNICKPV*

>Woma_00010832

MDDKSGDSENMSPRNNDDPSENSQQSVNDADLQEMAAVNDSLDELSNVLDFFEQRTDNIIEQLKELLHSNREIRQELALENAANSVKNLELEKPKT*

>Woma_00010801

MKKYLIMFYFFILFINKAYTDETCPLGRNEVFLSLGPNCDRECANLNKPCPKKGVPHRTGCFCRNGFARNAEGLCVPIKFC*

>Woma_00010805

MDLKEIITFFVICITVNANIVSTLPTFGNEFIHINVHIPSEPSSTKTVLKAKPPLVHQKPAFASHKVIHHYYRRRKPTKKISIEESVPTLENLYLSELRNHHPVSYSHHDEHGHHHTEETFVVKALHDSHYHHLEKPKANVNTFKIIEKYEDDHHLNHKEQGKAPIESVKFIETSDDHHQNHHHQNENPNIETVKLIESSDGHNHQHHYYLTGQHNPLKGYGLTAIETTKLIETTDKHHHPHHYHVHNAYSHIQPPKSDTKSDHYVHYQKPIEEEHIYHKPQADALDIVKTIATIPKDNYKNYETKNHYLVPELIEETLDIFATKKTKIHNYEVPAYQYQQPQQETAAHYHHETPSTHYDYKPKQEISIDELIEHKRPQSHVQTNYPHVTPVTEKIPHHYSSSEGNYDSTHVEHTDVAYETQNKPPANYRQYLPPSETPPNFEQSQVAPQTAINEDIQPGDINAKEDSAHAVENPRGPETYTGYDSYSAGHVQGVNSGGYSQHPP*

>Woma_00010786

MYPLNILIVITKLLVIMGVDSLPITTTLSNSIAHKSKDINQQQQQQQQHTGALSFISTTSAVAAEAAAAATVASALTSASSSSSSLWSNTLRLRKRHRKRHSIWYDYNYYYNDNSTILEWSNPCGGEFDPNAKQPKRPTKKEQKRIYNNLRQTALNDFNYLNGSHQQDIDISNIEIWWLHNGTYKFLPKLKMNSSIALKRWYRNMQTYVATFAYLHRIQFKYDRSKSQRESATSLELKELLKSSRNMLCELETAVNKTYSYKNRQIPQITRLEMNQRLKLRTKQRFKDNANVLAETDSIDLKFVKFHYFEYLKSMWQLLRKFNRRKGQVALQQKPKQLNRRLTNAANANKQFNEVDKALNKNQTKRNKKRKNTKSKHNLQDLEILFYIKQYFQDHDNYDDDADDE*

>Woma_00010781

MILNIFAIVLLIKGISAQNCQLNATHMDKKWIFATKPSANSWDVLHTDYIEDRTYLYLVCKNINNTRNIFCNRGVLQEQTSTNTKCPRHLEAEVVLQSINDNSCRRMEGNIYKVVYNLANNVQIPLYDVCYSTVKEQTMFTRHRAYGFKLAASSYVRPSTFKTSNVVTRQRAVSFETDNVYERFKQLLGQNQTLIPNSKSLTIARGHLTNCQDFPTYDQMDATFKYVNVVPQFSGSNLSNWKRIENWLHRLIAKNTYAEVVSGIYGTLTLEHSQTRRPVEMYLLPNQKNNIPLWLYKVIKYGRNCYAFAVLNNDFNNETNVDSPICRSITCPNGITFTTPGSIGYCCNYNHLITKVGRHAALC*

>Woma_00014096

MFLLVLFFFILGQILQGHDKKKRLRKWQKTNSIPIIIWWTPILTGYSETRLCDKYFCQFTTSRKEVENAKAFLFYGSDVKIDDFPLPRQSHHVWGLMHEESPRNIAFMPYKEWLQNFNFTSTFSRHSDLPITTFYLPHSDNLTTPAYTIPIGQKTILNKDQGLVFFMQSDCDTMSGRDDYVKELMNYISVDSYGACLKNKELPESLQNIQSDYLNNLYAPELLKFMARYKFIISYENGICEDYITEKLWRPLIAGSIPIYFGSPSIKDWSPNEKSVIDISNFSSPEALASFLKELNTNDRAYNSYLNHKYNMIAPITNTRLLNELGRRKSAMYGDNQFQSFECAICSFIHEHTDITQKHIANELHCQCPHQPVYPPMTKKVSNHDDWRSFMSIGKCKAILLDRLLRRNKNYTEAEFMDLLTKEVSLEFTLLMINVKDEPKAKHRFADNEELIGVYQSSFGKFEMIYYGEIQGIKAQGEVKNLY*

>Woma_00014252

MKYLSVLCIFVILALAAAADLESSAAESSVAAKNPEKRGIYGFGYGLGGGYGGYYGGHGGYYGGHGIGYPGIGYAAVHQAVPYVGHGYYPYHGHGFYH*

>Woma_00014290

MFKILITFAYIFTFSWAIYVPEHTISSQNYESVYSRTPLVFEENKTLKLDLQQQSVNKYHYVHNNDDNNDGQQKLITAKYTKTIEEQQQQTLPLLNKRTPQKSYDIVNGDDVVNAKNRTSKNEHKNLEAQPTGLAELQQQQHQLQSNYIYTNDVAVIEEEEKGNNNDYLLTLSEHSRQKRQLSDFLIAPNTRWCGRGNTANGTYNHLGGASMADKCCRTHDHCKLYIPAMSNRFDLFNYRPYTLSHCNCDRRFRTCLKMANDEDANTIGKLFFNLVQTQCFILAKEVVCQQRANDGSCLKKHIKQKAYLRNNKKY*

>Woma_00014246

MLLRFLALLCFTVAVAKTHELSSEELLSKTDNLAEKVNTIKNDLKDIKTEVLDKYEEQKLSWQLQAFSKWYQDMVAEKCQRDLSYIFDGWTPIQRRIDGSQNFYRNWSDYQQGFGDKNGEFFLGLEEIHKMTVKNEALELLIVLGDHNEQIAYAKYDNFKLGSNEESYAVKAVGNYKGTAGDALTALIGEKFTTKDRDNDNSDLYNCAKLWRGAWWFNYCYQSHLNGPYLLEPLSESGEKGIVWDLWHGLNYSLKFSLMLVRPKTQGNK*

>Woma_00010975

MYFKIWLKTLFLSSLLSVLLVREQPLLGPGAAFPPFTAPEFHPTTPENWKGATIHPTGLMKEPAVVPGRNAAAVAAFTAQGQTQGAVMMVYGLDHDTSNTDKLFNLVCLYGNVARIKFLKTKEGTAMVQMGDAVAVERCVQHLNNIPVGTTGKIQIAFSKQNFLSEVINPFLLPDHTPSFKEYTGSKNNRFLSPAQASKNRIQPPSKILHFFNTPPGLTEDQLIGIFNIKEVPATSVRLFPLKTERSSSGLIEFSNIAQAVLAIMKCNHLPIEGKGTKFPFIMKLCFSSSKSMNGAWNNAANEGMIEKENDSEIKPDSYN*

>Woma_00010979

MKFILIIFILQLLLKSQSSSWDMEYVGKNLVLPIANFTEEVIFCICKECLMDENVSFLMKWLQFETMHP*

>Woma_00010986

MLKIFAVVLSFYLLVDQAWTACYRKPQGATGYRSPVDENYQILIDGNPATYLPGQQYNISLSAANGLKFISFTLLVESESPNTFLSTTAFGAEQLLGRFEIIDNAETKFSTLCENMIENSNTNAKNRIDVAWIAPSDSQSGCVLFKAAILQHRNVWFIDDGFLTKRLCPEEIDEVNSQTPPVNPCCACDEAKYEIILERKWMRNTHAKDFPSEAWRTRLGEVIGASHTLDFRFWEYGGRASQGLQDLAEHGATKTLENEIKEQTGNGYIRTIIKAPGIIYRPNVVGKTLATVRVGPNHHVISLVTKIDPSPDWILGVSGLELCLANCTWIEKKVLNLYPWDVGTDAGPSYMSPDQTQNPPDVIRRITSTFPSDYRSPFFDDTGAPMKPLATLHVTRKRTYERECDFEDIGPMECSTHPWSSWTECNAKCGAGTQYRTRSYKDDKVAENFNCKVVLRQNQNCIGTQCGVQQGADLPGAECELTSWSAWTECSKKCGKGFQTRTRGYTNPYAKEKCQAGVAVELQQTRSCNGENCGGNINTSFYDGNSNDMFQTNFEPEEQQPNPFLNRKSERKPFNSGFEGPIWNNRGQEEESTNFDINFNTDNNDFRTAIKQPTYNFGNEEQEGLNINNIYGQEKLVGKVRRPFQEEDSQNLYGEMDGNFNDNTRVRGNRNKQQFPTSLRPTENYGAESNSPRTGITSNTFYTFNRNLNPHSFKTTDAEEEETNNFCLQKPMASPDQCNKKRLIVLNYWFYDADDGECKLFTADNCDENKNKFFSMERCLETCSQTVKNQQEIMTGTRQGFSNTPYRSRWN*

>Woma_00010987

MNWLTLAISISATLNIALACSRVPTGTTAAKSPADESYVISVAGNPQTYIPGQKYNVSITAYNDQTFISFMLGLESETGGGLEAVGSFEIIDLAETRYSPRCANLIENTNTNVKTRIDVVWIAPAATNMGCVLLRATVVQHRDVWFMDDGFLTKKLCEEEVDDIDSQPSIEDPCCACDEAKYELTFEGKWSRHTHPKDFPANSWQTRFSDIIGASHTIDYRFWQYGELASEGLREVAEHGSTRTLESELKDKSDQIRTIIKARGITFPNTTGKTFAVFRVDSKHHLISLVSMIDPSPDWIVGVSGLELCLSNCSWVETKVYNLYPWDAGTDSGPSYTSADQPQLPPDVIRRIKPNFPNDPRSPFYDPSGAPMKPLATLYINRRRLYEKTCTVSDDENEPPECATYGWSNWDECSAQCGPGKQYRTREFKNPAKARRAKCRTSLREEQDCTGNQCNSFSEDVAAAIETENLHVVHIQETAECAISDWSNWSSCSVSCGTGTERRTRHYLNPRAKKKCQQVSRVKLQEQRTCKGVDCGGDISNNNGNNNIENEAQLDGTKSLFGNIDDDIRYRDLESFSQKLDDYIPPECGVSHWSDFSPCLGPCGGVGTSERQRIVWNNDEVYGVRKPDHDSNLDPCRHIKRKEVVNCTLPSCDAIVPPFCYEDLKVSSCRDSDVANYWFYDHMSDQCAIFWADKCDKNRNKFPSKEACEETCRQPRQKMELQSENLQVDPIDCLVSDWVSHACNATCGEGVQIKTRKVLRSPKYGGKPCPKHLVRLEKCYQRCDDIYSVGGYVSSSSYNERRRNHGGNKHHSKNQANEKTECRYSEWSAWTPCTVSCGDNSYRQKTRTLLNTDLSYKCKDRVRIEKCQMMPCLFNSNDELDKW*

>Woma_00011030

MDVNFNYAEDAEYEEMLNKFHATHLNSNILELPSPVEYNMSTMTVSPCPPVYNLESSTPTFKREFRKQGVKKTSLNFNEIKVEREYQDSLDDMQYSEEMDPNKTYTTKTMDPRQLALMLAALEESGE*

>Woma_00011016

MFNVPHPWYNLGDLTTRPVGWTNCILYKQNNALKQKCVAMFSESLLQRSVDLLLTSETECNEEDNPLAK*

>Woma_00013840

MLSAALLRSFKPLKMRYLDVLFLSLFLNACIVLAAAKDSNSWSFSLGNVHLFDIPKFSGSFSLFGASSEANERHANSVENIDSTEFSIDPDIIEDSLLKTPDLIKKYGYPSETHNVVTEDGYILETHRIAKPGAVPVLLTHGLLDSSATWVLMGPNRGLAYLLYDKGYDVWMTNVRGNTYSQKHVKYTPKHATFWEFSFHEIGKYDLPATINYVLNETEHKQLHYMGHSQGTMTFFIMCSERPEMCDKVMLMQALAPVAFMKHAKSPVVEFLAFFHEPLALLLKLIGAHEFLPSSEFIKMFNQIICDEDSITEAICSNVIFLVVGFDKKQMNETMLPVMLGHAPAGASTKQMQHYGQLKRSGHFRQYDYGWIRNHWRYNSISPPDYNLTNVRAKVALHYSVNDWLATPDDVEKLHQQLPNVVGKFKVNYADFNHLDFVWGINARELLYNKVLKLMEMVETGVF*

>Woma_00013844

MLPQKFHMIVTFVFTVFALVVLLLPAGQCGPVPKVNEHAMLMPKVPQWHCLRYFKHDVLMMRRCRHLRVPTAPRLGDVKKLNQIFNYNVAKMADGKTS*

>Woma_00013845

MENKMSPFNTCLVIVLSLMMLVLVAARPQLNRYQHIAVIENDAWEQSLPSELRNPFYKTPRVRNALAKSSWFGPGEMPVLDREAEKIARREIYNVLSHAGLIERRNFV*

>Woma_00012754

MINIFSRRMSSGKRVLCVGMCVLDIIHVSAEYPMEDTDKRCLDGYWQRGGNASNNCTVLRKLGSKCEFLGMTSSAPAFQFIYDDCNQRMIKTHNCPKTEMNPPFSSVILVNAKGTRTIIHCNTGFPILTFEHFRKINLNDYCWIHFEGRNVEETQQMIDMIRNFNRTQAEPIKISVELEKLNKDLYILAMRADVVFIGKDIAHHLGWHSAKEAVLKSHEIIEDLKVSSIKDGVLSEWPRQTIICPWGSECTDALDGSCGNEYFNIKAVHVENIVDSLGAGDTFVAAVIYALNSLKKNLPEALSFGNQVAAYKIQHRGYDCVENFKI*

>Woma_00012753

MTYFALIFWILSLLTLNNFIKAEEQRENFKKTNPTNQNMCKSQYNGAILANPDDCQKFFICINQQAHAYNCDPSYHFDKTTHSCVPGSSCNANANKPIVSCSEGDVRAVQDDCYIFEACIHGQYQKINCPANYYFYAPNSMCVPFKYDAQFKCSCLMPTHTVLENKNNCETYYVCKDKSAILKKCPLGKYYNPQINTCITDVTGVCLMKPTMTPNIEKELSKQNKNDMNVVEATCALLGTEGVQFQTFAKGCNTFYICINGQLYEQHCPQDFFYDIDKRYCVFDTEHKCKTNDDESIKVNAA*

>Woma_00012770

MEIKKGEKFLSFFIVISGFFLILTQADFVSSADYTHLCRLFKPNTQVRVPGSCDQYMICNGQTAKVLKCDKQLKYDAKKQKCEATVANAKQLCENRCYNSKDGVWKADPTNCRSYYYCQNNQPLVGHCDINFHFDESKQMCVYTKNSKCIDVNNICELVPDGTTFRNEANCAYYYECKKGQHESVKCKKDLYYSVEQGTCISKTSVACNAHPKVECKKGLKNYVGLLPDGATCRGYFKCANKGKVEDIDPMWYQCPLGTFFSEDHQACVHPVDAKCSADRCESRGNALVNSGNNNCHNYLICENNKTVKEVICDNDYFFDEIIRACVPRIIYYKCCDVPDSYLNNTKVNY*

>Woma_00012747

MKVLLYLCAFIVYCSGYSLTEYNYLCFDRHGDYLKHPNDCQKFIRCSHGRAYEFQCPGNLRWHQKIKSCDYIYRTKCDLSIGESSSDRDTVTVNVTEENIKKCITDNIASVENFTTNKILELSTKQITVNLTENYNGNSRVELSTELVTVLITKLSNVNFTTDNSSDLLTTQDNLHFKNQLIENFTTDNVSELTAIEDNLTTKPFTASNFIHNVLELSTEVNTVRLKESLLNNSAVFNETELSTEKTTLQLTEQLLDNTTMDYHSELSTEENTIQLTEQLLSHTTVDHVSELSTEESSEFLFNKEPVSIKITPIQLTGQILDDTTVNSVHSESSTEENIFQLTEELLNHITMDYVSELTTEENTFQLTDKSLDNNAGDKVNYVADLSVDEIPIQLTEQLFDDTTVDKTNYVPELSTEKSTIQLTDQLLKDTTADYHRELLIEENTTQLLGDNRVDYHPALSTTDNTLNLTEKIVDDIAETGKQLVNYHTDNVSKLPAQESIENSTEINIPELSTAAATKTTESKVQSNFKGNEADISRDLVDTTMIYGTETTTEIVYSHAITNNKNGKKLFSKLSHQLLTSTSTPTYSQRPPDSTTQDPRLPFRAIECQGENNRQQFKALKCYQYYQCFAGWAFLLNCHEGWWFHADMKRCIQNVDNECSLLAVAVLATIAISYAVDEECLLRKARGLPVHWPNPTDCSSFYRCTNKNVKRELLCPEGKEYNPSKGRCAPKGKHLCKLSLKAPLTDAENPCAGAISGTSVPAANSCRGFNICNNNVAYPQSCDAGSYFNTKLLACEPDNDLECWENLCVNKKDGDFAANNKDCYSFYVCLNEQPKEQLCPEGSYFNKEDLNCQPGTCPETTTPVTDTTTPATETSTPATETTTPATETTTCTTEITTPTTTTCIAETTSTTTCTTETTTPTTTTCIAETTSTTTCSGKTTTLTTACAAETTTPTTTTCITTTTTPTTTTCTTPTTTSTTTTTTTSTTTTTTTTTTTCTTTTTTTTTTTCTTTTTTTTTTTTTTTTTTTTTTTTTTTCTTTTTTTTTTTTTTNTTTTTTTTTTTTTTTSCTTTTTTTTTCTTETTTTCAAEITTTLTTTCAPQEDKVCPSSNCLNKPDYYTFPATNTTFGFCRCSKDKAEYYSCAADGYVYNTEFGVCVKSDDCEDEPTTTPPTEKLCEGVYKEGDFVPNPDNCMLFYICFDGELEESSCETGNYYDDKIKACMPDIDGKCPDCQQPQPNIRRNKRSIKRAPTCMEGAKTPAADCDMYYKCVNGVQLSYGCGKDKFFNLETLSCEYDLQHKCWPAASCIAGEKRPHPTDCDKYFECCSGTEELKECGKNYYYNRLTQTCTLDTKHVCWALEPCTCPGGYKEGHISAHPDECDKYYICLDGQQVLQDCGKGNYFNKKKGLCELDVDHVCSTDDSTCVCPGGYKDGELSPHPTDCDKYYICVEGTQVLQDCGKDNYFNNDTLTCEPDLYNECWAKTPCTCPGGYEEGDFSAHPTDCNKYYICNDGHQVLQDCGIDNYFNNDTLICEPDTYHVCWPNLCMGQPKGTLVANPQQCNAFYLCEDDNSTPGSCYEGEYFDAEKGTCLPDFKATCYDPCANETSDGVVLLPHPICNKYYFCNGGKTFVGECVTGSFDPNTGVCNETKICQPNPCKGQIGFLSFPYPYDPTKFYVCIKGQAVLKQCGPNREFDTEHGICMLVSNRDCPACPDMETNTYPTLDGENDREYCYCYRGIAYKSECAIDYVYNPDIGTCYSNAPCDPAFCIDKVDYTVAPNRNCTKSFCMCINHKPSIVNCPNDGTFNPFLNECTNIEISPECDQSSCQGKADGQLGEFTKGLKPNGICVCRSQIATFEPCAYGSIYNPTLKICQQKEINCNSKLCTGLADNTNIPALTTNEGYCKCVKGQATYYNCLDGGLFNVDTAECDAPVCPLSQCKFMGDSFKPTNTSAGTCICLGNSVDYLPCDIGQLYSEKFSGCADKCKLSLCPANQANYNIPSAISKHNYCTCDGLGNTVFKECSPKYYSYNMAKQQCVPDVATLCPKACTTTVEAYEAEGTLEGYCICNGKGQQIFQPCGENTIYNPSRGVCIRMANSICNVEYCQDATIDSPITMPAEDDDKNIFCYCSGPNDVMVLTCPAGKEYVHELRMCVNPLEANSKCPAGTANGDKVPDDTSCNKYFICENGEMELKTCPKGQYFNKNTDNCCVDTQGICPAQTELTCLGAYYEGEMIKHPIYEFMYYMCENGALNEYKCPKQQVFSAYNKACVRKSYGPRKRSIDIPTTTNAINDHEFNCLDNEKRSVPSNCSQYEVCINQNWQLETCPNYRYYNPEQKVCLEPRDDFVCSYARITSLPPCDNKLNHQVYQAKSCYQYYRCENGKWRLKSCGREHYFQAHIKTCLPIPQDTQFQCNLAFNKSFAKIENQALTCQHLSQRFYTKNCAMFLICLDNEWWYQYCPAGMYFNRSLNYCVPDLQQECKDVIKNLANLNMEEYSQDVCNQDGLLRPSAMACNRYYVCLNGKWQVQRCAYQEFYDVANAKCSPDTEGQCTKLNRDCWLNEKRSVARNCSLYEQCSSEGKWLEMACKPGEMFDELLQQCVGNQDICSKHGLKRVCRDNESIMMASNCTKFYYCSNDSWQVGSCLKGYMFLSELTQCVPHREENICSPLDSQDTNPSLKSAENKRNLCQSKLDGSSVAHPTDCTRYYICIQETTSHEMQCPQGSYFETIMGYCRPNNGSCRVPLSGVCANATDNSLIAHPTDCQAFYHCSTVNGTELLYCPKGEYFHNQTLECRPDNGECKNNLTIKQKCAGLAHGSRIAHETYCNIYYACVKGLAIPVECPNNYQFSALLSKCVFDKEQQCQNGQLINSNVSYSCGNLADGSFLADRKDCTRYFICSEGQALPKKCANSSYFDSELLLCVPDDGSCPFVTDNAEQPKPSKPDPEICEGKNGYLMADPFNCNNFYACIHNKLKHERCYSQQFFNSTTQQCQPIIAAEIGPKSNITTINEFNFKTVNQQCLDEFTSTQELCKELTEGTSLAEPGDCRRYLTCQETDEQPLRQRCRNGESFDSLLGFCRQNDGTCLMENGQRVGECSGKHGQLARDPKNCRSYFVCINGQKIAQTCNKDEYFSRTQNICLKDTQHVCNPAPTTEEPNKQSCIGLSESTLYPYIQDNCRSYFQCIKGVAKIHKCADDLYFDPIAIKCVKNKTECIMPTQTETQTLQRSPVFDQKFSKLCTNKVMEVPYANIDNDCRSFYICHTNGEITHELCPLGKIFNKDFNICFAENKTICHERFV*

>Woma_00012744

MKKTCILFLVACYAAVNTNAAYTEQELKEICQGHEFQFLAHTDIHKYIICLQDGGLEKTCPNTDCFDELNPPCSATCPASNANDLARCNDPKVNVLDRIPDSLDCEQYYLCMGASTDPLHLRCPTNQHYSEKEKKCMGVFEANCAVTTKWCQNKRNGTTFASENCFEYYECEGQQTFKMSCPFNEHFSAELGKCIKGSCFGDTRKPSCSNVADGTRLPHAKCYKYYVCLNKALFEAQCGAGFHFDAVAGMCVRDMNNVCNDI*

>Woma_00012743

MSKAYRLKLLLPPIILALFLAPSTANWQACLGQPNGAIIPFRYDCHMYLYCQNQTPNYKRCPYNQGFSSETSSCVVYALSDCNKGQNSNQGGTCNNKKRPENKQACKGQPFGTLVPYPGDCAKFLVCNCEYPTVKDCPYGTWFDNKIKVCNYPQYVDCDNGGSTGVTTTTTTALPGTTTSMNTATTGEISTETSTTADIPITLSTTTEIITTTVINDWGKDCEPPEGMSADICLKYDNGMLLRYPYNCNAYINCTQGCPLMQYCQYDKVFNNFLKICDTPDTSQCIELPLPSPTTTMTTTTDGESSIETTTISYTPQPECLPPIGFDSDICQSHSSVFEGYPYNCTAYVVCREDKTCLEYCENGKIFNNLLGICDEDIQCHETPLPNTTTTEVPNLSTTTELPVITTTTLPADVDPDLCEGKQDYTTYPYEKDCTKYLQCFNDQVKVEDCPQGWLFNAQLMLCDEGTGDLCFRETTSSTSTTSTTTEQSITPTTTTISTPSTTGEPIYDLCLGQPEGTTLPYKENCERYYFCRGNGHYYIIYCPYNNYYDPYSGNCGPDVSPTECQEPITTTTLSPEKQCANKPTGSTLPYESDCSQYILCDGKGNYYILKCPYNNYYDPVSGDCGPSNNVNICRDPITTTTPTTTLSPQKQCANKPAGITIPYEPDCTQYILCNGKGNYDILKCPYNNYYDPVSGDCEPSNNVNICREPITTTTTTSTTSTSTTSTAEITSTTTTPTPEFPALGLCGNLSNGELIAYPDNCQKYISCVRPIPIGFYCSNDWYFNEQQQKCAPNWQGSCSEIPYPEGSTIKPPNSICKGLKEGYWQPYTKNCQFYYECLDEDNDMLKICPKNQYFDPLRGVCTTQVGAEYCKQDFDTTTVLPSTGGTTPTAKPQGICAGQSIGCKISYPHNCTKYIECAVPIPVGHDCPTGLSFSSDEQECVSPEQSDCGLLTTTTTTAEPSTKPLNLCTNKPDLTFLPYQQNCAMYIICWEDMEHLAYCGSGEYFNINEMQCLANANENSCRAITTTEAPDTTAVTSTTTPDPNLGPCYEQPEGTKFPYPNNCSKYIVCRDPLPVGYDCPEGLEFSDYYKECVIPQLSDCGMKTATTATEISTTTTPITPSSFTTSTPQTCANKPTGTTLPYPEDCSKYYMCLNGQEKLVNCMPNMYYDPQTGECDDISPIACKSSTTTTTEATTIPTTTEQPLEGPCKDESNGYIFVNIANCAQYYMCLEGSPVLCACRRDTFFDPNTKECNRQTSAIACKKAITTSTTIETTTETVTKPTTKPITATTTSTEVTTITPNYSTTTVNPEPAQYNICCGQKSGTYLAYPGDHTKYVICKYPVPDVYTCPSGSYYETITSSCQSVPANDFSKPNCDTENYGRYMVFEGDCNKYYFCYGSKLVTSTCEFNWYFNPMFSQCVPRDYYNCPW*

>Woma_00012764

MFTTTTHYILTLLALILFFYGIPTLAAYEFNPDLLCTYIANNTKIKDPSACNKYITCVDQKPISGMCEGNLFYDRNKLSCVEPNTVKCCSSNPCAATPGVNGFVADPYTCNGYYYCLNGEGTHGNCSPGMNFNPDTLNCIRNFPCELEILPEDYCNIVPDGVFIKVPNSCTEYQTCWRGELLNGTCPDTFYFNALTGKCDYPSNVQCNEVTTLAPEIPSDVKCSKSGVFISDGVSCNGYYYCKGQPDGSIEPVHNKCPLLRFFDPNSGGACVIRTNITCTHNRCVTLGFKNIQMANINNDGCTGFLLCQDGEIIGRSACPSEGTTNKSDVLTTIGIKTTSADSETTVKVLNTTTKVLDTTTA*

>Woma_00012765

MWAKSLLASITLVFLAHYANADYDVTQYCQLVPAGTKLPSLTSCQTYYTCLSSSQAQSARCTGGQIFDKDSQTCLPASAGNCAIANSNSNPCANKDKVWVADIYTCGEWHYCSNGAVLGSGVCPSKEIFNETSQLCQYGKCSTHIEGGVIIIENMCTIMQSGQFFGDFNSCDTWHKCNGMTMQSGTCENGLVYDTKHRMCVRDNGKTCNAQGPSSTCTAAHNGNLTADRSICSVYYQCVEHNSNWLWQRHQCPTGQYYDVRMQGCNRRQAAMPIATCDRCEFATTTWVNAVNSTCTEYLTCSNGREIASGFCKAPNSYFNEQQQLCLENSNSLANYRMHNGACALPINTPTTTSTTTTATLPPSSPVTAHPANSTNASNTTLHTTPAVHKTAPVTSTNSTTKA*

>Woma_00012767

MISKILRACMAIVLFSTASADYDAAQYCQLVPQGTKFPSLDSCQKYYTCQSQTQVTVASCPSNQVFNKDYQTCVAPSNGNCNIDLGNPCQNINGKWVTDPKDCRVWHYCQNGKILGTGSCSSGQIFDDRAQRCVHGKCSSDGSGTGNNNGISNLCIIMQSGQFFGDFVNCKTWHKCDGLEEKSGPCPNGFVYDPQQRMCIDSKDNTCGGPTQECNSDSNGKLVANSKICSNYFVCTKQGTKWVWQKETCTNNNYFNPGTNSCLSRQKATPVSTCDRCQYSSLQWVNAVDPKCQKYYICQNGVEIRSNTCKSGYFNEQMQGCVEGSTLKSYSEDNGACATEPTDKPTDKPTDKPTDKPTDKPTDKPTDKPTDKPTDIPSDKPTDKPTDKPSDKPTDKPTDKPTDKPTDIPSDKPTDKPTDKPTDKPTNKPTDKPTDKPTDKPIDKPTDKPTDKPTDQPTDKPTDKPTDKPTDKPTDKPNDKPTDKPTDKPTDKPTDKPTDKPTDKPTDKPTDKPTDKPTDHEKTPEKTPEAK*

>Woma_00012760

MSANKFYFSLYLITILSFITLINSSPTTETETSPGFIASIISTFQRWKSESQPTLQKWQDNSFHNKETSEISAKRNKLWQQLMATHFN*

>Woma_00012761

MQTYILGLSMALLCITQALEDLTQFCLNKNLGDVIAHPYDCRAYIICNREPSVYYCDTKFHFDSEHKICNWPEYANCQAQVGHFTTWAPFMAIDIQTGNIVDPLTAYDPHNVMCRHFGAYFLPHPTRCRSYILCAYGHMHEHTCGQGSLWHYKLKQCVLNINAECYNLTPQEAVEIEESRNEPLNDANVVCYTITTVSTTPATTTLTTTKITATTTIKKTNSTFKNSATTTHRPHATTTTTLIVPAKKPHTHAFNIVCPHKKQSYVAHPKDCSKYFMCIMGNPVLTSCPHGLMWDSKKEFCDLAKNVKCFA*

>Woma_00012762

MKGIQMTLGWMLLIVADLTGSSSAIYTMNETCLYIQKGFIADPASCSSYGYCDNGSLISVGNCPKGAFYNADDGVCSYKPNCRLSPGDVCQGATDMKYVADPLNCSVYYYCANGVSTKYSCPKYQIFDPINAQCNWASKVPRTCNDASICLLVQNKVFAADPKQCGNYILCRNGSGLSTKCANDKHFSPLSGMCQKTKYCPGAGDDDDNSSGNTNSDLQNNPKKCAGKPNNQKTFVSDGQTCYGYYICTGDGEGGIWGKCPINTHFNGTKCVTPYTYPCPHDRCGNTDGSFMGAIGNNCHNYLNCKNGRSISQNGNYVGTQCDNGYFFDEYLQLCVKNNPANANRNYKLCPAVPKPPGADK*

>Woma_00012763

MFKAKLLVICSAALLGFSFAKSSSIDELCALAPGTTILRPNTCTDWIKCPSVRGAKDMEEGSCVFGLYFNKDTGHCEYKDNVACPYEAKPKNRCSKSDDGIFLADEENCSGYIYCSGGEELKSTCPANLVFDPVNKACVYKHQYACPDIKQFSESHPLCLSLPDGLFFADRLDCTKFSECKNGALLTHSCNSSFGWDYFKGICAPVEEVECVPNSKKPEPELKVCIDRVGPVADGLSCSGYYFCKKVANDTHDRKPEHFQCSAGLFFDSTTMSCRDRLNVNCGLDRCEGMGNKYVNVAGDCRAYVLCNNGVANTKGICPNGHYFDERTQGCTPQVVSYAACAAK*

>Woma_00012768

MGVTYRLAILLGCLVCFANGISLQLDSGNNICRLFPDGAIIRKPGSCKELLKCQNGLSTPHSINCNANEIVGLDKMTCVTKKDTYCTQTCPKNSPKWISDKKNCNEWIACKKTTVTSTGICPPGQIFDQAQQRCIYRPRKFKCERKFDVCDLAKVGQKFWDEVNCHKYYECAKNAKTIHKECPISTYYDVRSGECIRKAEVNCYKHPVPVEACGNRKLAIHDRFVPDRATCRGYFFCRNLNKNGTQIPDEHPPWGQCPLGYFFDATLEACRNQNHIKCLEDRCDGIVNGKVLSEMPGCQHYLVCENGMTKIPEKCEGNKYFDPITEKCVDTKISYPICEP*

>Woma_00012769

MNERLFICLLPLAVLWTFMPLNILAQRYLDPCRLFANGTKLRDPDDCSQELICINFKSIKGLKCTGSTPFYNKDTNKCVKSLDQSTCDSQCQNKTGKFVRDPISCSGYYYCQDDGLALYGQCPLNTHFNETLQYCIHSYKSNCKITQLNFCAIVKKDVKFKDESYCGGYFTCNSKKGELEHKNCTDKARYFDASQGKCIKEPIAGCHALPAGVCGSTLSPKRNKLVGDGATCRGYFHCANLGAGKPDPNPTWLQCPQDTFFSEAKQACLHPLNVTCTYDRCEGRQLTFVVAGVNGCRHYSLCKDGVKVADGTCGNLFFDEEKGGCTTKIKTYPACSK*

>Woma_00012490

MNWPLVLKLLALMQIFTLSENSHVKLPEEFIKTEVNASIGHLPTTVNFSSSSTTPTTISTTRAHPFHDHQPERHLNKRHSNYNAPPLQPLYAQKRQHTYLPQNGPSAQRRHYRVYHRRRPHNRWVPRHHYHHHHIQYTAWTAWSRCTSECVQRRERYCKTKRKCGHIKHIEERKCWQCHPIWPAKSSSLPPHDVQQQQQQQQQQQQQQVNSAPPTIVINAAAAAATAPASATSVITQPASSHHVPTETSNVQHPVIISDHQPVEDHHYLHRHHHETLHSNEDGSNEEENNGDTDFYVIKAKRKRPQRYNRKPESDPFEDYFDYTENNGEKQKEGLISMEQQHDDLKDLNLATSTEFLPDFHDTTPTQLRSMKEYRQNRKRSKTPQRQGGNRNSRRNLKGAVGAFDDGAIEGDIFQYEDFDFDVNRGDHQDDDVSYEDPGDDDTSANTSNFTPSKERATPEHLIPKHRRIYSKWSRWTKCSPKCTTRRYKKCRIREQCGREVLREIAYCYTEGSFCQQWLQAQVQKSPSYDLRPVATRRRDISDDGGTLSNSIGGTLSDFIMNGKGYRGPEYAPMKLKCGIAPIRSNKRNMYNMLKIIGGKAARKGEWPWQVAIFNRYKEAFCGGTLIAPQWVITAAHCVRKVLYVRIGEHNLDYEDGTELQVKVLKSFKHPNFDKKTVDSDVALLKLPKPVNSTTWIGFSCLPRPYQPLPKNMDCTVIGWGKRRNRDVAGTSVLHQAEVPIIPMDNCRSVYHDYTITKNMFCAGHKRGRIDTCAGDSGGPLLCRDTTKPNHPWTIFGITSFGDGCAKRNKFGIYAKVPNYVDWVWSVINCNGNCKMHQRTKTKNEIVQR*

>Woma_00012474

MNLVMASVLLLAMLALQPTLIYGTKNLAFARNGGGVSGNINNRSLRSLKYGKRHSLAESSHLPALSSLPLTSSAKLVMSLESGNVLVRSARGATTTTTTTNLDNQNTSPTNANGNNDGGKRQKNNKKLTKNLASQDNNPTAAGAPSLPSSSSSINRRKHSEKKFSATSGLASNQHQQQQHANKNNGNRQRQMLKSQNHQNKRSGNKANKENVEKTQSESEKAPTTCRYAKSAWSECDPKTNMRSRILTLKKGEENCLETRTMQKKCRKSCRYEKGAWSECINGQMTREDKLKATGGIGESSCEVLRSINKKCNPNNGASKHTAAAAASATGNTSNTGGSGGKKERKNKEKGSRRAVKN*

>Woma_00012475

MTMKSVILILILFLINLVICVPEQKWRESEDGSKFYIETANKYNWFEAWSDCARKNMSLIAIDTYDKHRQIDNLLRKLYNTCPPVWIAGHDNAVHLRFEWATTGQAFTFTNWGQNQPADTTKNEPCILMWTDFQWHDYPCTNKLGYICEENQLVKNTCGKPKKTKGQDLSEFKNILFYFNNGNN*

>Woma_00012476

MKHYRHHNQLATSSSSVCTLIVVALASLSMLPHLAYGTTEVQETPLALPVGEQTQPTTSSQGEIWEEDDHEILIRNERGTKNDGLSCRYGKNPWTECDTKTNTRSRTLTLKKGDPACDQTRTIQKKCKKACRYEKGSWSECATGQMTRADKLKATSDPSCEATRVIKKNCKPGKSKDKTAKEQRKNKDKGARKGRA*

>Woma_00013014

MKFVILLCVLSVLLLQQAQASPVKVVERQERGLARQQASDVAPAAVQASDDDDDDDEDDDDDEPDLGDLIDDDDVLFVSGLSDDEEDDDDEDDDDDEEEDRPQGQAAPNSGASDDDDEEDDDDDDYLDRLFDDILGDEDDDEDDDDDVPAASAQNTVAAAVPAAPAAPIEDIAPVGQAASAGISDGVDLPAESGNVNEPLANVADNEVAPAASSSNNEGALTGDDDDDADDDDEDDDDDIIGDDVIEARREARESKTSKKHTTALKTSSKKSTSALKSKKSLKH*

>Woma_00013013

MLHVLSSKMIILILTLAIVSLRNGNINATPQCSENPISKEKIICTNVKNTADLEALIQNSWIQLQIVNDNTAEFLWEETSQKFFNLQHLDLSHAGELILSDNSFKNLPKLKSLNISACDVETLEADHFSPTNELTTLDASWNLLHNLSDYVQNKLMAIEVLNISHNEIYKIDSNGLMPNLRILHLDFNELRSVRLHSYRNLKRVTLSDNQIERLPSISFDALNSLEELTITSNPLRYIEDHTFKSLTALKTLNLSANSLELLSAEVFAGLQRLLQLDLNSNDLKELTHLQFNYLVKLQYLDVSGNSIKSLNSSHFSGLTSLRKLFIYENDLKEITANAFANLPALDTLDLSHNSLEFVDMDVFGSNTMPRLRKLMLKNNNLKNLHPLAFSSLPFLQYLSLGNNELTSLDVRMFAPLRRLEKLHLGNNLLEIISPDVLEAFDNVSELLLDNNRLSFLPNSNATFRNLKKVSLEGNPWECPCWDEMTQWLEDRKVLYVRPHSPYFKGQKPLCVVIPVNFCVKDINLVREHKIEEIFQNA*

>Woma_00011145

MNYPPKLVPPLSISRALAALLILPCLVCALPTVAQSLQGNLIHNIDKSYFQPPALEETIHEVQKILNRDPSLPRLTRGEIKELYEKVTREEYEKSVKAGDKVRADSMRALMLVLPYNTDNNTEENLQELFTRPPVTKVVDSYSSHQPIQFLTPSMNSEAKTGLKAVGQLDVTTYKPSFRMKTQTQPLNPTTYHPSNPPSGFYRVTPTVIDPPNLVPFKPIASDLPLNYDFNPKPVQRFSSHYNAKNAEFLKLGNEKKKYQTSSISTVKPVADVLESLGIVARHTQTRSRLGIDDYYAAESAPKPSIVSIADLKGLGTQSLGPQINADAYSMFKPLNIGEEIRVKPEVEGYLTRFGIVGGRKRKAIKKTVSSSTQNSLEPLSQESEIATAKVPSSHHVTAKKGATNVGNTELEKLLENLKELERLNVNSKAVVTTSRPTTTSTTSTTTTTTTTPAPTTAAPPSLITHGEPMLVEPKKPRRRIDPNIDINFANQGNHQTESNPADQLTKLLENLQELEKLKINPENVLKTTPPLNKGSNEAASRNVAVVTPAPIQSSALANRFSAQQPLTQSQQQLLQFQQQAQAGTTSPDFDQLQKVLQQLQVYEKANAKTSTTTTSTTTKRPVNLQAQVGPTDLWQLRKLLGDDKELEKLFEQAKARQQQQAKPPTTKTTSTTTTTTSTTKKPAATMADLLQLQRYLAELEKATSRITTTPKTTTTSTTSTTTTTTTTTTTTPRPAVLNPYSFPIDDYDARAKKQTVENSELQALINRVQQLERLNSQTRPTDTPLSPLLGFTTRNVKTPPPKIQDIRLPVNDFANLRTLYRQVEEAERVTQGDIEISTKATAIKAYQEQQLETATRKAQNLAQRIIDITNDTGSGSSLQEVDPNEFVQLQKLLSKVQELERVRINVPTPNPRSKETTKSYYLDAAASQRTQNVQNVNGAHIVYASKVETPVEAKNTKAEDLKLELPAYSKSSSDTTLPKPISTKDLTSSEELKDYVYASQPAVSEQEAYKNLQPPMIYKTITKEPPRFVATKSPKETKPQAKSAENLEEIQKLLNNAQELKKLGVALPKELTQRLETKFTPTDDNSYNSFKKVPLTVESNGKTHKKEAASLDEYLQRFGLTDYDNVKSQTESRKAVAFTPTTASWEEEEEEREKTAVFSLTTMKSLTPRLPKTTTESTVELPVYSATKIIEAAIKRAANRIGVATEGPKGFHRREDMNIDETQEEMEFGVAKDSDLMADFSEINYKLALPEDDQPLSYTKLKELEGNYSEKVYQTALPETKNKEEIPQVDLTANVDELQRLIKNLQELKNLNLSPDDKALLRKADLKYLENLKKQQTEADDTIKIRRQSNQPTEGTTTNTQKTTEASTTESSSRIQLNLNLDDGDVNTESSTTTAPVTSSTTEESLNGSLADLEDSFGPNPITESPPPPKKKNGFYFLADWNSFLEVGDGDDQVVVRLSPKIGDPRLFLPVKIP*

>Woma_00011149

MCLQDILLFSTTICFMLTVVIRASDIKVSCKNENGDNVDWFYLYKLPARYTSKDVSSQHNNLGLNYMFLTNTSLNEWTLSKQKINESTSMAALTLDPLYKDPLVLLLAYNDEFPDGQVKMTGGHTKGVIATDGSTAIWLIHSVPKFPSIPKYEYPKTGALYGQSFLCITLNVTEMENIGQQLLYNEPNIYYQRVPLTLSGQFPDVEQAIEKQWIKSEPFNNVLDLKTLQGVTFKSFAKSSKYHKELYEDFVAPVLDTNLMVETWRNGAGNLRSNCSLNDNVYNIESLRLENPLLQFNTSQDHSKWAVSQSVGLRFWRWRIPSTSNWICVGDINRQKHQLERGGGCLCQRHKKVAKLYRKLIERYETCK*

>Woma_00011137

MFYNLCGGLVAVLFFFLVEVPAGHSPYSVEIDNVEIIKEYDQKFIDWDTFGIRQKGRNEFVVSGNIILNLNLADEQKIVIQVFTYDKDRKVKGPMVFNLEKEVCKFAAEDKDLYPGIVEKSNLPEPGTCPIPKNDYKIDDYELSADFLPDETPKGDYFLVFLLKDGLKPVAGLSVVITLSG*

>Woma_00011134

MWSKIIVILAICSALSCDKGQAYPQSNSQGQGSGPCGPPPSGPPPSGPPPSGMPPRNGKGPCGPPPNGSRPSGPPPTTSTASSG*

>Woma_00011135

MQKFWFISCLALICFGVVLCQPFLTNADFHHFDKAGHRGGAGRNGRGGGCRNGTSTTTAADTTTTTTTAATTT*

>Woma_00011136

MLLKLIYAFLTIILFLGALNSLPVPEWSEQDYMDNLQNFFKHFQEQINEFEATNDYDFAIQEEMEL*

>Woma_00011177

MLNSHFQLAVAIVGFSSILFNCVNSISTAKPITEQLLHCDSVNWSNIQRQYELYIDAQQEPCDDFWEYACGDWRAPARLRFARPTDTLTAVKASNKYLLLKYFDDVEYKGLKVNDSFNSLTTFYKSCLDKRAFKSKVQDEQSIRVYMDILQNFSSFWPVLQRNFSSQKLNQAFNWQSVAAEMRRYGAQAMFSTKIQPNWQNSQQLIFYIMPPSFELLKSRQADTSFDAEAVFLYQRYVKLLMLDLGVRVRKANQIAEEIVAFEKSLMSLVHNDRSVVLKEPQTLRSLAKEMPDIDFLKYFNTLLQDFVLPEHYEDIMLIVADHNYLKALAKFLKTASNEIVAKYFLVQFLAHFEVNLHDDYSFIKQKEDCLMQLNDFMPSELSYLFLELRHGNAEEFLELSQQHLTKIFDNLKQQFEKLLNTTTVFERDLATKILSKEKLKAMRLLLPTLELRSQEDMPYEIGDNYDVNLINLSKLKTSKELKKTIAHVVKDVSSISMDKELSLYSLYREQSYGPLDVNAYYRLKKNAIELPIGLLQAPLYDPCLKSARIYGGLAYILAHELLHGFDYDGLNYDKSGNLANGWGVKAIIKFGVKSNCYLNERYNNGHVAINENIADSEGLRLALETFLESEMEEAFDQEDIKLFFLSFAQTWCGNNANDSKRITLNLHASHKERVNNVLGNFMEFADVYKCKPGTTMHPEEKCRIW*

>Woma_00011179

MLLKFSKLTTLLLLFILCVIQCEISAAYKTEIEITPAPPEEPTTKKSGFFGGFKKFFGGSSKTTTVEPLLDVTTAKPITPVPQQNSPAADSRPKPPPLAIPNAPLVPIGPRPDTPSSSPFGVGPPQWPQPNPSSTSTTPTNSFGIKPPGSSQLPQPSTSTSGGVKPSGSPQLPQPSTSANGGGVKPPGSPQLPQPSSATGGVMSATSTSTLKPSQQQNTFNVQNNLPRPAQPNLPPGFGPYQPPKSQPSGLDLSYGGGFGSGGRPQQQSSKPAFGLASMTSTTTTTTTSTTTTTTARPSQQLDFDLRGDFDAKKPASPQTGKEDFPTLPAPRRPSIKEDFPALPVPKSPTPTPSTPNSPSSPSAWNKPLPTPANVGSGTTTQRTLPASGPAAGSGSGSGGVSFVPHSQTTTTVRPGFEKQGNTLATDDEIRSLTETLYSKEVNSQLNSITINPQGKTRSIDSTDEAPQPFLNIDPKVFDTPTIAKMHLLFNNYEEDTMVNEHVTAMERKEENDFLDAVMATNVMRQAMLFLQNKGIVSPDPKTHRDLVKELWFTQYSRGNGKIGSSGFEHVFVNEVKNGTIIGLHNWEYFSDEEKAGRLDYKGYLDQLDLGTKGKVFKVRFTHNGLNKPVNTLFVGTSPELELALYTVCFKLRPDRTCPVSLGNSKFGVVTYSWRYRGKSLIGSAYPEI*

>Woma_00011147

MPKYSSILLASVVCSSAVGNQAKNIKCVDLLLMLAGGGRDSDCWVGGGGGGGGGGGGGNNVGLPGYLKNYKHKHVINQILKQQRK*

>Woma_00011196

MKYFVYVATVTVCLSFVDAGRPSTKYLPPNFAAVGAATAGSSQYPHYHGVNTGTAHGAVAGISPVHTSAQYAHNRAAAQIPILRSDYSNDGSGNYNFGFETGNGIKRNEAGHFQGSWPSSSLNVAGSYSYTGDDGKLYSVKYKADGNGFRAEGDHLPTPPPIPKEIQETLHLHQHNTRNQNSAGHYTAPSGAGVHHSHGGNFAAASHHSGSTVQAPNANYLPPQLGEGYNYGRHN*

>Woma_00011065

MQFNMKLTVVLLICSLSLAKGNSILNDTGGLNVSTTTLPRNIEVTFDLYTSLNPTTPQELRTNDINVLVSSNFNQRGISRFITDGLIIASSVDIFYDTPSTLLEAGAANVFIVKPKIHDINQNYDEASRYVKAVGQLLAEFIDFLHKEGGMSFSSLGLLGQNLGADISGYAGKHVTGGVINSICAIDPSNYKNCKISLCSTDAENVKICHTNVEHLLEHIGNITHPCYGVGLPINCVSPTV*

>Woma_00011129

MNFLLFLSFVIATLTVVIGLTVLESPTNVESESIGLLSDVTGGEIVLETCEIAAVGEFTFHVLIKFETIYGILFCSGAVIDEQWVITSAHCAEGAVEAFIFVGVVTVYSDDNYHSKAGRIIQHESYNRLTHEHDIALIYLEKAITPNERVSPALIPTTELEITYNFAITFWIPVYNDAHRLFSRQVSAARKYPCSTASVAIDGVICYDTNFLQKQCDGDPADIASYKDGLNQYVSCIKSFSPVPGVVVYTKISPHLKWIQTITGKTF*

>Woma_00011128

MKFLLFLSFVIATLTVVWGLSGLESELTGVISETNEGDIIFEDCQTAYLGQFRYHLAIRFVTSNGVLTCSAIMIAPNWAITAAHCADGATEAFLYAGTIAVDPDDYQHYPVKREDIYVHPKYNSQTYENDIALVYFKIAITSNDRVAPALLRKTELFDNCVLYRTFLVPNDYGILYQTYSVIPVSRKTVCGTANVYFDNVNCFNLNFLQGKCLGDPSYIVACKDGDYYYARGIFSFSPVPGVAVHTKIPAYYDWIQATTGMAL*

>Woma_00011127

MKSVCLIAFILVTVYAVHVATFCDIDSDGKPECLETSVKRAFRNFWDPAHYWYCANANEEPQLLRCPSSTLFDDSTNECIPWRHWEWINPCPDNDE*

>Woma_00011089

MNFRILPVLFLVHLTIALEYSQLIDPFVISNNPCETIRNGQFICKDCTTLAFCTQQGGSWTTVEISQCDTNHNMYCDEETRGCVFQQSCKLPNRGPKFECQNAGIFPDPYDCKYYHVCNNNNQGERLVCPRGTAYSPASKTCSLPSNSDICYEPQYNCTRLGEMSAWPTDPSIFYVCHVNGVGSQQVRYPLLYSCSSGYVFVNNKCQLYTSPTLAPPPAPTAVPEIKTCKLGSGLTMNPYDCYSYFVCVDGLLVSTNCPTGTYYNDKTKSCTFGKC*

>Woma_00011064

MKLTVVLLICSLSLAKGNSILNATGGLNVSTITLPHNIEVTFDLYTSLNPTTPQELRTNDINALVNSNFNQRGISRFIAHGLMIAYSVELFDDTPSTLLEAGAANVFIVKPKIRDINQNYDEASRYVKAVGQLLAEFIDFLHKEGGMSFSSLGLLGQNLGADISGYAGKHVTGGVINSICAIDPSDYKNCKISLCSTDAENVKICHTNVEHLLEHIGNITHPCYGVGLPLNCLSPTV*

>Woma_00011092

MWRRHVSNRVMQECLITTIVLAIAISLVRLPKCQAQDTKYGQYVPDKTCAESANLIQWTGGQFDFPSTSTKSLFKSSGKFIPKNIIATRAQIINDTVFLALPRYKKGVPATLVKTTIQPGTCSTTFKPYPCWDLQEEGNCKAIQSVVDLVVDQNEIIWVLDTGIVNTLETPVRKCPPKVLAISAKTGKILKTIDLDALTSPNSRLQYLAIDYASDNSCFVYVSDAANRAIIVFNIQADRGFRIILPKAVTTGSRSRDVLYIALIRRDCGSTELYFTYLSTKKLFSLKTEYLRSGVANGKIIDLGSKPSRMVIIGTDNGSAIFFRNEGDAEVYRWDTNTPFVETNFKPVYRSPTCQLATHAVPDYKRNTMRVLQSNFPDFMQNRIGCGAIQQLTMMQGCW*

>Woma_00011091

MYFKVLSLLLVVTYNIVTPCWAYDPPDTMCNKSVIANLTFQISGSSLLWPCESTKNIYIQSGRYVPRNVIVTRAQLLRDIAYVALPRYKQGVPFTLGKINLKKGKCLTKIAPYPCWAIQEEGNCQALQSVVDIAVDHNGLVWALDVGIVNTLEQPIRRCNPKIVAINTANAKVVKSIDLSDLLTAESRLQFLVVDYGADNRPFVYVSDAGARSILVYDIIAAKSYRIVLPKATCPTNDVLYIALATQYDGTSTLFFTYLSSPRLYSIKGEYLRVGQGAGSIVDVGAKPYGKQMVLLGADGGTSLFFRYKGENDIYMWDTETCFKSTNLQEVQRGGDCRLSTQVLPGHKRFMWALESNFHDFISERTGCNGASIILHPVVGERDD*

>Woma_00011106

MSSNYLNACSLLILSIVSGVYSQCSWEFVRTTSDIKCNLRAINSTQPLNLQAAETFTRLEISCSSDVLFANELANNTFAGLQKLLELQLDSCKMHRLNANTFEGLISLKRLTVQTHNAISGSGKTLEIFPQTFYGLRELNELNLNDNNIRQLPEGVWCSIQFLKVLNLTSNRIRSVKSLGFSEKLCTVNGDSGGADLQVLDVSFNELSALFDVWGTSGLRRLQQLNLQHNNISFLASNALAGLSSLRILNLSYNHLESLPADAFAGNKELREIHLQGNKLYNLPKGLLHQLEQLLVLNLSGNQLISYHVNNNTFVGLIRLIVLNLANNAITRIGSKTFKDLYSLQILDMHNNSIGNIEDGAFLPLYNLHTLNLAQNSLHTLDNNIFNGLYVLTNLILNDNLISIVETQAFRNCSDVKELNLSSNQLAEVPDAVQGLSIILTN*

>Woma_00011102

MAALKLFNIMAVLAIFGQITSSQSQIVSSGICPGDVEVVSDFEISKYLGKWYEYAKYPTFFEADGTCTTVEYSLRADGKVAVKNSQINGTTGEAEVINGSATIVSNAKLCVVFPVSRAFSVSSNYWVLDTDYTSYSVVYSCKPLADNQHSTIVWILTRERLPSSATIETAKDVLVKNNISLVPLTLTRQDCSN*

>Woma_00011113

MANNNLQLRYGLLLILLLCSKNGLAVNYNTYANNQHHLLDDNQYTVAMMRNTNQNQQNPNAAFLNVARNVQDYTGYQQQLRSYYYTPPPTPLPEYQEQSQQHHPSTIQSGSYQPTIHLWPGSPIVVDDVVNQQLSSSTARANQAKATTVFYKQEQQKIPTNSSNTLLPALNRNSNTQTARHLNYNNNNNNNIVYHKDFRPQIDVIPDTEYMQQQQQDDRITLWYNRQQQQYQRQLQLQMVEEYRKQQQQLQLQQQQQQQQQFYDTNTNLSFNGIANDLRKDNTPADYRELFRRYLENNPQRIKPIY*

>Woma_00011090

MKCILVILLFPYMVLSVSAVSLTDSVLRIQKQPRETNTCEGRQSPGPICESCDLLSTCVKHSNGWVNIPVETCDTLNGFYCNARLASCSNTTGPCHPFSSEGNFPCTSHGVFPDPYDCQKYHMCYFVGPTLVSAKVDCGGDKAFSPATGQCSLNINHAVCHEKQFKCLNAGDVHAWPMSPNIFYICKATSNQDERVLYPTLYRCADGEIFDGYYCRAAKPNEQFPSNPPIKPSDPVTSAPSSGITPTTTAPGKPRVCSETGLFADTEDCHKYYYCSAISGNLRQLQCPMGTFFNEATLSCTFGDCEN*

>Woma_00007796

MKLTVAVVSICCVLLLNNGLAHSQPLDQDKMEARQSYANNDDYDDEEEEEQEEEDELQQALSEQNDVISPGGQGEGGFDVINKEDEDDNENEEGDYEEERFMKNNKFLMKIFNIL*

>Woma_00007795

MKELYCGIGFLLLMVIANCLAIPTVLVNSGNSKENSQVPAAAEGVTEKETLLQNSKELVNSIETKPAVRLPTLEQKPMEVLNESTRNIEIMDNTDKSLKSIADLVAENNKKEEDPKLKDSPKDVEEKEIDKQSEVWKDFENDFSLLEQRLPDLTAEEYNEINQDGAMPWNFLKSLDNRREINEVRPKPIQAVAPIYVTVPIVINTHSSLPITLSIGGQNVPLNSEGITVKEPAEQITATVKQPADNTTPTSTYNRLMNYDKAYIRHRPTNRHRSNFRKKIYARNSGHVVEAYEY*

>Woma_00007799

MRSLTIFLIGILCCNHIRGHLTREARNVPQIYYTQQGPPGPPGPPGLPGPVGNKGPRGPTGDVGPAGAIGPQGLPGPDGRDGAPGMQGPNGLRGRRGFPGARGPPGPQGPPGLMGPPGPPGT*

>Woma_00007798

MSLKFVFMICAVICIASTLEAASLGKANNSKAVQRKLDYEAVRVRNARQAYEEDDSGEDSDEDDDEDDAELQHQNAVRQQQAHEQSVDDSQEGSDEDDEDAVAGEEEDDYYGRRMRRDVAESVNTNEIEENAKPNNSVEKEEEEKQKPAAEENSSAPSSTTSTSTSSPNTSVLILIRDAIKKVTELPNTQQVATSAQQYFQLFEHFLQQTIEQVIGDDDDEDEAESTTIASEPANETEGEKETETATVKPQEESQSSDNESSVEHSHQAQPEKTTEQI*

>Woma_00007870

MFKLSLMVLLSLTIATPSFGIYHEDCRHAPIDSYIAVKNNCRAFIYCQDEETSFEDNCPEETYFDEELSECVIDKDNICPAEEENEESEEAGNENSITIESGPITTTILPTLSTTSAKHEFNVEPTPRPKCNRHSNAYYPHGQRCEYYFKCISGFLTISRCSFNYAWDYQKEMCVPFQMVNCYGKSKLGSKISLLKINY*

>Woma_00007821

MYRFLLSIILFINLSGWCCTYTIQPIEFELFDNKFRLSIPNDPDLKSVSFHVNINKEFLAFETGQLTGIASMLENQKWCYEFQRILKATDILYIWLAVQHNRLFRYKIDPISIEAFRTGKPYIITTSSTTSANSTDGATQNPITVGDIIKENKICYPTISELPKTYAGGPYCKNDLIFADNFEILNAQYWINEVRMPLTTDDAEFVLYNGTANVANGVLTIGAHLNSLRLKKDSIDLGPRCTSVKVQNECSLTPQGFLVLPPVIAGRLSTKNYFSFKYGRIEVRAKLPIGDWLFPLISLEPLTPLYGEHHYRSGEMRVAYARGNINLKWQNKNINGARLFGGVILNDKAELRHQFMNDTTLPNVDHFGDQFHTYSLTWLPDELILAVDGNEYGRINTNFKEQINEPIWSNGERNAPLDQVFYITLGLAAGGNGDFPDTDGKPWTNTDPRGSLTFFRNRANWFPTWSQPSLEVDYVRVYAV*

>Woma_00007823

MRCILIFTIIASVAITALATNPPRWDPNYIVKGTLYIPYAEIAEPFYAWYDKNTRRSRIDYYGGMVKTYQLANEHPYGTSLKLAPITTKTELNKETCLQLNGTSEDPIQIQSILPNAKDFELIGTETFLGFNCDKFRLEETIGQKKNVYTLWVRYKKSPHYPASRMPIPVRYEMRGYNTLLGSHYDHYYLDYDSYDHDDIPNEVFELDDNMECMAFPGPGKGHYATFNPMQEFVHPTTEHVEHAFDHFKKKHFIDYRTEQEHEYRKNIFRQNLRYINSKNRGKLSYTLAVNHLADKTDEELRARRGFKSSGVFNTGKPFPYKVEQYRDNIPDQYDWRLYGAVTPVKDQSVCGSCWSFGTIGHLEGAFFLKNGGNLVRLSQQALIDCSWQYGNNGCDGGEDFRAYQWIMEMGGVPTEEEYGPYLGQDGYCHAKNVSLVAPITGFVNVTSNDPQAFKIALLKHGPLSVAIDASPKSFSFYSHGVYYEPTCKNGLDELDHAVLAVGYGTINGEDYWLIKNSWSTYWGNDGYILMSARKNNCGVMTMPTYVEM*

>Woma_00007828

MRLLATLLILGFVSYSCVSARLQSSEESHADSDPESISLSEESVESADIRRKRETSISNKLSIDEEPGEDLPEGVEEPKAVVDDKPAVDGAGVDDKPTVDEILNGELVEEFGEPDVVGENDADILIHQFSSIARNCA*

>Woma_00007803

MSLKVLFILGFITICFALEQHEQRAFGALNRNTNAQAFYQSVPHCICTPGPRGNPGEQGPQGIKGARGDPGLKGSKGPPGLVGPSGLQGSNGSDGPPGLPGLPGSRGRRGFPGGRGAPGPMGVPGLPGPPGPPAPAAAENGP*

>Woma_00007802

MKLCVLGFSVFLALLSQTLQQGFGAGLGAGVGAGIGGGFFPGFGLGGCNCPPGPPGPPGSNGLPGPDGDPGEKGPIGDPGDVGPVGRPGDRGYDGRNGPTGPLGENGRRGPRGFIGAVGEVGPRGPPGPAGPKGPPAEANARTSLYDLEDFEYDVEDDEIMQ*

>Woma_00007801

MSQTVQIFAILLILCASARCRGTSFKVLKQQHARTNDNVDNPGQVLFYSQNDHYQKKDKPKEPQIVEEDFEESEPDPEVEVKDGRKVPDYLYPTTIPMMNDNFKILIPQMSNYPLYGLENRLAAANEMRPLINNQYWNTQRLGLPNPNLQFGLNINANAAGMYGEHRPGKKPIGEKEDLNSHMLQPTAILPVIIAGPPGPPGPPGPPGPQGPMGRMGPQGKPGPQGPMGPPGPQGPPSPAANAYATLGQSHGLWYASNGKPIILQTSNNNNGNSNSNNYNNNDDE*

>Woma_00007800

MRSLTIFLIGILCCNHIRSDLTREARNVPQIYYTQQGPPGPPGPPGLPGPVGNKGPRGSTGDVGPAGAIGPQGLPGPDGRAGAPGMQGPNGLRGRRGFPGARGPPGPQGPPGLMGPPGPPGT*

>Woma_00007805

MKSKELTKRLLILMAMVCCCQQISAGLVIKTGKNKNSTNTNKDIQAATTKHPKLTYKAKTATTQKPEMEITTLLPLELDLDGRSAYINNEFVADPEEPVQDTSSPLYELLQQQMEKVMSNAAGKIEMYTDNSPQMKEAIDADAGDKLKPPQKPLFSYDLGQFDDYDEEDTSEYAGLEGVGGGEYIPENCEDTQESLGDNDDATANKDNIAPLIHNNQQNLQNNGYGQSSSTLDSSSQQQPIYSQLAQNNPNDSSNYEPQTQQATSSIYNSTPHTSSYGLKKPQLSNNKNHNKPKPQGSKQPKPSNPQHSNQNSYGQIKPTEQQVSLNNYAQPKPSENYGQSNNQNSFDKQPTNKQQSSSQKPQYASGNIQQVSFNNQKSTIKPVTQHSKPKPSYTSLNPKTQVSTSHYTLPVNTPNYANTSTNPYFVTNPLTTSSIAVNPSLISNYAYSHNSNTETSSSPFSSTLSQKRHLTFRTVASSTQYVSSPLADLMFKFSIGMAKPTNSNTNAINNYGGNQAQIFNVFNDMEPKTDMK*

>Woma_00007804

MRIYQLAIIIASLLLLTSTCDAKKRKHNRSSSAEEKTKTKTKWSSTTNLSPQTKNSMVATEEHGYYVKRTFLPLNAEGKNASLLQSPPFLAVPIAWFSCKDVECLKLNSLAINSNTNALSEGFLCDDDCTGPYEPICGKTPSEVAVFYNKCKLNVAKCRSHGYWSDLPYEDCQTQYPEEVKYAEMKFRRSPYFRKAGEVALKEEINMVEENKTEAKPEEITTTPTEQLLIQEFKDTEPVQKMEESKDSYKEIKTASGIVTEMKL*

>Woma_00007788

MQTLKSFLNILAVFVAFASAKRLSHQNEQRALGLVHHQQPTAAVLLPQSACVCQPGPPGPPGPPGLPGPQGSKGDKGPEGLPGRSGPTGPQGQRGSPGRSGIPGPIGPPGRRGRRGFPGERGPAGPPGPPGPIGPPGPPGAAARSLEDGVRLNLPDYDADVEPDVFADDGDDEDNEYDADDYRKKK*

>Woma_00007784

MYEDNYCNLPQATSTNSSISKSNKNNTSNGFLIESRTTNNRHSLSVIDFCITPKPASRSAGLPVYHQVAKIFIAVVYNIKKQKLIKNRILMTCAALQIYWQQMLVINSSIYQQHYKKL*

>Woma_00007787

MSLIVVLLLGCCFTLTLTQFQTQYGPPGPPGPPGPVGLSGPKGEVGDRGAEGRQGASGARGSNGRRGKTGLPGLMGPPGTPGLRGPRGYDGERGPAGPPGPLGPPGLPGPTAAAAQAVAGIRIGADADDNGDGRLRTNVIYTQQQDL*

>Woma_00007869

MISLNLLILTVLISILNCTFQASNAIRYVSHKNCNQVKKGTIVTNPGNCSQYIICNGLRSTLGECPDGQYFNVEMLSCDKNPIQCQQKLTTTNSVTSTTKTLVIATITTTTSPPPLMIAQSELLTTIAPLRPICSTLHLEHSVAHPQNCVFYYQCARGVLSLKRCPLGHGWDWKKQRCIPLAKAICFKS*

>Woma_00007970

MFLNNLLIILPVSLFLNGVETKNSFGQVDTLQKQTKNIDSNADVVVKSRYRRDSEDSSEIGWSIINEEVDTVIPLLLEHLQKLEIEHLQLPDIKENLSVKPLFLTYEAGLYLTNGIVYNVAGIQRYGPAYMTYEDKSFLIRFYLNIKSLQFEYNFLLKLMALEGFGKVIGTLEDTVIYAELAVDILTFKMKLYDFRVIQFRNIQVQLDQTRFIRNLTGVILSPITNLFKDRITTSISDGLKDQMQMVFDDFNNEDPLELRTFAKQILGGLTGQKN*

>Woma_00007936

MPPILILVIVLYNIFYEVNTAGVPNIKAFQKSTLFDENNITHMMQNEFLNNFNYKFEALNTIIRKMDTSIQDLQDKSHTWAIFHHHINSWNEGIRVLENKLDILKRTNEEQQQQLQKLELIITTNRNVKIPNECDDFSKDEVKQFLNGERKVTNEILNKINSILRHLERTTTWGNDKKPQRIANIMAPVGGVTETSKNVCGNISHRVEQRLNVISQQMAQQKDIKQLNSLERRNSKNLENINQIFSDQLDKQDEITTRLQKSNECCYSLSSELTTFTESSDILLKRIEKLVRNVSEKLNDIEVSTNNNNDSENSENELDKDYASEDLDVKENKAKDMEEISTDNSIEKDATTSLSYDYDNSPDVELIRPNLNGCHELVDEIMADTSEDARIDGIYKFSAPELNEAERDFFERYCVFPSSPNDTGLPWTLIQRRGPYEPHENFNRSWSEYRDGFGHLSRDFWFGNEFIHRLVYHDDYELRIELEDFNDTLKWAEYSIFRLDSEKYNYNLLIGEYRGNAGDAMKYHNDNDFSTYDRRNDKTVDACCSCALGYASGWWFDNCSEANLNGIYHYNPFGHNYIGIMWELWLGDYSLKKTRMMIRPRNVNVQPTYIHSQDYNDYTEDP*

>Woma_00007961

MLNKNINKAYYLLYIVFLLPTFVLSKNVSTNNKTFILNKISFTSPVTFDNEINAVNENNSTIHITKNRKYTLAETLRRTFIEFEELFENIVEIIAKNVPKITRDTEVFLAKLTNHTMDALNTLSNKILLEDDDGIFSEMFLNMERQLVSVKNEFENLAISVDEHFNWAVTQLNQTLMNNTEEYDWWTAHIKKRLRSVNDTKIYQEGCHVIDEFIQYSTKELHNCCKVTMTPIRSLCKSANSLITEALHIVVDVIDRMQACLEEKNSYLNYLLPCIHLAYDEMSNLISRAAQMTHLVNNMLPMKIFYTKSCFAIVRVDMKVRRNSIESSLFPNTYI*

>Woma_00007962

MFKTNWQANFIIFVFSIVFIQSSEEYNVDNSIRPPIRLGAPAHVINGEHISLEFYAALESGDLAKNNLQSSLHNFFENLQRNLSTSSILNSEVVYKPSDIEINRYQLGKLNDLADYFQYSSESAGQFAHKAEHFFEISKNESYFLLLKLRQVPSAQRFLPTAMKMRLTDYFAEMEFFHVMFSEIIDEALEYIVDTLRSIQRTFLTYTDIQREVLRTWNFKSNEWCCNMYMDFLQQWSAHIFKCSAGTNLNIAYDVYATSETFTKHIMRQLEFRIQRLYNCFIFNNYEIRCQFLRNPEHDFEKLFAKLDELQQYFDIKIKKGRVNINRIRRRRREQEFHDKCIPYGFPDIQMTSTLKTCFNFPS*

>Woma_00007968

MADRLTQLQDTVNLQAEHFCNAIGIIQQTSYPSKFPNFDRTGSQTPLQNQQQEDYAQLFAQLISRCAKDIDTLIESLPNEDSSTELQNQSLKRLELENQEAAERLEDIAIKGEVLLGKIQSALEDIAQAQLDMQITMKNNKS*

>Woma_00007907

MCQPNPTERNESQEILNTNESTLIGPINENFETEEAIFPLNQEVANRLEHLEHYESPNVEEQDERTNFSGENITNIDYDFSHPYWPEPHITNEMAVIPNNRPSYVTSRSRSRSRNRRQNRRHSREMKRLQEHKQMHQHFRSIFKLLRKTARSVKNVKRLLNQHMEIVKSLQASQNHRIITN*

>Woma_00007902

MKLYLLVLIAAVTVNCLVTAINGETSSDTLAVPYTDMLLPPFEHEAESSDADSNVKDKVKTVSTKPTTITTTKKPITNDADNNLPELSIEALLPPLFEARIKEDETKETTIKKTTTTTTTTTTKRPTTTRAATTRAPTTTTTTLQATKIEVPDLLLEPFLPPLLDNEILTKDEVLTTTTQTALTKTTKESYYQQQQQQQTKHLTADSKKYLKNFNVKISADKQVSESKTQTKQVSLLNKKLPSNIESLAKQHAAKQVAYQQRRVTNSNSSLNNDKQYNYSFNLFLGSTTPRPAKSGLPTITPFPRRLRR*

>Woma_00008025

MQKYIILTVIFALQWQLGAYAATPKQIATTDCPNCVDEMNYMNKWTMPLLKLGEKRYYLGIFFKANWFKATQYCRYHGMHLASVSSQEENDRLEKHIRDFGLGHEHFWTSGTDLADEGNFFWMSTGRPITYTNWNAGEPNNFRYENGEEENCLELWNRDGKGLKWNDSPCSFETYFVCEVQPN*

>Woma_00008027

MKSFTILLIIFLTAVSVDSNGNDSKNYNNTSSSNISSRNDSNNNTSSGINNIHSNSFTKNSFRTNFNNFNFSSIGHDSASSTFTVFRDKRGTDFALDPSEIARQNQITQQILSNYLERHLFPHRSGNQKIPTIAEILPKPKPPTARARPRGFASSSASFKKGAAKQQQQQRKRYGNGKKTRNSLYRRYKPATTTTTIAPTYDYDYEEEEVVNKKQKNYNTSRKRQNSYSNSNSSIGDDYNDHLHNEEVEDDGIEVEDVEEEPVVNNNDDEQQQQSAEIELEHENHRQQQHSRLHRHRQLLRHHFRFLEDIDDPELDNQESLQSVESEQHDHYYSSNGKSGDIFSRDPSENEIDSGKCVWE*

>Woma_00008068

MVDFTVANPDIGTGSSQQRPEEQNGLWEELSLKLNSTGPPIRDVPQWIKFGGTIKPTQRKEW*

>Woma_00007991

MKHSFYQFSIFILFYVNGILSDCNVCQSSNYVACHKENVYSLCINGIATKDYITCPENHVCTDGPYVCYPLPEAKPSCAPKTNLCGVCSENQVYACVNETTIAFCYGQNEPLDGADVSYCLGDTVCDIYSTKGFCTESYLTKPSCSTQSDDDGDDDDSRFTTITTESTTSTEPTTITTTTEPTTATPTTEPTTTTTTTTASTKPRDPNTICKDADTTGSFYADDPTCTSFVVCYIMSGSIYGLKKFCPSGQYFPPGAEKCAADKPDSCI*

>Woma_00007993

MFNKFIKTITILLLSVQISVTSLVGECNVCFAQSKIACRNETHFSTCFEGLPTTTITKCPGTRVCTDDVLICRSTEEGFTPSCYHDYCGDCSVSNGIFTCLDETTYGYCFGNITPLPNTLRSCPQGFVCNYNSQEVCVPDDTNQPSCKSKTTTTDPSTTYTTDISTISTESTSNSTTTEMTTETTTTTSTTTEEETESTTDSTTRTTTTQMLTTETSTQTPTTETTTTTEVQATETSTTTEIPTTQTTMTTETTSTEKPTPTTTEISTTETSTTAEIPTTETTTQTKTTTAAPRDPRTICGEIDQQGYFKSDDPTCREYVYCYILSGQYLGWFYYCDGYFNAETGKCQKERPLDCVAYEY*

>Woma_00007992

MMKLISILCLLLIVVHQSHANCQDIDRLCREHFNVTMPYECDRTCTKFVVCFYMLDNIRGILKTCPNGMYYDSSHKMCSKYKPEGCL*

>Woma_00008044

MKLQLAVLLVATVMLMGCSTCLAQGPPGGGGGGGGQGGGQGGGGAGAGGGAGGQAGMGFGMSAGAGAGGGAGGQGGAGGGGGR*

>Woma_00008043

MRFQLTLLLVAAVMLMGCSTCLAQGPPGGGGGGGGQGGGQGGGGAGGGAGGQAGMGFGMSAGAGGGAGGQGGAGGGGGH*

>Woma_00008051

MSSKTIFGILIIPILFLMPQISAQTNSKEDICDFVNTVNLTNREKLEDGSYRYDDIIIPPEKQAFYDYQLRFLGMRETVQLHLRGCACEKRPCIKLCCPRNEFFNFSADKCEKMNSGMKTNWNVEMLSRTGTRKTVNLLEKFTTQIGLPCDLLEPFDYKLDVWILKEVSCAV*

>Woma_00008013

MFQQNIYLTCLALICLSSYAWASPYQANPEYQVVYPAPEYQQDTANAAATAITDIAAAKASAAKIDNINRAALDQYGYQIGVPLLVKPYGSLTNLYAALAPKRSFVGTIDAGFFKDSYGKIKFADETAIGAIVI*

>Woma_00008015

MNKSIIFAVIALTAVVCCLAKPGYWPSSVAYSSPIYRSSAAAAAASSVARPSYLYQVPSYAIDVGHPGYAVYSPSYGVSYPGYGYY*

>Woma_00008014

MNKFIFITVAALALVTSVRCGSYGGGCGSSVGSAYNVNSYSLGAGNAASAYASPAYAPPAYSPPASPAYSAPKYSVQPLYAHELYPGQQNYKSYHSAPTYSQVALPIVPSAPVAKLYVPAQQSYAAPAGSYSSAY*

>Woma_00008016

MLPKVLIFCALIVAAKAGYSIPSDGSQKSCAKPSAAGYAAQIQPAVTLTVLPAYGSSSASAYGSAPAYGSASAYQVSQASQSYANSAPSYQASKASQAYSSSPSADIDAEILNLLKLVFALPSAGGPDAALINVPQYPATVYTAPAVELSSDNYSNAISSSSAAAAAANVKSASSGYGPAPATASVQYAGSGYGSVPVASQSAY*

>Woma_00008033

MQYFYLLLLFLVISLNLCMAQDKWVWSKAKKNDKVRGYYPAADKRLYYTEEQYARADREREPTTKRPIPGQPQNDEIDDYNDANDEPTKQNSGGVGFPPGNTANGILIGPGGPTGIIGRPPTQYPAGVPPPAFPNFGQGSYPGFEVGQNYPGGIINSNAGYPNYNGFGQYPVQQPSAFPNTQQFAGSQYPQAQYPAAQFPQQPQYTEGYGLANLNPVFNANPSFSGNPAYNNNFNPNYGFGFDEYPAQLEGKSAQVKAIDKKVDDKVAKNLKKL*

>Woma_00007981

MKFLLFLSFVIATLTVVWGLSGLESELTGVISETNEGDIIFEDCQTAYLGQFRYHLAIRFVTSNGVLTCSAIMIAPNWAVTAAHCADGATEAFMYAGTIAVDPNDNQHYPVEQEDIYVHPKYNSQTYENDIALIHFKTAITSNDRVAPALLRKTELFDNCVLYRTFLVPNDYGILYQTYSVIPVSRKTVCGTANVYFDNVNCFNLNFLQGKCLGDPSYIVACKDGDYYYARGIFSFSPVPGVAVHTKIPAYYDWIQATTGMAL*

>Woma_00009121

MKLICLLLLYAVWIEAAPAGYPRTRPSAPAPKPKPPRPPPKPGNGNGPSTNYLPPNGGGRGGNGGGGNTYLPPGGGGGGGGNGGGGNTYLPPGGGGGGGGGNGGGGGGNGGGGGGGGGPVIPIIKMESKISIDGSYMYEYETGNGINVDENGYLKNPGGDEEAQVAEGSFSFTSPEGDTFVVTYIADENGFQPTGDHLPTPPPIPIEIQEALDKIAAGGGHPDDGSGGGGGGGGNGGGGGGGNGGYVY*

>Woma_00009108

MKFVVVFAALFAVALAAPHPEDAVVLRSESEVGPESYNYAYETSDGNKAEERGQLKNIGKEDEAIVVQGSYSFVADDGQQYTVNYVADENGFQPQGAHLPVAPVA*

>Woma_00009109

MKFVIVFAALFAVALAAPPHHEEATVLRSESEVGPESFNYAYATSDGTEAQAQGQLKNIGTEDEAIVVKGSFSFVADDGQTYTVNYVADENGFQPQGAHLPVAPEA*

>Woma_00009115

MKFIIVFAALFAVALAAPPHHEDAVVLKSESEVGPESFQYAYATSDGSEAQAQGQLKNIGTEDEAIVVKGSFSFVADDGQQYTVNYVADENGFQPQGAHLPVAPQA*

>Woma_00009114

MKLIIVFAALFAIALAAPEVEIVRRDSDVQPDGFKFSSETSDGNKHDAQGQLQNIGSDAEELVVRGSYSFVGDDGVTYTVNYVADKNGFQPQGAHLPVAPAA*

>Woma_00009111

MKFIIVFAALFAVALAAPRPEDAVVLRSESEVGPESYNYAYETSDGNKAEERGQLKNIGKEDEAIVVQGSYSFVADDGQQYTVNYVADENGFQPQGAHLPVAPQA*

>Woma_00009099

MHYMFKYSLCSIIVVLCCLQEYCLSQSSTVRPYKFGFTIDQQQHRSEQRDERGIVMGEFGFITADGIYHVTVYATDEQGRFRILAMKSYPYESPPKTVEVMVKPKIPIPTKTTPKPLTRHNFNTEACSGCFLKNGNNKPKDVNGIQEKTMNNGIRPLSKPLASESNAPASPKLPNQSSSASKPTAGGTSLIPASKTATGSSNPPIMDIIMQKVLPTLMGSKGSTPSIGNAKPNVVPSAKKSDSSNSGIDSRRVAGGSAKGDGDLYRFKYILDYHGHSETGKRNGDKEGTYYAIGDDNVERTIEYVANEFGYQPRIRWRKLDANEIKSNENTLKDYEFVWFN*

>Woma_00009100

MKFVIVFAALFAVALAAPPHHEDAVVLKSESEVGPESFQYAYATSDGVEAQAQGQLKNVGTEEEAIVVKGSFSFVADDGQTYTVNYVADENGYQPQGAHLPVAPVA*

>Woma_00009101

MKFIIVFAALFAVALAAPRPEEATVLKFDSDVGPESFQYVYETSDGTQAQAQGQLKNVGTENEAISVQGSFSFVADDGQTYTVNYVADENGFQPQGAHLPVAPVA*

>Woma_00009102

MKYQIVVLACLVALALAAPQQQDDSHAETLRLETENNGVDKYNFAYDTSNGISRSEEGVLKTTEDGAAIVVQGSTSWTAPDGKKYEMTFTADEFGYHPSFKLVA*

>Woma_00009103

MKYRVKNVFITLYCLSIIIFYIDISTAAPLEKVDSTEQPKQLQQQQQDSVKIVKYENINIGTDGYDFAFETSDGVSRKETATVKNIGTEEEAISVEGSVSWVGPDGVHYTLNYLADENGFQPKGEHLPLPPDV*

>Woma_00009106

MKYIIVFAALFAVALAAPPHHEDVVVLKSESEVGPESFQYAYATSDGSEAQAQGQLKNIGTEDEAIVVKGSYSFVADDGQQYTVNYVADENGFQPQGAHLPVAPVA*

>Woma_00009107

MKFVIVFAALFAVALAAPRPEDAVILRSESEVGPESYNYAYETSDGNKAEERGQLKNIGKEDEAIVVQGSYSFVADDGQQYTVNYVADENGFQPQGAHLPVAPVA*

>Woma_00009076

MEVKMNFSSFFKILLVILTFLLLKVRTARNNTKHNVVFIVFDDLRPTIGAYGDRLAQTPYLDAFINGSYFFTRAYSQQALCAPSRNSFLTSRRPDTLHLYDFYSYWRTFSGNFSTLPQYFKSHGYYTYAAGKIFHPGVSSNNTDDYPLSWSEKPFRPKTERFMNSPVCPDKLGILRKNLICPIHLQTQPYKTLPDIESVSEAIRFVENRKKSRRPYFFALGFHKPHINFRFPRQFLDRFSLKEFYNYTSDTYKPHEMPNVAWNPYTDVRSRDDFKYMNISFPYGPISEFQSAQIRQAYYASVAYVDDLFGKFISHIDLDRTVIVITSDHGWSLGEHAEWAKYSNFEVALRVPLIIRSPEFPLHEGGKRINVITELVDIFPTLIDLNHLPTVPKCNYKRPMSEQLLCTEGKSLYPLINGIGLGQEYFALSQYPRPGMLPTKHPNSDKPKLKNIQIMGYSVRTNHYRYTLWVRFYVQNFSRDWSNVFGEEMYDHRLDASEEINLVNWPEFDDIRLWLRDKLIKSFSK*

>Woma_00009012

MNTDKYCPCLADKAHSSKTTTSSTSTISTNGFSTSNQNNPSSFNSNENNFQQFASSGSFGGQTHFGSIVNSMALLQQIPVVPQVPGYPSFYPPDQIPKGAIVAFMPVIILPEAAYASCKESSDKFTSEHIQNPLGVQPATIPFGFSLHSLFPEGAKKDQCMCPCSCTQNIPNHLHAKREVENINEPASTLSVTDSTANKPSDGDEKNKLESKDIQTEKTTAVEYEKSATKIEDVKNV*

>Woma_00009013

MNFKIIAFNLMLLVCFSYSQSVAVNENNKNIKERTSDLLPLETNGKLLNLGSLCGYSNSGYYSNTNRLGSVFYPNKFPSLYPGGYYPNKNYYPSTSFGTNKLGGGYGGYGGNGGLRQYYGYLDPNYIGLRNRGFGYYTDTNFYGLA*

>Woma_00009011

MARAVFCMAIACILVQCAFSVKLDAENRRLILDIKDQKTTNNQYYSSNFDTNAYRYGYDIGKTGNFHHETRGPDGVTYGCYGHIDPYHVLRATHYVADAHGYRTVEPMKPVLTYPKPDDPVTGRNDGVLLQWNELYFPIGCGKFEGGVRPNIPLIYVEDNKSKDSVAPGYSKFPSLVIKGSDQKSPQIHRYGDNTHKLVSGGTGLFDTGKGHTFEGSPGVQSPSSHSVAALRPSENGKYKNEASGQYHHVPVPYEHVVGAQGGHGGFGGNGNGGGLGGNGGQGGFGFGGFSGSGSSIGPFGPHGPPGPNGPPGPNGPPGPNGPPGPNGPPGSPGSYGPSSPGPFGFKAPPGPPGPPGPTSPGNYGPPGPDSSGKPGLYVGGSSTGPYNNGKYTHKDGIVVSGFPGDVGYTEGNGKYVNHDDGRYKGIQDGKYYHDDSGKYVHIEGPTGPPAPPYVHIVGPNGGEGGFGGNGNGGGVGPGGPGGPKGPGGPKGPGGPRGPPGPPGPPGPPGPLGPTAPGPKGPKGPPGPPGPPGPNAPGPKGPYGPPGPPGPTAPGPKGPPGPQRPGPKGPFGPTAPGPYGPFGPDAPGPYGPPGPSRPGPPGPNRPGTEPPYPPKKPKGPRDSDLSVPGYLPPHQKNARGYYY*

>Woma_00009038

MMSRTVRYFKLIYIVAGILLSNEEICETVDQIQIATRVEMKEKINQQSMKKIQGPEPVQNSKGSLSKCWYRKFDVFDILNRLKGRIRPQWRTFSLWTIPNYLIERSTNLLYRKFIINSDCDVIYK*

>Woma_00009046

MKTFIALSLFVVIASAGVLSSVEQRLPLVPVMPLEELEGRITNGELAKPGQFPYQAGLSLVFGDKGAWCGGTLISDRWILTAAHCTDGADGVTVYLGATDIKNDKEKGQQRIYASKLNIIVHAEWDPNTLSNDISLIKLPVAVEFNNLIKPATLPKMDGKYSTYEGDLVWASGWGRESDSATSVSQLLRYIEAPVLKNRSCKIYYLGAITEKMICISTAGKKSTCNGDSGGPLVYKDGDINYLVGATSFGIALGCEKGWPGVFTRVTSYLDWIEQKSGVVNK*

>Woma_00009118

MKLMLVFGFLAFVACTLAAPQKDVEIISQNSDVGIDSYKFDFATSDGTSRNEEGVIKNAGTDNEVLEVKGSFTWTAPDGQTYTVNFIADENGFQPEGAHIPK*

>Woma_00009117

MKFIIVFAALFALAMANDPRNAETLHYESNVEPESYNYAVETSDGKSAQESGHIENLGSEDEAISVRGSYSYVGDDGVTYQVNYVADRNGFQPQGAHIPVA*

>Woma_00009116

MKFIVVFAALFALAMANDPRNAETLHYESNVEPESYNYAVETSDGKSAQESGHIENLGTDDEAISVSGSYSYVGDDGVTYQVNYVADRNGFQPQGAHIPVA*

>Woma_00009113

MKFFIVFAAVIALALANPVDIVKSESDVGPESYSFHLETSDGTVRHEDGQVKDVGSEHPAIVVHGTFSWKDEHDGKVYTVNYVADEHGFQPSGEHLPPLPKH*

>Woma_00009112

MKIAIIFAALFAVALAAPAQDYAHAEVLRLESDVRPEGYNFALETSDGKTHQEEGQLKNVGSEDEAIVVRGSFSFVGDDGQTYTVNYIADENGFQPEGAHLPNVPIGH*

>Woma_00009110

MKFIIVFAALFAVAFAAPRPEDAEILRQDSDVGPESYNYAYETSNGIKAEENGHLENPGAENEAIAVKGSFSFVADDGQQYTVNYIADENGFQPQGAHLPVAPQA*

>Woma_00009084

MFTTTTSNIMDSHNNNLYKDSEKIEENIERSATALRELTIQGTQNEASDDKLSCQNSTTKATGSVIEPHEVKGVRYGKCKWFNVAKGWGFITPNDGGQEVFVHQSVIQMSGFRSLGEKEDVEFECHLTKRGLEAIRVSGRQGTDCHGSTFRPRIKKRHRRVRCYNCGEFANHIASKCHLEPQPKRCHLCKQGDHLFANCPTKKANPTLQHSKSLPAIKDSNAQEGTLNETS*

>Woma_00009024

MLPPRIFKFVFLICLILVLILQATDSAQTKTRRRLRRPTSAPEEEISSSSVSRARSTRVSGKKSAESVAEKRIDQESSSSTTVTRTRVRSKSKLRSSGSSDASVSSGLIASGSSLKSKKTKADDGGKKIVCYYTNWSQYRPKIGKFVPEDIPADLCTHIIFAFGWLKKGKLSSYETNDETKDTVPGLYDRMMSLKKANPKLKVLLALGGWSFGTQKFKEMSATRYTRQTFIYSAIPFLRKRGFDGLDLDWEYPKGSDDKKNFVLLLKELREAFEAEAQELKRQRLLLSAAVPVGPDNVRGGYDVPAVASYLDFINLMAYDFHGKWERETGHNAPLYAPSTDSEWRKQLSVDNAANMWVKMGAPKEKLIIGMPTYGRSFTLANTDKHGPNAPATGGGREGVYTKESGFLAYYEICEMLLNGAVYVWDDEMKVPYLVDGDQWVGFDDERAIRNKMQWIKTNGFGGAMVWTVDMDDFKGEVCGGNVKYPLIGVMREELLGISRGKNAKDVNWSEIAATFEDIEEIEKPEPIKISVEEVLAKVRKPVKKHKIKSGLLAVEQNSRPAQVFCYLTSWSAKRPGAGKFEPSNIDPKLCTHVVYAFATLKDHKLAENSDEDPDNYEQVIALRDTNPDLQILLAIGGWAFGSTPFKELTSNVFRMNQFVYEAIDFLRDYKFNGLDVDWEYPRGSDDRSAYINLLRELRVAFEGEAKSSGLPRLLLTAAVPASFEAIAAGYDVPEISKYLDFINVMTYDFHGQWERTVGHNSPLFPLESATGYQKKLTVDYSAREWVKQGAPKEKLLIGMPTYGRSFELINETQFDIGAPASGGGKPGKFTNEAGFLSYYEVCSFLAADNTTLVWDSEQQVPFAYRENQWVGFDDERSLKTKMEWLKEQGFGGIMVWSIDMDDFSGRCGNGKYPLLNSLNDELKGYKVTLEYDGPYESRGPRGAYTTKDPHEVTCEEEDGHISYHKDWNDCTHYYMCEGERKHHMPCPANLVFNPQENVCDWPENVEGCHVPTEAPA*

>Woma_00009149

MKFLIVFAFALSCALAELYQRNVIVSVVENKGRITNGNTAYVGQFPFQVGLSLKLNDPLLASWCGGSLIGKEWVLTAAHCSEAKNTVKNDISLIRIPSVTYTTKIQPVKLPAIASSLSTYTDDYVIASGWGHISDSATGATNNLQWTRLQIIANTVCAVRARCEIGYPAAFNRLTSYLEWIKANTGTAY*

>Woma_00009142

MRSFVIIFSLALIGHSSVYGQQHQQIDPAYLRQYYQQLQQQTGAAQSGDATPIYEQNSEPTQQQYVSPGQQLRVKDNVQEQIRASQQQQQQQGYVAPVVREYLQHQPQQIQYQPQQVAYRQPQAAAPAPRRPPPQQQYQPQQYQAPRPKSKQQLEQEEEEYDDQNSSYQFGFDVKDDEFTNYQNRKEVRDGSVIKGSYSVVDSDGFIRTVKYTADPKEGFKAEVIREPTDIVVKIPTPSSQQQQQHLLRPHHAPVKAQEYNLKPQYPHQQYQH*

>Woma_00009104

MKFYAVLMFVALIGCALAAPPQDAAEVKILRFDSDVQPEGYKFAVETSDGKTHQEEGQLKDIGTENESIVVRGSYSYIGDDGQSYTVNYIADENGFQPEGDHLPRL*

>Woma_00009105

MKFIIVFAALFAVALAAPRPEDAVILRQDSDVGPESYNYAYETSNGIKAEENGHLENPGAENEAIAVKGSFTFVADDGQQYTVNYIADENGFQPQGAHLPVAPQA*

>Woma_00008381

MFFGFWQFKTTVSFLLLASCLGQDLLRTCDRNYCYGRSIERRDINSEHLREWNRVEKLNNDRERENEFRQGHIENRDDVIKEVRANRGEMSDLESRRLVNRRENQVNRRELDNITSNRKEYTRRESNRIRNENRLVIDNISAVTDEDRRSESSKMDLLAEDSHDNYRDINRSFDKTHRTQHVNERTVERRQNSGENERESRSVTSRRVSERDNQQDEKRESLRFIDRLENRIEKSDSKSRGLVSRRENCEIRREVDNLTERINRESYRIRNEHRFVIDDKSAMADEDRRSESSRMDLLAQNNHDDHRDVDRSFDETRKPERFRERTVERRQSLAENERDDRSATSRRFNERDNQQNENRESLRLNERLSETGQDSEQRKGREINTANRRLNERKTGEKLEDLQRIFEERTDKEEHCNHREHLTRTKTKNKHDNTNAIRIFSDRAPENERRFEHLRNDGSRRLSLKELDTLDNYERRLEERESSKRNRRFIKNLPEENNRQKRVIRLERNHESQRHNNHFTEVKGFSEERENVRRLSEQESERSRFNEDLNERNNRLRDINRESLNLNERHDRRLQKISHIRNENEQERGQRDRQETLRQLDNRDNRYDINRRLNERAQRESKNKQEREDSRVRISENDSEINQRELEDRREILKMNRQVNQPGIRRELNKRLEHPNENLAKSENILNGNHESQRELINLSIRQHENHEEQSNSREHLNGNREYRHIDERQDFLLDEDIESRHSHREYELIERRDENIRELDDRREKLRQLKIQDNKRVFDENQAPRNDRDGNQREHNQRSVRNNEILNYRDFYNMVEEYKEKPTLSLNKGYMAFGQGALLAYVIMKTLNFKNDTKYNIYKKLQEAVGLMGF*

>Woma_00008380

MFFRFWQFKTTVSLLLLASCLGQDLLRTCDRNYCYERSIEGRDINSENLREWNRGEKFNNGRKRENEFRQEHIENRDDVIKGVHENRVEMSDLEFRRLVNRRENQEIRHELDNRTPNRKEYTRRESNHIRNESRETVTDEDRSSESSKMDLLAEDSHDNYRDVDRSSDKTRRTQRVSERTVRRRQNLGENERESRSVTFRRVNERDNQQDEKRKSLRFSDRRENRIEILDFESRRLVSRRENREIHCEIDNLTANLKKRIHRESYGIRNENRVMMDDNSAMVDEGRRSESSRMDLLAKDNRDDHRDVDRSFDETHRTERIRERIVERRQNLAENERNYRSVTSRRFNEPDNQQNENQESLRLSDWLSETKQGLEQRRGREINTGNRKARRLNERMTGEKLEELHRIFEERIDEGEHCNHREDLTRTEVGKWDTLHNCERRLENNRQKRIIRLKRNHESQRANNRFTEVEGFSEERENLRRLSEQDNELSRLNEDLSERDNRLCDINGESLNLNERHDEKLQIISHIRNKKAQERGQRDRRETLRQLDYRNNRYDVGRRLNKKAQRQSEDKQEREDYRVRISGKDSQTKKRELKDRQQILKIDRQVTRRDPSRELNNRLERQNENLAMSEDILNGNHESQRLNNELSGSERERERINHSLRQYENHVSERQDILLDEDIASRRFQKKYELVKTRDENTRQLDDRREELRKLEKWDNNRAFNEKQASQNDQLNRHNDDELLHRDENQRQQKQRSRSVRNDENQNYREYRNMINKYKDKPILSLNKSYMAFGQGALSAYVIMKTLNGKNDIKCNIYQNLLEAFVLMGF*

>Woma_00008385

MLLKLSSFTFIVILAIILKCSNITNSQEILYVPRTKLLARWPFFGGFRNILRVVRPNVHVGRKSDYRSDFDENYLNYHGAQLWKIMFSQELTKNNVTRTEEMQKFIEKYGKCAEKKV*

>Woma_00008387

MLKSKRWNTLALLLHIQLLVALMNVAIAFDITKITPIADRNIRTGNKMLYEKEISENPSQFKRNGTTLSLEMAVADMEDYQKGQDEDVLDVNEIPVRYDEAQLWRIYNISDNMRRQMMPVADILENKYGGTVWKQNSKFLDVSITKDHLKAARSFLNAHNLMPEVLNINIQELIDMEQMVGVNLTQSEPGQRTKKASRSGIHWKDYHDLDVIYSFMREIRGKFPNICRLYTIGKTAEGRDLKVLRISENPRDYKKIWIDGGIHAREWISPATVTFILYQLMSKWAKQPDYIKTKTWYIMPVMNPDGYVYSRSVNRLWRKNRSPSKHSTCLGVDLNRNFDIGWNGYGSSNNPCSDTYRGTSPGSELETDAVVNFLSKRKYNLESYLTFHSYGQMMVYPWAYKAVKVKDAAALQRVANTAVQRIEEKTGSIYRASVTHEVLGIAGGGSDDWTRAALGTKYVYTIELRDRGTFGFVLPPSQILETAVEGYTIVNTVAQAIA*

>Woma_00008399

MKLSAAVVFALLGLAAADVSHLSGAYNYPKQDDSYHYSPPAQDEGYHYNPPAPVPQQTYLPPAQPQQTYLPPAQPQQTYLPPAQPQQTYLPPAQPQQTYLPPAQPQQSYLPPAQPQQTYLPPAQPQQTYLPPAQPQQNNLPPAEPQQTYLPPAQPQQTYLPPAQPQQTYLPPAENAGQDGYHYRVVKRYRYRSH*

>Woma_00008326

MRRSSFTPVFNISTQEPLIVVEETQTTDENEELQGAVGGAEGGSASGMGRTNSDDSDPDSPKNPYLLCPLPDMQQRRKHSLPSLQITEGITASQVRRLSEVGGETSGLSPHEVEFLATLSQKTNPGGGGRRHSVVTISAVPPTLFGRNRRESISGALYSGSRRGSGIQGPPLTEHRGSIHNLQLDIMDGIVQARKTRSGSGVWQAPILKETESNVPVQT*

>Woma_00008352

MNAFKTFAVILSIVGLSSAGYFYPAHGHHGHDGGVAVHYSVVTEHKADSHGYGGHGHNDGHATVHTWVNDDGHHGAYDDGHATVQAWGNAEHGHYAEHGIGYGHAHGYGHGHGHDDGHDHHAYPKYEFSYGVKDPKTGDFKSQKETRDGDKVEGSYTLKEADGTTRHVDYKADKHSGFNAVVHKLGHAHATGHDGHHGHGYAHGHGHGEASSFVTIKKHEEKKH*

>Woma_00008353

MNAFKIFAVILCIVGLSSAGLIYPEHGHHGHDGGVAVHYSVVTEHKADSHGFGGHGHDNGHATVHTWGNDDDHHGAHDDGHATVHTWGNDELGHHAEHGIGHDAVHYDGYYGHGHGYAHGHDDGHDHHAYAKYEFSYGVKDPKTGDFKSQKETRDGDKVEGSYTLKEADGTTRHVEYKADKHSGFNAVVHKLGHAHAAGHDEHHGHGYGHGYAQGHGHGEASSFINFKKHEEKKH*

>Woma_00008351

MNTFKTFAVILSIIGLSSAGHFYPAHGHHDHDGGVAVHYSVVTEHKADSHGYGGHGHNDGHATVHTWGNDDGHHGAYDDGHATVQAWGNAEHGHYAEHGIGYGHAHGYGHGHDDGHDHHAYPKYEFSYGVKDPKTGDFKSQKETRDGDKVEGSYTLKEADGTTRHVDYKADKHSGFNAVVHKLGHAHAAGHHEHHGHGYAHGHGHGAASSFVIVKKHEEKKH*

>Woma_00008324

MSLIRFIIFCALLVAVTDSKWTEVFGKIHKAINGSDNLMDSLVHLPDQILKANNKAVDQFDILQHKVSALKDKTEKALQENMKEVKKEWNKES*

>Woma_00008393

MMKYVVVLCVAAALMHDAQAFKIVTFQTGAQSSAPASSAPAVDPSALIQGVQSAIAGKLQQVSSLVGSLVQQKMALKQNALNSIQSSLGSFKSQASSVAGSVRPPFVIRKSIYVGAAAPAKPAATTESTTSVDVDSTTSSGGY*

>Woma_00008396

MKNQILWTMVVLLVLVKTISMEPQWYDAADAQRYLIDTEEKYNWYQAWNECARYDAQLLVIENEMEYENLKKFLKTLNDKNKYLWIGGNVLYEKISFYWAPTEQLFTFANWQTDDVGLSTLKKQCVYLMRHRKNFPWNFVTCDAKQYGFICEEMQYDTAESLDKAGDFKAVENENKGTMYNGSIYGLIG*

>Woma_00008394

MRILSICCTVLLITMCIIMQQQNVEAKPFIFKFFVPQAPARDASSSASSSTSSSSSSSSSITSGIDISSLISTKINLLSSFLQAGLTKSVSVGFNKLFPNTAITTTKRPYTSTTNTTPDTDFEPESSTAATTSRKTTTTTTTTKATTTTTSTTESSNFEKFTDSAIDSEGLEEKTTTTEKSVADNTFSKVTTEKTSSGFDIVTGKYEITYGSTEKLDNDDDDADSKVTTTSVGNNGGSDVNIDSKITTDNISKVFSRIGTDNIVASTNSVSAGNDSSSKVNIDDDKVSGYDYNTNSKVTATTDDKFQNALNIDNNKDRGTNAASSKVTTTSGNGYDYNTGSKATTTNDDKSDTGYSYSTSKLNIDGNKNGGDIAASSKVTTTSGNGYDYNTGSKVTTTNDDKSDTGYSYSTSKLNIDGNKNGGDIAASSKVTTTSGNGYDYNTGSKVTTTNDDKSDTGYSYSTSKLNIDGNKNGGDIAASSKVTTTSVSVNNGYDYNSGSKVTNTNGGKSDTGYSYPNSKLNIDGNKNGGDIAASSKVTTTSGNGYDYNTGSKVTTTNDDEPDTGYDYKTGSKLTNTNGGKSDTGYSYSNSKLNIDDDKNGGNIASSSKVTTTSVSVSNGYDYKTGSKVNRGDNTDAGYNYYNDRITTFKPIGYKYTTPVPKTGYNYQLSGLESMSDNEISNEVLTVNNQYLPAF*

>Woma_00008333

MNPLRIVCVLATLLAVCGANPTQSNMRSNAVSGSLKPSEWLTPSELENTPALDELTMQQLEEMPLEKGAYLVRKLYHISQINNELSPSFVPSPSNIPVYIVKNNGQKENCNLNNFVEVAKQQSNFGNEEVTIFITGLPSNTETVKKANRKLIQAYLERYNNKQQQPQSYQNSEEVTGSRTSSEESSSEWKNPKSTSGNLIVIDLGQTLNNAKRFALLNVAETGAMIGKSLVQLTNECNVPQEIIHIVAQGVAAHVAGAAGDEYQRLTGCQLRRITALDPSKIFAKNPKVLTGLSRGDADFVDAIHTSVYGMGTPSRVGDVDFYPNGPSAGVPGAKNVVEAAMRATRYFAESVRPGNERNFPAVEANSLKQYKSNNGFGKRVYMGIATDYDLSGDYILEVNSESPFGMRTPAQKQNYFHGVHGTLSQDY*

>Woma_00008334

MNPLRIVCVLATLLAVCGANPTQSNMRSNAVSGSLKPSEWLTPSELENTPALDELTMQQLEEMPLEKGAYLVRKLYHISQINNELSPSFVPSPSNIPVYIVKNNGQKENCNLNNFVEVAKQQSNFGNEEVTIFITGLPSNTETVKKANRKLIQAYLERYNNKQQQPQSYQNYEEVTGSRTSSEESSSEWKKSKSTSGNLIVIDLGQTLNNAKRFALVNVAETGAMVGKSLVQLTNECNVPQEIIHIVAQGVAAHVAGAAGDEYQRLTGCQLRRITALDPSKIFAKNPKVLTGLSRGDADFVDAIHTSAYGMGTPSRVGDVDFYPNGPSVGVPGAKNVVEAAMRATRYFAESVRPGNERNFPAVEANSLKQYKNNNGFGKRAYMGIATDYDLSGDYILEVNSKSPFGMRTPAQKQNYFHGVHVALSQDY*

>Woma_00008335

MNPLRILFVATLLVAAANANVSGRSSQSSLKPSQWFSSSELQQTPAVDEIAWQKLENMSTQKGSQLMEQIYHLAQINHDLKPSFVPSPSNIPCYIVKPNGQKVATTLDKLVSACQQQPKFGNEEVTILITGLPAAYSETVKKANRKLIEAYLQRYNNKKQQPQSFAALSENMARTSSEEDSNEWQNQQPSSGNLVIIDLGDKLNNFKRFAMLDVEQTGATIGSVIVQMTEKCNVLDETVHVLGQSIAAHVAGAAGNEYTRQTGRQLRRITALDPSKIMAQNPKILTGLSRGDAEFVDAIHTNTYGLGTVQRVGDIDFYPNGPYNELPGAENIVEASMRATRYFAESVRPGNERNFPAVAANSLKDYKNNDGLGKRVYMGIDTDYDLEGDFVLEVNARSPFGKRVPAQKQNNYHGLHHASSN*

>Woma_00008336

MASSRIICVIALLVATAIASDNQSGYSKWKPSQWLTSAELNAIPSVNELALEKLENMSVEKGNKLLETIYHVSQINHDLKPEFVPSASNVPSYIVKPNGQKVTTSLENLASACQQESDFGKQEVTILITGLPATTQSVKKANYKLVEAYLQRYNYIKQQPKRFDYSSEKMLRTSSEEDSNEWMNQQPSSGNLVIIDLGSKLTNFKRYALLDVKETGAKICSAIVQMVEKCNVVDDTIHVVAQGIAAHVAGAAANDYTRQTGRQLRRITALDPSKLWAKHPNYLSGLSRGDADFVDAIHTNSYGLGTVQRVGDIDFYPNGPCDAVPGSQNIVEASMRATRYFAESVRPGNERNFPAVAANSLKDYKNNDGLGKRVYMGIDTDYDLEGDFILEVNANSPFGMKAPAQKQNNYHGVHQV*

>Woma_00008337

MNPLRTVCLLMGIFALASANNGLSMKPTDWLSPSELESMPSLNEVSFQKLENMPLQEGADLLNKMYHLAQASEDFEPSFVPKPNQIQAYLLTPNNEKISFKLNELPKIAQEQKEFGQQEVTIFITGLPQNSESTEKATRKLVSAYMQRYNSQVPQPLNIKYQSGSAENNNADSSQEESWNNRPNKPSGNLVVVDLGTLLTSFNAYASLDIEETGAQIGNVLVQLTDQANVPQEIVHLIGSNIAAHVAGAAARQFSRQTGHQLRRVTGLDPSKIYAKRSNSLTGLARGDAQFVDAIHTSAYGMGSPARCGDVDFYPNGPSAGVPGAKNVVEASMRATRYFAESVVPGNERNFPAVGATSLQEYQNQNGNGKRVYMGINTDYDVEGDFVLQVNANSPFGRGTPAQKQQNYHNIHKTWHSTSSSNM*

>Woma_00008379

MLYKFWQLKISAFVLCLALSLGQELLPTCERDYCYEQLIERRSTDADYLRESNGGKGFHRIKEEHPENVIRKVHENRVKISDFESRGHVSRRENLENRRELDYITSKCKELIGRVSNRIRNENRVVIDDISAVANEDCKRSKIDFLAVNNHDNYRDISFDEIRRIERFGERTVERRQNPVENEREDRLVTTRRLNERDNQQDKNQEFLRFIDRRKNLVEISNSESSRENREIRLQADNLILNRRELREDNKAIEEENRRSGMSKMNLLTVDKRNNYQYVDRRFDEIRRSKRFSERTFERKQSPIEYELENRSVTSKRLNERQSHQDENRDAFRFKDRLSQLSHVDHLNERDESTYRLRSAFGEHTDNERRCNSREDLTGIEANNQRDKSNDDSNRIPNERVSENKSRFERDESRRLEEYKPQLVEREQLNENHRYIERHSEENRQERDKLERNHEARRYKDRFTETERLSEKLENLRRLSERENRRFNERLSLSLNKDFSECDNKRGDMNRESLNLNERPNRRPQGIPQLYSENEQERQKRECRKNVRQLDNQDNRYDISRLFDERTRRQSENNRDRVDNRARISKNDSKIKEGELEEHREIILRAKRHVNRLGISSELNSRLERQYDNLRERENVLNRSYESQRLNSRLADFEAGALQNRDESQRDHFNRRIREHENYKEHRNIRGRMNENREFRSIDERQDILSYDHMKDILSKDETLQYLDQIQRQPSNRRIKDHENYRGQRDIWERLDENREYRSINERQNILLDIDIESQCSRTEYILVEKRDENIRKLDDRRSGNREGHRDNNIAFSEDQAARKDHLNHPNNHSHDEIVQRHNKNKRSYNQRSRLIQNEEILNMIDEYKEKPIFFLNKGYMAFGQGALLAYVIMKTLNSKNDIKYNIYQNLQETLGLMGF*

>Woma_00008312

MATSQTNSLESLDKEQMKTFSDFLMSYNKLSEMCFTDCVRDFTTRQVKDSEEKCSLNCMEKYLKMNQRISQRFQEFQMIANENALAMAQKTGKL*

>Woma_00008314

MRFLKVFTALYVLCGFYNKIATSQNIVPTVLVHGGAGDIAESSIPVKKYGVKLAARIGYETLKSTGSVVDAVQQAVEYLESDPNYNAGYGGVLTWDGDVEMDAAIMDGCNLNAGCVSIAKDIMHPVALARSIMDKTRHKYLAGDGAMAYAKSQGFELLPKGALVTENAKKSLEAFKNSFCNNSKLMEEKIFGSPGTVGAVAIDAYGNVAAATSTGGITGKMPGRIGDSPILGGGTYADNHSGCVSATGNGETIMRYNVASRILALTEYFGLSAQEATVKVLDEMTERFNQTAGVISIDPKGNLGIYFTSRRMSWAYQRGGELHFGVDKDEDNVEIVGEPRV*

>Woma_00008338

MNPLRTVCLFIGMIALVSANSPMRWPSSSNSIKNNWKPTDGLSVSQLESLPSMNTITFKKLEQMSVQEGADLLNKMYHVSQAGQAFEPSYVPKPSEISSYLITPENKRLYFKLDELPTIAKQQKDFGNEQVTIFIAGLPQETESVKKATLKLVEAYMQRYNGQAPQPLNINYEDNSHDNKNPTSSEEDYVETWKNRPNKPSGNLVVIELGNVFSNTNDYVSLDVERTGIEVGNVLVKTTDQANVPHEIIHVIGSNVGAHVAGAAGRQFTRQTGHQLRRITGLDPSKIYSHHPQSVTGLARGDAEFVDAIHTSGMGTTARSGDADFYPNGPGAVAPGADNVVEASMRAIRYYAESVVPGNERNFPAVRATSLEDYNQHQGFGKRVYMGINVDYDVEGDFMLKVNSKSPFGRSEVAQKQQNQHYIHKPWKMSA*

>Woma_00008400

MKAVTVLGLLTIGVALTNAYGVSFGHSGGLGLASSSSGFASASSSSSASIGGGYGGAFGSTGAGFGKGNDFGHHKGAAGSAVVGLGLGLGLGTGLDLDYYHGHGHGHGHGHSHAHGHHKASVAGAGLSGGASLGFGGGHGAGHSSFVEGHHAHSHNKAVATGAGFGGGASLGYGGGYGHTHGHHKAGARAGLGFGGDVSHLSGAFGSLSGFESGHKIGYSGGHAAGVGSGFGGSLGGTLGGSHKGGYSSGHIAGGFTSSSASTGVSGGIGGSYGGHGSGHSVIGGIHKGDYGGGHSSHTSSSAGAGAGNIGGSFGGSHSSGHSTAIGGSSTVGAGGGYGGSIGGGHHGGYGGKHSTSSIGSSSAGVSSSIGSSLGGSISGGASGASAGGSGSSGSLGSGFINSVNGGASSTASTSTSGLAAGIGGGLSGSSSGSSSSAMSGSSSTGAGGSLGFGLGAGIGAGLSGSSSGSSSSAMSSSSSTGASDSLGLGSGAGIGGGISGSSAGSSKLATSGVGAGIGGSAGLSSATSGSSSIASTSTGSSGFGWNAGIGSGSSSTGAATGFGASAGSTSGSSSSPGASSGASSSSSSQATFGAGISGSAGLSSSTSGSGSGSLTSSKYTNSHAVASSGVSGSGFSSGSSGLSGSSANAGASSAGSSSSTSNSAGISGNIGGGLGGSNGFSGSDSSQSSSAASGGSIGGGFSGSSNSLTSASAGAGSSLSAGLGLGAGVGGGSAFGGSASGSASTSSSSQVTGSGAGSDLFGAAGIGVGAGASLSSSDSVASSAGISSSAGVGAGANADAGSSSSVGSGGSIGGSGGVSGSLGGINTSSSQASSSSTTTTSGSTNGGFGISGSLGSSYGSSIGGAGAGAHASGGLLSAVHGVASSLTSGISNSLGGATGGIHGAHGAGSHFSTAGSAGFGGSHLGGYGSKGSFSGLGGYSGHKGSYSGSYASSSSSSFSGGYGR*

>Woma_00008401

MKALIVLGLLTVGVAITNAYGVSFGHSGGLGHGSSSSGFSSASSSSSASIGGGFGGSFGGGFGSAGAGFGNGNDFGHHKGAAGSSGIGLGLGLGLGSHSSSSAGVAADNIGGGFGGSHDSGHSTAIGGSSSAGVGGGYGGSIGGGHHGGWGGKHSTSSSASSSASASASGSSGSGLGGGIGGDLSGSAAGSSYLATSGEGAGKGGHHGSKHSTGSSVSSSAGASASGSLGLGLGGGLSGSAASSSNLAASGAGAGKGGDHGGYSGKYSTGSSASSSAGASASGSLGLGLGAGIDGGLSGSAAGSSNLATSGAGAGKGGHHGSYSGKYSSGSSASSSAGASASGSLGLGLGGGSSGSSTGSSNLATSGAGAGKGGHHGSYGGKYSTGSSSSSSAGASASGSLGLGLGAGIDGGLSGSAAGSSHLATSGAGAGKGGHHGGKYSTGSSASSSSGASASGSLGLGLGAGIGGGLSGSAAGSSNLATSGAGAGKGGHHGGKYSTGSSASSSAGASASGSLGHGLGAGIGGGLSGSAAGSSNLATSGEGAGKGGHHGSKHSTSSSVSSSAGASASGSLGLGLGGGLSGSAAGSSNLAASGAGAGKGGHHGGYDGKYSTGSSASSSADASSSGSLGLGLEAGVGGGLLGSSNLATSAYSGHKGSYSGSYASSSSSSSSGVYGR*

>Woma_00008253

MTSKNILNILFCLICAQSVIGVTIVSKSEWGGAPATREIFLPNGLSYAIIHHTAGAYCSTKASCIQQMKNVQRYHQKTLAWDDIGYNFLIGGDGNIYEGRGWNVLGAHAINWNSRSIGICFMGNYNNNQPTEAQIAAAKDLLAAAVSRGQIISNYILKGHRQVGATECPGNNLFNEIKTWSHWKA*

>Woma_00008254

MVSKTLFGLLSVLALSQAVFGVTIITKSQWGGAPATSKTALGNGLSYAVIHHTAGAYCSTKAACIQQMKSIQSYHQKTLGWADIGYNFLIGGDGNVYEGRGWNVMGAHATSWNSKSIGISFMGNYNNDKPNAAQIKAAKGLLADAVKRGQIKSGYTLYGHRQVGSTECPGNNLYAEIKKWANWKA*

>Woma_00008238

MKYFIVIMAVVMTVALSQVKADWKDDIHKIDMACREESRVSEDEIKSFFKGDLKDPKDALKCHIKCFMEKQGTWKNGSFDDNVAKKQIQSIPGLKDKQDEISKAIDECKNQKGSNECDTAYMITKCLNEHKASSM*

>Woma_00008237

MIFLTILLSISNLADPALSKTFTRCGLVRKMLILGVPKYELAPWTCIAEHESSYSTNFVGPANSDGSNGYGIFQISNLYYCQSSNGPFSYNECLLSCEDLLSDDISKDIKCARQIQFKKGWTAWSTWRYCKDSLSSIDECF*

>Woma_00008619

MKRSKWSLSSTQHSILSWSLMMMLLLNNGPQLSYGHGPTTFMPALECYDKYNKPQKCMPEFINAAYQLQIEATNTCGEQGENHFCVQTMNSNYKNCEFCRWEDHNPSFLTDLHDPQNPSWWQSETMYEGIQHPNHVNLTLHLRKSFDITYVRLLFRSPRPESFAIYKRTCETCPWIPYQYYSATCRDTYALLDSRAIRKGEGEAHALCTSEYSDISPLRDGEIAFSTLEGRPSGINFERSTELQEWVTATDIRITLDRLNTFGDELFGDAQVLKSYFYAISDIAVGARCKCNGHASKCVPSTGMNGERRLVCECRHNTDGPDCEKCLPLYNDVKWRRATSTDVNECKACNCNGFADKCFFDAHLFNTTGHGGHCLDCRDNRDGPNCERCKENFYMREDNYCIHCACDPVGSRSLQCNTQGKCQCKPGVTGDKCDRCDANYYQFGPHGCQPCGCDPRGSFGNMPSCDAETGICRCKDNVEGKRCNECKPGFFNLDMTNRFGCTPCFCYGHTSECQTAPGYSVVSTTSNFNKHKERWSAVDLYNHDIDIKYNQYSHSIGTTAHGNEYVYFQAPERFLGDQRASYNRDLKFKLQLVGQIAPSTSASDIILEGAGTKISLPIFAQGNGMPDNDVKEYTFRLHEHRDYQWQPSQSARGFLSILSNLTAIKIRGTYSKQGEAILDDVELQTAHRGAAGQPATWIEQCTCPEGYLGQFCESCAPGYRHSPARGGPFMPCIPCDCNGHAEICDSETGRCICQHNTTGDNCDQCTRGYYGNALGGTPYDCKRCPCPNDGACMQINGDTVICTECPVGYFGARCEQCSDGFYGDPTGLYGEVQTCKSCDCNGNVDPNAVGNCNRTTGECLKCIHNTAGKYCGECLPGHFGDPLALPHGQCDRCSCYPAGTEQDIDGISQCDQVTGQCQCKPNVIGRDCGECQPGYFNIMSGNGCENCMCDPVGSYNSTCDRSSGQCFCKPGVVGLHCDQCDVYHYGFSSEGCKPCECDGSGSKGFQCDQNGQCPCNDNVEGRRCDRCKENKYDRHLGCIDCPDCYNLVQDAANEHRAKLKNLSSTLDEIARTPVTNDGEFEAKLKTVQEKVDILLNDAKYGSGGSGKTYVEVLNDLHKRLESINSHLDSADTLQDSANDEIEKAKHNHTIAQDIIQAAKNRLKNALDLLHDEGEQALAKAQNKSVEFGVQSDQISDISREARALADRLKSEAQFDLKNAKDANDAVEKAYELAKSAINLQQKVSDELRTEVRLELETVKQGLGTATQTTKEALRKANEVYDAALSLLNDVSGLTAPNIDIQKFKEEALEANKKADELLQQVNDISNSNGELFGDFEEEISLADIMLQRANQQKLDDIELLKRAQDAYDKATKAVEQGDNTLKEANNTYHTLAGFQSDVQKSSEKADLALQTVPSIEQEIEKAENLIKQAEEALEGANKNANEAKINAQEAQQKYAEQASKDAEYIRKRANETKVNARKLRDEADQLNHRVKVTEIDIGKLEESSSKDDNLVDDAKRKVGQAKADTEEAKKQIDKAVNELDDIKEELINLKDINIADLDKLEQRLDAVELDVARVNLTGRIEKFREMRNIQKKTVEKYEKELIDLQQEVANVRLISEALPANCFSRSRLEP*

>Woma_00008611

MSFIRQEIVYNESRIRLSSTAKVARWFQQLPWNTASLSRPESGFVSGDSRSEKFHDDQQYFSLKDTDDLCDEFLLVEGSSTIWTKLSAKKRRKLLGMRLGKTSVMDSFNVDDLDNDKSLIEKGNK*

>Woma_00008537

MLPFHWGLLSVAFLIALTRGATINSPANDDYAADTQRSKRSYKRGQTQSQYLNFGQPELDGKAEAEATESGSRSTVSGTHGMGQAQSQFSSGDCSGCGSPVYEYPTGSPDPLTLGGNVISYTDGKPSSIIGLPGTPTGGTQIGKPSAGSGDFAPGTGVAYGPGTSGASPGFDPGAGRSGQVGQPGIGGGFIPGAPAAESGGPGASASYTPSVSGAGQVGGAPGVSSGYGPGSRVGGFPQTGTGVGGVGQQSGPGAQFGPGAGVGRVGQQGIPGTQLRPGQLGVPGTQFGPSTGVGQQGTPGAQFFPGTGGLGQQGAPGTQFGPGTGVGQQGAPGAQFGPGTGGLGQQGRPGTQLGPGTGVRQQGAPGSQYGPGTGVGQQGAPGSQFGPGTGGLRQPGAPGTQFGPGMGPGIGQQSATGTQFGPGTTSFEQQGVPGTQFGPDAGVQPTGGIGQPGQRSQRPQLIAPPAALPGQAPGFGHQPGVGYGQQMPSYGPGSGPAQPGTGQQISIGGQQAQQYRPGGQTAPTYDVGGRQPTGVTNGQQVPSYLPGQTGGVSYLPGQQGVDKQISQPGVHYRPGTGHVPGQQQIPQYGTDQRGIGYSQQPGYAPGYGGIGESGRQPLGVFQPGGGAQPGGFVQPGVGGQPGRLQPGYGQVGNVQSGTGHVLPSGEIIQPGERGQPGYGVQPSVGFVQPGAGTQQGHVQPREGLGQRGIGYVQPEVGVQPGTGISSGTGYGQPETGVQAGTGVQAGTGVQAGTGVHSGTAYVQPGGRAHPGTDYIQPGVGVQPGAGAQPGIGYVRQGLGVDEQGTIMHQRGHQPGTGFGPRGTGYQPGTEAQQLPGRGQERQGVLPEYGLTISGLQPGTATGGGHPGARPIGSVSQPELVYPHGGVQPAVGGARGGVGVQQGTDFQHGPGFETVYDANFGQQPSKGRPGTGYGQPGVESQYGRPGYGQPTTGVQAGYGQPTTGVQPRAPGGYGQPEAAGRGAQVGYGQPGSTAPGTQSGYGQPVAISPGAQTGYGQPGEIGSGTQAGYGQPGSIGSAAQPSYGQPGATVPGTQAGYGKPGAIGLGAQTGYGQPGAIGSGTQTGYGQPGAIGTGAQAGYGQPGTGVTQYDQGPQQIIQPGSTFVPSGVQTSGVQQGVGSTGVLSSGVSGADDAFSQAESSIGNGQAAASAQGKKNGGTAKTQVQGTYSSSGSFSASAMTSDSDRSASAQVTGGTEGAMSQSQGQGGAAQSQAQVQVNEKTGGTKASSQSGGILHQSQSEVQANDKGGLADAQSSGPGQTSSQAQIGFRPGQDGEIVRSTGGGQASAQSGVHSGNSQSQIQGTSKYGVSYHGAAQSASGTKEQVATYREQNRDLFNTISQFGNSDAVTDRVDTVYNPSLTSESDIPDLQLKNSKTNKEVANSNKLDNDDSNLPDEDDEEEPYDEYEDEEDYYNENPIKMDSKNDNDKKLDSQSLYRSHTQSSPTQTQQVAVADSTKEYSVVQNQNGRVQTYRSRTTTETVPSGFRGTVNVEKKFHTKSLEPHKSENKLNITADEKSENKSRTPDSYVTVTKSVTGSMDNSKNPPQDNKNFQSTYYTKSSTCGYFTFSCNIVYGANGRSKICRPKAPANGKC*

>Woma_00008600

MSASIIKFILMASLTAGVFCSSTDRNPKKRQIYREQVLPHAGGRIPDTAFETDRLFLNQLQKDGIAVPDGIVPEQRNPQVYPFFNRPSRKVFRIALSSDGRFIPEVGRYHNQELPSVDTTYLYPQIHGHRGTFAPQTVELPVYRPFRIHNPLLNQVKFQPKDKAQFHNVNRVLSGKANNNPKSYQNVQLLNTPLSPLTSQTSNCGNIYLPSMTSSSHLFNEFDNLFNQFEIYDGKIKKTIKNEHLPATC*

>Woma_00008553

MSAARVLNPITEFCMMTSSPHSLRMNLSCGRQLNSIKRNLFGPVNPIETNKIFNEELEKHQEIAAKKWGFDFRTGSPLTSSSQYVWEGVFLKEGFITPEMHTVIRDAHVRPSLTISAREILMNERADRENFGMFQSTTNTDSCDESQGEPPSAFVFKVPALPTRLRKGQPKITEYMEERKRSGQTLKTASPAKRVRTSPGCCTTHYNHAIRNNSIAAYFDRRTQRHD*

>Woma_00008589

MLVYCFYFLIWHLYVGIAADQKVGHNDNPIFEMEYTTVSKEMRKEDHRHLSQFIAHAALDLVDEHKWKTSNMHLKSIDRFNQWFVSAFVTASQIRFLIVHDHKNDEGIKNFFNEIYETYVKYSMNSFYKVNTPIKSPMFEKKAQLYGRKYLLS*

>Woma_00008606

MFLVRLVLIFIIILAFSLATYKFTNIKCQNLDSTFAIFSKCELKLIKRGVPALNIYVKLLQLPVTNISVFASLYKKANGYKPFLYNVTLNFCDFLKNRKKYPVMNFFSDPLIKHSNMNHTCPYNHDVFVNNLVLTDTMYNKLPMPEGDYMIDVIAYVYNDPKVNIKYYLEHT*

>Woma_00008569

MFKIFVLTALLAFAYADNIDKDAQILSLKNDPADAEGNFAYAFETSNGIQQQEAGNPNGVAGSFEFVSPEGEKFFVTYTADENGFQPTGNHLPTPPPIPEAILKSLEYIAAHPPQEVVQKK*

>Woma_00008567

MKLFVCIVALAFVTVCRADNIDKDAEIRSLTNEAADAEGNYQYAYETSNGIQFQESGNPAGVRGALNYISPEGEHIALTYTADEEGYHPVGDHLPTPPPVPAYVLRALEYIRSHPPPQVKEQQ*

>Woma_00008566

MFHIWTTLFLAFFAVHSLAISEMDAEITKYRVAHADENGVFKYAFTTSNGIDVQAAGSPLETIGIFSYNSPEGVPIEVRYIADELGFHAVGRHLPKPHPIPEYILRSIEYIKKHAPPQQLK*

>Woma_00008586

MIFSARLAMLVLLVLLTLVNAFPISPNNDQKHHELSRTRQKRQIDLTLSAEHDDKEDETEIALEAIANLWRSSDGSTQIDGSAKVLHRSNSLQNGSGLDWRLGFRVLFT*

>Woma_00008786

MAQAKVCLTLASLALMATISSLSFECTEAAVYSQRALFHPAHPGKCFDKFTKRAMLPNKEYRPKGICAALTCDIETETINIETCPTIEMPGCEELPTNPSWTFPKCCPQFKCTDFKTGKEFVVSV*

>Woma_00008777

MKFLKSFECNRMLLMVLLVCYMDGISASKCKYAVQRGTKSLTSKYEYGISKKVIVSKNPEVKIHAVDLTPKHEREDQDQKKFYVLKNEPKFKDLNDSQAKENAWKTIINALDRKPPATPKTSVPSSTHSTTTHTESPYNPTIYSAGPLNRQSIPTHYGYNHVYNIQNHGTHHHYYGSPATDNTQNNAFESKPFREANNTTQFTNKVPLNK*

>Woma_00008736

MKLFVAILALSCACLAGAQTNVIDAAEPTNEYLPPEAAAEESAQLTGDGYRYKTIRKLKYRARHRRDVSQEYLPPMEEPTQEYLPPVNEKAETKLSDDGYLYKTVRKLKYRSRHRRDVSEIEEPSKDYLPPVDVELAPDLKTVLADDGYRYKTVRRLKFRRHRRDAGNDDAAAAPVNEYLPPVEEKPDNADTVEVKSALLADDGYRYKTVRKIRYRARR*

>Woma_00008779

MHKNQHQQNCILLLLIILVTVHYITTEIATEDASPSNTEVLVKNKAATKKGEELQPANGSTSPKERSTPAEEIQCFVFSQSYDDGPIKQNISELEKKQSQQELLLKQLIQDLAFIKRTLVKIINQNPDWTVILRRQDGSEDFYRTWDEYKEGFGDSQGEFFIGLDNLNEMTSGDDAQELLIILEDFENKTRYAKYDKFVVGSEDDKYAIVELGEYSGDAGDSLSYHRGRKFSTKDQDNDNNPNTNCAKEYKGAWWFDNCYSSHLMGPYYRGARSCRGGINWYQWHNYMYSLKFVEMLVRPAPSADEG*

>Woma_00008776

MNFNTFYIFVACCLCLFYAGNLIQAEIVCPAEQPDDSLVIQFPSPTNCSEFYKCDRGVAVAIPCPKGLHYNARLQVCDYPKHANCKLATTA*

>Woma_00008795

MKGNVFVSINEKYSNFGLFNSGIPQGSVLDPVPYTLYTSDMSTMDNLAIATYADDTAILSLHHSPTMASETNLDATKSVKSYPRLVTEMERKSKCTKIGSRNIYLKKRRLLFSIFKWNYFTKK*

>Woma_00012523

MDFIRMLYGVSILLILCLQTFCLSQSSSRFLTLPQSQTIAEGDFVDFNCHATPTRGLLYTWTLNGHLIANTSRIYQNGSDLHIESVSRDSDAGDYVCIATNVANGARLASPPARLSIICKGAP*

>Woma_00012504

MRTISLALLLAVMAVSLVDFSSAINYVPAEKWKEYKDRFNKNYNSAVTEGYAQYYYAYNKRMIDRHNNLYERGLRTYKLAENEFTDMRFRQFNALFPEVTSSGPSYDSPLPEVQPAAPRYDPITDFGISSNIENQGIKCNSGWAYAAVKAIELLQAYQSGNMSPAPLSAQNIIDCAGQAAACKTQVPQAAFDYLTQYGMDLHLESDYNNNNTLSEPAMCTPSGQLVTNLANYSRLIDGDDESLKAYVSGGIPVVVEFNPTSFEFMHYSCGIFQQPATNRGTHFMVVLGYDTDITTGMDYWLLQNSFGVDWGEMGLMRLFRSPTMKLTKNAIFPTELA*

>Woma_00012505

MARISLIILTIVMISLLELSAALLYVSPQEWADYKLKYNKDYGGFPFSDTYAQYYYTYNKKMIEKHNALYDRGLRTFRLEVNQFTDMRFIHYNALFPEARSWTRFPDTQLPTLSTAVADNFDIRTFVTNHRIEDQGTKCNSGWAYAAVKSIEIQKNFQSPLPGPLSTQNIIDCAGRSNACRNEVPQAAFEYLTTHKQNLHLESDYMNNKQLSEPDMCKPNGQFFTNLALYAKIDGTNDELIKQCVNAFYPVVVEVNPSSFEFMHYSEGVFQQPPARRGSHFMVVLGYGHDTSLGMDYWLLQNSFGDTWGEQGVMKLHRAPNSKLSKIALVPLE*

>Woma_00012494

MFFSLLQTLVCVLLVNTGLYALHLTGSASSSGGSGGNGGGGGGGGNNMGSGPNSGSSTQFSKPSAALNPFKSSTSNRLNEELADLAQLERERLMVMGLAKQTAEESIGYHGRPIITGRIKMHPAEDMDSEYSAWLSQQARNIMFGNVVKQPLNVEEDYDSGGDTGGFDILRPATTKYEYGRVVQPMDRERERDFVNPHNLDLDDFMELEPVNDMMEEDVFPDMDGYAYLEELMAEENKERMQQLQQQHQQHQHQQHHYAQAQQHQQQMPQFPGLRQTQTHHMPYSPSASSSALYQNHEPVDETKQAVQEFLEKEQKLQNLNHHQQQHQQQQQQQFNTKYRNNIRMQQKWLRKRNQQKEQLTRNIFGQKNAKALTQTLHQPVSLHHFKGLSSNKLAMANSEATATTRINGGGKQHEIAQQQKLNLHEKDINKNSSSASSSLSAAATSTAAVNNNNELKLELLQHPNHKRSGLTPASLASSAGVSSPHSLASQLMLRTARGQRQYDVPQIGKCNQKNIENKIYK*

>Woma_00012503

MFQIHNLTALLALMTLTMCISMTYALSNFPKLCDVRNYDDFLQQTGKVYQDEKEKMFRESIFKAKKAAIDLGNKYAALGLMSFHMELNTLADLSNREVAKLLGSKITFTGEEITSKHTNFVTAKTNTQNLPDHFDWRELGGVTPAGFQGLDCGSCWSFATIGALEGHLFRRTGLLIPLSEQNLVDCAEDYGSMGCDGGFQEYAFEYIRDHGVSLEAKYPYTEMENAECGRNETYDKGVFIRDYARIKPGDEEKMKEVIATLGPLACSIKADVITFEQYSGGIFDDDECNQGEVNHSVVVVGYGTENGRDYWIIKNSYSANWGENGFMKLPRNENSFCGIASECSYPIL*

>Woma_00008813

MKRQLKNFAPLLVIAVLTCCALEGNCAEALFKSAENNQYLVQPAYKYNWYEAYKECNQRDMDLLTIESKEKAKEVEDLLNKIFTGKRIPRFHIGANDLNKFRTFSWINTRGRTPFTYTNWEKTEPNNYKKLNERCVHIGFHGSVQWNDINCGRKYGFICQERKDFESLLNQVSRA*

>Woma_00008889

MKFFIYPVVLLTIILSLTLADLNHADEPLENTTKAPVLAKCKERICSRIYSPVCVSINGFEVTLPSRCYLARQRCVAMRKSLKNTKATASPPDIRVLYNGECYAKSKMKPKRKSIAQRRKNSKFLKFPQHDKYEDY*

>Woma_00008884

MATINTTTKSPPLSLSTKSKSADHTHKLKMKYLQSHTEYPMSTLSPRNFTLKRQKRIGENRPLSRKSRPSSMRKSKSLDAEHTYTLATIQAPLWVTLTNARTIEELSKEQI*

>Woma_00008799

MGCLHPLVLLLMAVGLQITIIESRGYYTYECKPNQFQCRSGSCIDGSKRCNRVADCPDGDDEDERCPAECSDIEYQCRDGINCIIEAKICDGKIDCLDGDDEEHCDSIVPKLKFFCPKGKFTCRDYSCISIVHRCDGHTDCPHDRSDEEGCPCLHDKWQCDDGTCITKNLRCNGNIDCPEDISDERHCDGGDDSDDSNYCKHYEFRCNNGQCIPYREVCDNIYDCDDYSDESDCDLHDFNQNIFDEDEIIREYAPHHKRPATSAPPTSYHHQQQRPYGSSSTGHRGGVLHEMDMRDYYYYHPNIYNKANQHNPCSERQFRCGNNVCIPLHLRCDGFYHCNDMTDEFDCDQYRADGEEQKNKPFTESPPANIIRTSTTAVPPRNTTTSRPTTTTTVITTTTTTTTPAPTKKTCLSMEFMCESGECIPLESVCDNIRDCERMDDESYGLCHCSSEKFKCIRGGGCIPKTQVCDGKPQCRDGSDEMSCHFTANFNKTRNLAECLSFQFQCADGICIAGYKLCNGITDCLDGSDEINCPLNYDDANYDFVPEDNTLSECDIYEFECDYSRCIPIEKKCDGYPDCDDETDEIDCPPFTDNCNDNEFECDESFCILRDQQCNGVPNCNDGTDEKNCTFCREGAYLCNTGECILDNLHCNGHNDCADGSDEYNCVKDECPPLFMKCNDTCITWNLRCDGKIDCYDGSDEQGCNGHKNDEVKIIFGNETCRTDEWQCDNFECINKKLLCNDHYDCQDQSDESPKQCATMVTTVLTPDDCNSNQFFCDDDCHPISIRCNGQYDCADRSDEQECSRPTRRPPTYPCPQHTCPDGQCYSENERCDGISQCDDGSDEAECCPADQFRCRNGDCVPGYAHCNGRFECLDRSDEEDCTDTMPAYPSRCSSSQFRCESGQCVSALARCNGYTDCLDSSDEKYCSMSTVTTITSTATQTPTVGISKACGPNMFRCENGPCISISLRCNGNFDCPFDTSDELDCPEMDNSIDSNVPTTASPQLNLKTYPDNQIIKESREVIFRCRDEGMLRAKVRWTRPGGRALPVGARDKDGRLEIPNIRMEDSGTYICEAVGYPRHVAGQQVSVQLTVEKFNEERVPSSACSNTQATCLNGECIDKSQICDGIPHCSDGSDEHSCSHGRKCQPNQFMCRNSKCVDRVWRCDGEDDCGDHSDEESCDPEPSGAPCRYDEFQCRSGHCIPKSFQCDDTNDCRDGSDEIGCMAPDKIRDPPPAANLKQGDSLNLTCVGVGTPTPVIVWRLNWGHVPEKCVTKSFGGTGSLYCPDMQVSDSGAYSCEIINTKGSKFATDTMVTVEPPTRPGVCPAGFFNMLARRPEECINCFCFGVTKSCKSADLYNYAIQPPITSHRVVDVELSPYSSIVINEAPTSGIMNLRHGVQFRATDVLYGSRSAPYLALPPEYMGNQLKSYGGFIKYDVNFMGNGRPTTSPDAIVTGNGFTLTYRSRIQPQPNIVNHMEIQFTPDQWRKPDGRIATREEIMMVLANVDNVLIRLSYIDATERQVELTNILMNSAGVHDQGLGQASLIEQCACPVGYTGDSCESCAPGYVRQPGGPWLGRCVPFTPEPCPVGTFGDPRRGIPCRECPCPQTGSNNFASGCSLGPDNEVTCNCNEGYTGRRCEFCAPGYSGHPLTPNGRCFPIPENTCNAEGTYSANANGTCTCKPLVTGPRCDTCKPDSFHLNAFTYTGCIECFCSGLPTTCDSSSWSRDQISSSFGRSSAPHGFGLISHYNTDKPLSVKFSQSGNTLSFSEPRTSEPLYWQLPAQFLGNKITAYGGKLNYTLSYTAMPGGLMSRSTSPDVVIKSGEDLTIIHYRRSGVSPSSANSYSVPIIESAWHRSDGQPVNRQHLLMALSKIDAIYIKATYTTSTKDGSLTQVSMDIATPTPLGTARAVEVEECRCPEGYIGLSCERCAPGYKRNPEAGLYLGVCEPCECNGHSTQCDSETGACINCADNTEGEFCERCALGYSGDATNGTPYDCQSALEPGDYKTTPRPGGNQSCTYCNPDGTRSCDNGYCYCKSNVQGTYCDQCRPGTYGLSGSNENGCNECYCSQKSSTCRAANLYRQFIPVDYISHPPLITDEEGLIADTENLNFDIERNEFTYSYTSYTNKFWSLRGSVLGNQLYSYGGVLSYNLDVQSYGHYIPGNDVVLIGNGQKLLWSRPANEQENTEYSVRLHEDENWQSMQRGVMQRASRVDFMNVLSNLEHILIRATPKIPTTRTTISDVILESAVEIPGHDAQRATDIEVCTCPPGYSGTSCESCSPMHYHDNNGNCLPCPCQDESTNSCNLDERGYVKCQCKAGYTGDRCQNPVIVSPLQPTPPQMICDISRGFCCSGFQFHIEPNQSISYNETLLLYRGRQYVGNVTKLRYGCNIRDGYETETPAPRPTPESDIRTQITVSIAPPEITIIPVGGSITLSCSGRLVWNGSPVIVSWYKLNDDMPYGWEQSNGVLRLHDLQIHDSGVYICQARNNETRTVFEDKISITITESSRRTPAKIDNLPPYYTFVEYERSEVNCEVSGNPTPSVVWTRVDGQMSNEARVEGTRLIFEMPRKSDEGNYRCQVNNGVGYEEKYTQIYVRPSTPLPTPPPRELVYIEPPSFSGESGEYVRLSCQPTTPIILKYEWTKDGYPLYRQHNLIINDNMLEIRDAAPRDSGVYTCIGIDHRGNRNYTSDAHVVIEDTRPPYQPGGGGSNLGPHTGSIAPTVQRLPEENRVIQGHDFTITCEVSGTPYPTIRWTKVHESLGDNVHQTGNVLRIMNARPDNRGIYLCIAENEAGNDQSSTFIDIEPREHPIVDVDPKEPQVITVGSQAVLYCSATGIPQPRVQWHRVNGQPLSPRHQIQQNEPGYIIINDAMLVDAGDYKCVAENEVGNATAVSTIRVIEAPVIEIEPSQEVLTVTEGDEVKVTCTASGFPNPSVRWVEDSAVNTYAYNPEAEHYNQAFLEFYRVSMQNGKAYKCIATNEAGTDERYIVLDVKPRRGDAPDDSDVDRSPYPYDRQPQRPPRPQPQPIPYQPTSYPSQVHPENVYETKPGDNVTLNCDLSAAYETAWVREDGRPLPSNSHFERNTLVIYHMQESNAGRYRCNAYDSRGEIITYHLSELVFIPIPHITLNPRMPIHVNANDNIDISCDVEGAQPIFVSWHTDNNRPLPPSVSIEGKYLRFIAITPAAAGRYFCSASNSYGNTTEMAEVIVNRGHSYEPRPQAKNYELNEGETVRISCDVEPHNIPIRGDVYYTWTREDGSRLPPNAQIRNNELYIFNVRKQDEGRYVCEAVTNGVRSKPSYAELYVKRGHSINDIPCMVLYICTDYKSLKGLKTKTPTATAAASRTSAYTCQPSDFKCVSHPHTCIAQHMVCDGIHDCTDHSDEFNCTLEQVNYKRWKKHSYQPPLKPLNAFHDFKRRHKRKFHQMLEKQPNEKKLYPFQKWQKWNHTNVLLKKGRRNSAGVRPMSPSYYPPPIMSTTPRIRDTMLRVDQQQSKLRVGESTEVECYSSDNSYTDVVWERADGEPLPPHLQQIGNRLIINHVTPNDAGRYVCKCKTDEGDLYTTSYELNIEASPHEWKHPKIVHADVGTRAKLHCDAENIYTSPSYRWSRQYGQMQIGTDILSDQLNLVDVQANDAGTYVCTATTSDGESVDYPTILVVTGAIPHFHQDPISYMSYPTLRDSYIKFNFDITFRPEKPNGLLLFNGQKKGNGDYISLSLNERYPEFRFDFDGKSMVIRSEQPVELNEWHTIRVNRFRRDGYMQVDDQHPVAFPTLSPSSSLDLVDDLYLGGVPSWDILPRNAVDQPVGFIGCISRLTLQGSIIELMKDAKVKEGITACKPCQDNPCNNGGICLESQTEMAYTCICQQGWTGRNCAVAGTQCTPGICGTGRCENTETGMECLCPLNKTGDRCQYIEHLNENSLAFKKNSFAAYGTPRASKLNIKFQVRPNSLADSVLLYAAESKLPSGDFVAVVLRNKHVELIINTGARLKPVVVRSLNPLPNNKWTEIEIARRFGEGILRVGLEPEQKAKAAGAARTLYIKTPLYIGGYNHEKITLNRDVNVTQGFDGCVSNLYEGQRQINLIADIDDAANIQNCGEINEIDQNETFAHEEQTNNEDDMAALDACASDPCENGGTCMVVEEEAVCSCGVGFAGKHCEDHITLKFDANFHGNGYLELNRTQFDEEIEQKYSFAAMVFSTTDPNGLLLWWGQPKGEAYNGQDFMALAIVDGIVEFAFRLNGEEAVLRNPDKRVDDGRRHIVLIKRTDNTAILELDHVLYADETRPTGKNTMSLPGHVFIGGAPDLDNFTGGRYKNHFNGCVRVVEGESSGIIELGKVAVSGLNVDTCPEAWNSSSLVYHDLDYNDFRFDPSMFDALDQPPPVHILYPRPTPPALNTYTSGVCYYLPQETILNLVIFNTLIESLAEYMSINRKYLQFFTRIKLKT*

>Woma_00008798

MGTLVESRPIGKLRRNLSLMWKLLAILVIIQASGINAKQIQNDLTFDDTFDIDQFEDAVSTNKLRSGRDLDEQAFIDDELQPVDENESESNNGGSWLMQSVKRVRRELGRLFGSEEKKAFAGKKHVKGAKAEKQRKRQAKLAEKQKRKALAARNKAKNRLNKRQSYGEVEGSGDDNENFGERDLWQTLFTVNEPWQEEYRLGKGHLIYDELQQQIVTAFHDFASQIYGDDDDEALHPVLIRVEPTSDVYKIHCVVQIELPEKWTNFGEEMRSYILKYHRFGADFSADADSGFYFRRVRELQDHESVDVTPDYEHNAEQQHHGEGGAHMGVGNIGDQQLEFGNCDDQGYFHCRNNQIIECAFRCDNKADCDDDTDEDVEMCERFFNEENAEEDEEEEDLDVNEVEHPESEHHNEVTENPNNNEENNGYGAYDYDEQERNRQYEEQLAREQDEQRERERQYQEKLAAREEQERQHQYELEQQEREREERERAQQERERQQQERAREQQEREREQQEREHEREREHERELEHERRREHEREREHDREREREHEREREHEREREHESGPAQPDFNGYDYNPEDTDHENEYDCQANPNGYFGCRNGEVIPCYLLCDNNVDCSDASDEEECHPDTATEEVPNVTDTGSDHSYDFEASGEDYAPVTETSPVYSSPVDYDGCRGDATFTCSESGKVICDEQLCDGIEQCPDGEDESNCPTDGDVDGNGDNEYEPETPCEDYEFKCDNRCLPKEYLCNGHHECFDKTDELNCPEKASHHSTLLSDFTLCPSFMLLVY*

>Woma_00008991

MDFKTHFLVVVGILMIISPIVIKANGDVKPNEITIRNQLISDVYLLKEGVDSLLINTEILNKRFTSQVEILKTVQSKLLAESAEIEDDSCECITGLFNSTFRKINVLENRLVLKLEDMQDEQTTRLDEILAKQKHNKNCDSKPSLPSSEFEEVTSLLTNIGSKIQTFDQKFAQLEKLEQKLNAQQNTLNKFVESQTTILQQISDKLAKNQELLEKYEKEFPKINDLKSELLAAQRRSRPKLPMEVILGLTSSNITSLK*

>Woma_00008992

MQSRMFSSKILACLTILWLRINVSKAYDNHGINGYFPIEEINTIKANMRLLYLDLDALQTRLHLQSDLINSIRTEVINTDTDFCECNAIEKFNEIDLQLNKIKKTLIKLLNTNDDTNFDKIDKILFNVKTSKSCAFADNAQNMEDIKMELLNKTTKINELIERLEYLESKLENHMKTDNENIKAMDYIVEQIHANLNVQENKLLELNQHLRELLTKTSSEVNKKSRCFSGNCLIHNLIEADKKLSQKENLLK*

>Woma_00008969

MNLKIVVLCMCILILSSPVSEGRRGRGRGRTKSRVQIGLPITGKYRDPESDQYYNNNNGAKILQASHFDYEYVLGHKIAFLCVAKGNPRPHITWYKDGTEIFQHLYMHVHEWRIGEDRVKSKIEIDPATQMDAGLYECTADNMYSIDRRSFKTDFSIAFD*

>Woma_00008965

MKILILSNVFIQISVILSATTQFNGYHYPRPFENALEYPNNNEKGSDLPPPVIQGDFIEDIITEHRQQVLSSILTETLDSDSSVGAEDSKLFLGNKCATCSCGVPNINRIVGGNRVRTNKYPWIAQMLLGSFQFCGATLINDRYALTAAHCVYGMDIKRISVRILQLDRGAVNRGIVRKVVFANMHRLYNPTTLQNDIALLKFDQPVPLLDPIRPVCLPTNSNQNFDFKKAIVAGWGLTSDGGSQSHFLREVEVPVLTNAQCRATSYKSMILKTMLCAGYLEGGKDSCQGDSGGPLIVRDGIFRLAGVVSYGYGCANPNAPGVYTRVSKYLDWIAANTRDACYCVR*

>Woma_00008947

MYKILLVVYLCLSPPLEAFKQRVRPRQTNVCLGHKRGEFAENPKDCRQFYLCGDEGEATLATCPPNMLFNPLTKLCDKPENVQCNDKPISFAVPPAEAVTTTTTDVPSKADQYCFNLYAIQRNTYALVFLPHSSNCQQYYMCYHGQALLQACSADLHWNSKLGKCDLPTQAECSAEIDTATVYQDDLNALDEGVKCPLYREHIFPNIERCDSFIYCVNGQAILQSCPFYQHFDVESGRCKWRIRARCIKDLNLKYRSYTL*

>Woma_00008949

MLKTVFACLVLTICCTAIVYADYFEECDGNDGTFISVPEDCSFFIFCDVEDSYKDSCPEDNPYFSLEDLTCDTDRKVCGDRPFPNSVLKEDETGAVTESNDTTEPTKFVSVISTSSTPNTSLISPESSTSNPTTIESSSSTTFYISSTPSSVPFACPEVDNPNKAVFVPHPKSCSEYFLCYQGQRLPMRCSHMLHFDFRLQKCNYAENVKCDVKDVISPRDQCLPFTYDVYPHPSNCNYYYKCSSGYLQVMQCPLNMGWHYEKRACMLRSQVKCYNSRRI*

>Woma_00008996

MIHHYAYIYALSLLCFLGLQNFVQGEFNHEITDNILLKESFLCDNTHMETQLETLKLQMEIMQEQLNKQTDILNRLDKKIDEIPRQMQAMQEKLLQDNKFNFEDLKYSLHKQIQNEVRAAAQPQVDDKTFILKDIKEILKDHDKALDNILSNQNNTEKLLRETIISNKLAILEKVEGKLVNIENDAMTKTEQLKFRCDVQRYLDKNEVTKNTCKGDYLNLFCNSASYPTNCADFNESHCISNKCCISNEIYGSAAFLVSCNNKEEGGGWTVIQRRMDGSVDFYRNWSEYKNGFGDVNGEFFIGLDKLHTLTTTLKPMELLIQLEDFNNTKKYAKYDDFQVANETENYKLIKLGSFNGNAGDSLRIHEGYGFTTIDRDNDLADRYNCAVRFSGGWWYRKCYNSNLNGKYFKDGDKPISRNGIAWNAFRGDEYSLKFVQMMIRPKGVKISYKIK*

>Woma_00008948

MFRCILQAIVVVIGVLSTPTVSVEFPECFNQPIRTFVPVKEDCTKAIYCNGIDSSIIECDEEKPYFSALEKKCYDDITVCEERFLTENQPTTLGEEKQTTTTTVRTTTQRPTPAILSTIPSTISTQISTIIMRTTEAGFSTNKPTPPTIPSVPIVSSTKRTTTTTEAISTITPSTANLSCPLLDDPNRPLYLPHPQSCSQYYLCYYGVPMVMQCPKLLLFDVIQQSCNAAESVNCQLSSVSAPPTKCLPHMVMVYPHPKNCNYYYRCEFGYLKVMQCPFGMGWDFELAICTQLTKTKCYKPANGFFN*

>Woma_00008932

MRLNYIVILLTALMGLCQGAWDGEIKPNIVIILADDMGFDDVSFRGSNEFLTPNIDALAYSGVILNNLYTPSMCTPSRAALLTGKYPINTGMQHYVIVNDQPWGLPLNETTMAEIFQKNGYFTSLIGKWHLGMSRKAYTPTLRGFNQHYGYLGSYVDYYDQTLEQFGQNYSRGHDFRDNLRPIHEGNGSYVTDLLTEAALKCIRQHNYREKPLFMILSHLAPHSANDDMPLQAPDEVIEKFAYIDNPDRRVYAAMMSKLDDSVGQIVKGLAEKGILNNTILLFLSDNGGPTQGMHSTTASNYPFRGQKNSPWEGGIRAAAAIWSSKLEKLGTIWKQPIYIADLLPTLAAAANIALDRDKLKLDGLNLWPAFKYGYESVEREILHNIDEIFNYEVYAKGKWKFINGTTLEGMYDKWLSQRSTTEIDPRHGLYEELVKSTPVWQELQKFSPSTVNISELRSSAVIKCLYENSTEGIECNPLESPCLFDLDMDPCEQQNQYEKYKNSKILSDMLERIAFFRENAHAINNKPTDYRCDPAKYGGEWTWWEDILEGNTASVTGNIEFKKNSIKKPLMNLNLLTVC*

>Woma_00008933

MKRTLLISIVLWLKITMILAKVNKGPPNIVIIMADDMGLDDVSFRGANEFLTPNIDALAYHGKILNRYYTPPMCTPSRAALLSGLYPIRTGSQHFVLFNEEPWGILENVTTMAEVFQANGYSTNLIGKWHLGMGRKEFTPTFKGFDYHYGYWGGVIDYFRRRSKILISNFSMGYDFRRNMNLECAPPGSYVTDLLSEEAERVILNSKKEQPLFLLVSHLATHAANYDELLQAPQEVIDKFAYIPDINRRTYAAMATKLDESVGKIVKALHKSKRLENTILIFYSDNGAPSLGLYNNTGSNWPLRGQKQSPWEGGIRVAGAIWSPLLKNKASIFQLPMYVGDWLPTLAAAANISLQPFNLSLDGINLWPDLVTSKTNTLVTKNSEREILHMLDDIFNVKSYMKGQFKYIEGTTDNGQHDYVLVPRNPNVTDSREQNYANIIKNSEVSLALKKYDERPLTATKIQNLRQQANIQCGVLHAPCNPLEEECLFNIWQDPCEENNLAKQPLYTNILNDMRNRIKELEGQQAAPCTGGEDLNYDPSRHNCVWTNYLDEPVSNVILECDYNSPPCQGGIESVTHQS*

>Woma_00008970

MCLKNSILKILLLTLIFIIYGREIEAQRNVGGRGVLRRNYTGKKPVVIQHRTPDAANYYDHENGAKIIKSSHFELDYTLGRKITFFCMAEGNPRPTITWFKDGAELYQHRFFQVHESHIDNNIIKSKMEIDPTTQMDAGYYECQADNIYAIDRRGFRTDYVMVNF*

>Woma_00008950

MLAKGKALFLLVLIFESSETLRKHMVKRQNNHCANHSMGDFVENPDDCRLFYWCGDNGQAISASCPPNMLFNLDSKLCDMAASVKCKQTTHLPPVPTTQATTAADLSEATIQAYCTNAYAQQPNSDSLIFLAHPSNCQQYYMCYYGQALLQQCSSYLQWNSRIGKCDLPTNAKCKPNDGSIQLPITPPSASGSTASIDDDGIVCPSFGQHIFPHMQRCDFFIYCVKGYAILQRCPFYHYFDVESSRCISLKRALCIKDLKLEYRKNTF*

>Woma_00008954

MKFIIVFAAVLVTAKAGQIPIEATVHQALVPVPANDLVPPLLENVASVEQATVVNEVSKEHDHKPAITEQPVTVQPEEEQKDVTTITPPTTPKQTIIETTRLTRAVPYTDNEAIKPQLATDVNVIKNILNQRKTQKVLANIPSRSMRSVEQEIMMMVTPETVAVKTSADTTTTKKPSLPPLMNPCDLLCTKFEFDPICATNGLCLHEFPNQCILDTFNCKNPSQKFSATKDDRCQMHWLTKCNEQDMI*

>Woma_00008955

MKVFVVLVSLSLMLLINAKDVQKEQLGHDQHGHVVLDVDKQGVHVTHVPIHKGETVQNNEVVTEAVVTGQSVTEHNQHVEQHHHVKPTVEVQHVDTEHPEVAVQIPSHVQHVVTQVSQHVPVAVGQHAAKQRAVRSVWRYMQQKQIVEKPRKCDFGCSELDLPICAYNGRCHREFEGQCKMSAFNCLNAHKIFHQVEDHMCQNPDTVKCYPGDD*

>Woma_00008956

MKVFVVLVSLSLMLLINAKDVQKEQLGHDQHGHVVLDVDKQGVHVTHVPIHKGETVQNNEVVTEAVVTGQSVTEHNQHVEQHHHVKPTVEVQHVDTEHPEVAVQIPSHVQHVVTQVSQHVPVAVGQHVAKQRAVRSVWRYMQQKQIVEKPRKCDFGCSGLDLPICAYNGRCHREFEGQCKMSAFNCLNAHKIFHQVEDHMCQNPDTVKCYPGDD*

>Woma_00009217

MKLTIFVGLMALFAVIFTADAGRRYQFVSAKCVEHDTQNQVDGPLKICTFPPKHQSVPQEDINAIIKHIQTLHLK*

>Woma_00009174

MTKYLITFVLLAFLALENNHIYNRLHAKSHPSGTTNTVTHFMRQHHQYRQHSTHAHNKHYPTHEQHQTHHSRKHINHQKHNFDRGQEEDDSVQDSTRQVERIKRHMKQQRQHRLEHNKILQQKSQRPTYSAESLKTMPRIWQHLSMVDDYDYSDDKDRMYGDGPVEDIVLDTDESNIKVEIPMTVEENYEVEEDAVEEEEEEKEIENIENHHTTRCPKCESNRKVEHVTEEELTRLRIEFVKQQILEKLRLKERPNVSAVGLPKPIYEGVTIEQEEDASDKKDLDDYYARTSKKFIFLEVEKTECRKLGSQPSMCFSFKIDDADADGYDVSTAVLWLFKNIPQKMLKRNDTILSQQTIVVSEVQQQLDSKYLPVVKTIAIQSVDAKDEWMKIDIEWPIKRWFGNHDLSHLIQITCQSCDIESMEHMISTDKDYRPFIMVDTQNRKRQPRQKREINCTDGVTECCREKLYISFDDIGWGNWIIQPRGYDAYFCRGTCGMVASISESLSTHNTILQKLLNKPGRRRKNLELVPCCTAKQYSSLQLVFMDSNNTATQKTFPNMVVESCGCR*

>Woma_00009171

MERKQVCVITLLLGILIFKLNTLIVVADQDEDIPHNEVRNWALKFGVDLWEFGRQFTKMNEIKSNIYSQEKSKKQEGVQHKEEQLCVRV*

>Woma_00009205

MDLINYLIVLVLTTFTLSTAPPIEVVFLIQSQDTRYQNELSRNLKEDIIRQSFELHPKYRIKVHVLHEIFNYPGGWTIHNLIPKLMLFQHKRHMNNDDNMNRNANKQIEMEMEIETEMEKAKAKEKDIINDNTRWIVFCEETTKVKVKLLMDRLNNENHTQPLYLGHPLYDREPTIIHHFSFFENPKWFPYPMLRAGVVLSWPLFKSIADVYTNHNSNSNSNSNKRFKQATTLLQHSEFSIDAAHELARFIYDNIQIPTTHTTTISLTDDKNNKNRNENVYHHTKSVTVAATKVSPQITFNVKDYDKKDSDLPHSVLDSSTALANEIIAKYKDDTIESNSLNAPIRHFTTTNTVTWDTSSKDNGKTCAMYAEQTTATAAISSSASSVTCTPIKREDIYFAVKTCLKYHSERLPIIQATWAQYAEHVIYFSDFQDNTIPTINTGMPNVEMGHCDKTLQILKLALKDIEKYNSNNNNNNNNNNNNNNNNNNNDNNNKNENKYRHQQQQQHLRYEVNQAKHEKHQIKWLFLTDDDTLLSVSGVCQVLGCYDSMDAIYLGERYGYRLHAKDGFNYITGGGGIAFSLPVVKLILQHCSCPSPTAPDDMILGSCLHSLQLKALHSSRFHQARPNDYSIELLKLEPPVSFHKFWQLDPLEVYKTWFEVQDSQLLDEEQLKENLLAMEHRTNTRQLLTKPDSFNFNEIPVAFVASLHHSKERHVDL*

>Woma_00009268

MWTNEQQISFDKLKQLLASPPILQQVDVNKPFFIKSDASNYALGAVLIQEEKEEKHPIEYASRLLLPAERNYSTTERVVWAVNKFRRYIEGAEVTVLTDHQPLKWLFGLKSPTGRLARWALQLQT*

>Woma_00009249

MKVFVLVLAVLALAAPTPSLARTYNRCSLAKAMYDLGVPKDQLARWTCIAEHESSYRTSVVGPTNSNGSNDYGIFQINNYYWCQPSNGRFSYNECKVSCDALLSDDIRESVKCARKVLSQQGWSAWSTWKYCDGNLASIDSCF*

>Woma_00009182

MTFKFIIFLSLIALASAGDYEDSKPSSANNQSSGNYDTKYNGNDNKNKHLVDPTPVQHDDSYQTNTSNNNGSYDDVTSSSSYALSIDNTNNSADAQAYQASNSDSYRNSNFGYDFNTVAYNPPAKYEYSYSVNDVTTGDVKSHNESRDGYYVRGVYSLVDPDGYKRTVTYTADNVNGFNAVVNREPYAIQYTLPRGSHKAPGAVVDESNDKSKESQYNLVNSLQEKDSYSGHPVQDVKGPYA*

>Woma_00001709

MNAYQEIEERFNFLSQLTTIDSELLRQKCNQFAKYYKIDINAFEFESECFHLRQYIKNLTNEPTNFTILSMHKHIRSNNLEETFPNVEVATRILLCLMSTNCSSERSFSQLKRIKNELRNSMLQERFCNLSIMYIESDVLQKIYFADIIEDFAAQKSRKKILS*

>Woma_00001706

MRGLILVFAILACIMALNWTAATEQTVPDNEAATTLLDVDTGADQQNEGVRQTRQFFGGYYRPYYGYYGGYYRPYYGYGYYRPYYGFYRPYYGGYGFYG*

>Woma_00001707

MRHILWIFCIGLLICHLNIIPGECCATVDNKDETDPKADGDIQEQTVNDENQNLNNSNDHHAVDNFENEDVSAIAQEKRVTRQLFWPLPFIGGFRPSFGGFRRGFGQRCIIRGHRFG*

>Woma_00001705

MHKKFVIVLMCIFALTHHLPKIFASASDLSFSTTSSLPSLSSSASSSAATSSSVATATADVKLPQSSGGGDASAKTVSIAYDDNSAPAKEISIAASSTSLSSSSSPGGEAGQENLATRRKTRSLFYPYRAANLYIGYGAGSAYYYPSAYSYAYYPAFYKYWGVYNPYYGYPRVIPV*

>Woma_00001728

MFQFSTTIGLFLLVGQIWCLPLIPQIGDEEEFIEVEDSSLNEPETSKDVIDLSFYGTAIYGDPDDKYTAQMVANYTPEMYQVNPEELGTYLEGDILIPRQRLIMKNGITSQSSRWPKGIVPYEIRGNFNARDMSIIEHAIGEYHRRTCIRFVPRTTERDYVSIVSGNSGCWSSVGRVGGRQEVNLQSPGCLIKPGTAMHELMHALGFLHEQNREERDSYVSIQYQNIQPSAMSNFDRASRTLAFGIPYDYGSVMHYSANAFSTNGRPTIIAMQNMNGAKMGQRNGFSAFDVEKLNKMYDCGYGGPSIPAPIPAPAPNPLPSPAPAPNPFPSPSPIPSPGIFPSTGAQGVDNNLINSFISGILTGLGLGDDPSA*

>Woma_00001729

MYKLPAILFVVLVGQVLCIPLIRQIGDDSEIIEVSDNSLNESEHLKDVIDLSFYGAAIYGDPDDKHTAQLVASYTPEEFSVNPEELGTYLEGDILIPKQKLIMKNGITSQSSRWPKGIVPYEIQGNFNARDMSIIENAINEYHRRTCIRFVPRTSEQDYVSIVSGNSGCWSSVGRVGGKQEVNLQSPGCLTKPGTAIHELMHALGFLHEQNREERDSFVSIQYQNIQPSAKTNFDKASKTLAFGVPYDYGSVMHYSARAFSTNGQPTIIPMQDMGSVKMGQRDGFSAFDVEKLNKMYKCNLSAPAPAPAPAPTPSPAPAPITAPGTGAQGIDNNIVGSFVSGILTGLGLGDQPNV*

>Woma_00001840

MSCPGIKFCLNSWLPLLLLLANLVWADIVVQLNINRPIYDVSDKFISFTLKPEDLYKALDGSNRKTITRMATMLSHAHVKFVGDYYFATKTNAIRLRNPTKIIWKGFNKWTKAVNWTMIIPVPYKPNDWDPMKTLTILNTSHMVGIDEAIWQFGTNFGSSRASDYVSELKTFHIMVESFRSSKMNWQTMGADISAASSPDETRRYVDMSRDLNVAYGWIQPDTFWSNSLDTLLGEQDPALRVLLKENVPVWLSLPKRQNILTRLDDDVVMEGLIWAQTMGDAANVGFETIFKPLSLEDFEMPKYDFYVTALFKKIMGSRVYPARPLGLLGRSNKLYSHCANMVSGGLAFMVVNKNEQTLQIAVRPMSRLQDTEVWQYALTMTNGKIMLNNKVVTVNSTLIPEIKYKPNKSALQLRTPGRSVSFWVLPNANLEHCHFTEVETTIVPNEEHNYKTKQYSASDRLLKELIKESAKPSYLPLAGLFRRQRRSVDNEDRFKRFVLKGAEQENYPLVSNFAENIMGDVKSPLKRDIFSPERRLAALQWLSNIFKEPLNFELPQLKRNARQSSIYGGPFFMTRDGKKTVFTKKITDIRKPEKKKIPKNEMEPQEFDEEKFFKNVDEQAEPEYVHVPEGDVYLTNIAPVDGDDNEEVLRIETGRQQPVEPVNEMKPLRLLPTEFFEALPARPIEPVNTPMLNLGTALNNLLLVDPFTKGYGHFNADQYNRNYLSSGVAEMETDKDLDVDNDASYNEADDFVQAQIDISQQAALNHENQEQQSKEEKYLQSETPHTNAEALLDATEPKMQEHIEDFSANFLNTHDSNVFEDASMAETNNEDQVEHDASQTKSDETPWWDLSTKKVKRSLDLRYPSDSWHNNIIPKDNELLKVSNQLSPMRVNQFEQKVNDAVRNTDVTSKNNIDTDESVAVKLVKTLKTQVSKLIRIVSQHVGEIYNTLTRHSDTSSDETSKTQSNRIS*

>Woma_00001727

MVLNLILLLAVIRSFNYKVSCIPMSSDHYSGLLSQPTTDIVDDSEIVEVASNQIPEDDESNLIDLSVYGLSLYGVPDTNKTGQLVAKFEPNKTSVNPEELGSYLEGDILVPQNMLMLKNGLISKTTRWPDGKIPFEIAGDFTKNELEVIAFAIDEYHARTCIRFVAHTTETDYISITNGDSGCWSSIGRVGGKQEVNLQSPGCLTKPGTAIHELMHVLGFLHEQNRQERDTYVKIQYQNIHQKAVPNFEKVPKTAAFGVGYDYGSVMHYSPTAFSHNGKPTIVAKKPNKNKVMGQRDGFSPMDVEKIYRMYNCKANRKPEEPPIILPQRVLSSRFPFVESFVNNTFGKLINNIFSGLSSIG*

>Woma_00001801

MNLTQFILHLCLLAICMNTAMACHGKLLAAGITEEEKEIILKEHNRLRQTVATGRYPGQPGAENMREIVWDNELAARAQQWAENCQFRHDPQRTINRFTMGQNLAIIWSTAPLEPDDGDFPSRIQSWFNEVQKYSFGDAWSPKTGHYSQLVWGETSLVGCGFAEYKDTSKYNKLYVCNYGPGGNVVGYKPYEVGRPSCATYGMKPSTRYQGLCVAPESQGFGSANAIETYANSNNNNNKITTTSFVNVQQTHYQQTSSLSSTATKKVSGYGGHSFGSKTHGGSHAYTTEISQQQQYNKYRNKLEDYKPSPEEFKRAVYKHTVLRTSLDQQQQQHQQQKQQQQQTTLQPAKNGWSLLSWRG*

>Woma_00001826

MMEQLVLKIFCVLAALFVNNSLSQSITFPEVSCFTPNHLEGVCIDLLKCPPLLETLKNVNRSPMETRFLQQSKCGQIGTTVLVCCRFDGGNRVLIPQPTSADNSNLLPNIRQCGRSFDNRIYGGSSTKIDEYPWVALIEYTKPGNNKGFHCGAALINDRYVITAAHCVTGRGVPPDWRVSGIRLGEWDLSTAPDCQISATGERDCAHLHLDVGIEEIIYHPDYNREAANNNYHDIALIRLDRSVQFTDFISPVCLPVQPNMRSKTFENVRMDVTGWGTTEGGYSSPLKLKAAVDGWTFESCRQKYATKRIQLQDSQMCAGGEKGVDSCSGDSGGPLVSKERIENRDVYVLSGVVSFGPKPCALEGWPGVYTRVGAYIDWITRTIRP*

>Woma_00001878

MTSSTPHNNNNRPHRQASEREEPYTQLTPHIPTFNLIESDDNEDSDNATITATLNGNQNSKDLKEFMERLPDIGTPNRKDLVETAAIKTNDNLPADKCRAREESNM*

>Woma_00001781

MFYLQFKGFLLFCIIVVAYGTYRVQCEYAYKQILKPNTPSMIQIIMQDMQQAFCSITNNTQLIRQMYDFETASSDVSTLLFKLPNSEENPEMETLVLMNRRDFIQPMFPNLTESYMTLRRFKVLKTIISENTDPNVKQLLKQEMLSLTQEYSNDAWSDMGLDGWKGDIADSKLPANHKNPQVLANDYPQIEITKDLSKGDVNKYFQDINVIEKTEDVEEREEMDRTVFIARANDPFGYSTKWHMRNSSSQSKHKRDIFRLYSMIKCSTGCEPLVYKGYGCYCGFLGNGIPTDGIDRCCKLHDKCYEHSNCVSYLEYFVPYVWKCYRGKPLCAIDHGEWGGPHSCAARLCYCDLRLSRCLRQYACPGRRAVCRSSASRRLQNLLFVK*

>Woma_00001806

MFKYFIIVMSLLIMSVKTQDMPPPDLPIIPLTTTTTEEPKTTTPEIITTTTETATTTTATTTTEISITTTTEEPMTTEEPATTEATTTTTTTTEAITITTDVPLTKTTTPELSTTTTTSPPTTTSNSTIGNDLPITPTTDSPFTPTRTTPIYPGHPSYPHYPHYPAYDRQCYFRKQYGYAQLRWTCYGLRYVYPVECWKCCRYQYSHFAGCFRVADRNCNMYKIQCVKIREYLRGMGRGKPLSPYEQGQIVALHEQGLSMRGVMKKLGRSLCVVQNFLKNRENCGKLKITGRLKVLDARDMLLHHCCHPNNPKIVF*

>Woma_00001890

MLPRIYLILFIVFGFLLIIDCRETSKRTKRAKRVRAPEPVHFEPEPHQDLESDDSGSQEKELPEIPTNFLSPSVREYLELGKSIPGRPGVDYPILSAIPYTNFYCDEQPYPGFFADMETRCQGWHYCDIDGRQASFLCPNGTQFSQAVFVCDWWFNVRCDLSPRLYAINARLYQRPKVNPTRPHRIITKELVDDIFN*

>Woma_00001856

MTRKRSSPMGLEIYLPVLLAFIMLPLLTQAAPLQDYTEIQQYTEGCYYNYNHYNEGDRIMTNEPCLNCTCHNKMLMCYLRVCPFTKPIGHDCIVEKREDQCCPIITCPEVPVDVAHATPEPGTELSFPEKFGCSIDGKFYLEGAQVPSNPNKPCELCYCIKNRTSCLMQECTLHIDGCTPIYNKGSCCPVRYNCDHENDVLELEDHSTSTEAATTTTTTTTERPTTGFIIASTMSPSVSTDCVHNGELYADGARIAGENACENCYCMRGDIICAVQECHMPMLNGNGKSCHALPAAEGECCPSNYVCEDDSSTTQIIEASTLPYDGVDEDNKVTTTPPAKDLHSGIPEEDLQLQAHIDDEETATKAPATESETEEEDKKDAAKPESVTKSSEEAEVTTSASIGLHEPVESDESIIKPEDESKIEGPETQISEDPIKKEQDQHISDVKPASEESIAADVLPEISTDNIASGEEDDQETSTTEKSEIVDIATKAPAADDLETQEVTKESTESTEEGETKPNVEEEYATPSSITDDEVSSTKVPEGEELHEDVTKISLDMEQAIGEDDSSTKAPAAGEKEDKTEDHHKDVTTDSLDMEQATGDFEEVSTKAPLAVEEQEMEHVTETPKESAEKQEETSNEGSGEEGFEQPIQAPASEEEKEPQPIVTEDNFTVTTVASENVKDDTSTEGPKKTAKPEEEHETITESQEESKLPSEDELPTTVPAIEKEPVPPVDMSTESEKSETATATPVSADKDQESVTISPHIEEEEKKDESSEYVPTEIPTFVTSDSSEEEIHGSTDASPEKEDEEAPGEGKPLAADKQEIDRVTETPEQEHAVTSTLFDDEFGSGDHATEVSIAIEEASQPAVIASTTEGVTSVPESQDKEKEHVTADVLVEDKLSEGEVLTTSTDISGESAESEEDTKLMDLTTAKTSVEHVDATTIKTPTADITTSAAETSEEPSEEEQSEYTTVKTIADKVSTEEQPAERPADKVEFEEATTQKAIEDTIQETTAVPTAAVGHGTEEKEDQNKIVPESGDHTAEQPSGITDSPMVVTEDAETQSESEADKGHEIETTSIFTTKVEEEAASTTVKPFIEPTHFEEMPDMQFPSVIPGEGDCLVGQKTYQNNTIVPTSDECEIVCKCVSSIVSCERVICDIPENIEKCVLDERSTNKCCPSYICATDILPSKTDDSTESTEQDEDKENVYTTTAASHKLPEQPAVAEETKEKAESTTVKASVTAEDVAPGINIPEIEMPTASTEEPMSHVKDEEEEIEPEATKAPIEVTAGDKNYTYGESEVHTTPSAVVEDVVVPQSMAPVETSEHDQAHIVTDKTPVASANKEIEPEISTGLQPEKELHEDTTASVVETVTDSAATSGLPSADEYQTVQPIEEEPEEASGELIDNTGKPISGQDVDTAPASEEEKDTESETQTSIPTSSEEETAHHVSSAEKDSEIENQTTVASTSFEEETADQISSAEKESEIENQTTMPSTSFEEETADHISSAEKGSEIENQTPMPSTSFEEETADHISSAEKESENENQTTMPSPSFEEETADHISSAEKESEIENQTTMPSTSFEEETADHISSAEKESEIENQTTMPSTSSEKETSGEGESETHTAIPSISSEEDKASETPEEHTAIPAISSEEEYKETSQEAEIQTPTPSELEKETQTSKPIDTPVQPTEEVIGEPTVSSAGNKHEETTETSTSVPIKDIEKIEEKPSISESVEDVSDITADSEEDITRTTGVPSIVEAEDHGIPSEHTVAPSLAEEEPTKLLESSPSSTIAPVSEQEQDEHKGTIAPEETGEDISATTASAVSPIDEAEEQKQVDAEMESSTIIPGPSDEIVESVTPLEGEEYLEHTVLPAVHDELKETSDGSITETSTSAAPVSESEEDMKQTPVSISEEQTIAPTSEDSGEESGEKDIKATSKPISEETSTTTTTDVATEKPVDFETKIDTSSSVPSLAEVQTSIPSQEDAATTVSAIVEKPEEEKEFGEDFAQATATDKSEQDQTESTPVVHDQKPTDIPTSSEFVTPEDKVTALDEDRNEESVTIPSIEEAKEELEPSVAHTVSSPDQEEKEHVTKYPSVTDDNKPEEGIENVTKESTISLIATEEISTDMAPVEKDEFYNQKPVSPAEEYIPTVPSVEVESTHTPSKQPSLDLSEEASGESEESEEEIGTEPTKIQHVGEFVTERVEEATVKPSETEVSSEEGKETSSEEDETEKLPSSTKKPYQPTDEESEPVTEVEDVELSADKLPEDDISPGITKAPEGTTAENVTAQQPQEAIPSATMSSIDEHDIEDVTAQQPEEQDLLVTSVTTAPFIEQEIKDTSDQEPESVTTGPQSSENIDNVTAHELESAEEISTEQGTSQIPEISIVSSEQPAIEGMEDIRESEEGLSSVTKSPIAVEDTTTQQEGKDTLAVTNAPVGVDETETVTAKQPEEEEKDVVQHVTYPVKQPQEEDIVVSVTKSPMAADEIEHVTAEKEESISSTVQTSVASEEAINVTTQPDEIPGEIDLTTVKLSTEEEADKTEETISQESQGEHMETATSKPQIFETIDEAKPSEEADETLDITEVPESLAEGTETTTKVPVAQQADELFDVTASTEDQKESEEEDKTSEPTIPGQEQEPVEATTAKLPELADHITESANKDTESEETKEESPEYSTQLPVISHISTETSTKVTEAEIQAEPTEMPSRTHEELEPAQHTVTDQPTHATEFEQEESTDAHVLTDQLPEAEKESNEDATDLAVVDTESTEKLPEHVTEIPIASSDSEDKEKSQDKTTEVSLEADKLTEIVDHATDMPIKHVESEEVEKESSEKITESPFGEHVSVDELPEHITESEEESTELPVEAHVSSELPSEHTTELPTKGTEQKEEDMEGHKAATEKPTGVDISTDKLPEIFEHVTELPTEIAEPEMAEHVTELPTESAVVEDEEKISHDESTKLPESVTKVPVKDTKSETTETPDKLSEIPEQHVTETEAEKEESHELSEIPEEHVTDSEAEKEESHELSGIPEEHVTESEAEKEVSHDEITELPIVVPVSTDKSSEVSTELPTKDLESDEKPHELPVDAQKIPTKDTKTEDEITEMPVDHGLPEHEAHVTETPARDVESEEDVHGEATELPMVVAHDVSTVGQAVDQSTETVSKEEESYEETTSNETAEEITDDRLLPTTTTHPLFPHLATTLPTVAIDSRFDDTNLTSTAHSTAAPATLTSSTTSTTSTTTPEPITTTAPHHYQPQPPIYGQPPQYGPTQYEDEYTDEDEGEVFGPGTCRYGGKLYVSAQQIPRDDPCDFCFCFRSDIICLQQSCPPPIAGCHEEPISGFCCPRYECPVSMATVLNITTSTTTTSTTLPPHFLHHSYGNNVQRNGCLINGRSYRVGERIESTSGPCINCTCGGDGKMKCDPQACVPEPTMQQVMAVVAAGRKR*

>Woma_00001698

MRSLTVLLVTLVGSLCVSCEPIPPLNKDKTKSSIATISFEKQLKETDVQQFNAEIKNVPKLHLEDGLKELVEESAAYLADNESLKKRATRQLAVTASFPYTFYPYIYYPTVYPASINYYRATIFPFLGI*

>Woma_00001873

MLLLGIFLLGIIFFVYQAVSINQLPNTSTPWSLEGTMQGLKRKHKQLQASLGKKQTDNDNAVGAGAYDEGGGGGGNSGNNNENVKTSPDAIKIIKGIRLFDYDSYKPNFEGKFRCLDGSLEIPFERVNDDYCDCVHDGSDEPSTNACNNGRYYCRYQKRHITGRGRDIFVHSSRINDGICDCCDGSDEWQSTTKCKNICL*

>Woma_00001810

MKFNELFTLLGIFATVKAQYNQLEQLHCTIPKILQGSWFSWENGLPTQTVIDARQMSKRGYCISMEKHHGDEYSFVFEERGKGCYHCVRTFIRTLNVFEKFESPCVTLPEGDKPSVYNVCRGIKDDQQLITLFNENFIPINCRSSLEGVWHFTYQNRFRFTGVCDKPDARIQSCQTAGTQFLIQNQKFNITYQQCEGMDGTFTGIVEYSCLGDWFVGKNHYFAVANTKESRKDEKYRCFLKNRDDDLYVGVSITAECNTLKTPEKSPERLKLTPVKAEYVEPGCTLPQNFSGEWVNTANIDADVTISETHINETYYPDKARYRRTIYVCRERRGNRIMMARLTVDGCQKDYVCFDFMPRHHNVIRYRKGLAVIKDDFSTVCSWVQFKNDEAWKYDLFLAKNPVPVRCPVAGKFNFTQRGEHPFKTRILGGVTLSPRPDIHCKQNISDLSVCDTDQKAMAIDENYCLSVDHLGRPVDIYSDPDYRMKCIGFWKENLKSYLITYDDLDPLSKYRCWVYQRADLNRVLMSQAVGAFCKLNQDVTSWNHSEGAAVAIDAIEYERERDDCPMYFDDGENPWLESDASNIVFDWDFYKASANHLEPILNFKTLFTFTFGFVLREILRLYV*

>Woma_00002096

MSKRLFVVAVIVLFTFPISVVYANDKKSHKPKDTESCQEIARHDIEKYLSTKTPYRVVANFNDKPKKYEGCQPLRIWSVIRHGTRNPSKKVIEHINTELRSLRDNILASKDIELCPKHLEKLRKWNFTVVEEEEKYLVAEGEDELIELAERMQNRFPSLMPEVYTPKQYFFKYTATQRTLKSAQSFATGLFGRNRINEIDYPEALHRDPVLRVSITEYNIKFLIILAGKGAVHDFLSLHQFTCNHFLKSGKTLSQM*

>Woma_00002091

MKFLNSLTQLIIKLSIFVAICQTHPVLKSDVSYNAEHTAPALAHQTVLSEDLALHSQAEEGSTDSGFRISLFKAAEKTRESVNLETEAVPSKILSLYDNSHKKFVDKSQPLPILDTISEHEKYGNSGDMFDGISRSLVNGFEAFSNLLNTLIQKPKEIARSVSKGITAQLDVIGGKLVGL*

>Woma_00002182

MQDKRFTNFDVENGQSARSPLEGGSPSAGLVLQNLPQRRESFLYRSDSDFEMSPKSMSRNSSIASERFKEHEASILIDRSFVNAFYNEISNTYASTVTQHLFTKT*

>Woma_00002184

MIATLFIIFAFIRAFPWSRRRAYTGDRPNIDDTHNHPPSLIQNNQNGQHVVPNLEQQQSSLTTTTTNQSTPLTSAPVDTKNFTSTTTLTTRTLTTTATTSSTSSQSVNEIVPRKVSSARRKSSKSIKAHEVTFSGSVGGGDSDSERTPTSSRSNRLVRQPRSYTGPVTSPSRATSPESNSSSNLYRSALSSRNQLTRASSEYETAVSDYYTSRSRSSAERELPESNGIQRSSSNGYLKHSDTLAAHSDEEEMEHERLRGGTGAEADNEADGDDSYDRDTEEFYSNIQDATGTSGTRSKRSSLFSRSDSSATTTSSSGGTFTGGKRRSTASIISTSMCSEIIPIDRRRSSTATEYSVRSLTSQPRRLSGRIRRHISRMTIAGARRRTTGRN*

>Woma_00002186

MIATLFIIFAFIRAFPWSRRRAYTGDRPNIDDTHNHPPSLIQNNQNGQHVVPNLEQQQSSLTTTTTNQSTPLTSAPVDTKNFTSTTTLTTRTLTTTATTSSTSSQSVNEIVPRKVSSARRKSSKSIKAHEVTFSGSVGGGDSDSERTPTSSRSNRLVRQPRSYTGPVTSPSRATSPESNSSSNLYRSALSSRNQLTRASSEYETAVSDYYTSRSRSSAERELPESNGIQRSSSNGYLKHSDTLAAHSDEEEMEHERLRGGTGAEADNEADGDDSYDRDTEEFYSNIQDATGTSGTRSKRSSLFSRSDSSATTTSSSGGTFTGGKRRSTASIISTSMCSEIIPIDRRRSSTATEYSVRSLTSQPRRLSGRIRRHISRMTIAGARRRTTGRN*

>Woma_00002189

MSVKLQRPLLLLLVALSTLHKALAEQQCLENDEDCREKLMHPPLREDQFQRRSVQQEVFTEYWLDKGERFVAYKDATANRPLRNKAKNIILFLGDGMSLATLAATRVYLGGEEQSLSFEDFTDTGLAKTYALDRLVPDSASSATAFLCGVKANYGTIGVTGHVKRGDCIRSKNTKYHVDSIAKWALKAKRSVGFVTTTKVTDATPAALYAHTAERDWENDKKVKNDCGKNSDIRDIAWQLIKGDVGKRLKVIMGGGKSQLIDQDFYAEGKRRDGLNLIEDFLNENPFNKYVETREELLAVNSNTTQRLLALFNDGHLKYNLKALNSKKNKQPTLTEMTQKAIELLQANNDEGFFLLVEGGRIDTAHHSNKAILALDETAELAKAVSLARTFTSMEDTLIVVTADHSHTMSISGYAQRGHDISKLASLGDDHLPYMILSYANGPGFHNFYNSKNHYREDPHEVLDAFEGDNYDLQYPATVPMASETHGGEDVPVYASGPWSDLFSGVFEQSTLPYLMGYAGCFGPGEKAC*

>Woma_00002143

MSNIIWLSVFVSLMALCQAMPHPPATTAAPLKQTYDNRFDNVDIDEILGQERLLNNYVKCLEGLGPCTPDGKMLKETLPDAMATNCAKCTERQKYGSDKVTHFLIDNRPEAWQRLEKIYDPEGTYRKAYLMQKEKSSTTNDDDGADDDKKEDEKDNNKKD*

>Woma_00001941

MGFHMHCGLTVLLVCAYVTNIVFCVDLRKTEIKIVNDETRYTTLNPKAVLRPFKKLTHVKGNIIFSIGERIDGDRLILTYMEETSYSRLTDIEVSMWYPAQGRNGLIISSVQVYTDLSTSDAEIYFVNDGGIGHTYAEMLFVGNQTYNYGYQIYLYGY*

>Woma_00001942

MMQISKFILWCVCVKFITAASIGHEDYTYKSIKVFRESTVEQNQYDQFTLGKVEEGAQIIYMYQYTQSFVDRQPEISLDFNYPIEGSYDVDETKNLPVITQIQLYVKTEADALTQAYISSGGINENNIGLRIVTNNSNLLNYMIVIYGKKPDEVVN*

>Woma_00001901

MDNFKYFIILLIKWILTKVISAQYSTPNLHPNCILATTTTTGSDKSNLTLLSSRLGICRIGTPVYPETPRCIPELSVMIQTRKRRIINRRLYCKAKKILEF*

>Woma_00001995

MQKSFILLCLALACLTSSTQAAAYRRGDKISENFKQLELDPAEIDPNVAPLGPNKVETLEESSTDDILNASTDNEILDEANEAAAAVGENEIIDESANEVAQSVATVAPAVATTAAPAAAAPAVSEASTPVKGEEEASGLAKYCKCTNNYCDCCRTFNLPFIPVKGPGCARMTYLGNEKMSISLKYGDLTLASRTVSSKRARPICVGLPGGYSQFCGRVYGLSKAKENFKACLGFELRADDEVEASLRVSCFKFGPEGLRVADAEPLPVEVNKEEEDDDDIFGFGAGGDDDEDEDDYEEDDEEEEEPEDDDADADYADDDAEEEAPADADYGGFSLAGLLDELDDDDDEDNKPSTTPVVAPENTATRTAVVSAQSKDENGTAAAAFPTTVADSTETVAPAATEQVTPAAESTPDTTAKTKKSKKVKNNKKKKAADTEGDFAYEFLSGLLDFFN*

>Woma_00002206

MHWSINFMLPVFCLLLLTSLTTSSLSSSSSLSSYLAASSIHLADQLDANSNDIVTPEESLHQKFANSSANHTEYVRAVRESIKEYELYRQRAKPARVKRAAKRVCYGELGCFEDSGPFAYLEMLPSPPEEINTKFFFYSTKNRSDRPLMELPFLNMTEAFQNSKSYKNFNKKRRLNESEFPNVTTEAPQTTTLPPTRKTVSFRKTPITLDDLEGFDEMSVRVIVHGFGSACPHVWIYEMKTALMAVEDCIVICVDWENGATFPNYVRAATNTRLVGKQLAMLIRNLQEHKGLNLSRTHIIGFSLGAHVSGFAGAELPGLARITGLDPAGPLFEAQHPKVRLDNTDAEFVDVIHSNGENLILGGLGSWQPMGDVDFYPNGGRVQTGCSNLFVGAVTDFIWSAQTAEEEEGRSLCNHRRAYKFFIDSVAPRCLFPAFPCANYDDFLKGKCFPCAQDDEDLAEGVPRCGNMGYYADRSSGRGQLYLVTREEEPFCAHQFQLQIFNSFNDLPLRTIGRLEAILEGEGGLNETFKISEKDDSEFFAGDIVSKIIVPHPALGFPTTLSLNYKPYSGWLSKGLPHWDIDKVVLTDSYGRSYSLCKPNTKLSTGAPVRIKLQPGNCELDNQDEYGAFTSRPSPSTEFPDSIGQESINLPEADAIETIKKRKNFLNLGTSFKLDENQTYSLDDNGDLPWQPILEGNSLDKDLGESSRSFSSQPQEIFEPILNDKRLNVNHGRNLHEEIVEPVLKATTPRIKKGKEINLSAESFVSDKPQGPEFIKITTISKTDRPTKLNSETQTITVQLFPFRLGDLLQRAERYARETILPLISVQAPRFFGFNFAAPNEENIKTETRKPRYIPRFEDPPSIIKSNKNQTKSINKRKTIDSNETREQRNIFELLRPPTTSTTSSDVPQAESEQEAQESKNEVTYYTNVLRTESRFLKPENPEYEPVFIDLPTFKPPKAKSKRSIPSSSSSSFKRRLLLVQP*

>Woma_00002048

MTTRNCLILAAIIFAILQEINSACVCNEKGTDACNKDCTDLPAVKPKQKYGEKPNIPLPPVGDSCWCKSTLIEPAALPKPCTTQQKPPSSCVCKKFKSYQNAPSMPPASSYSTYSMPPAYQNAPSMPHDSSYSTYSKPPASSYSTYSMTHVNVPTAPPNDDSPPADPISISLAMQAGKAIEVPEAKLAYGFVQKPIDDQPKVYHSSVKEENLYALKKDVITWKKPNAAYKQPEEEYYPEPEDEDTEGYADEKETPSLTYKQLGYVAEKYKNPQFQQMIPVYTASYTPREPASVPGKVSKDCGYRPGRIVKYKQNKKQCVGECLKRY*

>Woma_00002044

MNLIFIALGVAIIASAKVYGYSAGSAYKQQSVADDDTAASEYQYQAAGYGAYPGIGDASSSATAGASANAGDVAAGFSSAVPSTYSGGSQNIPYQPANTRGNSVSSSISYPNNKGEILIHRPAAIIVKRPPTKVVVNHPPLVVKPAPVVLHKPPAVIVRKVFVKHHPRPVKVEPVYVNVVKPPAEKYFVNEKQQQYAPPQAGYAPINVNAGYTPLAGDVSATSGISDVGSAYASAGVDNAAANSGYQLLQNNQGLAALANLSGSSSYSQGTAPSHSSYSDPSY*

>Woma_00002046

MFRYGNLWNYTILLLLGLITTLPQLQAAISEHFPNQRYDYIDVAKETFSPRSKGRTTRIFTTEQDLDEKFWLKYLGEEFNNAVHLEVDRNNNIKPVEKNKPQKTLPKITDEKRTRFVRQPSQVKKLIPVKSQYPTEKPETFKATQTSKSRNHTTNHKHEHKNKLFLHNKYKQKIIKVKPSNTLKAIHEPIVVNLPETEKSEYQINDEQIPVVVQMPPVIYHQRADFYTRQNSNLSLNRRLRVHEHPTHIHTYTQRQINSNLPQSLQHEHNLSPEFTGQVELHHQHPRVISQLPPASYEVPASN*

>Woma_00002047

MKLKVLFLTSMIETLAMITHSQAGPMKVIYTGDTCDCPKNLNYYVQAPPPISSPYNDYKTAEYGYKLDVPLEAKGKEKVTYSFDFAVLEPKTPPPLSYQEPKLNLYAKIDRIMEHKKDCYSPTAEATPNSYDSSNSAASRYTSSESSTTRYTSAAEYIPYACGCSSSPASGYISSETSADHYTPTAESIPNTCGCSSSVASSYISPESSATRYTRNSSKHPNSIRNHMEPSTTQIMAIFFEPLQQTETNFIKPRNKRDISRIFMRPRKNEKRSERIVKQYNKPLTDQVSNAAIFEETTKPIRSRSIPLPRSSAGIRPLNYRLKHFLNLPGRLRNRRDIKGEYDLIEKERAEMDLTLPEIIQYMPETLDPNFKRGSCQFGQLCQNSKSPTDFTSTTASSKSETISTASMSETKTDEESNSNDPSTQKMESGNAIGSTHNKRNAAIYGKPCPLVLTYNNPTPGPSYTYEPLTNDQAVPRPFVSANSVYGLDPVPQLYVGAPYNPYPSADLTKTPLVGATYASPNVKSFTNAQLDQVIAEIVNKHADFIEATSHRGGALVDPILEQKEQNKSYFKQLFGSSLFRSLDERQQPSTYANIPYYQTPPLASY*

>Woma_00002043

MSNIAIWSNKANWSILLLLLWTNLQIAMTNAASIKTQQMYVWSKDVTTVKNNSLIKQFKPQIEKQQQATAAASSKAQTFLELLLGIIDTLQFGKRKIGARS*

>Woma_00002000

MDLKKHILLYGFVILSAALSCFAHTALTKIALNNSIPLDEKSYSKNKTEFLKDTQTTDSTLSLTAKKAEDITSTTPTFTDTYSVETIRDENTTVAAIVKVADKKVAHNDLSCSCGIFLSSQFVKGSSKQPKGEPIISNTLEKKFVCSPIGKKQCQTKCLEQIVHHLPNSARILCASLDRDIRKERAYLFVRNCSSKWHNTNLAAGREYCCKNGLPYSCPKK*

>Woma_00002003

MKQYFIGGLFICSLLHCVHAAVALNDARFVYNKSKTDTDTNSAENADANTNVKLLEENETDPIPMDFYRQLNNITDISEFIEKFIDPDSIDPQLGIHENIKRNVERAAVVRAKAANCIPENTVVDLTPINPKNNYFPRCTRVKRCGGCCSTQWMSCQPTKTEVINFQVYRYCYEEVKAKFCGFEMVPVEQHLECKCDCRKKPEDCNAYQRYQDCRCHCINDEAREKCLNLENKIWDDENCRCVCKQNENCTTGSYYDENQCKCLLLSGGSDVNTDDIAVQPIVNSRRRFYVKPIEVDPDNSTIYEV*

>Woma_00002193

MKFYLIFILAIIFGLAVKDSMQHTDSASSDEEYDDSGVNEHNESRISENASGNSEYSDVEISGNDNGENESENEGEVEDADTEDDGESEDSLGENDVSENTTDNNTTESSTEEIQAKKPKKGKHGKGLAHGRGGKHRSHGRHGNYTRNLAKQSRESA*

>Woma_00002190
[truncated: 313,036 more chars]
